# Dimerisation of aryl-substituted bicyclobutanes (BCBs): revealing a new mode of 1,3-dipolar background reactivity

**DOI:** 10.1039/d6sc01258b

**Published:** 2026-03-31

**Authors:** Malini George, Daniil A. Knyazev, Kamil Swiatek, Heinrich F. von Köller, Daniel B. Werz

**Affiliations:** a Albert-Ludwigs-Universität Freiburg, Institute of Organic Chemistry Albertstr. 21 79104 Freiburg Germany daniel.werz@chemie.uni-freiburg.de

## Abstract

We present a detailed investigation of dimerisation reactions involving bicyclo[1.1.0]butanes (BCBs), catalysed or mediated by Lewis or Brønsted acids. The study is supported by optimisation experiments for distinct dimers and corresponding mechanistic analyses, including DFT investigations. By carefully selecting the reaction conditions, the dimerisation outcome can be directed toward the formation of the desired product. The observed processes reveal a novel type of 1,3-dipolar reactivity in BCBs involving aryl substituents, which holds potential for the construction of new molecular scaffolds that are inaccessible through conventional synthetic methods.

## Abstract 2

We present a detailed investigation of dimerisation reactions involving bicyclo[1.1.0]butanes (BCBs), catalysed or mediated by Lewis or Brønsted acids.

## Graphical abstract

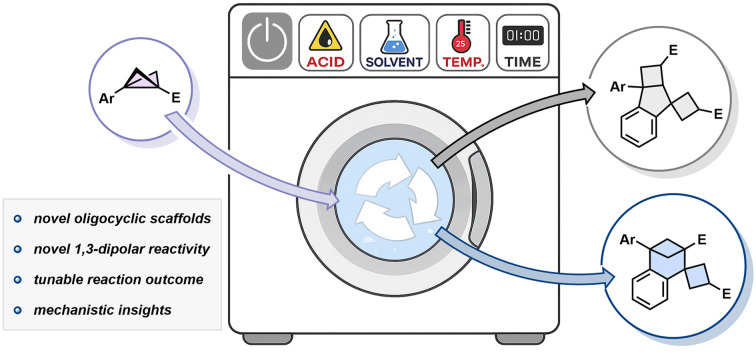

## Introduction

**Scheme 1:**
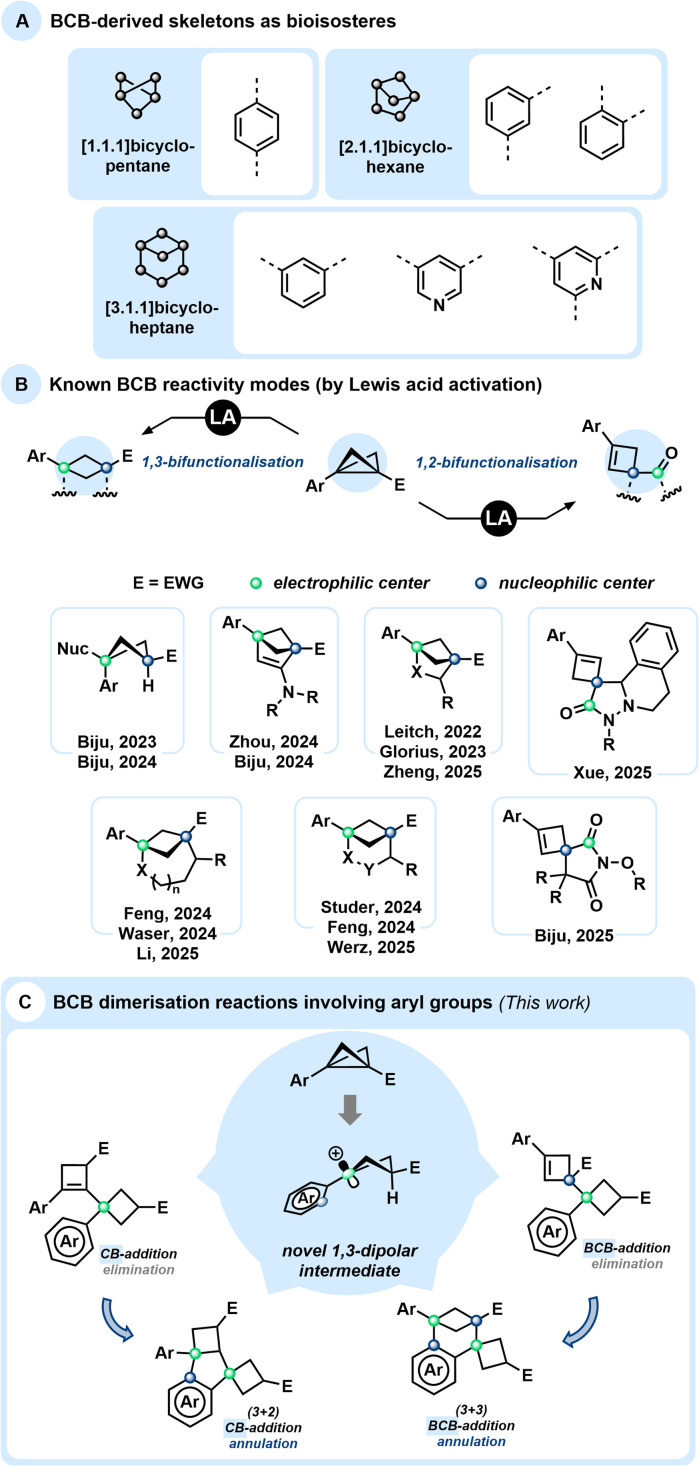
(A) Bioisosteres derived from reactions involving BCBs. (B) Lewis acid catalysed BCB reactivity: 1,3-dipolar cycloaddition and 1,2-addition with cyclobutene formation. (C) This work: BCB dimerisation.

Bicyclo[1.1.0]butanes^1^ (BCBs) have recently garnered significant attention^2^ and are currently undergoing intensive research.^3^ This growing interest is largely inspired by Lovering's “escape from flatland” concept, which emphasises the importance of moving from planar (2D) structures to rigid three-dimensional (3D) frameworks. Such an approach enhances molecular diversity, with potential applications in the pharmaceutical and agrochemical industries.^4^ BCB-derived bicyclic skeletons, or bicyclo[*n*.1.1]alkanes, are often regarded as bioisosteres of various heterocyclic and aromatic systems (Scheme 1(A)).^5^ These saturated bioisosteres frequently exhibit higher biological activity and improved physicochemical properties compared to their planar aromatic counterparts. Despite the rapid development of BCB chemistry, many reported transformations involving these highly strained molecules suffer from incomplete mass balance, suggesting the presence of competing background processes that remain insufficiently understood.

From a synthetic perspective, BCBs, particularly those bearing aryl and electron-withdrawing groups at the bridgehead positions, can in many ways be compared to donor–acceptor cyclopropanes (DACs),^6^ acting as more reactive and significantly more strained analogues.^7^ In particular, DACs are known to undergo acid-mediated dimerisation processes, which have provided valuable insights into their intrinsic reactivity and reaction pathways. The Baeyer strain energy of BCBs has been determined to be 66.3 kcal mol^−1^, compared to only 27.5 kcal mol^−1^ for DACs. These values clearly demonstrate that BCBs are considerably more activated than DACs, particularly regarding their central C–C bond. While DACs are typically activated by Lewis or Brønsted acids (LA or BA), BCBs have also proven to be versatile reagents in photochemically driven reactions.^8^ Nonetheless, activation of BCBs by LA or BA has been extensively studied, revealing two distinctive reactivity patterns that deserve special attention (Scheme 1(B)).

Most often, a 1,3-difunctionalisation takes place, typically associated with a ring-closure: the central C–C bond becomes polarised by the presence of electron-donating/withdrawing pair of substituents and serves as a source of formal 1,3-zwitterionic structures, opening access to various substituted bicyclic systems. These strain-release-driven reactions include ring-openings facilitated by various nucleophiles to access multisubstituted cyclobutanes,^9^ as well as (3 + 2)-,^10^ (3 + 3)-^11,12^ and (3 + *n*)-^13^ cycloadditions, generating a broad diversity of (hetero)bicycloalkanes. LA-catalysed additions to triple bonds have also been reported, particularly with ynamides, resulting in unsaturated bicyclic skeletons.^14^ BCBs also exhibit unique reactivity stemming from the electron-donating character of their central strained bond, which often behaves nucleophilically. Subsequent ring-closure frequently proceeds *via* nucleophilic attack on the carbonyl carbon rather than on the cyclobutyl cation, followed by rapid proton release to form spirocyclic cyclobutene derivatives (Scheme 1(B)).^15^

Since many Lewis acid-mediated transformations of BCBs result in cyclobutenes as major by-products, we questioned whether BCBs could undergo homodimerisation under Lewis acidic conditions. Dimerisation reactions of DACs have been reported in the work of Trushkov and Tomilov, leading to the discovery of new reactivity patterns in DAC chemistry.^16^ Due to the significantly higher ring strain in BCBs, the driving force for such transformations is expected to be substantially greater. Detailed GC and NMR analyses of Lewis acid-catalysed BCB reactions conducted in our laboratories^11,17^ have indeed revealed trace amounts of various products with a molecular mass corresponding to twice that of the BCB starting material (Scheme 1(C)). Aside from known dimerisation *via* cuprates^18^ and polymerisation through free-radical or anionic ring-opening pathways,^19^ no comparable study of such dimerisation pathways has been reported to date. Very recently, the Anderson group published an in-depth investigation of the bicyclobutonium cation as a non-classical carbocation, generated *via* Brønsted acid addition to BCB, and examined its structure–product relationship in detail.^20^

Since various dimerisation pathways are conceivable for BCBs, we anticipated that subtle electronic differences in the aryl substituents, as well as the nature of the electron-withdrawing groups, would significantly influence the reaction outcome.^21^ Consequently, this study does not aim to establish a broadly applicable synthetic methodology, but rather focuses on elucidating the structures of the resulting dimers and investigating plausible dimerisation mechanisms.

## Results and discussion

During the course of these investigations, a previously unobserved 1,3-dipolar reaction pathway involving the aryl substituent of the BCB scaffold was discovered (Scheme 1(C)). Following the serendipitous detection of several dimeric compounds, we initiated a detailed study using BCB 1a as a model substrate. The initial step involved identifying and characterising the various dimer types formed. Subsequently, we examined the influence of different solvents and Lewis acids on the reaction outcome (Table 1). Solvent screening was limited to THF, toluene, CH_2_Cl_2_, and MeCN, in combination with Lewis acids of varying nature (see the SI for further details). Notably, and consistent with previous reports, cyclobutene (CB) 2a was detected as a side product in nearly all reactions, and the starting material 1a was occasionally observed as well. While THF and toluene did not lead to significant formation of dimeric products, MeCN selectively favoured the formation of BCB dimers of type 5. In contrast, reactions conducted in CH_2_Cl_2_ led to the formation of cyclobutene-derived adducts of types 3 and 4 as the major observed products.

**Table 1:** Optimisation studies of dimer formation in MeCN and CH_2_Cl_2_^a^

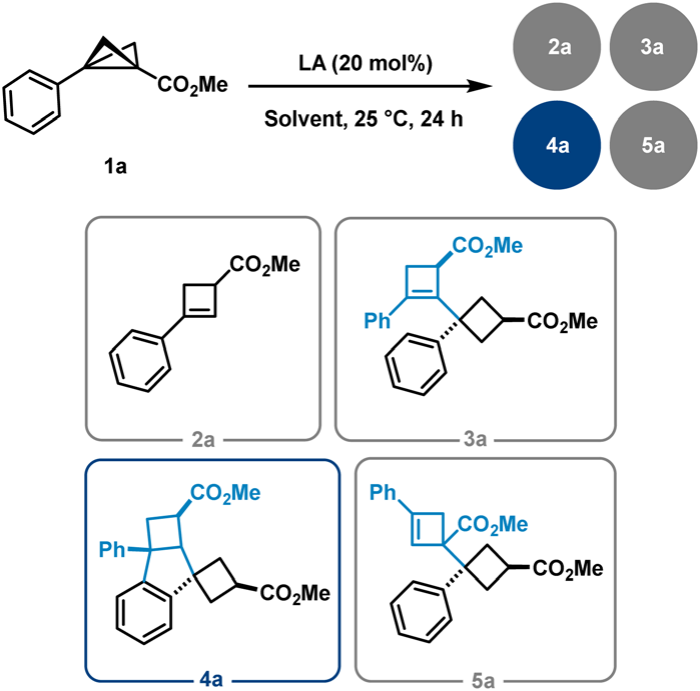								
	2a	3a	4a	5a	2a	3a	4a	5a
	MeCN				CH_2_Cl_2_			
AgOTf	6%	—	—	—	4%	8%	—	14%
Mg(OTf)_2_	6%	—	—	—	19%	—	—	—
Sc(OTf)_3_	6%	—	—	20%	5%	20%	15%	10%
FeCl_3_	4%	—	—	12%	—	4%	22%	36%
B(C_6_F_5_)_3_	32%	—	—	—	7%	—	—	—
Zn(OTf)_2_	12%	—	—	—	—	6%	—	8%
Bi(OTf)_3_	2%	—	—	12%	8%	48%	10%	12%
AlCl_3_	4%	—	—	—	1%	28%	18%	2%

a NMR yields are provided, determined with mesitylene (0.1 mmol) as internal standard.

Representative yields and product ratios for reactions conducted in MeCN and CH_2_Cl_2_ are summarised in Table 1. It is particularly noteworthy that dimerisation in MeCN was generally suppressed, except in the presence of three Lewis acids—Sc(OTf)_3_, FeCl_3_, and Bi(OTf)_3_—which selectively yielded dimer 5a. Although the distribution of dimeric products varied among different Lewis acids in CH_2_Cl_2_, Bi(OTf)_3_ exhibited remarkable selectivity toward the formation of product 3a. From these optimisation studies, it can be concluded that Bi(OTf)_3_, Sc(OTf)_3_, FeCl_3_, and AlCl_3_ are particularly effective catalysts for promoting BCB dimerisation. Additional concentration studies for Bi(OTf)_3_ showed that, above *ca.* 0.1 M, the yield of dimer 3a was only weakly affected by further increases in concentration, while the combined yield of the dimeric products increased, but the reaction became less selective for 3a. Upon dilution, a general decrease in dimer formation was observed.

**Scheme 2:**
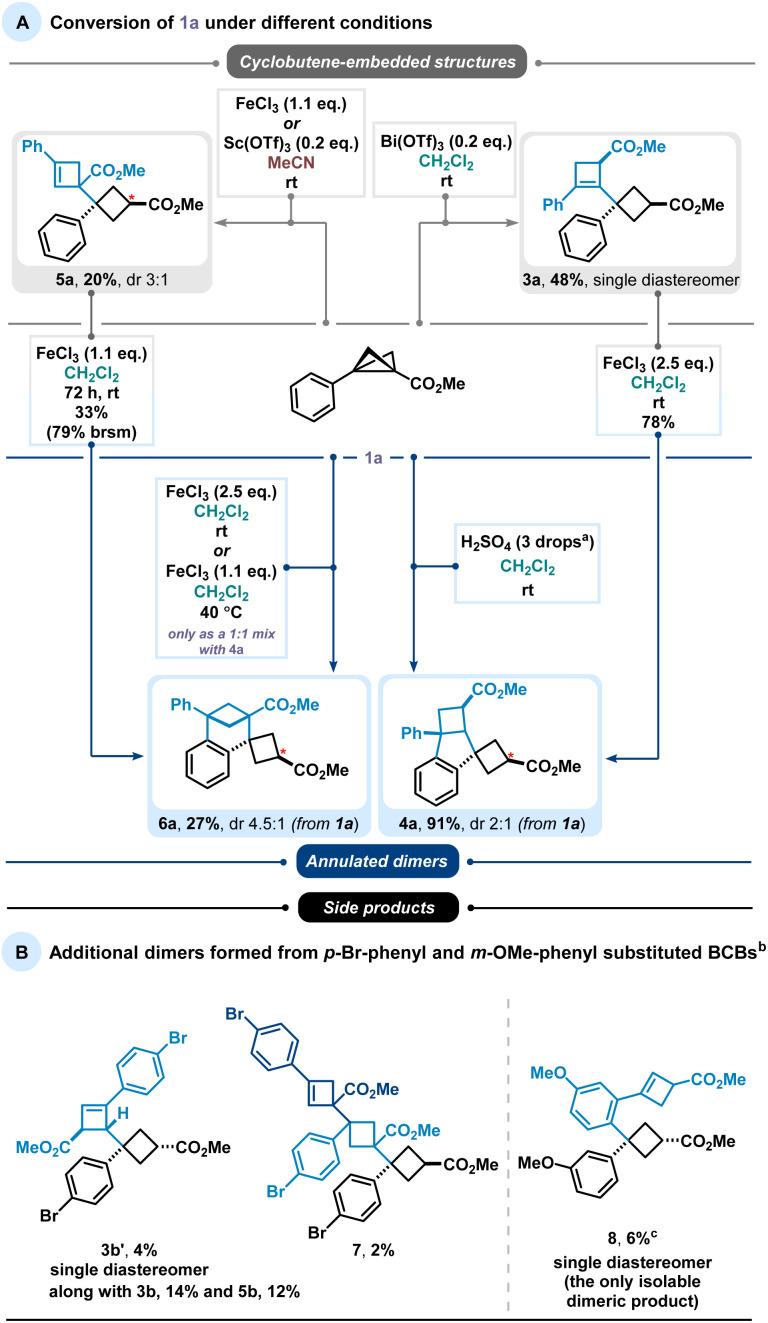
(A) Major dimerisation pathways found and (B) minor dimerisation pathways observed in the reactions of *p*-Br-phenyl- and *m*-OMe-phenyl-substituted BCBs (1b, 1c). ^*a*^One drop (∼50 µL) of H_2_SO_4_ per 1 mL of solvent is used. ^*b*^Reactions conditions: BCB (0.3 mmol), Sc(OTf)_3_ (20 mol%), CH_2_Cl_2_, 24 h, 25 °C. ^*c*^Together with 30% of corresponding cyclobutene 2c.

During our studies, we identified two types of dimer scaffolds: one containing embedded cyclobutene moieties (3a and 5a), and the other comprising oligocyclic structures with annulated aryl units (4a and 6a) (Scheme 2(A)). Product 3a was primarily formed in Bi(OTf)_3_-catalysed reactions in CH_2_Cl_2_, whereas 5a was obtained *via* Sc(OTf)_3_-catalysed or FeCl_3_-promoted transformations in MeCN. Additional experiments showed a slight improvement in the yield of the latter transformation when using an excess amount of FeCl_3_ instead of only 20 mol%. Notably, 3a was generated in a diastereoselective manner, while 5a consistently appeared as a mixture of diastereomers regardless of the reaction conditions. The cyclobutene-containing product 3a can be readily converted to the oligocyclic adduct 4a. Similarly, product 5a can be considered an intermediate en route to the benzene-fused bicyclo[3.1.1]heptane 6a. Furthermore, this bridged dimer 6a is also directly accessible from BCB 1a under harsher reaction conditions, although only as a 1 : 1 mixture with the oligocyclic dimer 4a. Product 4a, in turn, can be selectively obtained in excellent yields using Brønsted acid. As previously noted, these processes are highly sensitive to subtle electronic effects.

Other types of dimeric scaffolds were observed when electronically altered substituents were present on the BCBs (Scheme 2(B)). For example, with *p*-Br-phenyl-substituted BCB 1b, we discovered two additional dimers: 3b′, featuring a less substituted double bond, and a trimeric structure, 7. Another unique reactivity was observed with *m*-OMe-phenyl-substituted BCB 1c, which forms dimer 8, where cyclobutene and cyclobutane rings are connected through an aryl system.

To prove that *in situ* formed cyclobutene is an active intermediate we started with cyclobutene (CB) 2a as an alternative precursor (Table 2, see the SI for further details). Our experiments have shown that products 3a and 4a are accessible *via* both BCB and CB pathways. Thus, we conclude that the reaction proceeds *via* the same carbocationic intermediate. This result is in accordance with previously published acid activation of cyclobutanols.^22^ Our further investigations revealed that cyclobutene is less prone to react in MeCN, but at the same time is more reactive in CH_2_Cl_2_. For example, treating BCB in CH_2_Cl_2_ in the presence of Brønsted acid, H_2_SO_4_, gave oligocyclic adduct 4a in excellent yield (91%, entry 1), whereas CB under identical conditions showed significantly decreased selectivity (entry 2), leading to decomposition of the majority of the starting material, though formation of 4a was later achieved in the presence of FeCl_3_ in good yield (63%, entry 3). The same reaction, conducted at – 10 °C, gave a mixture of the starting CB and the products 3a and 4a (entry 4). The weaker Lewis acid Sc(OTf)_3_ stops the dimerisation at the level of addition–elimination products. Depending on the solvent used 3a and 5a are formed respectively in CH_2_Cl_2_ and MeCN (entries 5 and 6); noteworthy, in entry 6, BCB 1a was utilised, while CB 2a was found unreacted under similar conditions (entry 7). An attempt to use the BCB selectively as a nucleophilic partner and CB as a source for carbocationic intermediate was unsuccessful, further indicating that MeCN is not a suitable solvent for CB activation (entry 8).

**Table 2:** BCB *vs.* CB reactivity difference^a^

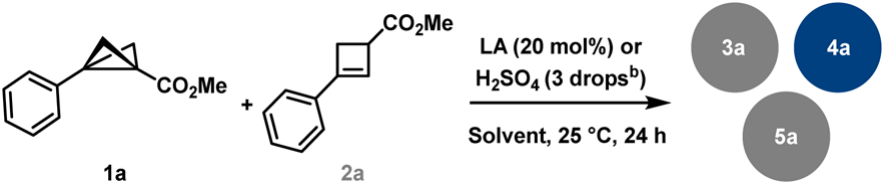					
Entry	1a (mmol)	2a (mmol)	Promoter	Solvent	Products (or 2a)
1	0.3	0	H_2_SO_4_	CH_2_Cl_2_	91% 4a
2	0	0.3	H_2_SO_4_	CH_2_Cl_2_	28% 4a
3	0	0.3	FeCl_3_	CH_2_Cl_2_	63% 4a
4	0	0.3	FeCl_3_^c^	CH_2_Cl_2_	30% 2a
					47% 3a
					18% 4a
5	0	0.3	Sc(OTf)_3_	CH_2_Cl_2_	40% 2a
					28% 3a
6	0.3	0	Sc(OTf)_3_	MeCN	30% 2a
					20% 5a
7	0	0.3	Sc(OTf)_3_	MeCN	99% 2a
8	0.15	0.15	Sc(OTf)_3_	MeCN	64% 2a
					5% 5a

a NMR yields are provided, determined with mesitylene (0.15 mmol) as internal standard.

b One drop (∼50 µL) of H_2_SO_4_ per 1 mL of solvent is used.

c The reaction was conducted at −10 °C.

**Scheme 3:**
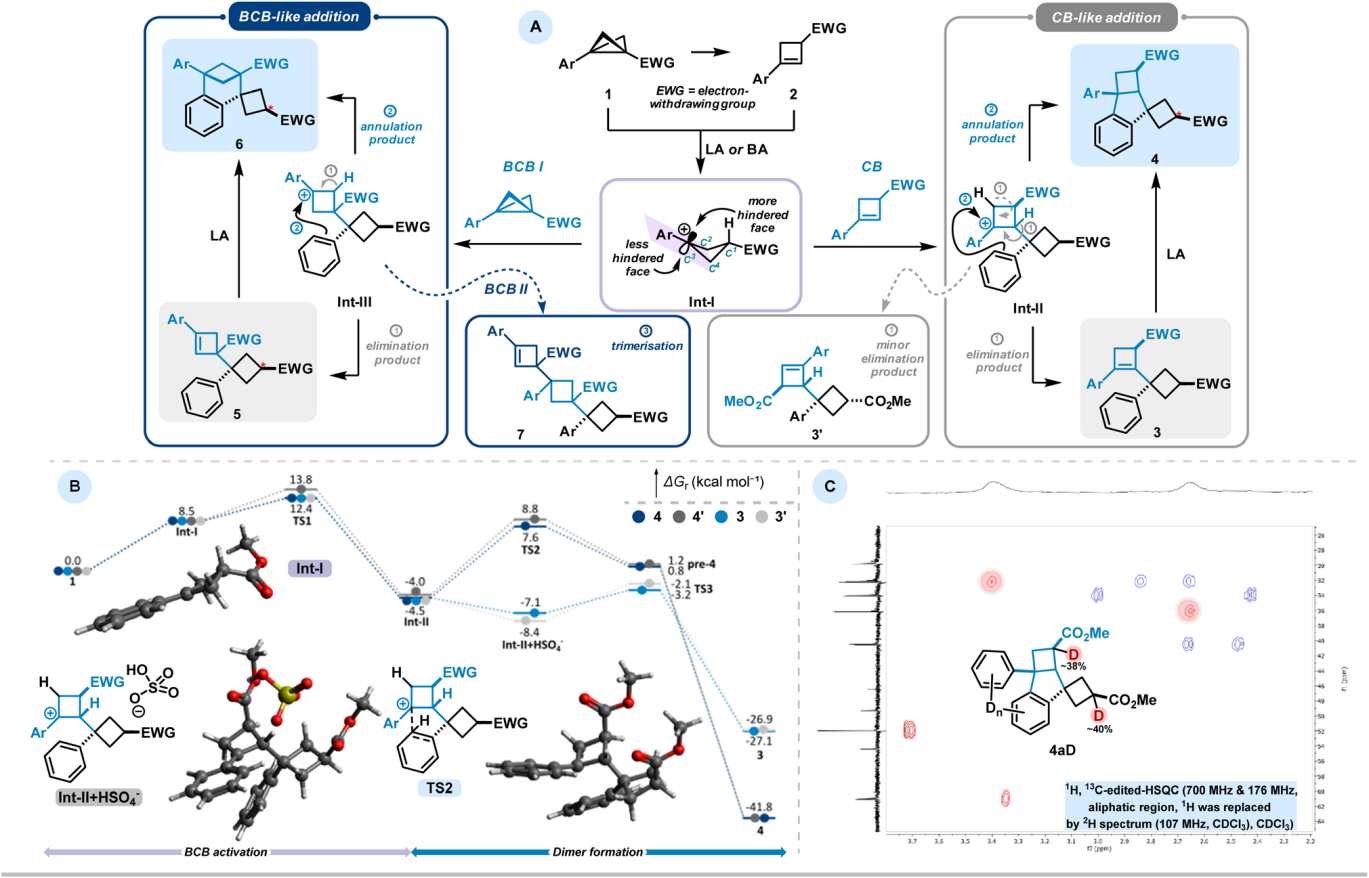
(A) Proposed mechanism of observed dimerisation pathways. (B) Energy diagram of the DFT studies (M06-2X/def2-TZVP-D3//r^2^scan-3c, CPCM (CH_2_Cl_2_)). (C) ^1^H, ^13^C-edited-HSQC (700 MHz & 176 MHz, zoomed aliphatic region, ^1^H was replaced by ^2^H spectrum (107 MHz, CDCl_3_), CDCl_3_). Scaffold 4 synthesised in D_2_SO_4_. BCB 1a (0.3 mmol), D_2_SO_4_ 99,5 atom% D, CH_2_Cl_2_, 25 °C, 24 h.

Based on the aforementioned experiments, we propose the following mechanistic scenario (Scheme 3). In the presence of LA or BA, BCB forms a carbocationic intermediate (Int-I), either directly or *via* a cyclobutene intermediate. This intermediate can then be intercepted by another molecule of CB or BCB, generating aryl-stabilised carbocationic species Int-II or Int-III, respectively. Intermediate Int-II can undergo further transformation *via* two competing pathways: (1) proton elimination to introduce a double bond, yielding product 3, or (2) Friedel–Crafts-type annulation, leading to the formation of indane-type product 4. The formation of 3 may be accompanied by the generation of less-substituted alkene isomer 3′, as observed in compound 3b′. The occurrence of 3′ can be attributed to reduced stabilisation of the carbocation prior to elimination, indicating a stronger influence of kinetic control in BCB 1b. Similar reactivity is observed for intermediate Int-III: annulation results in the formation of aryl-fused bicycloheptane skeleton 6, while proton elimination affords the BCB addition product 5. As noted above, under harsher conditions, both 3 and 5 can undergo further transformation to form their respective annulated isomers. This suggests that the proton elimination process in both cases is reversible. Furthermore, we propose that the trimeric structure 7, observed in the reaction of the *p*-bromophenyl-substituted BCB 1b, arises from Int-III*via* nucleophilic attack by a third BCB molecule, followed by proton elimination. A minor reaction pathway from Int-I, observed in the case of the electron-donating m methoxy-substituted BCB, reveals that activated aromatic systems can act as nucleophiles toward BCBs. This leads to the formation of Friedel–Crafts-type addition products 8, rather than reaction at the emerging double bond.

Separately, we would like to address the stereochemistry of the observed processes. In general, the major diastereomer in all cases is the one in which the initial substituents on the BCB ring are positioned *trans* to each other. We attribute this to side-selectivity in intermediate Int-I, which arises from its non-planar structure. Specifically, C^2^, C^3^ (the carbocationic center), C^4^, and the aryl group lie in the same plane, while C^1^ is displaced from this plane, with the electron-withdrawing group (EWG) occupying an equatorial position. This creates sterically less and more hindered reactive sides. As a result, CB or BCB preferentially attack the carbocationic center in Int-I from the less hindered face, leading to products 3 and 5, respectively—i.e., the aryl substituent and the EWG are positioned *trans* to each other. NMR analysis of derivative 3 (resulting from CB addition followed by elimination) indicates the exclusive formation of a single diastereomer. In contrast, compounds 4 (CB addition followed by annulation), 5 (BCB addition followed by elimination), and 6 (BCB addition followed by annulation) are formed predominantly as mixtures of diastereomers, with the *trans*-isomer being the major one in each case. Notably, the conversion of 3 to 4 proceeds without any detectable loss of diastereoselectivity.

To elucidate the reaction mechanism, density functional theory (M06-2X/def2-TZVP-D3//r^2^scan-3c, CPCM (CH_2_Cl_2_)) calculations were performed for the sulfuric acid promoted reaction pathway (Scheme 3B, for further details see SI).^23^ Protonation of BCB 1 generates carbocationic species Int-I, which undergoes nucleophilic attack by CB 2 to form intermediate Int-II. The envelope conformation of Int-I induces facial selectivity: attack from the convex face proceeds with a barrier of 3.9 kcal mol^−1^, while the concave approach requires 5.3 kcal mol^−1^, rendering Int-II thermodynamically favoured over Int-II′ by 0.5 kcal mol^−1^. Intramolecular Friedel–Crafts alkylation by the pendant phenyl ring occurs with barriers of 12.1 kcal mol^−1^ (TS2) and 12.9 kcal mol^−1^ (TS2′), respectively, affording pre-4 and pre-4′, which upon deprotonation yield products 4 and 4′.

Competing deprotonation from Int-II was investigated, as this side reaction was observed for electron-deficient substrates. A hydrogen-bonded Int-II + HSO_4_^−^ adduct enables elimination *via*TS3 (3.9 kcal mol^−1^), 8.2 kcal mol^−1^ lower than cyclization. Nevertheless, elimination is disfavoured: bisulfate, formed upon protonation, is consumed during rearomatisation and remains only in catalytic amounts, thus disfavouring the bimolecular elimination pathway. Moreover, the annulation product is thermodynamically preferred by 13.9 kcal mol^−1^ (Δ*G*_r_ = −41.0 *vs.* −27.1 kcal mol^−1^), providing an additional driving force for cyclization.

As a final step in our mechanistic investigation, we synthesized 4a in deuterated sulfuric acid to obtain the isotopically labeled analogue 4aD. Deuterium incorporation within the aliphatic framework occurs predominantly at *α*-positions to electron-withdrawing groups, consistent with the expected protonation pattern (Scheme 3C). No deuterium incorporation was detected at the bridgehead CH position or at any of the CH_2_ groups. This finding excludes reversible protonation of CB under the reaction conditions and indicates that this intermediate is either rapidly consumed or engaged in competing side processes prior to isotopic exchange. In contrast, the aromatic ring system displays extensive deuterium incorporation, reflecting efficient H/D exchange under the strongly acidic conditions employed.

**Scheme 4:**
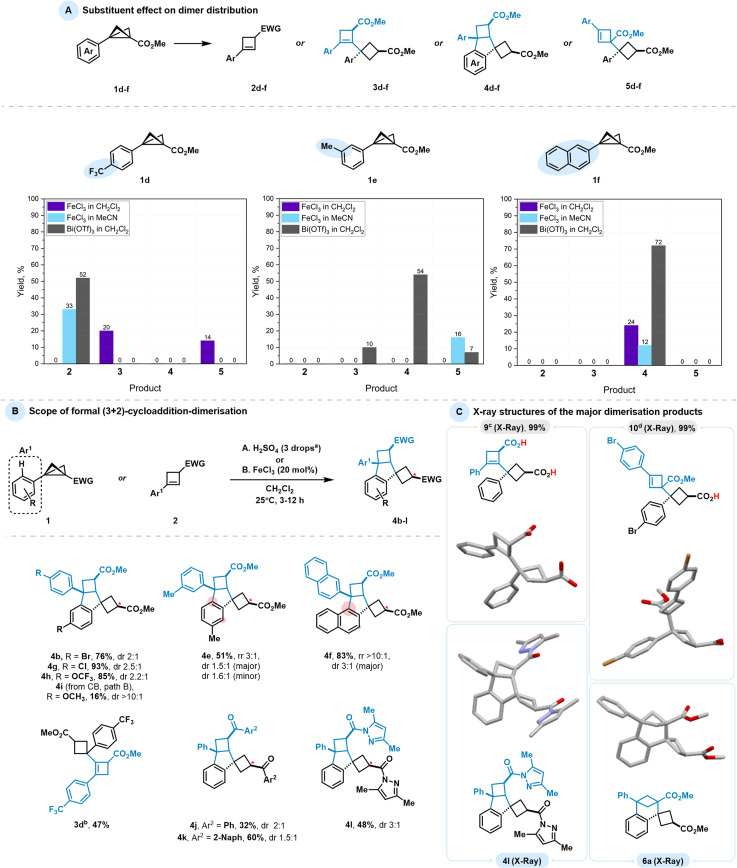
(A) Product distribution in case of dimer formation of BCB of different nature (1d, 1e, 1f). The reactions were conducted in LA (20%), solvent (1 M), 25 °C, 24 h. Isolated yields are provided. (B) Scope of formal (3 + 2)-cycloaddition-dimerisation (in all the cases except 4i, conditions A were used). (C) Molecular structures (scXRD) of the major dimerisation products (9, 10, 4l, 6a). ^*a*^One drop (∼50 µL) of H_2_SO_4_ per 1 mL of solvent is used. ^*b*^Product 4d was not detected, 3d is the sole product. ^*c*,*d*^LiOH (18 equiv.), THF, 24 h, 0 to 25 °C.

Using the optimised conditions for Bi(iii)- and Fe(iii)-catalysed reactions, we examined the effect of substituents on the dimerisation outcome. We selected a range of aryl-substituted BCBs bearing a strong electron-withdrawing group CF_3_ (1d), a weakly electron-donating methyl group (1e), and an alternative aromatic system represented by a naphthyl group (1f) (Scheme 4(A)). Each compound was subjected to a series of dimerisation experiments under the selected conditions. As controlling conditions, we chose the strong Lewis acid FeCl_3_ in both CH_2_Cl_2_ (Conditions I) and MeCN (Conditions II), and the relatively weaker Lewis acid Bi(OTf)_3_ in CH_2_Cl_2_ (Conditions III), reflecting a gradient of activation modes based on our optimisation, from the most harsh (Conditions I) to the mildest (Conditions III). Electron-poor CF_3_-substituted BCB 1d showed dimer formation only under harsh conditions (FeCl_3_ in CH_2_Cl_2_) as a mixture of BCB- and CB-addition elimination products. Milder conditions were insufficient to initiate the dimerisation process and instead led to termination with CB formation. In contrast, the *m*-methyl substituted BCB 1e did not produce noticeable amounts of dimer or CB under harsh conditions because of its high reactivity, but higher oligomers and polymers were observed. Under the less activating Conditions II, the transformation yielded, to a major extent, the BCB-addition elimination product, similar to the parent substrate. Notably, trace amounts of BCB-annulated product were also detected. Under the least reactive Conditions III, a mixture of dimers was observed, with the predominant formation of skeleton 4, as the presence of the methyl group in this position promotes annulation processes. Replacing the phenyl group with naphthyl in 1f behaved similarly, with derivative 4 being the sole product observed in every scenario. We also extended these studies to BCBs bearing alternative electron-withdrawing groups, including ketones, amides, and Weinreb amides. Under the examined conditions, however, no dimeric products were detected in any of these cases (see the SI).

As a concluding step of our investigation, we explored Brønsted acid-promoted indane skeleton formation and tested the applicability of the novel 1,3-dipolar reactivity across a series of BCBs and CBs (Scheme 4(B)). BCB esters with variously substituted aryl moieties led to the desired product formation. For example, halogens such as Br (4b) and Cl (4g) facilitated the transformation in good to excellent yields (76% and 93%, respectively). A substrate with a trifluoromethoxy group (4h), which has a similar electronic effect to halogens, gave an 85% yield. As previously mentioned, the weakly electron-donating aryl group in BCB 1e yielded a mixture of regioisomers corresponding to *p*- and *o*-annulation relative to the methyl group. The naphthyl-substituted product 4f was obtained in a higher yield (83%) compared to the Bi-catalysed process.

For the electron-donating *p*-OMe group, the product was accessed using CB in an FeCl_3_-catalysed process but in a moderately low yield, as the corresponding BCB is less stable than its less electron-rich analogs. Fe(iii)-catalysed conditions were chosen because the H_2_SO_4_-promoted process showed decreased selectivity, consistent with mechanistic studies (see Table 2). No indane product was observed for BCB 1d bearing the CF_3_-substituted aryl group; in this case, dimerisation halted at CB 3d due to the strong deactivating effect of the CF_3_ group. The transformation was also feasible for other electron-withdrawing substituents on the BCB, such as amide 4l and ketones 4j and 4k, with moderate to good yields. Heterodimerisation attempts using BCB pairs 1a/1d and 1a/1j were unsuccessful, showing no selectivity for heterodimer formation. While the 1a/1d combination mainly afforded homodimer 4a and cyclobutene 2d, the 1a/1j pair gave a complex mixture of dimers of different composition. However, the Ph-substituted Weinreb amide and the unsubstituted BCB bearing a ketone (1m and 1n, see the SI for further details) did not furnish dimers. In these cases, the only detectable product arose from water addition to the BCB in a 1,2-manner, similar to transformations reported for mono-substituted BCBs.^20^

The structures of the four main observed pathways for BCB dimerisation described in this study were confirmed by single-crystal X-ray diffraction (scXRD) (Scheme 4(C)). To obtain compounds 9 and 10, we performed hydrolyses of 3a and 5b, respectively. Dicarboxylic acid 9 was obtained *via* LiOH-mediated complete hydrolytic cleavage of the ester groups of 3a, whereas 5b yielded the mono-carboxylic acid 10 under the same conditions, consistent with the more sterically hindered ester remaining unaffected. Single crystals of the corresponding derivatised products 9, 10 and annulated products 4l and 6a were obtained by vapor diffusion of *n*-heptane/*n*-hexane into a solution of the compounds in CHCl_3_/CH_2_Cl_2_.

## Conclusions

In conclusion, dimerisation products of aryl-substituted bicyclo[1.1.0]butanes (BCBs) were investigated. To initiate these processes, a range of different Lewis and Brønsted acids was examined. Although the outcome strongly depends on the type of BCB and the catalyst or mediator used, four representative scaffolds of such dimerisation products were obtained, whose structures were unequivocally confirmed by X-ray crystallography. Two of these consist of linked cyclobutene/cyclobutane adducts, whereas the other two reveal an oligocyclic framework. The latter can be regarded as formal (3 + 2)- and (3 + 3)-cycloadducts formed *via* a previously unknown 1,3-dipolar reactivity of such species involving the aryl residue. Structurally, either a spiro-annulated indane scaffold or a spirotetrahydronaphthalene with a bridging methylene unit is formed. Mechanistic studies, including DFT computations, explain the origin of the observed reactivity patterns. We believe that these results are of fundamental interest to the growing number of researchers working on BCB chemistry, as such dimerisation can occur as a background reaction in all Lewis or Brønsted acid-catalysed transformations of BCBs, potentially reducing yields significantly. It goes without saying that other dimerisation products can be expected under photochemical conditions.

## Author contributions

M. G. and D. A. K. conceptualised the project, carried out the reaction optimisation, conducted mechanistic studies, investigated the scope, and synthesised the BCBs; K. S. conducted mechanistic studies. H. v. K. performed computations. D. B. W. conceptualised the project, provided supervision and acquired funding. The manuscript was written by D. A. K. with contributions from M. G., H. v. K. and D. B.W. All authors agreed with the content of the paper.

## Conflicts of interest

There are no conflicts to declare.

## Supplementary Material

SC-OLF-D6SC01258B-s001

SC-OLF-D6SC01258B-s002

## Data Availability

CCDC 2454075 2465273 2473002 2476077 and 2476959 contain the supplementary crystallographic data for this paper.^24*a*–*e*^ The data supporting this article have been included as part of the supplementary information (SI). Further data that support the findings of this study are available from the corresponding author upon request. Supplementary information: experimental procedures, mechanistic experiments and characterisation data of new compounds. See DOI: https://doi.org/10.1039/d6sc01258b.
