# Supplementary material for: Dimerisation of aryl-substituted bicyclobutanes (BCBs): revealing a new mode of 1,3-dipolar background reactivity
Source: Chem Sci. 2026 Mar 31;17(20):10159–67. doi: 10.1039/d6sc01258b (PMC13071511; doi:10.1039/d6sc01258b)
Supplement: SC-017-D6SC01258B-s001 [file SC-017-D6SC01258B-s001.pdf]

Supplementary Information (SI) for Chemical Science.
This journal is © The Royal Society of Chemistry 2026

Dimerisation of aryl-substituted bicyclobutanes (BCBs): Revealing a new mode of 1,3-dipolar background reactivity

Malini George,† Daniil A. Knyazev,† Kamil Swiatek, Heinrich F. von Köller and Daniel B. Werz*

Supplementary information

Albert-Ludwigs-Universität Freiburg,

Institute of Organic Chemistry,

Albertstr. 21, 79104 Freiburg, Germany

(*corresponding author: daniel.werz@chemie.uni-freiburg.de)

Table of contents

1. General experimental S3
2. General procedures S4
3. Synthesis of starting materials S5
4. Optimisation of the reaction conditions S6
5. Synthesis of bicyclobutane bimers S9
6. Mechanistic experiments and additional investigations S34
7. Follow up S39
8. DFT Calculations S42
9. NMR spectra S57
10. Crystal structure determinations S143
11. References S209

1. General experimental

All reactions were carried out under an inert atmosphere of argon unless stated otherwise. Solvents for column chromatography were distilled prior to use. Dry solvents were either taken from a solvent purification system and stored over molecular sieves under argon (THF, CH_2Cl_2) or purchased dry over molecular sieves (Thermo Scientific; DCE). Commercially available reagents were purchased from ABCR, Thermo Scientific, Sigma-Aldrich or BLDPharm and used without further purification. Column chromatography was carried out using Silica 60 (0.04 – 0.063 mm, Macherey-Nagel). Thin-layer chromatography was carried out on SIL G-25 UV254 glass plates from Macherery-Nagel. For the detection of spots, UV light (254 nm) was used. Automated column chromatography was carried out on a CombiFlash® Rf 75 system by Teledyne Isco using pre-packed Büchi FlashPure™ Ecoflex cartridges (spherical silica, 25 µm). or Interchim puriFlash® SI-HP cartridges (spherical silica, 30 µm). Solvent systems, gradients and cartridge sizes are stated individually. Further purification of scope molecules by preparative HPLC was carried out on preparative normal phase HPLC was carried out on a Shimadzu Nexera LC Prep System equipped with a LC-20AP pump and a Macherey-Nagel VP 250/21 NUCLEODUR 100-5 silica column. NMR-spectroscopic analyses (^{1}H, ^{13}C, ^{19}F) were carried out on a 700 MHz Bruker Avance III Neo 700, 500 MHz Bruker Avance DRX 500 or 400 MHz Bruker Avance II 400 Instrument. ^{1}H-shifts are given relative to the solvent peak (δ_{CDCl3} = 7.26 ppm, δ_{CD3CN} = 1.94 ppm, $\delta_{DMSO\text{-}d6}$ = 2.50 ppm) and $^{13}C\{^{1}H\}$-shifts to the solvent peak (δ_{CDCl3} = 77.16 ppm, δ_{CD3CN} = 1.32 ppm, $\delta_{DMSO\text{-}d6}$ = 39.52 ppm). Signals are described as following: "s" = singlet, "d" = doublet, "t" = triplet, "q" = quartet and combinations of the latter. Multiplets are labelled as "m". High-resolution mass spectrometry was carried out either on a Thermo Exactive mass spectrometer (ESI and APCI measurements with Orbitrap analyser). Low-resolution GC/MS with CI-ionisation was measured on a Thermo Scientific Trace 1300 gas chromatograph with an ISQ LT MSD using ammonia as reactant gas. ATR-FTIR spectroscopy was carried out on a Spectrum Two Spectrometer from Perkin Elmer. Samples, if not stated otherwise, were measured neat on a diamond ATR crystal. Melting points were measured on a Schorpp MPM-HV2 apparatus using the open capillary method.

2. General procedures

2.1 General procedure (GP1) for the synthesis of bicyclobutane dimers

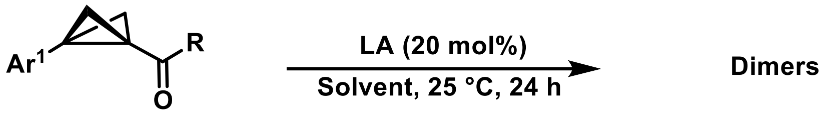

To an oven-dried microwave vial equipped with a magnetic stir bar and a rubber septum (vacuum and argon cycled) was added bicyclobutanes (300 µmol, 1.00 equiv.) and vacuum and argon cycled again. The mixture was then dissolved in 3 mL of solvent at 25 °C under argon and stirred for 5 minutes. LA (60 µmol, 20 mol%) is then added to the reaction mixture. The vial is then capped and stirred for 24 hours at the same temperature. The solvent was evaporated and the crude residue was pre-adsorbed on Celite and subjected to automated column chromatography.

2.2 General procedure (GP2) for the synthesis of bicyclobutane dimers

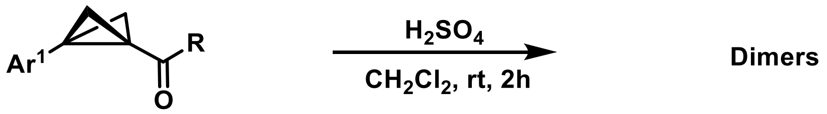

To an oven-dried microwave vial equipped with a magnetic stir bar added bicyclobutanes (300 µmol, 1.00 equiv.). The mixture was then dissolved in 3 mL of CH_2Cl_2 at 25 °C. 3 drops of conc. H_2SO_4 is then added to the reaction mixture(One drop (~50 µL) of H_2SO_4 per 1 mL of solvent is used.).The reaction is stirred for 2 hours at the same temperature. The solvent was evaporated and the crude residue was pre-adsorbed on Celite and subjected to automated column chromatography.

2.3 General procedure (GP3) for the synthesis of bicyclobutane dimers

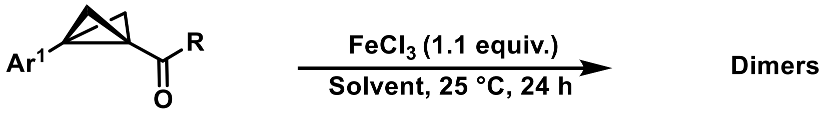

To an oven-dried microwave vial equipped with a magnetic stir bar and a rubber septum (vacuum and argon cycled) was added bicyclobutanes (300 µmol, 1.00 equiv.) and vacuum and argon cycled again. The mixture was then dissolved in 3 mL of solvent at room temperature under argon and stirred for 5 minutes. $FeCl_3$ (53.4 mg, 330 µmol, 1.10 equiv.) is then added to the reaction mixture. The vial is then capped and stirred

for 24 hours at the same temperature. The solvent was evaporated and the crude residue was pre-adsorbed on Celite and subjected to automated column chromatography.

3. Synthesis of starting materials

3.1 Synthesis of bicyclobutanes and cyclobutenes

All BCBs **1a,**[1] **1b,**[2] **1c,**[3] **1d,**[2] **1e,**[4] **1f,**[5] **1g,**[2] **1h,**[2] **1j,**[6] **1k,**[6] **1l,**[7] **1m,**[8] **1n**[9] and CB **2i**[5] were prepared following the literature procedures. The analytical data were in accordance with those reported in the literature.

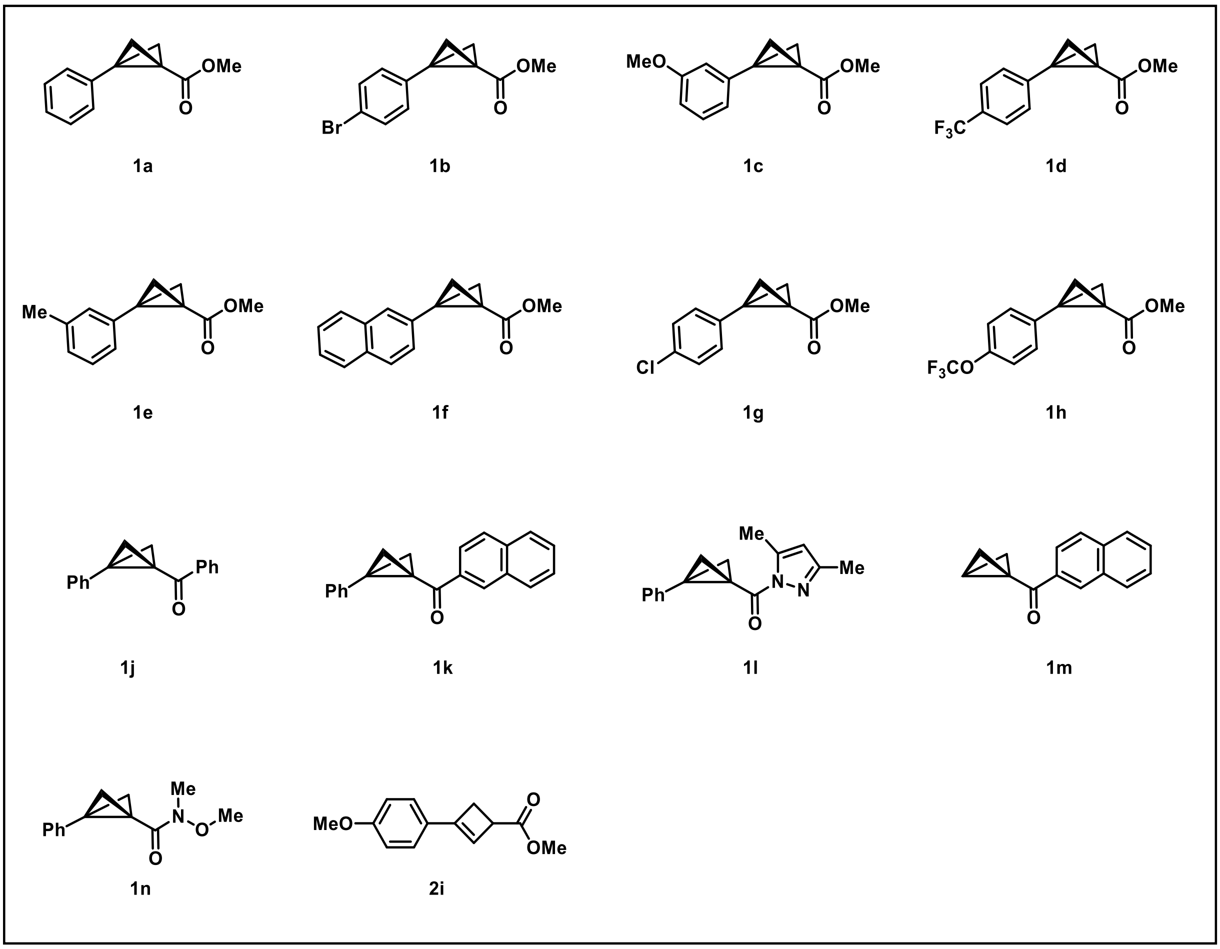

4. Optimisation of the reaction conditions

4.1 Optimisation - Lewis acids and solvents

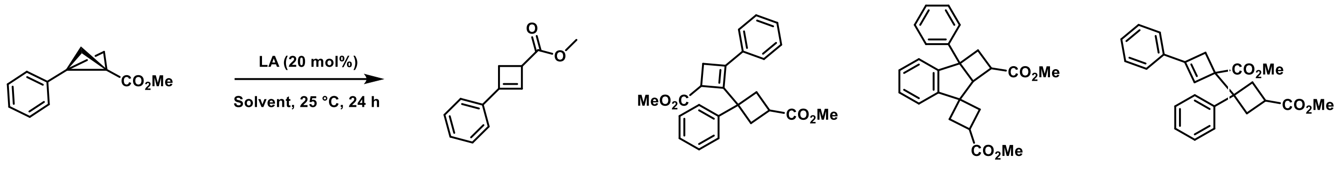

1a 2a 3a 4a 5a

**Lewis Acid**	**THF** (NMR yield in %)					**CH_2Cl_2** (NMR yield in %)				
	**1a**	**2a**	**3a**	**4a**	**5a**	**1a**	**2a**	**3a**	**4a**	**5a**
AgOTf	0	10	0	0	0	0	4	8	0	14
$Mg(OTf)_2$	70	3	0	0	0	80	19	0	0	0
$Sc(OTf)_3$	0	10	0	0	0	0	5	20	15	9
$FeCl_3$	0	14	0	0	0	0	0	4	22	36
$B(C_6F_5)_3$	0	29	0	0	0	0	7	0	0	0
$Zn(OTf)_2$	0	34	10	0	10	0	0	6	0	8
$Bi(OTf)_3$	0	60	0	0	0	0	8	48	10	12
$AlCl_3$	0	75	0	0	0	0	1	28	18	2
**Lewis Acid**	**MeCN** (NMR yield in %)					**Toluene** (NMR yield in %)				
	**1a**	**2a**	**3a**	**4a**	**5a**	**1a**	**2a**	**3a**	**4a**	**5a**
AgOTf	83	6	0	0	0	0	0	0	0	0
$Mg(OTf)_2$	88	6	0	0	0	0	0	0	0	0
$Sc(OTf)_3$	0	6	0	0	20	0	22	0	0	0
$FeCl_3$	0	4	0	0	12	0	0	0	0	0
$B(C_6F_5)_3$	0	32	0	0	0	0	0	0	0	0
$Zn(OTf)_2$	72	12	0	0	0	0	54	0	0	0
$Bi(OTf)_3$	0	2	0	0	12	0	0	0	0	0
$AlCl_3$	0	4	0	00	0	0	40	0	0	0

Reaction conditions: **1a** (100 μmol), Lewis acid (20 μmol), Solvent (1 mL) at 25 °C, 24 h. NMR yields were determined by using mesitylene (100 μmol) as an internal standard.

4.2 Additional experiments with cyclobutene and bicyclobutane

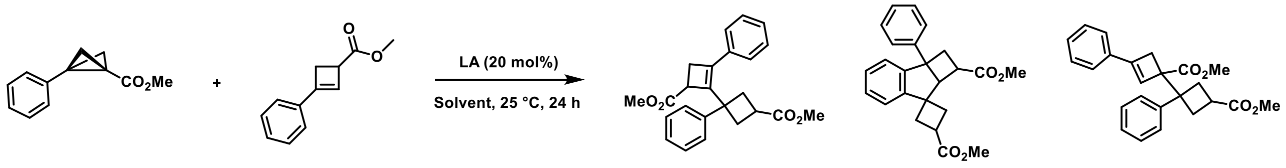

1a 2a 3a 4a 5a

Entry	1a (μmol)	2a (μmol)	catalyst	μmol	Solvent	T [°C]	NMR Yield [%][]
1	150	150	$Sc(OTf)_3$	60	CH_2Cl_2	25	2a
2	150	150	$Sc(OTf)_3$	60	Toluene	25	2a
3	150	150	$Sc(OTf)_3$	60	THF	25	2a
4	150	150	$Sc(OTf)_3$	60	DMF	25	n.r.
5	150	150	$FeCl_3$	60	MeCN	25	6% 2a<br>53% 4a
6	150	150	$Sc(OTf)_3$	60	MeCN	40	16% 2a<br>10% 5a
7	150	150	$Sc(OTf)_3$	60	MeCN: CH_2Cl_2	25	53% 2a<br>9% 5a
8	150	150	$FeCl_3$	60	MeCN	25	3% 2a<br>4% 5a
9	150	150	$Sc(OTf)_3$	150	MeCN	25	35% 2a<br>4% 5a
10	300	0	$FeCl_3$	150	MeCN	25	8% 2a<br>8% 5a
11	0	300	$FeCl_3$	60	CH_2Cl_2	0- 25	53% 4a
12	0	300	$Sc(OTf)_3$	60	CH_2Cl_2	-10	60% 2a<br>20% 3a
13	0	300	$FeCl_3$	30	CH_2Cl_2	25.	77% 4a
14	0	300	H_2SO_4	3 drops	CH_2Cl_2	-10	55% 4a
15	0	300	$FeCl_3$	60	CH_2Cl_2	25.	28% 3a<br>44% 4a

Reaction conditions: **1a**, **2a**, Solvent (3 mL), 24 h. NMR yields were determined by using mesitylene(150 μmol) as an internal standard. One drop (~50 μL) of H_2SO_4 per 1 mL of solvent is used.

4.3 Concentration study

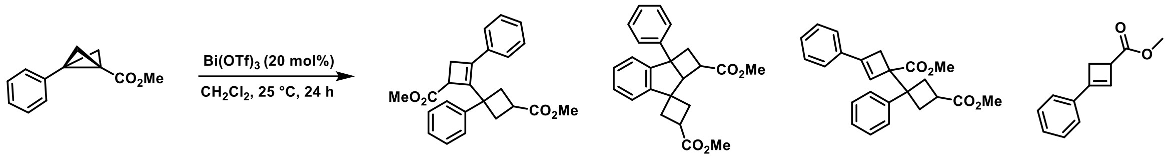

1a 3a 4a 5a 2a

1a (μmol)	Solvent (mL)	Concentration (mmolar)	3a	4a	5a	2a
100	2	0.05	30	12	10	3
100	1	0.1	48	10	12	8
200	1	0.2	46	24	14	1

Reaction conditions: $Bi(OTf)_3$ (20 mol% of **1a)**, at 25 °C, 24 h. NMR yields were determined by using CH_2Br_2 (100 μmol) as an internal standard.

4.4 Scope study

	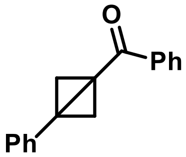	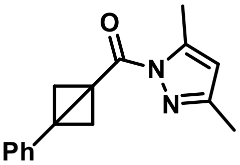	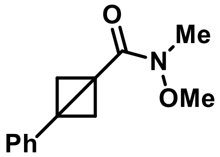	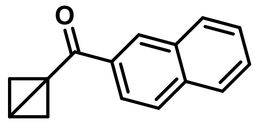
**$Sc(OTf)_3$ (0.2 eq.) DCM rt**	No reaction	No reaction	No detectable dimers	No detectable dimers
**H_2SO_4 (3 drops) DCM rt**	32%	48%	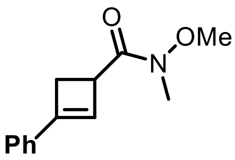	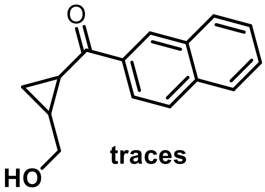
**$FeCl_3$ (1.1 eq.) MeCN rt**	No detectable dimers	No detectable dimers	No detectable dimers	No detectable dimers

5. Synthesis of bicyclobutane bimers

Methyl 3-(3-methoxyphenyl)cyclobut-2-ene-1-carboxylate (2c).

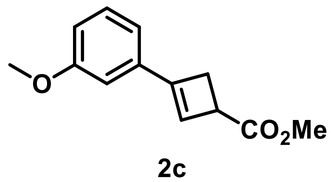

2c

Following the **GP1** taking $Sc(OTf)_3$ as LA, in CH_2Cl_2 **2c** was obtained as a colorless oil (10 mg, 45.8 µmol, 15%) along with **8**. Purified by automated column chromatography (25 g 30SI-HP cartridge, 15 mL/min) using pentane/EtOAc = 100:1 – 10:1 (Rf = 0.8 in 10:1 pentane/EtOAc).

^{1}H-NMR (700 MHz, $CDCl_3$) δ = 7.26 (t, *J* = 7.9 Hz, 1H), 6.96 (dt, *J* = 7.6, 1.3 Hz, 1H), 6.88 (dd, *J* = 2.6, 1.5 Hz, 1H), 6.84 (ddd, *J* = 8.2, 2.6, 1.0 Hz, 1H), 6.28 (d, *J* = 1.3 Hz, 1H), 3.82 (s, 3H), 3.72 (s, 3H), 3.69 – 3.63 (m, 1H), 3.06 (dd, *J* = 12.9, 4.8 Hz, 1H), 3.00 (dd, *J* = 12.9, 2.0 Hz, 1H).

^{13}C{^{1}H}-NMR (176 MHz, $CDCl_3$) δ = 174.0, 159.8, 148.3, 135.3, 129.6, 125.0, 117.5, 114.3, 110.2, 55.4, 52.0, 41.4, 32.8.

IR (Diamond-ATR): $\tilde{\nu}$ (cm^{-1}) = 2927, 1731, 1685, 1598, 1583, 1488, 1437, 1210, 1172, 1038.

HRMS (APCI,Q-TOF): $[M+H]^+$ calcd for $C_{13}H_{15}O_3^+$ =219.1016; found: 219.1015.

Dimethyl 1',3-diphenyl-[1,1'-bi(cyclobutan)]-2-ene-1,3'-dicarboxylate (3a).

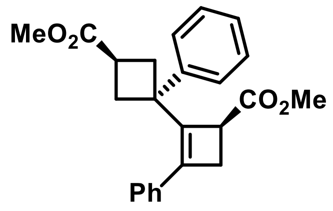

3a

Following the **GP1** taking $Sc(OTf)_3$ as LA, in CH_2Cl_2 **3a** was obtained as a colorless oil (11.1 mg, 29.5 µmol, 20%) along with **4a** and **5a**. Purified by automated column

chromatography (25 g 25SI-HP cartridge, 15 mL/min) using Pentane/EtOAc = 100:1 – 10:1 (Rf = 0.52 in 10:1 pentane/EtOAc). It was further purified with normal phase HPLC (Gradient, 95:5 to 90:10 hexane/EtOAc).

^{1}H-NMR (700 MHz, $CDCl_3$) δ = 7.53 – 7.49 (m, 2H), 7.38 – 7.33 (m, 2H), 7.31 – 7.26 (m, 2H), 7.25 – 7.19 (m, 4H), 3.64 (s, 3H), 3.65 – 3.62 (m, 1H), 3.47 (s, 3H), 3.15 (p, *J* = 9.1 Hz, 1H), 2.91 – 2.84 (m, 2H), 2.82 – 2.76 (m, 3H), 2.73 (ddd, J = 11.5, 8.8, 4.3 Hz, 1H).

^{13}C{^{1}H}-NMR (176 MHz, $CDCl_3$) δ = 175.2, 173.9, 144.2, 143.9, 139.5, 134.3, 128.8 (2C), 128.3 (2C), 127.9, 126.9 (2C), 126.6, 126.4 (2C), 51.9, 51.6, 44.8, 41.8, 37.4, 35.9, 32.6, 29.5.

IR (Diamond-ATR): $\tilde{\nu}$ (cm^{-1}) = 1728, 1444, 1434, 1337, 1255, 1218, 1195, 1162, 1093, 1030.

HRMS (APCI,Q-TOF): $[M+H]^+$ calcd for $C_{24}H_{25}O_4^+$ = 377.1747; found: 377.1749.

Dimethyl 1',4-bis(4-bromophenyl)-[1,1'-bi(cyclobutan)]-4-ene-2,3'-dicarboxylate (3b).

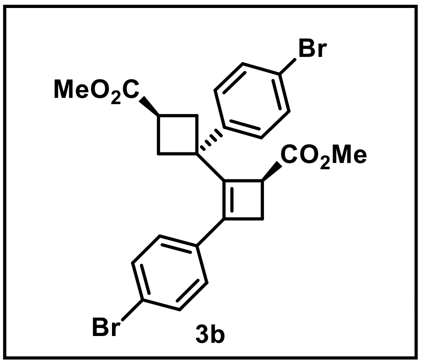

Following the **GP1** taking $Sc(OTf)_3$ as LA, in CH_2Cl_2 **3b** was obtained as a colorless oil (11.4 mg, 21.3 µmol, 14%) along with **5b**. Purified by automated column chromatography (25 g 25SI-HP cartridge, 15 mL/min) using pentane/EtOAc = 100:1 – 10:1 (Rf = 0.63 in 10:1 pentane/EtOAc).

^{1}H-NMR (700 MHz, $CDCl_3$) δ = 7.49 – 7.44 (m, 2H), 7.42 – 7.37 (m, 2H), 7.39 – 7.32 (m, 2H), 7.02 – 6.96 (m, 2H), 3.67 – 3.64 (m, 1H), 3.65 (s, 3H), 3.52 (s, 3H), 3.14 (p, *J* = 9.1 Hz, 1H), 2.91 – 2.81 (m, 2H), 2.82 – 2.71 (m, 2H), 2.73 – 2.64 (m, 2H).

$^{13}C\{^1H\}$-NMR (176 MHz, $CDCl_3$) δ = 175.0, 173.5, 144.2, 143.2, 139.1, 132.9, 131.9 (2C), 131.6 (2C), 128.3 (2C), 128.2 (2C), 122.2, 120.7, 52.0, 51.8, 44.4, 41.9, 37.4, 36.0, 32.5, 29.5.

IR (Diamond-ATR): $\tilde{\nu}$ (cm^{-1}) = 2933, 1729, 1486, 1434, 1363, 1255, 1198, 1173, 1072, 1008.

HRMS (APCI, Q-TOF): $[M+H]^+$ calcd for $C_{24}H_{23}Br^{81}BrO_4^+$ = 534.9938; found: 534.9945.

Dimethyl 1',4-bis(4-(trifluoromethyl)phenyl)-[1,1'-bi(cyclobutan)]-4-ene-2,3'-dicarboxylate (3d).

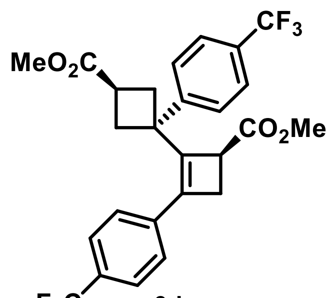

3d

Following the **GP3** taking $FeCl_3$ as LA, in CH_2Cl_2 **3d** was obtained as a colorless oil (15 mg, 29.3 µmol, 20%) along with **5d**. Purified by automated column chromatography (25g 25SI-HP cartridge, 15 mL/min) using pentane/EtOAc = 100:1 – 5:1 (Rf = 0.37 in 10:1 pentane/EtOAc). It was further purified with normal phase HPLC (Gradient, 92:8 to 80:20 hexane/EtOAc).

^1H-NMR (700 MHz, $CDCl_3$) δ = 7.62 (s, 4H), 7.53 (d, *J* = 8.1 Hz, 2H), 7.22 (d, *J* = 8.1 Hz, 2H), 3.73 (dt, *J* = 4.7, 1.9 Hz, 1H), 3.66 (s, 3H), 3.52 (s, 3H), 3.17 (p, *J* = 9.0 Hz, 1H), 2.97 – 2.90 (m, 1H), 2.90 (dd, *J* = 12.6, 2.0 Hz, 1H), 2.86 (dd, *J* = 12.6, 4.7 Hz, 1H), 2.85 – 2.79 (m, 1H), 2.80 – 2.72 (m, 2H).

$^{13}C\{^1H\}$-NMR (176 MHz, $CDCl_3$) δ = 174.8, 173.2, 148.0, 145.8, 139.4, 137.2, 129.9 (q, *J* = 32.6 Hz), 129.2 (q, *J* = 32.6 Hz,), 127.0 (2C), 126.8 (2C), 125.9 (q, J = 3.7 Hz, 2C), 125.4 (q, J = 3.8 Hz, 2C), 124.2 (q, J = 272.3 Hz), 124.1 (q, *J* = 272.8 Hz), 52.1, 51.8, 44.8, 42.1, 37.4, 36.0, 32.4, 29.8.

^{19}F NMR (659 MHz, $CDCl_3$) δ = -62.6, -62.7.

IR (Diamond-ATR): $\tilde{\nu}$ (cm^{-1}) = 1730, 1322, 1258, 1221, 1196, 1163, 1117, 1070, 1035, 1015.

HRMS (APCI, Orbitrap): $[M+H]^+$ calcd for $C_{26}H_{23}O_4F_6^+$ = 513.1495; found: 513.1490.

Dimethyl 1',4-di-*m*-tolyl-[1,1'-bi(cyclobutan)]-4-ene-2,3'-dicarboxylate (3e).

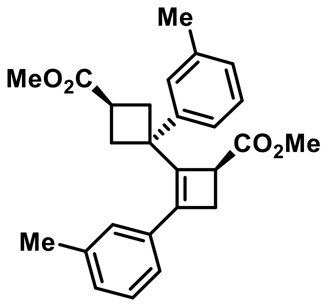

3e

Following the **GP1** taking $Bi(OTf)_3$ as LA, in CH_2Cl_2 **3e** was obtained as a colorless oil (6 mg, 14.8 μmol, 10%) along with **4e** and **5e**. Purified by automated column chromatography (25 g 25SI-HP cartridge, 15 mL/min) using pentane/EtOAc = 100:1 – 10:1 (Rf = 0.51 in 10:1 pentane/EtOAc. It was further purified with normal phase HPLC (Gradient, 95:5 to 92:8 hexane/EtOAc).

^{1}H-NMR (700 MHz, $CDCl_3$) δ = 7.32 – 7.29 (m, 2H), 7.23 (dd, *J* = 8.4, 7.4 Hz, 1H), 7.19 – 7.14 (m, 1H), 7.07 – 6.99 (m, 4H), 3.64 (s, 3H), 3.61 (dd, *J* = 4.8, 1.9 Hz, 1H), 3.49 (s, 3H), 3.13 (tt, *J* = 9.4, 8.7 Hz, 1H), 2.87 – 2.82 (m, 2H), 2.80 – 2.69 (m, 4H), 2.36 (d, *J* = 0.7 Hz, 3H), 2.30 (d, *J* = 0.8 Hz, 3H).

^{13}C{^{1}H}-NMR (176 MHz, $CDCl_3$) δ = 175.3, 174.0, 144.2, 143.9, 139.5, 138.2, 137.8, 134.3, 128.6, 128.6, 128.1, 127.6, 127.3, 127.2, 124.1, 123.6, 51.9, 51.6, 44.7, 41.8, 37.2, 36.0, 32.7, 29.6, 21.8, 21.5.

IR (Diamond-ATR): $\tilde{\nu}$ (cm^{-1}) = 2933, 1731, 1603, 1434, 1335, 1255, 1196, 1164, 1036.

HRMS (ESI, Orbitrap): $[M+Na]^+$ calcd for $C_{26}H_{28}O_4Na^+$ = 427.1880; found: 427.1881.

Dimethyl 1',4-bis(4-bromophenyl)-[1,1'-bi(cyclobutan)]-3-ene-2,3'-dicarboxylate (3b´).

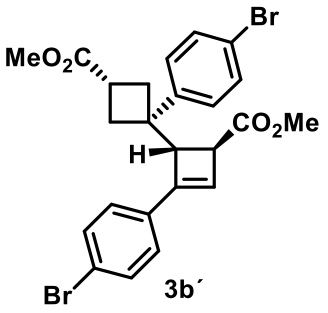

Following the **GP1**, taking $Sc(OTf)_3$ as LA in in CH_2Cl_2 (1 mmol scale) **3b´** was able to isolate as a colorless oil (10 mg, 18.7 µmol, 4%) along with **3b**, **5b** and **7** (even though traces were observed in 0.3 mmol reaction, isolable amounts were formed in 1 mmol scale reaction).. Purified by automated column chromatography (25g 30SI-HP cartridge, 15 mL/min) using pentane/EtOAc = 100:1 – 10:1 (Rf = 0.35 in 10:1 pentane/EtOAc). It was further purified with normal phase HPLC (Isocratic, 90:10 hexane/EtOAc).

^{1}H-NMR (700 MHz, $CDCl_3$) δ = 7.46 – 7.41 (m, 2H), 7.31 – 7.27 (m, 2H), 7.09 – 7.02 (m, 2H), 6.79 – 6.73 (m, 2H), 6.04 (d, *J* = 1.2 Hz, 1H), 3.86 (d, *J* = 1.8 Hz, 1H), 3.68 (s, 3H), 3.61 (s, 3H), 3.20 (p, *J* = 9.0 Hz, 1H), 3.09 (dd, *J* = 1.8, 1.2 Hz, 1H), 2.76 (ddd, *J* = 11.9, 9.0, 3.0 Hz, 1H), 2.71 – 2.62 (m, 2H), 2.54 (ddd, *J* = 11.7, 9.2, 0.6 Hz, 1H).

^{13}C{^{1}H}-NMR (176 MHz, $CDCl_3$) δ = 175.2, 173.2, 150.4, 145.2, 133.5, 131.8 (2C), 130.9 (2C), 128.4 (2C), 127.6 (2C), 127.1, 122.5, 120.2, 54.6, 52.2, 52.0, 44.8, 44.0, 35.9, 34.1, 32.7.

IR (Diamond-ATR): $\tilde{\nu}$ (cm^{-1}) = 1726, 1485, 1434, 1395, 1342, 1252, 1206, 1166, 1054, 1009.

HRMS (ESI, Orbitrap): $[M+H]^+$ calcd for $C_{24}H_{23}Br^{81}BrO_4^+$ = 534.9938; found: 534.9927.

Dimethyl 2a'-phenyl-1',2',2a',7a'-tetrahydrospiro[cyclobutane-1,7'-cyclobuta[*a*]indene]-1',3-dicarboxylate (4a).

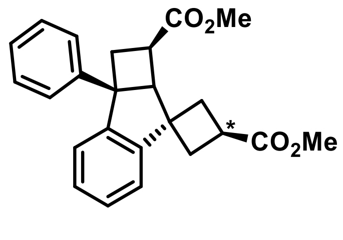

4a

Following the **GP1**, taking $Sc(OTf)_3$ as LA, **4a** was obtained as a colorless oil (8.5 mg, 22.6 μmol, 16%) as a diastereomeric mixture(dr= 2:1) along with **3a** and **5a** . Purified by automated column chromatography (25g 25SI-HP cartridge, 15 mL/min) using pentane/EtOAc = 100:1 – 10:1. The diastereomers were separated by normal phase HPLC (Gradient, 95:5 to 90:10 hexane/EtOAc).

4a can also be achieved as a diastereomeric mixture (dr = 2:1) following **GP2** (51.3 mg, 136 μmol, 91%).

Major- (Rf = 0.51 in 10:1 Pentane/EtOAc).

^{1}**H-NMR** (700 MHz, $CDCl_3$) δ =7.50 (ddt, *J* = 7.7, 1.3, 0.6 Hz, 1H), 7.33 (td, *J* = 7.5, 1.2 Hz, 1H), 7.30 – 7.25 (m, 2H), 7.22 (td, *J* = 7.5, 1.1 Hz, 1H), 7.21 – 7.13 (m, 1H), 7.15 – 7.10 (m, 2H), 7.07 (ddt, *J* = 7.7, 1.2, 0.6 Hz, 1H), 3.74 (s, 3H), 3.72 (s, 3H), 3.42 (tt, *J* = 9.5, 7.8 Hz, 1H), 3.37 (d, *J* = 7.3 Hz, 1H), 3.02 (ddd, *J* = 11.3, 9.3, 0.8 Hz, 1H), 2.86 (ddd, *J* = 12.2, 7.9, 1.1 Hz, 1H), 2.71 – 2.64 (m, 3H), 2.49 (ddd, *J* = 12.0, 9.3, 2.7 Hz, 1H), 2.45 (ddt, *J* = 11.2, 8.7, 0.7 Hz, 1H).

^{13}**C{^{1}H}-NMR** (176 MHz, $CDCl_3$) δ = 175.8, 175.3, 149.3, 148.4, 146.4, 128.6 (2C), 128.3, 128.2, 126.2, 125.8 (2C), 125.0, 122.9, 61.1, 54.5, 52.0, 52.01, 49.4, 40.6, 36.2, 34.1, 32.3, 32.3.

Minor- (Rf = 0.61 in 10:1 pentane/EtOAc).

^{1}**H-NMR** (700 MHz, $CDCl_3$) δ = 7.56 – 7.51 (m, 1H), 7.34 (td, *J* = 7.5, 1.1 Hz, 1H), 7.30 – 7.25 (m, 2H), 7.22 (td, *J* = 7.4, 1.2 Hz, 1H), 7.20 – 7.13 (m, 3H), 7.05 (ddd, *J* = 7.6, 1.2, 0.7 Hz, 1H), 3.75 (s, 3H), 3.72 (s, 3H), 3.41 (dd, *J* = 7.3, 0.7 Hz, 1H), 3.21 (p, *J* = 9.1 Hz, 1H), 3.08 (dd, *J* = 11.3, 9.3 Hz, 1H), 2.82 (dd, *J* = 11.8, 9.4 Hz, 1H), 2.74 (dd,

J = 9.1, 7.3 Hz, 1H), 2.63 (ddt, J = 12.0, 8.8, 1.8 Hz, 1H), 2.51 (dd, J = 9.1, 1.7 Hz, 2H), 2.48 (ddd, J = 11.3, 9.0, 0.8 Hz, 1H).

^{13}C{^{1}H}-NMR (176 MHz, $CDCl_3$) δ = 175.9, 175.5, 148.3, 148.2, 146.2, 128.6 (2C), 128.3, 128.3, 126.3, 126.0 (2C), 124.9, 124.3, 60.0, 54.6, 52.1, 51.9, 49.3, 41.1, 36.8, 34.0, 32.0, 31.8.

IR (Diamond-ATR): $\tilde{\nu}$ (cm^{-1}) = 1728, 1434, 1359, 1249, 1197, 1168, 1102, 1090, 1049, 1028.

HRMS (ESI,Q-TOF): $[M+Na]^+$ calcd for $C_{24}H_{24}O_4Na^+$ = 399.1567; found: 399.1572.

Dimethyl 4'-bromo-2a'-(4-bromophenyl)-1',2',2a',7a'-tetrahydrospiro[cyclobutane-1,7'-cyclobuta[a]indene]-1',3-dicarboxylate (4b).

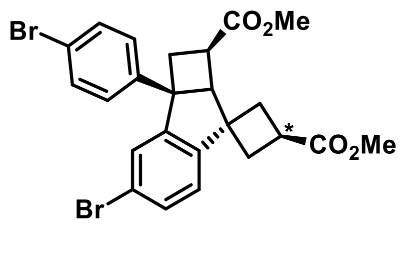

4b

Following the **GP2**, **4b** is obtained as a mixture of diastereomers (dr =2:1) as a colorless oil (61mg, 114.2 µmol, 76%). The major and minor diastereomers were by automated column chromatography (25g 25SI-HP cartridge, 15 mL/min) using pentane/EtOAc = 100:1 – 100:15.

Major (Rf = 0.37 in 10:1 pentane/EtOAc).

^{1}H-NMR (700 MHz, $CDCl_3$) δ = 7.45 (dd, J = 8.2, 1.9 Hz, 1H), 7.43 – 7.39 (m, 2H), 7.37 (dd, J = 8.3, 0.5 Hz, 1H), 7.13 (dd, J = 1.9, 0.4 Hz, 1H), 7.01 – 6.93 (m, 2H), 3.73 (s, 3H), 3.72 (s, 3H), 3.37 (tt, J = 9.5, 7.6 Hz, 1H), 3.32 (dd, J = 7.3, 0.7 Hz, 1H), 2.97 (dd, J = 11.4, 9.3 Hz, 1H), 2.83 (ddt, J = 12.4, 7.7, 1.2 Hz, 1H), 2.67 – 2.60 (m, 3H), 2.48 – 2.39 (m, 2H).

^{13}C{^{1}H}-NMR (176 MHz, $CDCl_3$) δ = 175.5, 174.8, 150.0, 148.4, 144.5, 131.8 (2C), 131.8, 128.1, 127.6 (2C), 124.7, 121.8, 120.5, 61.1, 54.1, 52.2, 52.1, 49.1, 40.4, 36.1, 33.8, 32.2, 32.1.

Minor-(Rf = 0.48 in 10:1 pentane/EtOAc).

^{1}H-NMR (700 MHz, $CDCl_3$) δ = 7.47 (dd, *J* = 8.2, 1.8 Hz, 1H), 7.47 – 7.38 (m, 3H), 7.12 (dd, *J* = 1.9, 0.5 Hz, 1H), 6.99 – 6.97 (m, 2H), 3.74 (s, 3H), 3.73 (s, 3H), 3.35 (dd, *J* = 7.4, 0.7 Hz, 1H), 3.18 (p, *J* = 9.0 Hz, 1H), 3.02 (dd, *J* = 11.5, 9.3 Hz, 1H), 2.77 (dd, *J* = 12.0, 9.3 Hz, 1H), 2.70 (td, *J* = 9.1, 7.4 Hz, 1H), 2.67 – 2.57 (m, 1H), 2.50 – 2.39 (m, 3H).

^{13}C{^{1}H}-NMR (176 MHz, $CDCl_3$) δ = 175.7, 175.0, 149.8, 147.4, 144.4, 131.8 (2C), 131.8, 128.0, 127.8 (2C), 126.1, 122.0, 120.6, 60.2, 54.2, 52.3, 52.0, 48.9, 40.9, 36.6, 33.6, 31.8, 31.6.

IR (Diamond-ATR): $\tilde{\nu}$ (cm^{-1}) = 1727, 1488, 1434, 1360, 1255, 1201, 1168, 1071, 1047, 1009.

HRMS (ESI, Orbitrap): $[M+Na]^+$ calcd for $C_{24}H_{22}Br^{81}BrO_4Na^+$ = 556.9757; found: 556.9763.

Dimethyl 5'-methyl-2a'-(*m*-tolyl)-1',2',2a',7a'-tetrahydrospiro[cyclobutane-1,7'-cyclobuta[*a*]indene]-1',3-dicarboxylate (4e).

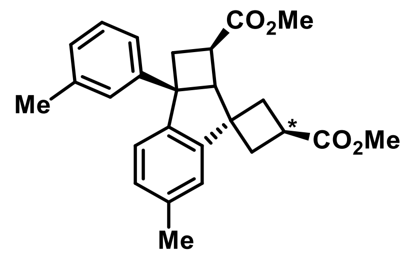

4e

Following the **GP2, 4e** is obtained as a colorless oil (31.0 mg, 76.6 µmol, 51%). Purified by automated column chromatography (25g 25SI-HP cartridge, 15 mL/min) using 10:1 pentane/EtOAc = 100:1 – 10:1 (Rf = 0.38 in 10:1 pentane/EtOAc) The fraction containing a mix of 2 regioisomers were subjected to normal phase HPLC (95:5 hexane/EtOAc). The title compound was isolated as 1.5:1 a mixture of diastereomers.

4e was also obtained in 54% (dr= 1.8:1) following **GP1** using $Bi(OTf)_3$.

Major

^{1}H-NMR (700 MHz, $CDCl_3$, major diast.) δ = 7.39 – 7.36 (m, 1H), 7.35 – 7.27 (m, 1H), 7.12 (t, J = 7.6 Hz, 1H), 7.02 – 6.99 (m, 1H), 6.97 – 6.95 (m, 1H), 6.90 – 6.88 (m, 1H),

6.84 – 6.82 (m, 1H), 3.72 (s, 3H), 3.69 (s, 3H), 3.38 (ddd, *J* = 9.5, 7.5, 2.0 Hz, 1H), 3.21 (dd, *J* = 11.5, 9.5 Hz, 1H), 3.16 – 3.12 (m, 1H), 2.82 – 2.78 (m, 1H), 2.71 – 2.61 (m, 2H), 2.56 – 2.52 (m, 1H), 2.49 (tdd, *J* = 9.5, 2.3, 1.0 Hz, 1H), 2.35 (ddd, *J* = 11.5, 8.7, 1.0 Hz, 1H), 2.28 (s, 3H), 1.90 (s, 3H).

$^{13}C\{^1H\}$-NMR (176 MHz, $CDCl_3$.) δ = 175.8, 175.4, 150.3, 145.7, 144.8, 137.8, 135.4, 129.8, 128.9, 128.2, 127.1, 126.8, 123.5, 120.4, 62.6, 54.1, 52.0, 51.9, 49.2, 40.5, 36.3, 32.4, 32.3, 30.0, 21.7, 18.5.

Minor

^1H-NMR (700 MHz, $CDCl_3$, minor diast.) δ = 7.32 – 7.29 (m, 1H), 7.15 (t, *J* = 7.6 Hz, 1H), 7.03 (ddd, *J* = 7.8, 1.6, 0.7 Hz, 1H), 6.97 – 6.94 (m, 2H), 6.92 – 6.91 (m, 1H), 6.90 – 6.88 (m, 1H), 3.73 (s, 3H), 3.70 (s, 3H), 3.44 – 3.39 (m, 1H), 3.34 – 3.32 (m, 1H), 2.97 (dd, *J* = 11.2, 9.3 Hz, 1H), 2.85 – 2.82 (m, 1H), 2.71 – 2.61 (m, 3H), 2.50 – 2.46 (m, 1H), 2.42 – 2.38 (m, 1H), 2.39 (s, 3H), 2.29 (s, 3H).

$^{13}C\{^1H\}$-NMR (176 MHz, $CDCl_3$, minor diast.) δ = 175.8, 175.3, 149.4, 146.5, 145.7, 138.0, 138.0, 129.2, 128.4, 126.9, 126.4, 124.6, 123.3, 122.8, 61.3, 54.1, 52.0, 52.0, 49.2, 40.6, 36.2, 34.1, 32.3, 32.3, 21.7, 21.6.

IR (Diamond-ATR): $\tilde{\nu}$ (cm^{-1}) = 3053, 2979, 2948, 2846, 1726, 1630, 1599, 1505, 1434, 1358, 1270, 1249, 1198, 1167, 1034.

HRMS (APCI,Q-TOF): $[M+H]^+$ calcd for $C_{24}H_{25}O_4^+$ = 405.2060; found: 405.2067.

Dimethyl 9a'-(naphthalen-2-yl)-7a',8',9',9a'-tetrahydrospiro[cyclobutane-1,7'-cyclobuta[4,5]cyclopenta[1,2-*a*]naphthalene]-3,8'-dicarboxylate (4f).

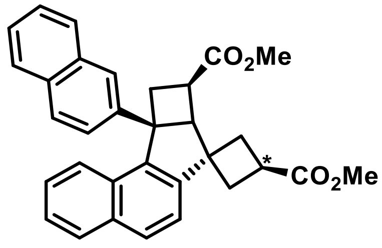

4f

Following the **GP2, 4f** is obtained as a colorless oil (59.1 mg, 124.0 µmol, 83%). Purified by automated column chromatography (25g 25SI-HP cartridge, 15 mL/min)

using 10:1 pentane/EtOAc = 100:1 – 10:1 (Rf = 0.41 in 10:1 pentane/EtOAc) The title compound was isolated as a single diastereomer from a 3:1 mixture.

Following the **GP1**, taking $Bi(OTf)_3$ as LA, in CH_2Cl_2 **4f** was obtained in 72% yield.

Following the **GP3**, taking $FeCl_3$ as LA, in CH_2Cl_2 **4f** was obtained in 24% yield.

Following the **GP3**, taking $FeCl_3$ as LA, in MeCN **4f** was obtained in 12% yield.

^{1}H-NMR (700 MHz, $CDCl_3$) δ = 7.96 (dd, J = 8.6, 0.8 Hz, 1H), 7.88 (ddd, *J* = 8.6, 1.2, 0.5 Hz, 1H), 7.81 (d, *J* = 1.9 Hz, 1H), 7.80 (ddd, J = 8.1, 1.3, 0.6 Hz, 1H), 7.75 (ddd, *J* = 8.0, 1.5, 0.7 Hz, 1H), 7.72 (d, *J* = 8.5 Hz, 1H), 7.66 (dd, *J* = 8.5, 0.7 Hz, 1H), 7.52 (dd, *J* = 8.4, 1.1 Hz, 1H), 7.45 (ddd, *J* = 8.2, 6.8, 1.3 Hz, 1H), 7.42 (ddd, *J* = 8.1, 6.8, 1.4 Hz, 1H), 7.36 (ddd, *J* = 8.1, 6.8, 1.2 Hz, 1H), 7.24 (ddd, J = 8.2, 6.8, 1.3 Hz, 1H), 7.11 (dd, *J* = 8.6, 1.9 Hz, 1H), 3.76 (s, 3H), 3.71 (s, 3H), 3.57 (dd, *J* = 11.6, 9.1 Hz, 1H), 3.51 – 3.43 (m, 1H), 3.37 (dd, *J* = 7.4, 0.7 Hz, 1H), 2.94 – 2.89 (m, 1H), 2.84 – 2.75 (m, 2H), 2.70 – 2.60 (m, 3H).

^{13}C{^{1}H}-NMR (176 MHz, $CDCl_3$) δ = 175.8, 175.4, 147.1, 142.9, 141.6, 134.2, 133.4, 132.2, 130.2, 129.6, 128.9, 128.4, 128.1, 127.6, 126.6, 126.2, 125.7, 125.5, 125.1, 124.8, 124.7, 121.1, 63.5, 54.7, 52.1, 52.0, 50.1, 40.0, 36.9, 32.6, 32.1, 30.4.

IR (Diamond-ATR): $\tilde{\nu}$ (cm^{-1}) = 2945, 1727, 1434, 1356, 1270, 1249, 1200, 1169, 1099, 1036.

HRMS (ESI, Orbitrap): $[M+Na]^+$ calcd for $C_{32}H_{28}O_4Na^+$ = 499.1880; found: 499.1877.

Dimethyl 4'-chloro-2a'-(4-chlorophenyl)-1',2',2a',7a'-tetrahydrospiro[cyclobutane-1,7'-cyclobuta[*a*]indene]-1',3-dicarboxylate (4g).

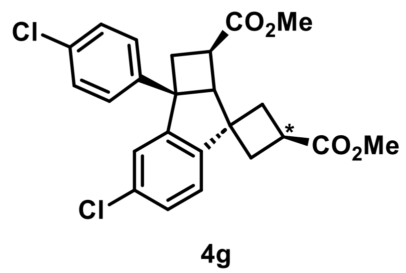

4g

Following the **GP2**, **4g** is obtained as a colorless oil (62 mg, 139.2 µmol, 93%). Purified by automated column chromatography (25g 25SI-HP cartridge, 15 mL/min) using 10:1

pentane/EtOAc = 100:1 – 10:1 (Rf = 0.29 in 10:1 pentane/EtOAc). Both diastereomers of the title compound were isolated (dr = 2:1)

Major

^{1}H-NMR (700 MHz, $CDCl_3$) δ = 7.42 (dd, *J* = 8.2, 0.5 Hz, 1H), 7.32 – 7.29 (m, 1H), 7.27 – 7.24 (m, 2H), 7.03 – 7.00 (m, 2H), 6.98 (dd, *J* = 2.0, 0.5 Hz, 1H), 3.73 (s, 3H), 3.72 (s, 3H), 3.41 – 3.34 (m, 1H), 3.33 (dd, *J* = 7.3, 0.6 Hz, 1H), 2.98 (dd, *J* = 11.4, 9.3 Hz, 1H), 2.87 – 2.81 (m, 1H), 2.67 – 2.60 (m, 3H), 2.48 – 2.39 (m, 2H).

^{13}C{^{1}H}-NMR (176 MHz, $CDCl_3$, major diast.) δ = 175.5, 174.8, 149.7, 147.8, 144.0, 133.8, 132.4, 128.9 (2C), 128.8, 127.1 (2C), 125.0, 124.2, 61.2, 54.0, 52.1, 52.0, 49.0, 40.4, 36.0, 33.8, 32.1, 32.1.

Minor

^{1}H-NMR (700 MHz, $CDCl_3$, minor diast.) δ = 7.47 (dd, *J* = 8.3, 0.5 Hz, 1H), 7.31 (dd, *J* = 8.3, 2.0 Hz, 1H), 7.27 – 7.24 (m, 2H), 7.06 – 7.03 (m, 2H), 6.96 (dd, *J* = 2.0, 0.5 Hz, 1H), 3.74 (s, 3H), 3.73 (s, 3H), 3.36 (dd, *J* = 7.4, 0.7 Hz, 1H), 3.18 (p, *J* = 9.1 Hz, 1H), 3.03 (dd, J = 11.6, 9.1 Hz, 1H), 2.77 (dd, *J* = 12.0, 9.2 Hz, 1H), 2.71 (td, *J* = 9.2, 7.4 Hz, 1H), 2.66 – 2.60 (m, 1H), 2.49 – 2.43 (m, 3H).

^{13}C{^{1}H}-NMR (176 MHz, $CDCl_3$, minor diast.) δ = 175.6, 175.0, 149.4, 146.9, 143.8, 134.0, 132.5, 128.8, 128.8 (2C), 127.3 (2C), 125.7, 124.9, 60.2, 54.1, 52.2, 52.0, 48.8, 40.9, 36.6, 33.6, 31.8, 31.7.

IR (Diamond-ATR): $\tilde{\nu}$ (cm^{-1}) = 2979, 2949, 1729, 1595, 1571, 1491, 1472, 1462, 1435, 1408, 1398, 1360, 1305, 1281, 1255, 1227, 1199, 1169, 1116, 1091, 1047, 1033, 1014.

HRMS (ESI,Q-TOF): $[M+H]^+$ calcd for $C_{24}H_{23}O_4Cl_2^+$ = 445.0968; found: 445.0974.

Dimethyl 4'-(trifluoromethoxy)-2a'-(4-(trifluoromethoxy)phenyl)-1',2',2a',7a'-tetrahydrospiro[cyclobutane-1,7'-cyclobuta[*a*]indene]-1',3-dicarboxylate (4h).

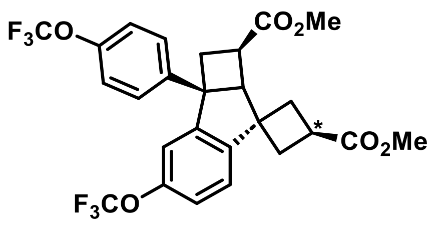

4h

Following the **GP2**, **4h** is obtained as colorless oil (69 mg, 126.7 μmol, 85%). Purified by automated column chromatography (25g 30SI-HP cartridge, 15 mL/min) using 10:1 pentane/EtOAc = 100:1 – 10:1 (Rf = 0.17 in 10:1 pentane/EtOAc). Both diastereomers of the title compound were isolated (dr = 2:1).

Major

^{1}H-NMR (700 MHz, $CDCl_3$, major diast.) δ = 7.50 (d, *J* = 8.4 Hz, 1H), 7.21 – 7.19 (m, 1H), 7.16 – 7.13 (m, 2H), 7.13 – 7.10 (m, 2H), 6.86 (dd, *J* = 2.2, 1.1 Hz, 1H), 3.73 (s, 3H), 3.73 (s, 3H), 3.41 – 3.35 (m, 2H), 3.01 (dd, *J* = 11.5, 9.4 Hz, 1H), 2.85 (ddt, *J* = 12.4, 7.6, 1.2 Hz, 1H), 2.70 – 2.63 (m, 3H), 2.49 – 2.42 (m, 2H).

^{13}C{^{1}H}-NMR (176 MHz, $CDCl_3$, major diast.) δ = 175.4, 174.7, 149.7, 149.4 (q, J = 1.9 Hz), 147.9, 147.8 (q, J = 2.1 Hz), 144.0, 127.1 (2C), 124.2, 121.5, 121.3 (2C), 120.6 (q, J = 257.1 Hz), 120.5 (q, J = 257.1 Hz), 117.6, 61.3, 54.0, 52.2, 52.1, 49.0, 40.4, 36.0, 34.0, 32.1, 32.1.

^{19}F-NMR (659 MHz, $CDCl_3$, major diast.): δ = -57.90, -57.92.

Minor

^{1}H-NMR (700 MHz, $CDCl_3$, minor diast.) δ = 7.56 (dd, *J* = 8.4, 0.4 Hz, 1H), 7.20 (ddq, J = 8.4, 2.1, 1.1 Hz, 1H), 7.14 (s, 4H), 6.86 – 6.84 (m, 1H), 3.74 (s, 3H), 3.74 (s, 3H), 3.41 (dd, *J* = 7.4, 0.7 Hz, 1H), 3.19 (p, *J* = 9.0 Hz, 1H), 3.06 (dd, *J* = 11.6, 9.0 Hz, 1H), 2.79 (dd, *J* = 12.0, 9.0 Hz, 1H), 2.74 (td, *J* = 9.0, 7.3 Hz, 1H), 2.64 (ddd, *J* = 12.0, 9.0, 2.9 Hz, 1H), 2.50 – 2.45 (m, 3H).

^{13}C{^{1}H}-NMR (176 MHz, $CDCl_3$, minor diast.) δ = 175.6, 174.9, 149.5 (q, J = 2.1 Hz), 149.4, 147.9 (q, J = 1.9 Hz), 147.0, 143.9, 127.4 (2C), 125.6, 122.7, 121.4, 121.2, 120.5 (q, J = 257.1 Hz), 120.5 (q, J = 257.1 Hz), 60.4, 54.0, 52.2, 52.0, 48.7, 40.9, 36.5, 33.8, 31.8, 31.7.

^{19}F-NMR (659 MHz, $CDCl_3$, minor diast.): δ = -57.89, -57.92.

IR (Diamond-ATR): $\tilde{\nu}$ (cm^{-1}) = 2980, 2953, 1731, 1609, 1588, 1508, 1481, 1436, 1362, 1308, 1251, 1205, 1161, 1114, 1048, 1033, 1019.

HRMS (ESI,Q-TOF): $[M+NH_4]^+$ calcd for $C_{26}H_{26}NO_4F_6^+$ = 562.1659; found: 562.1657.

Dimethyl 4'-methoxy-2a'-(4-methoxyphenyl)-1',2',2a',7a'-tetrahydrospiro[cyclobutane-1,7'-cyclobuta[*a*]indene]-1',3-dicarboxylate (4i).

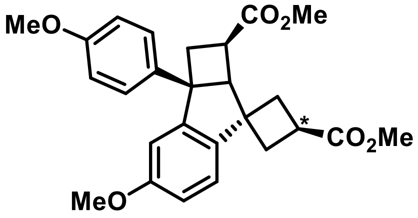

4i

Following the **GP1**, taking $FeCl_3$ as LA, in CH_2Cl_2 **4i ,** major diastereomer was isolated as a colorless oil (11.1 mg, 25.4 µmol, 16%). Purified by automated column chromatography (25g 25SI-HP cartridge, 15 mL/min) using pentane/EtOAc = 100:1 – 4:1 (Rf = 0.22 in 10:1 pentane/EtOAc).

^{1}H-NMR (700 MHz, $CDCl_3$) δ = 7.38 (dd, *J* = 8.3, 0.4 Hz, 1H), 7.06 – 7.01 (m, 2H), 6.87 (dd, *J* = 8.4, 2.5 Hz, 1H), 6.84 – 6.80 (m, 2H), 6.55 (dd, *J* = 2.5, 0.4 Hz, 1H), 3.77 (s, 3H), 3.73 (s, 3H), 3.72 (s, 3H), 3.71 (s, 3H), 3.37 (tt, *J* = 9.4, 7.7 Hz, 1H), 3.28 (dd, *J* = 7.3, 0.7 Hz, 1H), 2.98 (dd, *J* = 11.3, 9.3 Hz, 1H), 2.82 (ddt, *J* = 12.2, 7.7, 1.1 Hz, 1H), 2.65 (ddd, *J* = 9.4, 8.8, 7.3 Hz, 1H), 2.65 – 2.58 (m, 2H), 2.46 – 2.41 (m, 1H), 2.40 (ddd, *J* = 11.3, 8.7, 0.6 Hz, 1H).

^{13}C{^{1}H}-NMR (176 MHz, $CDCl_3$) δ = 175.8, 175.4, 160.0, 158.1, 150.2, 141.6, 138.5, 126.9 (2C), 123.6, 114.6, 114.0 (2C), 109.6, 61.8, 55.6, 55.4, 54.0, 52.0, 52.0, 48.8, 40.7, 36.2, 33.9, 32.5, 32.3.

IR (Diamond-ATR): $\tilde{\nu}$ (cm^{-1}) = 1727, 1511, 1487, 1463, 1435, 1246, 1226, 1200, 1172, 1032.

HRMS (ESI, Orbitrap): $[M+Na]^+$ calcd for $C_{26}H_{28}O_6Na^+$ = 459.1778; found: 459.1770.

(2a'-phenyl-1',2',2a',7a'-tetrahydrospiro[cyclobutane-1,7'-cyclobuta[*a*]indene]-1',3-diyl)bis(phenylmethanone) (4j).

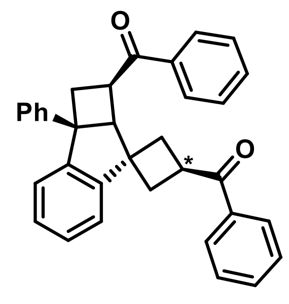

4j

Following the **GP2**, **4j** is obtained as a mixture of diastereomers (dr = 2:1) as a colorless solid (23 mg, 49.1 μmol, 32%). The major and minor diastereomers were separated by automated column chromatography (25g 25SI-HP cartridge, 15 mL/min) using pentane/EtOAc = 100:1 – 10:1.

Major- (Rf = 0.47 in 10:1 pentane/EtOAc), **m.p.**: 173.5.- 174.2 °C.

^{1}H-NMR (700 MHz, $CDCl_3$) δ = 7.97 – 7.93 (m, 2H), 7.84 – 7.79 (m, 2H), 7.69 (ddd, *J* = 7.6, 1.1, 0.6 Hz, 1H), 7.57 – 7.52 (m, 1H), 7.52 – 7.47 (m, 1H), 7.48 – 7.43 (m, 2H), 7.43 (td, *J* = 7.5, 1.2 Hz, 1H), 7.42 – 7.36 (m, 2H), 7.30 (td, *J* = 7.5, 1.1 Hz, 1H), 7.27 – 7.24 (m, 2H), 7.18 (ddd, *J* = 7.7, 1.2, 0.6 Hz, 1H), 7.16 – 7.13 (m, 3H), 4.22 (p, *J* = 8.9 Hz, 1H), 3.76 (dd, *J* = 7.1, 0.7 Hz, 1H), 3.48 (td, *J* = 9.5, 7.1 Hz, 1H), 2.92 (dd, *J* = 11.6, 8.7 Hz, 1H), 2.82 (dd, *J* = 11.0, 9.6 Hz, 1H), 2.77 – 2.69 (m, 2H), 2.66 (dddd, *J* = 11.9, 9.3, 3.0, 0.8 Hz, 1H), 2.61 (dddd, J = 11.6, 9.5, 3.1, 0.9 Hz, 1H).

^{13}C{^{1}H}-NMR (176 MHz, $CDCl_3$) δ = 200.3, 200.0, 150.5, 148.9, 146.4, 135.7, 135.6, 133.1, 133.1, 128.7 (2C), 128.7 (2C), 128.6 (2C), 128.6 (2C), 128.5 (2C), 128.4, 128.2, 126.2, 125.8 (2C),125.1, 122.8, 57.9, 54.3, 49.0, 40.9, 40.2, 37.8, 36.4, 32.4.

Minor- (Rf = 0.71 in 10:1 pentane/EtOAc), **m.p.**: sticky oil.

^{1}H-NMR (700 MHz, $CDCl_3$) δ = 7.98 – 7.94 (m, 2H), 7.92 – 7.86 (m, 2H), 7.59 – 7.53 (m, 2H), 7.51 (ddd, *J* = 7.7, 1.2, 0.7 Hz, 1H), 7.50 – 7.42 (m, 4H), 7.36 (td, *J* = 7.5, 1.2 Hz, 1H), 7.31 – 7.22 (m, 3H), 7.20 – 7.13 (m, 3H), 7.13 (ddd, *J* = 7.6, 1.2, 0.7 Hz, 1H), 4.17 (p, *J* = 9.0 Hz, 1H), 4.02 (dd, *J* = 7.3, 0.7 Hz, 1H), 3.54 (td, *J* = 9.6, 7.3 Hz, 1H), 2.99 – 2.90 (m, 2H), 2.81 (ddd, *J* = 11.1, 9.6, 0.8 Hz, 1H), 2.73 (dddd, *J* = 11.5, 8.9,

4.0, 0.8 Hz, 1H), 2.62 (ddd, *J* = 11.4, 9.1, 0.7 Hz, 1H), 2.33 (dddd, *J* = 11.9, 8.8, 3.9, 0.8 Hz, 1H).

^{13}C{^{1}H}-NMR (176 MHz, $CDCl_3$) δ = 201.4, 200.9, 149.2, 148.4, 146.3, 135.8, 135.5, 133.4, 133.2, 128.8 (2C), 128.8 (2C), 128.6 (4C), 128.6 (2C), 128.4, 128.2, 126.3, 126.1 (2C), 124.9, 124.5, 57.3, 54.4, 49.0, 41.4, 41.4, 37.5, 36.0, 31.6.

IR (Diamond-ATR): $\tilde{\nu}$ (cm^{-1}) = 1671, 1466, 1456, 1445, 1276, 1257, 1217, 1180, 1125, 1024.

HRMS (ESI, Q-TOF): $[M+Na]^+$ calcd for $C_{34}H_{28}O_2Na^+$ = 491.1982; found: 491.1984.

(2a'-phenyl-1',2',2a',7a'-tetrahydrospiro[cyclobutane-1,7'-cyclobuta[*a*]indene]-1',3-diyl)bis(naphthalen-2-ylmethanone) (4k).

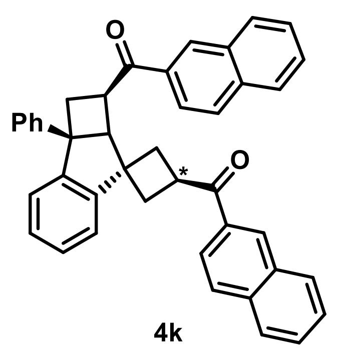

4k

Following the **GP2**, **4k** is obtained as a mixture of diastereomers (dr = 3:2) as a colorless solid (51 mg, 89.7 µmol, 60%). The major and minor diastereomers were separated by automated column chromatography (25 g 25SI-HP cartridge, 15 mL/min) using pentane/EtOAc = 100:1 – 10:1.

Major- (Rf = 0.41 in 10:1 Pentane/EtOAc), **m.p.**: sticky oil.

^{1}H-NMR (700 MHz, $CDCl_3$) δ = 8.41 (s, 1H), 8.27 (s, 1H), 8.03 (dd, J = 8.6, 1.7 Hz, 1H), 7.96 – 7.91 (m, 2H), 7.90 – 7.83 (m, 3H), 7.82 – 7.76 (m, 3H), 7.57 (ddd, *J* = 8.2, 6.8, 1.3 Hz, 1H), 7.53 (dtd, *J* = 7.9, 6.6, 1.3 Hz, 2H), 7.51 – 7.46 (m, 2H), 7.35 (td, *J* = 7.5, 1.1 Hz, 1H), 7.28 – 7.21 (m, 3H), 7.20 – 7.11 (m, 3H), 4.36 (p, *J* = 8.8 Hz, 1H), 3.85 (d, *J* = 7.1 Hz, 1H), 3.66 (td, *J* = 9.5, 7.1 Hz, 1H), 3.00 (dd, *J* = 11.7, 8.6 Hz, 1H), 2.90 (dd, *J* = 11.1, 9.5 Hz, 1H), 2.86 – 2.80 (m, 2H), 2.80 – 2.74 (m, 1H), 2.71 (dddd, *J* = 12.5, 9.5, 2.8, 0.8 Hz, 1H).

^{13}C{^{1}H}-NMR (176 MHz, $CDCl_3$) δ = 200.3, 200.0, 150.6, 149.0, 146.4, 135.7, 135.7, 133.1, 132.9, 132.7, 132.6, 130.0, 130.0, 129.6, 129.6, 128.6, 128.6 (2C), 128.6, 128.5, 128.4 (2C), 128.2, 127.9, 127.9, 126.8, 126.8, 126.2, 125.8 (2C), 125.2, 124.4, 124.3, 123.0, 58.0, 54.4, 49.2, 41.1, 40.2, 37.9, 36.5, 32.4.

Minor- (Rf = 0.59 in 10:1 pentane/EtOAc), **m.p.**: 203.3- 204.2°C.

^{1}H-NMR (700 MHz, $CDCl_3$) δ = 8.46 (s, 1H), 8.38 (s, 1H), 8.07 – 8.01 (m, 2H), 7.99 – 7.95 (m, 1H), 7.95 – 7.85 (m, 5H), 7.63 – 7.57 (m, 3H), 7.54 (dddd, *J* = 8.1, 6.6, 5.0, 1.3 Hz, 2H), 7.40 (td, *J* = 7.5, 1.2 Hz, 1H), 7.30 (tdd, *J* = 7.2, 2.9, 1.4 Hz, 3H), 7.24 – 7.14 (m, 4H), 4.36 (p, *J* = 8.9 Hz, 1H), 4.13 (d, *J* = 7.2 Hz, 1H), 3.74 (td, *J* = 9.6, 7.3 Hz, 1H), 3.07 (dd, *J* = 11.9, 9.0 Hz, 1H), 3.02 (dd, *J* = 11.1, 9.7 Hz, 1H), 2.95 – 2.89 (m, 1H), 2.83 (ddd, *J* = 12.5, 8.7, 3.8 Hz, 1H), 2.69 (dd, *J* = 11.6, 9.1 Hz, 1H), 2.47 – 2.40 (m, 1H).

^{13}C{^{1}H}-NMR (176 MHz, $CDCl_3$) δ = 201.4, 200.9, 149.4, 148.6, 146.3, 135.9, 135.8, 133.1, 132.8, 132.8, 132.7, 130.3, 130.3, 129.8, 129.7, 128.8, 128.7, 128.7, 128.6 (2C), 128.6, 128.5, 128.2, 128.0, 127.9, 127.0, 126.8, 126.3, 126.1 (2C), 125.0, 124.6, 124.3, 124.3, 57.5, 54.5, 49.1, 41.7, 41.4, 37.6, 36.0, 31.6.

IR (Diamond-ATR): $\tilde{\nu}$ (cm^{-1}) = 1669, 1466, 1455, 1445, 1275, 1257, 1217, 1186, 1176, 1125.

HRMS (APCI, Q-TOF): $[M+H]^+$ calcd for $C_{42}H_{33}O_2^+$ = 569.2475; found: 569.2475.

(2a'-phenyl-1',2',2a',7a'-tetrahydrospiro[cyclobutane-1,7'-cyclobuta[*a*]indene]-1',3-diyl)bis((3,5-dimethyl-1H-pyrazol-1-yl)methanone) (4l).

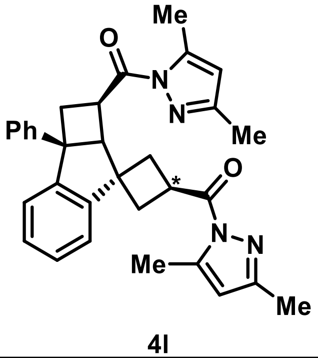

4l

Following the **GP2**, **4l** is obtained as a mixture of diastereomers (dr = 3:1) as a colorless solid (36.4mg, 71.3 µmol, 48%). The major and minor diastereomers were separated

by automated column chromatography (25 g 25SI-HP cartridge, 15 mL/min) using pentane/EtOAc = 100:1 – 10:1.

Major- (Rf = 0.6 in 10:1 pentane/EtOAc), **m.p.**: 159.5.- 160.3°C.

^{1}H-NMR (700 MHz, $CDCl_3$) δ = 7.66 (dt, *J* = 7.7, 1.0 Hz, 1H), 7.37 (td, *J* = 7.5, 1.2 Hz, 1H), 7.30 – 7.22 (m, 3H), 7.20 – 7.10 (m, 4H), 5.94 (d, *J* = 1.1 Hz, 1H), 5.91 (d, *J* = 1.1 Hz, 1H), 4.46 (tt, *J* = 9.3, 8.3 Hz, 1H), 3.81 (td, *J* = 9.3, 7.3 Hz, 1H), 3.68 (dd, *J* = 7.3, 0.7 Hz, 1H), 2.91 (dd, *J* = 11.2, 9.3 Hz, 1H), 2.88 – 2.80 (m, 1H), 2.82 – 2.70 (m, 3H), 2.66 – 2.60 (m, 1H), 2.57 (d, *J* = 1.0 Hz, 3H), 2.54 (d, *J* = 1.0 Hz, 3H), 2.23 (s, 3H), 2.14 (s, 3H).

^{13}C{^{1}H}-NMR (176 MHz, $CDCl_3$) δ = 175.2, 175.1, 152.0, 151.9, 150.1, 148.6, 146.8, 144.2, 144.1, 128.5 (2C), 128.2, 128.0, 126.1, 125.9 (2C), 125.0, 123.1, 111.2, 110.9, 58.8, 54.4, 49.3, 40.9, 37.2, 37.1, 33.6, 32.8, 14.5, 14.5, 14.0, 13.8.

Minor- (Rf = 0.71 in 10:1 pentane/EtOAc), **m.p.**: 161.5.- 162.2°C.

^{1}H-NMR (700 MHz, $CDCl_3$) δ = 7.57 (dt, *J* = 7.6, 0.9 Hz, 1H), 7.34 (td, *J* = 7.5, 1.2 Hz, 1H), 7.30 – 7.27 (m, 2H), 7.24 – 7.20 (m, 3H), 7.18 – 7.15 (m, 1H), 7.10 (ddd, *J* = 7.6, 1.1, 0.6 Hz, 1H), 5.95 (d, *J* = 1.2 Hz, 1H), 5.94 (d, *J* = 1.1 Hz, 1H), 4.39 (p, *J* = 9.1 Hz, 1H), 3.90 – 3.81 (m, 2H), 3.04 – 2.93 (m, 2H), 2.84 – 2.79 (m, 1H), 2.80 – 2.75 (m, 1H), 2.59 (d, *J* = 1.0 Hz, 3H), 2.57 (d, *J* = 1.0 Hz, 3H), 2.56 – 2.50 (m, 1H), 2.46 (dddd, *J* = 12.1, 8.4, 3.8, 0.9 Hz, 1H), 2.21 (s, 3H), 2.16 (s, 3H).

^{13}C{^{1}H}-NMR (176 MHz, $CDCl_3$) δ = 175.7, 175.5, 152.2, 152.1, 149.4, 148.4, 146.6, 144.1, 144.1, 128.5 (2C), 128.2, 128.1, 126.2, 126.1 (2C), 124.9, 124.4, 111.3, 111.2, 57.6, 54.6, 49.1, 42.3, 37.9, 37.1, 32.7, 30.9, 14.6, 14.6, 14.0, 13.9.

IR (Diamond-ATR): $\tilde{\nu}$ (cm^{-1}) = 1715, 1455, 1446, 1410, 1377, 1359, 1321, 1310, 1252, 1024.

HRMS (ESI, Q-TOF): $[M+H]^+$ calcd for $C_{32}H_{33}N_4O_2^+$ = 505.2599; found: 505.2610.

Dimethyl 1',3-diphenyl-[1,1'-bi(cyclobutan)]-2-ene-1,3'-dicarboxylate (5a).

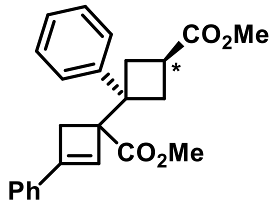

5a

Following the **GP1**, taking $Sc(OTf)_3$ as LA, in MeCN **5a** was obtained as a diastereomeric mixture (dr= 3:1), colorless oil (12.1 mg, 32.0 μmol, 20%). Purified by automated column chromatography (25 g 25SI-HP cartridge, 15 mL/min) using pentane/EtOAc = 100:1 – 10:1 (R_f = 0.61 in 10:1 pentane/EtOAc). It was further purified with normal phase HPLC (Gradient, 95:5 to 92:8 hexane/EtOAc).

Major- isolated by hplc

^{1}H-NMR (700 MHz, $CDCl_3$) δ = 7.43 – 7.39 (m, 2H), 7.38 – 7.33 (m, 6H), 7.33 – 7.28 (m, 1H), 7.28 – 7.23 (m, 1H), 6.39 (s, 1H), 3.67 (s, 3H), 3.46 (s, 3H), 3.06 – 2.98 (m, 2H), 2.96 – 2.84 (m, 3H), 2.75 – 2.64 (m, 2H).

^{13}C{^{1}H}-NMR (176 MHz, $CDCl_3$) δ = 175.7, 173.8, 148.3, 143.7, 133.5, 128.7, 128.6 (2C), 128.3 (2C), 127.2 (2C), 126.8, 126.5, 125.2 (2C), 58.6, 51.8, 51.5, 46.5, 34.8, 33.4, 33.0, 31.8.

Minor- found as mixture with major in 3:1

^{1}H-NMR (700 MHz, $CDCl_3$) δ = 7.43 – 7.40 (m, 2H, combined peak), 7.39 – 7.32 (m, 2H, combined peak), 7.33 – 7.28 (m, 3H, combined peak), 7.23 – 7.17 (m, 1H), 7.13 – 7.09 (m, 2H), 6.51 (s, 1H), 3.60 (s, 3H), 3.47 (s, 3H), 3.25 – 3.18 (m, 1H), 3.13 (d, J = 13.4 Hz, 1H), 3.11 – 3.06 (m, 1H), 2.96 – 2.84 (m, 1H, combined peak), 2.83 – 2.76 (m, 2H), 2.73 – 2.64 (m, 1H, combined peak).

^{13}C{^{1}H}-NMR (176 MHz, $CDCl_3$) δ = δ 175.6, 173.8, 148.5, 146.8, 133.3, 128.9, 128.6 (2C), 128.1 (2C), 126.4, 126.4, 126.2 (2C), 125.1 (2C), 58.0, 51.9, 51.6, 46.1, 34.6, 33.9, 33.6, 33.0.

IR (Diamond-ATR): $\tilde{\nu}$ (cm^{-1}) = 1725, 1254, 1233, 1196, 1171, 1112, 1068, 1043, 1029, 1010.

HRMS (APCI,Q-TOF): $[M+H]^+$ calcd for $C_{24}H_{25}O_4^+$ = 377.1747; found: 377.1747.

Dimethyl 1',3-bis(4-bromophenyl)-[1,1'-bi(cyclobutan)]-2-ene-1,3'-dicarboxylate (5b).

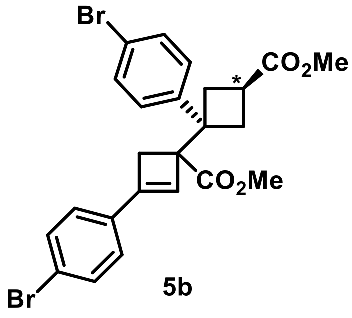

5b

Following the **GP1**, taking $Sc(OTf)_3$ as LA, in CH_2Cl_2 **5b** was obtained as a diastereomeric mixture (dr= 10:1) colorless oil (9.3 mg, 17.4 µmol, 12%) along with **3b**. Purified by automated column chromatography (25 g 25SI-HP cartridge, 15 mL/min) using pentane/EtOAc = 100:1 – 10:1 (Rf = 0.75 in 10:1 pentane/EtOAc).

Major Diastereomer with traces of Minor (dr= 10:1).

^{1}H-NMR (700 MHz, $CDCl_3$) δ = δ 7.52 – 7.44 (m, 4H), 7.29 – 7.23 (m, 2H), 7.22 – 7.18 (m, 2H), 6.37 (s, 1H), 3.67 (s, 3H), 3.48 (s, 3H), 3.01 (d, *J* = 13.3 Hz, 1H), 2.95 (d, *J* = 13.4 Hz, 1H), 2.93 – 2.81 (m, 3H), 2.68 – 2.56 (m, 2H).

^{13}C{^{1}H}-NMR (176 MHz, $CDCl_3$) δ = 175.5, 173.3, 147.4, 142.7, 132.2, 131.8 (2C), 131.5 (2C), 129.1 (2C), 127.1, 126.8 (2C), 122.9, 121.0, 58.3, 51.9, 51.7, 46.2, 34.7, 33.4, 33.1, 31.7.

IR (Diamond-ATR): $\tilde{\nu}$ (cm^{-1}) = 2923, 1729, 1487, 1434, 1258, 1196, 1166, 1075, 1033, 1008.

HRMS (APCI, Q-TOF): $[M+H]^+$ calcd for $C_{24}H_{23}Br^{81}BrO_4^+$ = 534.9938; found: 534.9945.

Dimethyl 1',3-di-m-tolyl-[1,1'-bi(cyclobutan)]-2-ene-1,3'-dicarboxylate (5e).

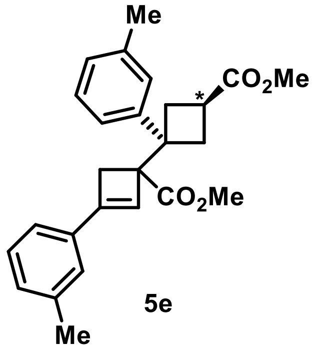

5e

Following the **GP3**, taking $FeCl_3$ as LA, in MeCN **5e** was obtained as a colorless oil (10 mg, 24.7 µmol, 16%). Purified by automated column chromatography (25 g 25SI-HP cartridge, 15 mL/min) using pentane/EtOAc = 100:1 – 10:1 (Rf = 0.6 in 10:1 pentane/EtOAc). It was further purified with normal phase HPLC (Gradient, 95:5 to 92:8 hexane/EtOAc).

5e was also obtained in 7% following **GP1** using $Bi(OTf)_3$ as LA, along with **3e** and **4e**.

Major diastereomer with minor(dr = 5:1) along with traces of BCB-annulated product.

^{1}H-NMR (700 MHz, $CDCl_3$) δ = 7.28 – 7.20 (m, 4H), 7.15 – 7.11 (m, 3H), 7.09 – 7.04 (m, 1H), 6.37 (s, 1H), 3.67 (s, 3H), 3.46 (s, 3H), 3.02 (d, *J* = 13.3 Hz, 1H), 2.97 (d, *J* = 13.3 Hz, 1H), 2.96 – 2.82 (m, 3H, combined peak), 2.74 – 2.60 (m, 2H), 2.38 (s, 3H), 2.36 (s, 1H).

^{13}C{^{1}H}-NMR (176 MHz, $CDCl_3$) δ = 175.8, 173.8, 148.3, 143.6, 138.2, 137.7, 133.5, 129.5, 128.5, 128.1, 128.0, 127.6, 126.4, 125.8, 124.3, 122.3, 58.5, 51.8, 51.4, 46.4, 34.8, 33.4, 33.0, 31.8, 21.8, 21.5.

IR (Diamond-ATR): $\tilde{\nu}$ (cm^{-1}) = 2950, 1726, 1433, 1361, 1255, 1235, 1195, 1171, 1113, 1075.

HRMS (APCI, Q-TOF): $[M+H]^+$ calcd for $C_{26}H_{29}O_4^+$ = 405.2060; found: 405.2067.

Dimethyl 1',3-bis(4-(trifluoromethyl)phenyl)-[1,1'-bi(cyclobutan)]-2-ene-1,3'-dicarboxylate (5d).

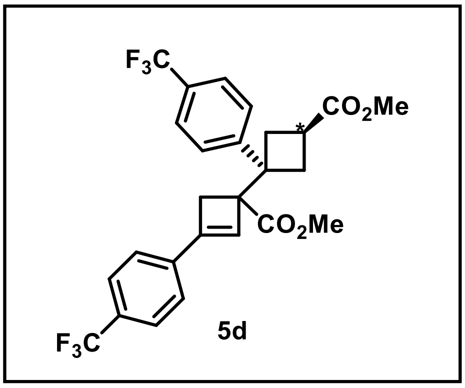

Following the **GP3** taking $FeCl_3$ as LA, in CH_2Cl_2 **5d** was obtained as a diastereomeric mixture (dr = 3:2), colorless oil (10 mg, 19.5 μmol, 14%) along with **3d**. Purified by automated column chromatography (25 g 25SI-HP cartridge, 15 mL/min) using Pentane/EtOAc = 100:1 – 5:1 (Rf = 0.52 in 10:1 pentane/EtOAc). The diastereomers were separated normal phase HPLC (Gradient, 92:8 to 80:20 hexane/EtOAc).

Major

^{1}H-NMR (700 MHz, $CDCl_3$) δ = 7.66 – 7.60 (m, 4H), 7.53 – 7.49 (m, 2H), 7.48 – 7.43 (m, 2H), 6.50 (s, 1H), 3.68 (s, 3H), 3.48 (s, 3H), 3.10 – 3.03 (m, 2H), 2.95 – 2.88 (m, 3H), 2.76 – 2.63 (m, 2H).

^{13}C{^{1}H}-NMR (176 MHz, $CDCl_3$) δ = 175.3, 173.0, 147.8, 147.3, 136.4, 130.6 (q, *J* = 32.5 Hz), 129.3 (q, *J* = 32.6 Hz), 129.0, 127.7 (2C), 125.7 (q, *J* = 3.8 Hz, 2C), 125.4 (2C), 125.4 (q, *J* = 3.8 Hz, 2C), 124.2 (q, *J* = 272.0 Hz), 124.2 (q, *J* = 272.0 Hz), 58.5, 52.0, 51.8, 46.6, 34.9, 33.5, 33.2, 31.7.

^{19}F NMR (659 MHz, $CDCl_3$) δ = -62.5, -62.7.

Minor

^{1}H-NMR (700 MHz, $CDCl_3$) δ = 7.66 – 7.61 (m, 2H), 7.61 – 7.52 (m, 2H), 7.53 – 7.48 (m, 2H), 7.25 – 7.21 (m, 2H), 6.61 (s, 1H), 3.61 (s, 3H), 3.49 (s, 3H), 3.20 (dddd, *J* = 12.6, 9.6, 3.0, 0.9 Hz, 1H), 3.16 (d, *J* = 13.4 Hz, 1H), 3.13 – 3.04 (m, 1H), 2.92 (d, *J* = 13.3 Hz, 1H), 2.82 (dddd, *J* = 12.5, 9.5, 3.1, 1.0 Hz, 1H), 2.78 (ddd, *J* = 12.6, 8.6, 0.9 Hz, 1H), 2.72 (ddd, *J* = 12.4, 8.6, 0.8 Hz, 1H).

$^{13}C\{^1H\}$-NMR (176 MHz, $CDCl_3$) δ = 175.2, 173.0, 150.5, 147.5, 136.2, 130.8 (q, J = 32.5 Hz), 128.9, 128.9 (q, J = 32.5 Hz), 126.7 (2C), 125.8 (q, *J* = 3.8 Hz, 2C), 125.4(2C), 125.2 (q, *J* = 3.8 Hz, 2C), 124.3 (q, *J* = 271.8 Hz), 124.1 (q, *J* = 272.0 Hz), 57.9, 52.0, 51.8, 46.1, 34.7, 33.8, 33.5, 33.0.

^{19}F NMR (659 MHz, $CDCl_3$) δ = -62.4, -62.7.

IR (Diamond-ATR): $\tilde{\nu}$ (cm^{-1}) = 1728, 1436, 1322, 1255, 1237, 1197, 1164, 1116, 1064, 1014.

HRMS (APCI, Orbitrap): $[M+H]^+$ calcd for $C_{26}H_{23}O_4F_6^+$ = 513.1495; found: 513.1490.

Dimethyl 1'-phenyl-1',2'-dihydro-3'H-spiro[cyclobutane-1,4'-[1,3]methanonaphthalene]-3,3'-dicarboxylate (6a).

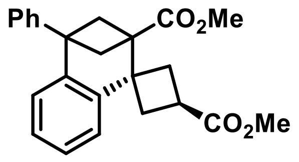

6a

To an oven-dried microwave vial equipped with a magnetic stir bar and a rubber septum (vacuum and argon cycled) was added bicyclobutanes (100 μmol, 1.00 equiv.) and vacuum and argon cycled again. The mixture was then dissolved in 1 mL of solvent at room temperature under argon and stirred for 5 minutes. $FeCl_3$ (40.5 mg, 250 μmol, 2.5 equiv.) is then added to the reaction mixture. The vial is then capped and stirred for 24 hours at the same temperature. The solvent was evaporated and the crude residue was pre-adsorbed on Celite and subjected to automated column chromatography.

6a was obtained as colorless solid (10.2 mg, 27.1 μmol, 27%, dr 4.5:1) along with **4a.** Major diastereomer was purified by automated column chromatography (25 g 30SI-HP cartridge, 15 mL/min) using 10:1 pentane/EtOAc = 100:1 – 10:1 (R_f = 0.63 in 10:1 pentane/EtOAc). It was further purified with normal phase HPLC (Gradient, 95:5 to 92:8 hexane/EtOAc).

The same result was also obtained when the BCB was refluxed with 1.1 equiv. of $FeCl_3$.

^{1}H-NMR (700 MHz, $CDCl_3$) δ = 7.66 (ddd, *J* = 7.8, 1.3, 0.4 Hz, 1H), 7.41 – 7.35 (m, 2H), 7.32 – 7.27 (m, 2H), 7.14 – 7.10 (m, 2H), 6.97 (td, *J* = 7.5, 1.2 Hz, 1H), 6.29 (ddd, *J* = 7.7, 1.4, 0.4 Hz, 1H), 3.81 (s, 3H), 3.72 (s, 3H), 3.39 (tt, *J* = 10.6, 7.7 Hz, 1H), 3.13 – 3.07 (m, 2H), 2.66 – 2.58 (m, 4H), 2.29 (dd, *J* = 6.9, 2.7 Hz, 2H).

^{13}C{^{1}H}-NMR (176 MHz, $CDCl_3$) δ = 175.8, 175.1, 148.4, 144.3, 143.4, 128.6 (2C), 127.5 (2C), 126.8, 126.7, 125.9, 125.7, 123.3, 52.1, 52.0, 48.9, 45.3, 45.0, 41.5 (2C), 35.6 (2C), 30.5.

IR (Diamond-ATR): $\tilde{\nu}$ (cm^{-1}) = 1728, 1434, 1359, 1249, 1197, 1168, 1102, 1090, 1049, 1028.

HRMS (APCI, Q-TOF): $[M+H]^+$ calcd for $C_{24}H_{25}O_4^+$ = 377.1747; found: 377.1749.

m.p.: 145.8- 146.2°C

Trimethyl 1',1'',3-tris(4-bromophenyl)-[1,1':3',1''-tercyclobutan]-2-ene-1,3',3''-tricarboxylate (7).

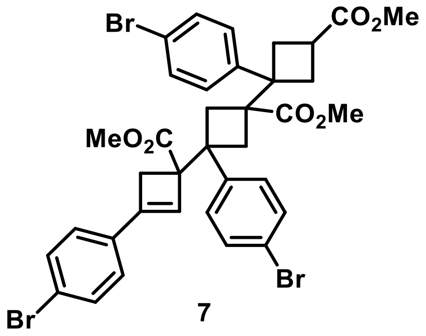

7

Following the **GP1**, taking $Sc(OTf)_3$ as LA in in CH_2Cl_2 (1 mmol scale) **7** was able to be isolated as a colorless oil (4.5 mg, 5.6 μmol, 2%) along with **3b**, **3b´** and **5b** (even though traces were observed in 0.3 mmol reaction, isolable amounts were formed in 1 mmol scale reaction). Purified by automated column chromatography (25 g 30SI-HP cartridge, 15 mL/min) using 10:1 pentane/EtOAc = 100:1 – 10:1 (Rf = 0.28 in 10:1 pentane/EtOAc).). It was further purified with normal phase HPLC (Gradient, 95:5 to 80:20 hexane/EtOAc).

^{1}H-NMR (700 MHz, $CDCl_3$) δ = 7.49 – 7.43 (m, 4H), 7.31 (d, J = 8.5 Hz, 2H), 7.15 (d, *J* = 8.4 Hz, 2H), 7.05 (d, *J* = 8.5 Hz, 2H), 6.83 (d, *J* = 8.5 Hz, 2H), 6.19 (s, 1H), 3.51 (s, 3H), 3.49 (s, 3H), 3.48 (s, 3H), 3.33 – 3.28 (m, 1H), 3.01 (d, *J* = 13.3 Hz, 1H), 2.94 (dt, *J* = 13.5, 1.4 Hz, 1H), 2.85 (tt, *J* = 10.1, 7.3 Hz, 1H), 2.76 (d, *J* = 14.0 Hz, 1H), 2.66 (d, *J* = 13.5 Hz, 1H), 2.64 – 2.58 (m, 3H), 2.57 – 2.50 (m, 2H).

^{13}C{^{1}H}-NMR (176 MHz, $CDCl_3$) δ = 175.8, 175.3, 173.1, 147.9, 145.0, 144.3, 132.0, 131.9 (2C), 131.7 (2C), 131.0 (2C), 128.3 (2C), 128.1 (2C), 127.1, 126.6 (2C), 123.1, 120.9, 120.7, 58.0, 52.0, 51.8, 51.8, 49.1, 46.7, 42.1, 36.9, 35.9, 34.1, 33.4, 33.4, 32.3.

IR (Diamond-ATR): $\tilde{\nu}$ (cm^{-1}) = 1723, 1434, 1396, 1298, 1248, 1214, 1148, 1118, 1074, 1009.

HRMS (ESI, Orbitrap): $[M+Na]^+$ calcd for $C_{36}H_{33}Br_2{}^{81}BrO_6Na^+$ = 822.9699, found: 822.9690; calcd for $C_{36}H_{33}Br^{81}Br_2O_6Na^+$ = 824.9679; found: 824.9672.

Methyl 3-(5-methoxy-2-(3-(methoxycarbonyl)-1-(3-methoxyphenyl)cyclobutyl)phenyl)cyclobut-2-ene-1-carboxylate (8).

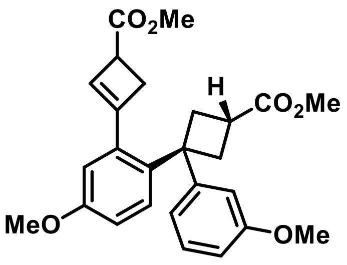

8

Following the **GP1**, taking $Sc(OTf)_3$ as LA, in CH_2Cl_2 **8** was obtained as a colorless oil (3.9 mg, 8.9 µmol, 6%) along with **2c** (10 mg, 45.8 µmol, 15%). Purified by automated column chromatography (25 g 30SI-HP cartridge, 15 mL/min) using 10:1 Pentane/EtOAc = 100:1 – 10:1 (Rf = 0.23 in 10:1 pentane/EtOAc). It was further purified with normal phase HPLC (Gradient, 95:5 to 85:15 hexane/EtOAc. Still contaminated with traces of the minor diastereomer).

^{1}H-NMR (700 MHz, $CDCl_3$) δ = 7.27 (d, *J* = 8.6 Hz, 1H), 7.20 (t, *J* = 8.0 Hz, 1H), 6.90 – 6.84 (m, 2H), 6.78 (t, *J* = 2.0 Hz, 1H), 6.71 (ddd, *J* = 8.2, 2.5, 0.8 Hz, 1H), 6.69 (d, *J* = 2.8 Hz, 1H), 5.43 (d, *J* = 1.2 Hz, 1H), 3.82 (s, 3H), 3.74 (s, 3H), 3.68 (s, 3H), 3.65 (s, 3H), 3.43 – 3.35 (m, 2H), 3.06 – 2.83 (m, 4H), 2.80 (dd, *J* = 13.0, 4.7 Hz, 1H), 2.76 (dd, *J* = 12.9, 2.0 Hz, 1H).

^{13}C{^{1}H}-NMR (176 MHz, $CDCl_3$) δ = 175.6, 173.9, 159.9, 157.9, 150.1, 148.0, 138.8, 134.8, 130.4, 129.6, 129.4, 118.5, 114.3, 113.1, 113.0, 110.5, 55.5, 55.3, 51.9, 51.8, 46.7, 41.6, 39.5, 39.2, 35.8, 33.1.

IR (Diamond-ATR): $\tilde{\nu}$ (cm^{-1}) = 1728, 1600, 1577, 1484, 1463, 1433, 1282, 1200, 1169, 1040.

HRMS (ESI, Orbitrap): $[M+Na]^+$ calcd for $C_{26}H_{28}O_6Na^+$ = 459.1778; found: 459.1784.

6. Mechanistic experiments and additional investigations

Conversion CB addition-elimination product to CB annulation product.

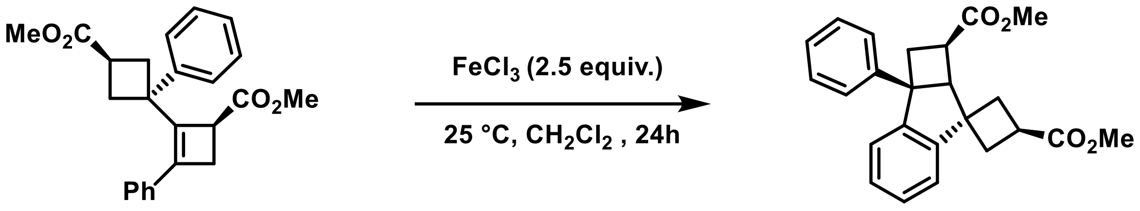

3a **4a, 78%**

To an oven dried microwave vial, add **3a** (23 mg, 60 µmol), dissolve it in 1.8 mL of CH_2Cl_2 add $FeCl_3$ (24.7 mg, 150 µmol, 2.5 equiv.), vacuum and argon cycled, add solvent, cap it and stir it on a magnetic stirrer at rt. 700 rpm. After the completion of the reaction mixture was adsorbed on celite and performed automated CC in 25 g Buchi catridge. 0 to 10% EtOAc: Pentane afforded the product **4a** (18 mg, 480 µmol, 78%).

Conversion BCB addition-elimination product to BCB annulation product.

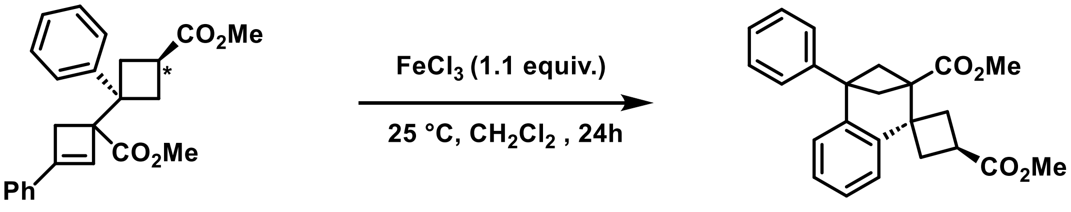

5a, dr = 3:1 **6a, 33%**
(58% 5a)

To an oven dried microwave vial, add **5a** (26 mg, 70 µmol), dissolve it in 2 mL of CH_2Cl_2 $FeCl_3$ (12.3 mg, 80 µmol, 1.1 equiv.), cap it and stir it on a magnetic stirrer at rt. 700 rpm. After the completion of the reaction mixture was adsorbed on celite and performed automated CC in 25 g Buchi catridge. 0 to 10% EtOAc: Pentane afforded the product **6a** (8.6 mg, 23 µmol, 33% (79% brsm)).

Investigating potential involvement of a hidden Bronsted acid generated in-situ from $FeCl_3$

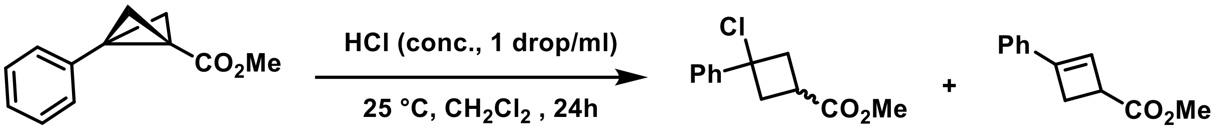

1a

1a+HCl, 48% (dr 2.4:1)

2a, 43%

Following the **GP2**, with HCl (37%) instead of H_2SO_4. The NMR yields are provided, CH_2Br_2 (0.2 mmol) was used as an internal standard. The spectral data for **1a+HCl** matches with the one reported in literature and with the data for the precursor for BCB **1a**.

Comment: To assess the possibility of hidden Brønsted acid involvement, a control experiment was performed using HCl in the absence of $FeCl_3$. Under these conditions, no dimer formation was detected. These results support the conclusion that the dimerisation observed in the presence of $FeCl_3$ is associated with Lewis acid-mediated activation of the BCB substrate rather than arising from Brønsted acid.

Experiment with deuterated H_2SO_4.

Dimethyl 2a'-phenyl-1',2',2a',7a'-tetrahydrospiro[cyclobutane-1,7'-cyclobuta[*a*]indene]-1',3-dicarboxylate-1',3-*d_2* (4aD).

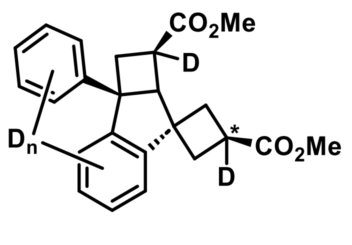

4aD

Following the **GP2**, **4aD** is obtained as a mixture of diastereomers (dr = 3:1) as a colorless solid (33,5 mg, 117.0 µmol, 76%). The reaction mixture was purified by flash column chromatography using 15:1 Pentane/EtOAc.

Comment: NMR studies showed that **4aD** contains 2 deuteriums in aliphatic region (35-40% saturation), HRMS studies also revealed that 2H-containing molecules

between 2 and 7 deuteriums, we assign additional deuteriums to aromatic substitution of protons, additional studies on the position of the deuteriums in the aromatic region have not been conducted. The analytical data of **4aD** matches the data of **4a** with the following changes, caused by deuterium incorporation.

^{2}H-NMR (107 MHz, $CDCl_3$) δ = 3.37, 2.63.

^{13}C{^{1}H}-NMR (176 MHz, $CDCl_3$) δ = 35.9 (t, *J* = 22.2 Hz), 31.9 (t, *J* = 20.9 Hz).

HRMS (ESI,Q-TOF): 2 of the highest intencity $[M+H]^+$ calcd for $C_{24}H_{20}D_4O_4^+$ = 381.1998; found: 381.1993 and $[M+H]^+$ calcd for $C_{24}H_{19}D_5O_4^+$ = 382.2061; found: 382.2054.

Attempts of heterodimerisations.

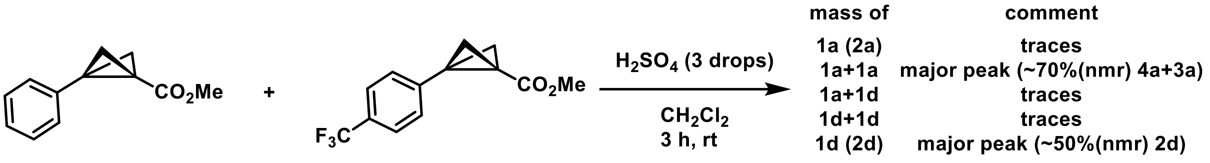

1a **1d**

Comment: Under the standard Brønsted acidic conditions, no convincing evidence for heterodimer formation between **1a** and **1d** was obtained. The major signal corresponded to the homodimer derived from **1a**, while the heterodimeric species was detected only in trace amounts.

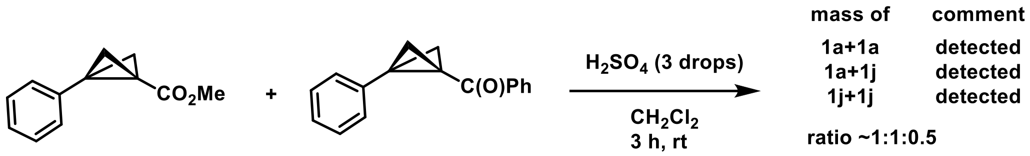

1a **1j**

Comment: Under the same conditions **1a** and **1j**, both homodimeric and heterodimeric species were detected. However, the observed ca. **1:1:0.5** ratio indicates low selectivity and no clear preference for heterodimer formation.

Competition Experiments.

To an oven-dried microwave vial equipped with a magnetic stir bar and a rubber septum (vacuum and argon cycled) was added bicyclobutanes **1a** (100 μmol, 1.00 equiv.), corresponding annulation partner **11/13/15** (100 μmol, 1.00 equiv.), and Bi(OTf)$_3$ (20 μmol, 20 mol%) under argon. The mixture was then dissolved in 1 mL of CH_2Cl_2. The vial is then capped and stirred for 24 hours at the same temperature. The crude residue was then filtered and submiited for NMR with CH_2Br_2 (100 μmol) as an internal standard (the yields mentioned in the scheme is crude NMR yields).

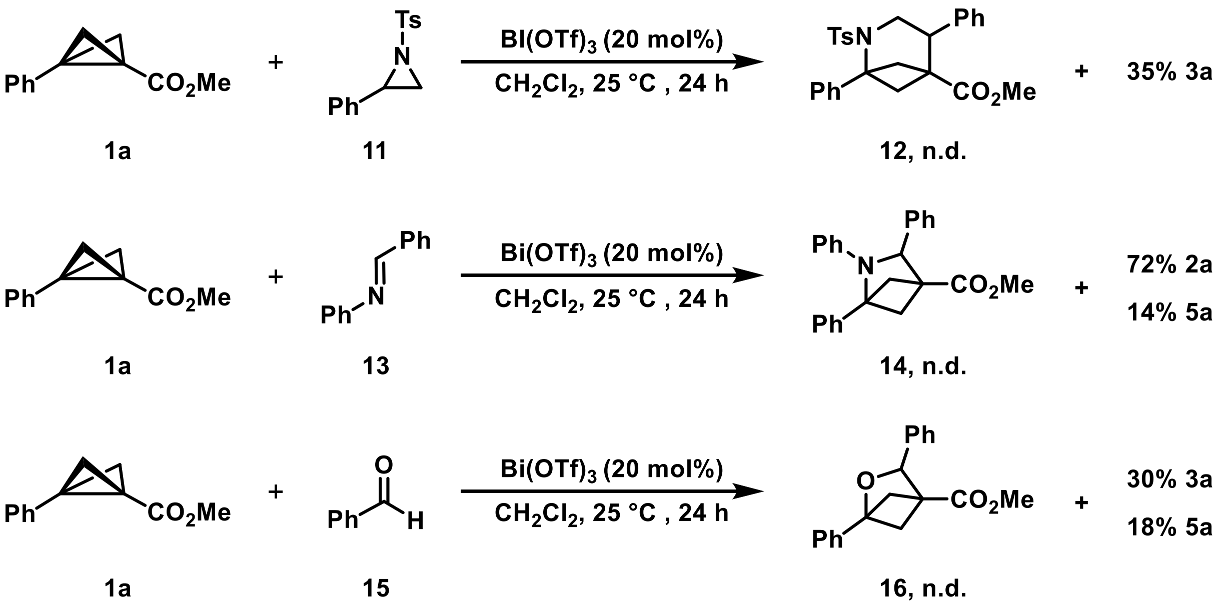

Comment: In all three cases, no products resulting from intermolecular annulation of BCB **1a** with reaction partners **11**, **13**, or **15** were detected. Instead, only the known dimerisation products of **1a** were observed, demonstrating that BCB self-dimerisation is a major competing background pathway under the Bi(OTf)$_3$-catalysed conditions.

7. Follow up

Hydrolysis of dimethyl 1',4-diphenyl-[1,1'-bi(cyclobutan)]-4-ene-2,3'-dicarboxylate to 1',4-diphenyl-[1,1'-bi(cyclobutan)]-4-ene-2,3'-dicarboxylic acid (9).

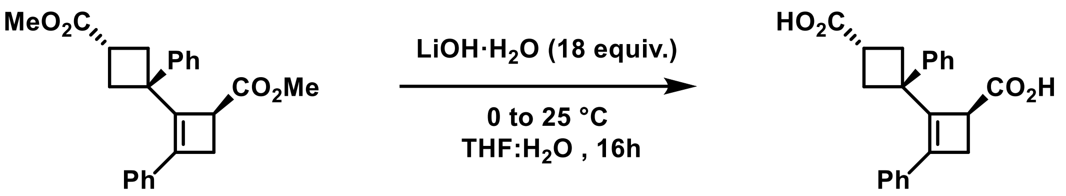

3a **9**

Dimer **3a** (16.7 mg, 44 μmol) was dissolved in THF (0.25 mL) and the solution cooled to 0 °C. A solution of LiOH·H_2O (33.5 mg, 800 μmol, 18 equiv.) in H_2O (0.75mL) was added dropwise to the solution. After 20 min of stirring at 0 °C, the cooling bath was removed and the mixture stirred over night at room temperature (25 °C). The mixture was diluted with NH_4Cl (sat., aq., 4 mL), HCl (1 M, aq, 20 mL) and the aqueous phase extracted with EtOAc (3×8 mL). The organic phase was washed with H_2O (8 mL), brine (80 mL), dried over Na_2SO_4 and concentrated in vacuo. It´s then recrystallised from EtOAc/ Hexane afforded **9** (16 mg, 44 μmol, 100%) as a white solid.[10]

^{1}H-NMR (700 MHz, $CDCl_3$) δ = 7.51 – 7.45 (m, 2H), 7.34 – 7.28 (m, 2H), 7.22 – 7.12 (m, 4H), 7.01 – 6.96 (m, 2H), 3.73 (dd, *J* = 5.0, 1.8 Hz, 1H), 3.28 (p, *J* = 9.2 Hz, 1H), 3.15 – 3.01 (m, 3H), 2.93 (dd, *J* = 12.5, 5.0 Hz, 1H), 2.81 (dd, *J* = 12.5, 1.9 Hz, 1H), 2.53 (ddd, *J* = 12.4, 9.4, 3.4 Hz, 1H).

^{13}C{^{1}H}-NMR (176 MHz, $CDCl_3$) δ = 181.7, 181.5, 145.1, 143.9, 140.0, 133.8, 128.9 (2C), 128.1 (2C), 127.8, 126.8 (2C), 126.6, 126.1 (2C), 43.9, 43.1, 37.2, 35.6, 32.2, 29.7.

IR (Diamond-ATR): $\tilde{\nu}$ (cm^{-1}) = 2933, 1696, 1493, 1445, 1417, 1301, 1231, 1183, 1129, 1027.

HRMS (ESI, Orbitrap): [M–H]$^-$ calcd for $C_{22}H_{19}O_4^-$ = 347.1289; found: 347.1283.

m.p.: 152.7- 153.5°C

Hydrolysis of dimethyl (-1',3-bis(4-bromophenyl)-[1,1'-bi(cyclobutan)]-2-ene-1,3'-dicarboxylate to (1,3'-bis(4-bromophenyl)-1'-(methoxycarbonyl)-[1,1'-bi(cyclobutan)]-2'-ene-3-carboxylic acid (10).

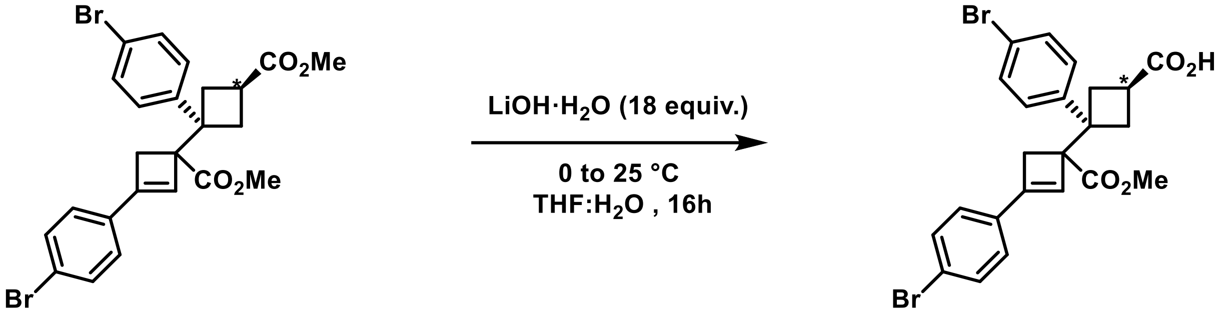

Dimer **5a** (30mg, 56 µmol) was dissolved in THF (0.3 mL) and the solution cooled to 0 °C. A solution of LiOH·H_2O (42.4mg, 1.1 mmol, 18 equiv.) in H_2O (1.0 mL) was added dropwise to the solution. After 20 min of stirring at 0 °C, the cooling bath was removed and the mixture stirred over night at room temperature (25 °C). The mixture was diluted with NH_4Cl (sat., aq., 5 mL), HCl (1 M, aq., 20mL) and the aqueous phase extracted with EtOAc (3×8 mL). The organic phase was washed with H_2O (8 mL), brine (80 mL), dried over Na_2SO_4 and concentrated in vacuo. It´s then recrystallised from EtOAc/hexane afforded **10** (20 mg, 56 µmol, 100%) as a pale yellow solid (dr= 4:1).[10]

Major diastereomer

^{1}H-NMR (700 MHz, $CDCl_3$) δ = 7.49 – 7.47 (m, 4H), 7.28 – 7.23 (m, 2H, combined peak), 7.23 – 7.18 (m, 2H), 6.37 (s, 1H), 3.49 (s, 3H), 3.02 (d, *J* = 13.3 Hz, 1H), 2.97 – 2.83 (m, 4H, combined peak), 2.71 – 2.60 (m, 2H).

^{13}C{^{1}H}-NMR (176 MHz, $CDCl_3$) δ = 180.1, 173.3, 147.5, 142.4, 132.1, 131.8 (2C), 131.5 (2C), 129.0 (2C), 127.0, 126.8 (2C), 123.0, 121.0, 58.3, 51.7, 46.2, 34.7, 33.3, 33.0, 31.6.

Minor diastereomer

^{1}H-NMR (700 MHz, $CDCl_3$) δ = 7.52 – 7.49 (m, 2H), 7.44 – 7.40 (m, 2H), 7.28 – 7.23 (m, 2H, combined peak), 7.01 – 6.96 (m, 2H), 6.48 (s, 1H), 3.50 (s, 3H), 3.22 – 3.15 (m, 1H), 3.13 – 3.08 (m, 2H), 2.97 – 2.84 (m, 1H, combined peak), 2.84 – 2.78 (m, 2H), 2.74 (dd, J = 12.5, 8.2 Hz, 1H).

$^{13}C\{^{1}H\}$-NMR (176 MHz, $CDCl_3$) δ = 180.2, 173.3, 147.8, 145.4, 131.9, 131.9 (2C), 131.3 (2C), 128.0 (2C), 126.8, 126.7 (2C), 123.1, 120.6, 57.7, 51.8, 45.7, 34.6, 33.7, 33.2, 32.8.

IR (Diamond-ATR): $\tilde{\nu}$ (cm^{-1}) = 1731, 1707, 1484, 1434, 1305, 1288, 1233, 1167, 1123, 1032.

HRMS (ESI, Orbitrap): $[M–H]^{–}$ calcd for $C_{23}H_{19}O_4Br^{81}Br^{–}$ = 518.9635; found: 518.9624.

m.p.: 193.7- 194.3°C

8. DFT Calculations

Method

All DFT calculations were performed using ORCA 6.1[11].Geometry optimizations were carried out using the r^{2}SCAN-3c composite method.[12] Solvation effects were treated with CPCM (dichloromethane).[13;14] All structures were confirmed as minima or transition states through vibrational frequency analysis (no imaginary frequencies for minima, one imaginary frequency for transition states).

Final electronic energies were obtained from single-point calculations at the M06-2X-D3ZERO/def2-TZVP level using the r^{2}SCAN-3c optimized geometries.[15] Solvation was consistently treated using CPCM(DCM).[13;14] Gibbs free energies were computed at 298.15 K and 1 atm by combining M06-2X/def2-TZVP electronic energies with thermal corrections (including ZPE, enthalpy, and entropy) obtained from r^{2}SCAN-3c frequency calculations within the Quasi-RRHO approximation.[16]

Results

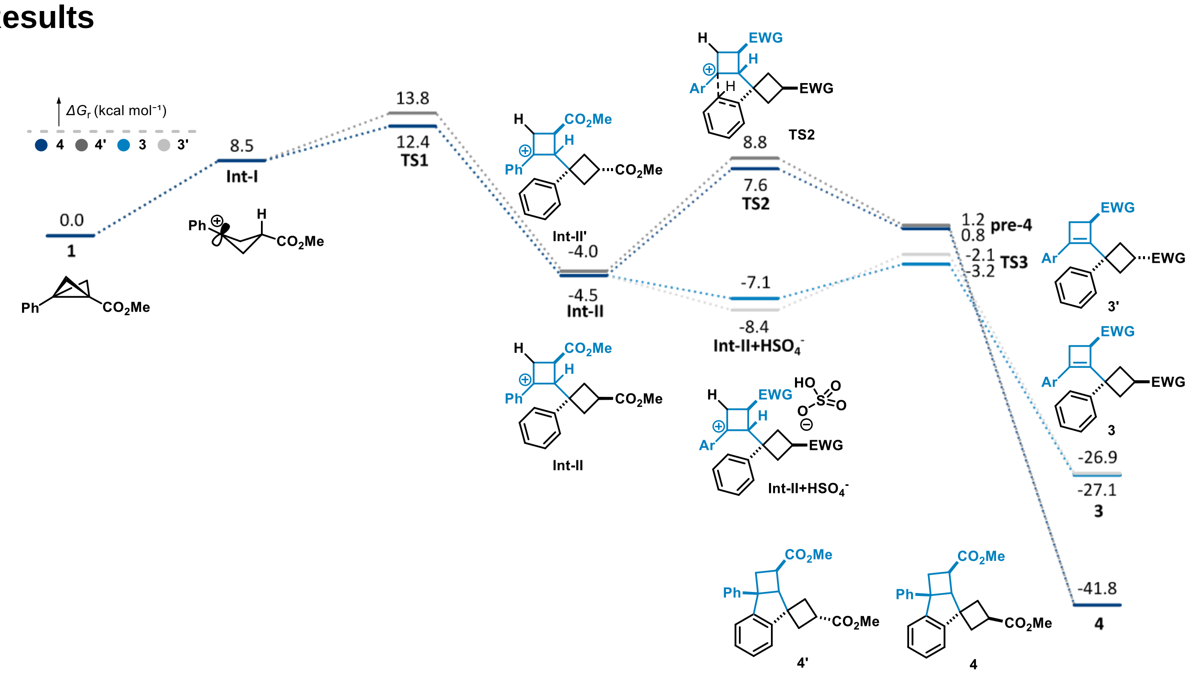

Figure 1. Free energy profile of a Bronsted acid assisted dimerization of BCB 1, followed by either an annulation (blue and grey pathway) or an elimination (light blue or light grey pathway); M06-2X/def2-TZVP-D3//r^{2}scan-3c, CPCM(DCM).

In the first step sulphuric acid protonates the **BCB 1** to obtain species **Int-I**. The carbocation is then attacked by the nucleophilic double bond of **2** to obtain **Int-II**. Notably, **2** can attack **Int-I** from two sides. Due a slide envelope (in the scheme it is strongly exaggerated) of **Int-I** there is a sterically more hindered concave side and a sterically more available convex side. For the attack from the convex side a barrier of

3.9 kcal/mol was calculated. With an attack from the concave side the activation barrier slightly increases to 5.3 kcal/mol and **Int-II** is by 0.5 kcal/mol more favored than **Int-II'**. Attack of the resulting carbocation by the phenyl ring (ΔG‡ = 12.1 kcal/mol and 12.9 kcal/mol, respectively) leads to **Pre-4** and **Pre-4'**, respectively. Deprotonation leads to **4** and **4'**, respectively.

<table>
<tr><th>Species</th><th>E(M062X-D3zero) [E_h]</th><th>G^{RRHO}_{298} [E_h]</th><th>ΔG [kcal/mol]</th><th>ΔΔG [kcal/mol]</th></tr>
<tr><td>BCB 1</td><td>-614.8757424</td><td>0.1714684</td><td rowspan="3">0.0</td><td rowspan="2"></td></tr>
<tr><td>2</td><td>-614.8977761</td><td>0.1705764</td></tr>
<tr><td>H_2SO_4</td><td>-700.3114169</td><td>0.0091993</td><td rowspan="2">8.5</td></tr>
<tr><td>Int-I</td><td>-615.2931159</td><td>0.1818685</td><td rowspan="2">8.5</td></tr>
<tr><td>HSO_4^+</td><td>-699.8795892</td><td>-0.0021736</td><td rowspan="2">3.9</td></tr>
<tr><td>TS1</td><td>-1230.2103373</td><td>0.3782138</td><td>12.4</td></tr>
<tr><td>Int-II</td><td>-1230.2417939</td><td>0.3826802</td><td>-4.5</td><td rowspan="2">12.1</td></tr>
<tr><td>TS2</td><td>-1230.2235828</td><td>0.3838048</td><td>7.6</td></tr>
<tr><td>Pre-4</td><td>-1230.2345674</td><td>0.3838573</td><td>0.8</td><td rowspan="2"></td></tr>
<tr><td>4</td><td>-1229.8718798</td><td>0.3738272</td><td>-41.8</td></tr>
<tr><td>TS1'</td><td>-1230.2091814</td><td>0.3792317</td><td>13.8</td><td>5.3</td></tr>
<tr><td>Int-II'</td><td>-1230.2405180</td><td>0.3821869</td><td>-4.0</td><td rowspan="2">12.8</td></tr>
<tr><td>TS2'</td><td>-1230.2225686</td><td>0.3847298</td><td>8.8</td></tr>
<tr><td>Pre-4'</td><td>-1230.2337201</td><td>0.3837015</td><td>1.2</td><td rowspan="2"></td></tr>
<tr><td>4'</td><td>-1229.8715483</td><td>0.3735200</td><td>-41.8</td></tr>
</table>

Beyond the intramolecular Friedel–Crafts alkylation, β-elimination to form an alkene represents a viable competing pathway, which was observed experimentally for substrates bearing electron-deficient arene moieties. To assess the feasibility of this side reaction, we investigated the elimination mechanism with HSO_4^- as the base. Initially, the hydrogensulfate anion forms a hydrogen-bonded adduct with **Int-II**. Subsequent deprotonation of the β-carbon proceeds via **TS3** with an activation barrier of only 4.4 kcal/mol, substantially lower than that of the cyclization transition state **TS2** (ΔG‡ = 12.1 kcal/mol). Despite this significant kinetic advantage (ΔΔG‡ = 7.7 kcal/mol), elimination remains disfavored under the reaction conditions. While HSO_4^- is generated in situ upon protonation of the substrate by H_2SO_4, it is consumed upon rearomatization, resulting in only catalytic concentrations of the base. This low steady-

state concentration of HSO_4^- renders the bimolecular elimination kinetically uncompetitive despite its lower intrinsic barrier. Furthermore, the annulation product is thermodynamically favored by 13.9 kcal/mol (ΔG_R = −41.0 kcal/mol vs. −27.1 kcal/mol for the alkene), providing an additional driving force for the cyclization pathway under the equilibrating acidic conditions.

Comparison Annulation vs. Elimination

Species	E(M062X-D3zero) [E_h]	G^{RRHO}_{298} [E_h]	ΔG [kcal/mol	ΔΔG [kcal/mol]
**Int-II+HSO_4^-**	-1930.1494286	0.4044309	-7.1	4.4
**TS3**	-1930.1386693	0.4007553	-2.7	
**3’+H_2SO_4**	-1930.1812100	0.4043097	-27.1	
**Int-II’+HSO_4^-**	-1930.1507594	0.4037135	-8.4	6.3
**TS3’**	-1930.1356237	0.3985906	-2.1	
**3’+H_2SO_4**	-1930.1802033	0.4036551	-26.9	

DFT optimized (r^2scan-3c) geometries

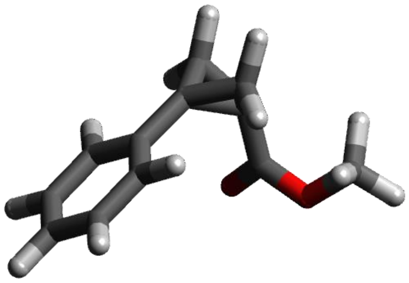

BCB 1

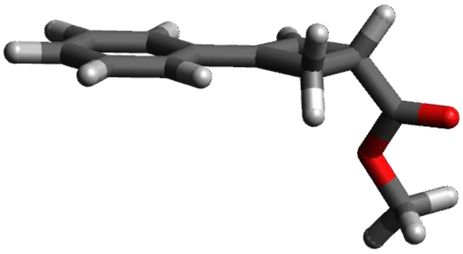

2

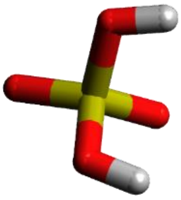

H_2SO_4

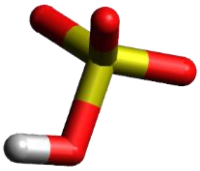

HSO_4^-

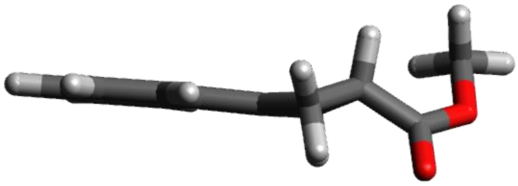

Int-I

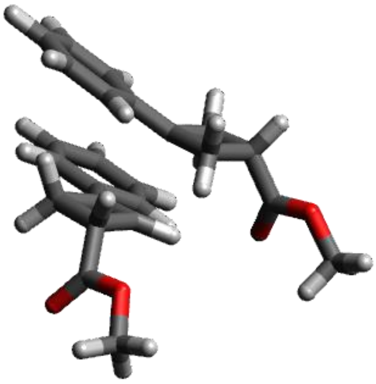

TS1

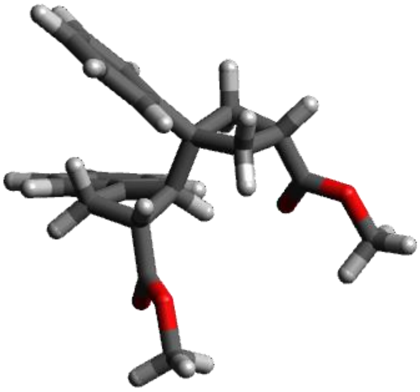

Int-II

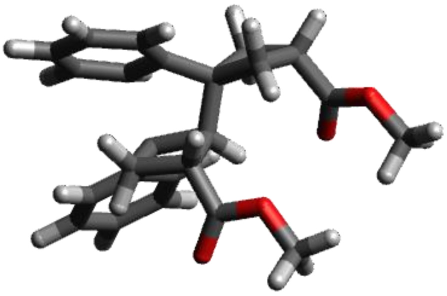

TS2

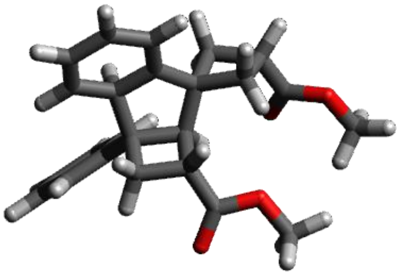

Pre-4

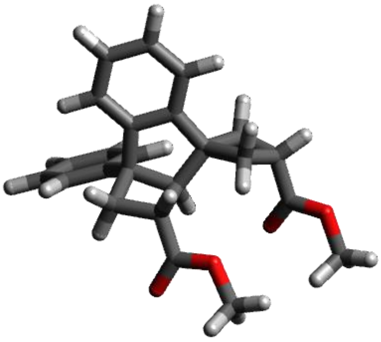

4

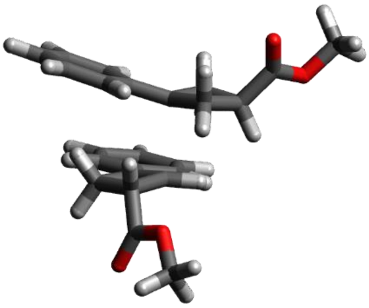

TS1’

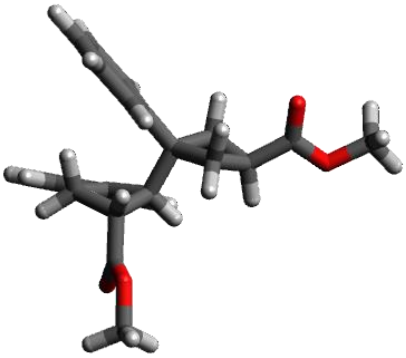

Int-II’

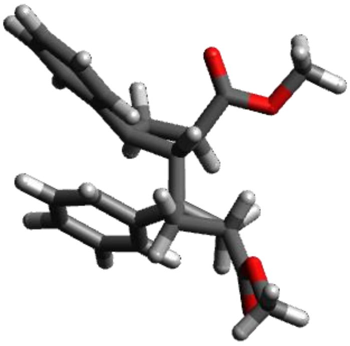

TS2’

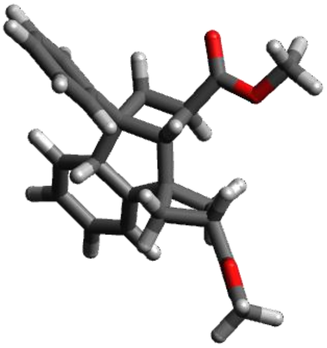

Pre-4’

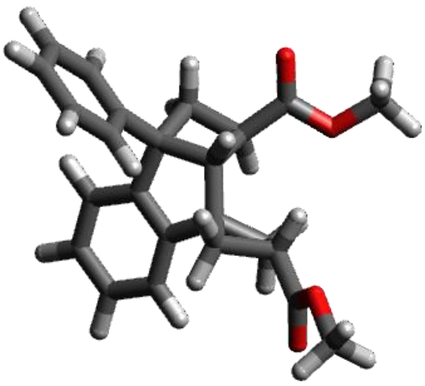

4’

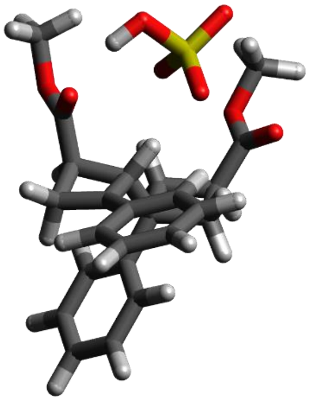

Int-II+HSO$_4^-$

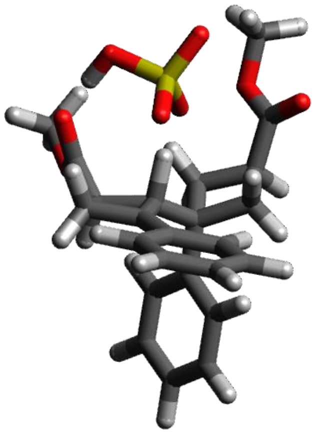

TS3

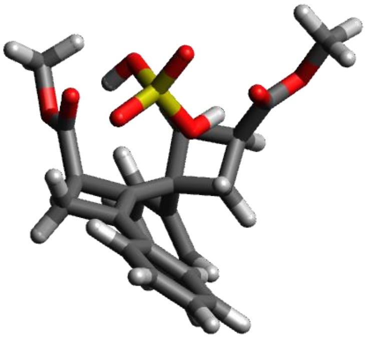

3+H_2SO_4

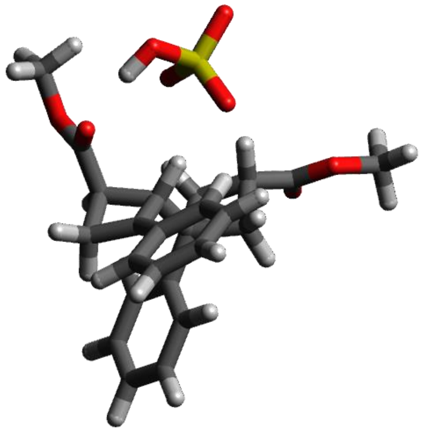

Int-II’

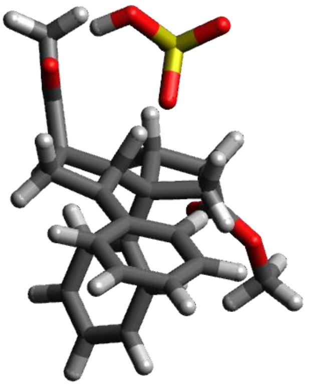

TS3’

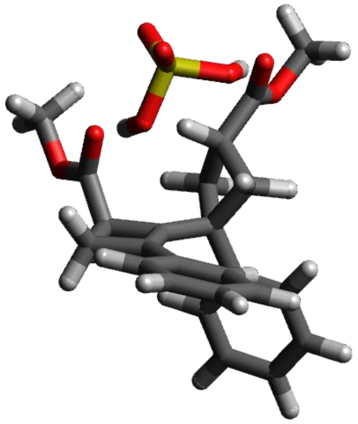

3’+H_2SO_4

DFT optimized (r^2scan-3c) cartesian coordinates

1
E(r^2scan-3c) = -614.763868 (conv)
Lowest Freq. = 21.38 cm⁻¹

C	-2.988311	-0.739018	0.335329
C	-3.005699	-2.058269	-0.113179
C	-1.956196	-2.528745	-0.899576
C	-0.895087	-1.693423	-1.230534
C	-0.859802	-0.369993	-0.767408
C	-1.929647	0.100796	0.008420
H	-3.808085	-0.357819	0.937949
H	-3.835896	-2.711490	0.139643
H	-1.965002	-3.552187	-1.264090
H	-0.091634	-2.071017	-1.855108
H	-1.937929	1.130948	0.351685
C	0.268571	0.504843	-1.089055
C	0.639539	1.820191	-0.479188
C	1.583694	0.667309	-0.292425
C	1.573027	0.166133	-1.713687
H	0.059263	2.142646	0.380591
H	0.959083	2.625506	-1.143440
H	1.802351	-0.885695	-1.861506
H	1.977808	0.845777	-2.465102
C	1.859515	-0.187931	0.860217
O	2.003363	-1.395954	0.761818
O	1.938063	0.361090	2.093723
C	2.113065	1.788133	2.276222
H	2.679986	1.894240	3.202532
H	2.672331	2.224671	1.445662
H	1.142630	2.279257	2.381508

Int-I
E(r^2scan-3c) = -615.195916 (conv)
Lowest Freq. = 31.34 cm⁻¹

C	-2.469178	0.816265	-3.167090
C	-2.558892	-0.390714	-3.871869
C	-1.582268	-1.386439	-3.745022
C	-0.508634	-1.184628	-2.906229
C	-0.400589	0.035477	-2.184601
C	-1.400872	1.035658	-2.325982
H	-3.239623	1.570981	-3.284787
H	-3.406207	-0.558827	-4.530895
H	-1.676340	-2.311537	-4.303909
H	0.257795	-1.945615	-2.794476
H	-1.312444	1.965597	-1.772501
C	0.705475	0.264901	-1.368825
C	1.064248	1.377013	-0.452458
C	2.037821	0.364741	0.257966
C	1.852357	-0.573774	-0.961449
H	0.256538	1.842494	0.119785
H	1.614666	2.163105	-0.990993
H	1.687705	-1.644471	-0.812139
H	2.695231	-0.444108	-1.661626
C	3.450108	0.843077	0.495951
O	4.360942	0.583382	-0.262779
O	3.696197	1.607350	1.568993
C	2.636917	1.930631	2.507388
H	3.094194	2.612366	3.223692
H	1.806967	2.432897	2.004028
H	2.293188	1.030093	3.023223
H	1.576818	-0.043747	1.159824

2
E(r^2scan-3c) = -614.790917 (conv)
Lowest Freq. = 27.36 cm⁻¹

C	-0.135711	0.898371	-1.378825
C	1.443570	0.871622	-1.464342
H	-0.636481	0.813525	-2.348735
H	-0.542813	1.755450	-0.830249
H	1.811546	0.741084	-2.487867
C	0.006427	-0.380664	-0.576102
C	1.353340	-0.383545	-0.628842
H	2.124852	-1.037297	-0.234143
C	-1.010160	-1.239437	0.006032
C	-0.664648	-2.406084	0.708393
C	-1.650064	-3.213879	1.258124
C	-2.997794	-2.875455	1.117694
C	-3.351293	-1.721397	0.422780
C	-2.366012	-0.908617	-0.129355
H	0.382970	-2.674921	0.819143
H	-1.370269	-4.113580	1.799328
H	-3.766617	-3.510506	1.548688
H	-4.398005	-1.452795	0.310152
H	-2.645349	-0.008020	-0.670786
C	2.086689	2.107046	-0.895828
O	2.359170	3.095672	-1.550139
O	2.283269	2.015967	0.430656
C	2.824276	3.199303	1.068755
H	2.906663	2.945598	2.125339
H	3.806347	3.437100	0.651997
H	2.148096	4.046460	0.927132

H_2SO_4
E(r^2scan-3c) = -700.209268 (conv)
Lowest Freq. = 208.43 cm⁻¹

S	0.203086	0.408837	-0.242167
O	0.370718	1.845107	-0.160671
O	0.724045	-0.390847	-1.343724
O	0.782088	-0.161439	1.158677
O	-1.392539	0.164671	-0.107809
H	0.925672	-1.124592	1.091390
H	-1.614070	-0.741737	-0.393697

HSO_4^-
E(r^2scan-3c) = -699.774652 (conv)
Lowest Freq. = 201.13 cm⁻¹

S	0.267429	0.401505	-0.208844
O	0.352401	1.879966	-0.203727
O	0.757613	-0.246806	-1.453198
O	0.746389	-0.255305	1.035471
O	-1.403055	0.176712	-0.216866
H	-1.563776	-0.781072	-0.215836

TS1
E(r^{2}scan-3c) = -1230.010135 (conv)
Lowest Freq. = -80.33 cm⁻¹

C	6.644238	0.192834	1.517009
C	6.951248	0.401066	0.002947
H	5.687316	0.621895	1.837292
H	7.437396	0.510121	2.200247
H	6.226770	1.003035	-0.558447
C	6.617108	-1.285835	1.217797
C	6.801054	-1.107034	-0.128268
H	7.011395	-1.785750	-0.948781
C	6.417524	-2.426371	2.061934
C	6.656645	-3.731811	1.586463
C	6.412305	-4.823387	2.404467
C	5.934444	-4.631307	3.702363
C	5.710220	-3.342611	4.188586
C	5.953117	-2.244231	3.378067
H	7.066076	-3.875528	0.589680
H	6.603354	-5.827901	2.039022
H	5.747357	-5.489851	4.340996
H	5.347190	-3.199571	5.201901
H	5.777080	-1.237787	3.748357
C	8.350884	0.867084	-0.327772
O	9.264002	0.963575	0.464131
O	8.457618	1.114868	-1.641457
C	9.778022	1.501784	-2.106457
H	10.497253	0.706877	-1.894272
H	9.672613	1.650903	-3.180353
H	10.090087	2.427087	-1.616208
C	4.273613	-2.800464	-0.851208
C	4.420500	-2.579800	-2.377575
H	5.095167	-3.374168	-0.405109
H	3.333600	-3.254069	-0.518390
H	3.528877	-2.836957	-2.953180
C	4.323847	-1.316964	-0.659653
C	4.527847	-1.033947	-2.124982
H	5.470228	-0.563172	-2.420374
C	3.714835	-0.511960	0.331682
C	3.265822	-1.093749	1.541986
C	2.659643	-0.306144	2.501545
C	2.488304	1.063220	2.274882
C	2.925514	1.651284	1.086321
C	3.537556	0.875376	0.117664
H	2.310415	-0.751000	3.428114
H	2.003055	1.675166	3.029847
H	2.780560	2.713961	0.920013
H	3.861509	1.323062	-0.817522
C	5.642978	-3.191096	-3.007844
O	6.547478	-3.726324	-2.396516
O	5.622076	-3.029778	-4.335195
C	6.786338	-3.519824	-5.053344
H	6.593010	-3.294174	-6.101192
H	7.684220	-3.003902	-4.704482
H	6.891674	-4.596686	-4.901979
H	3.709891	-0.444475	-2.554638
H	3.391449	-2.160060	1.705033

Int-II
E(r^{2}scan-3c) = -1230.036869 (conv)
Lowest Freq. = 18.29 cm⁻¹

C	6.431393	0.135639	1.550639
C	6.640277	0.305834	0.008372
H	5.465109	0.584642	1.825762
H	7.201850	0.463700	2.251892
H	6.103831	1.123377	-0.472908
C	6.284278	-1.313546	1.296988
C	6.036589	-1.137646	-0.156488
H	6.589957	-1.835160	-0.794109
C	6.516478	-2.440591	2.089097
C	6.570961	-3.734885	1.506389
C	6.758710	-4.837460	2.312467
C	6.890059	-4.671796	3.695810
C	6.848967	-3.401995	4.284493
C	6.676948	-2.287888	3.491919
H	6.502869	-3.845362	0.427894
H	6.813568	-5.830036	1.877699
H	7.037075	-5.545266	4.324990
H	6.957889	-3.298932	5.359108
H	6.641543	-1.295393	3.930923
C	8.105377	0.271554	-0.334538
O	8.880226	-0.517277	0.173070
O	8.431925	1.175175	-1.258377
C	9.828193	1.184296	-1.667563
H	10.094492	0.216003	-2.097766
H	9.902167	1.974162	-2.413508
H	10.465429	1.400471	-0.807045
C	4.049103	-2.588635	-0.935488
C	4.364870	-2.403243	-2.433965
H	4.558154	-3.411399	-0.429071
H	2.973911	-2.672820	-0.761359
H	3.548146	-2.634729	-3.122260
C	4.545190	-1.138879	-0.644918
C	4.550647	-0.869506	-2.180865
H	5.448035	-0.400170	-2.595690
C	3.648345	-0.294719	0.219472
C	3.097666	-0.844472	1.383235
C	2.292521	-0.077188	2.217259
C	2.024758	1.254096	1.899449
C	2.562644	1.807279	0.741790
C	3.368995	1.036680	-0.095166
H	1.869570	-0.519163	3.114987
H	1.394202	1.853863	2.549458
H	2.353328	2.841387	0.483155
H	3.773716	1.479458	-1.001711
C	5.613973	-3.087953	-2.916158
O	6.424136	-3.665188	-2.212297
O	5.746756	-2.956313	-4.243010
C	6.950251	-3.524189	-4.822154
H	6.886281	-3.307898	-5.887929
H	7.834725	-3.054702	-4.384309
H	6.977252	-4.602638	-4.648035
H	3.669182	-0.311606	-2.503350
H	3.299807	-1.883461	1.636869

TS2
E(r^{2}scan-3c) = -1230.022725 (conv)
Lowest Freq. = -257.91 cm⁻¹

C	6.134629	0.248689	1.451671
C	6.504518	0.179863	-0.051033
H	5.386722	1.014559	1.676772

H	6.981208	0.363357	2.132583
H	6.046741	0.947122	-0.680802
C	5.622962	-1.179151	1.366452
C	5.815926	-1.229591	-0.172928
H	6.467146	-2.028660	-0.538404
C	6.019713	-2.211733	2.306177
C	6.212990	-3.538432	1.883950
C	6.627278	-4.502059	2.790629
C	6.834308	-4.160254	4.127165
C	6.631918	-2.847456	4.559019
C	6.228425	-1.878036	3.656733
H	6.056085	-3.809259	0.844007
H	6.791428	-5.522323	2.457856
H	7.154371	-4.918770	4.835659
H	6.792518	-2.586663	5.600575
H	6.064436	-0.856782	3.990320
C	7.976570	0.069119	-0.341726
O	8.838682	-0.148690	0.484399
O	8.200682	0.174621	-1.659958
C	9.574819	-0.020382	-2.086814
H	9.910579	-1.024837	-1.816879
H	9.559728	0.103750	-3.168989
H	10.221494	0.726999	-1.620900
C	3.989090	-2.593198	-1.419372
C	4.449461	-2.199687	-2.837889
H	4.459470	-3.472303	-0.971986
H	2.900886	-2.686115	-1.365510
H	3.740908	-2.382551	-3.650164
C	4.463020	-1.197818	-0.900236
C	4.528204	-0.710921	-2.367046
H	5.414207	-0.142308	-2.663716
C	3.600688	-0.508477	0.115847
C	3.597415	-1.179197	1.379877
C	3.075216	-0.523322	2.523098
C	2.640782	0.781456	2.423113
C	2.651889	1.422075	1.175240
C	3.124408	0.782076	0.022915
H	3.026586	-1.055270	3.468929
H	2.269582	1.308902	3.295805
H	2.314733	2.452720	1.105661
H	3.168377	1.323148	-0.917329
C	5.797663	-2.739410	-3.235637
O	6.508459	-3.454820	-2.555665
O	6.140980	-2.292485	-4.453807
C	7.454675	-2.686006	-4.925976
H	7.561838	-2.218088	-5.904014
H	8.223795	-2.324382	-4.238258
H	7.514653	-3.773974	-5.010039
H	3.630229	-0.162672	-2.663086
H	3.599230	-2.266864	1.376860

pre-4

E(r^{2}scan-3c) = -1230.030871 (conv)

Lowest Freq. = 15.27 cm^{-1}

C	6.390700	0.229854	1.536353
C	6.669494	0.176639	0.021455
H	5.828494	1.105370	1.872053
H	7.299786	0.132063	2.132700
H	6.199806	0.978175	-0.558587
C	5.581639	-1.098036	1.449012
C	5.895397	-1.178128	-0.094327
H	6.505308	-2.022524	-0.422926
C	6.009301	-2.208017	2.368200
C	6.013409	-3.538895	1.942173
C	6.374987	-4.559499	2.816343
C	6.729586	-4.263686	4.130847
C	6.722101	-2.940899	4.565465
C	6.366359	-1.919579	3.688770
H	5.742901	-3.784448	0.918668
H	6.385007	-5.588208	2.467425
H	7.014518	-5.060693	4.811646
H	6.998640	-2.700957	5.588237
H	6.371095	-0.889104	4.035673
C	8.113188	0.071348	-0.387449
O	9.063035	0.038954	0.366850
O	8.212142	-0.023745	-1.725042
C	9.554342	-0.183415	-2.251679
H	9.996282	-1.110890	-1.878461
H	9.436498	-0.221371	-3.334191
H	10.175802	0.667508	-1.961873
C	4.024779	-2.423483	-1.442717
C	4.530705	-2.019473	-2.838260
H	4.455795	-3.319828	-0.990206
H	2.933641	-2.467326	-1.403884
H	3.823668	-2.142705	-3.663230
C	4.573475	-1.056838	-0.867635
C	4.686795	-0.552419	-2.328759
H	5.620216	-0.041365	-2.578636
C	3.666314	-0.420558	0.117532
C	4.034228	-0.857127	1.471362
C	3.384963	-0.193815	2.599987
C	2.380022	0.700937	2.372562
C	2.010435	1.016954	1.046138
C	2.632363	0.464659	-0.077920
H	3.680245	-0.473920	3.608366
H	1.865456	1.182685	3.197264
H	1.218788	1.746714	0.893147
H	2.333600	0.782086	-1.071893
C	5.843736	-2.645853	-3.230652
O	6.465939	-3.459161	-2.575996
O	6.255466	-2.168710	-4.415315
C	7.523110	-2.682206	-4.898588
H	7.710189	-2.155666	-5.833863
H	8.314575	-2.476347	-4.173870
H	7.449547	-3.759194	-5.069908
H	3.840099	0.066125	-2.634402
H	3.594355	-1.886475	1.525192

4

E(r^{2}scan-3c) = -1229.659360 (conv)

Lowest Freq. = 20.17 cm^{-1}

C	6.659844	-0.197079	1.980333
C	6.567871	0.315157	0.529958
H	6.280610	0.470316	2.759165
H	7.680981	-0.506783	2.219062
H	5.834603	1.115894	0.393702
C	5.771500	-1.414490	1.599126
C	5.978568	-1.070829	0.081129
H	6.715128	-1.691796	-0.434984
C	6.178860	-2.762470	2.133823

C	5.931363	-3.926018	1.399955
C	6.269337	-5.175092	1.911760
C	6.854003	-5.280878	3.172864
C	7.098307	-4.127876	3.914580
C	6.765281	-2.877897	3.396875
H	5.470370	-3.854722	0.418031
H	6.076518	-6.068549	1.323952
H	7.119108	-6.255620	3.572800
H	7.553903	-4.199525	4.898606
H	6.964057	-1.983045	3.981469
C	7.856421	0.671924	-0.148944
O	8.965750	0.359421	0.237187
O	7.635262	1.343930	-1.295680
C	8.811568	1.656291	-2.081276
H	9.326771	0.736483	-2.371055
H	8.440343	2.182249	-2.960855
H	9.489559	2.295191	-1.509337
C	4.366420	-2.156774	-1.673013
C	4.551468	-1.143205	-2.822594
H	5.073055	-2.990843	-1.651953
H	3.341595	-2.536624	-1.634733
H	3.770866	-1.122280	-3.586897
C	4.608906	-1.026958	-0.615134
C	4.459140	0.009533	-1.768953
H	5.225049	0.787162	-1.826853
C	3.651422	-0.950841	0.545879
C	4.287587	-1.167308	1.770721
C	3.565115	-1.160413	2.959968
C	2.191615	-0.928463	2.914526
C	1.551349	-0.713119	1.691601
C	2.277605	-0.723608	0.501984
H	4.063747	-1.335841	3.910773
H	1.614192	-0.917353	3.834936
H	0.479388	-0.536967	1.668265
H	1.771672	-0.559390	-0.447052
C	5.898519	-1.171453	-3.483819
O	6.885065	-1.749972	-3.069757
O	5.898628	-0.417097	-4.600332
C	7.164974	-0.319869	-5.296843
H	6.977831	0.333728	-6.148581
H	7.926672	0.110393	-4.641835
H	7.486223	-1.308802	-5.634225
H	3.470230	0.475087	-1.781135

TS1’

E(r[2]scan-3c) = -1230.009382 (conv)
Lowest Freq. = -94.70 cm^{-1}

C	6.817463	-0.681126	1.867069
C	7.151703	0.140756	0.585623
H	5.955684	-0.296762	2.425817
H	7.657236	-0.843610	2.548750
H	6.554512	1.045291	0.423258
C	6.517496	-1.836121	0.944290
C	6.713509	-1.091577	-0.191645
H	6.813315	-1.346268	-1.241567
C	6.135808	-3.190644	1.213097
C	6.139825	-4.164818	0.193621
C	5.733506	-5.460515	0.470109
C	5.322403	-5.803242	1.759617
C	5.327245	-4.850276	2.779240
C	5.735679	-3.552454	2.513178
H	6.491685	-3.902506	-0.801027
H	5.743998	-6.211031	-0.314410
H	5.006719	-6.820556	1.972585
H	5.013812	-5.125644	3.781666
H	5.741048	-2.805174	3.302209
C	8.614766	0.459306	0.374689
O	9.531184	0.013663	1.031848
O	8.763794	1.273483	-0.679973
C	10.132883	1.602533	-1.036534
H	10.685924	0.691665	-1.278551
H	10.055061	2.250712	-1.908528
H	10.618847	2.124654	-0.208798
C	3.938921	-1.851584	-1.295697
C	4.427228	-1.138297	-2.602204
H	4.433326	-2.786615	-1.010434
H	2.855101	-2.032266	-1.269659
H	5.377336	-1.540378	-2.960361
C	4.276494	-0.605807	-0.537886
C	4.554161	0.173715	-1.791793
H	5.480221	0.750043	-1.870974
C	3.857210	-0.172160	0.742651
C	3.300025	-1.096750	1.657245
C	2.858300	-0.662554	2.892219
C	2.963609	0.689443	3.235277
C	3.509220	1.612806	2.341999
C	3.956837	1.191252	1.101458
H	3.214069	-2.143000	1.377570
H	2.425332	-1.368596	3.593858
H	2.609805	1.025526	4.205735
H	3.575987	2.660769	2.616441
H	4.356538	1.907989	0.389689
C	3.418657	-1.135660	-3.718859
O	2.630031	-0.240654	-3.944101
O	3.479221	-2.282178	-4.409587
C	2.504136	-2.433199	-5.475291
H	2.637937	-1.643793	-6.218840
H	2.703731	-3.411327	-5.911057
H	1.492085	-2.391224	-5.065267
H	3.713004	0.855090	-1.987753

Int-II’

E(r[2]scan-3c) = -1230.036178 (conv)
Lowest Freq. = 14.99 cm^{-1}

C	6.655385	-0.582781	1.852598
C	6.881073	0.084568	0.455824
H	5.780876	-0.118069	2.332544
H	7.475696	-0.646090	2.571274
H	6.485635	1.091018	0.319260
C	6.267765	-1.810928	1.125940
C	6.045985	-1.097467	-0.160294
H	6.468067	-1.589773	-1.043579
C	6.289410	-3.160475	1.485471
C	6.113362	-4.177586	0.510507
C	6.083522	-5.499206	0.899871
C	6.222978	-5.826496	2.253654
C	6.406300	-4.837038	3.226839
C	6.453506	-3.511912	2.852102
H	6.039634	-3.911998	-0.538727
H	5.960947	-6.285070	0.161958

H	6.197749	-6.870218	2.554258
H	6.514505	-5.116097	4.269741
H	6.590015	-2.730656	3.593765
C	8.320583	-0.050180	0.037700
O	8.946616	-1.085305	0.169930
O	8.802020	1.071123	-0.496812
C	10.184905	1.015957	-0.946059
H	10.293144	0.253588	-1.720987
H	10.395065	2.007180	-1.344905
H	10.840003	0.789106	-0.101853
C	3.864597	-1.815012	-1.319300
C	4.325470	-1.115273	-2.631361
H	4.174584	-2.851891	-1.170052
H	2.780574	-1.739704	-1.195406
H	5.234473	-1.561024	-3.040719
C	4.567305	-0.693612	-0.499487
C	4.597397	0.162975	-1.799354
H	5.515347	0.713547	-2.023679
C	3.814057	-0.162102	0.688618
C	3.189213	-1.055201	1.566164
C	2.518059	-0.588808	2.691177
C	2.461640	0.779521	2.954569
C	3.071729	1.675050	2.081780
C	3.742711	1.207040	0.952544
H	3.222407	-2.123791	1.362820
H	2.033691	-1.293282	3.361414
H	1.936282	1.144647	3.832345
H	3.023721	2.743001	2.274534
H	4.205476	1.916852	0.271618
C	3.269553	-1.008066	-3.695085
O	2.242018	-0.369757	-3.578778
O	3.581526	-1.730820	-4.783528
C	2.594922	-1.726361	-5.847586
H	2.442630	-0.707940	-6.213553
H	3.012101	-2.358543	-6.630699
H	1.648685	-2.134958	-5.484047
H	3.738718	0.838648	-1.834960

TS2’
E(r^{2}scan-3c) = -1230.022487 (conv)
Lowest Freq. = -256.49 cm^{-1}

C	6.342844	-0.418347	1.922287
C	6.727275	0.049307	0.492880
H	5.693739	0.292682	2.441077
H	7.182747	-0.676367	2.570825
H	6.397122	1.055509	0.225699
C	5.652989	-1.627103	1.316985
C	5.844173	-1.097338	-0.130737
H	6.378281	-1.768216	-0.811778
C	5.901146	-2.983393	1.770800
C	5.887547	-4.061628	0.869332
C	6.157608	-5.345502	1.316861
C	6.425335	-5.571843	2.667173
C	6.427075	-4.509214	3.573334
C	6.167403	-3.223031	3.130928
H	5.679162	-3.890177	-0.182252
H	6.161406	-6.173577	0.614835
H	6.632421	-6.579552	3.015653
H	6.633443	-4.690414	4.623669
H	6.162363	-2.395582	3.835303
C	8.176620	-0.156231	0.147153
O	8.900960	-0.972578	0.680728
O	8.546686	0.633711	-0.869322
C	9.907296	0.455378	-1.345957
H	10.050519	-0.570918	-1.692684
H	10.018067	1.160648	-2.168640
H	10.615702	0.680758	-0.545170
C	3.851691	-1.607512	-1.706359
C	4.446579	-0.762617	-2.872904
H	4.148692	-2.657315	-1.644018
H	2.760455	-1.526848	-1.667405
H	5.391758	-1.191964	-3.217283
C	4.512885	-0.601912	-0.713379
C	4.647393	0.402143	-1.882397
H	5.581064	0.966018	-1.957899
C	3.744675	-0.268138	0.530721
C	3.645834	-1.377167	1.429277
C	3.210276	-1.166349	2.761811
C	2.950628	0.114815	3.200005
C	3.048279	1.188540	2.302147
C	3.438913	1.001909	0.970444
H	3.502281	-2.366465	1.000512
H	3.089452	-2.017441	3.425800
H	2.648456	0.297202	4.226202
H	2.847220	2.195803	2.656763
H	3.554395	1.858806	0.313671
C	3.525975	-0.539345	-4.036784
O	2.779149	0.410125	-4.165532
O	3.596257	-1.560488	-4.907464
C	2.691681	-1.490323	-6.039506
H	2.904414	-0.599228	-6.635379
H	2.882251	-2.393698	-6.617925
H	1.655586	-1.465377	-5.692465
H	3.797463	1.090176	-1.919628

pre-4’
E(r^{2}scan-3c) = -1230.030586 (conv)
Lowest Freq. = 6.49 cm^{-1}

C	6.563412	-0.458601	1.997717
C	6.860676	0.013563	0.561554
H	6.088389	0.290274	2.637083
H	7.446458	-0.857412	2.500661
H	6.513075	1.026365	0.331501
C	5.614928	-1.567552	1.452142
C	5.918116	-1.090822	-0.021453
H	6.415581	-1.811724	-0.675967
C	5.918216	-2.979043	1.873465
C	5.764077	-4.048503	0.987141
C	6.007694	-5.353628	1.403959
C	6.401329	-5.608471	2.715992
C	6.550472	-4.549856	3.607928
C	6.312882	-3.243787	3.188443
H	5.459411	-3.865968	-0.039709
H	5.894029	-6.173246	0.700066
H	6.593699	-6.627235	3.040050
H	6.857336	-4.738735	4.632753
H	6.437325	-2.424133	3.891855
C	8.288896	-0.116704	0.105697
O	9.215209	-0.510692	0.783461
O	8.398360	0.233231	-1.186853

C	9.720584	0.102334	-1.770232
H	10.055714	-0.936228	-1.711639
H	9.609646	0.413285	-2.808503
H	10.427343	0.750983	-1.246793
C	3.931841	-1.491483	-1.670657
C	4.582018	-0.645524	-2.800850
H	4.200041	-2.548257	-1.601223
H	2.843912	-1.376249	-1.651846
H	5.521217	-1.099267	-3.129781
C	4.621782	-0.529645	-0.626637
C	4.795621	0.490330	-1.782396
H	5.752269	1.016689	-1.818239
C	3.783987	-0.241989	0.562416
C	4.105066	-1.196707	1.631351
C	3.526408	-0.957902	2.952205
C	2.611353	0.040825	3.118716
C	2.271807	0.870473	2.026743
C	2.838503	0.737539	0.755388
H	3.551755	-2.116265	1.306976
H	3.794301	-1.625567	3.767543
H	2.146142	0.215243	4.083158
H	1.551842	1.668603	2.191789
H	2.569483	1.434643	-0.031971
C	3.696557	-0.368978	-3.981194
O	3.004900	0.620137	-4.118307
O	3.729566	-1.389228	-4.853537
C	2.850778	-1.270083	-6.002052
H	3.122215	-0.391608	-6.592733
H	3.003087	-2.182357	-6.577732
H	1.811506	-1.189575	-5.673452
H	3.972817	1.208837	-1.823989

4’

E(r²scan-3c) = -1229.659327 (conv)
Lowest Freq. = 5.61 cm⁻¹

C	7.084870	-0.678358	1.905067
C	7.064805	-0.067156	0.489638
H	6.992049	0.021972	2.739818
H	7.966999	-1.309769	2.042046
H	6.600425	0.922039	0.451633
C	5.833042	-1.527479	1.550427
C	6.050982	-1.179074	0.032798
H	6.530311	-1.968325	-0.555113
C	5.817522	-2.959440	2.017625
C	5.144879	-3.943457	1.287681
C	5.096824	-5.258065	1.740841
C	5.713915	-5.608366	2.940839
C	6.378814	-4.633541	3.680075
C	6.432254	-3.319468	3.219841
H	4.652882	-3.677988	0.355858
H	4.575877	-6.011352	1.155904
H	5.677560	-6.634583	3.295556
H	6.862416	-4.895995	4.617230
H	6.957877	-2.567256	3.802820
C	8.358690	-0.068505	-0.268090
O	9.283434	-0.832697	-0.069969
O	8.358519	0.853266	-1.250010
C	9.531520	0.870802	-2.101110
H	9.653587	-0.096464	-2.595480
H	9.345437	1.656137	-2.833356
H	10.422478	1.098628	-1.510367
C	4.082586	-1.617652	-1.617865
C	4.697333	-0.728933	-2.741727
H	4.377659	-2.670124	-1.606358
H	2.990796	-1.535501	-1.599684
H	5.652217	-1.139376	-3.081745
C	4.728432	-0.667183	-0.556825
C	4.868983	0.388543	-1.694196
H	5.796455	0.965476	-1.731071
C	3.921032	-0.343875	0.671179
C	4.521387	-0.825117	1.837503
C	3.911925	-0.650055	3.076040
C	2.691675	0.020451	3.138863
C	2.088379	0.502439	1.974462
C	2.699354	0.321884	0.734919
H	4.378825	-1.031719	3.981492
H	2.204843	0.167678	4.099053
H	1.135366	1.020678	2.036393
H	2.222773	0.694579	-0.169251
C	3.793739	-0.470183	-3.907657
O	2.933033	0.387809	-3.950207
O	4.010580	-1.353255	-4.901839
C	3.124676	-1.248724	-6.043892
H	3.230403	-0.268363	-6.515709
H	3.437976	-2.038213	-6.726602
H	2.087513	-1.398240	-5.732463
H	4.011450	1.067973	-1.710535

Int-II+HSO_4^-

E(r²scan-3c) = -1929.837189 (conv)
Lowest Freq. = 20.82 cm⁻¹

C	6.519316	0.400288	1.247909
C	6.613166	0.566527	-0.302373
H	5.529827	0.744070	1.587816
H	7.292039	0.824511	1.893950
H	5.929073	1.280382	-0.761034
C	6.516688	-1.063507	1.034129
C	6.225646	-0.953510	-0.416440
H	6.868578	-1.564689	-1.061911
C	6.839405	-2.134349	1.871189
C	6.974758	-3.446375	1.347203
C	7.235557	-4.497325	2.201603
C	7.362052	-4.261066	3.574221
C	7.241477	-2.970554	4.105735
C	6.993867	-1.907723	3.265068
H	6.919550	-3.603378	0.276093
H	7.351884	-5.502801	1.810670
H	7.567166	-5.093764	4.241405
H	7.347381	-2.812240	5.173960
H	6.892405	-0.900870	3.658765
C	8.030748	0.757856	-0.761162
O	8.970148	0.144646	-0.275222
O	8.127405	1.649367	-1.739923
C	9.452704	1.849349	-2.304933
H	9.772652	0.935634	-2.810898
H	9.337646	2.664767	-3.017582
H	10.159412	2.117954	-1.516375
C	4.480677	-2.659836	-1.305016
C	4.336826	-2.207673	-2.773718
H	5.314693	-3.346519	-1.146122

H	3.575080	-3.091635	-0.874868
H	3.321602	-2.340805	-3.165445
C	4.745413	-1.177668	-0.881845
C	4.622440	-0.735518	-2.374224
H	5.545872	-0.329437	-2.795246
C	3.754535	-0.596063	0.097298
C	3.374959	-1.344111	1.218907
C	2.522031	-0.808026	2.177240
C	2.030541	0.488701	2.031026
C	2.397312	1.239065	0.919044
C	3.253773	0.700549	-0.041486
H	2.235996	-1.405673	3.038297
H	1.361856	0.907330	2.777699
H	2.017717	2.249155	0.792771
H	3.526822	1.303783	-0.902652
C	5.273651	-2.854508	-3.757154
O	5.778099	-3.950702	-3.615740
O	5.450323	-2.081277	-4.838673
C	6.358790	-2.602601	-5.841195
H	6.411002	-1.832841	-6.610965
H	7.342475	-2.771490	-5.395582
H	5.968491	-3.535917	-6.255944
H	3.798827	-0.050093	-2.580578
H	3.748913	-2.357808	1.343034
O	8.074705	-1.327001	-3.012687
O	10.385909	-1.623466	-2.059815
O	9.568351	-3.194318	-3.753275
S	9.025127	-2.410548	-2.623958
H	10.080562	-0.992614	-1.374191
O	8.533903	-3.224836	-1.476267

TS3
E(r[2]scan-3c) = -1929.830361 (conv)
Lowest Freq. = -613.42 cm^{-1}

C	7.776973	-0.094364	1.197684
C	6.958246	0.518946	0.037030
H	7.673570	0.424097	2.156877
H	8.838893	-0.247690	0.977581
H	6.187116	1.202216	0.402241
C	6.941511	-1.333878	1.063526
C	6.366409	-0.916633	-0.168866
H	7.202103	-1.488475	-0.949476
C	6.855612	-2.437879	1.960665
C	5.980405	-3.523205	1.757407
C	5.949129	-4.572603	2.658600
C	6.782913	-4.557600	3.778320
C	7.656605	-3.490885	3.993884
C	7.697658	-2.440510	3.093729
H	5.340147	-3.548497	0.885686
H	5.276266	-5.408139	2.492556
H	6.753180	-5.382546	4.484466
H	8.306492	-3.486235	4.863359
H	8.383450	-1.614431	3.255247
C	7.699714	1.152104	-1.102757
O	8.860273	0.922945	-1.409525
O	6.932068	2.020380	-1.762131
C	7.521926	2.634205	-2.940146
H	7.808377	1.860160	-3.656490
H	6.740821	3.272950	-3.349935
H	8.396554	3.223491	-2.655409
C	4.812786	-2.662384	-1.349571
C	4.693248	-2.166662	-2.806822
H	5.685079	-3.304505	-1.207182
H	3.925552	-3.162285	-0.955803
H	3.667466	-2.234450	-3.189133
C	5.030111	-1.187149	-0.865006
C	5.059489	-0.730838	-2.367203
H	6.044411	-0.412444	-2.713142
C	3.909632	-0.573930	-0.044544
C	3.208584	-1.318830	0.909259
C	2.224563	-0.725498	1.695989
C	1.925416	0.625990	1.547200
C	2.617813	1.378875	0.602106
C	3.599684	0.784049	-0.184735
H	1.687476	-1.326405	2.424721
H	1.155139	1.087400	2.158654
H	2.391788	2.433714	0.472265
H	4.125415	1.387215	-0.920161
C	5.575386	-2.867777	-3.800575
O	5.829699	-4.055883	-3.771569
O	6.026080	-2.029570	-4.747941
C	6.902870	-2.616557	-5.740870
H	7.179516	-1.796924	-6.403600
H	7.788561	-3.034684	-5.254704
H	6.379512	-3.400505	-6.294485
H	4.327782	0.033280	-2.634022
H	3.415404	-2.375770	1.041213
O	7.895259	-2.033200	-2.010302
O	10.298387	-1.385398	-1.771465
O	9.716822	-3.663691	-2.461886
S	9.245551	-2.616215	-1.551689
H	9.846871	-0.547753	-1.498299
O	9.210059	-2.930341	-0.110288

3+H_2SO_4
E(r[2]scan-3c) = -1929.861404 (conv)
Lowest Freq. = 16.72 cm^{-1}

C	7.631276	-0.210406	1.839682
C	6.788338	0.562219	0.772163
H	7.526206	0.180493	2.857719
H	8.691091	-0.299686	1.580551
H	6.121417	1.319799	1.195968
C	6.779517	-1.419247	1.503059
C	6.099937	-0.785515	0.519335
H	9.114282	-0.830898	-1.376837
C	6.773906	-2.725819	2.141888
C	5.631178	-3.539013	2.188787
C	5.670276	-4.779382	2.814217
C	6.846384	-5.227807	3.413971
C	7.980968	-4.418384	3.396162
C	7.943894	-3.176407	2.773765
H	4.700622	-3.183109	1.758616
H	4.775068	-5.394510	2.845015
H	6.874148	-6.197160	3.903606
H	8.898392	-4.756345	3.869999
H	8.833184	-2.552104	2.757217
C	7.550141	1.145165	-0.380325
O	8.546917	0.653490	-0.896325
O	7.016459	2.286688	-0.814977
C	7.645155	2.891049	-1.978119

H	7.620018	2.192331	-2.817946
H	7.052481	3.779143	-2.192947
H	8.678290	3.159515	-1.746158
C	4.866412	-2.608648	-0.880233
C	4.898886	-2.242231	-2.382880
H	5.734710	-3.203573	-0.589661
H	3.956429	-3.098496	-0.529839
H	3.896603	-2.059163	-2.776447
C	5.083403	-1.104208	-0.524536
C	5.598499	-0.910410	-1.997147
H	6.685615	-1.014713	-2.046284
C	3.806707	-0.325741	-0.229788
C	2.775509	-0.891702	0.525856
C	1.641832	-0.152501	0.852887
C	1.523096	1.171742	0.438569
C	2.550988	1.750663	-0.301154
C	3.682128	1.008734	-0.630390
H	0.849063	-0.615201	1.434352
H	0.637701	1.748078	0.691663
H	2.474314	2.784847	-0.625763
H	4.475793	1.481961	-1.201391
C	5.658368	-3.136343	-3.309054
O	6.749568	-3.648095	-3.064683
O	5.042340	-3.316786	-4.471965
C	5.734255	-4.138440	-5.453836
H	5.080874	-4.150119	-6.324890
H	6.700807	-3.691156	-5.696449
H	5.875958	-5.147052	-5.059250
H	5.283626	-0.019420	-2.543845
H	2.849566	-1.919615	0.867740
O	10.332088	-2.715495	0.062977
O	8.241949	-3.457972	-0.939512
O	10.371233	-3.777440	-2.236642
S	9.718186	-2.930960	-1.243809
H	7.675741	-3.502358	-1.783939
O	9.479487	-1.531090	-1.993606

Int-II’+HSO$_4^-$
E(r2scan-3c) = -1929.838517 (conv)
Lowest Freq. = 19.47 cm⁻¹

C	6.692818	-0.882900	1.687881
C	6.937818	-0.141555	0.330320
H	5.847572	-0.401942	2.203001
H	7.515160	-1.034488	2.390857
H	6.593480	0.890183	0.261985
C	6.232234	-2.039417	0.889349
C	6.043024	-1.242448	-0.346888
H	6.438721	-1.709811	-1.257672
C	6.194043	-3.410466	1.159172
C	6.007713	-4.349708	0.111346
C	5.933355	-5.694713	0.410168
C	6.032227	-6.118265	1.739327
C	6.225478	-5.203771	2.782451
C	6.322963	-3.858609	2.500401
H	5.985629	-4.009150	-0.919120
H	5.808060	-6.423695	-0.383798
H	5.968913	-7.178826	1.966856
H	6.303610	-5.557069	3.805438
H	6.470610	-3.133760	3.295495
C	8.360353	-0.317348	-0.120423
O	8.940313	-1.391604	-0.036917
O	8.891011	0.799411	-0.595206
C	10.247007	0.697123	-1.114219
H	10.252920	0.029696	-1.978909
H	10.516154	1.711215	-1.405809
H	10.916344	0.324064	-0.335757
C	3.854735	-1.797659	-1.559910
C	4.358314	-1.016968	-2.808904
H	4.139215	-2.849425	-1.479489
H	2.771408	-1.705612	-1.434915
H	5.262993	-1.479132	-3.216408
C	4.587765	-0.753772	-0.664963
C	4.662990	0.189956	-1.903324
H	5.604476	0.718615	-2.076304
C	3.851791	-0.272694	0.555090
C	3.190363	-1.194428	1.374544
C	2.540768	-0.775643	2.530709
C	2.543133	0.573216	2.884977
C	3.190939	1.497751	2.071317
C	3.839723	1.077390	0.910746
H	3.180181	-2.247856	1.101600
H	2.027675	-1.502108	3.154702
H	2.034951	0.901205	3.787306
H	3.190436	2.551531	2.335487
H	4.333544	1.809154	0.276447
C	3.356079	-0.829316	-3.907221
O	2.773355	0.205183	-4.166015
O	3.154726	-1.984625	-4.566417
C	2.174287	-1.942567	-5.632709
H	2.482664	-1.227551	-6.399734
H	2.146199	-2.952948	-6.039701
H	1.195802	-1.659962	-5.235479
H	3.832199	0.900843	-1.900575
S	8.196845	-2.354411	-3.361251
O	7.950697	-0.913025	-3.069748
O	7.109464	-3.236063	-2.839989
O	8.610895	-2.659177	-4.746047
O	9.560456	-2.754125	-2.489213
H	9.456582	-2.362295	-1.596399

TS3’
E(r^{2}scan-3c) = -1929.827122 (conv)
Lowest Freq. = -724.16 cm⁻¹

C	7.507331	-1.405556	1.690177
C	7.289547	-0.373100	0.558532
H	7.152432	-1.075632	2.674026
H	8.527138	-1.793272	1.786516
H	6.651836	0.460766	0.863634
C	6.555238	-2.314041	0.980679
C	6.466978	-1.488483	-0.173488
H	7.330548	-2.164667	-0.851874
C	6.026147	-3.571017	1.385337
C	5.305261	-4.393634	0.495165
C	4.816364	-5.614998	0.921072
C	5.023906	-6.028858	2.239178
C	5.741193	-5.228251	3.129361
C	6.251795	-4.013120	2.706435
H	5.168134	-4.082959	-0.534321
H	4.274554	-6.252594	0.229507
H	4.630572	-6.985431	2.571331

H	5.903834	-5.560174	4.150006
H	6.814053	-3.388295	3.394023
C	8.515517	0.135109	-0.144353
O	9.617584	-0.388851	-0.066337
O	8.260216	1.221635	-0.867525
C	9.366988	1.743918	-1.654307
H	9.724143	0.972680	-2.340187
H	8.952210	2.589958	-2.200603
H	10.172449	2.067178	-0.990636
C	4.890752	-2.219199	-2.203128
C	4.752499	-1.050178	-3.205824
H	5.738860	-2.853597	-2.473903
H	4.007108	-2.835058	-2.031263
H	5.147528	-1.236990	-4.211914
C	5.294088	-1.179119	-1.105704
C	5.640779	-0.200619	-2.274615
H	6.690005	-0.292208	-2.559862
C	4.113768	-0.701837	-0.265824
C	3.135590	-1.596569	0.183032
C	2.089662	-1.164963	0.994334
C	2.003106	0.170530	1.377793
C	2.972802	1.069886	0.942616
C	4.016718	0.637228	0.129982
H	3.176258	-2.642419	-0.103699
H	1.337593	-1.877269	1.322361
H	1.183844	0.508747	2.005958
H	2.915240	2.116154	1.229911
H	4.752774	1.360545	-0.208131
C	3.361431	-0.493408	-3.379332
O	3.094392	0.689779	-3.458099
O	2.442690	-1.468669	-3.488945
C	1.078441	-1.028963	-3.698214
H	1.001816	-0.460668	-4.628824
H	0.485925	-1.941814	-3.755183
H	0.751373	-0.409122	-2.859006
H	5.375643	0.851192	-2.159024
S	9.122311	-2.590828	-2.621064
O	8.720918	-1.299888	-3.212180
O	8.131895	-3.040939	-1.534001
O	9.431770	-3.695996	-3.533414
O	10.532537	-2.348978	-1.822705
H	10.385825	-1.612967	-1.184909

3'+H_2SO_4
E(r^2scan-3c) = -1929.861003 (conv)
Lowest Freq. = 7.70 cm^{-1}

C	7.853538	-1.189729	1.442793
C	7.183366	0.040265	0.729950
H	8.036397	-1.020882	2.509253
H	8.770447	-1.546067	0.961385
H	6.994769	0.896894	1.384043
C	6.587707	-1.949779	1.115444
C	5.997975	-0.898571	0.494786
H	8.095146	-1.874468	-1.632347
C	6.253709	-3.324556	1.438940
C	4.945868	-3.826954	1.345136
C	4.672742	-5.151629	1.659947
C	5.697106	-6.001574	2.076187
C	6.997896	-5.512921	2.182825
C	7.274198	-4.186940	1.872034
H	4.137701	-3.169466	1.044788
H	3.654145	-5.522394	1.585740
H	5.481153	-7.037711	2.320860
H	7.800325	-6.167954	2.510271
H	8.290911	-3.812111	1.956511
C	7.940694	0.481583	-0.488437
O	8.312732	-0.262506	-1.392814
O	8.203233	1.784427	-0.492271
C	8.940840	2.291435	-1.639621
H	8.375061	2.102562	-2.554961
H	9.045657	3.360293	-1.459867
H	9.918230	1.807274	-1.696470
C	4.442713	-1.679057	-1.327183
C	5.118425	-0.649310	-2.279756
H	4.912654	-2.660652	-1.243037
H	3.382675	-1.783784	-1.575897
H	6.197892	-0.818355	-2.299118
C	4.667749	-0.652353	-0.168873
C	4.763853	0.454999	-1.272589
H	5.478940	1.269876	-1.124084
C	3.576806	-0.429337	0.859401
C	2.424928	-1.214345	0.922253
C	1.439472	-0.956663	1.875327
C	1.588295	0.094711	2.773311
C	2.731925	0.890709	2.712598
C	3.716213	0.628532	1.766926
H	2.281841	-2.037267	0.227975
H	0.552297	-1.583041	1.910833
H	0.819607	0.296249	3.514007
H	2.857420	1.718571	3.405179
H	4.601670	1.258183	1.726858
C	4.647794	-0.664846	-3.692598
O	4.961753	-1.557597	-4.482432
O	3.843227	0.331700	-4.021358
C	3.327703	0.328835	-5.383821
H	2.741115	-0.576461	-5.554273
H	2.701801	1.217246	-5.450931
H	4.156827	0.380108	-6.092685
H	3.771985	0.871300	-1.465678
S	7.997972	-3.212185	-3.367619
O	8.036141	-2.867903	-1.797359
O	6.455283	-3.517945	-3.646794
O	8.411959	-2.032840	-4.131663
O	8.688017	-4.486232	-3.513796
H	5.939720	-2.690849	-3.955536

9. NMR spectra

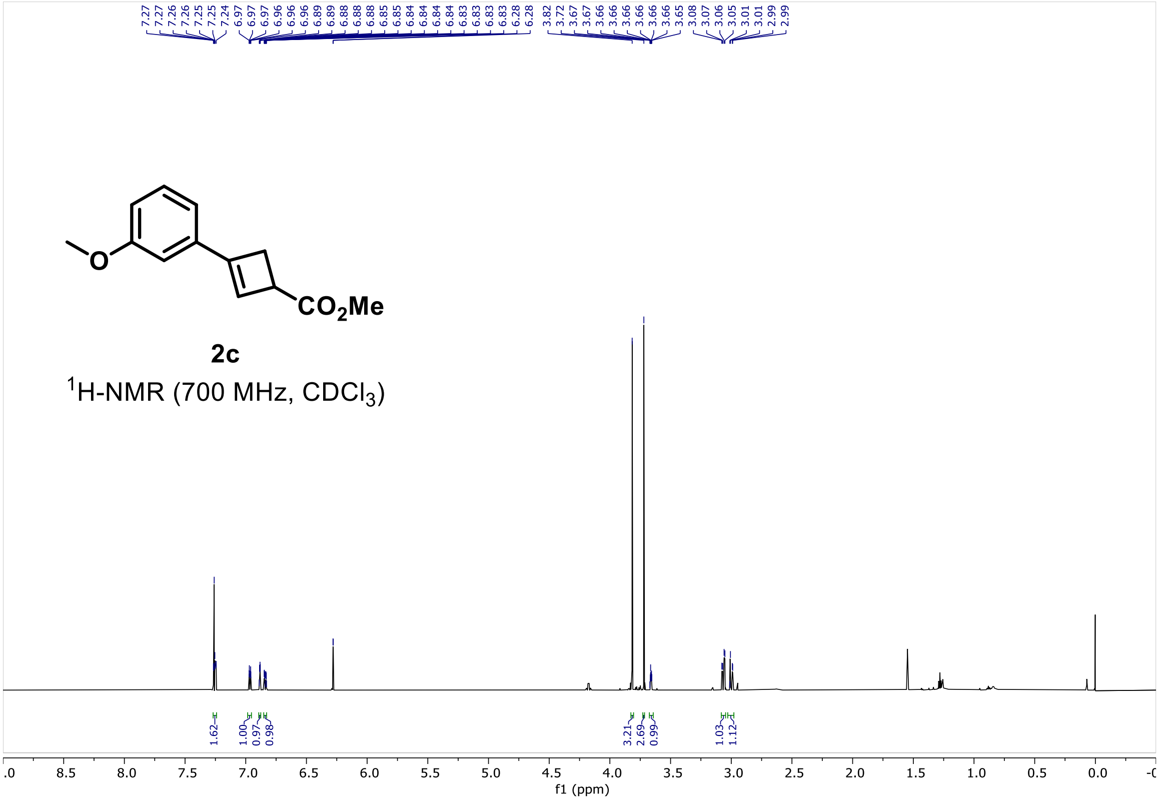

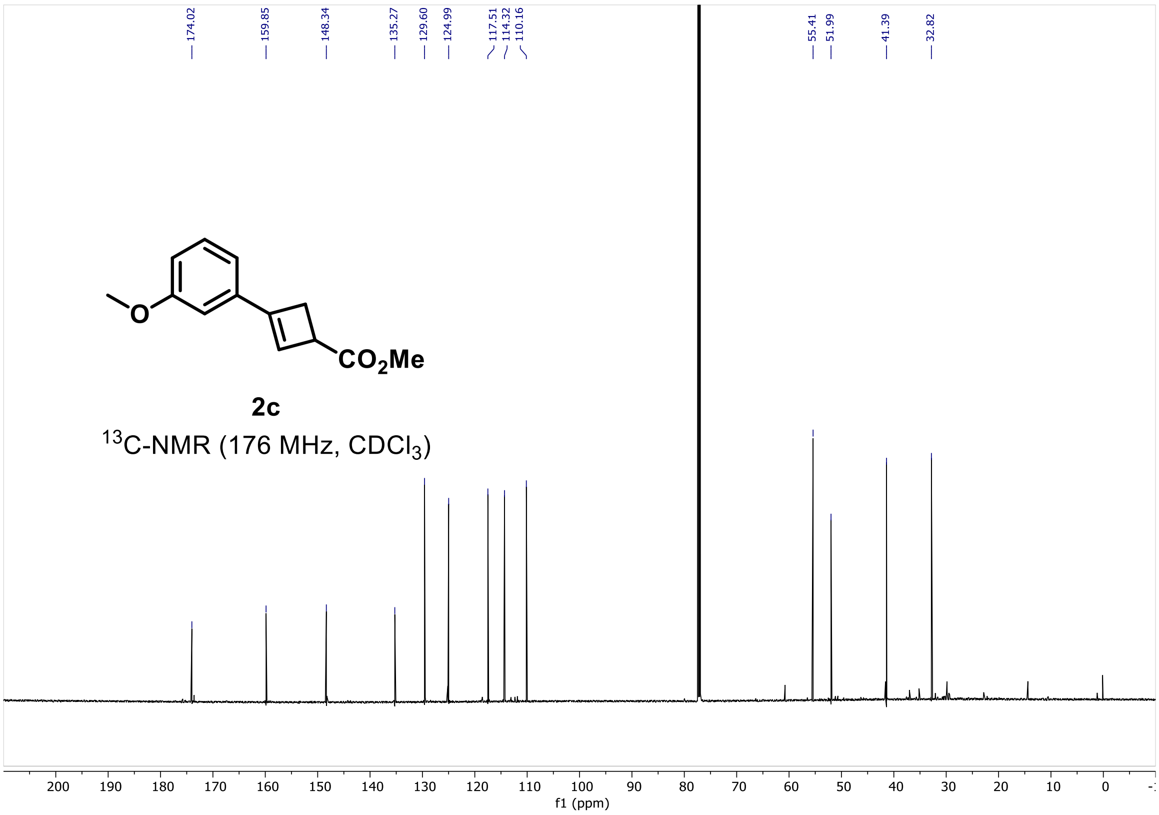

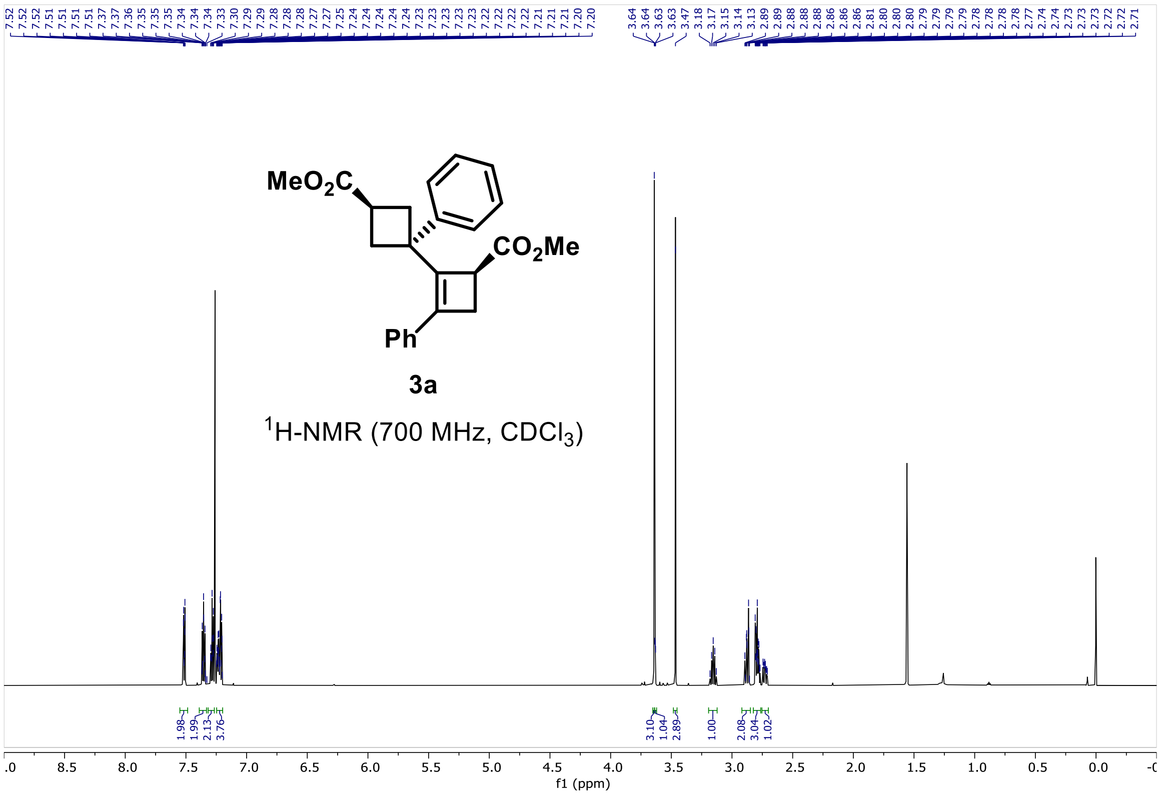
MeO2C
CO2Me
Ph
3a
1H-NMR (700 MHz, CDCl3)
f1 (ppm)

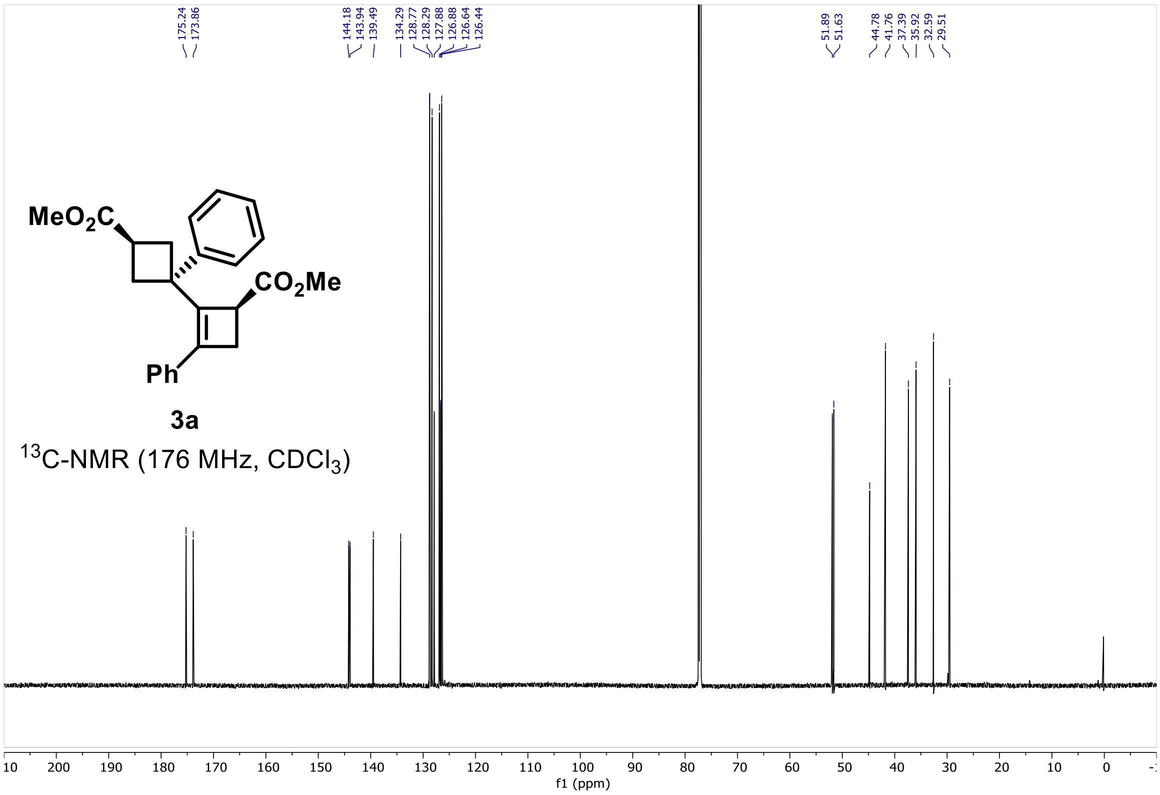
MeO2C
CO2Me
Ph
3a
13C-NMR (176 MHz, CDCl3)
f1 (ppm)

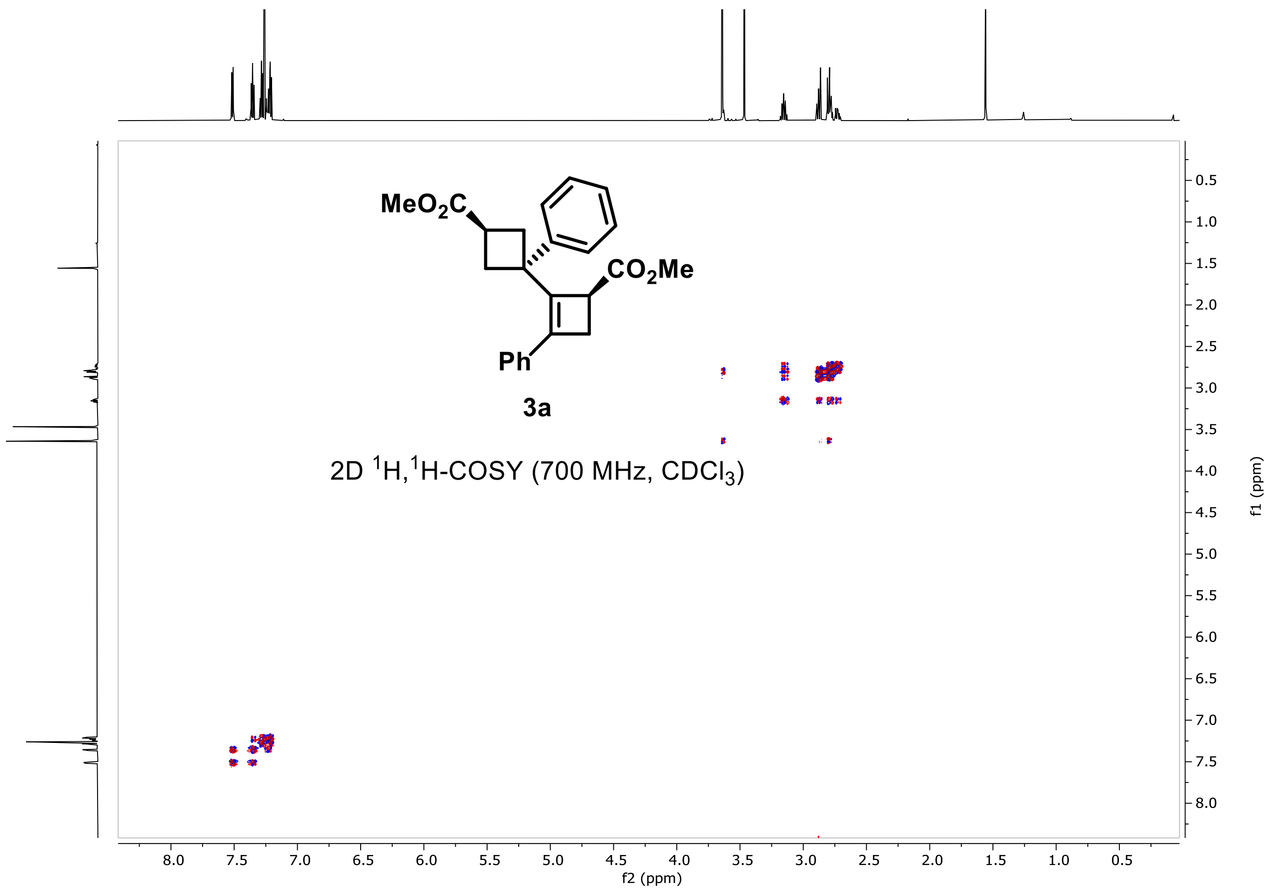
MeO2C
CO2Me
Ph
3a
2D 1H,1H-COSY (700 MHz, CDCl3)
f2 (ppm)
f1 (ppm)

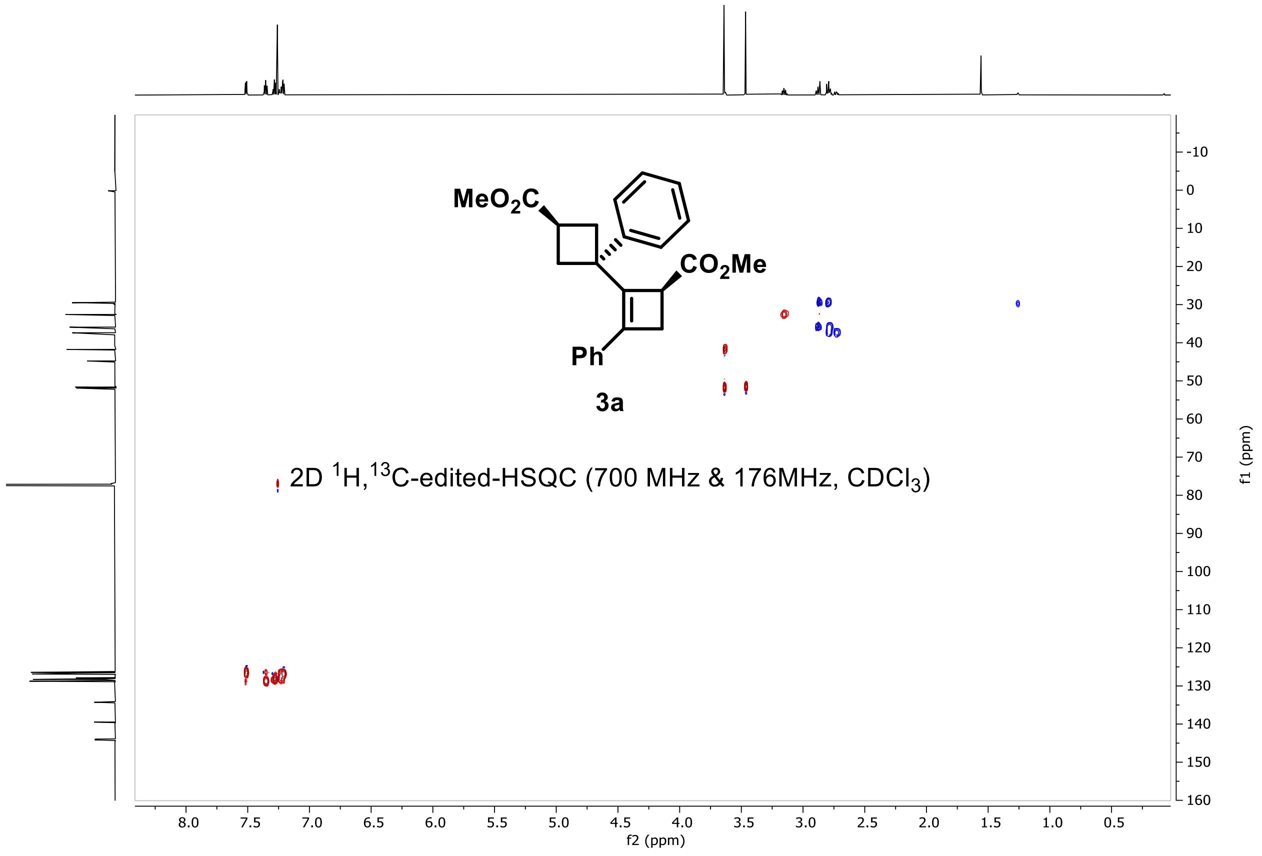
MeO2C
CO2Me
Ph
3a
2D 1H,13C-edited-HSQC (700 MHz & 176MHz, CDCl3)
f2 (ppm)
f1 (ppm)

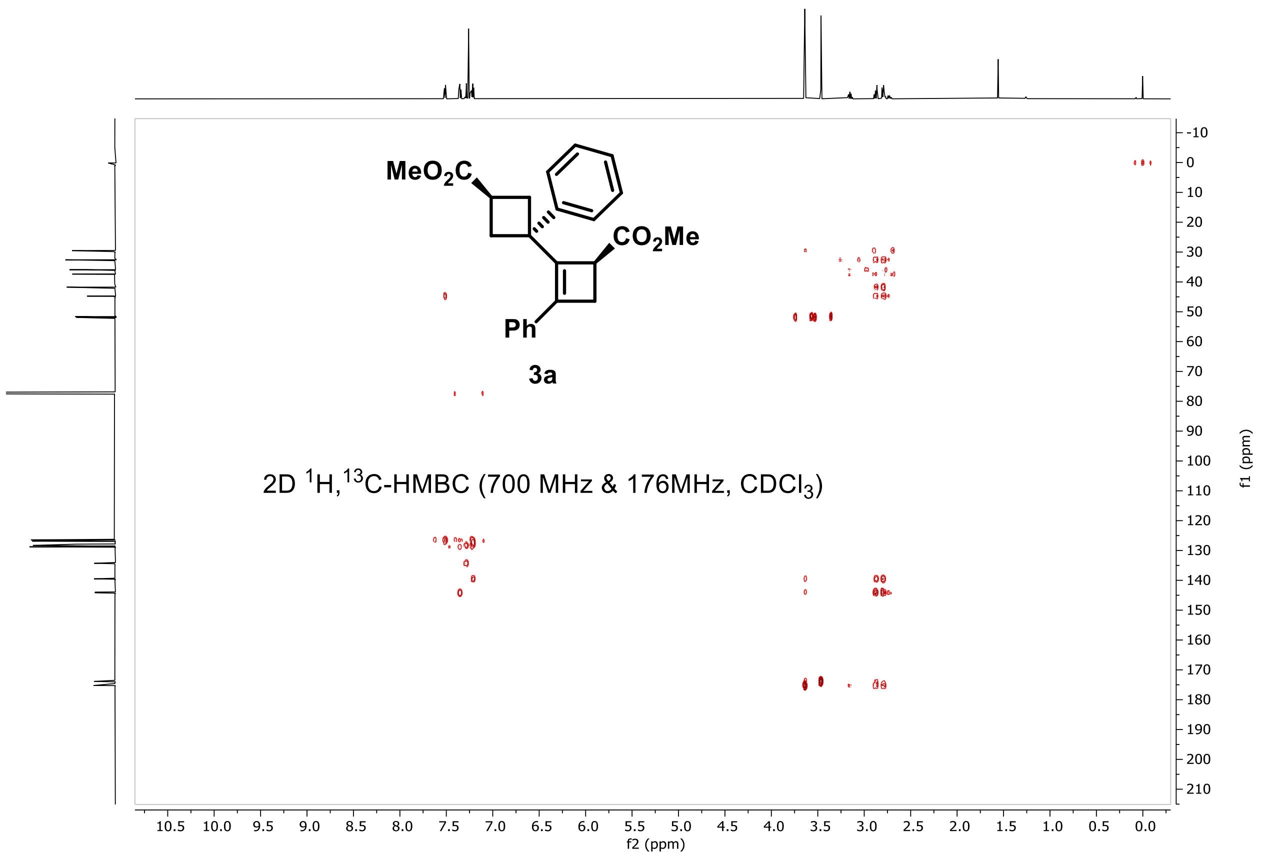
MeO2C
CO2Me
Ph
3a
2D 1H,13C-HMBC (700 MHz & 176MHz, CDCl3)
f2 (ppm)
f1 (ppm)

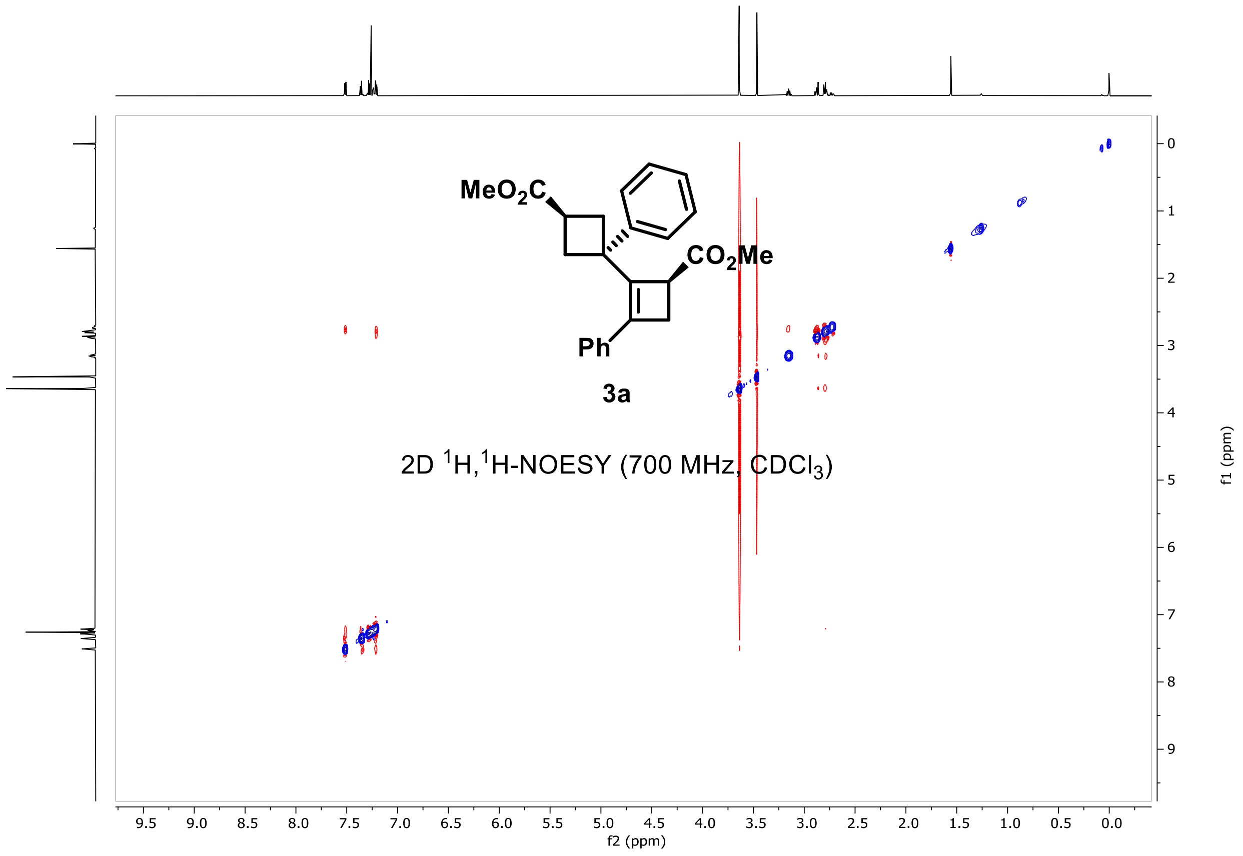
MeO2C
CO2Me
Ph
3a
2D 1H,1H-NOESY (700 MHz, CDCl3)
f2 (ppm)
f1 (ppm)

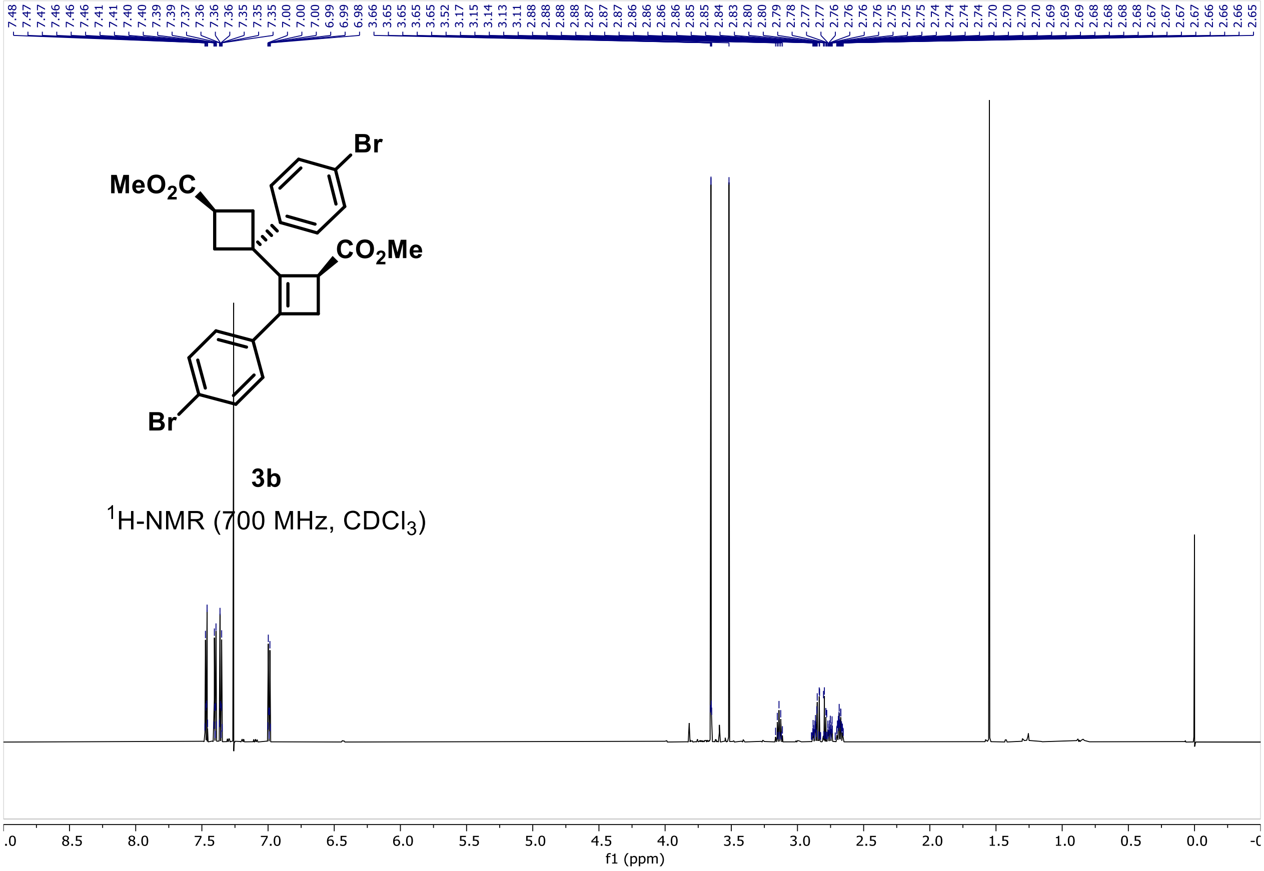

7.48 7.47 7.47 7.46 7.46 7.46 7.41 7.41 7.40 7.40 7.39 7.39 7.37 7.36 7.36 7.36 7.35 7.35 7.35 7.00 7.00 7.00 7.00 6.99 6.99 6.98 3.66 3.65 3.65 3.65 3.52 3.17 3.15 3.14 3.13 3.11 2.88 2.88 2.88 2.88 2.87 2.87 2.87 2.86 2.86 2.86 2.86 2.85 2.85 2.84 2.83 2.80 2.80 2.79 2.78 2.77 2.77 2.76 2.76 2.76 2.76 2.75 2.75 2.75 2.74 2.74 2.74 2.74 2.70 2.70 2.70 2.70 2.69 2.69 2.69 2.68 2.68 2.68 2.68 2.68 2.67 2.67 2.67 2.67 2.66 2.66 2.66 2.65
Br
MeO2C
CO2Me
Br
3b
1H-NMR (700 MHz, CDCl3)
f1 (ppm)

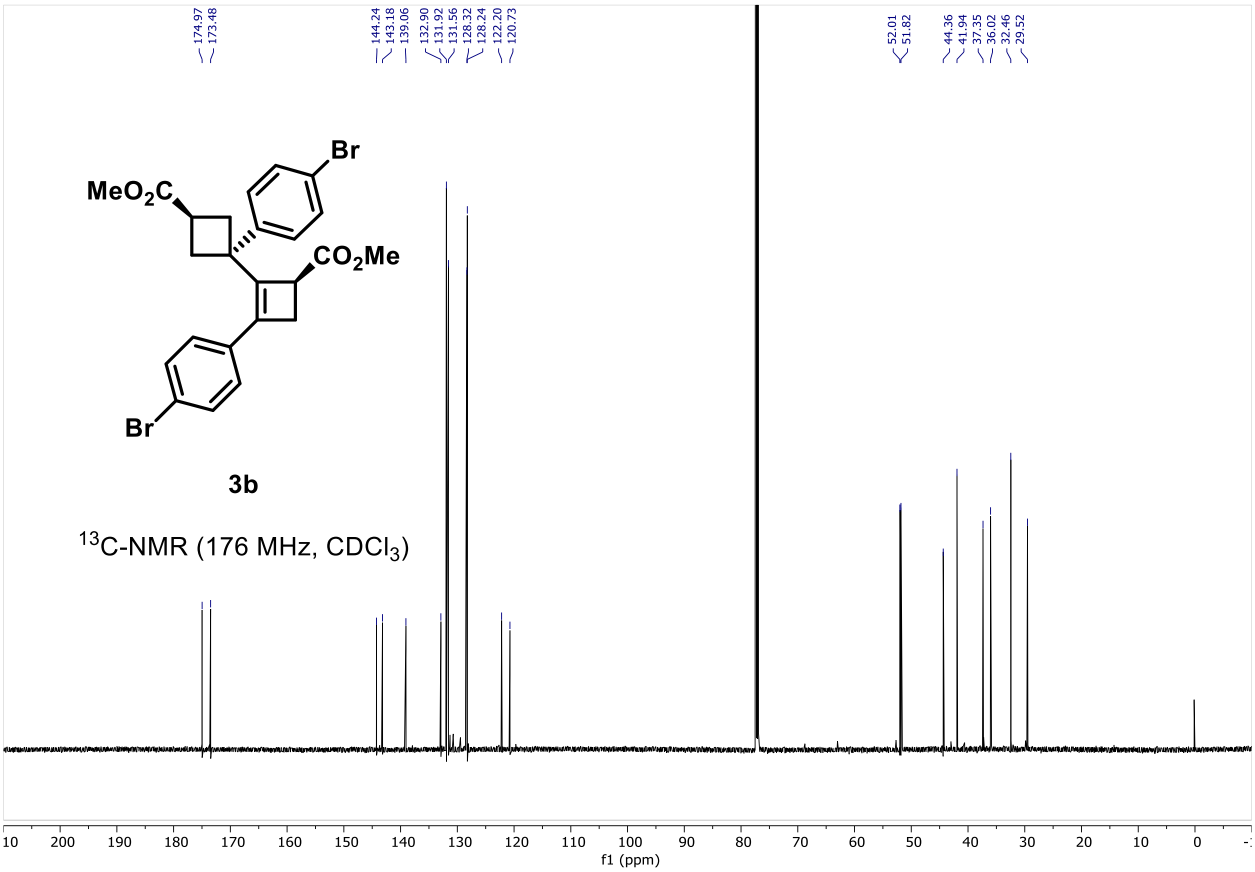

174.97 173.48 144.24 143.18 139.06 132.90 131.92 131.56 128.32 128.24 122.20 120.73 52.01 51.82 44.36 41.94 37.35 36.02 32.46 29.52
Br
MeO2C
CO2Me
Br
3b
13C-NMR (176 MHz, CDCl3)
f1 (ppm)

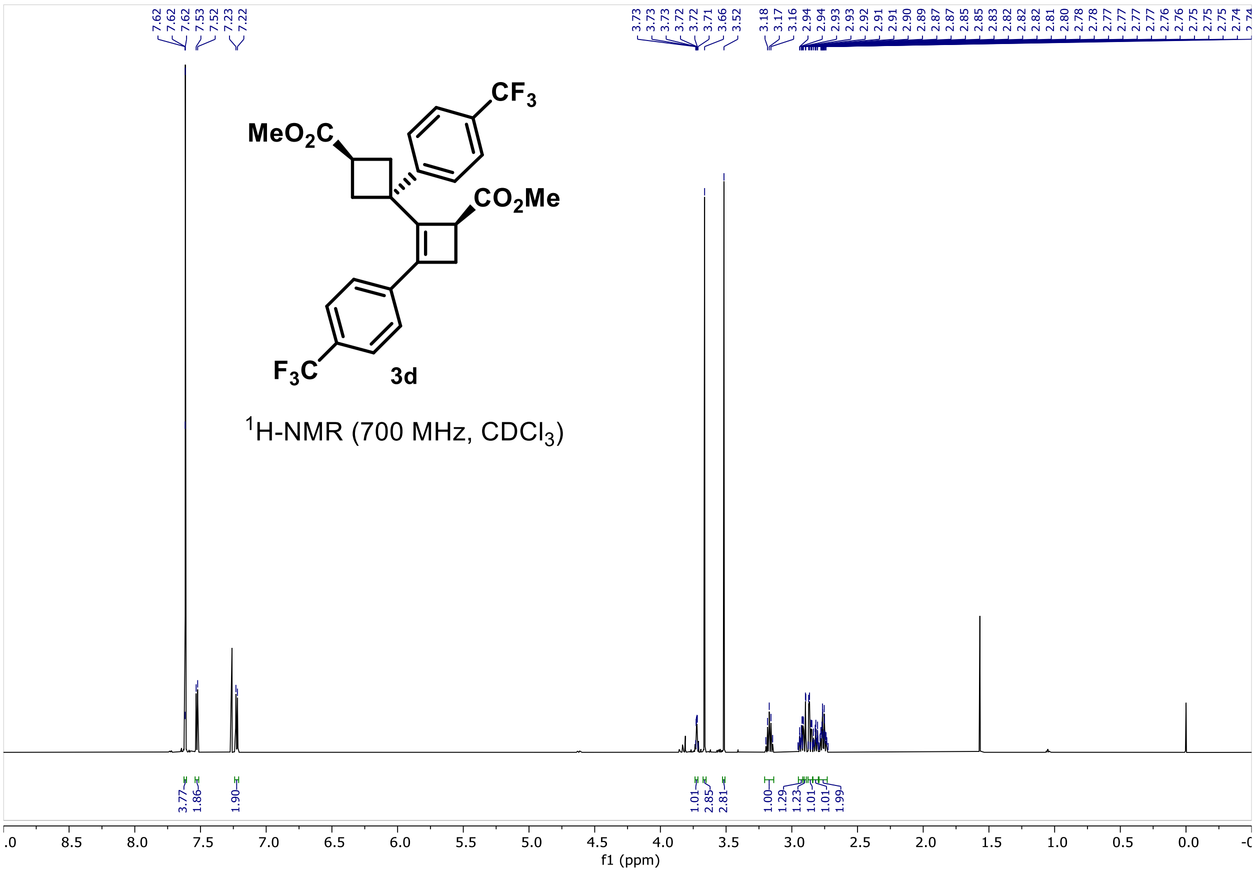

MeO2C
CF3
CO2Me
F3C
3d
1H-NMR (700 MHz, CDCl3)
f1 (ppm)

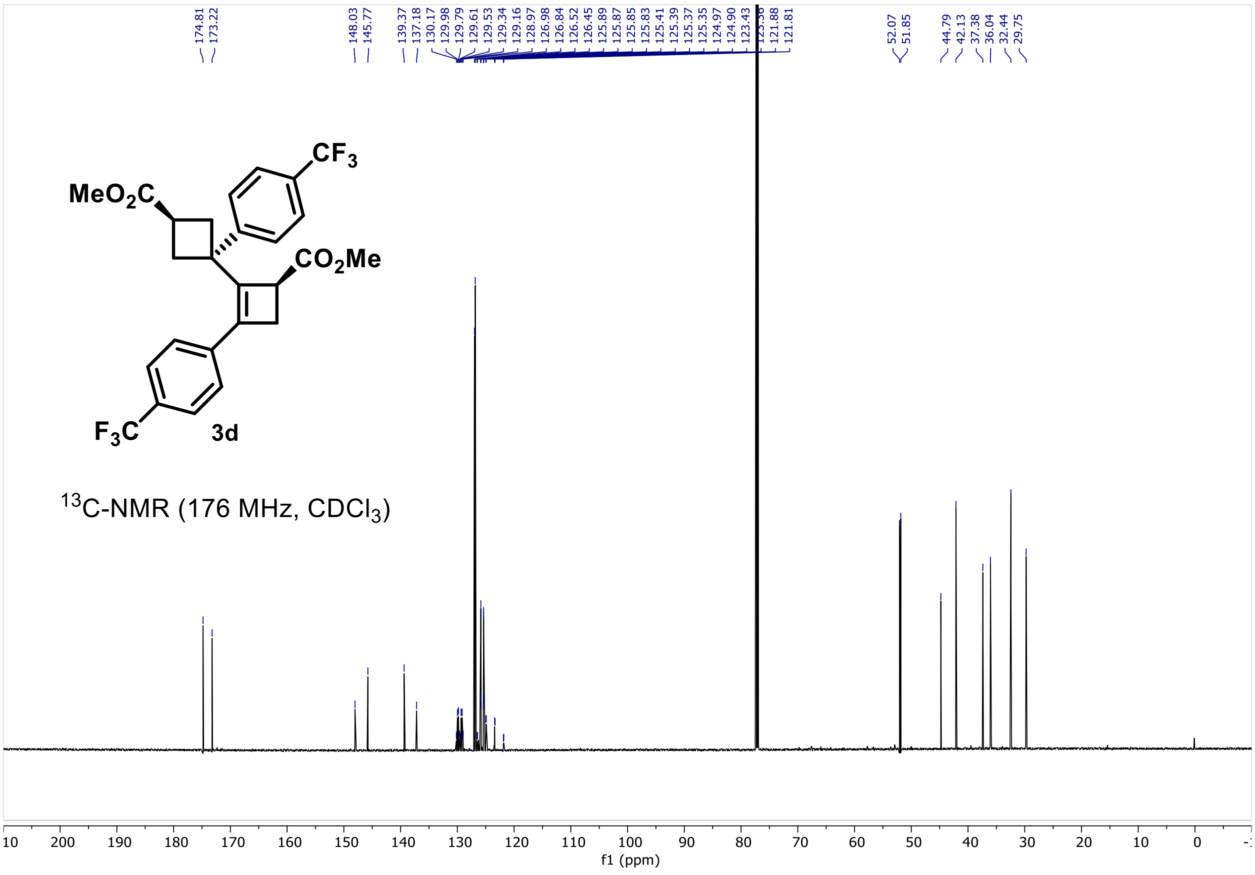

MeO2C
CF3
CO2Me
F3C
3d
13C-NMR (176 MHz, CDCl3)
f1 (ppm)

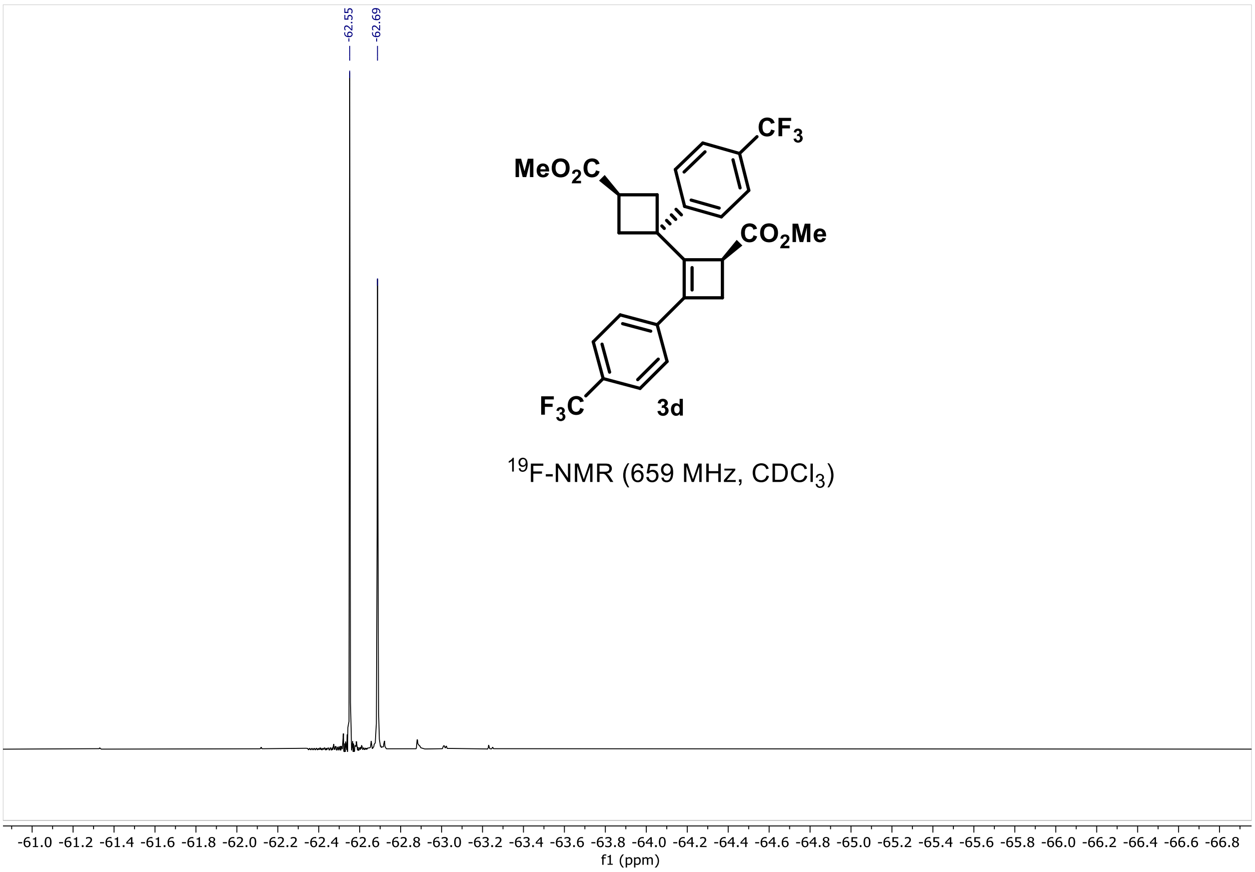
-62.55
-62.69
CF_3
MeO_2C
CO_2Me
F_3C
3d
^{19}F-NMR (659 MHz, $CDCl_3$)
-61.0 -61.2 -61.4 -61.6 -61.8 -62.0 -62.2 -62.4 -62.6 -62.8 -63.0 -63.2 -63.4 -63.6 -63.8 -64.0 -64.2 -64.4 -64.6 -64.8 -65.0 -65.2 -65.4 -65.6 -65.8 -66.0 -66.2 -66.4 -66.6 -66.8
f1 (ppm)

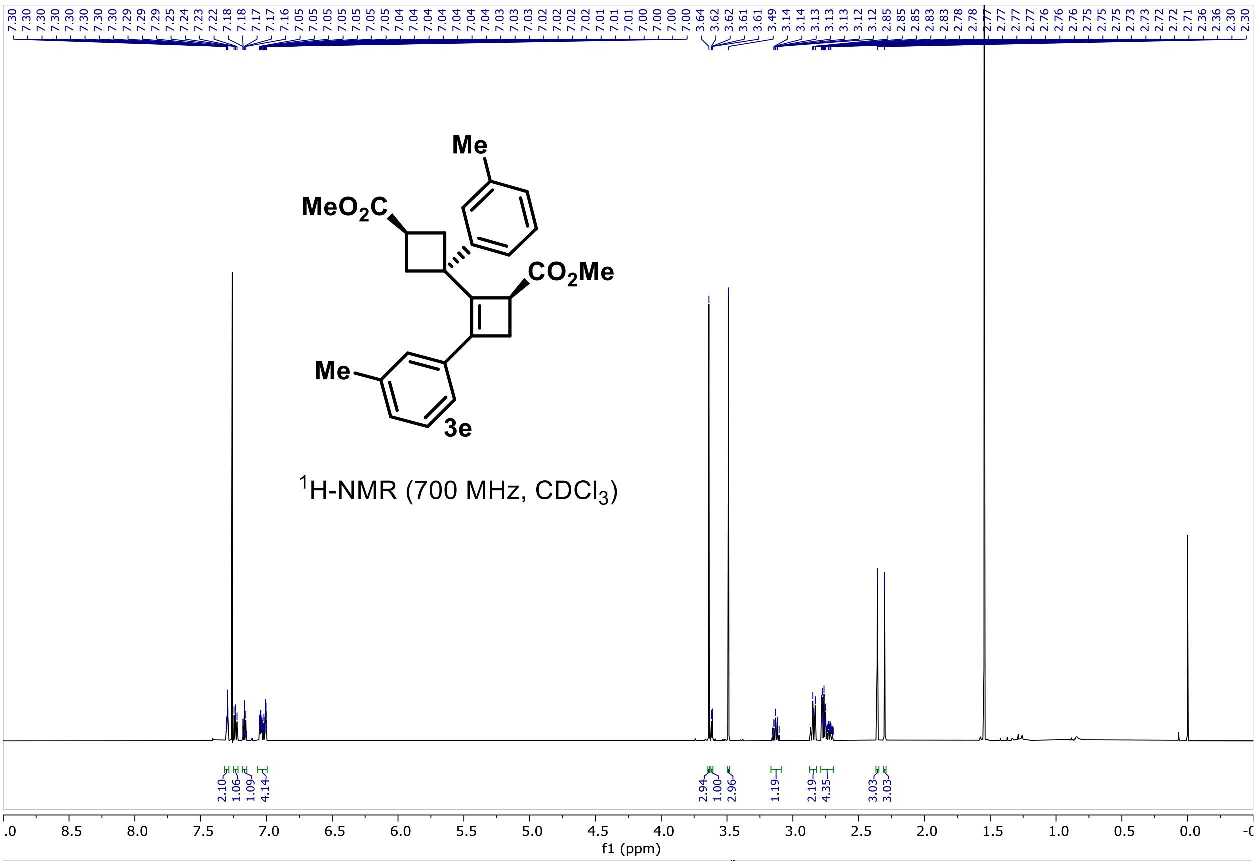
Me
MeO2C
CO2Me
Me
3e
1H-NMR (700 MHz, CDCl3)
f1 (ppm)

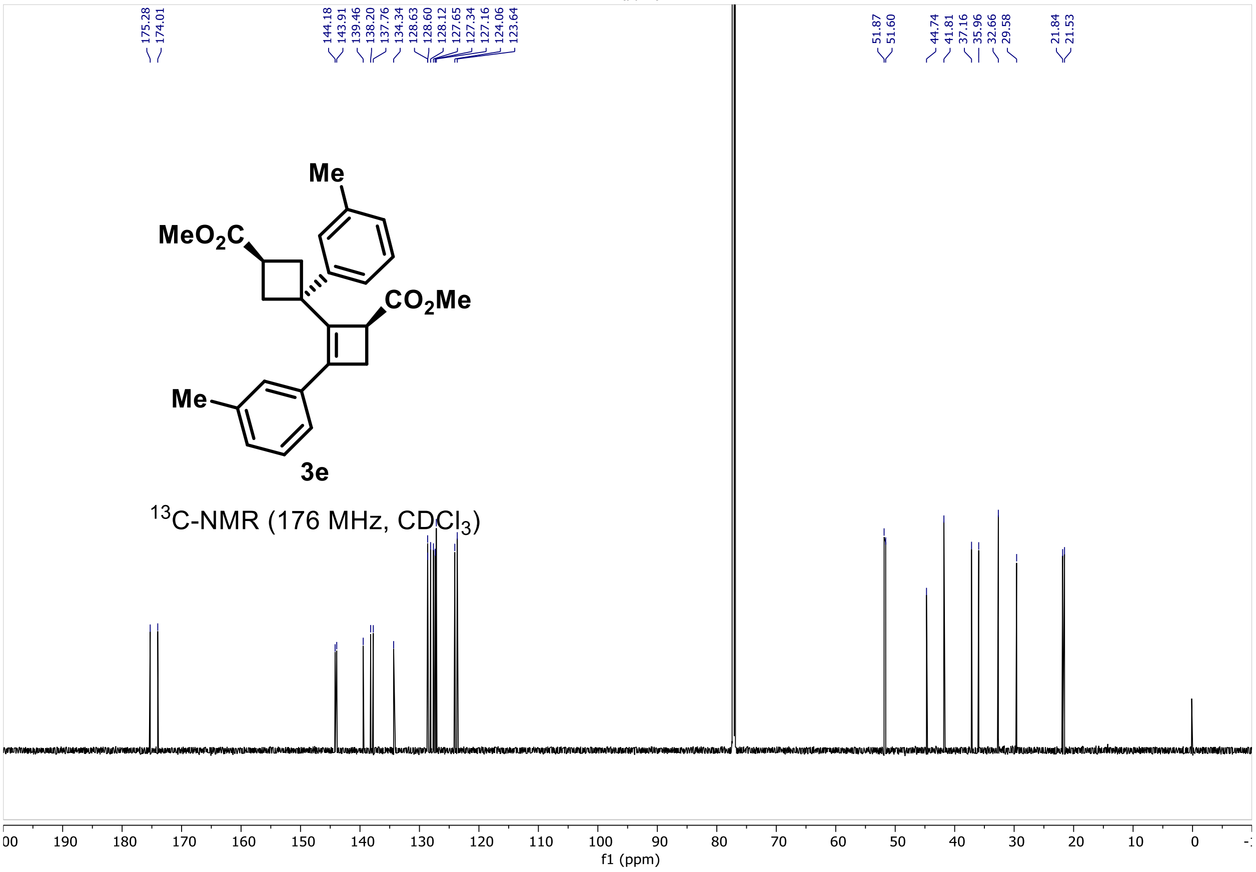
175.28
174.01
144.18
143.91
139.46
138.20
137.76
134.34
128.63
128.60
128.12
127.65
127.34
127.16
124.06
123.64
51.87
51.60
44.74
41.81
37.16
35.96
32.66
29.58
21.84
21.53
Me
MeO2C
CO2Me
Me
3e
13C-NMR (176 MHz, CDCl3)
f1 (ppm)

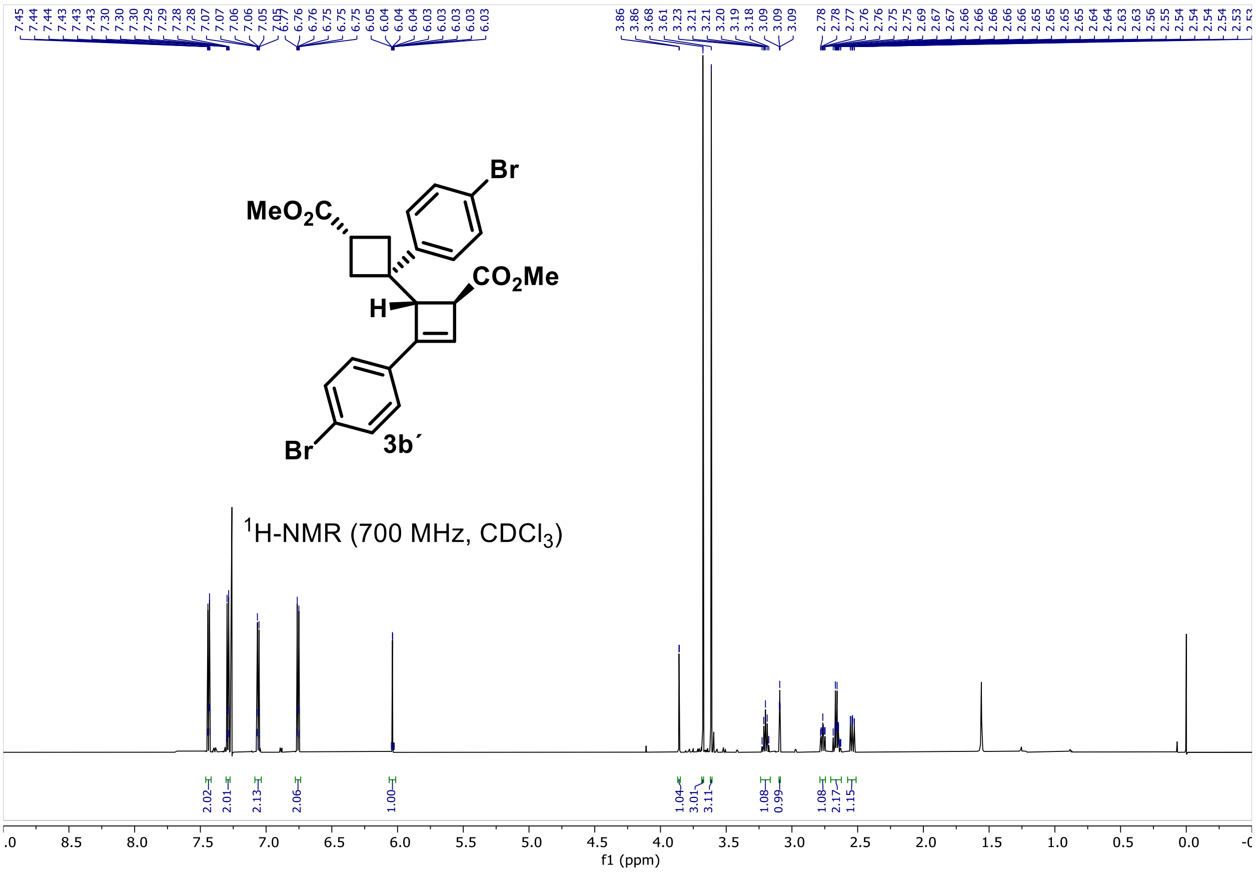

Br
MeO2C
CO2Me
H
Br
3b´
1H-NMR (700 MHz, CDCl3)
f1 (ppm)

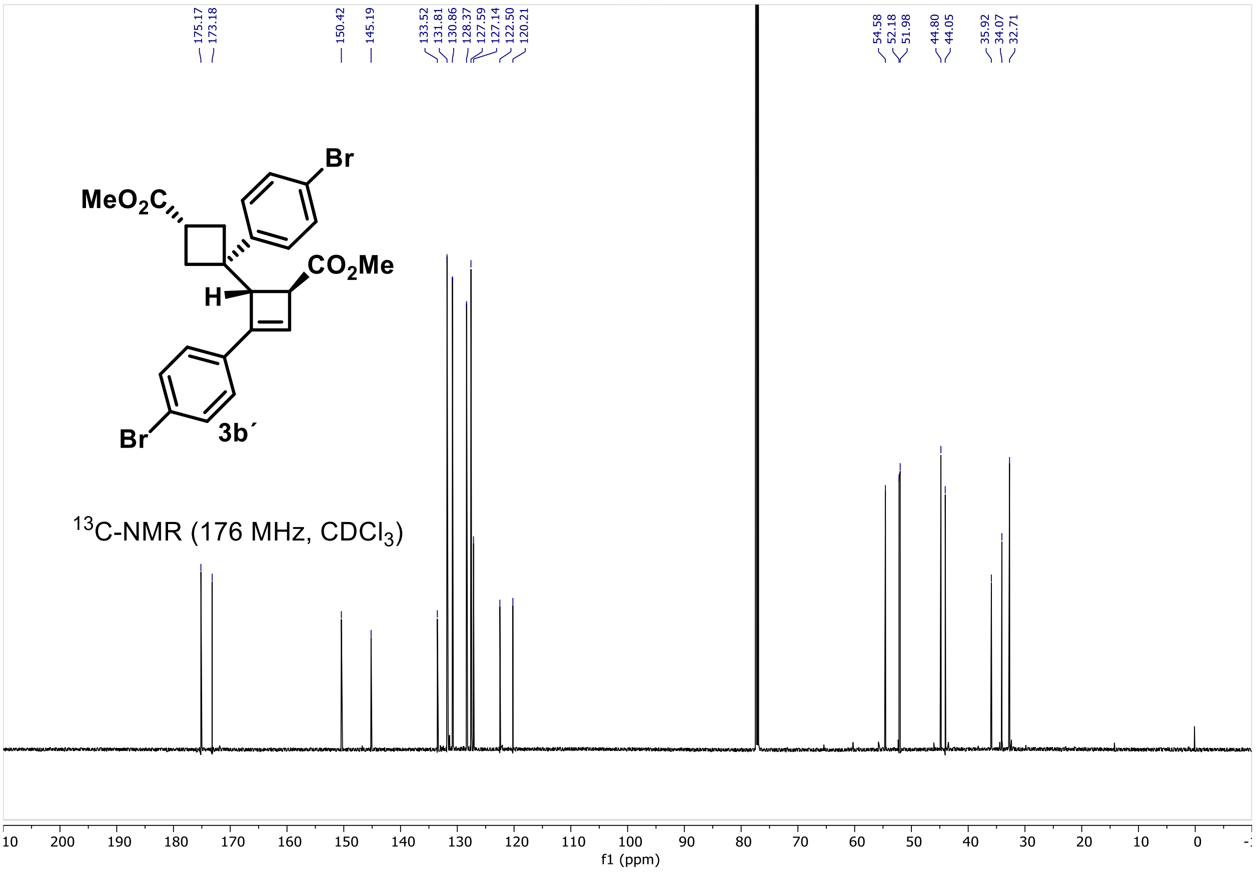

175.17
173.18
150.42
145.19
133.52
131.81
130.86
128.37
127.59
127.14
122.50
120.21
54.58
52.18
51.98
44.80
44.05
35.92
34.07
32.71
Br
MeO2C
CO2Me
H
Br
3b´
13C-NMR (176 MHz, CDCl3)
f1 (ppm)

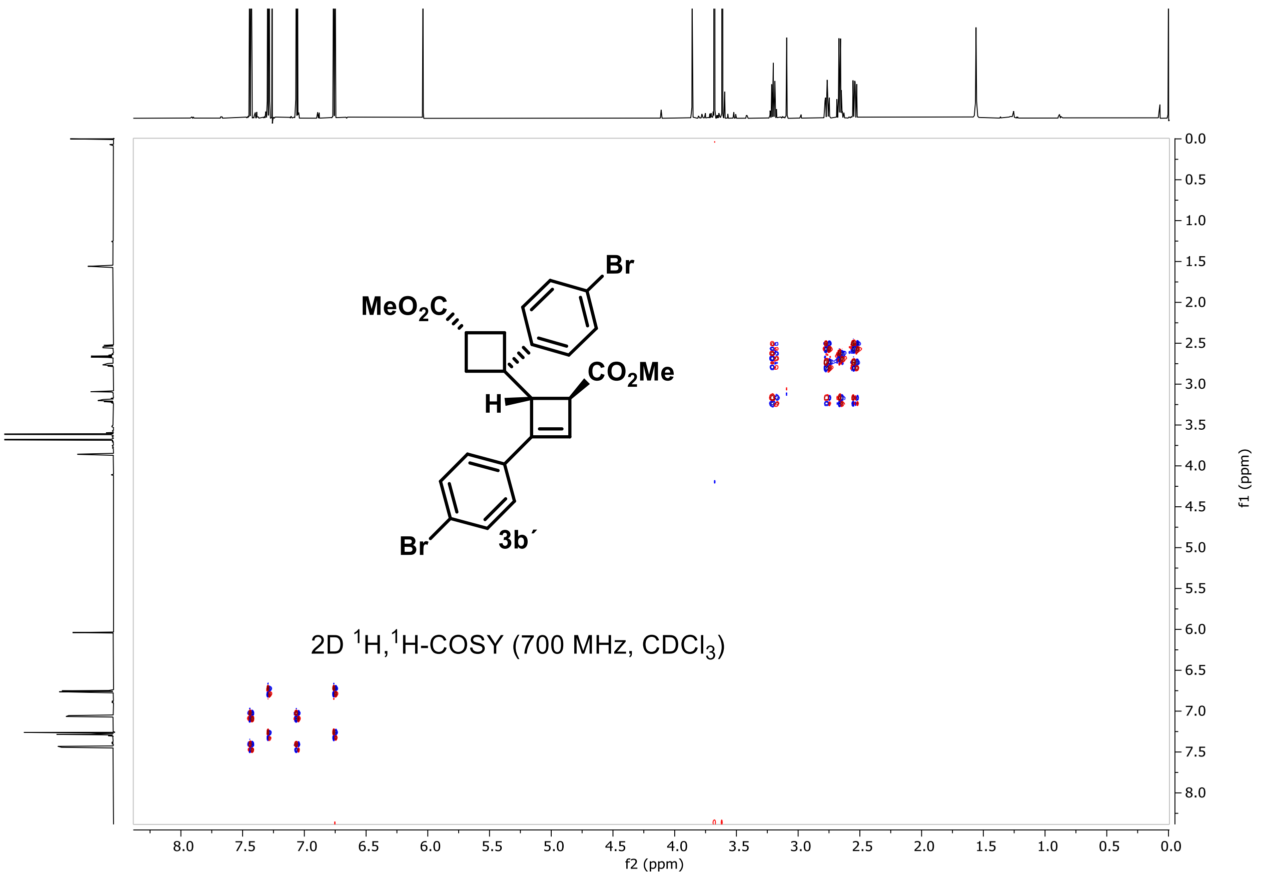
MeO2C
Br
CO2Me
H
Br
3b´
2D 1H,1H-COSY (700 MHz, CDCl3)
f2 (ppm)
f1 (ppm)

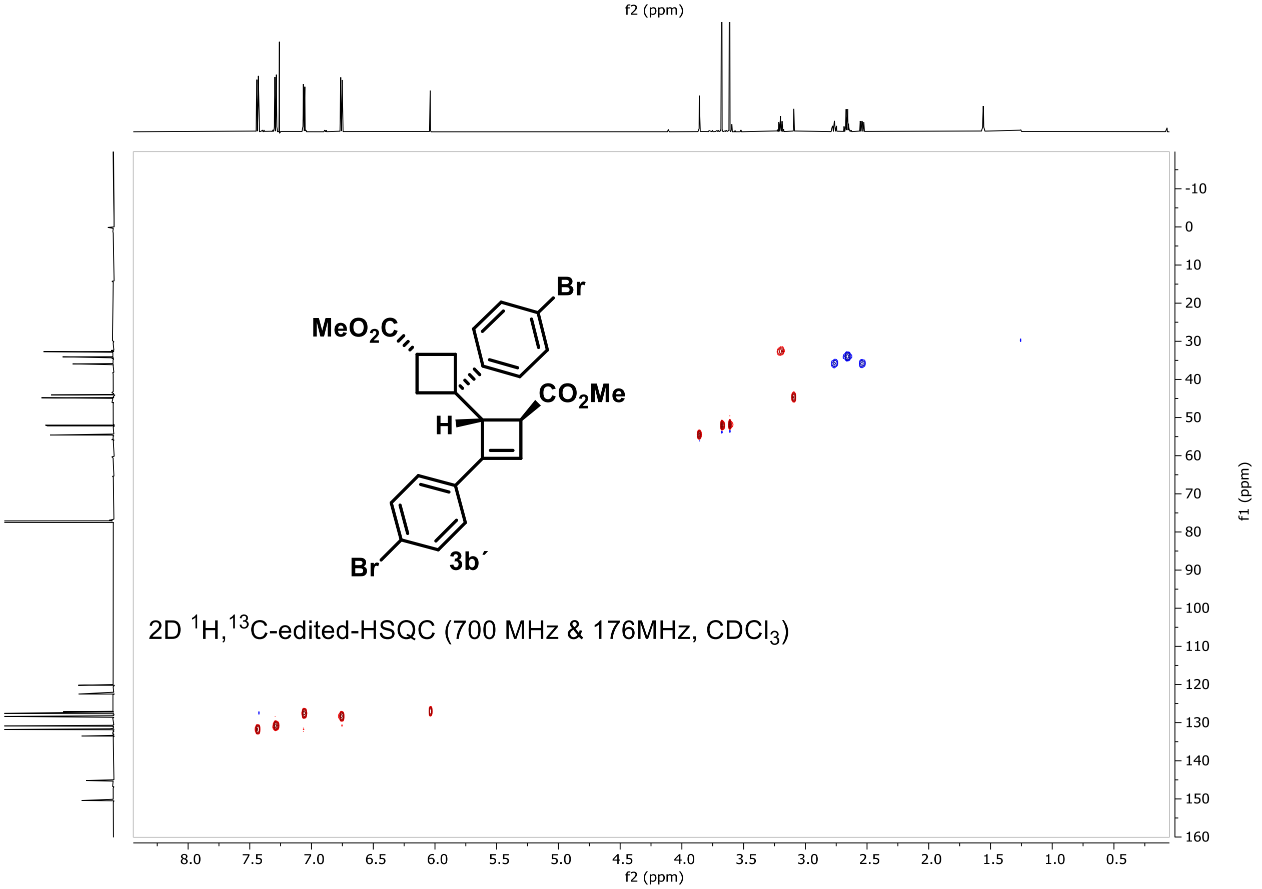
MeO2C
Br
CO2Me
H
Br
3b´
2D 1H,13C-edited-HSQC (700 MHz & 176MHz, CDCl3)
f2 (ppm)
f1 (ppm)

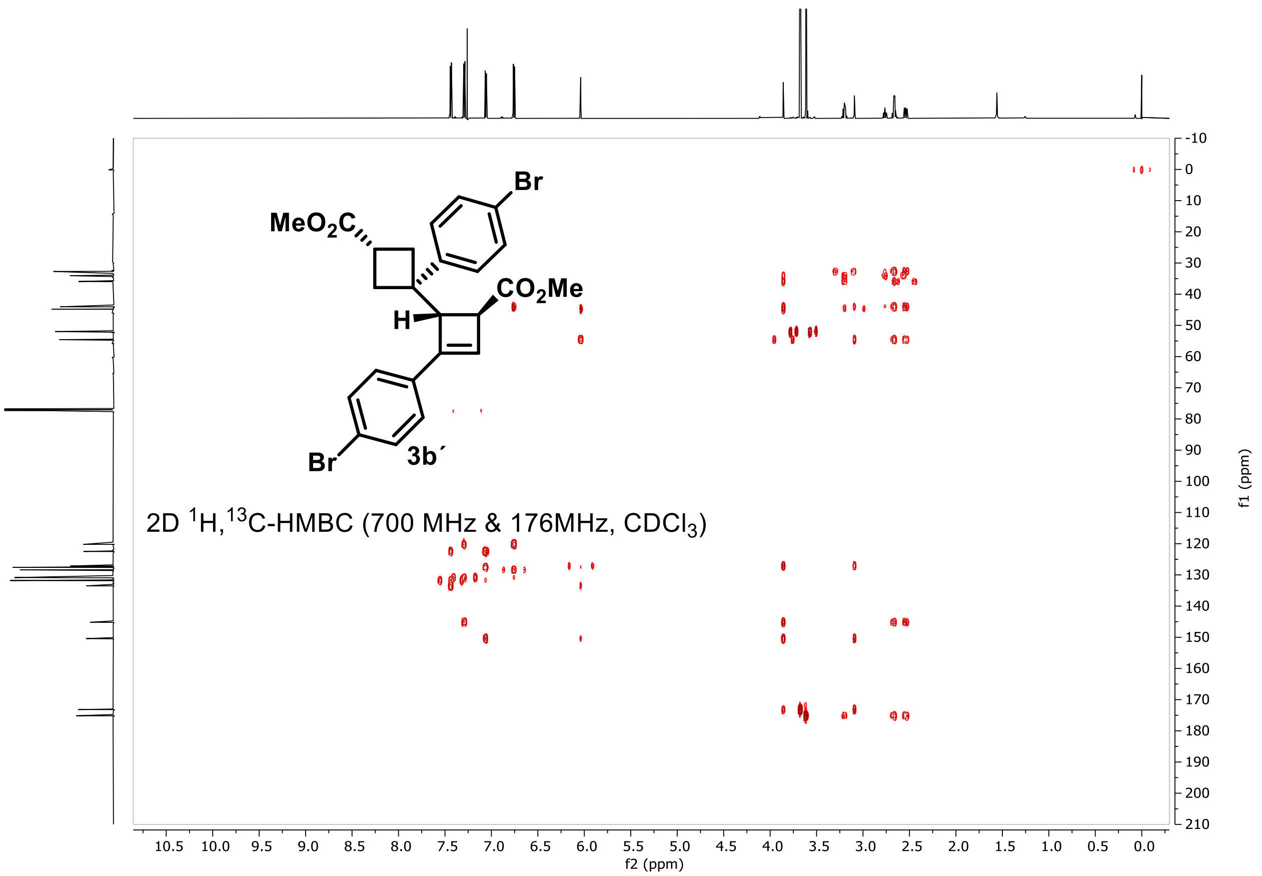
MeO2C
Br
CO2Me
H
Br
3b´
2D 1H,13C-HMBC (700 MHz & 176MHz, CDCl3)
f2 (ppm)
f1 (ppm)

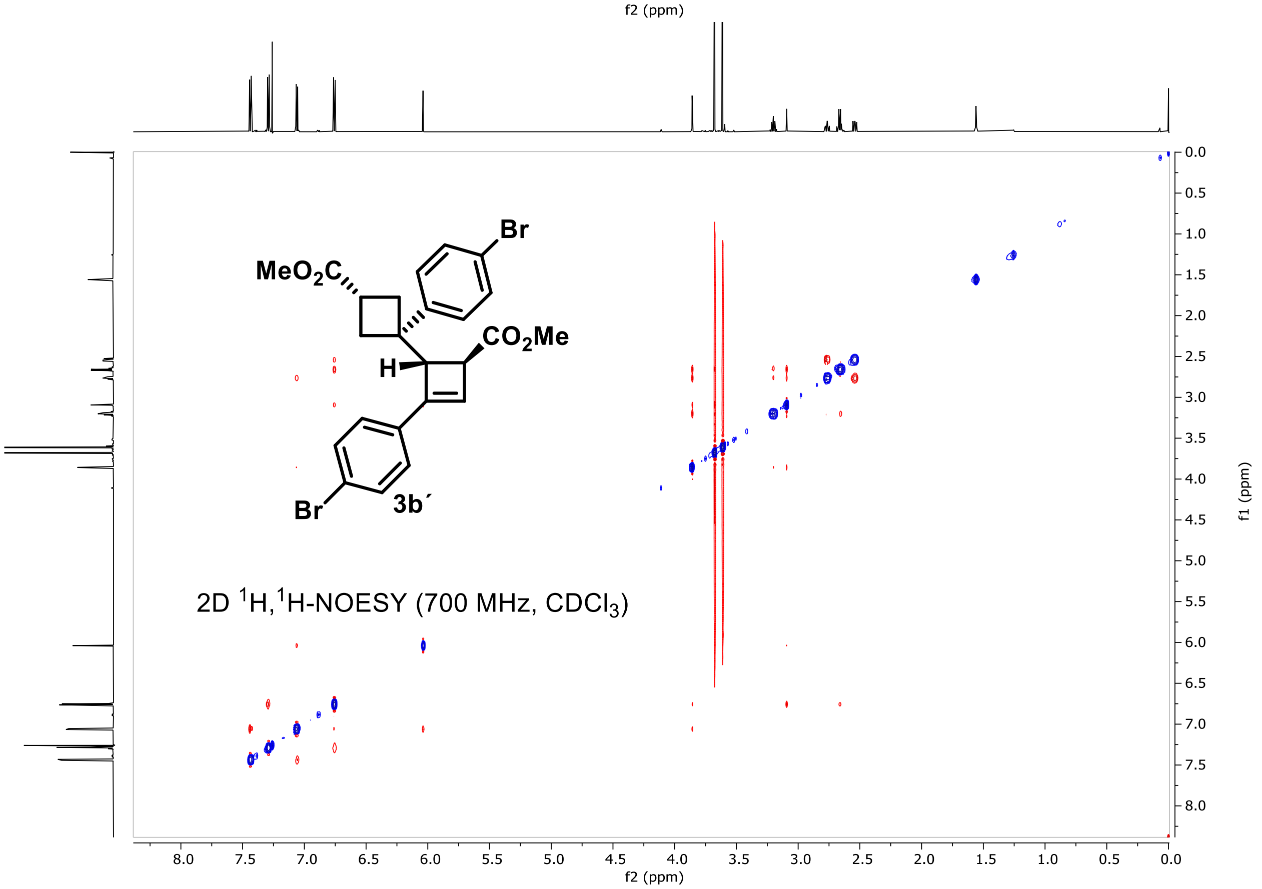
MeO2C
Br
CO2Me
H
Br
3b´
2D 1H,1H-NOESY (700 MHz, CDCl3)
f2 (ppm)
f1 (ppm)

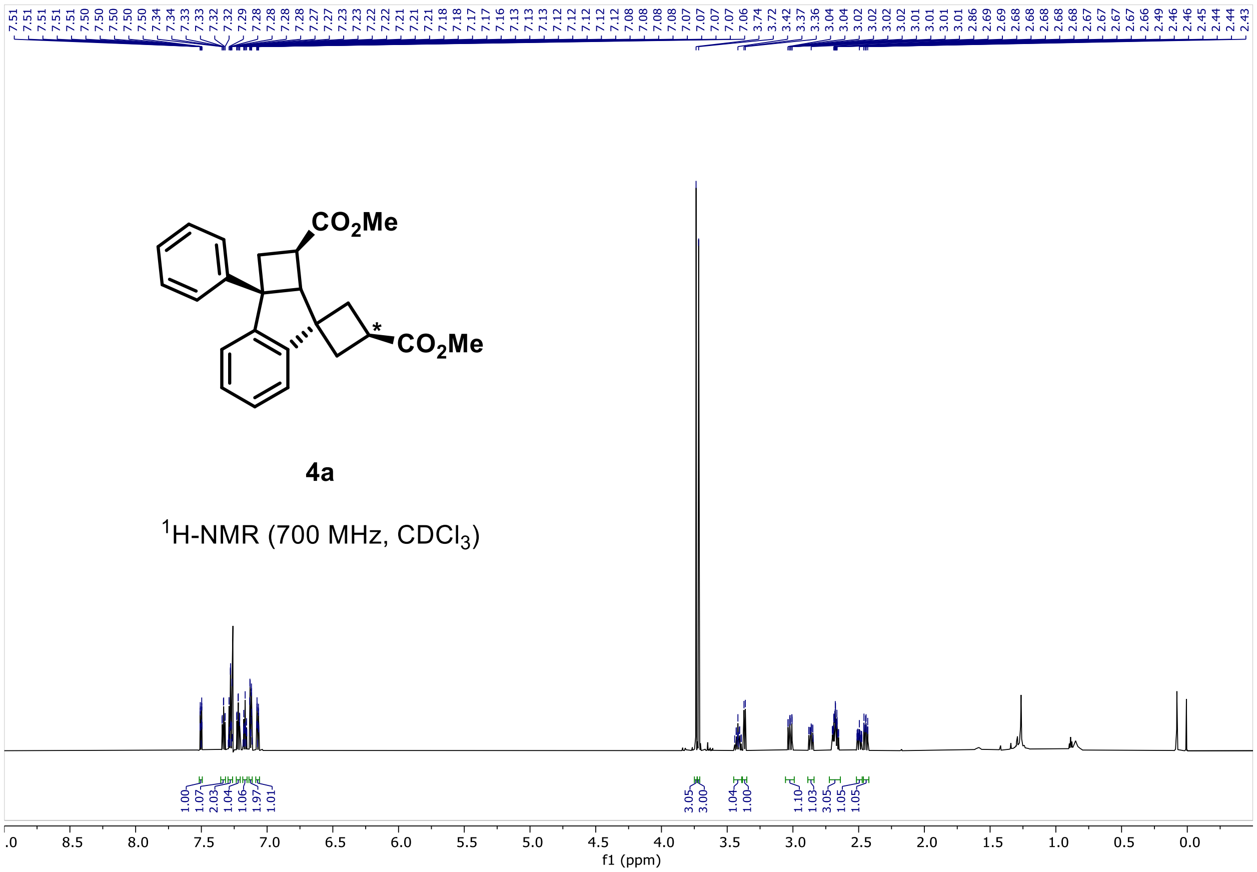
CO2Me
CO2Me
4a
1H-NMR (700 MHz, CDCl3)
f1 (ppm)

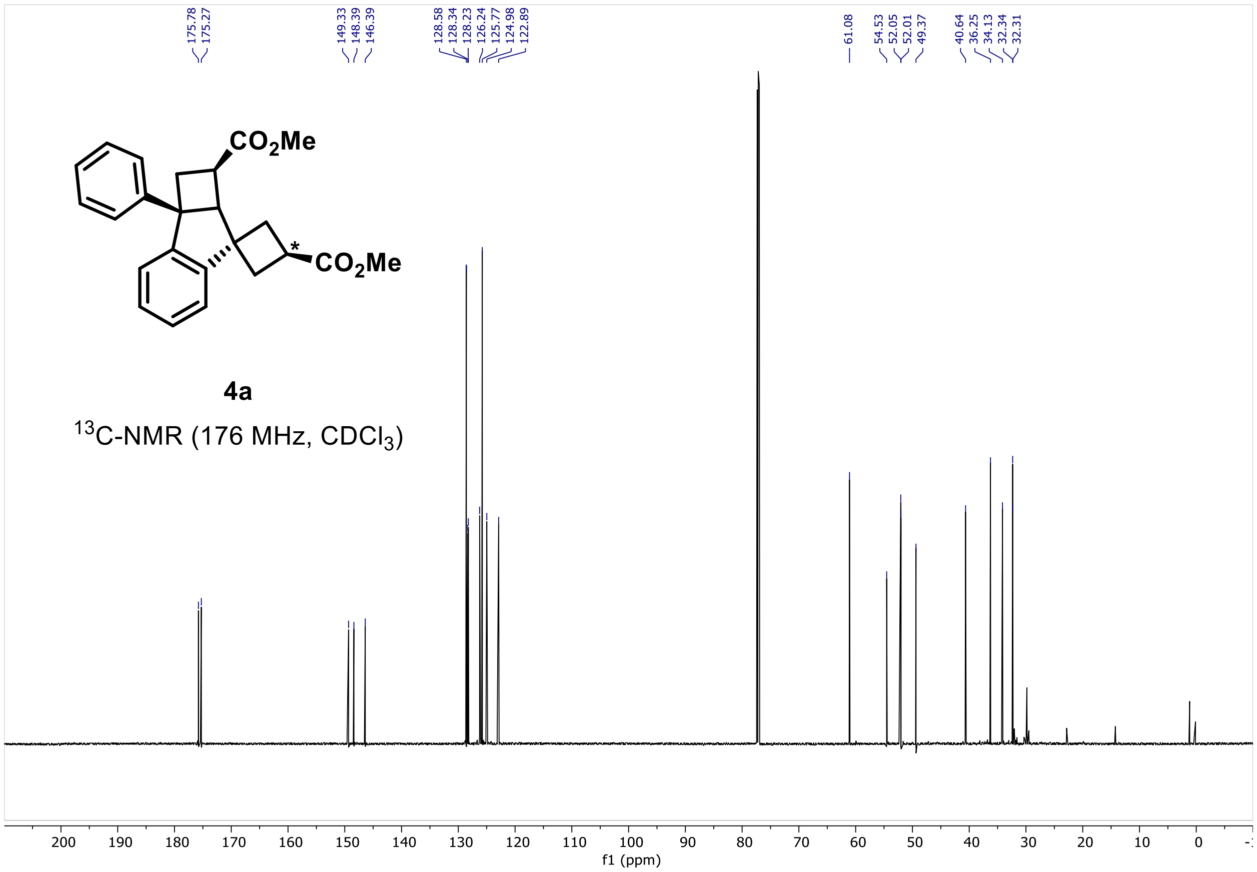
175.78
175.27
149.33
148.39
146.39
128.58
128.34
128.23
126.24
125.77
124.98
122.89
61.08
54.53
52.05
52.01
49.37
40.64
36.25
34.13
32.34
32.31
CO2Me
CO2Me
4a
13C-NMR (176 MHz, CDCl3)
f1 (ppm)

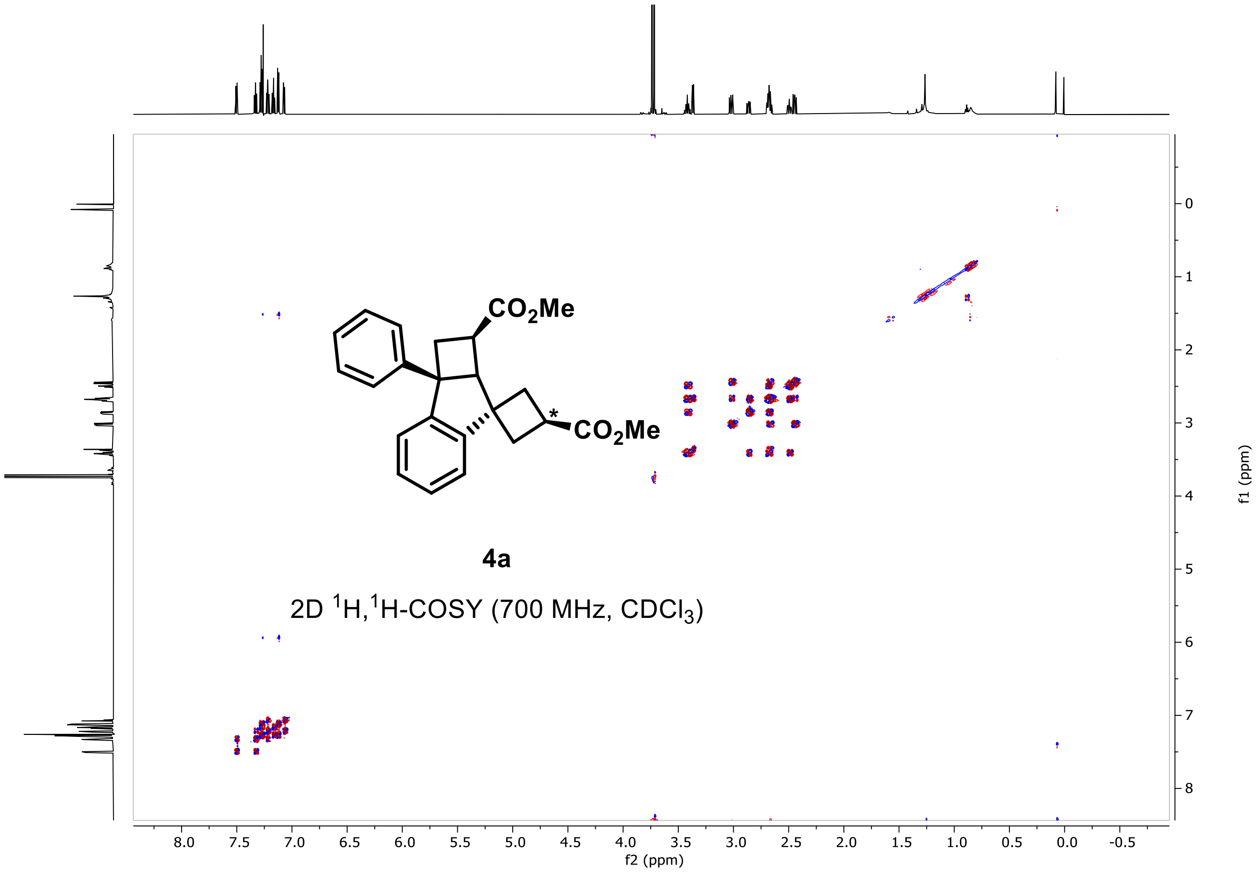
CO2Me
*
CO2Me
4a
2D 1H,1H-COSY (700 MHz, CDCl3)
f2 (ppm)
f1 (ppm)

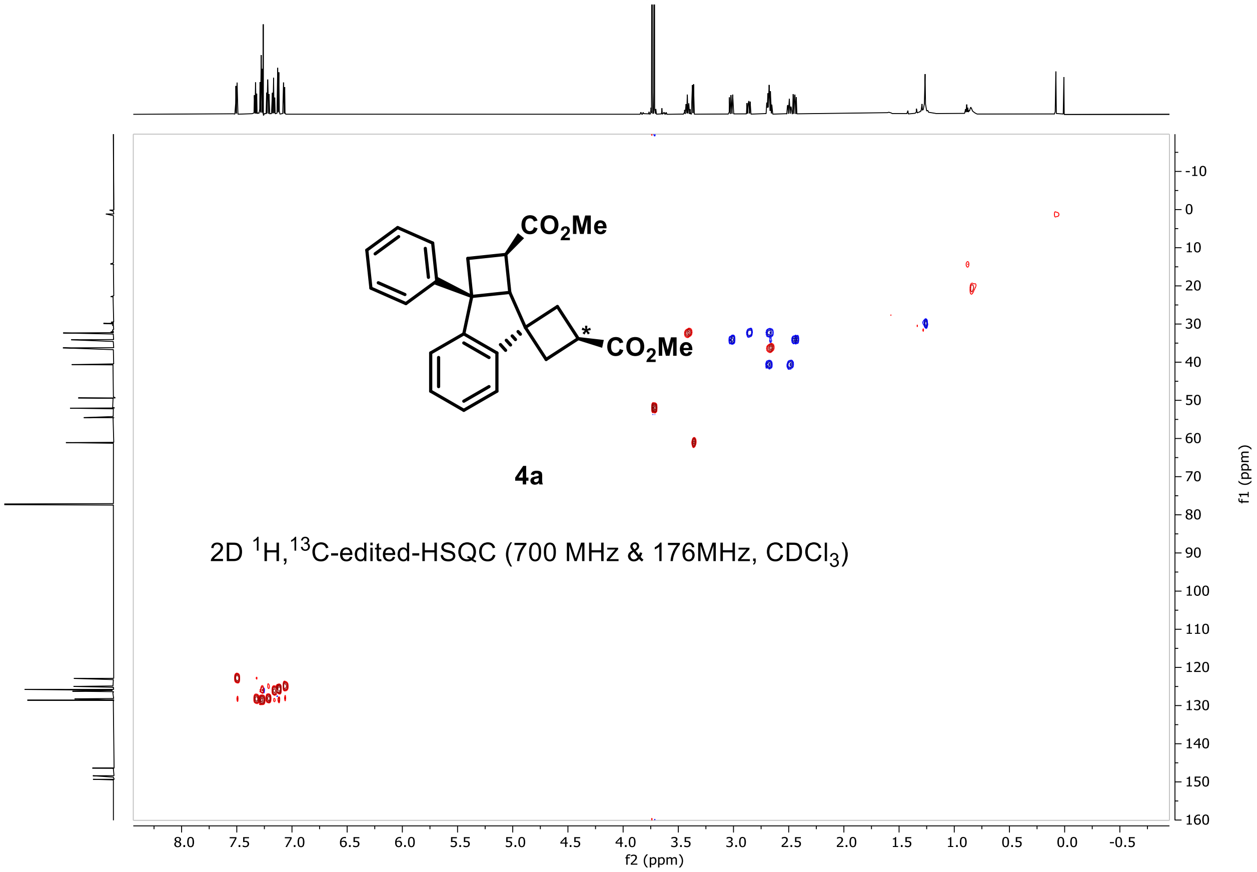
CO2Me
*
CO2Me
4a
2D 1H,13C-edited-HSQC (700 MHz & 176MHz, CDCl3)
f2 (ppm)
f1 (ppm)

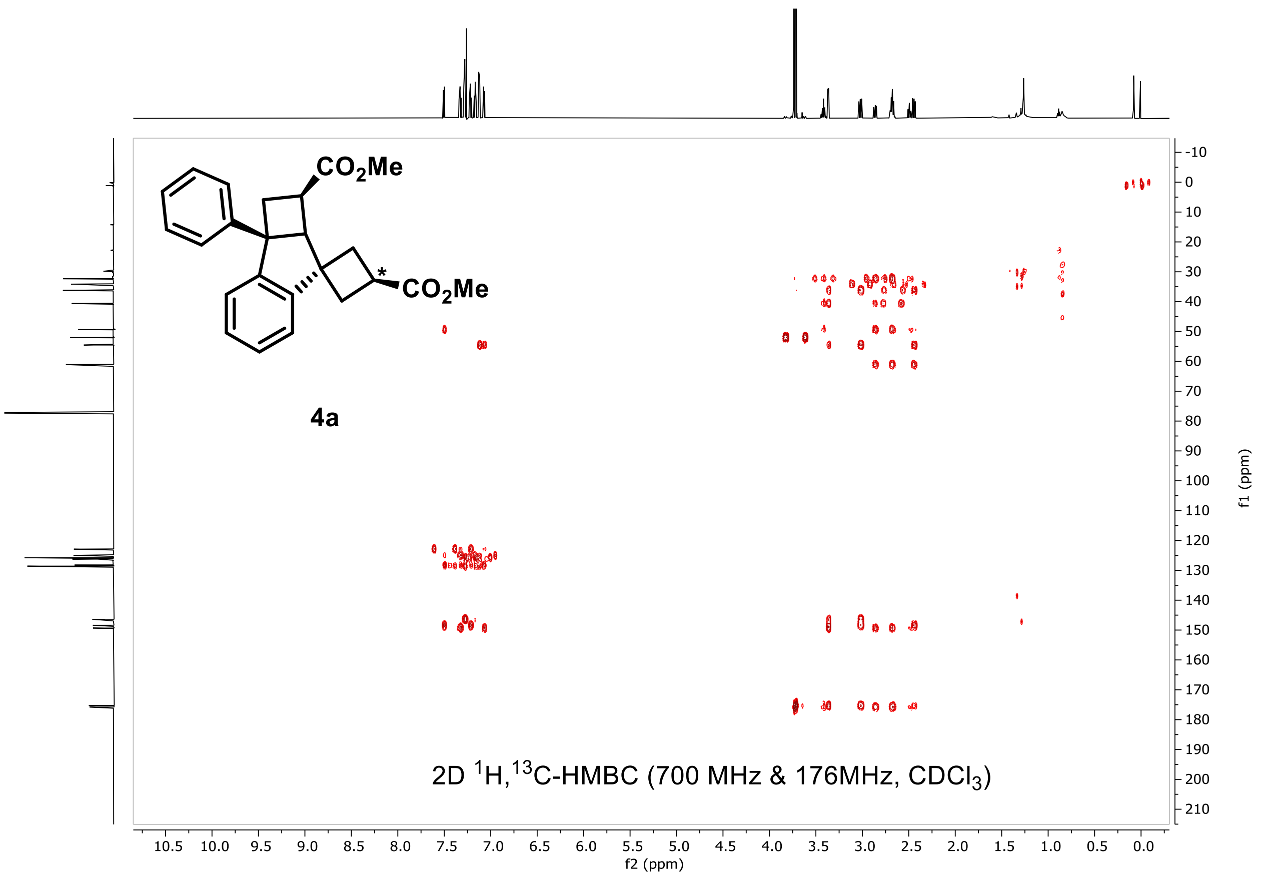

CO2Me
CO2Me
4a
2D 1H,13C-HMBC (700 MHz & 176MHz, CDCl3)
f1 (ppm)
f2 (ppm)

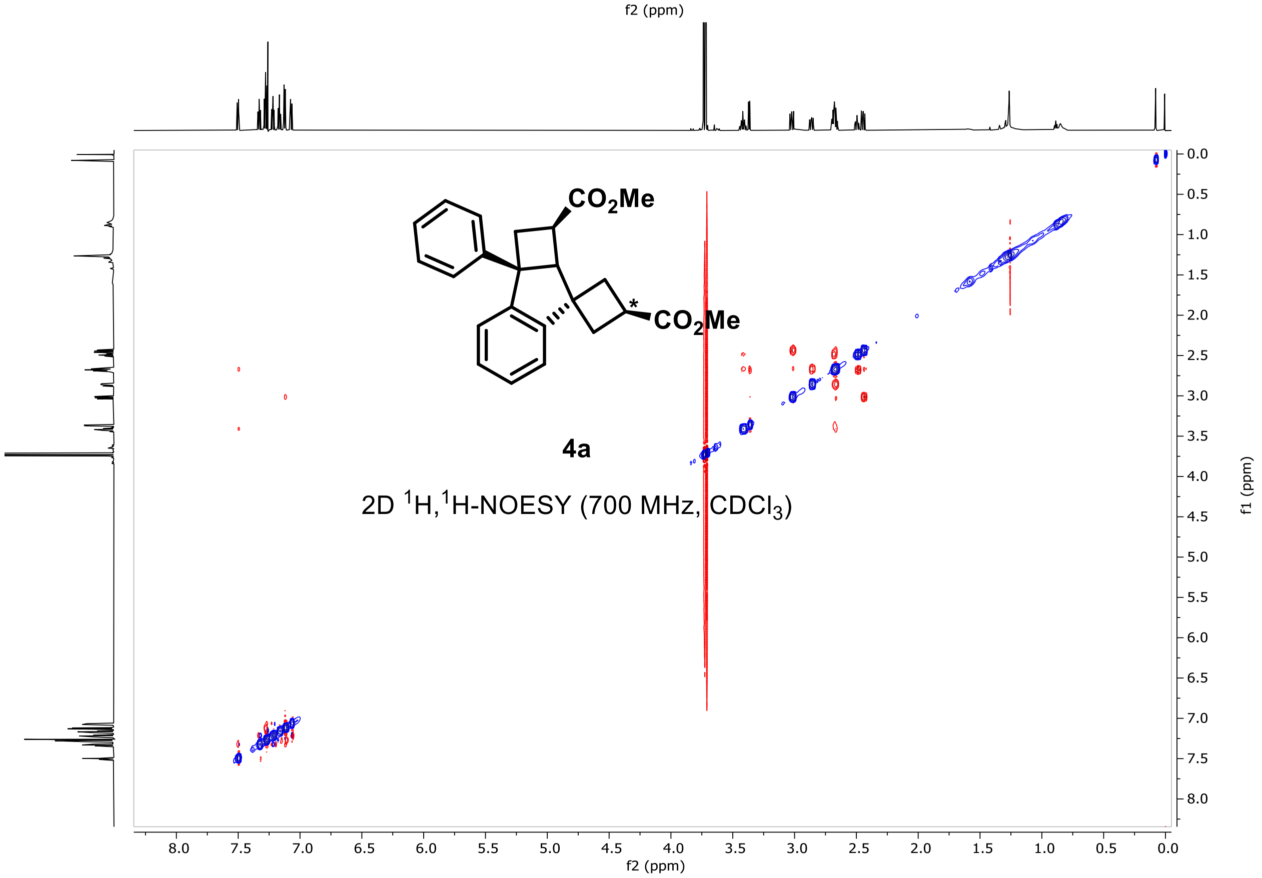

CO2Me
CO2Me
4a
2D 1H,1H-NOESY (700 MHz, CDCl3)
f1 (ppm)
f2 (ppm)

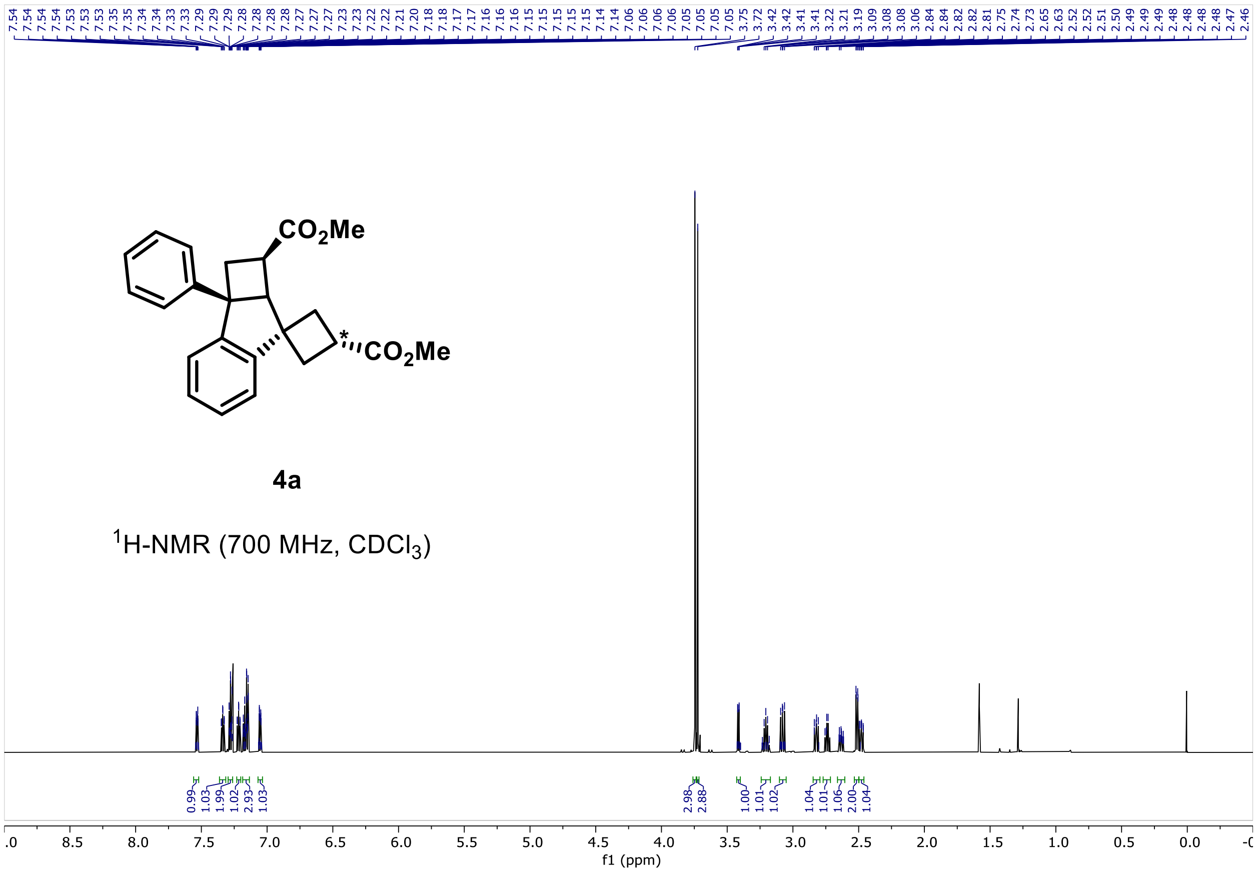

CO2Me
CO2Me
4a
1H-NMR (700 MHz, CDCl3)
f1 (ppm)

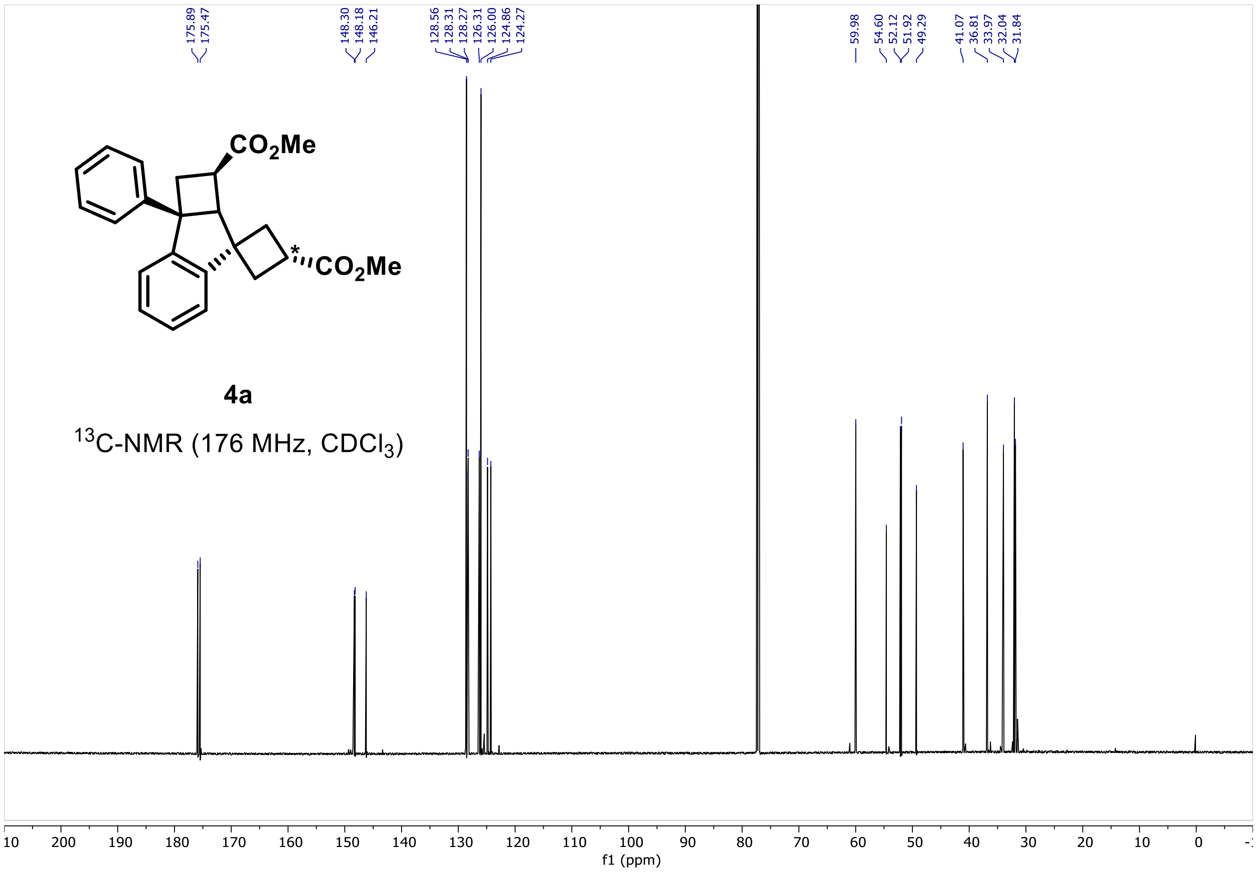

175.89
175.47
148.30
148.18
146.21
128.56
128.31
128.27
126.31
126.00
124.86
124.27
59.98
54.60
52.12
51.92
49.29
41.07
36.81
33.97
32.04
31.84
CO2Me
CO2Me
4a
13C-NMR (176 MHz, CDCl3)
f1 (ppm)

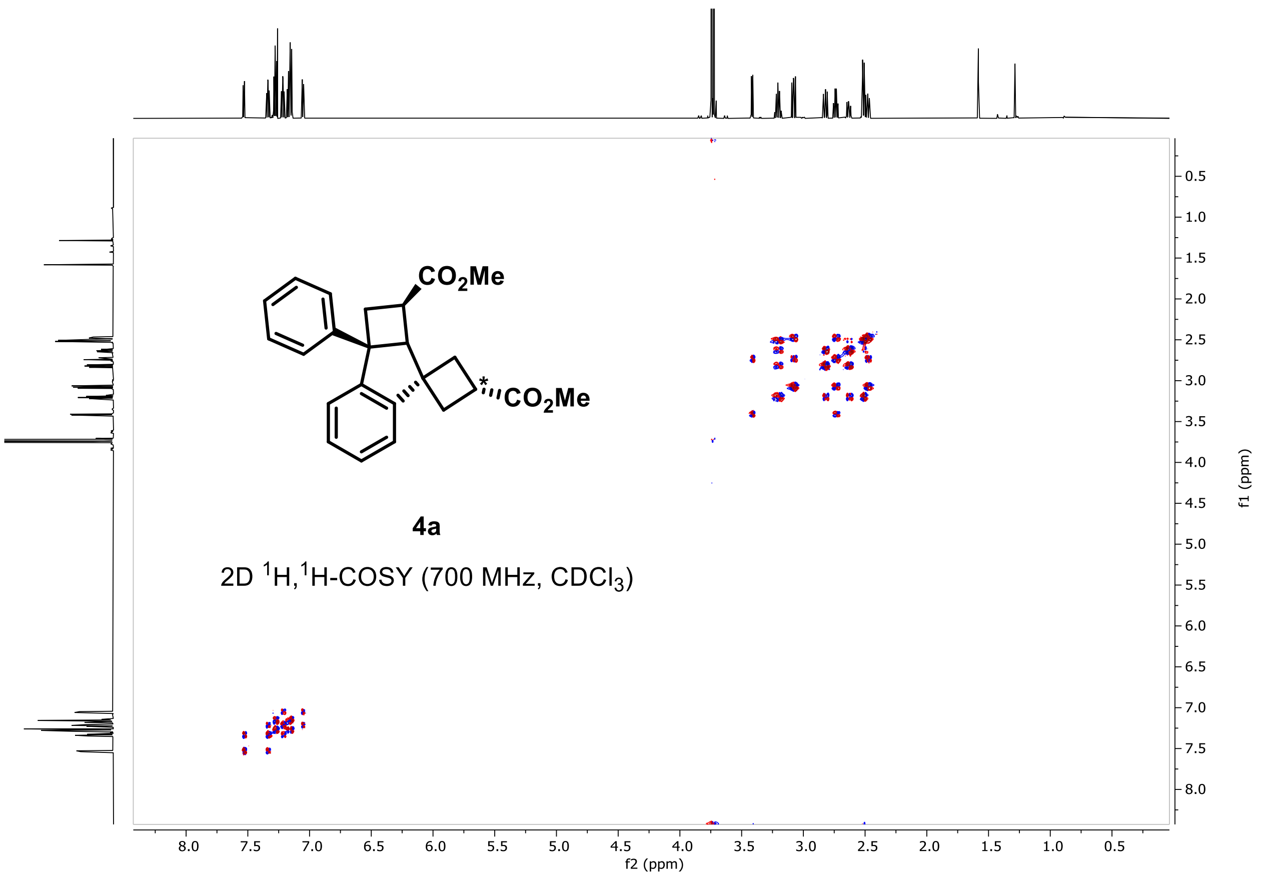

CO2Me
CO2Me
4a
2D 1H,1H-COSY (700 MHz, CDCl3)
f1 (ppm)
f2 (ppm)

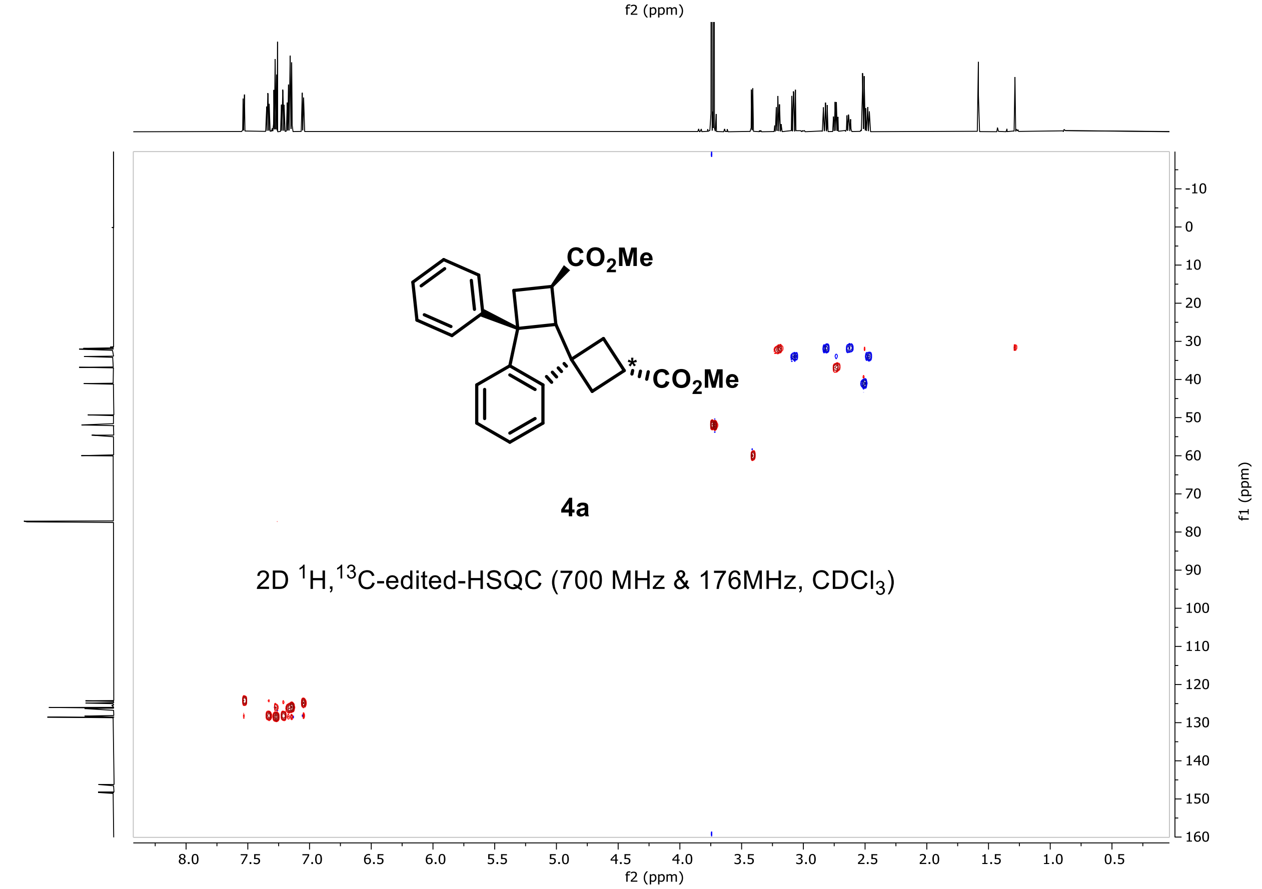

CO2Me
CO2Me
4a
2D 1H,13C-edited-HSQC (700 MHz & 176MHz, CDCl3)
f1 (ppm)
f2 (ppm)

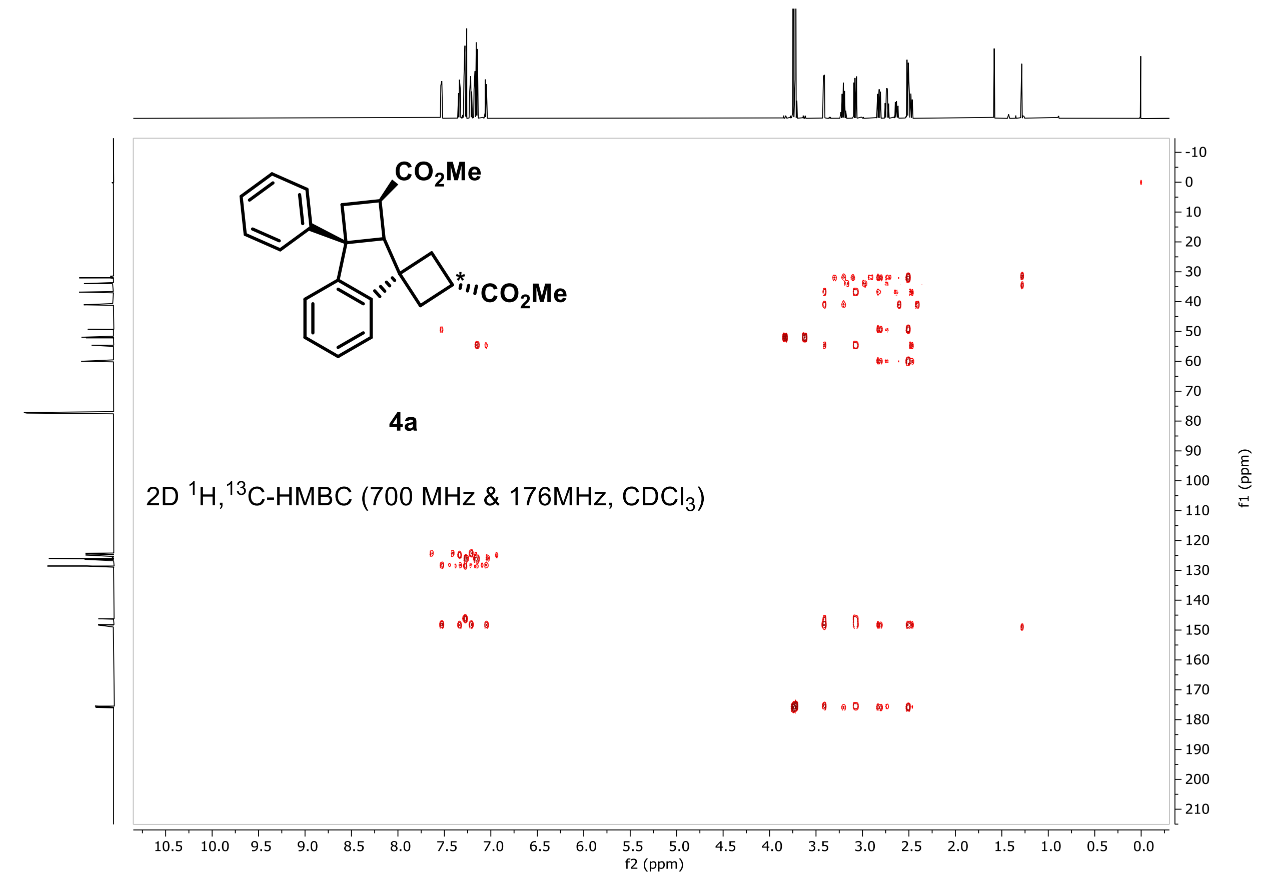
CO2Me
CO2Me
4a
2D 1H,13C-HMBC (700 MHz & 176MHz, CDCl3)
f1 (ppm)
f2 (ppm)

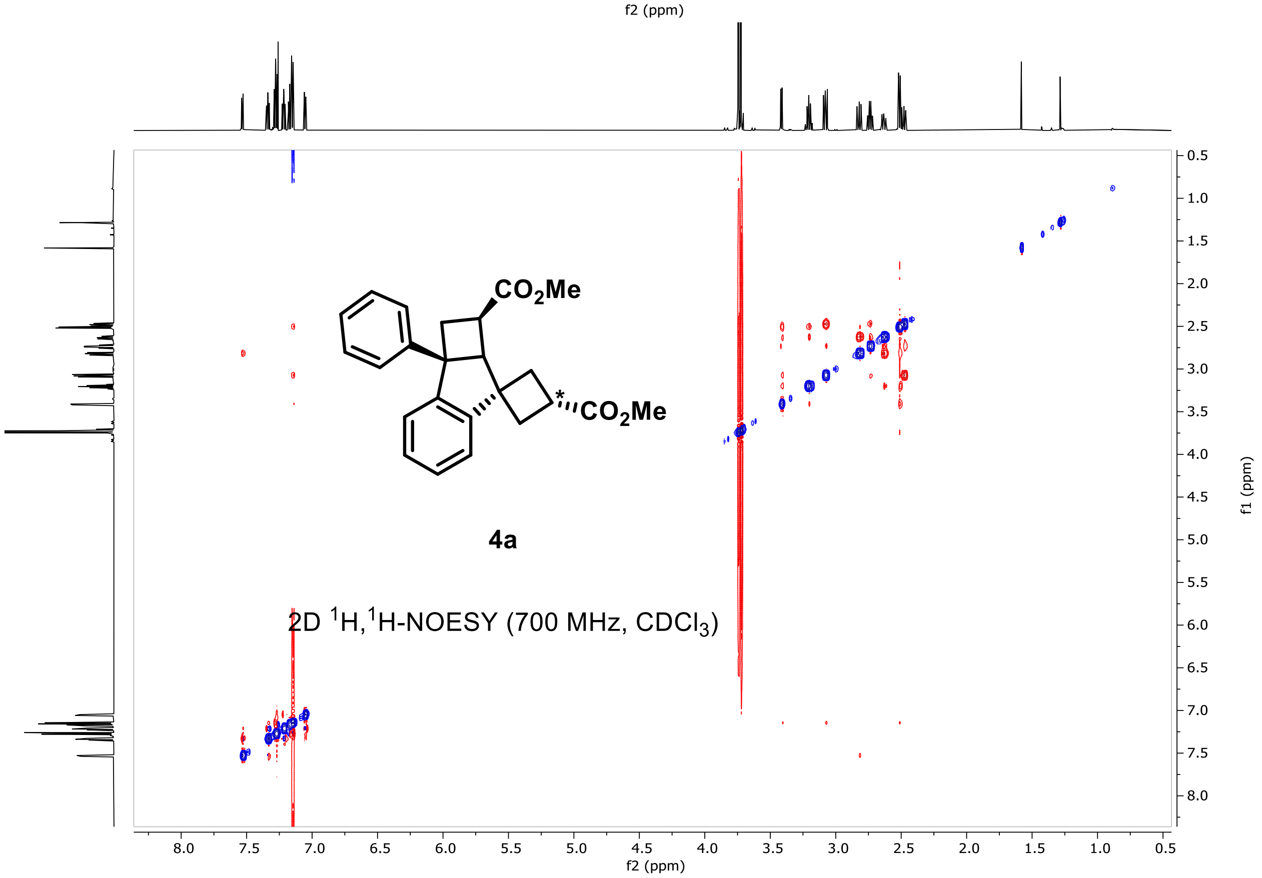
CO2Me
CO2Me
4a
2D 1H,1H-NOESY (700 MHz, CDCl3)
f1 (ppm)
f2 (ppm)

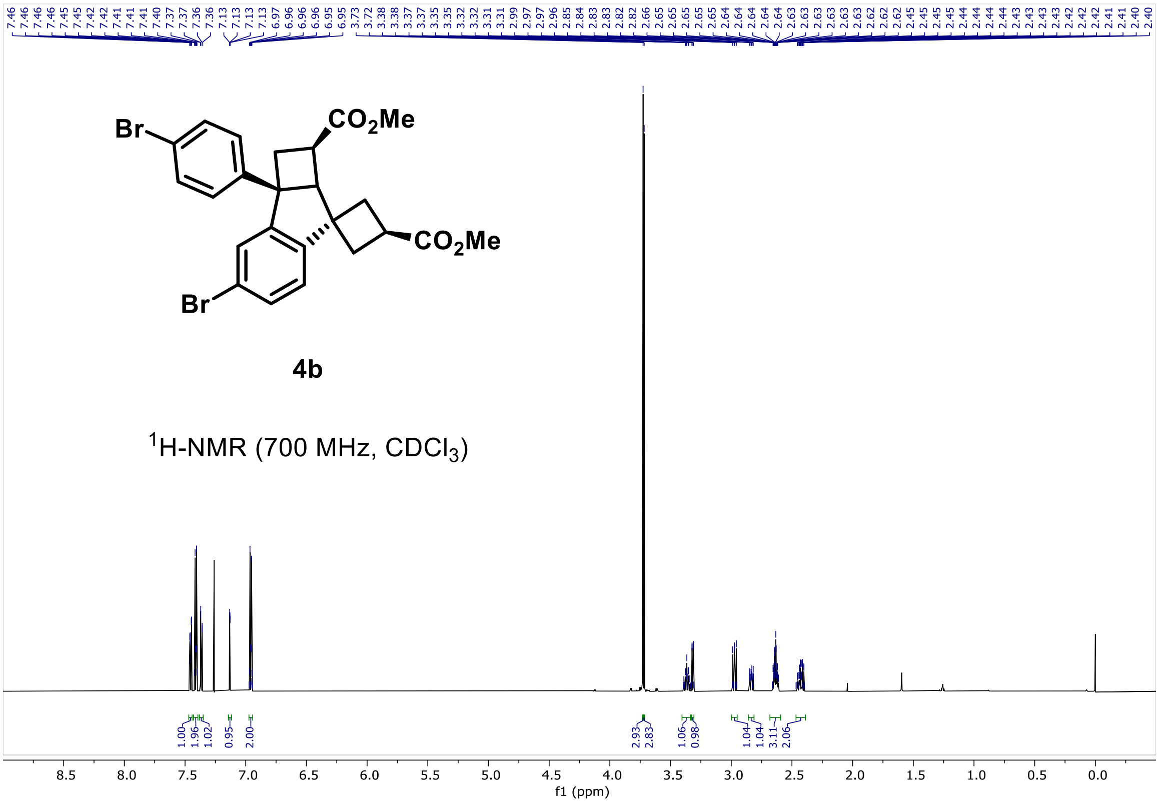
Br
CO2Me
CO2Me
Br
4b
1H-NMR (700 MHz, CDCl3)
f1 (ppm)

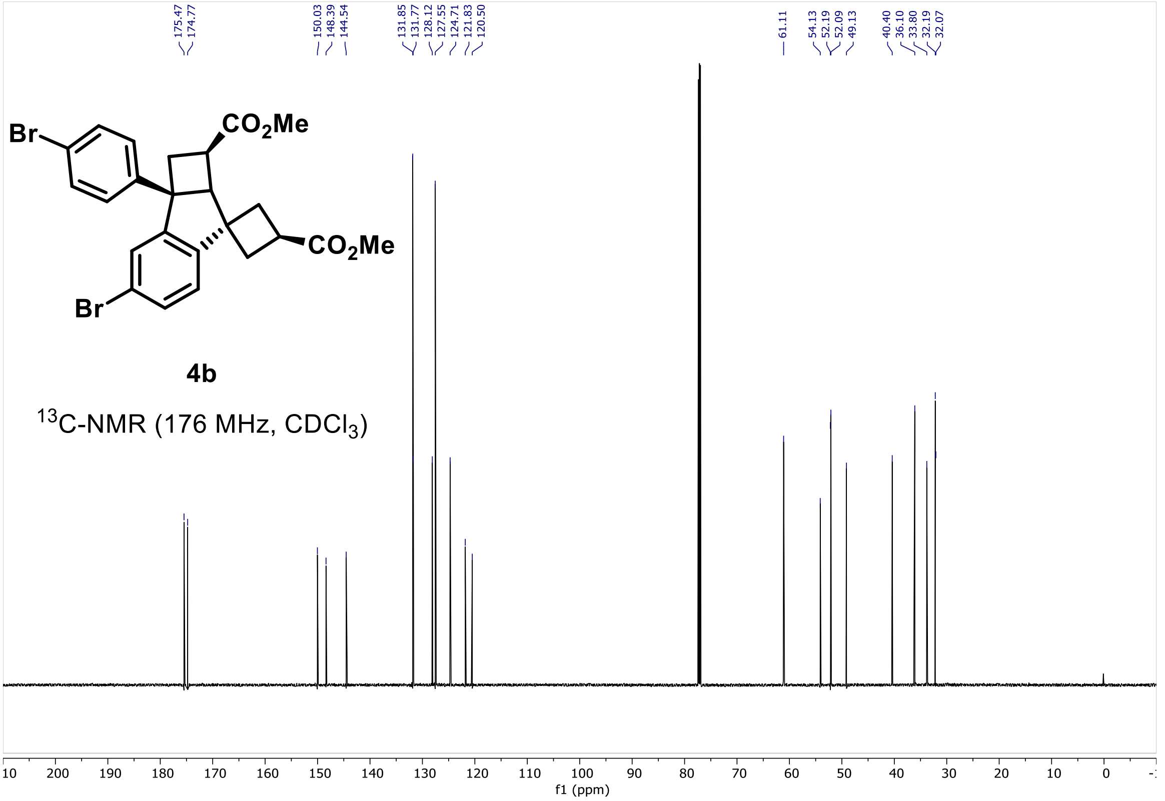
175.47
174.77
150.03
148.39
144.54
131.85
131.77
128.12
127.55
124.71
121.83
120.50
61.11
54.13
52.19
52.09
49.13
40.40
36.10
33.80
32.19
32.07
Br
CO2Me
CO2Me
Br
4b
13C-NMR (176 MHz, CDCl3)
f1 (ppm)

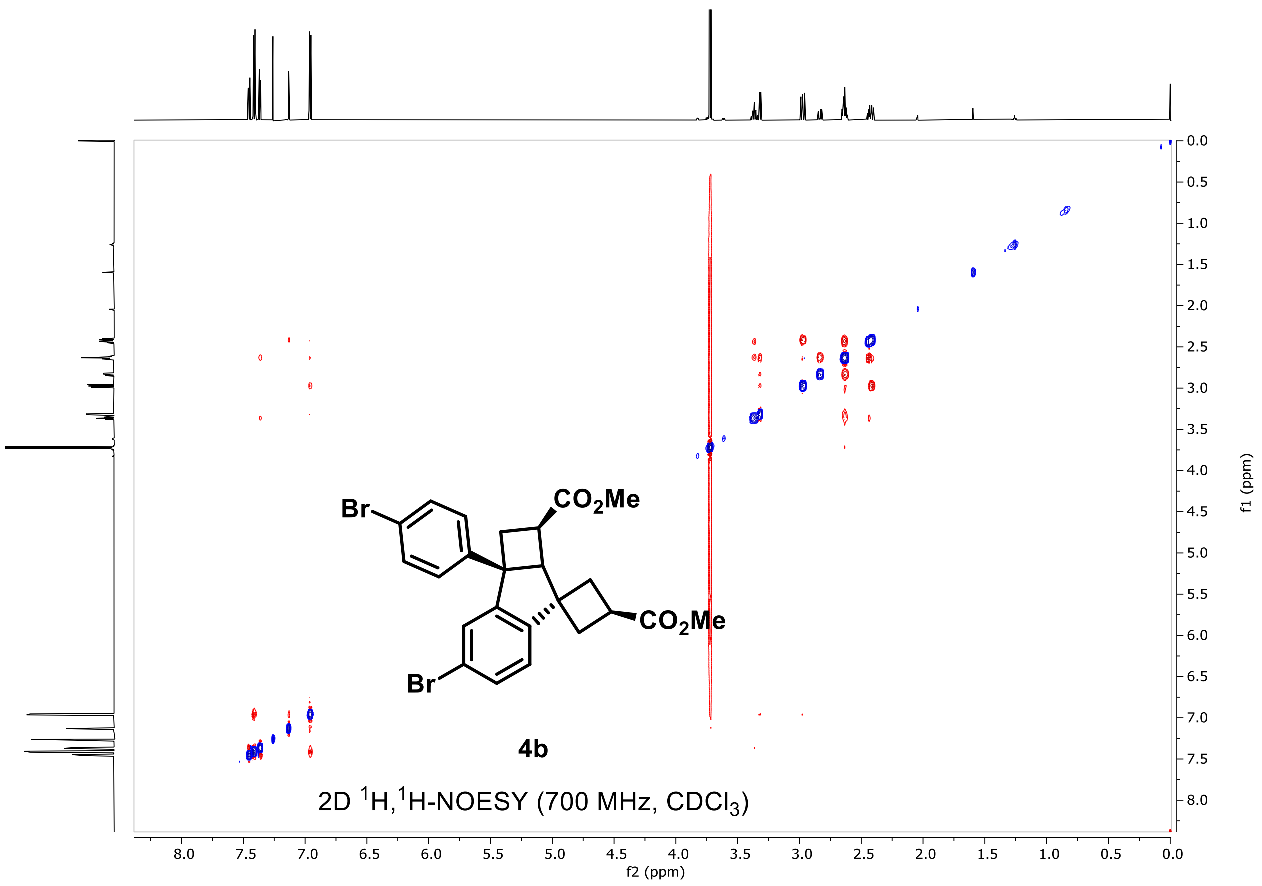
Br
CO2Me
CO2Me
Br
4b
2D 1H,1H-NOESY (700 MHz, CDCl3)
f2 (ppm)
f1 (ppm)

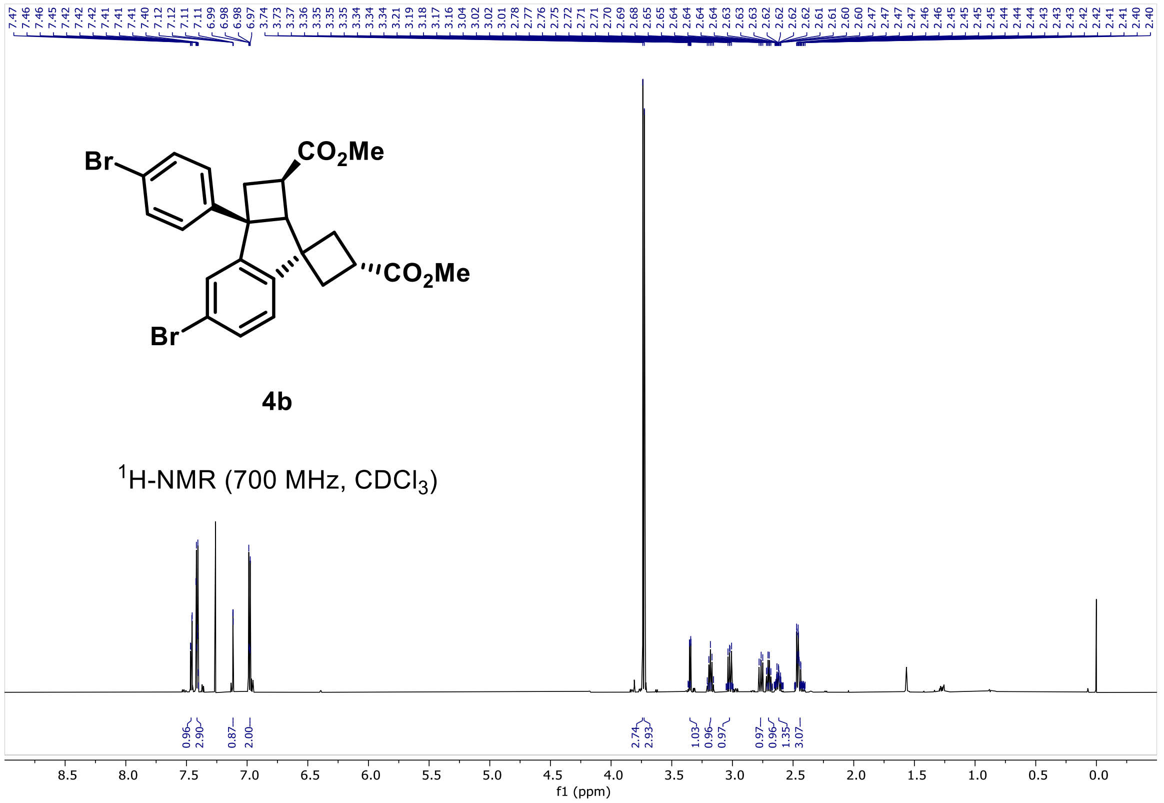
4b
^{1}H-NMR (700 MHz, $CDCl_3$)
f1 (ppm)

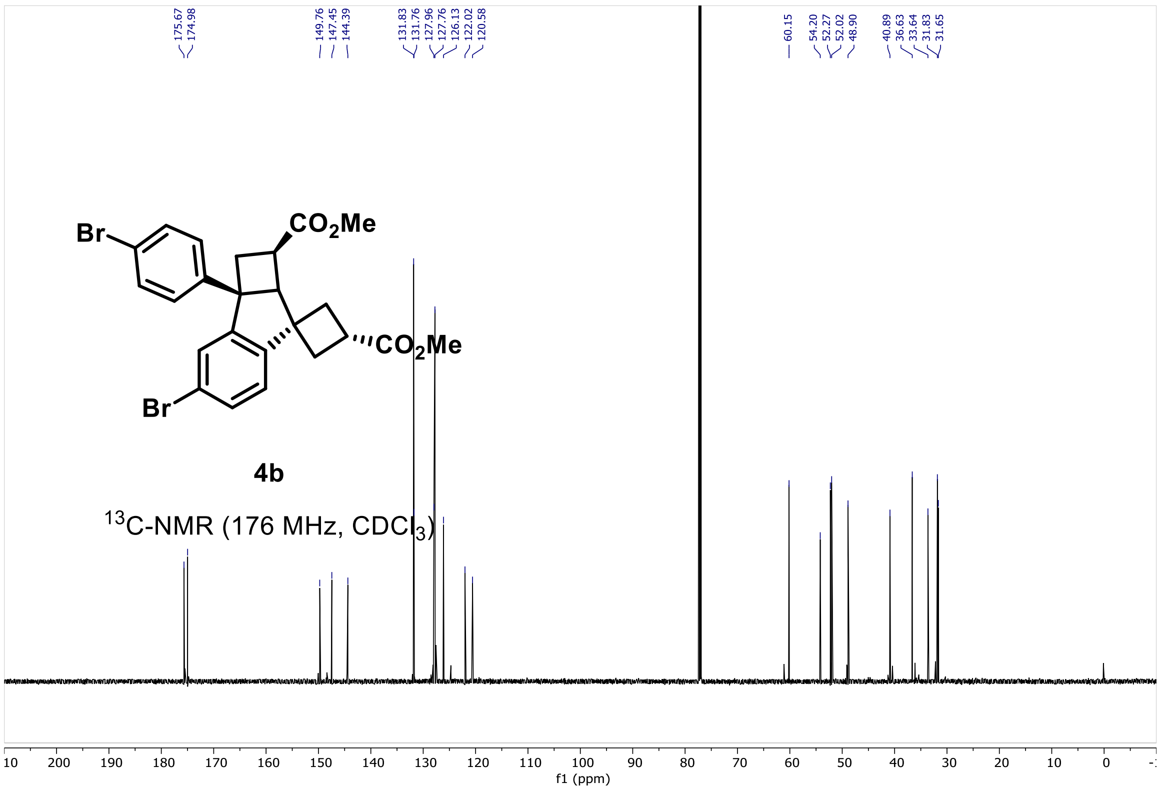
4b
^{13}C-NMR (176 MHz, $CDCl_3$)
f1 (ppm)

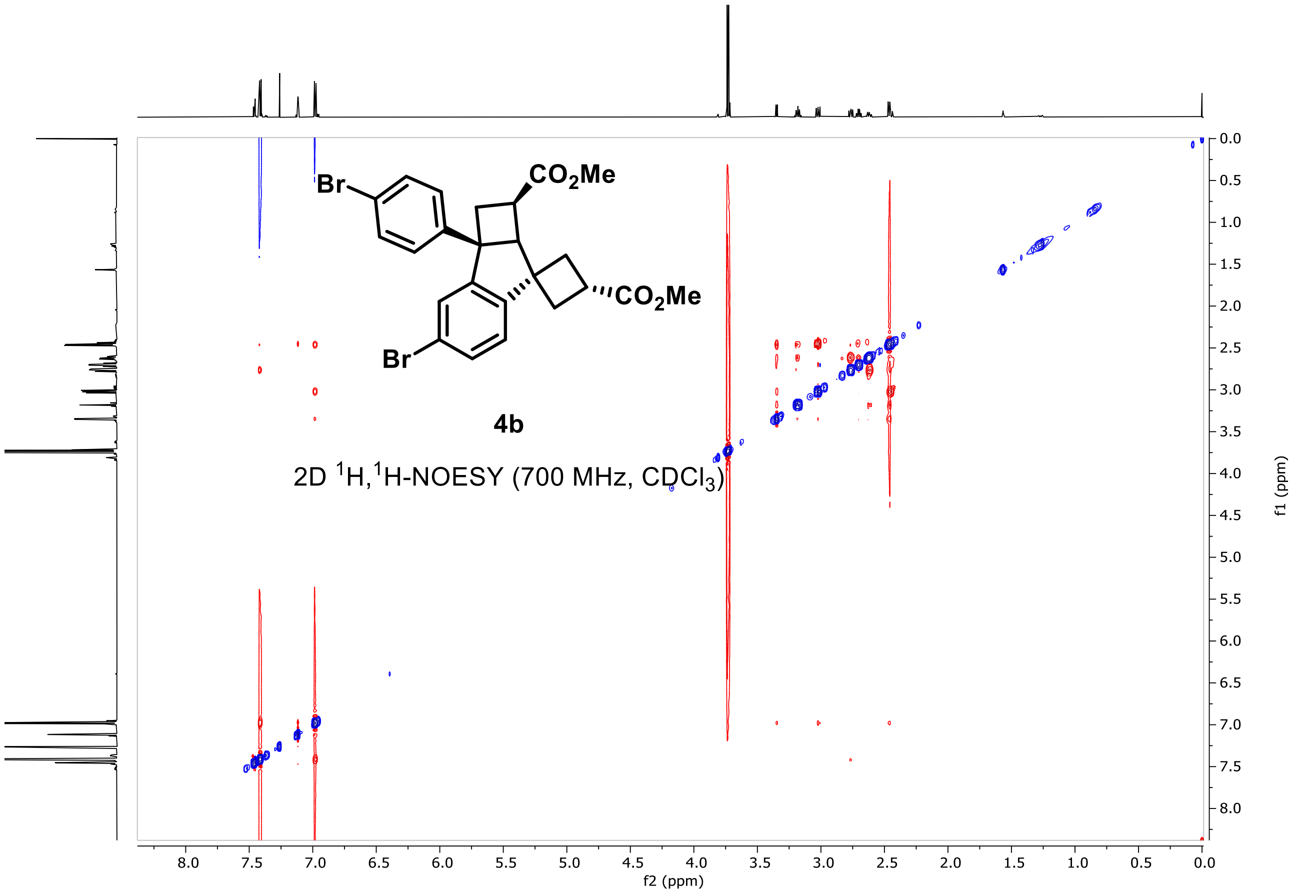
Br
CO2Me
CO2Me
Br
4b
2D 1H,1H-NOESY (700 MHz, CDCl3)
f1 (ppm)
f2 (ppm)

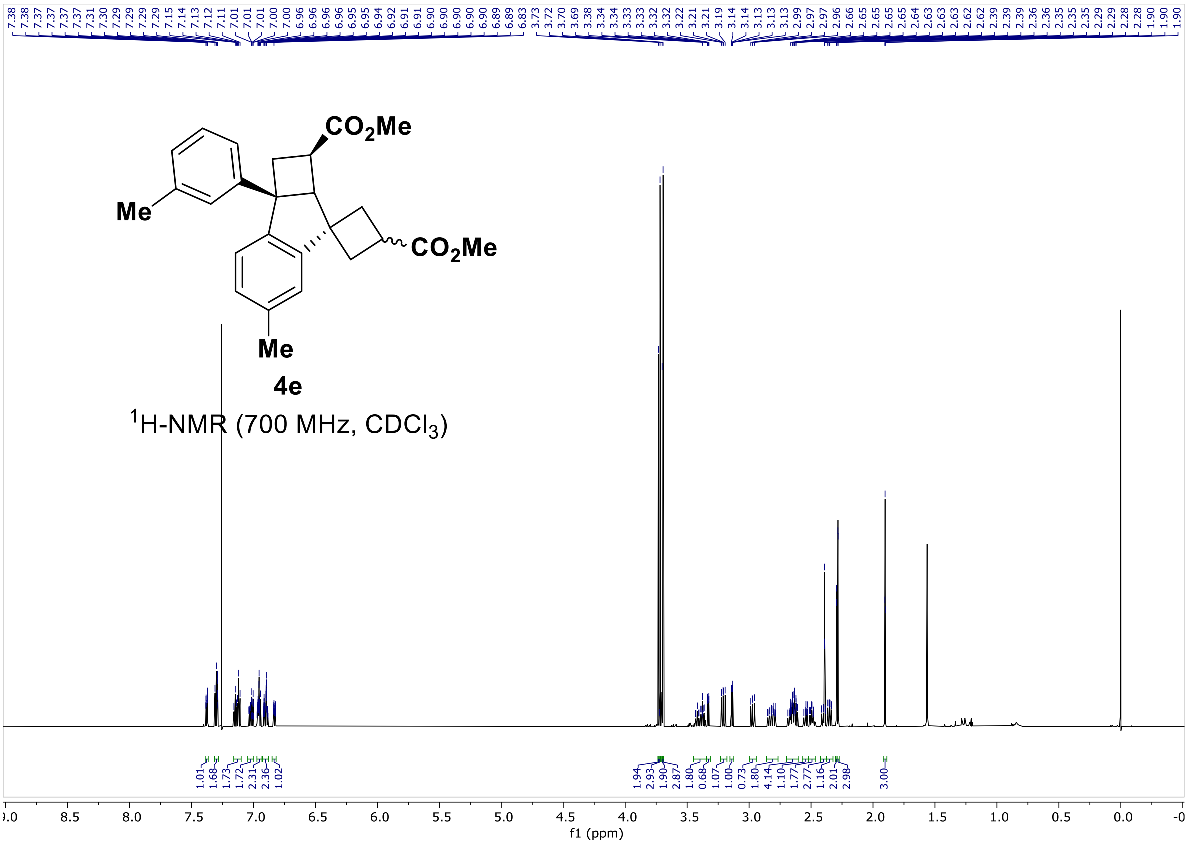
CO2Me
Me
CO2Me
Me
4e
1H-NMR (700 MHz, CDCl3)
f1 (ppm)

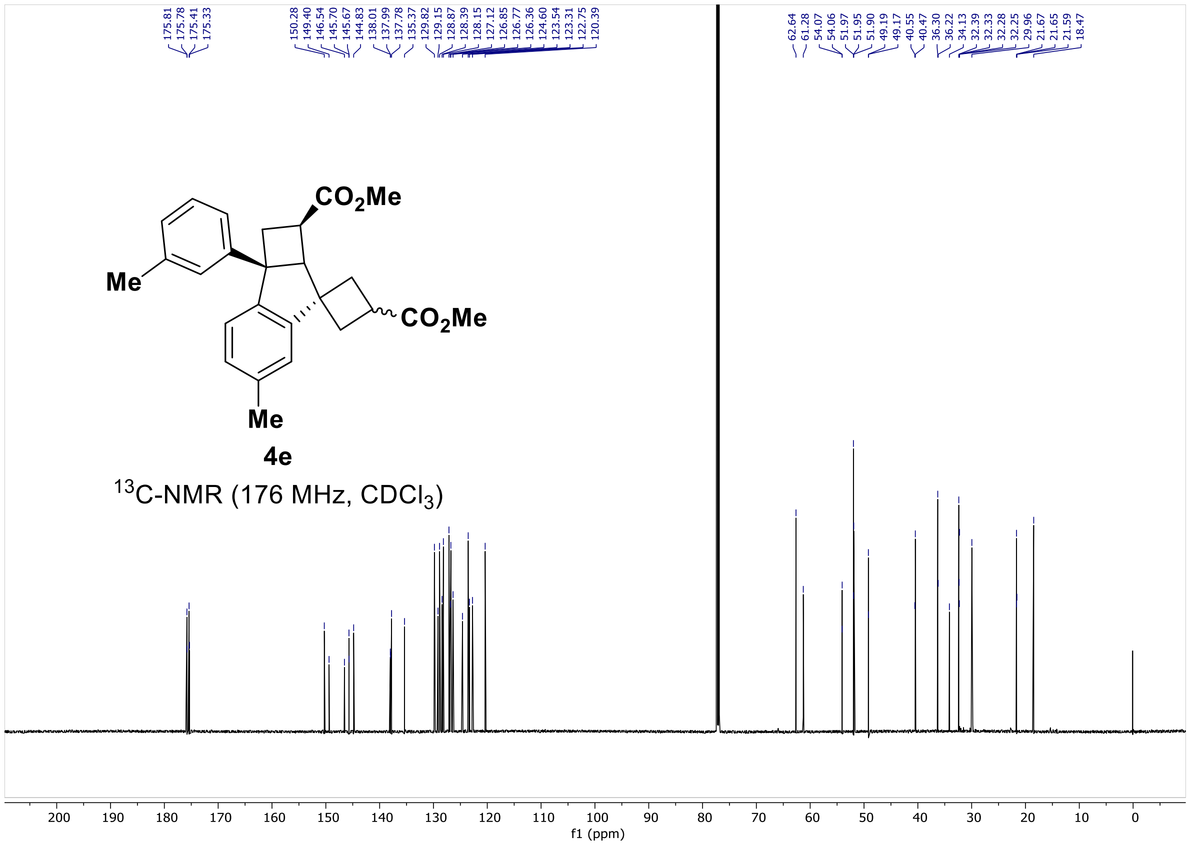
CO2Me
Me
CO2Me
Me
4e
13C-NMR (176 MHz, CDCl3)
f1 (ppm)

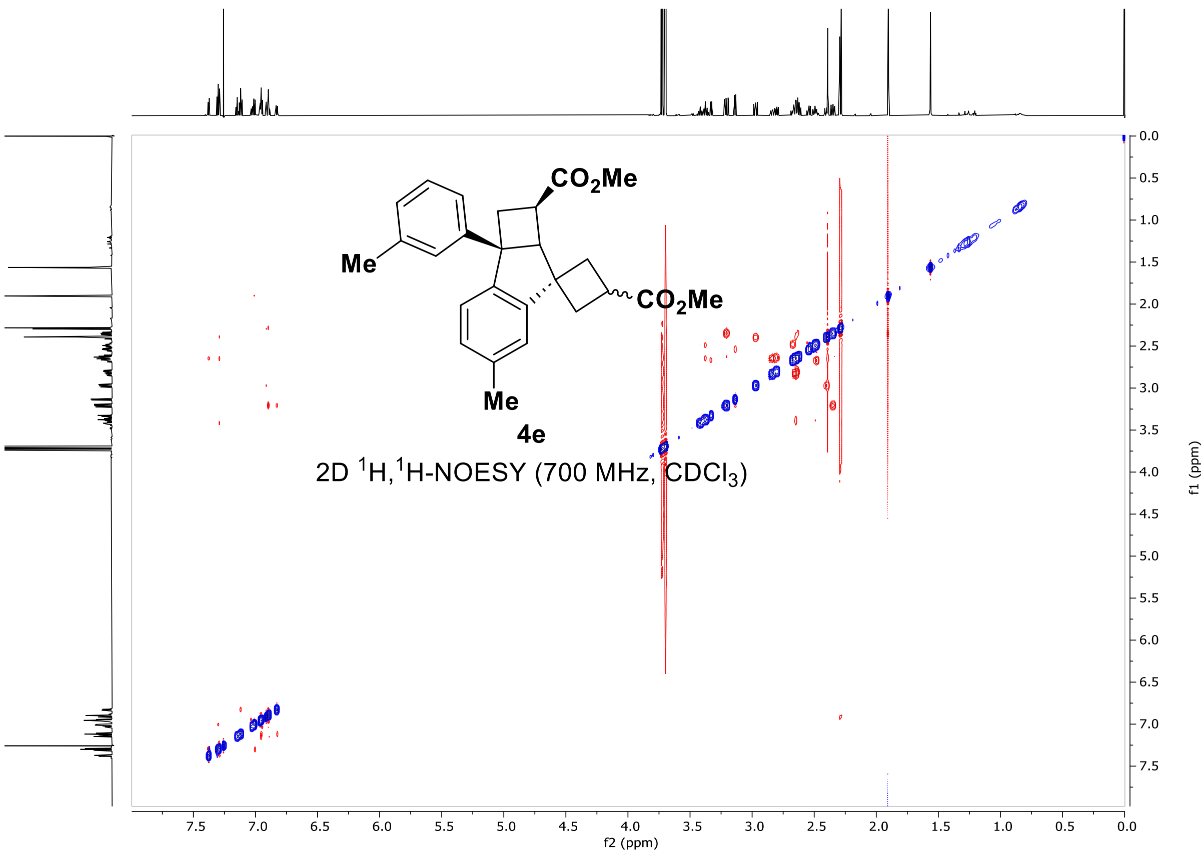

CO2Me
Me
CO2Me
Me
4e
2D 1H,1H-NOESY (700 MHz, CDCl3)
f2 (ppm)
f1 (ppm)

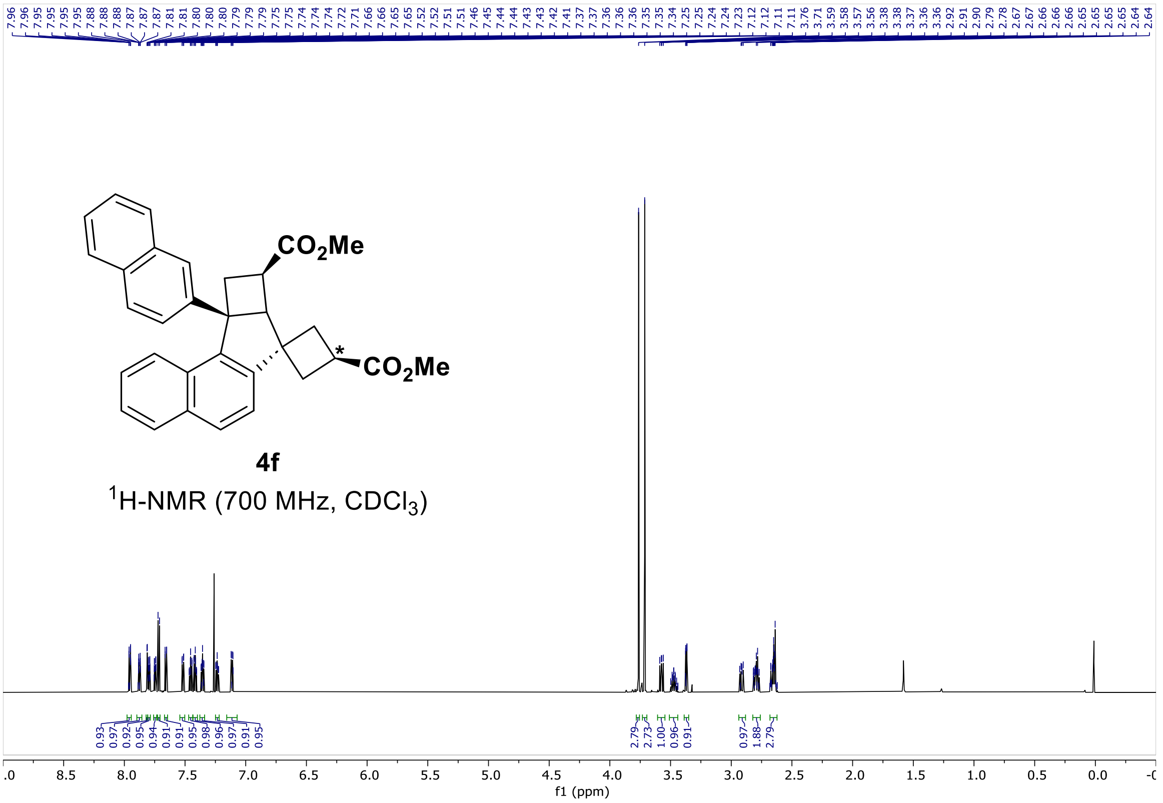
7.96 7.96 7.95 7.95 7.95 7.88 7.88 7.88 7.87 7.87 7.87 7.81 7.80 7.80 7.80 7.79 7.79 7.79 7.75 7.75 7.74 7.74 7.74 7.72 7.71 7.66 7.66 7.65 7.65 7.52 7.52 7.51 7.51 7.46 7.45 7.44 7.44 7.43 7.43 7.42 7.41 7.37 7.37 7.36 7.36 7.36 7.35 7.35 7.34 7.25 7.25 7.24 7.24 7.23 7.12 7.12 7.11 7.11 3.76 3.71 3.59 3.58 3.57 3.56 3.38 3.38 3.37 3.36 3.36 2.92 2.91 2.90 2.79 2.78 2.67 2.67 2.66 2.66 2.66 2.65 2.65 2.65 2.64 2.64
CO2Me
CO2Me
*
4f
1H-NMR (700 MHz, CDCl3)
0.93 0.97 0.92 0.95 0.94 0.91 0.91 0.95 0.98 0.96 0.97 0.91 0.95
2.79 2.73 1.00 0.96 0.91
0.97 1.88 2.79
f1 (ppm)

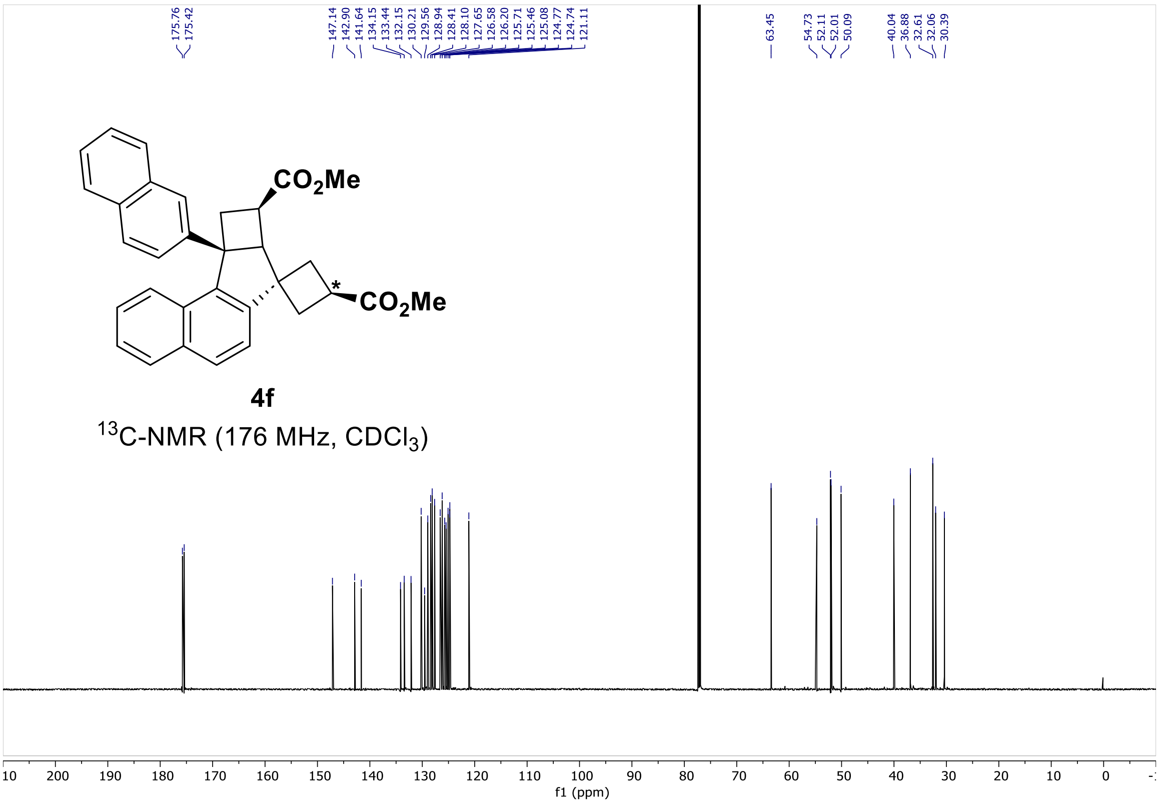
175.76 175.42
147.14 142.90 141.64 134.15 133.44 132.15 130.21 129.56 128.94 128.41 128.10 127.65 126.58 126.20 125.71 125.46 125.08 124.77 124.74 121.11
63.45
54.73 52.11 52.01 50.09
40.04 36.88 32.61 32.06 30.39
CO2Me
CO2Me
*
4f
13C-NMR (176 MHz, CDCl3)
f1 (ppm)

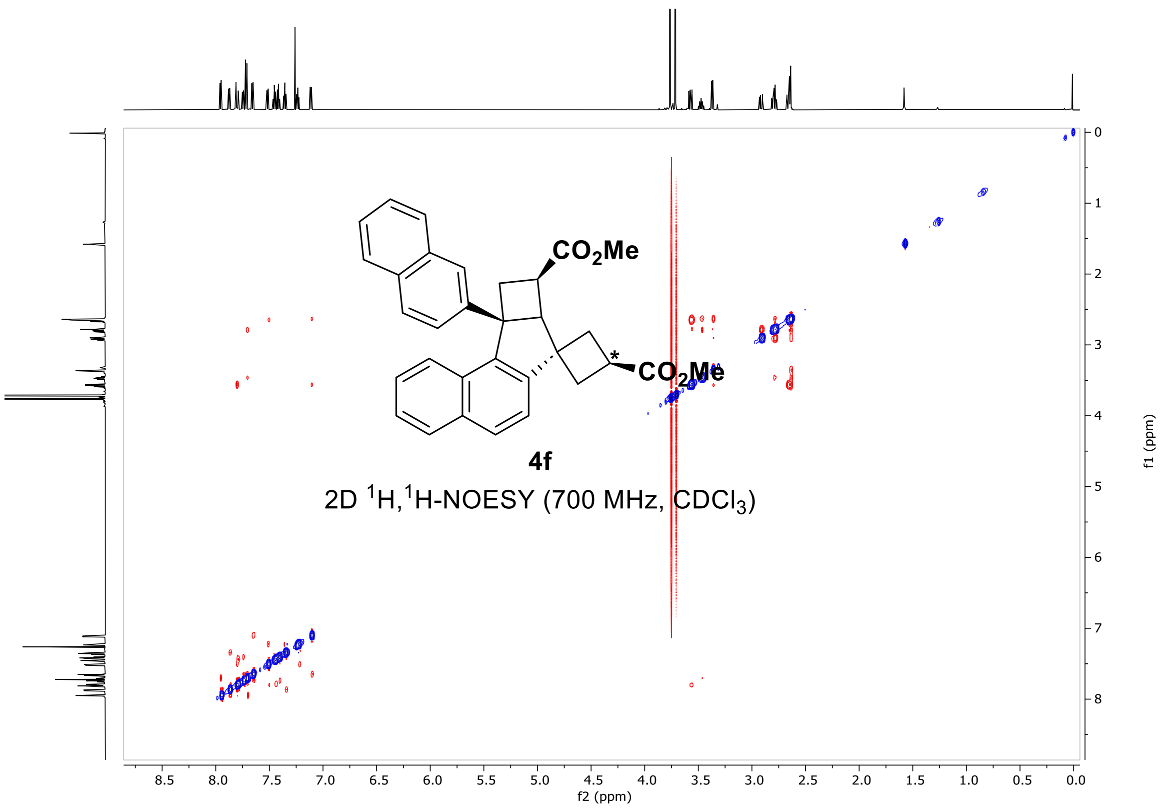
CO2Me
CO2Me
*
4f
2D 1H,1H-NOESY (700 MHz, CDCl3)
f2 (ppm)
f1 (ppm)

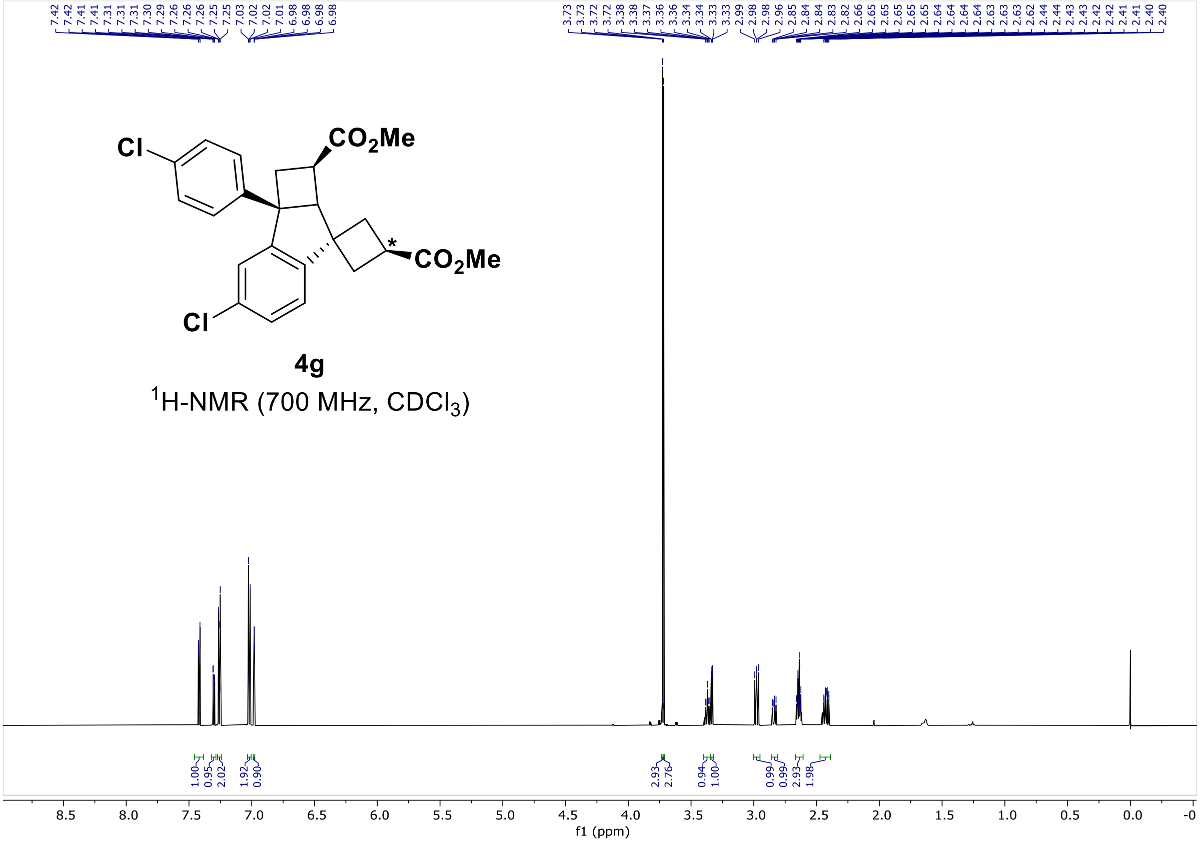
7.42 7.42 7.41 7.41 7.31 7.31 7.31 7.30 7.29 7.26 7.26 7.25 7.25 7.03 7.02 7.01 6.98 6.98 6.98 6.98
3.73 3.73 3.72 3.72 3.38 3.38 3.37 3.36 3.36 3.34 3.34 3.33 3.33 2.99 2.98 2.98 2.96 2.85 2.84 2.84 2.83 2.82 2.66 2.65 2.65 2.65 2.65 2.64 2.64 2.64 2.63 2.63 2.62 2.44 2.44 2.43 2.43 2.42 2.42 2.41 2.40 2.40
Cl
CO2Me
*
CO2Me
Cl
4g
1H-NMR (700 MHz, CDCl3)
1.00 0.95 2.02 1.92 0.90 2.93 2.76 0.94 1.00 0.99 0.99 2.93 1.98
f1 (ppm)

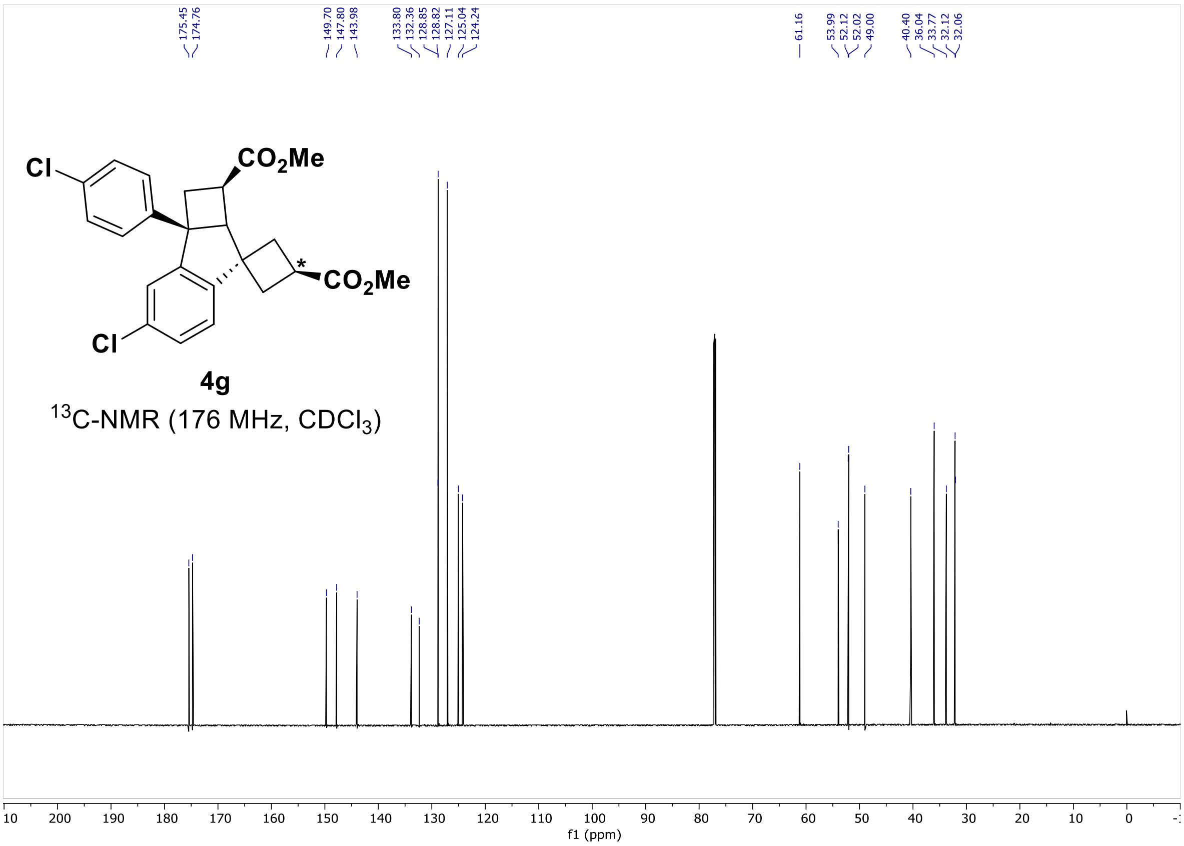
175.45 174.76 149.70 147.80 143.98 133.80 132.36 128.85 128.82 127.11 125.04 124.24 61.16 53.99 52.12 52.02 49.00 40.40 36.04 33.77 32.12 32.06
Cl
CO2Me
*
CO2Me
Cl
4g
13C-NMR (176 MHz, CDCl3)
f1 (ppm)

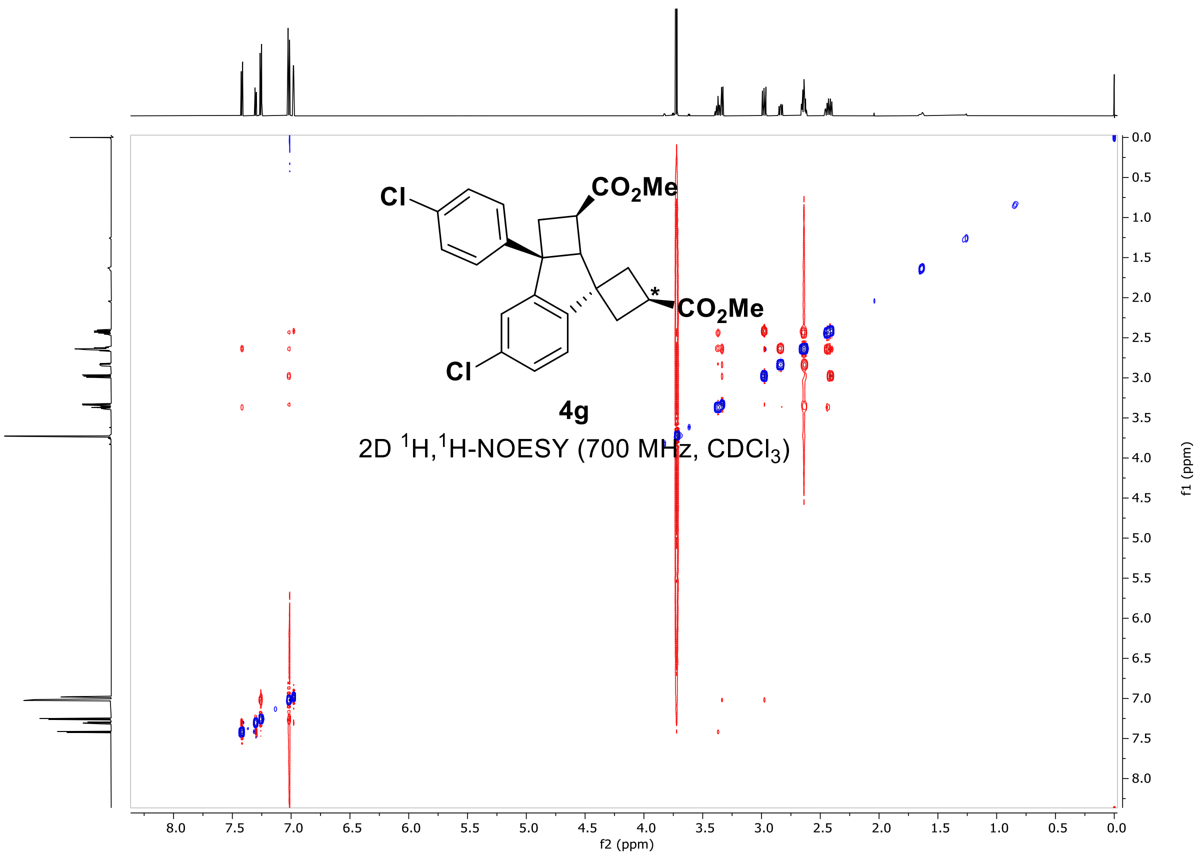
Cl
CO2Me
*
CO2Me
Cl
4g
2D 1H,1H-NOESY (700 MHz, CDCl3)
f1 (ppm)
f2 (ppm)

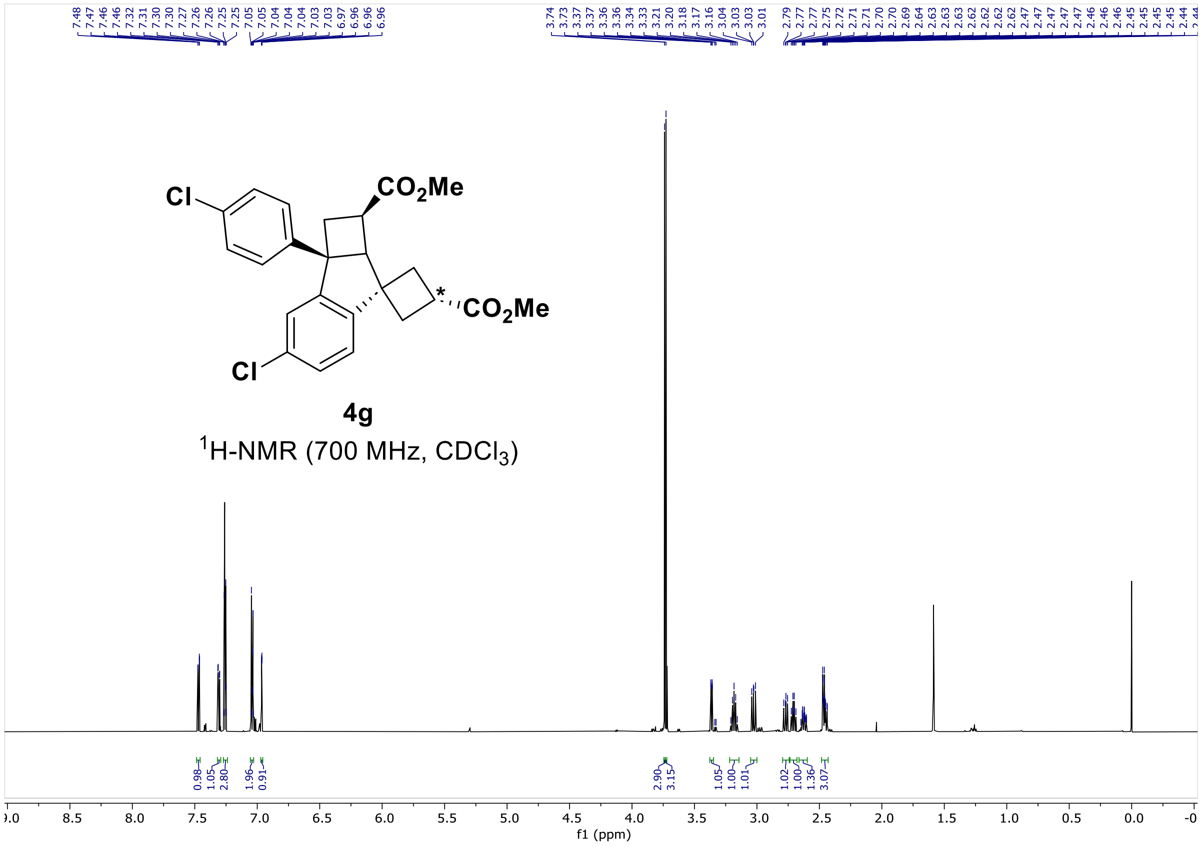

7.48 7.47 7.46 7.32 7.31 7.30 7.27 7.26 7.26 7.25 7.05 7.05 7.04 7.04 7.04 7.03 6.97 6.96 6.96 6.96
3.74 3.73 3.37 3.37 3.36 3.34 3.33 3.21 3.20 3.18 3.17 3.16 3.04 3.03 3.03 3.01
2.79 2.77 2.77 2.75 2.72 2.71 2.70 2.69 2.64 2.63 2.63 2.63 2.62 2.62 2.62 2.47 2.47 2.47 2.47 2.46 2.46 2.45 2.45 2.45 2.44
Cl
CO2Me
CO2Me
Cl
4g
1H-NMR (700 MHz, CDCl3)
0.98 1.05 2.80 1.96 0.91
2.90 3.15
1.05 1.00 1.01
1.02 1.00 1.36 3.07
f1 (ppm)

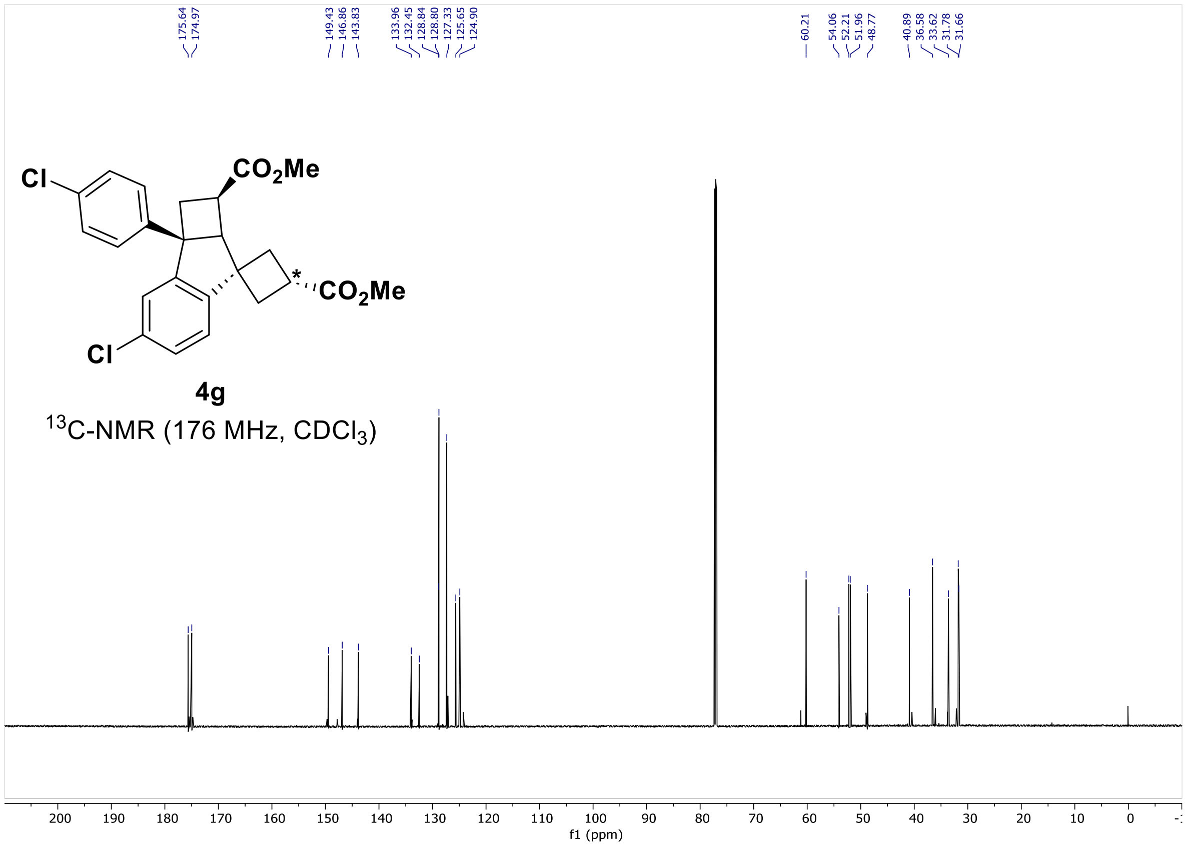

175.64 174.97
149.43 146.86 143.83
133.96 132.45 128.84 128.80 127.33 125.65 124.90
60.21
54.06 52.21 51.96 48.77
40.89 36.58 33.62 31.78 31.66
Cl
CO2Me
CO2Me
Cl
4g
13C-NMR (176 MHz, CDCl3)
f1 (ppm)

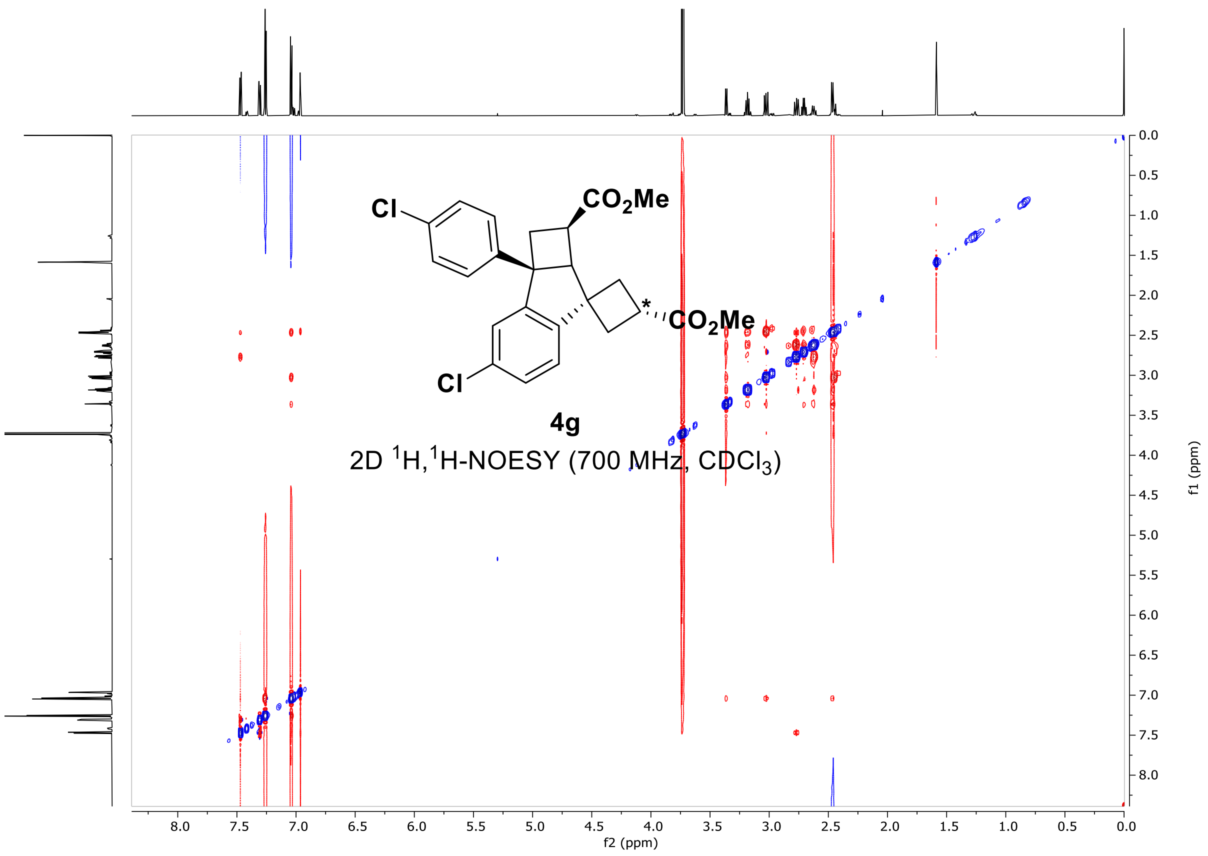

Cl
CO2Me
*
CO2Me
Cl
4g
2D 1H,1H-NOESY (700 MHz, CDCl3)
f2 (ppm)
f1 (ppm)

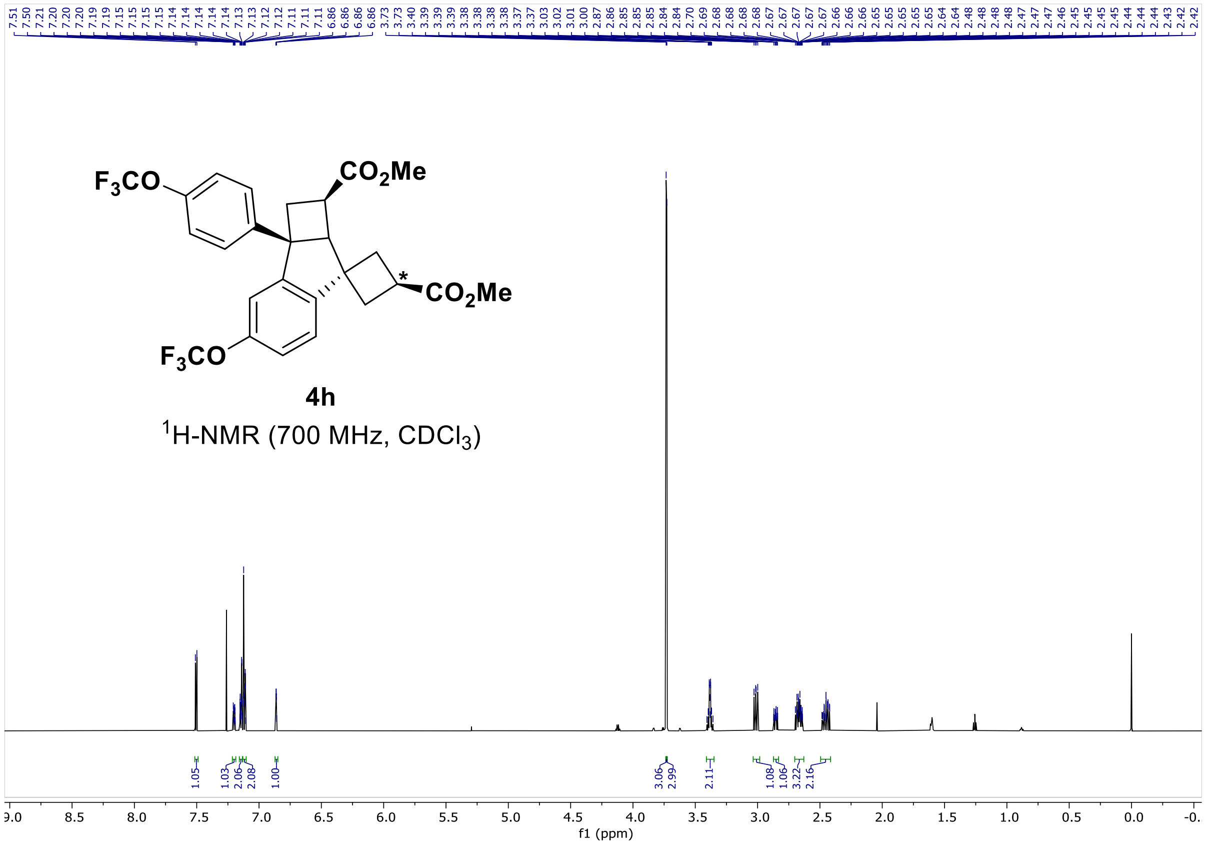
F3CO
CO2Me
CO2Me
F3CO
4h
1H-NMR (700 MHz, CDCl3)
f1 (ppm)

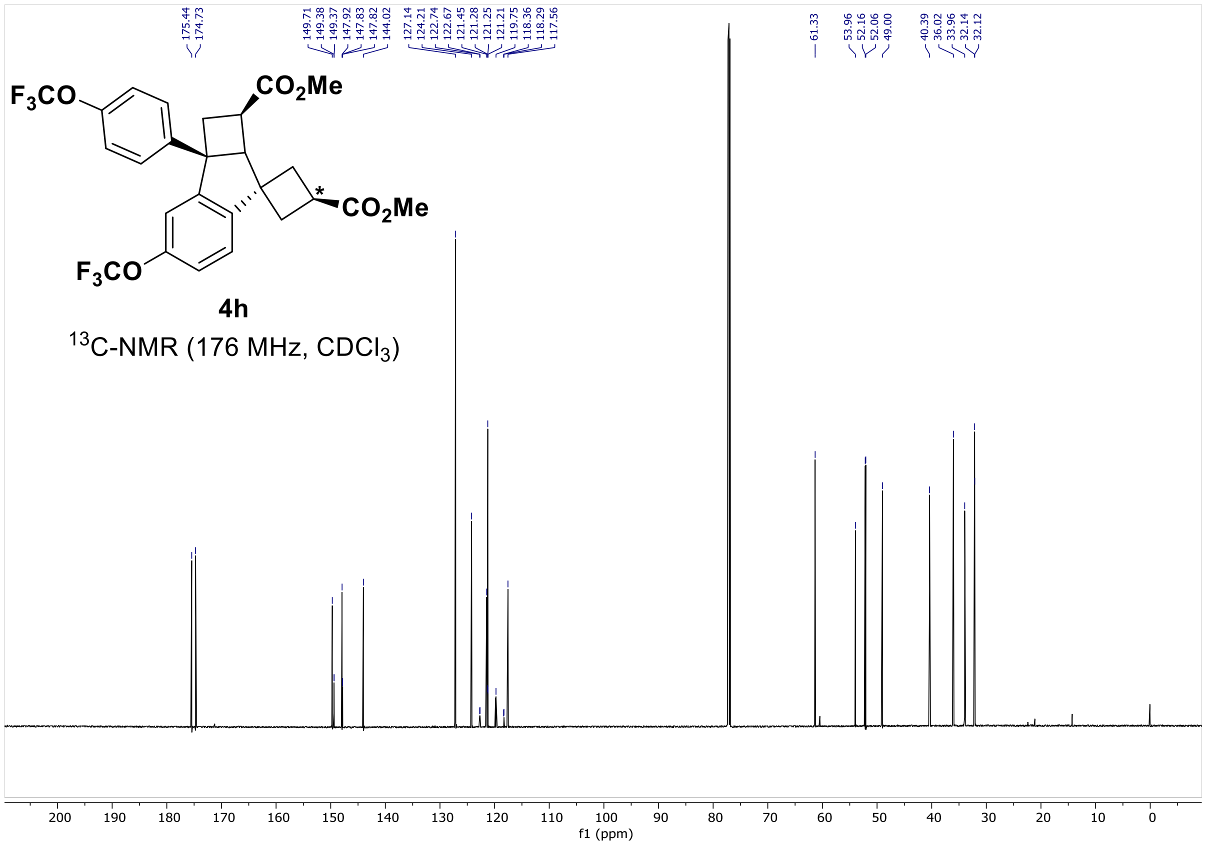
F3CO
CO2Me
CO2Me
F3CO
4h
13C-NMR (176 MHz, CDCl3)
f1 (ppm)

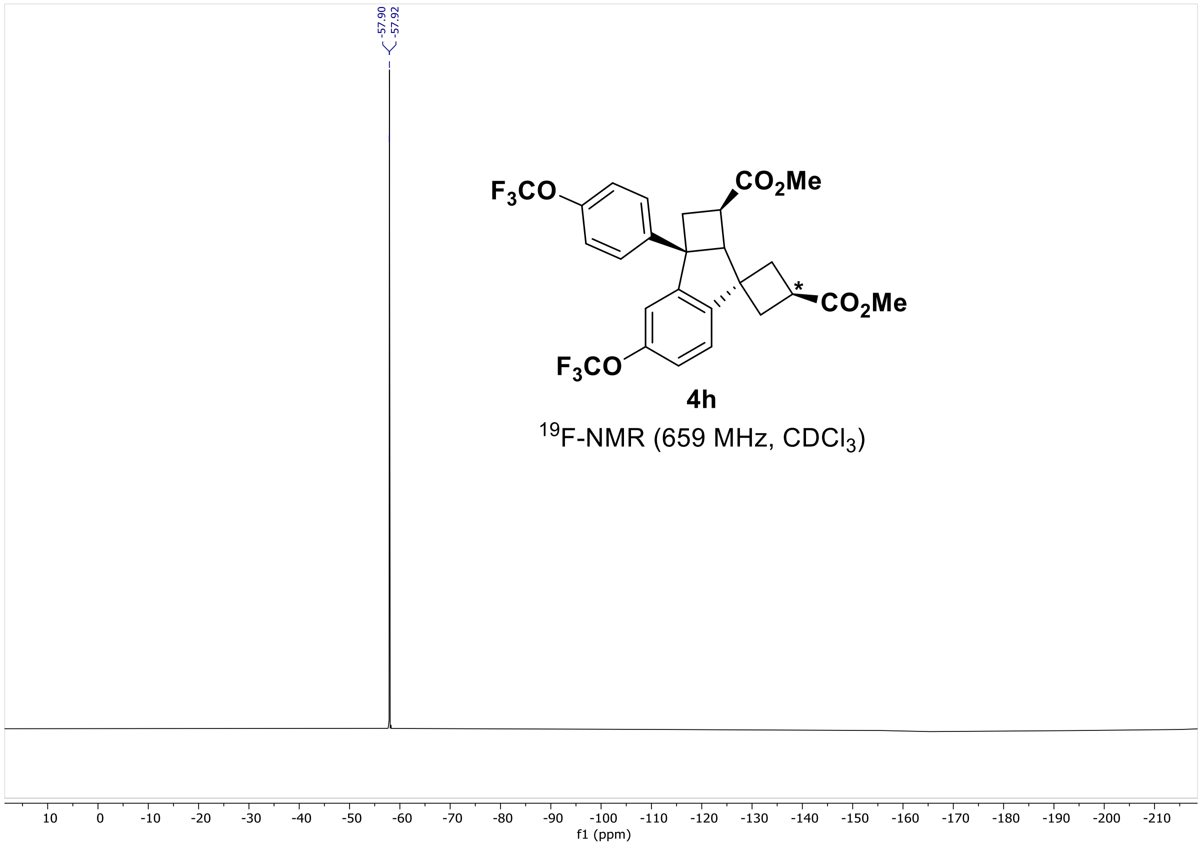
-57.90
-57.92
F3CO
CO2Me
CO2Me
F3CO
4h
19F-NMR (659 MHz, CDCl3)
f1 (ppm)

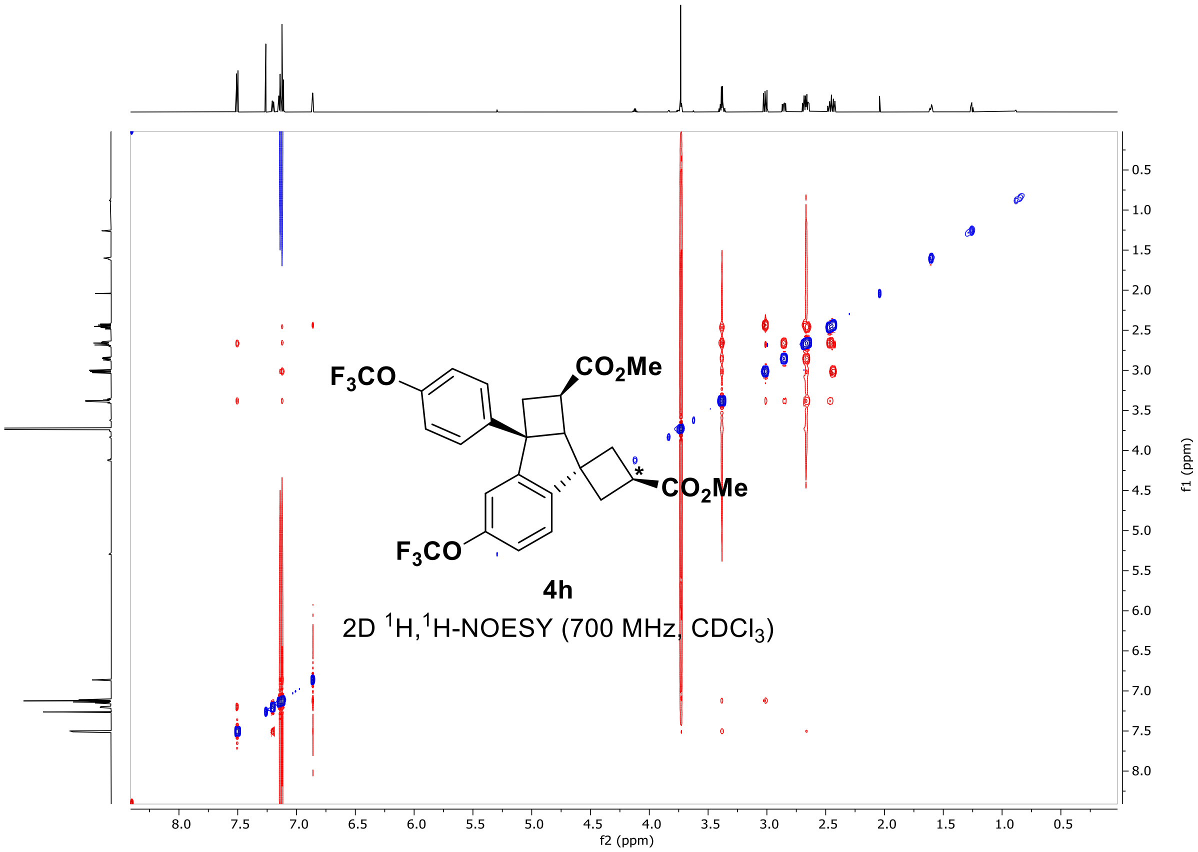
F3CO
CO2Me
CO2Me
F3CO
4h
2D 1H,1H-NOESY (700 MHz, CDCl3)
f2 (ppm)
f1 (ppm)

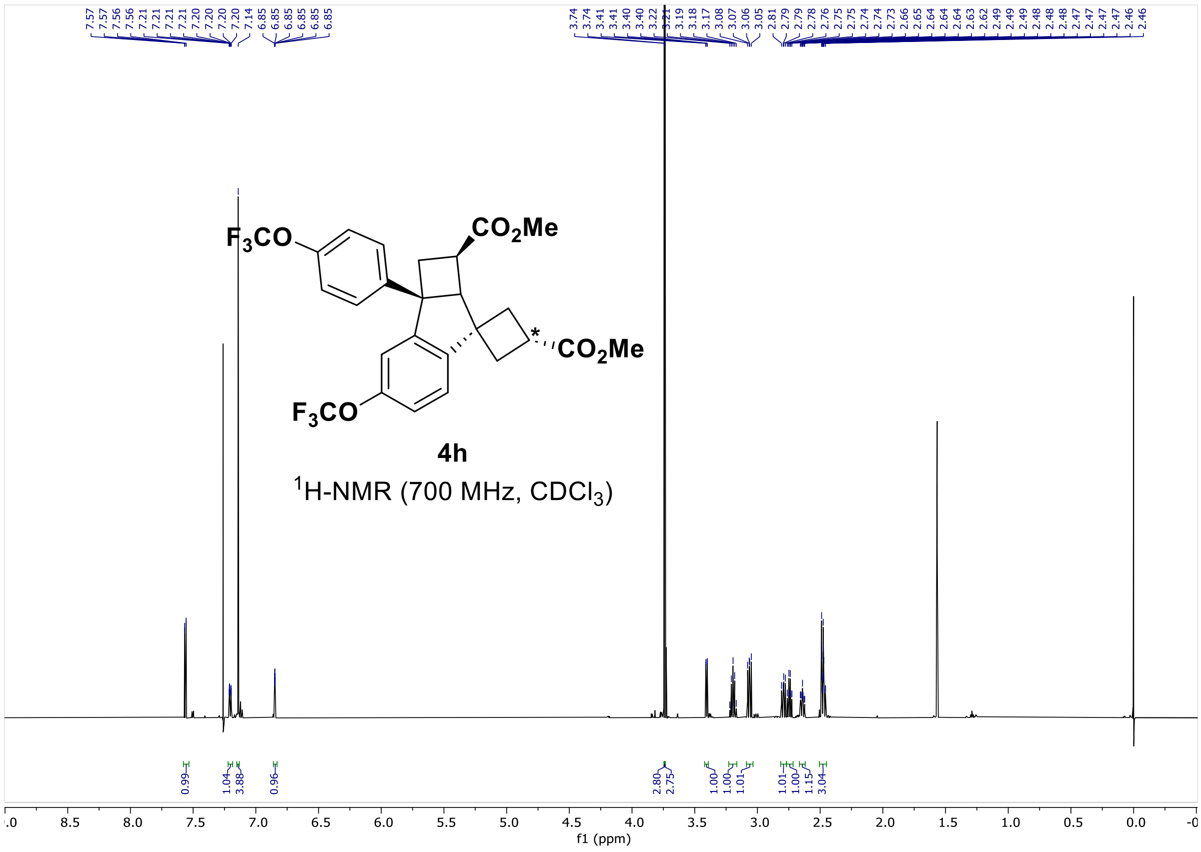

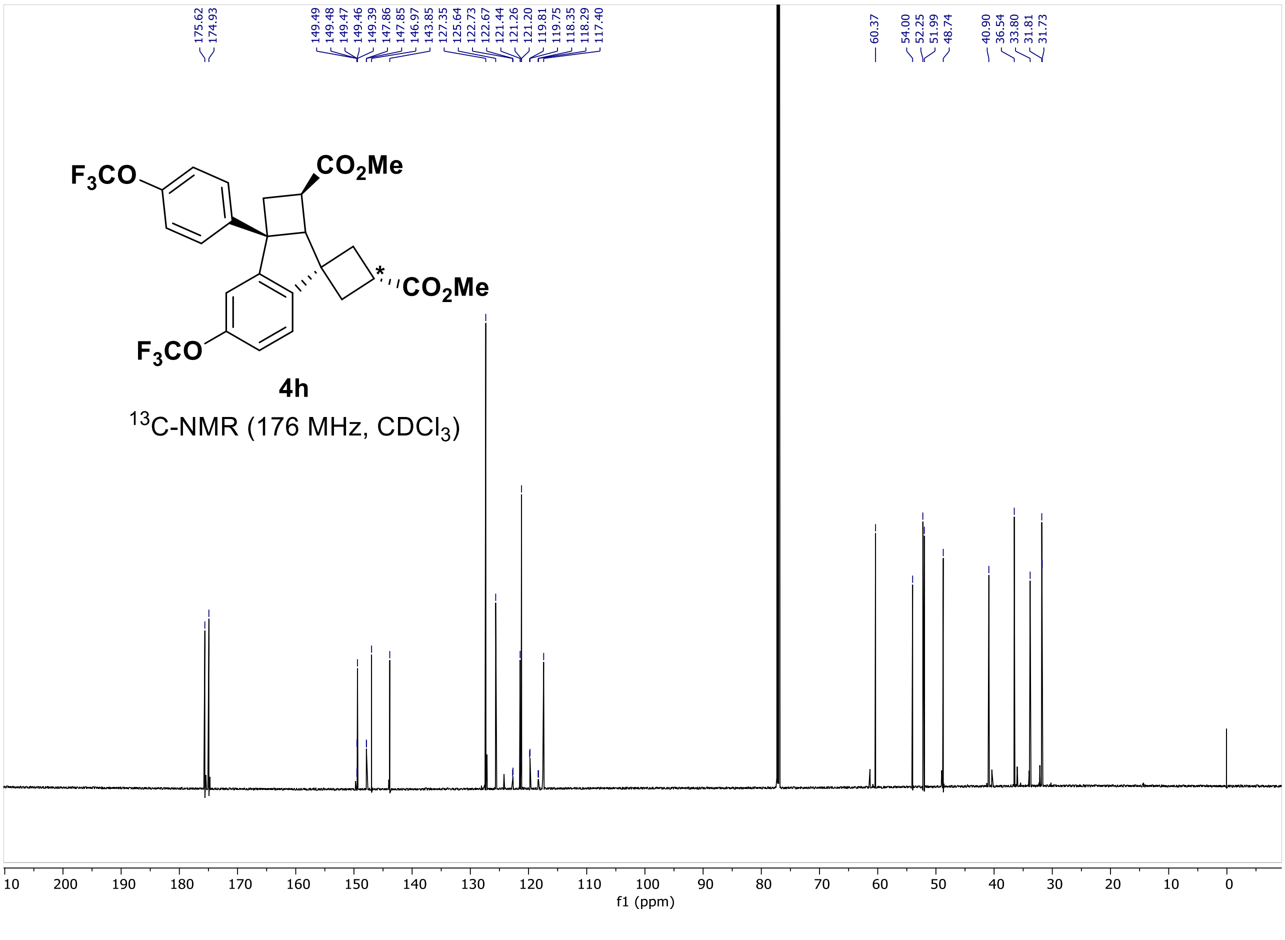

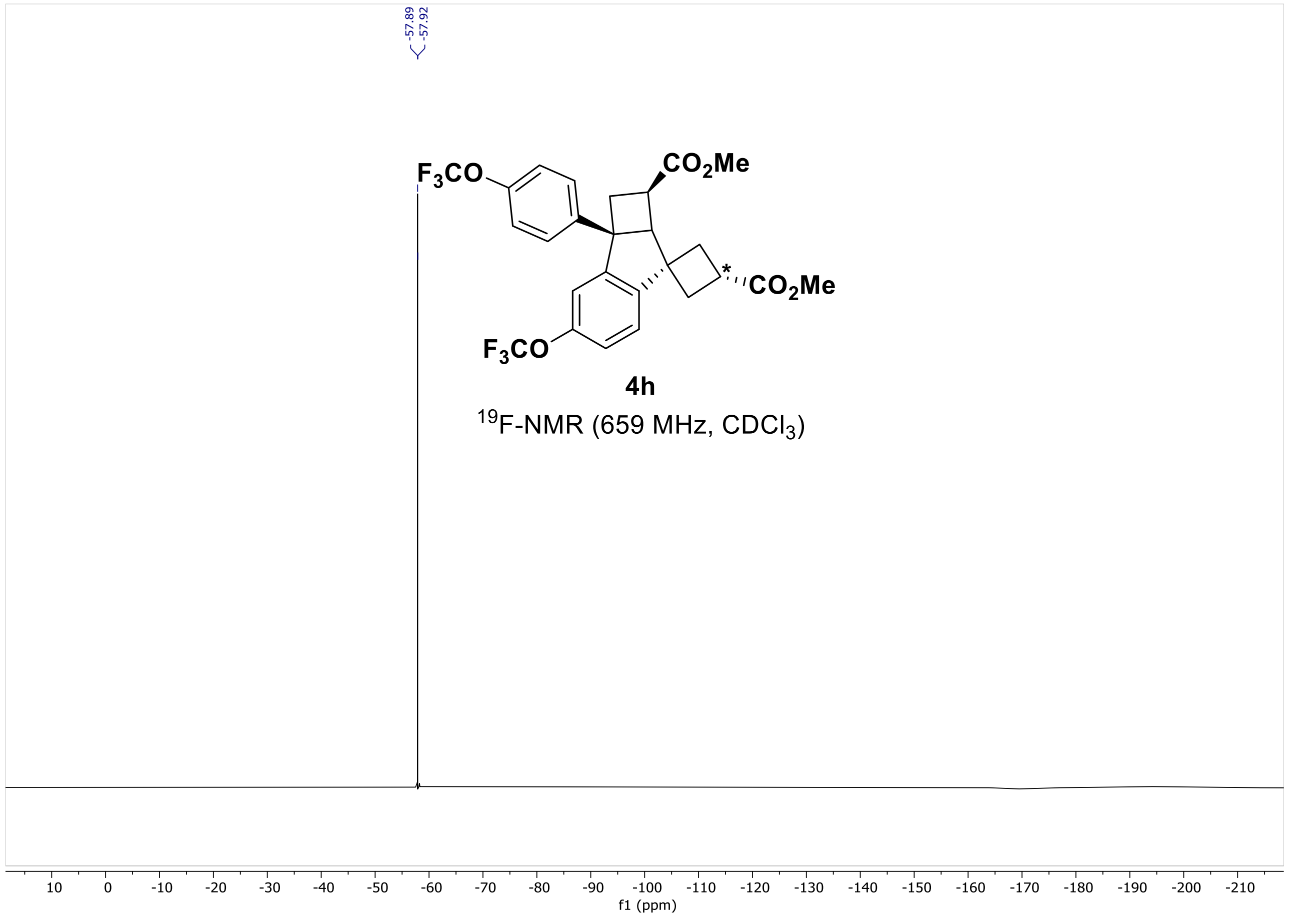

-57.89
-57.92
F3CO
CO2Me
CO2Me
F3CO
4h
19F-NMR (659 MHz, CDCl3)
f1 (ppm)

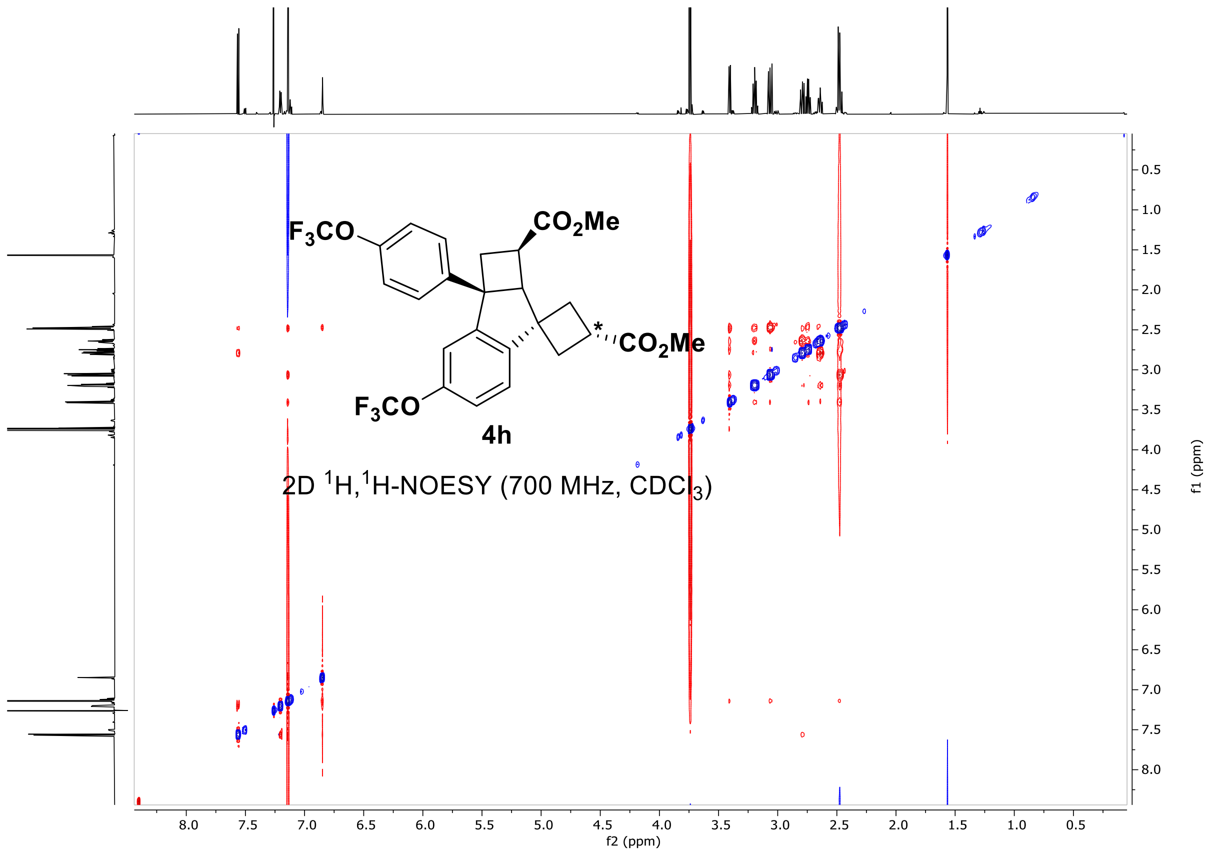

F3CO
CO2Me
CO2Me
F3CO
4h
2D 1H,1H-NOESY (700 MHz, CDCl3)
f2 (ppm)
f1 (ppm)

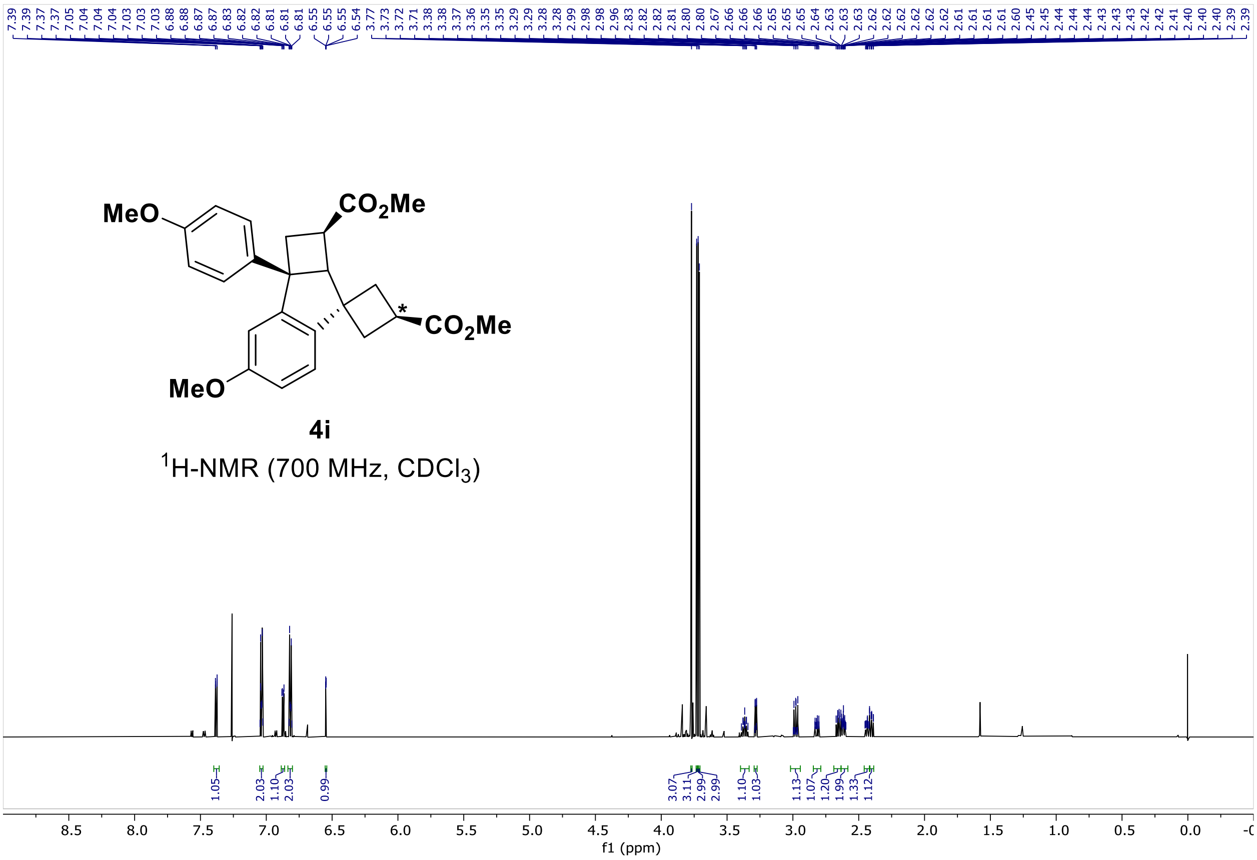
7.39 7.39 7.37 7.05 7.04 7.04 7.04 7.03 7.03 7.03 6.88 6.88 6.87 6.83 6.82 6.82 6.81 6.81 6.81 6.55 6.55 6.55 6.54 3.77 3.73 3.72 3.71 3.38 3.38 3.37 3.36 3.35 3.35 3.29 3.29 3.28 3.28 2.99 2.98 2.98 2.96 2.83 2.82 2.82 2.81 2.80 2.80 2.67 2.66 2.66 2.66 2.65 2.65 2.65 2.64 2.63 2.63 2.63 2.62 2.62 2.62 2.62 2.62 2.62 2.61 2.61 2.61 2.61 2.60 2.45 2.45 2.44 2.44 2.43 2.43 2.43 2.42 2.42 2.41 2.40 2.40 2.40 2.39 2.39
MeO
CO2Me
CO2Me
MeO
4i
1H-NMR (700 MHz, CDCl3)
1.05 2.03 1.10 2.03 0.99 3.07 3.11 2.99 2.99 1.10 1.03 1.13 1.07 1.20 1.99 1.33 1.12
8.5 8.0 7.5 7.0 6.5 6.0 5.5 5.0 4.5 4.0 3.5 3.0 2.5 2.0 1.5 1.0 0.5 0.0
f1 (ppm)

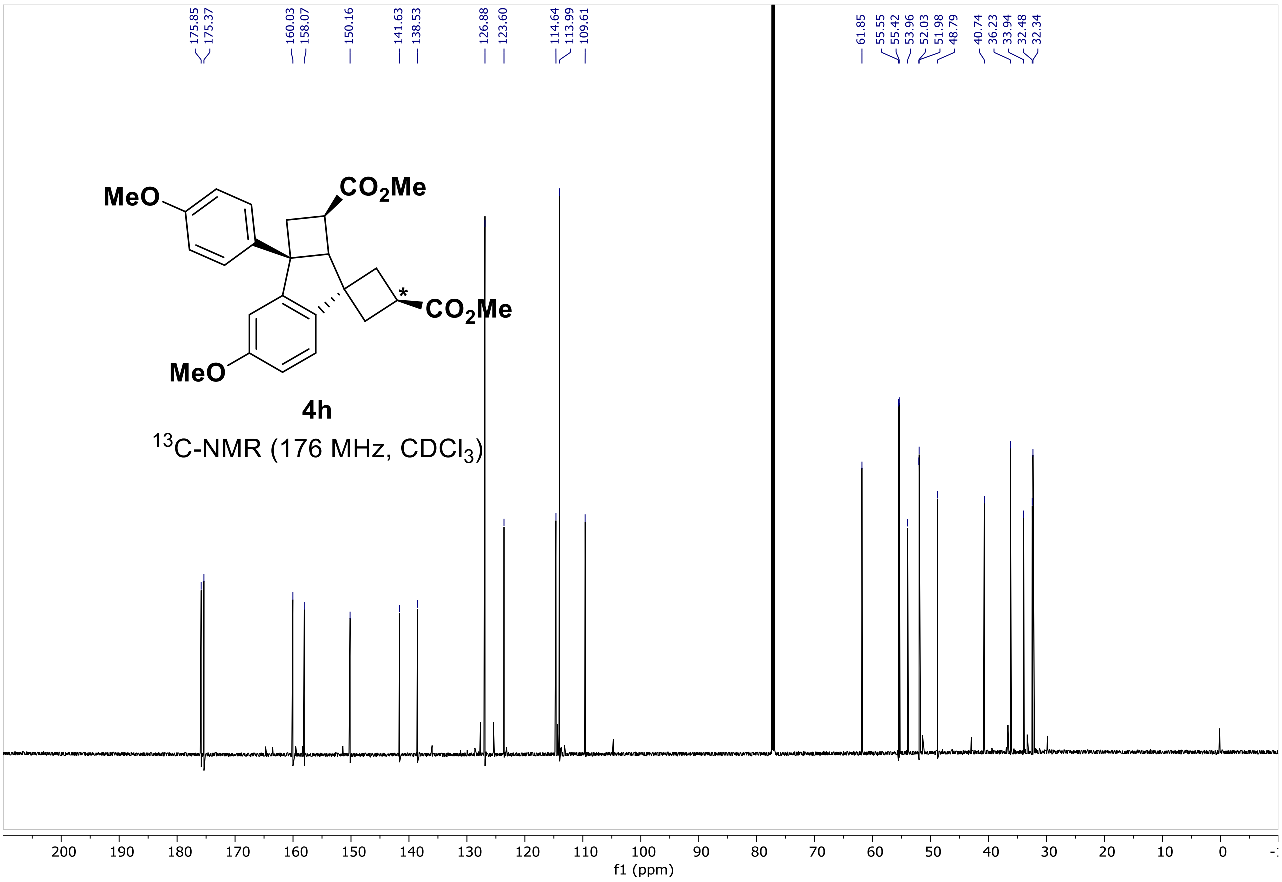
175.85 175.37 160.03 158.07 150.16 141.63 138.53 126.88 123.60 114.64 113.99 109.61 61.85 55.55 55.42 53.96 52.03 51.98 48.79 40.74 36.23 33.94 32.48 32.34
MeO
CO2Me
CO2Me
MeO
4h
13C-NMR (176 MHz, CDCl3)
200 190 180 170 160 150 140 130 120 110 100 90 80 70 60 50 40 30 20 10 0
f1 (ppm)

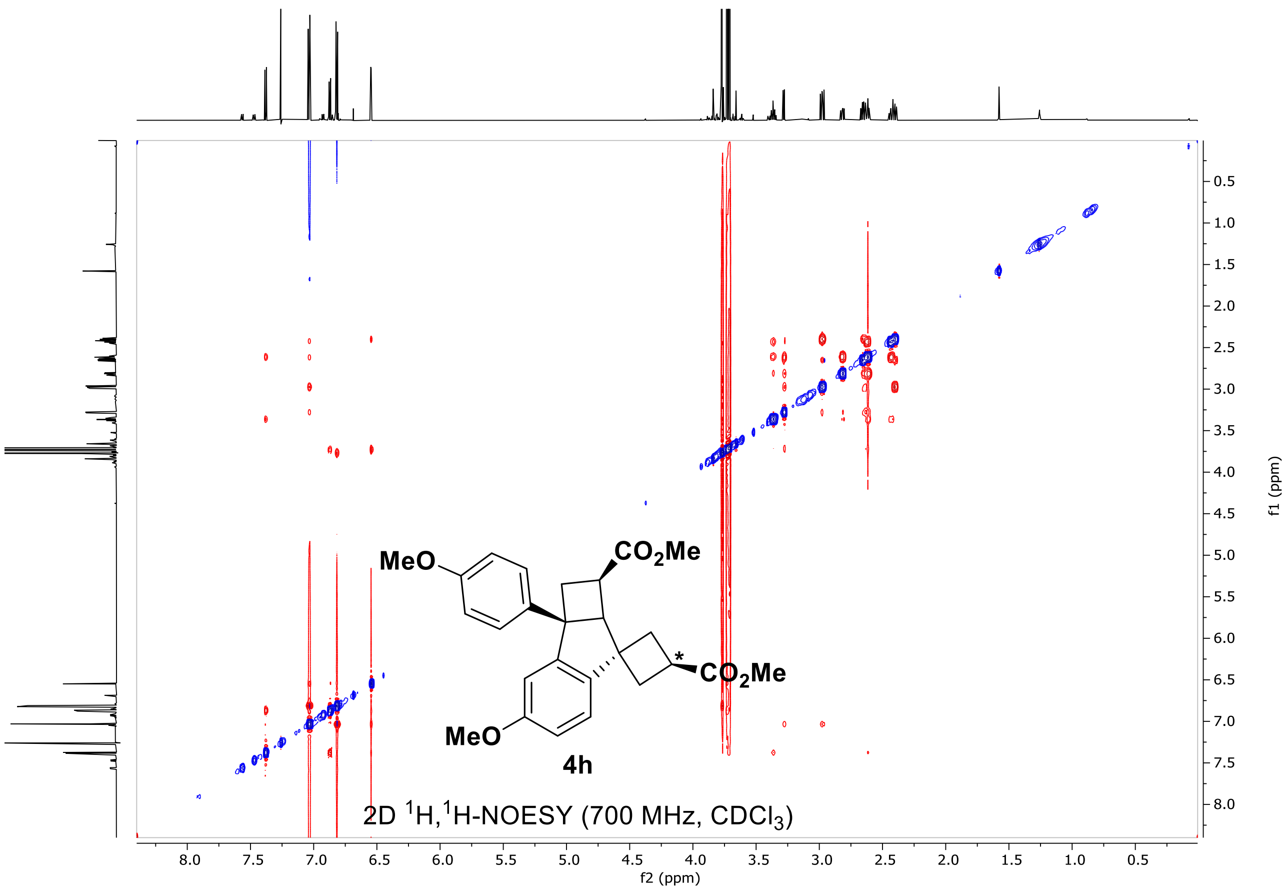
MeO
CO_2Me
*
CO_2Me
MeO
4h
2D ^{1}H,^{1}H-NOESY (700 MHz, $CDCl_3$)
f2 (ppm)
f1 (ppm)

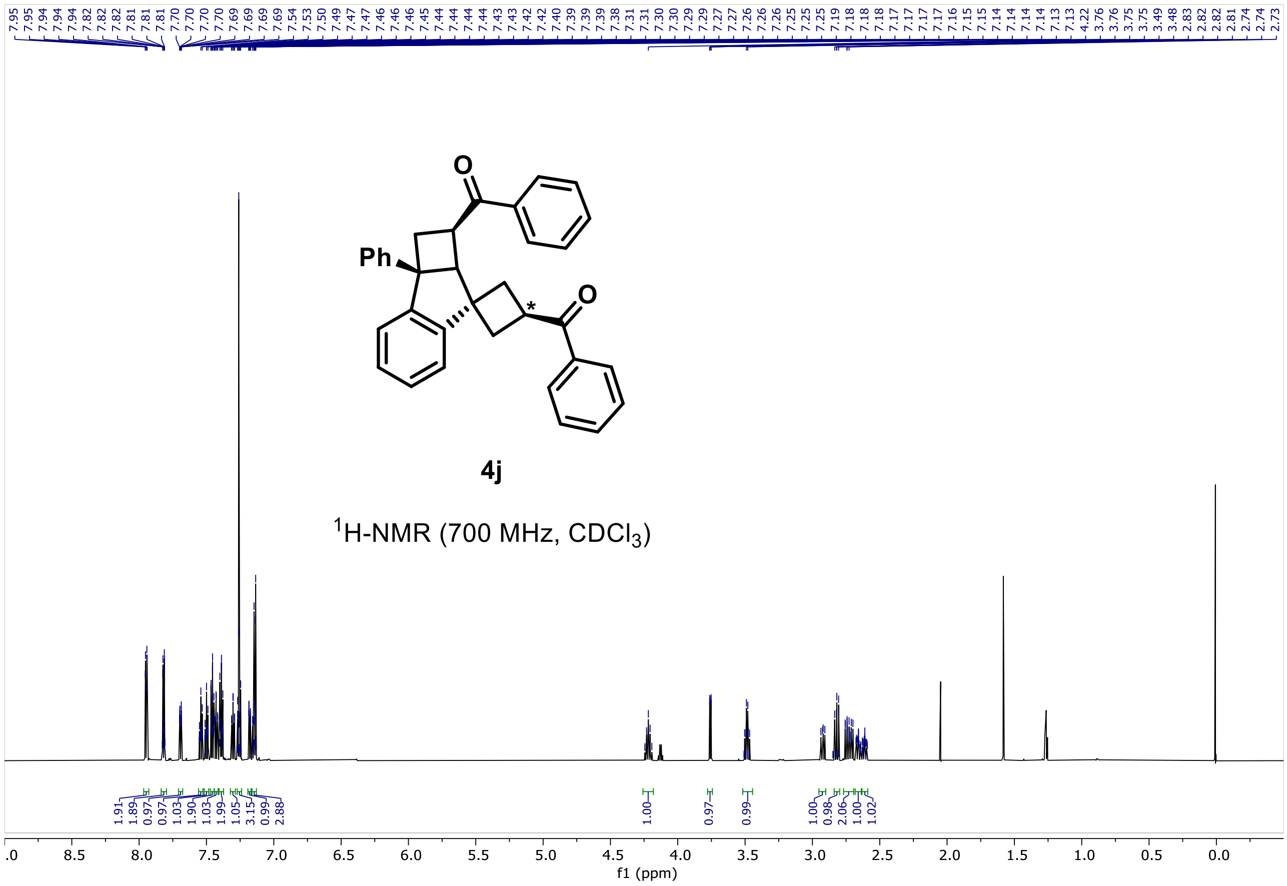
Ph
O
O
*
4j
^{1}H-NMR (700 MHz, $CDCl_3$)
f1 (ppm)

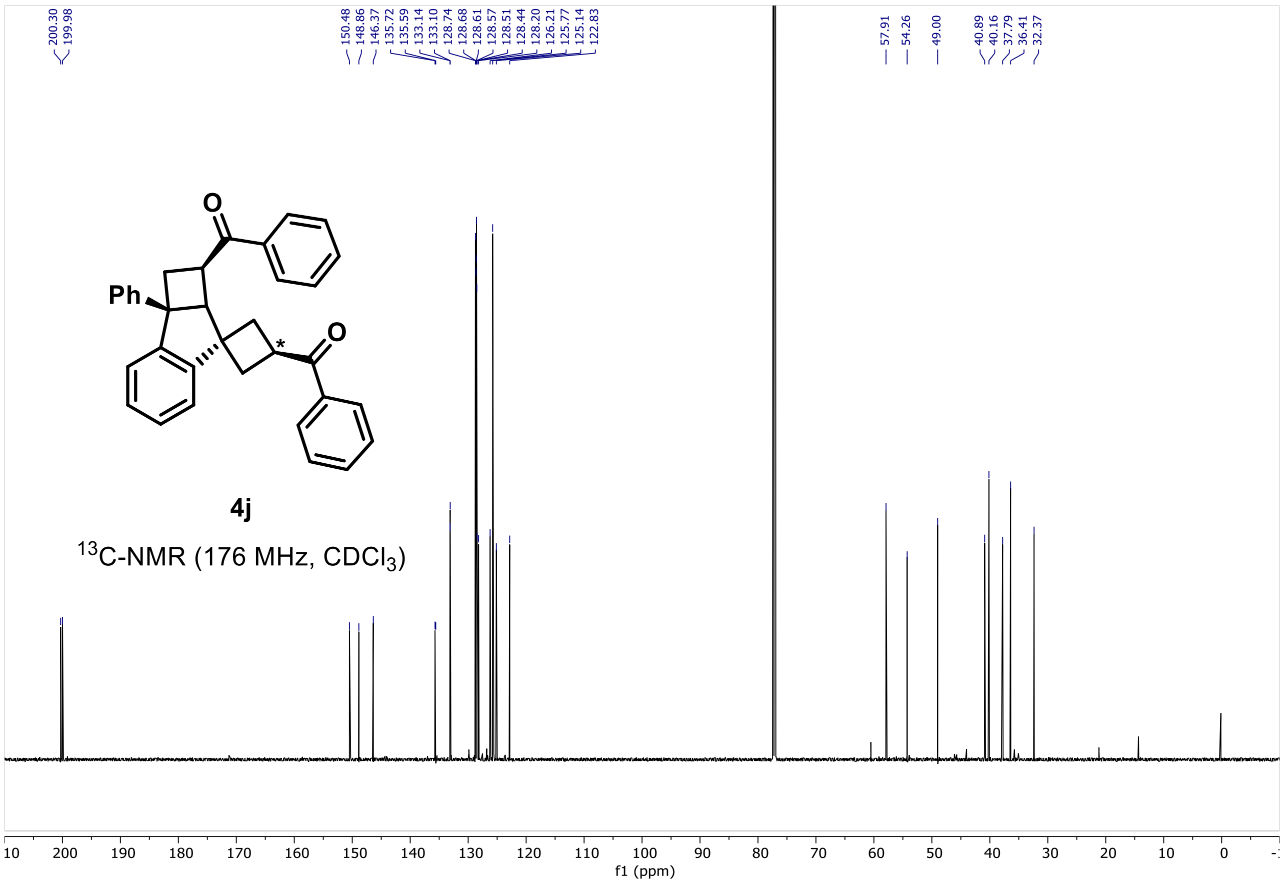
200.30
199.98
150.48
148.86
146.37
135.72
135.59
133.14
133.10
128.74
128.68
128.61
128.57
128.51
128.44
128.20
126.21
125.77
125.14
122.83
57.91
54.26
49.00
40.89
40.16
37.79
36.41
32.37
Ph
O
O
*
4j
^{13}C-NMR (176 MHz, $CDCl_3$)
f1 (ppm)

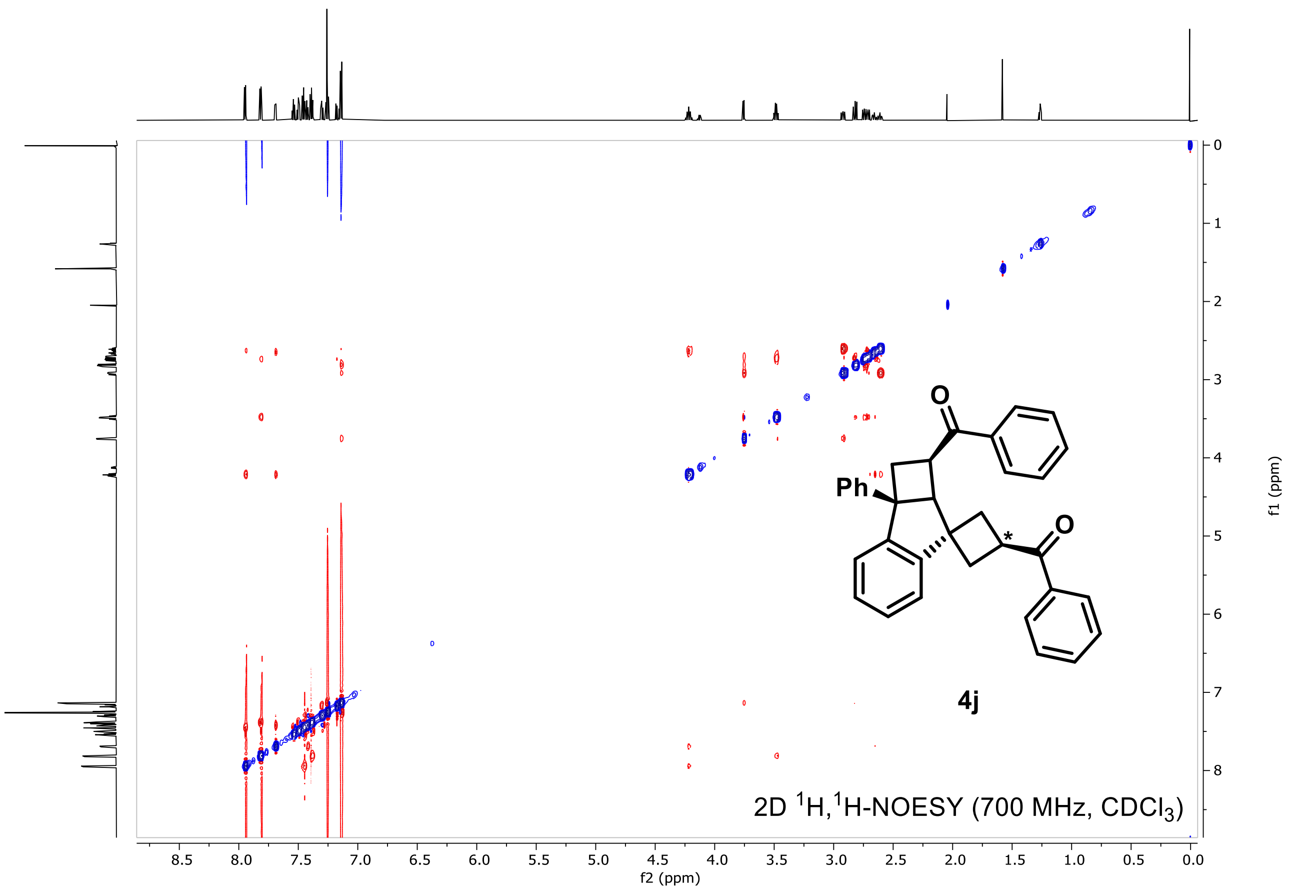
2D ¹H,¹H-NOESY (700 MHz, CDCl3)
4j
Ph
O
O
*
f2 (ppm)
f1 (ppm)

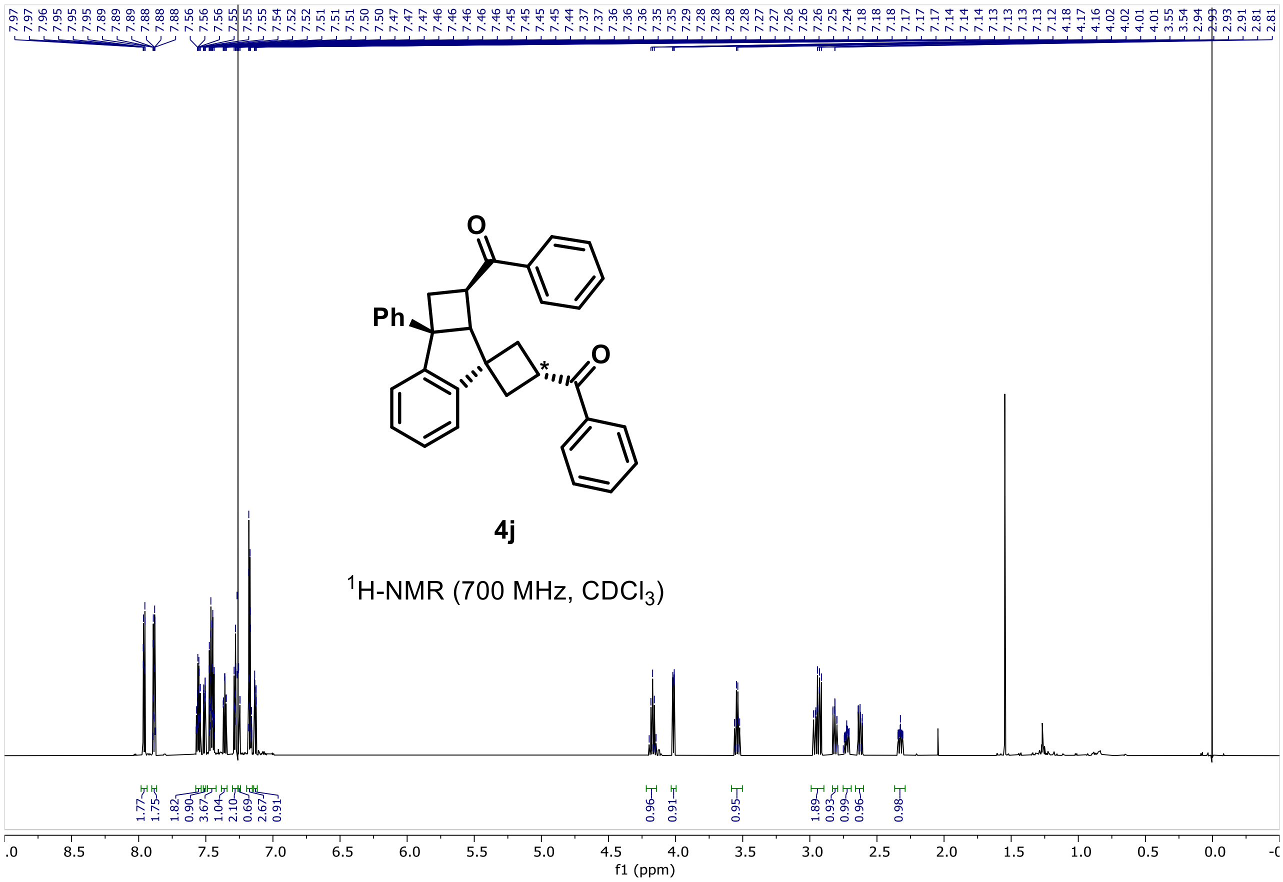
Ph
O
4j
1H-NMR (700 MHz, CDCl3)
f1 (ppm)

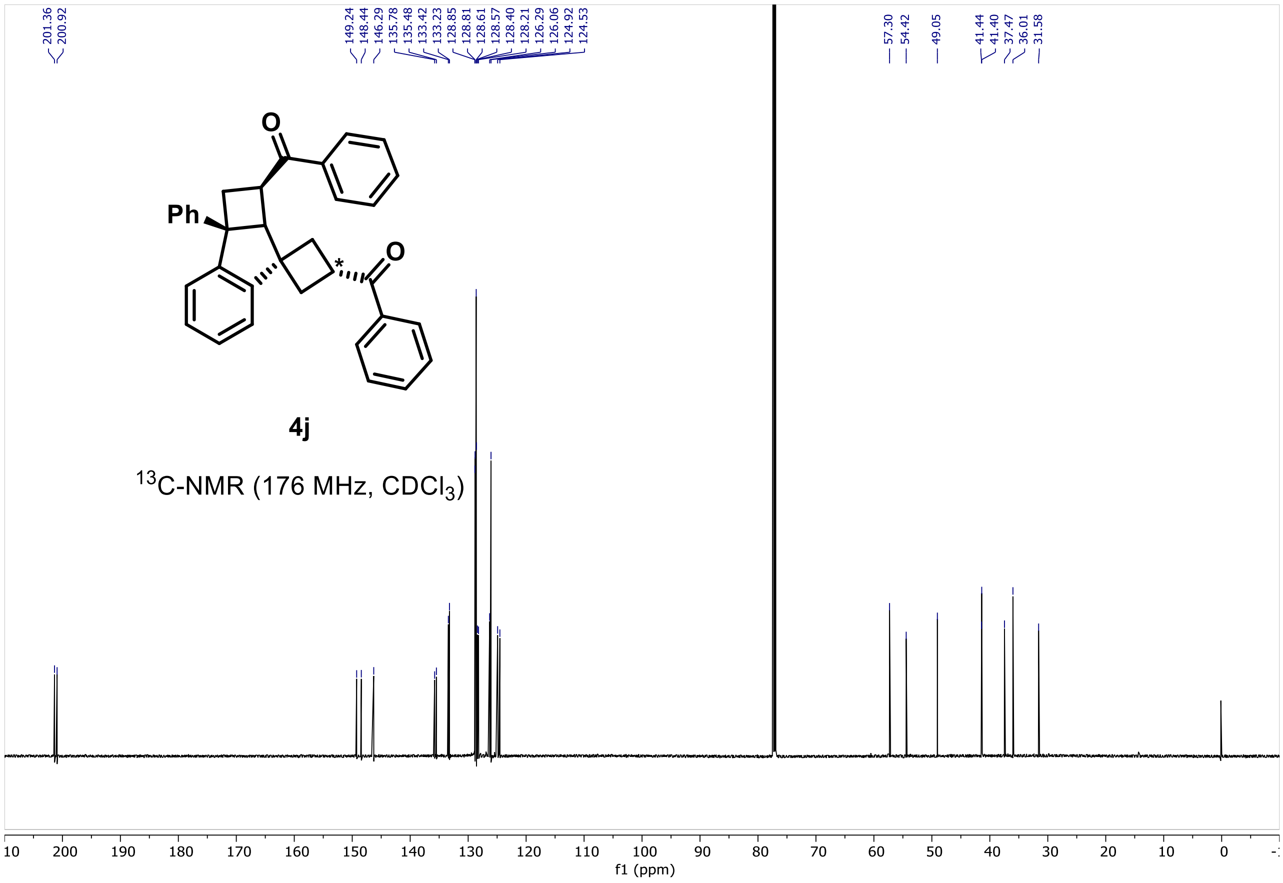
201.36
200.92
149.24
148.44
146.29
135.78
135.48
133.42
133.23
128.85
128.81
128.61
128.57
128.40
128.21
126.29
126.06
124.92
124.53
57.30
54.42
49.05
41.44
41.40
37.47
36.01
31.58
Ph
O
4j
13C-NMR (176 MHz, CDCl3)
f1 (ppm)

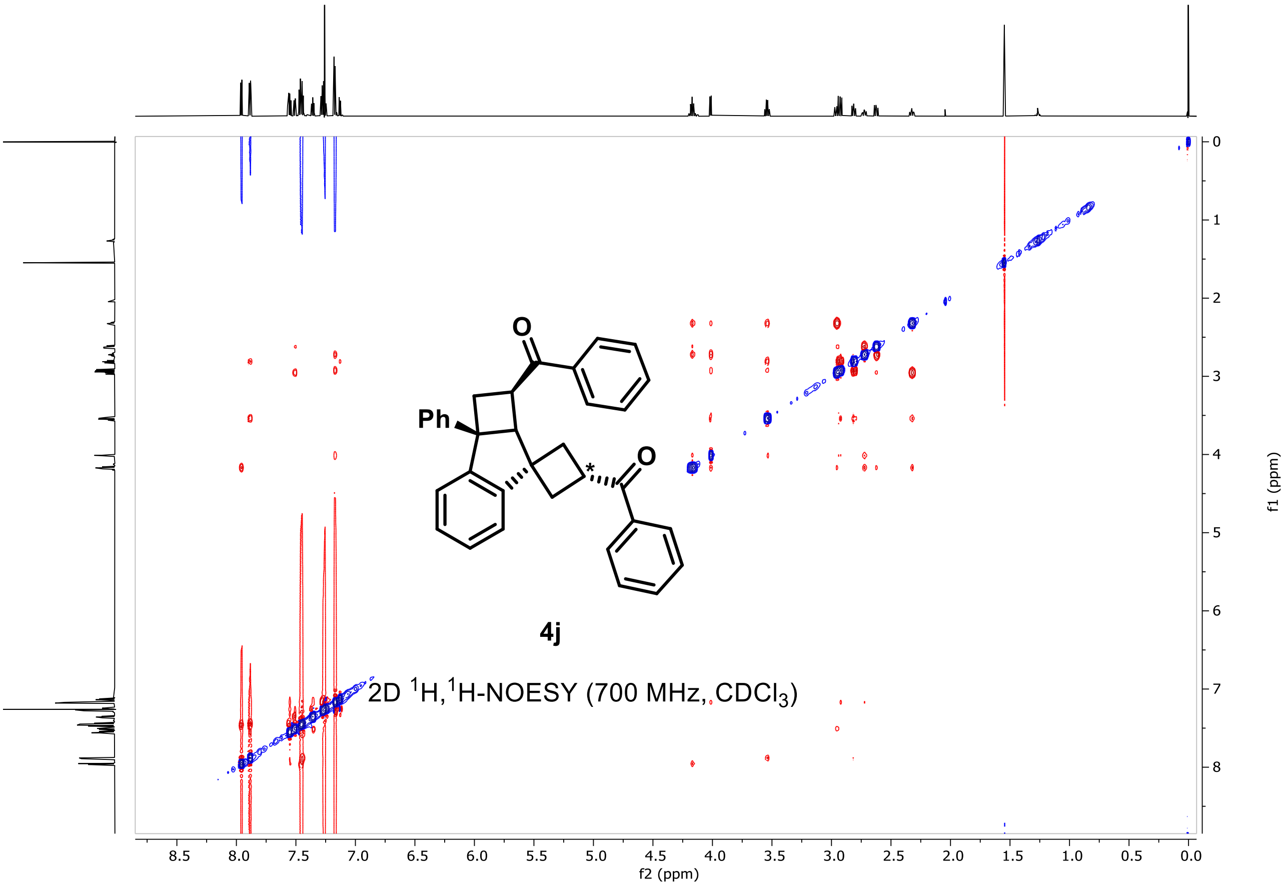
Ph
O
O
*
4j
2D 1H,1H-NOESY (700 MHz, CDCl3)
f2 (ppm)
f1 (ppm)
8.5
8.0
7.5
7.0
6.5
6.0
5.5
5.0
4.5
4.0
3.5
3.0
2.5
2.0
1.5
1.0
0.5
0.0
0
1
2
3
4
5
6
7
8

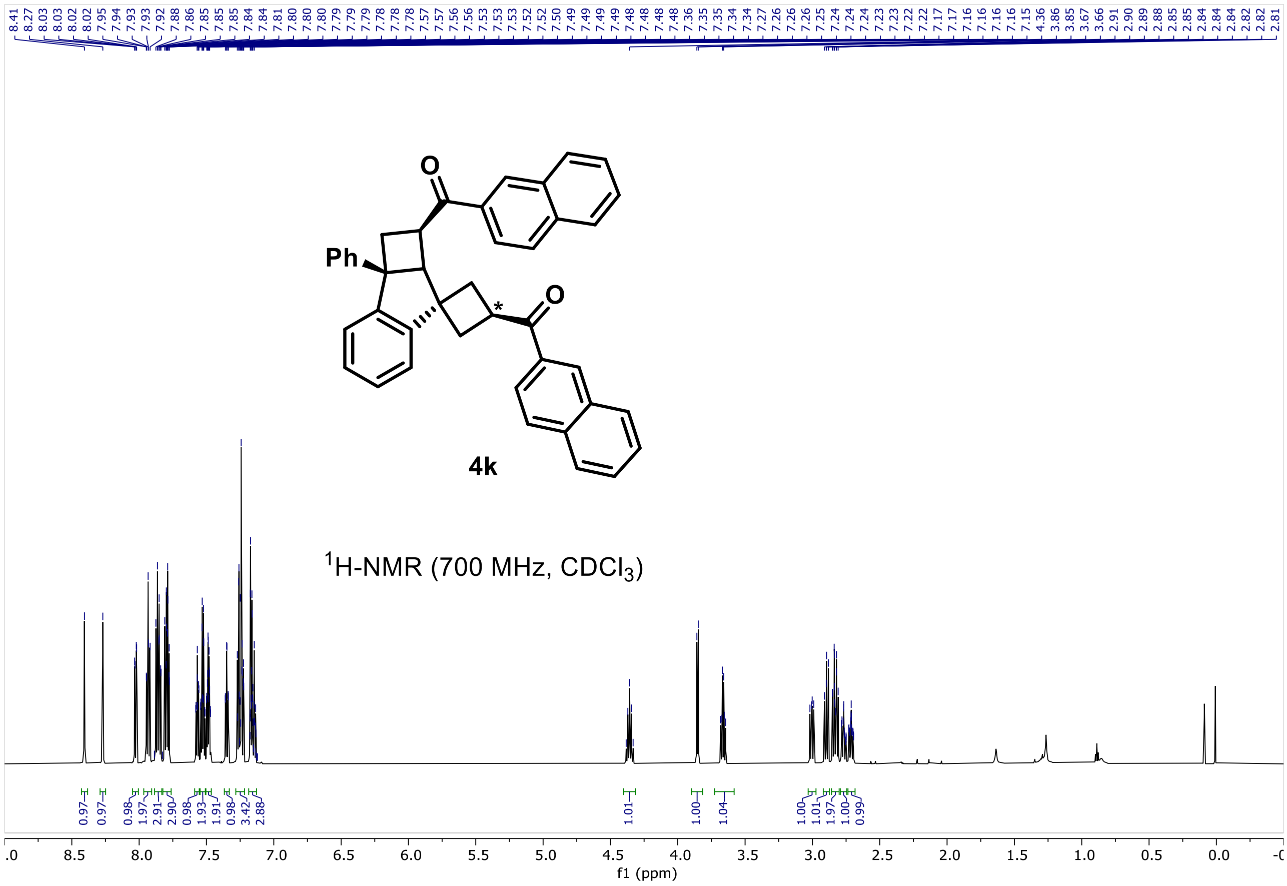
Ph
O
O
*
4k
1H-NMR (700 MHz, CDCl3)
f1 (ppm)

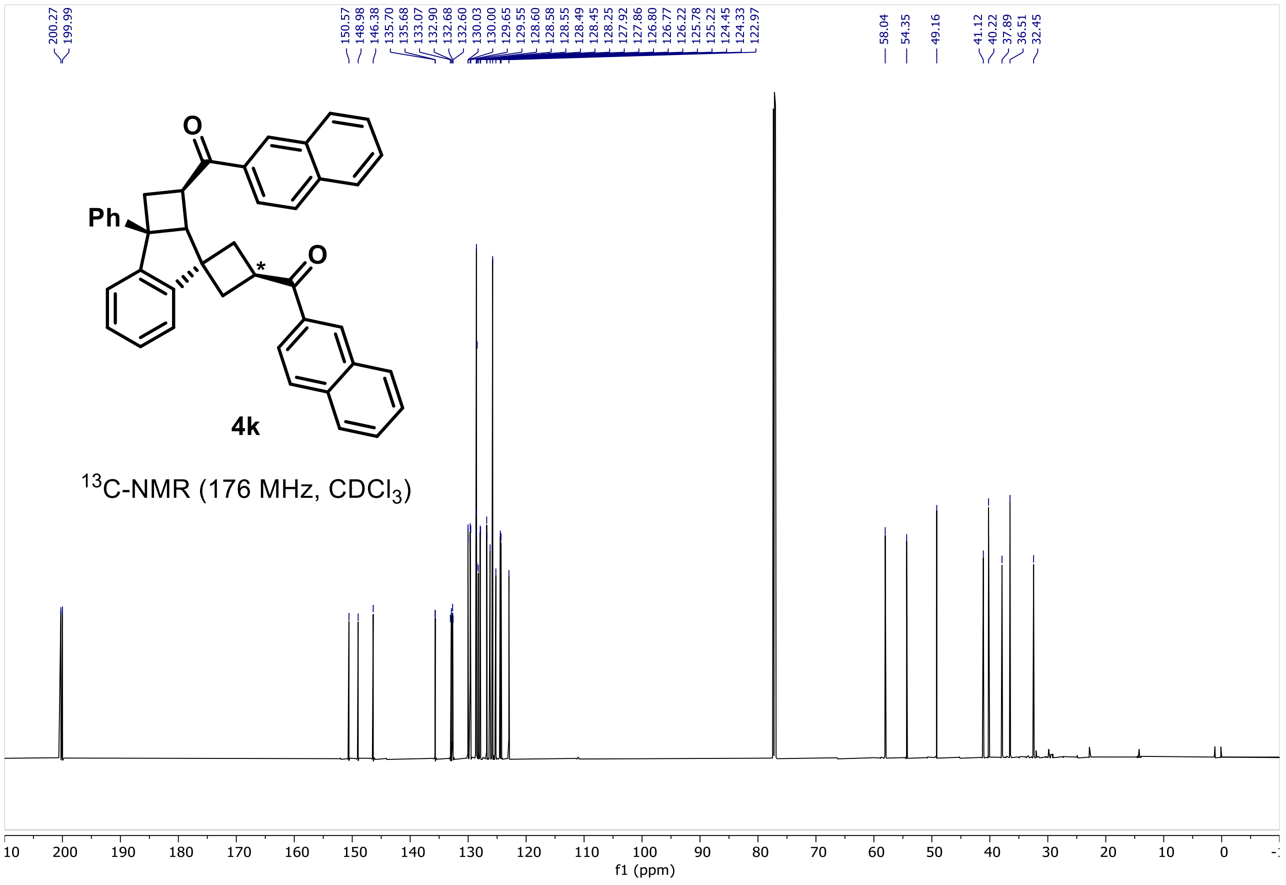
200.27
199.99
150.57
148.98
146.38
135.70
135.68
133.07
132.90
132.68
132.60
130.03
130.00
129.65
129.55
128.60
128.58
128.55
128.49
128.45
128.25
127.92
127.86
126.80
126.77
126.22
125.78
125.22
124.45
124.33
122.97
58.04
54.35
49.16
41.12
40.22
37.89
36.51
32.45
Ph
O
O
*
4k
13C-NMR (176 MHz, CDCl3)
f1 (ppm)

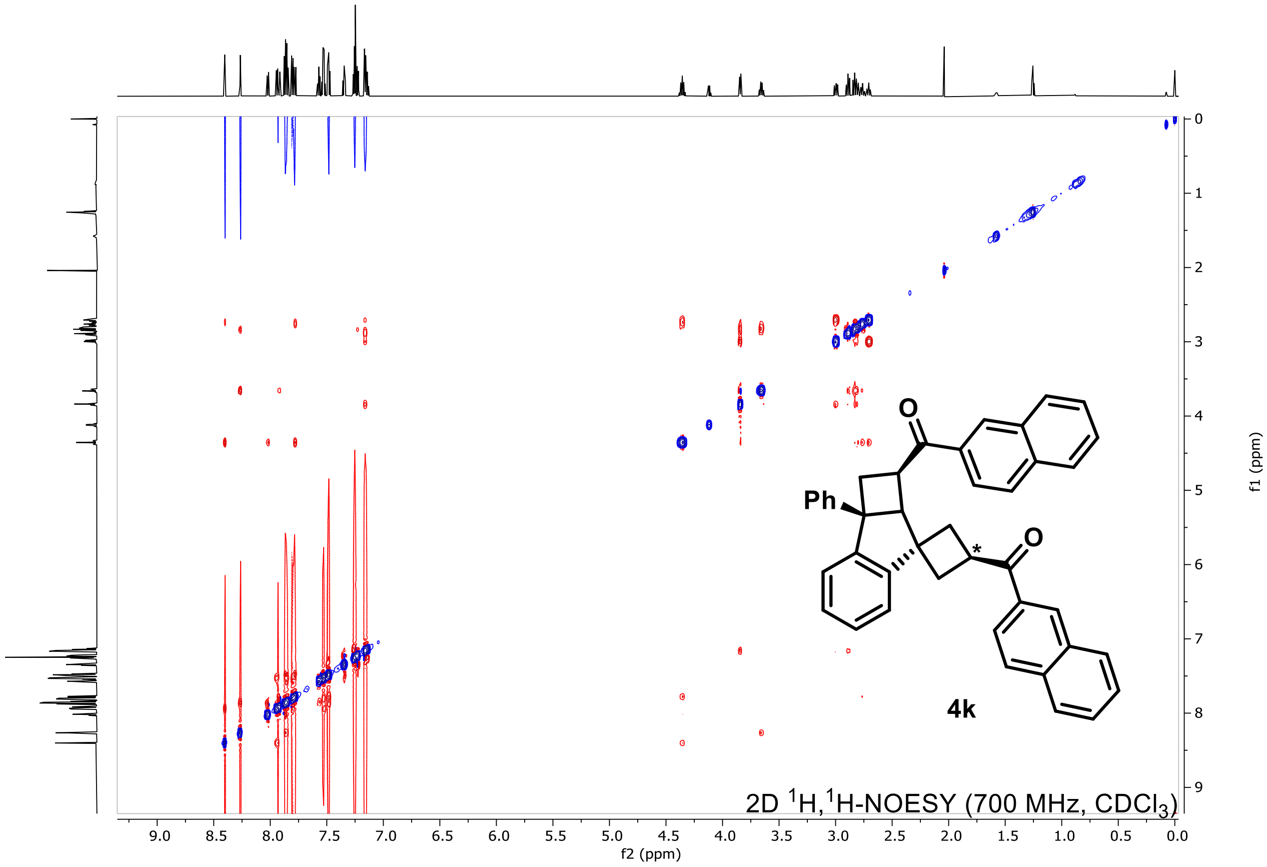
Ph
O
O
*
4k
2D ^{1}H,^{1}H-NOESY (700 MHz, CDCl$_3$)
f2 (ppm)
f1 (ppm)
9.0
8.5
8.0
7.5
7.0
6.5
6.0
5.5
5.0
4.5
4.0
3.5
3.0
2.5
2.0
1.5
1.0
0.5
0.0
0
1
2
3
4
5
6
7
8
9

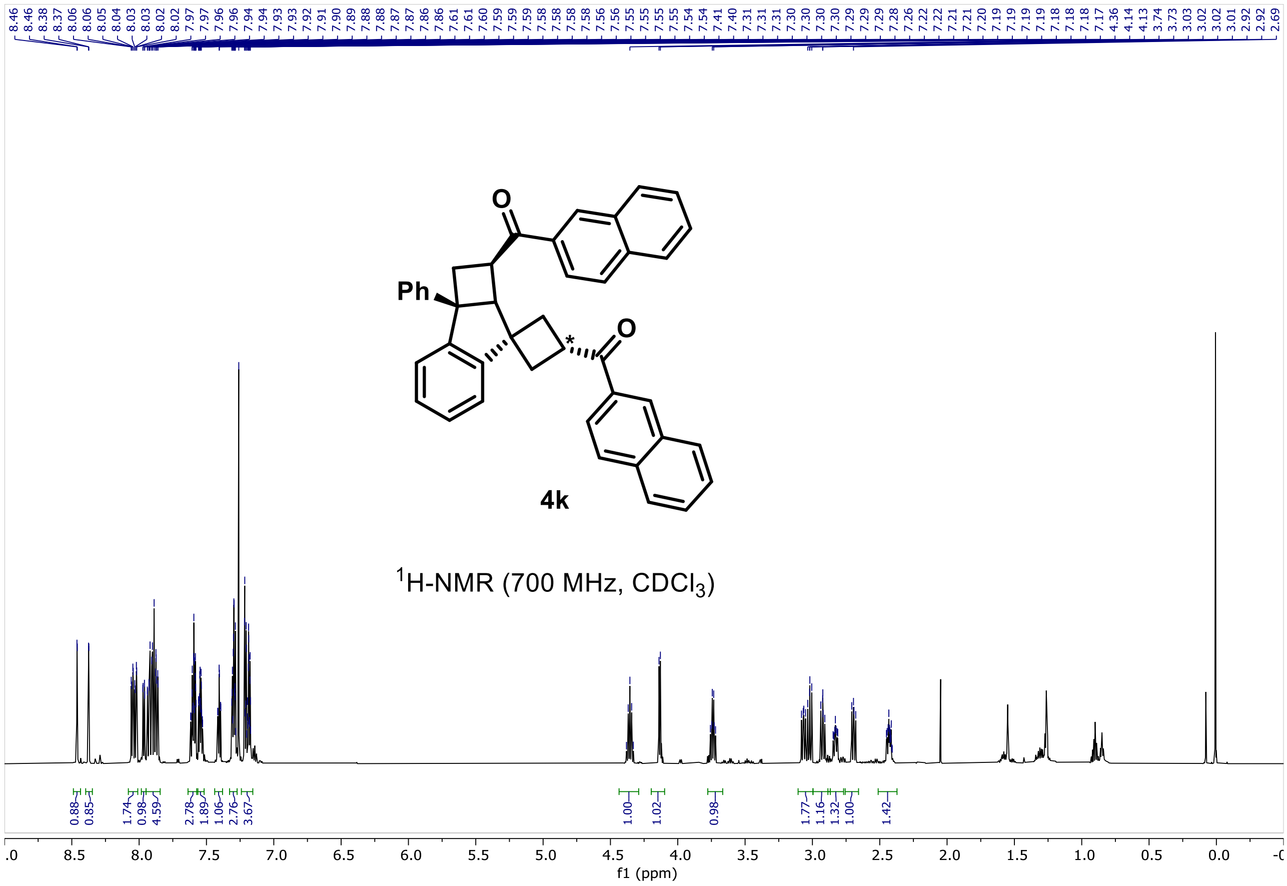
Ph
4k
1H-NMR (700 MHz, CDCl3)
f1 (ppm)

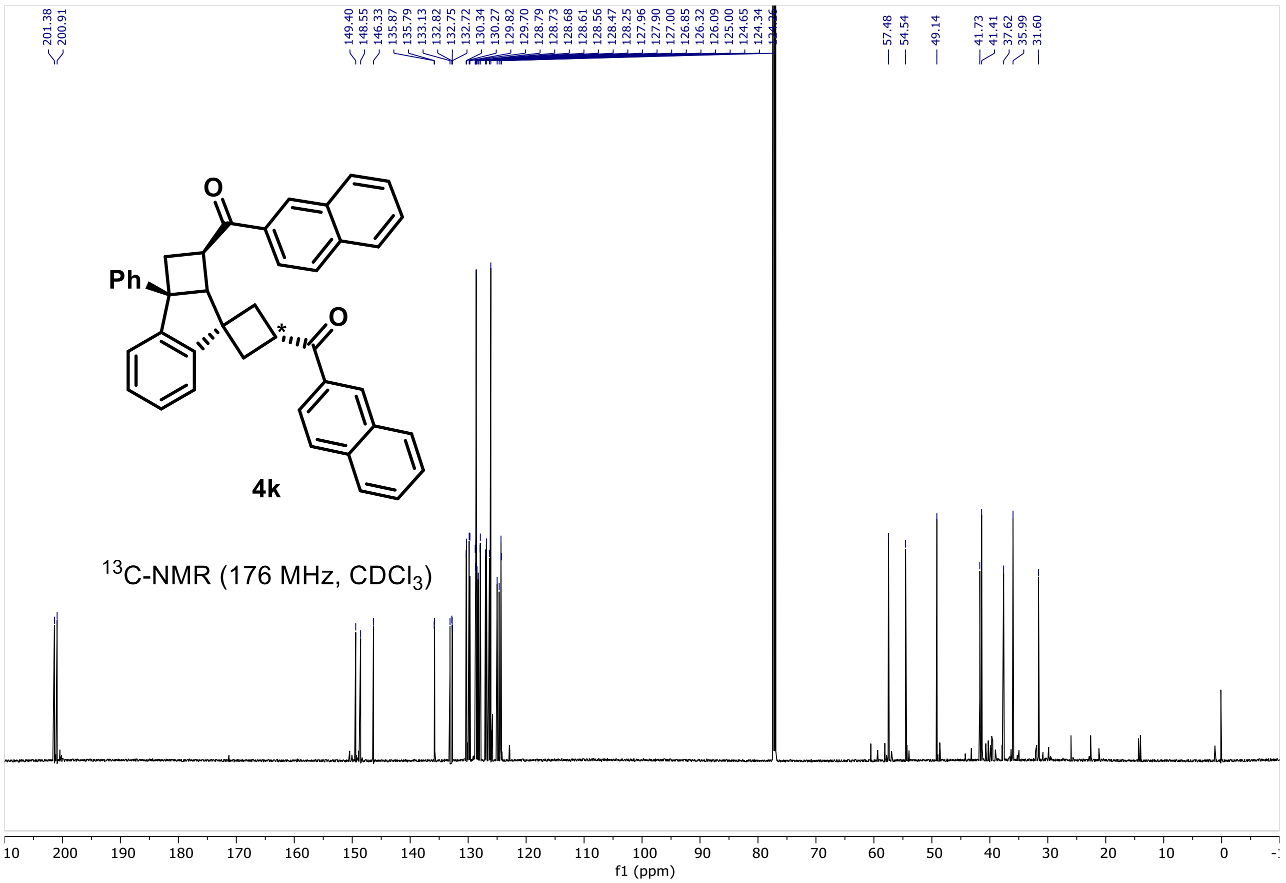
201.38
200.91
149.40
148.55
146.33
135.87
135.79
133.13
132.82
132.75
132.72
130.34
130.27
129.82
129.70
128.79
128.73
128.68
128.61
128.56
128.47
128.25
127.96
127.90
127.00
126.85
126.32
126.09
125.00
124.65
124.34
57.48
54.54
49.14
41.73
41.41
37.62
35.99
31.60
Ph
4k
13C-NMR (176 MHz, CDCl3)
f1 (ppm)

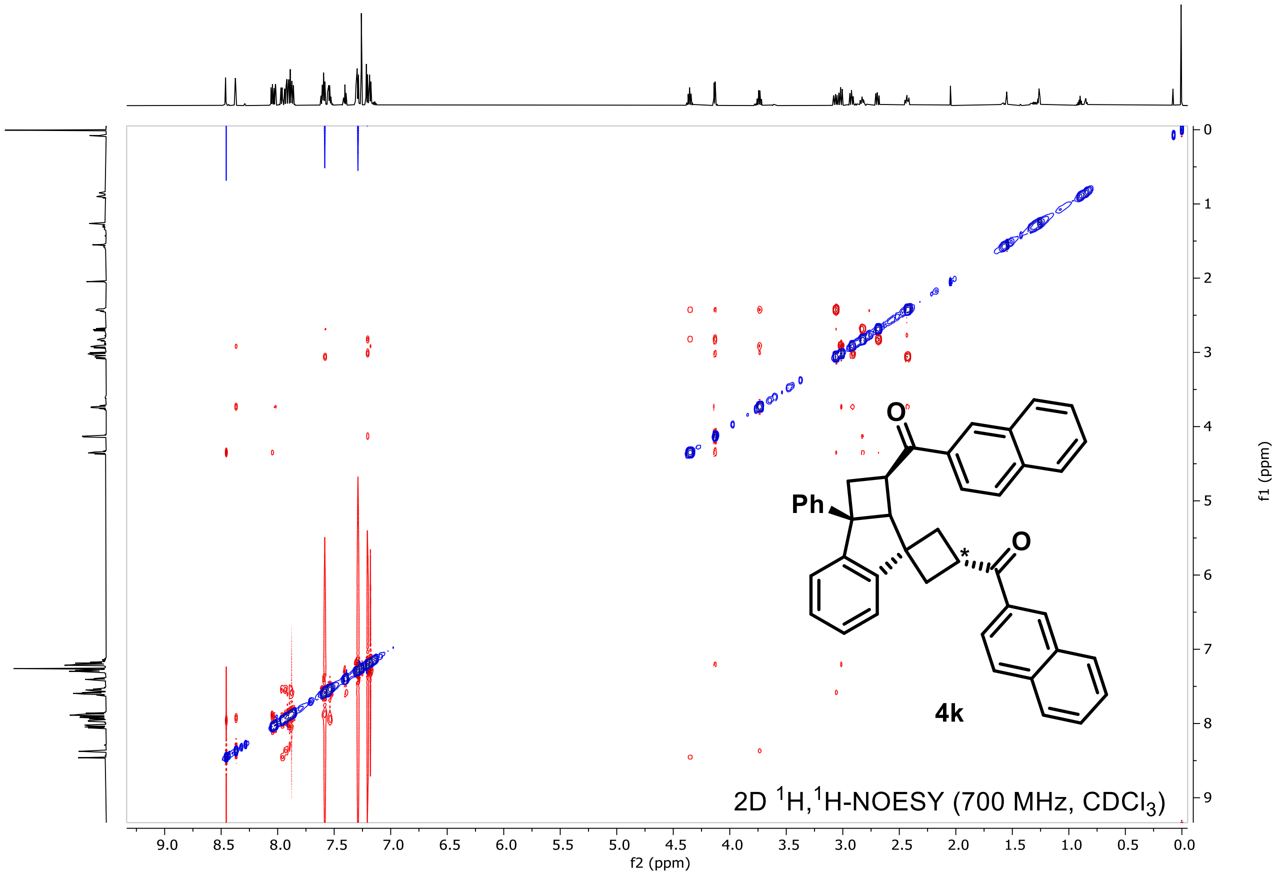

2D $^1H,^1H$-NOESY (700 MHz, $CDCl_3$)
Ph
O
O
*
4k
f2 (ppm)
f1 (ppm)
9.0
8.5
8.0
7.5
7.0
6.5
6.0
5.5
5.0
4.5
4.0
3.5
3.0
2.5
2.0
1.5
1.0
0.5
0.0
0
1
2
3
4
5
6
7
8
9

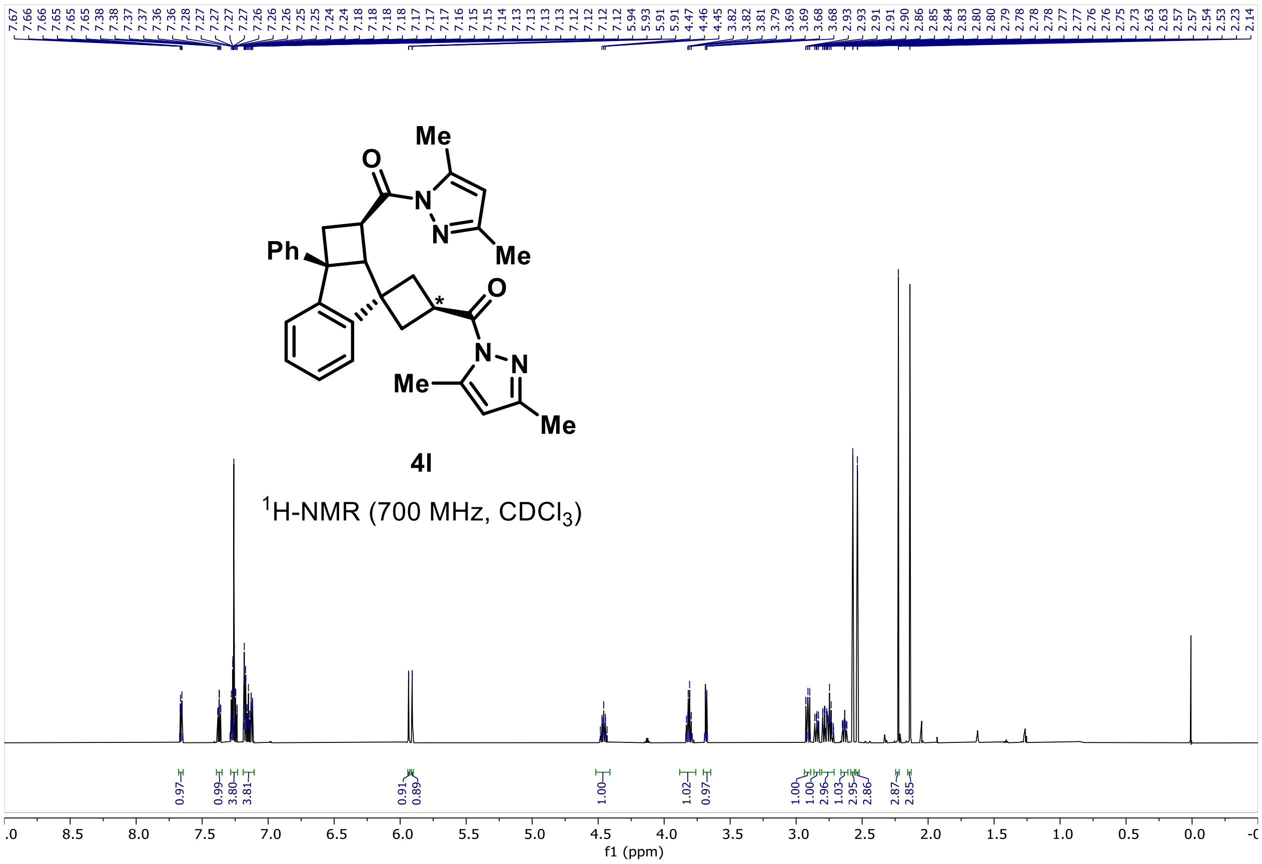

Me
O
N
N
Ph
Me
*
O
N
N
Me
Me
4l
^{1}H-NMR (700 MHz, CDCl$_3$)
f1 (ppm)

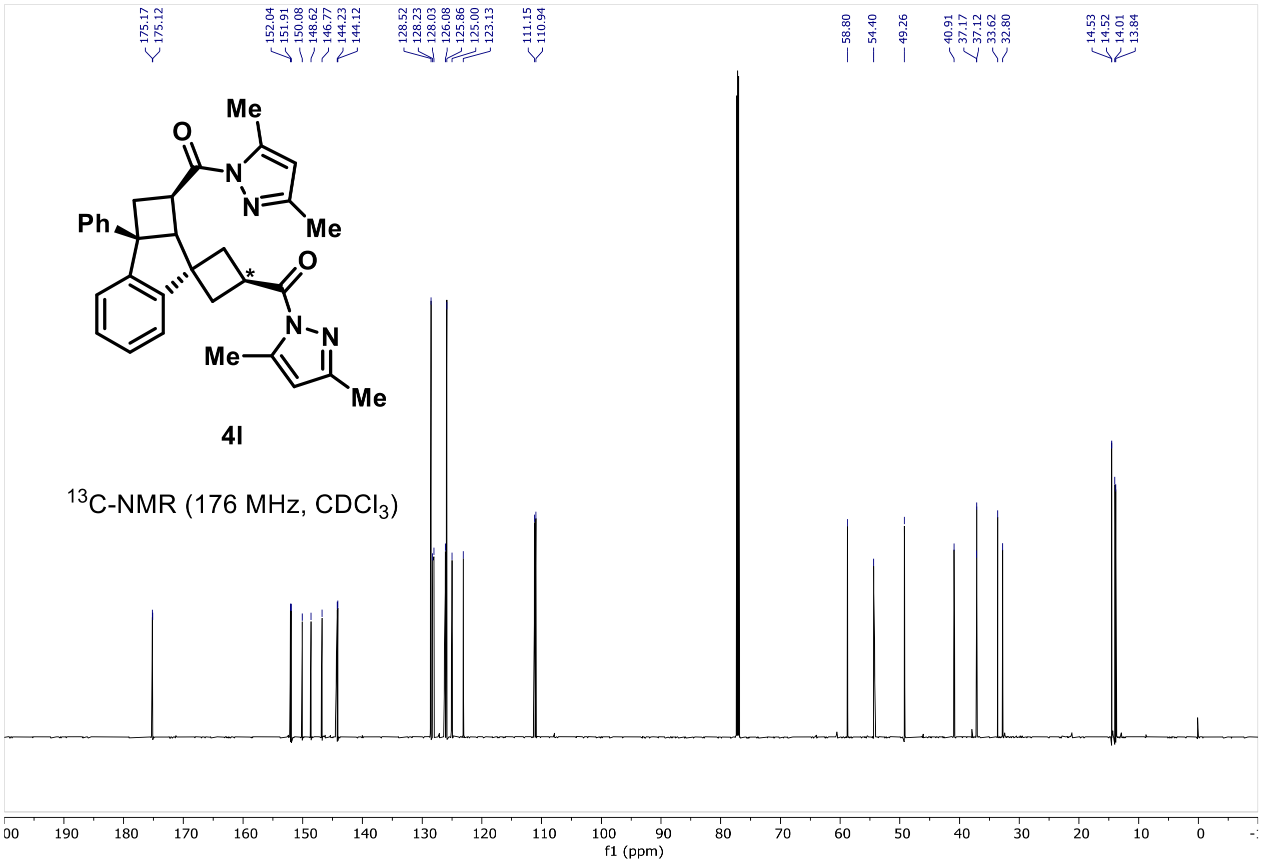

175.17
175.12
152.04
151.91
150.08
148.62
146.77
144.23
144.12
128.52
128.23
128.03
126.08
125.86
125.00
123.13
111.15
110.94
58.80
54.40
49.26
40.91
37.17
37.12
33.62
32.80
14.53
14.52
14.01
13.84
4l
^{13}C-NMR (176 MHz, CDCl$_3$)
f1 (ppm)

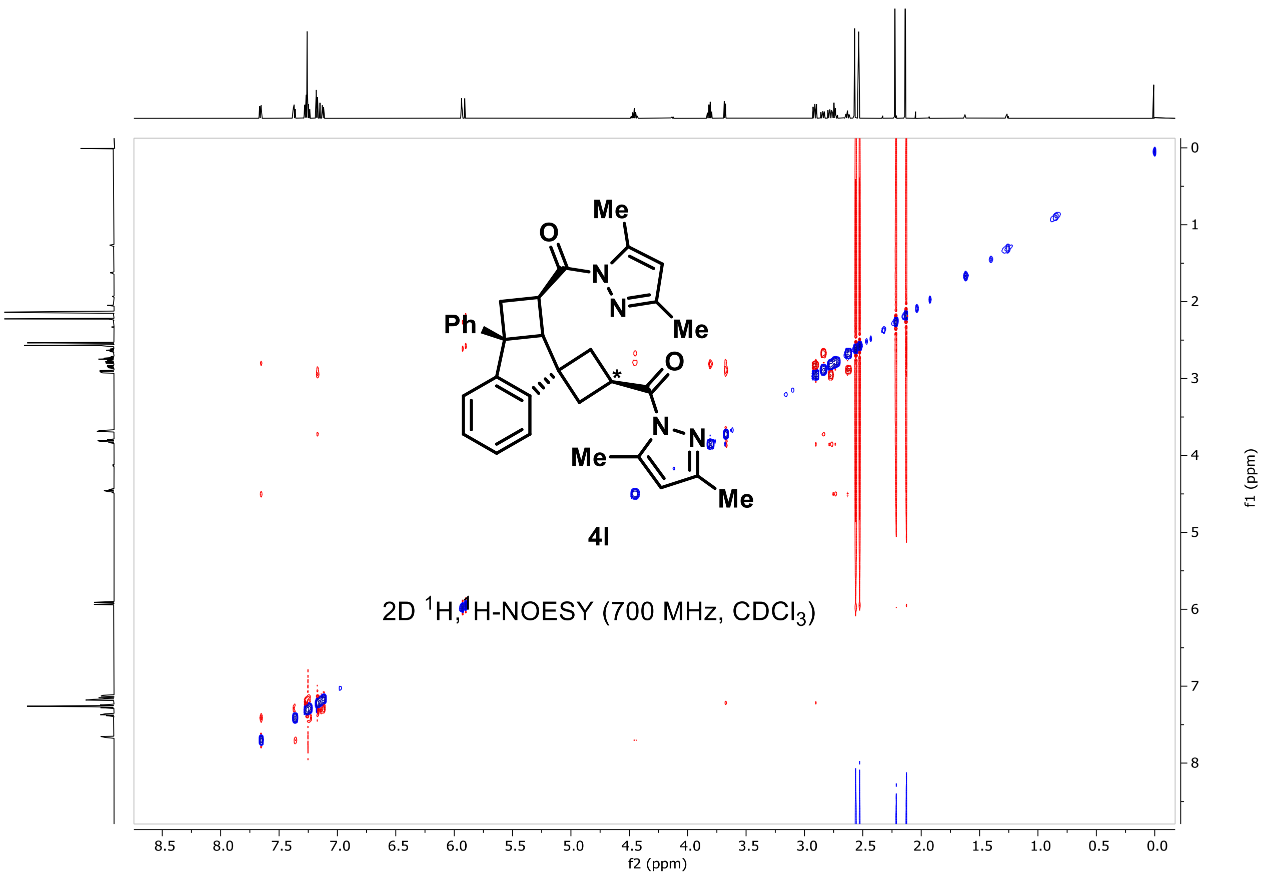
Me
O
N
N
Me
Ph
*
O
N
N
Me
Me
4l
2D ¹H,¹H-NOESY (700 MHz, CDCl₃)
f1 (ppm)
f2 (ppm)

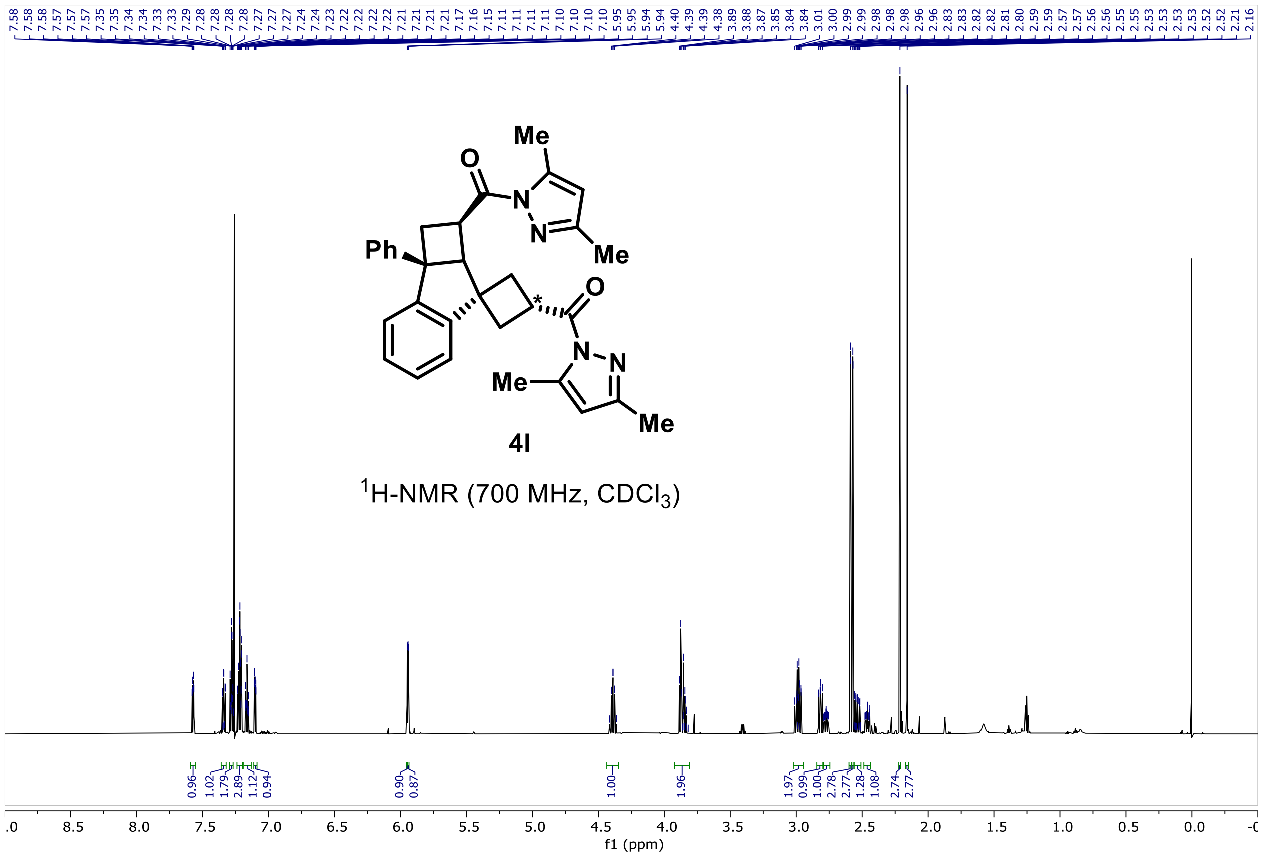
4l
^{1}H-NMR (700 MHz, CDCl$_3$)
f1 (ppm)

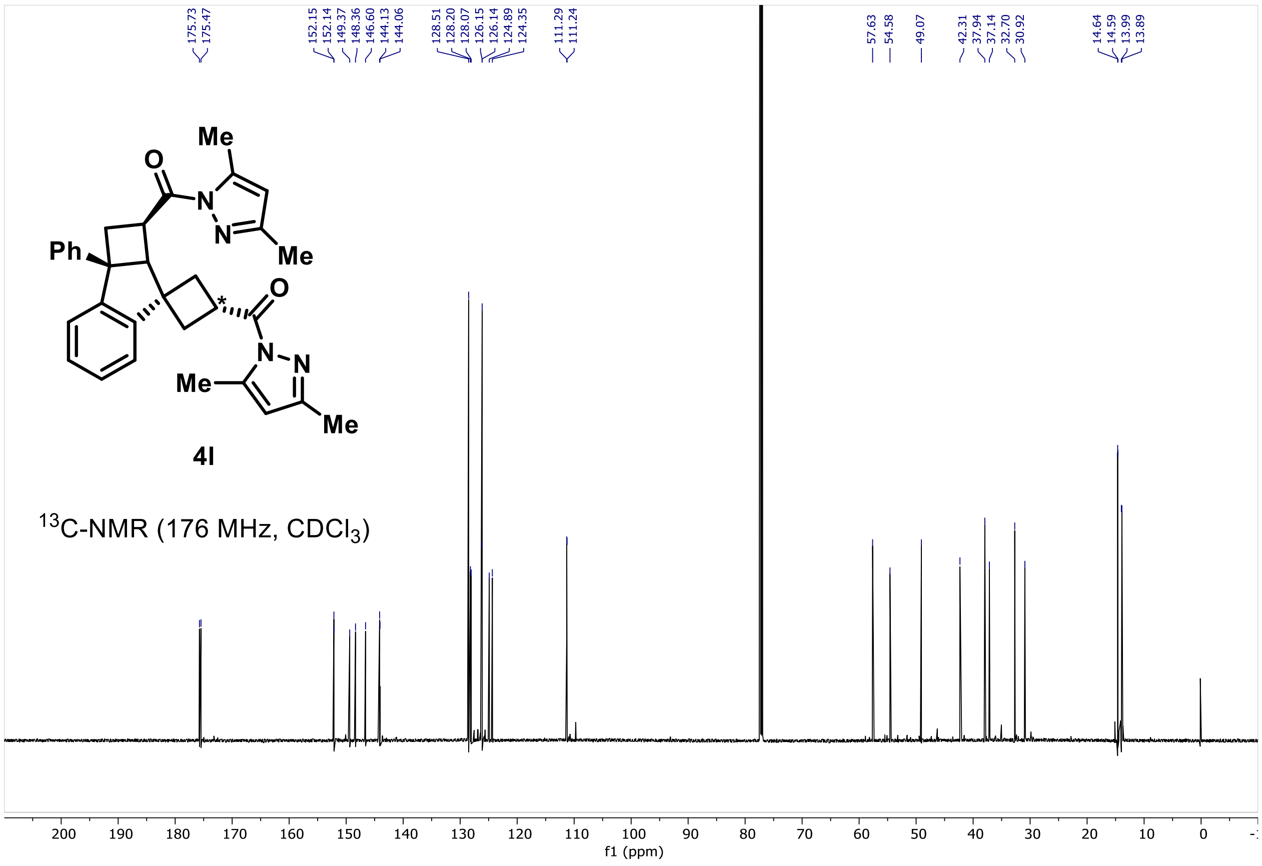
4l
^{13}C-NMR (176 MHz, CDCl$_3$)
f1 (ppm)

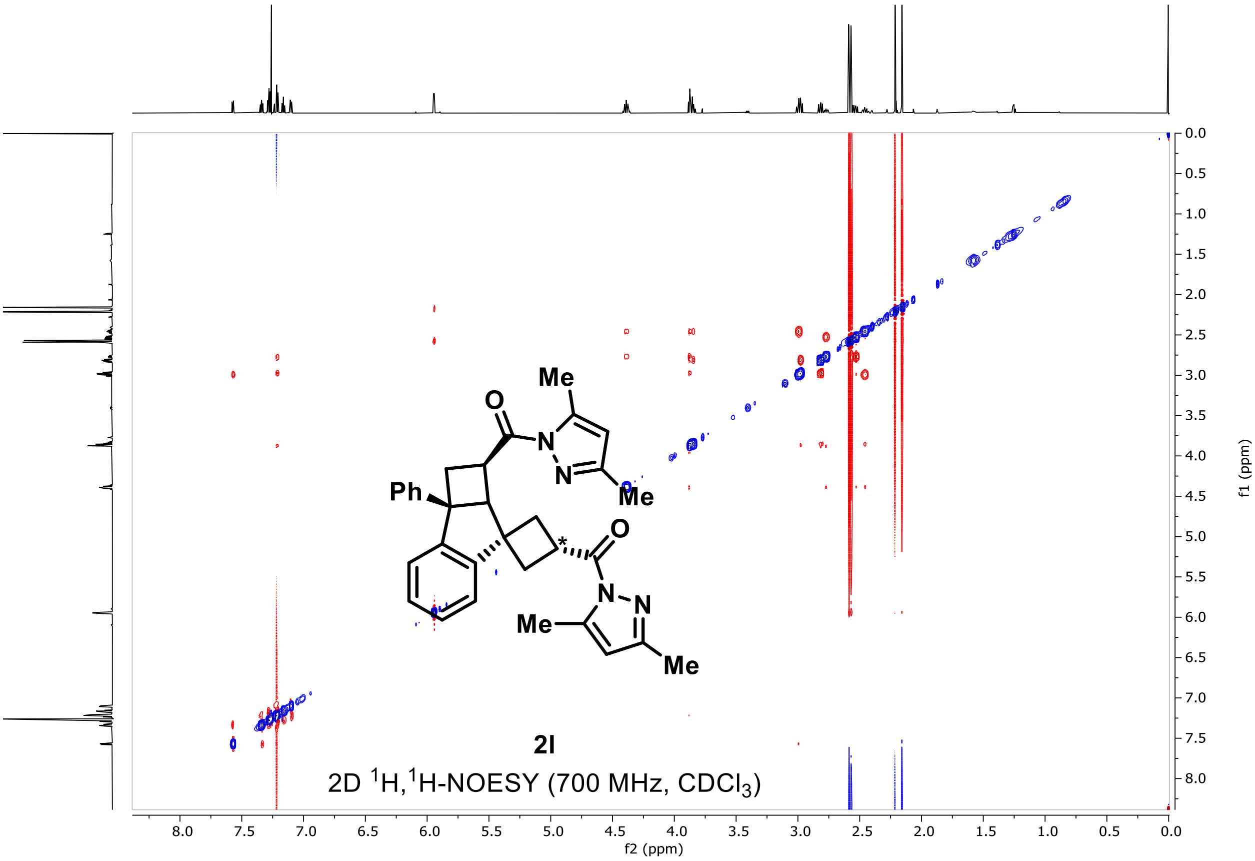

2l

2D $^{1}H,^{1}H$-NOESY (700 MHz, $CDCl_3$)

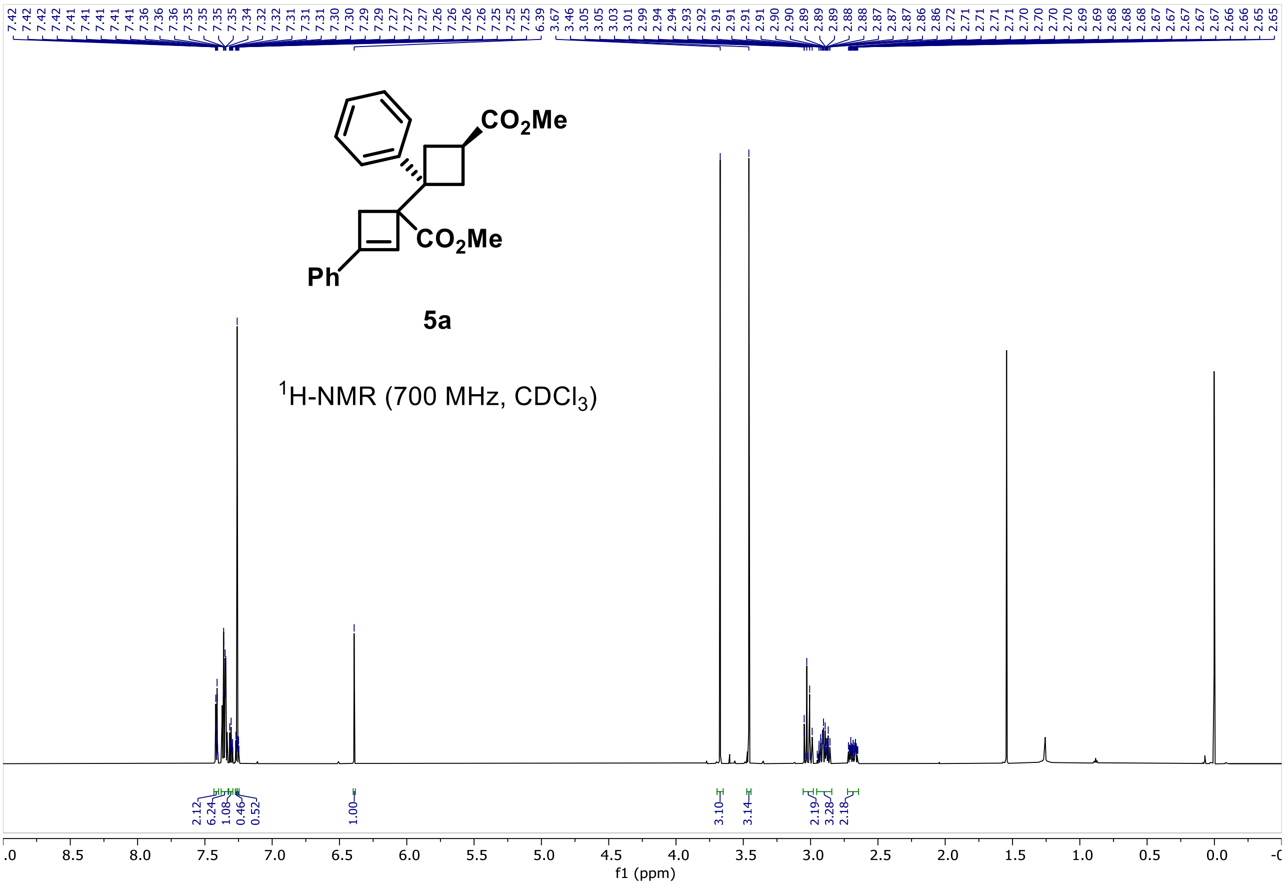
CO2Me
CO2Me
Ph
5a
1H-NMR (700 MHz, CDCl3)
f1 (ppm)

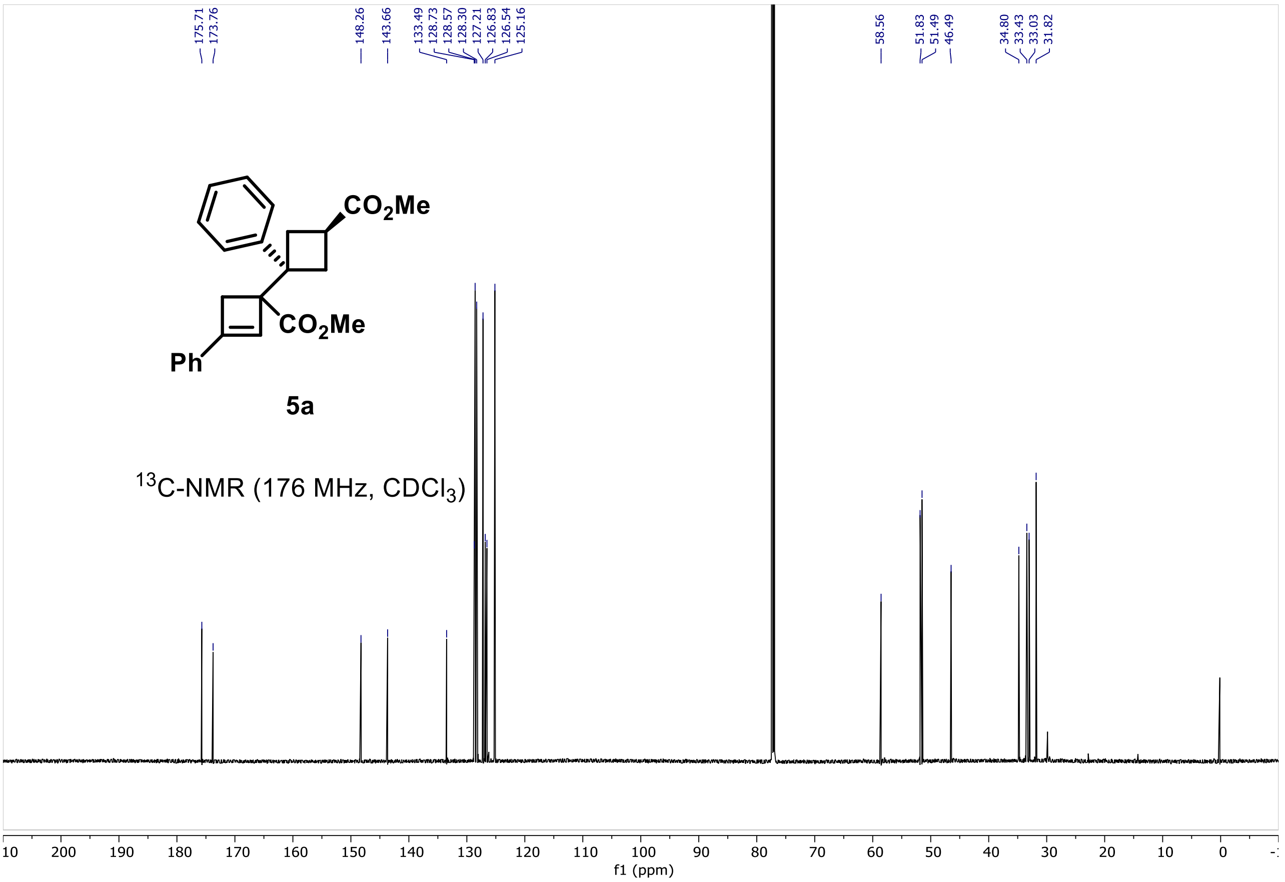
175.71
173.76
148.26
143.66
133.49
128.73
128.57
128.30
127.21
126.83
126.54
125.16
58.56
51.83
51.49
46.49
34.80
33.43
33.03
31.82
CO2Me
CO2Me
Ph
5a
13C-NMR (176 MHz, CDCl3)
f1 (ppm)

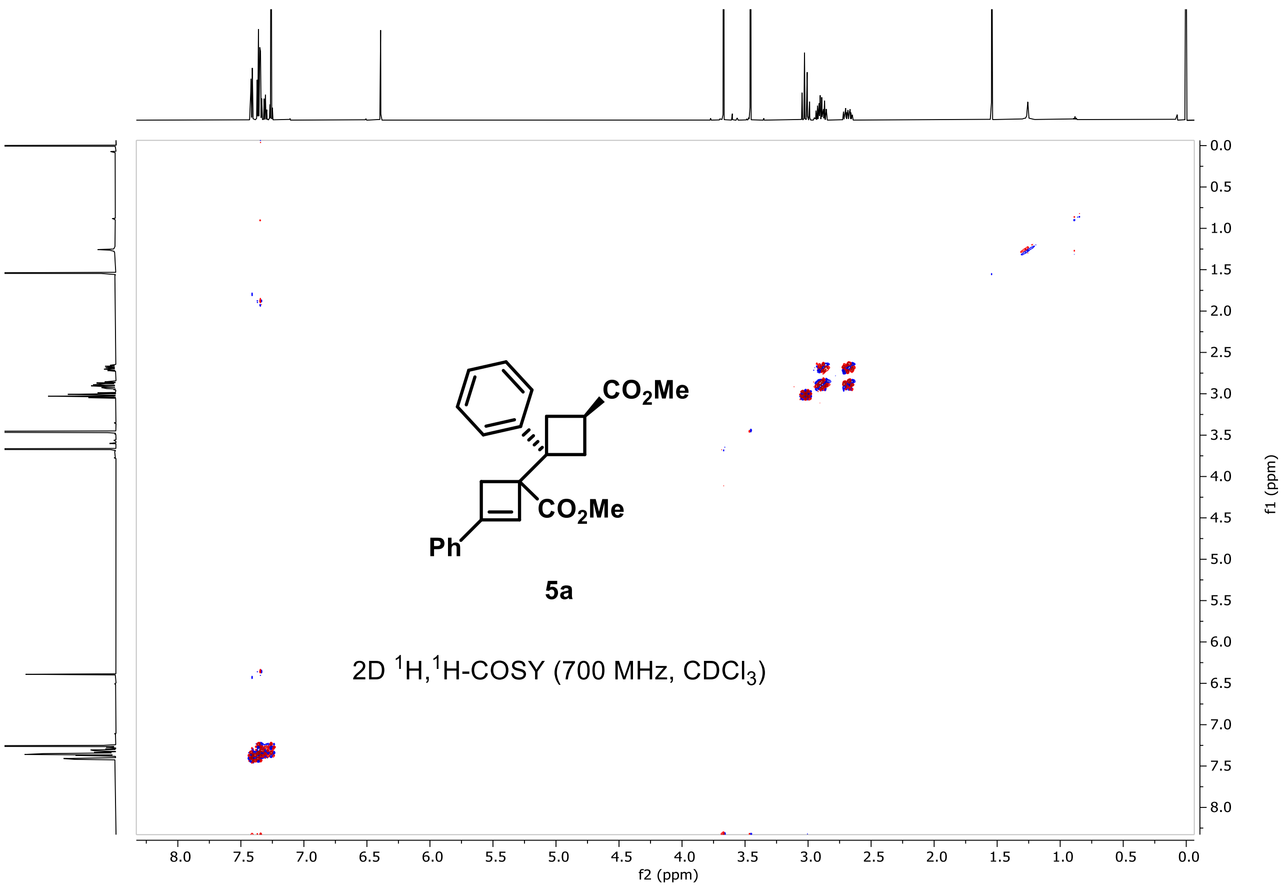
CO2Me
CO2Me
Ph
5a
2D 1H,1H-COSY (700 MHz, CDCl3)
f2 (ppm)
f1 (ppm)

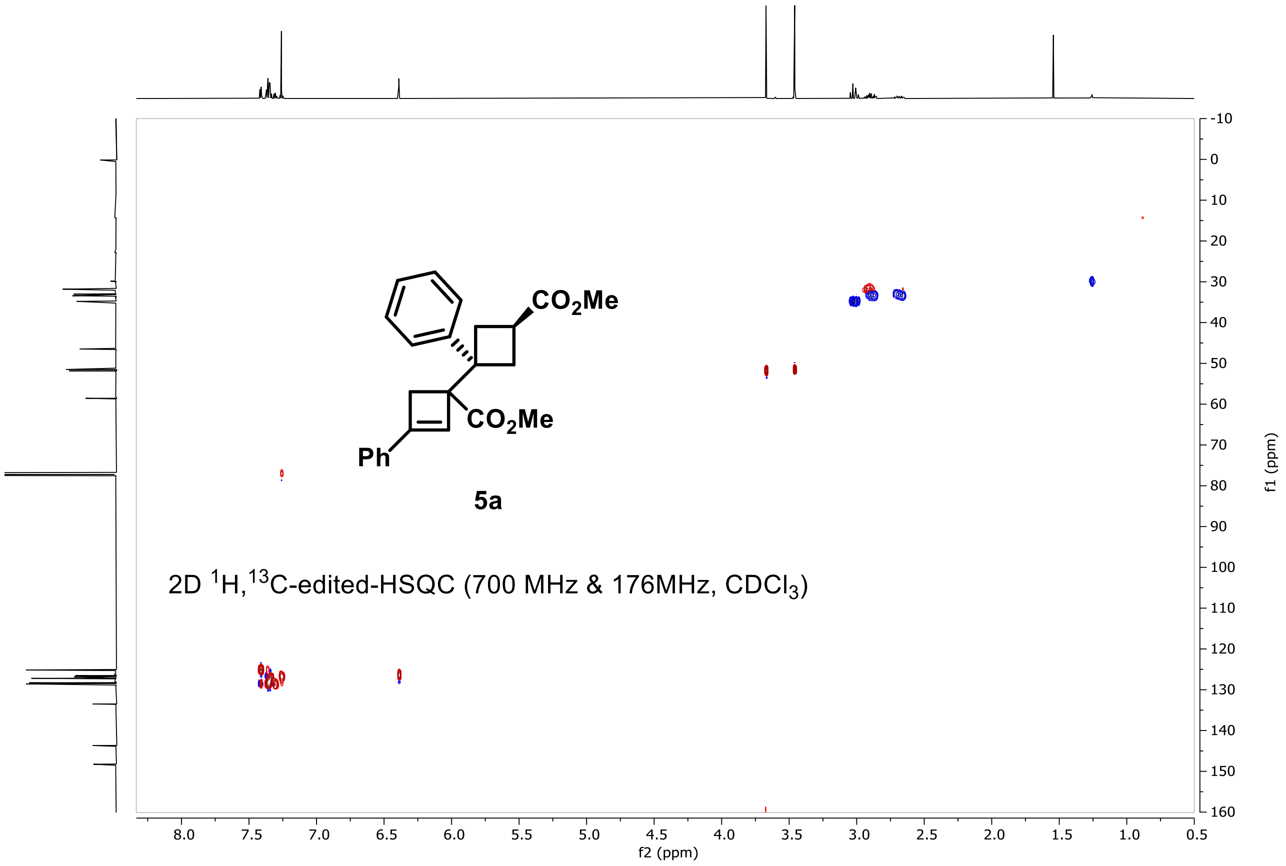
CO2Me
CO2Me
Ph
5a
2D 1H,13C-edited-HSQC (700 MHz & 176MHz, CDCl3)
f2 (ppm)
f1 (ppm)

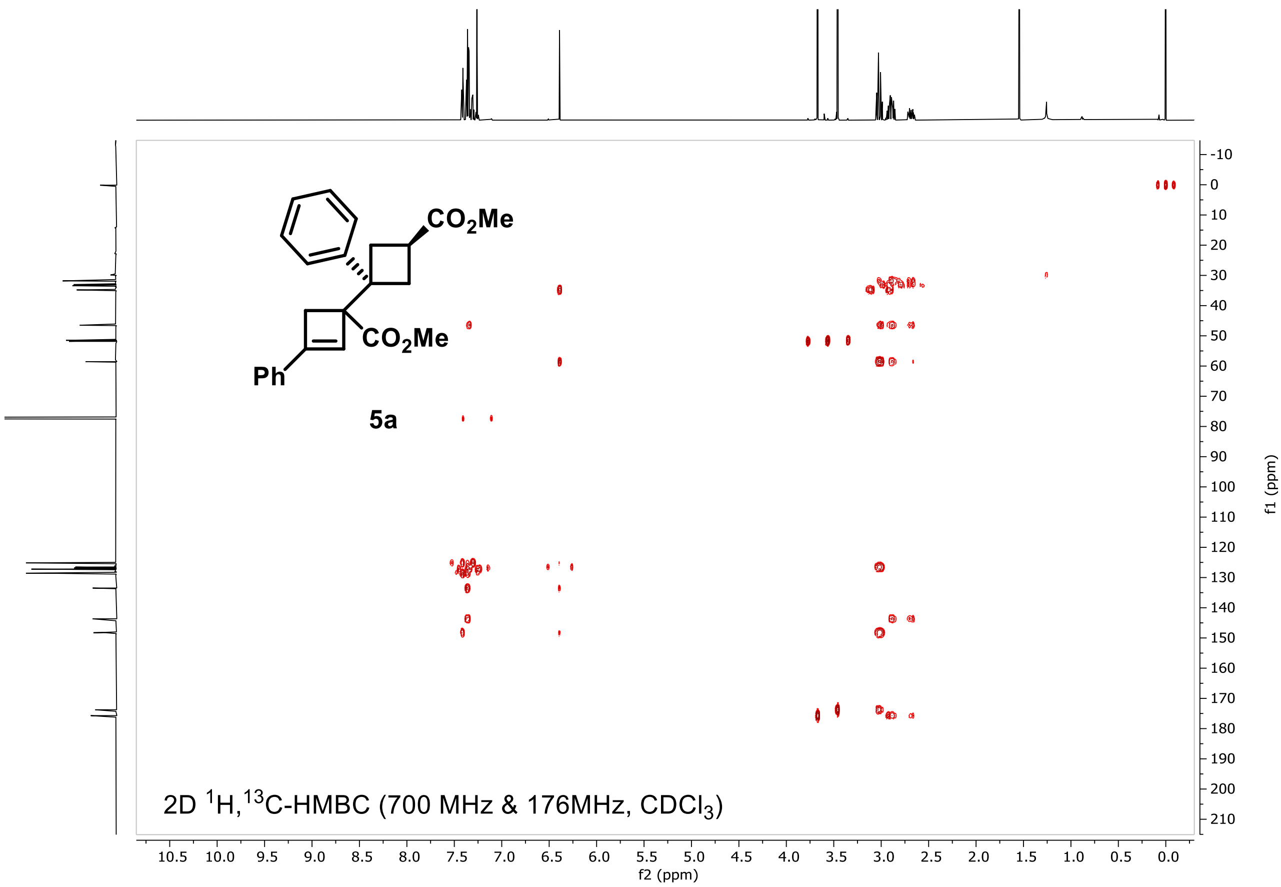
CO2Me
CO2Me
Ph
5a
2D 1H,13C-HMBC (700 MHz & 176MHz, CDCl3)
f1 (ppm)
f2 (ppm)

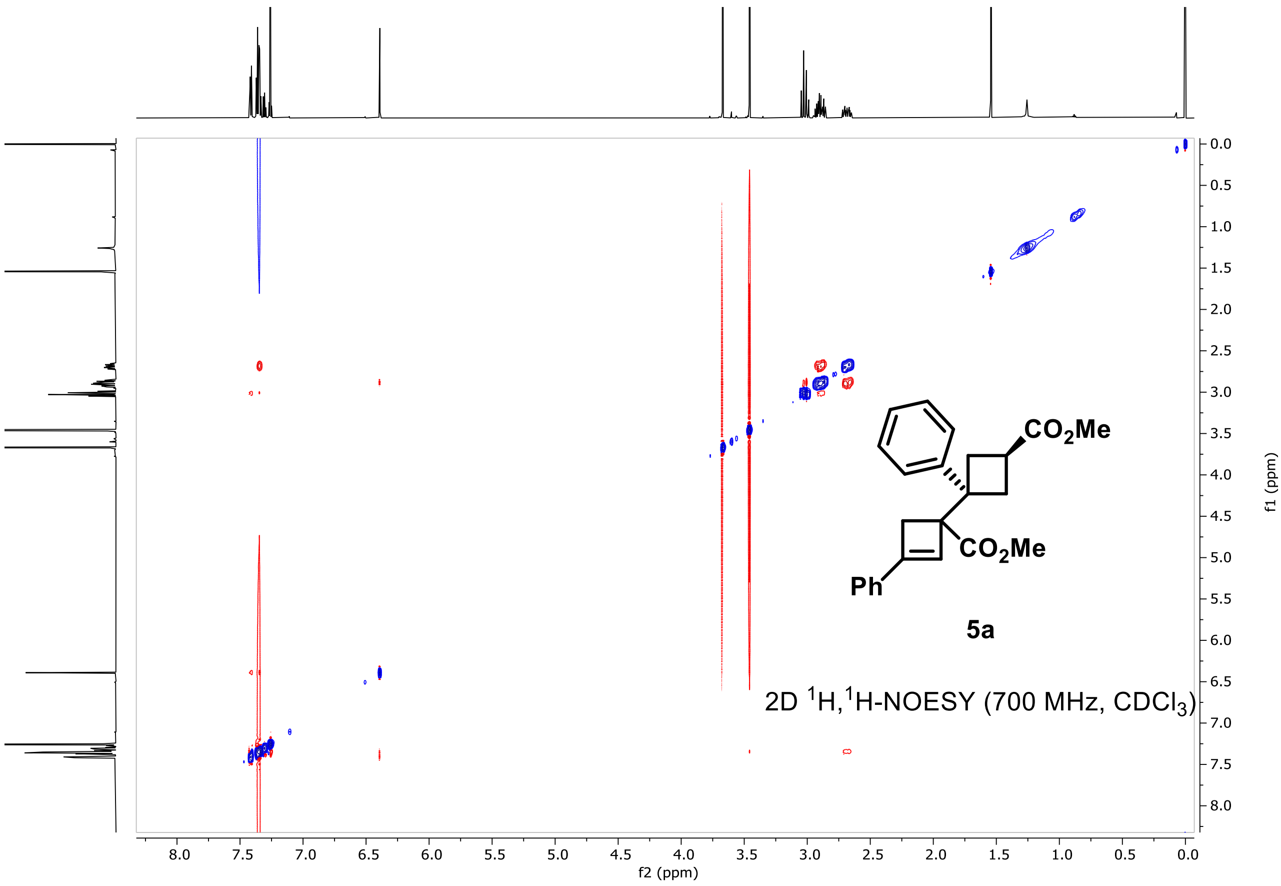
CO2Me
CO2Me
Ph
5a
2D 1H,1H-NOESY (700 MHz, CDCl3)
f1 (ppm)
f2 (ppm)

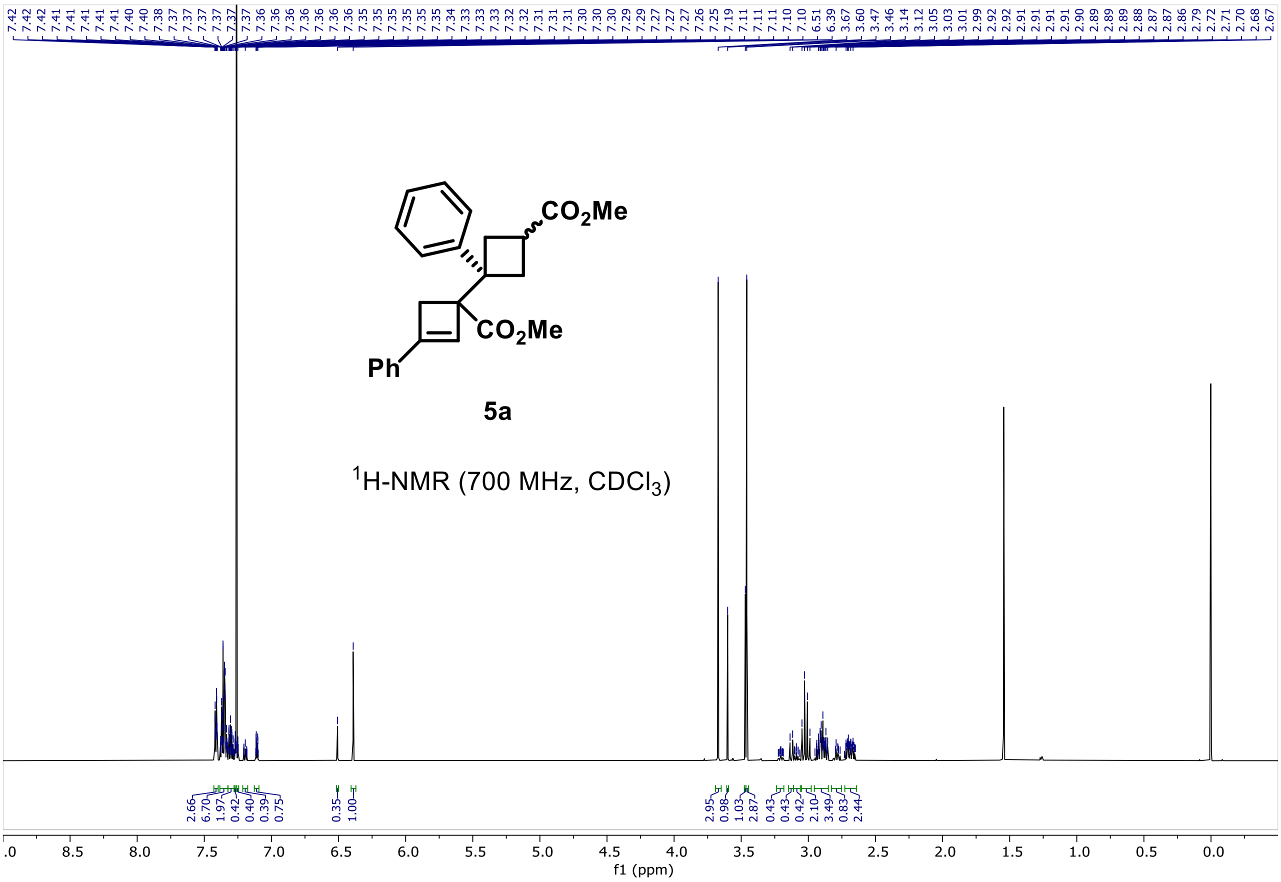

CO2Me
CO2Me
Ph
5a
1H-NMR (700 MHz, CDCl3)
f1 (ppm)

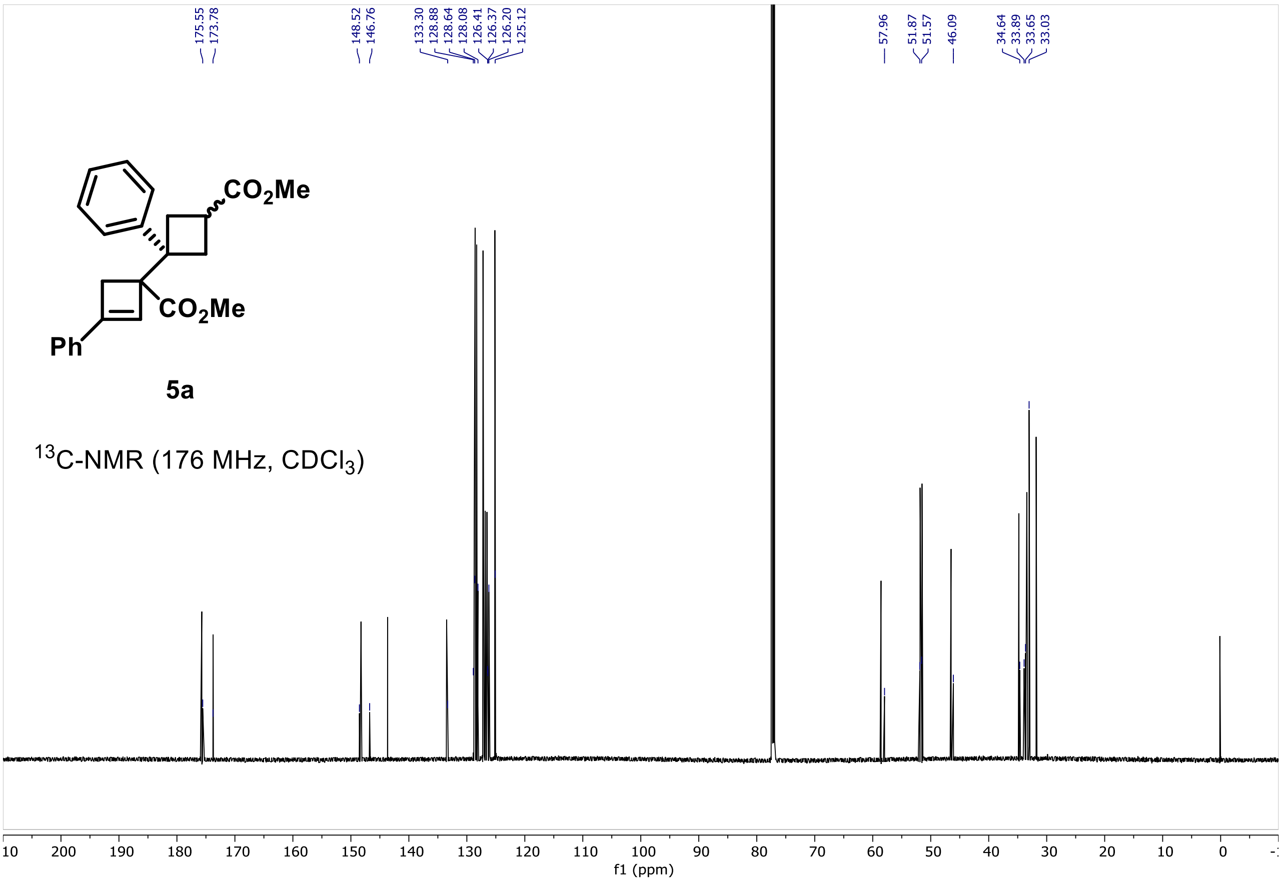

175.55
173.78
148.52
146.76
133.30
128.88
128.64
128.08
126.41
126.37
126.20
125.12
57.96
51.87
51.57
46.09
34.64
33.89
33.65
33.03
CO2Me
CO2Me
Ph
5a
13C-NMR (176 MHz, CDCl3)
f1 (ppm)

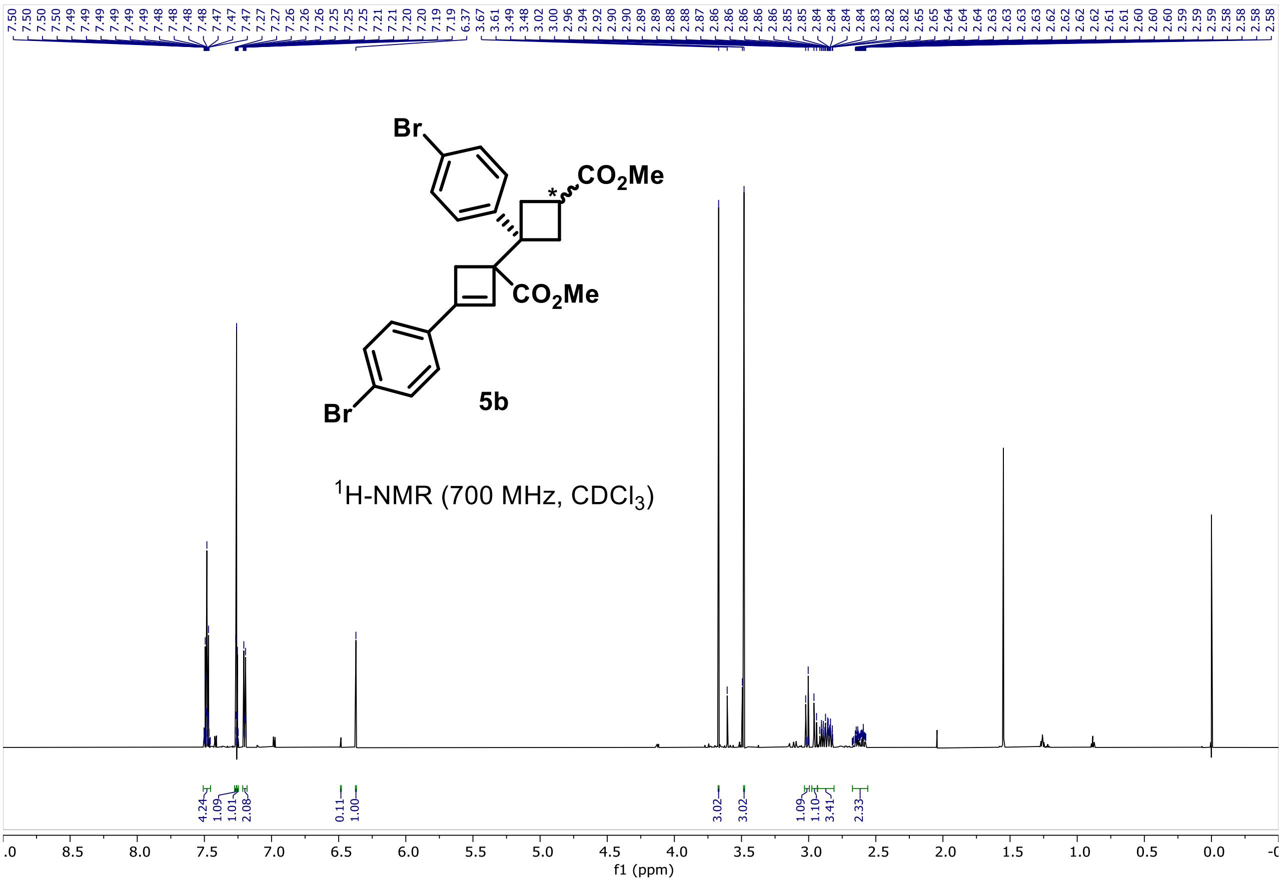
Br
CO2Me
CO2Me
Br
5b
1H-NMR (700 MHz, CDCl3)
4.24
1.09
1.01
2.08
0.11
1.00
3.02
3.02
1.09
1.10
3.41
2.33
f1 (ppm)

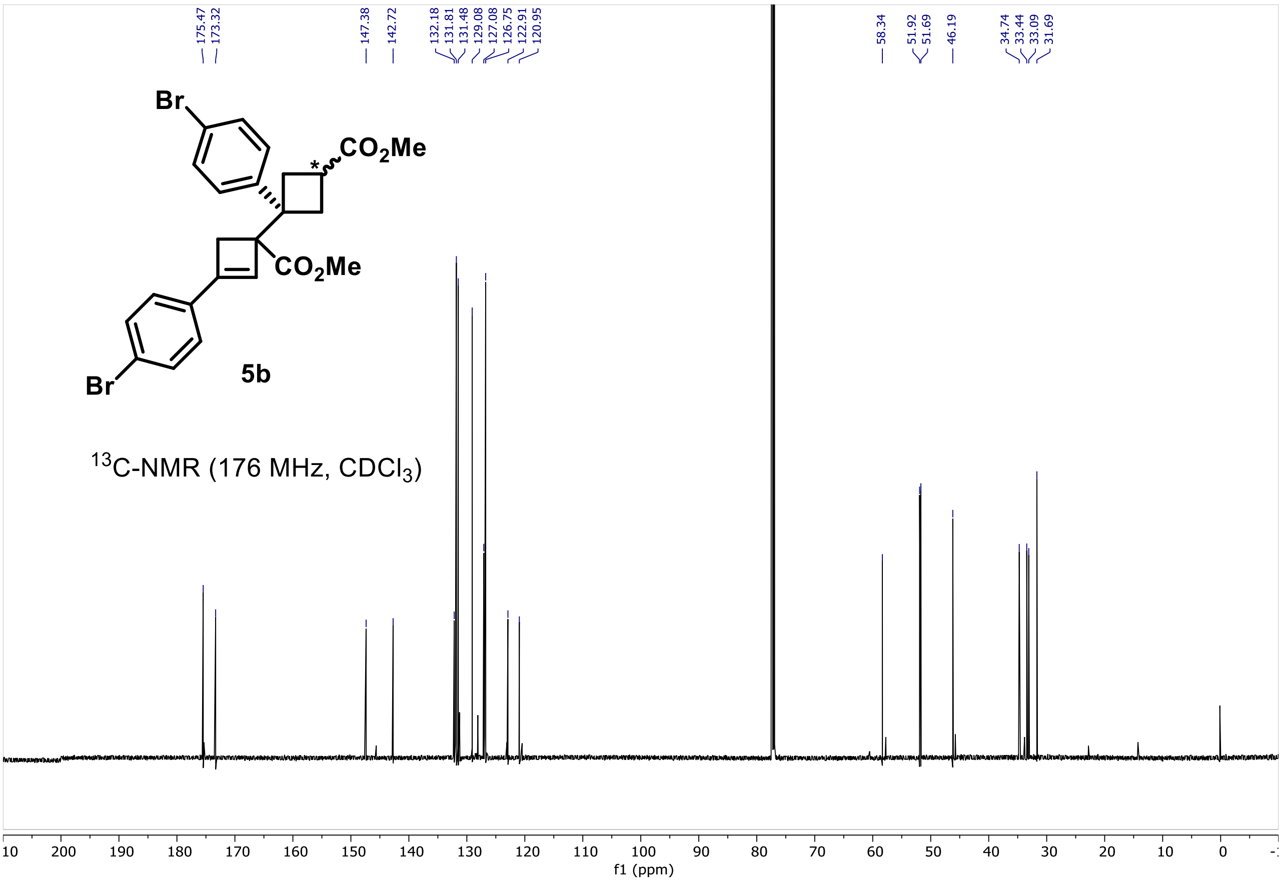
175.47
173.32
147.38
142.72
132.18
131.81
131.48
129.08
127.08
126.75
122.91
120.95
58.34
51.92
51.69
46.19
34.74
33.44
33.09
31.69
Br
CO2Me
CO2Me
Br
5b
13C-NMR (176 MHz, CDCl3)
f1 (ppm)

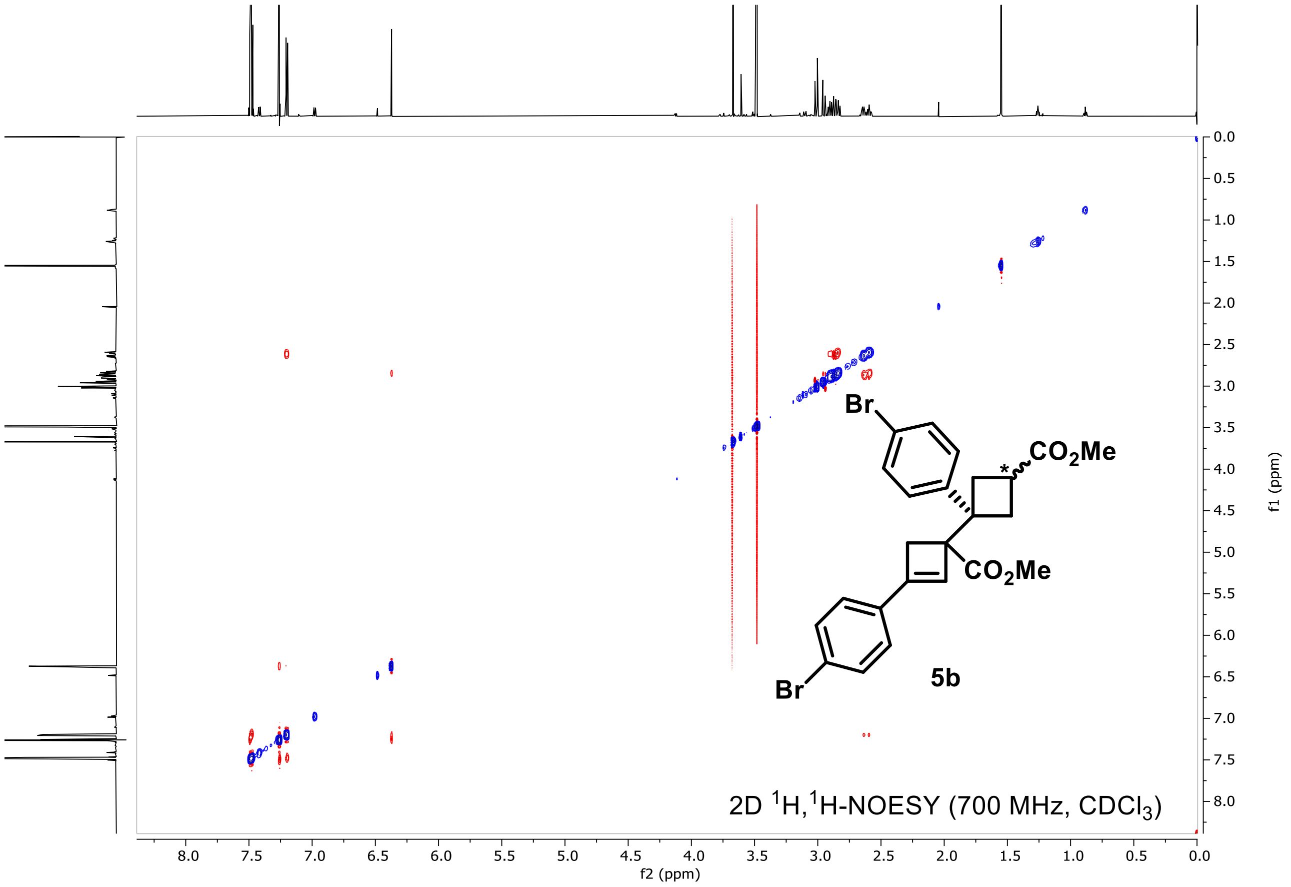
Br
CO2Me
CO2Me
Br
5b
2D 1H,1H-NOESY (700 MHz, CDCl3)
f2 (ppm)
f1 (ppm)

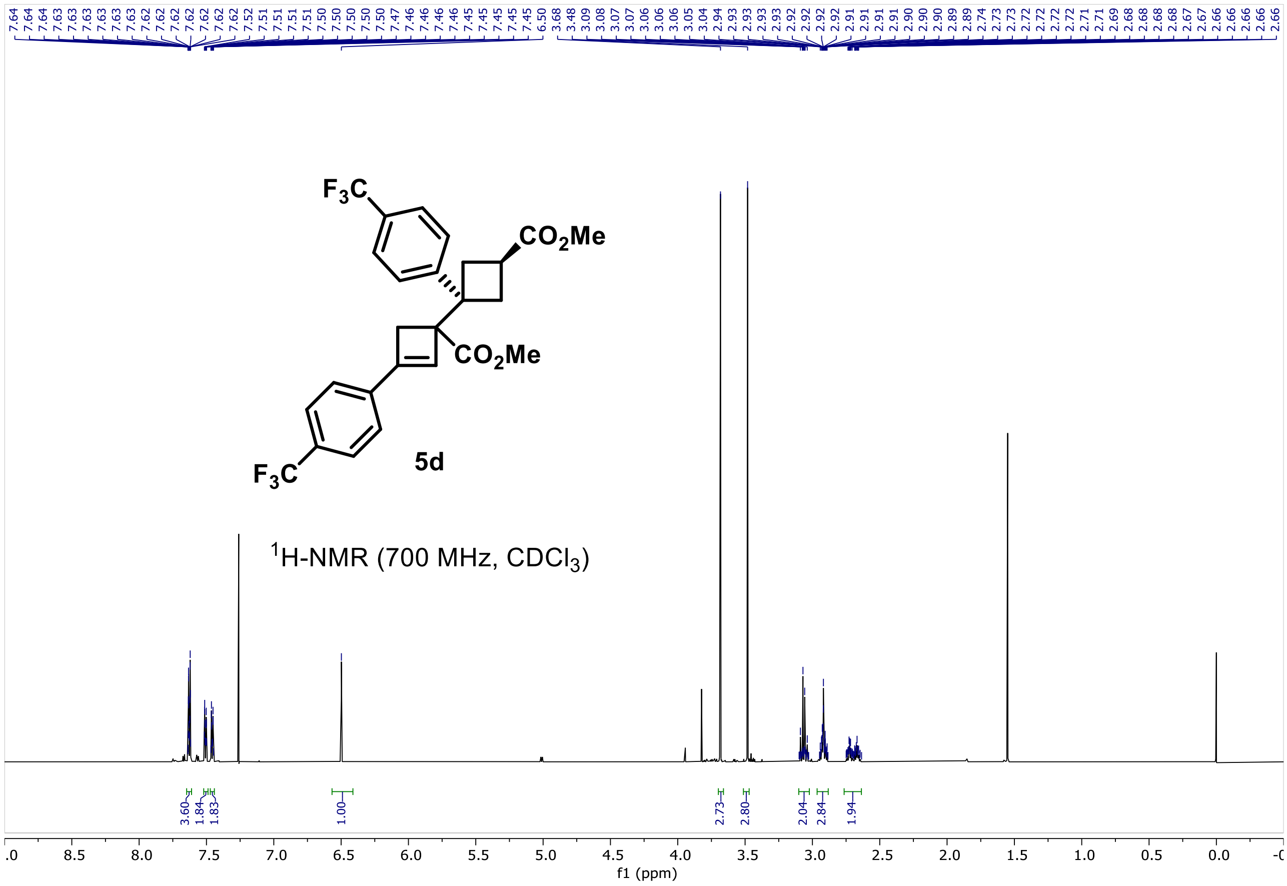
5d
^{1}H-NMR (700 MHz, CDCl$_3$)
f1 (ppm)

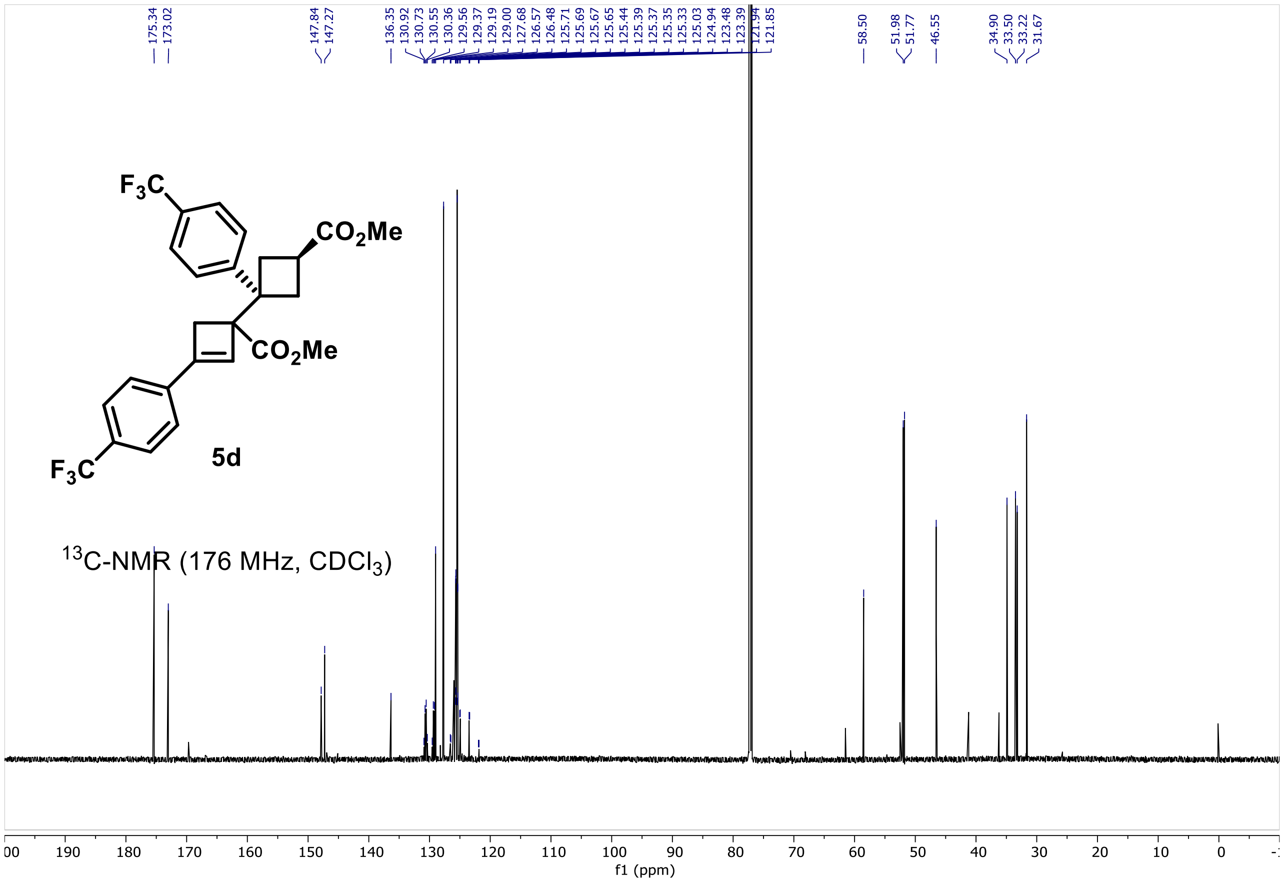
5d
^{13}C-NMR (176 MHz, CDCl$_3$)
f1 (ppm)

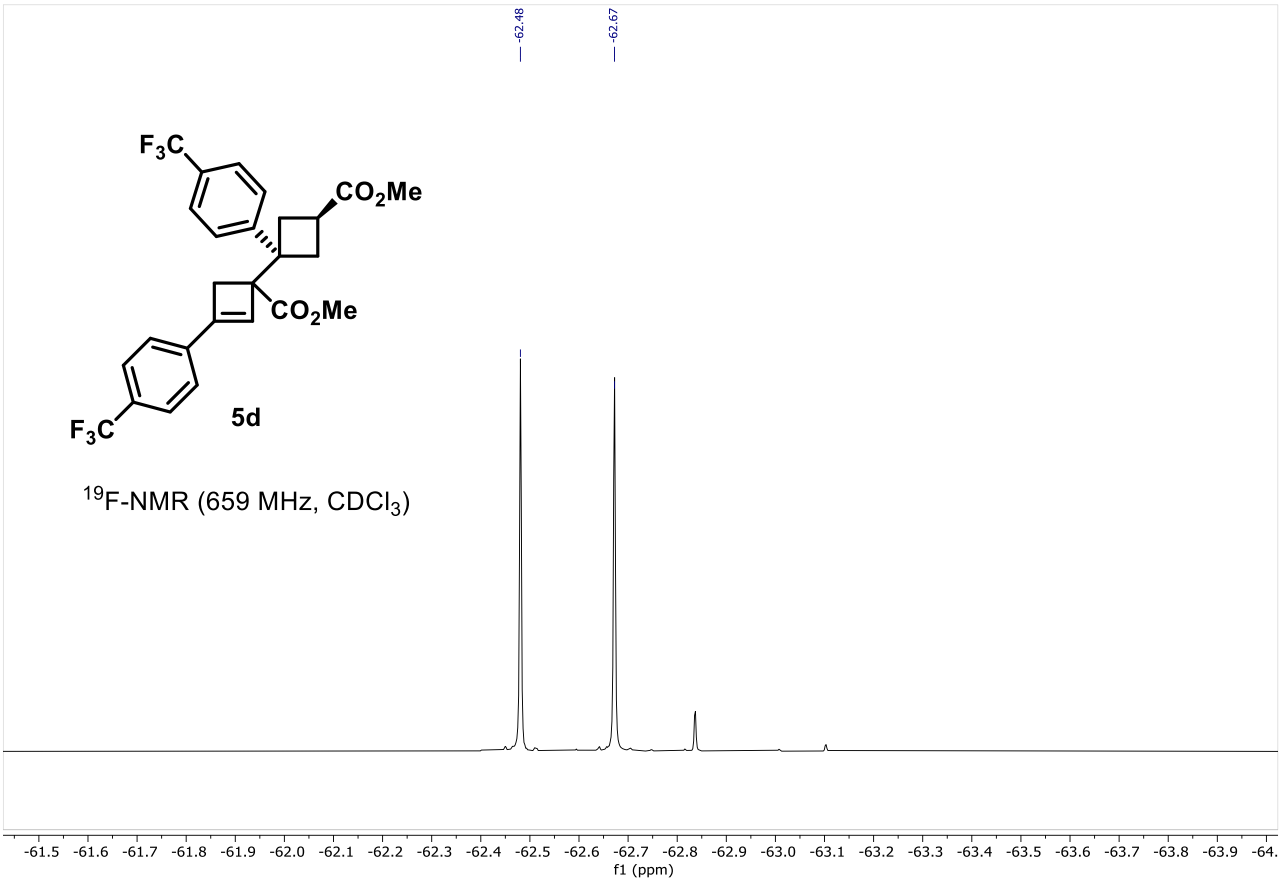
-62.48
-62.67
F_3C
CO_2Me
CO_2Me
F_3C
5d
^{19}F-NMR (659 MHz, $CDCl_3$)
-61.5 -61.6 -61.7 -61.8 -61.9 -62.0 -62.1 -62.2 -62.3 -62.4 -62.5 -62.6 -62.7 -62.8 -62.9 -63.0 -63.1 -63.2 -63.3 -63.4 -63.5 -63.6 -63.7 -63.8 -63.9 -64.
f1 (ppm)

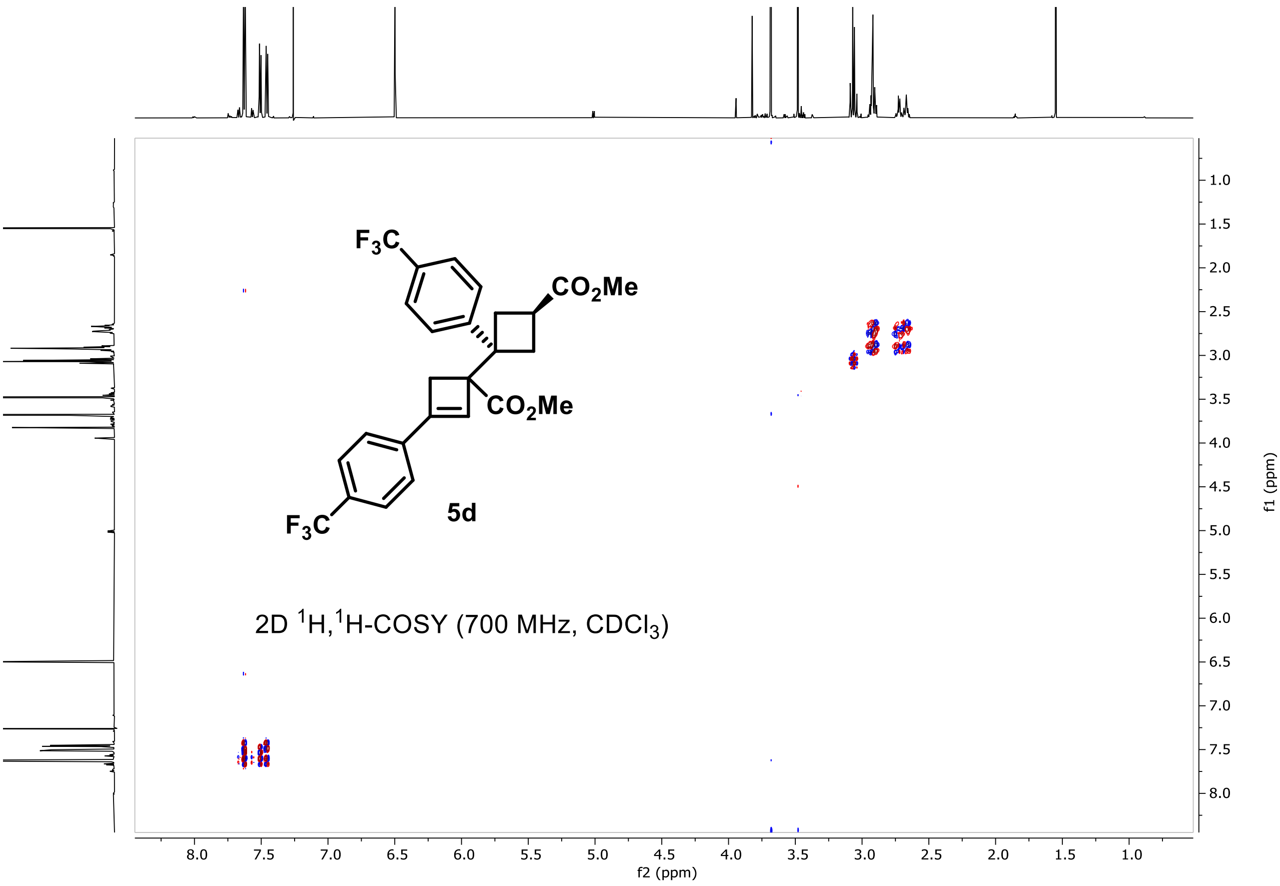
F_3C
CO_2Me
CO_2Me
F_3C
5d
2D ^{1}H,^{1}H-COSY (700 MHz, $CDCl_3$)
f1 (ppm)
1.0 1.5 2.0 2.5 3.0 3.5 4.0 4.5 5.0 5.5 6.0 6.5 7.0 7.5 8.0
8.0 7.5 7.0 6.5 6.0 5.5 5.0 4.5 4.0 3.5 3.0 2.5 2.0 1.5 1.0
f2 (ppm)

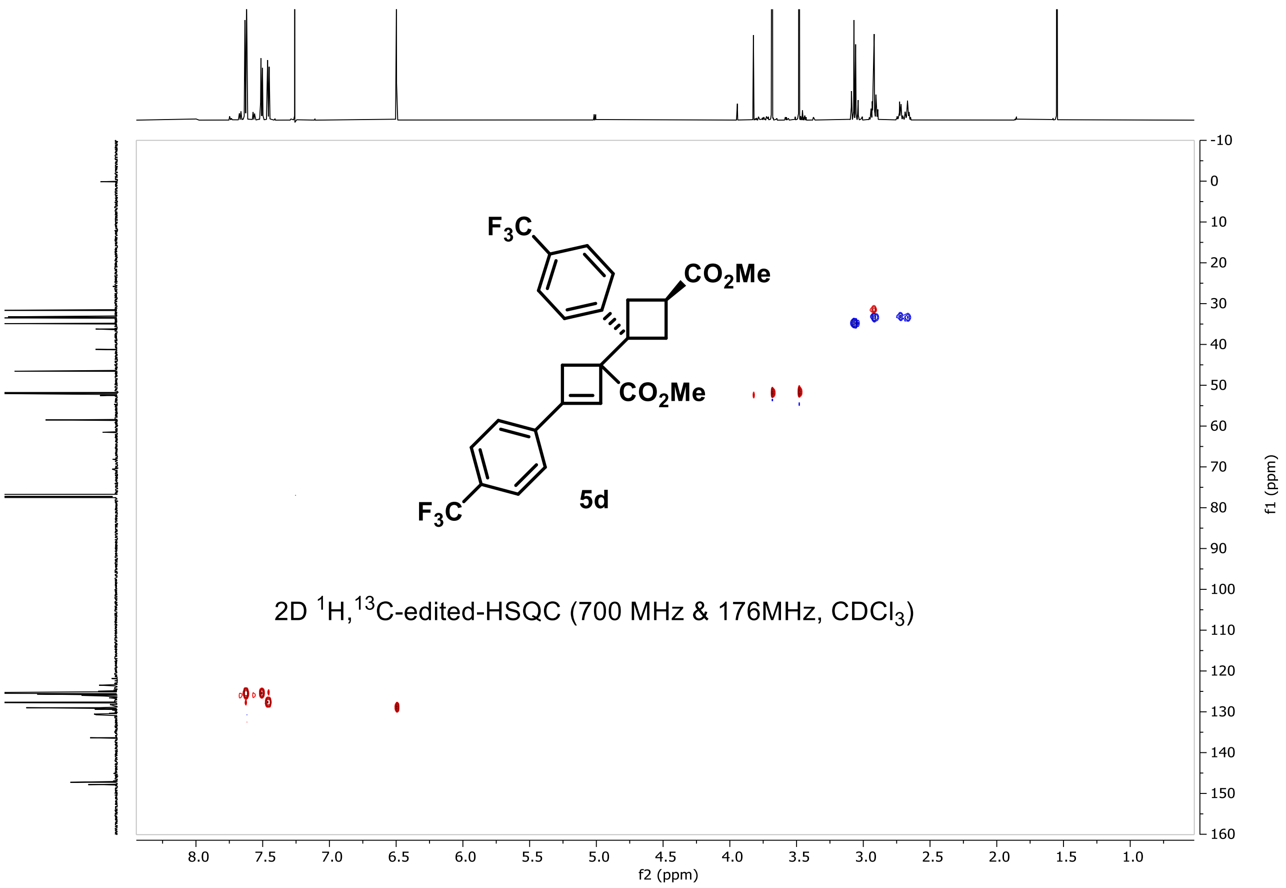
F3C
CO2Me
CO2Me
F3C
5d
2D 1H,13C-edited-HSQC (700 MHz & 176MHz, CDCl3)
f2 (ppm)
f1 (ppm)

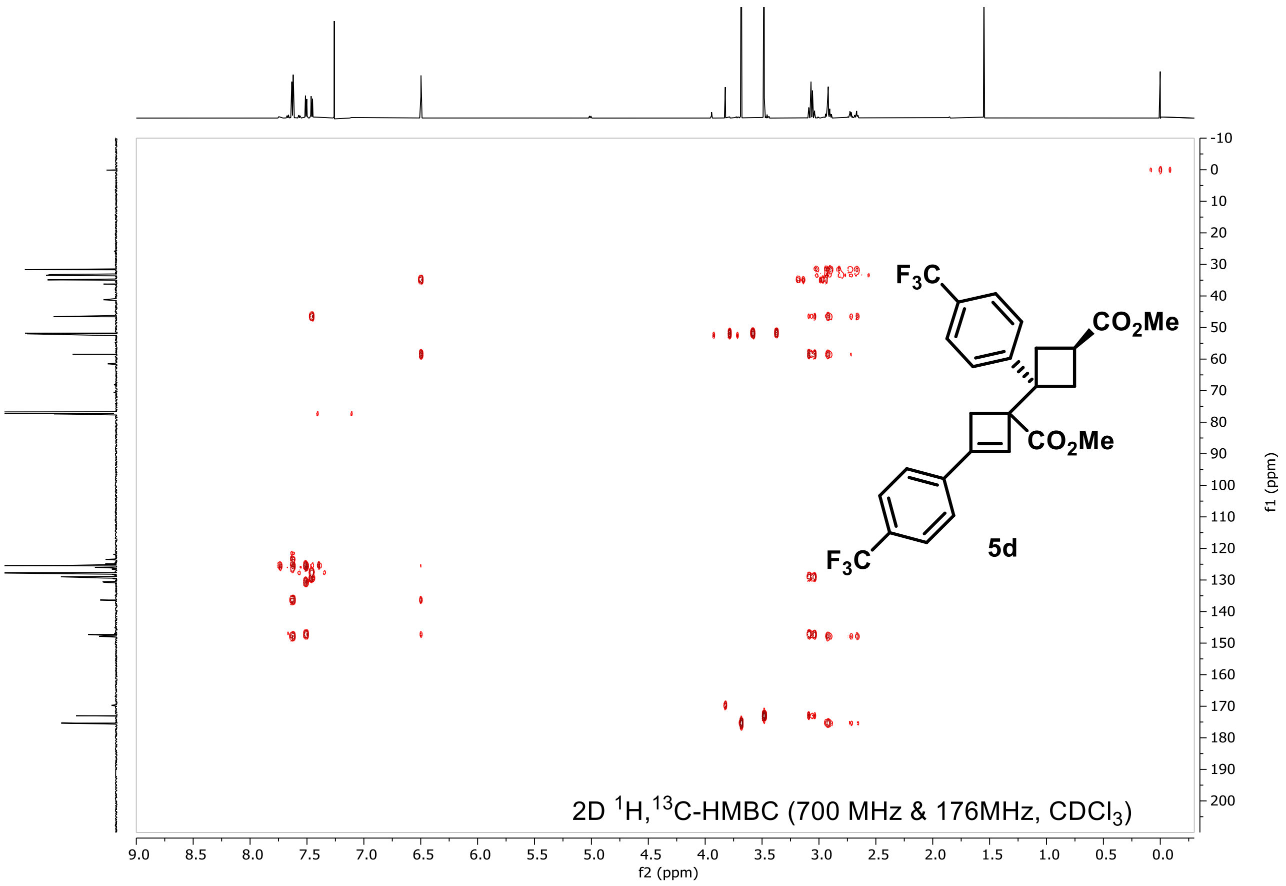
F3C
CO2Me
CO2Me
F3C
5d
2D 1H,13C-HMBC (700 MHz & 176MHz, CDCl3)
f2 (ppm)
f1 (ppm)

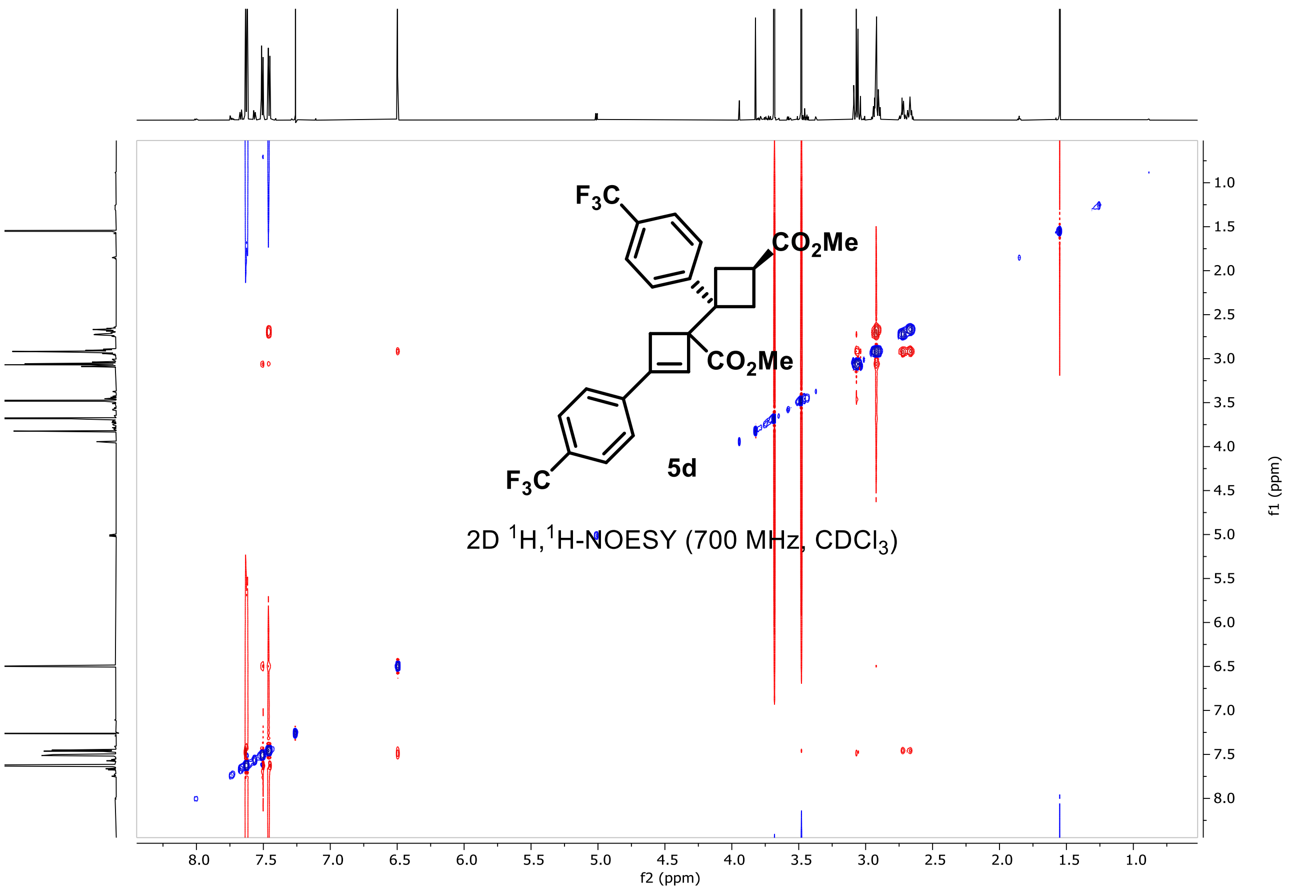
F3C
CO2Me
CO2Me
F3C
5d
2D 1H,1H-NOESY (700 MHz, CDCl3)
f1 (ppm)
f2 (ppm)

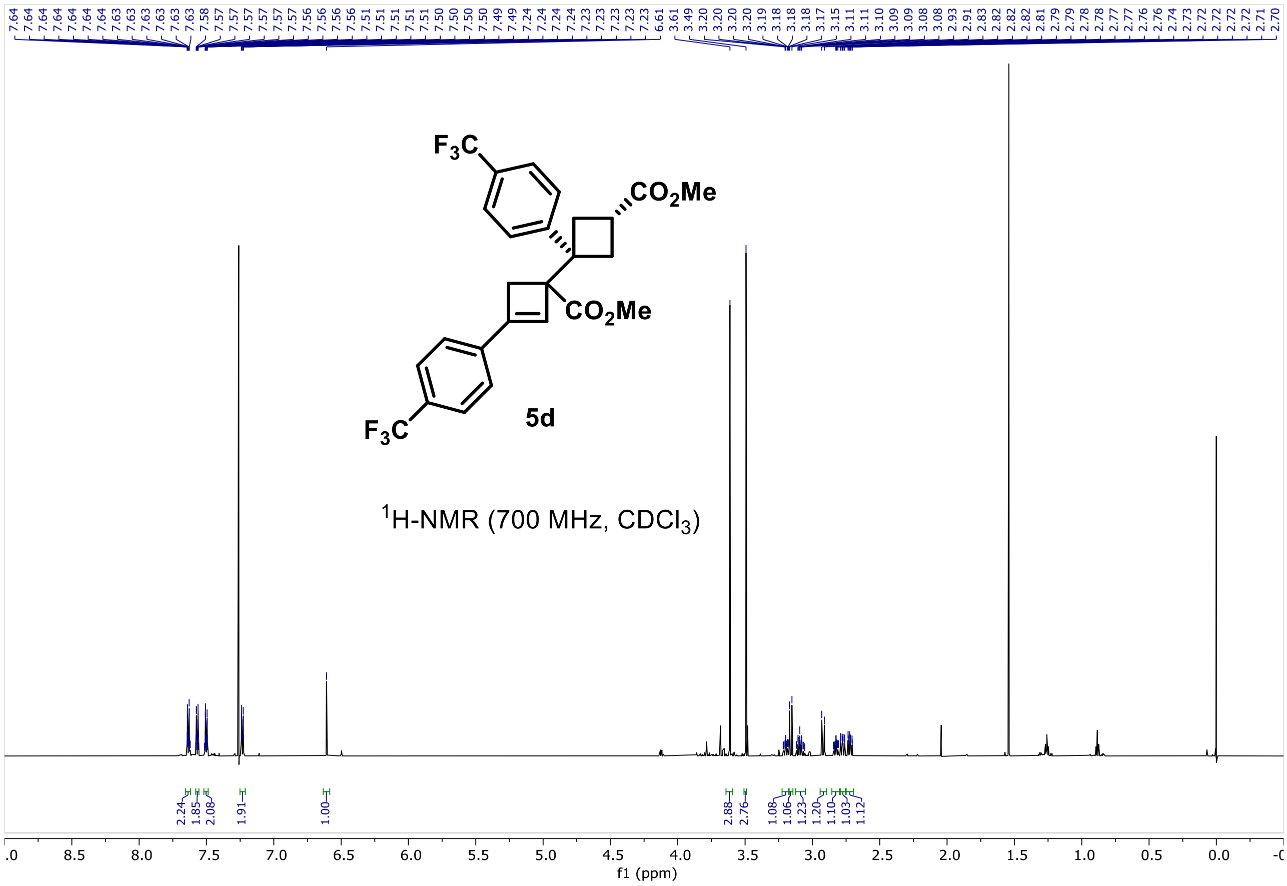

^{1}H-NMR (700 MHz, $CDCl_3$)

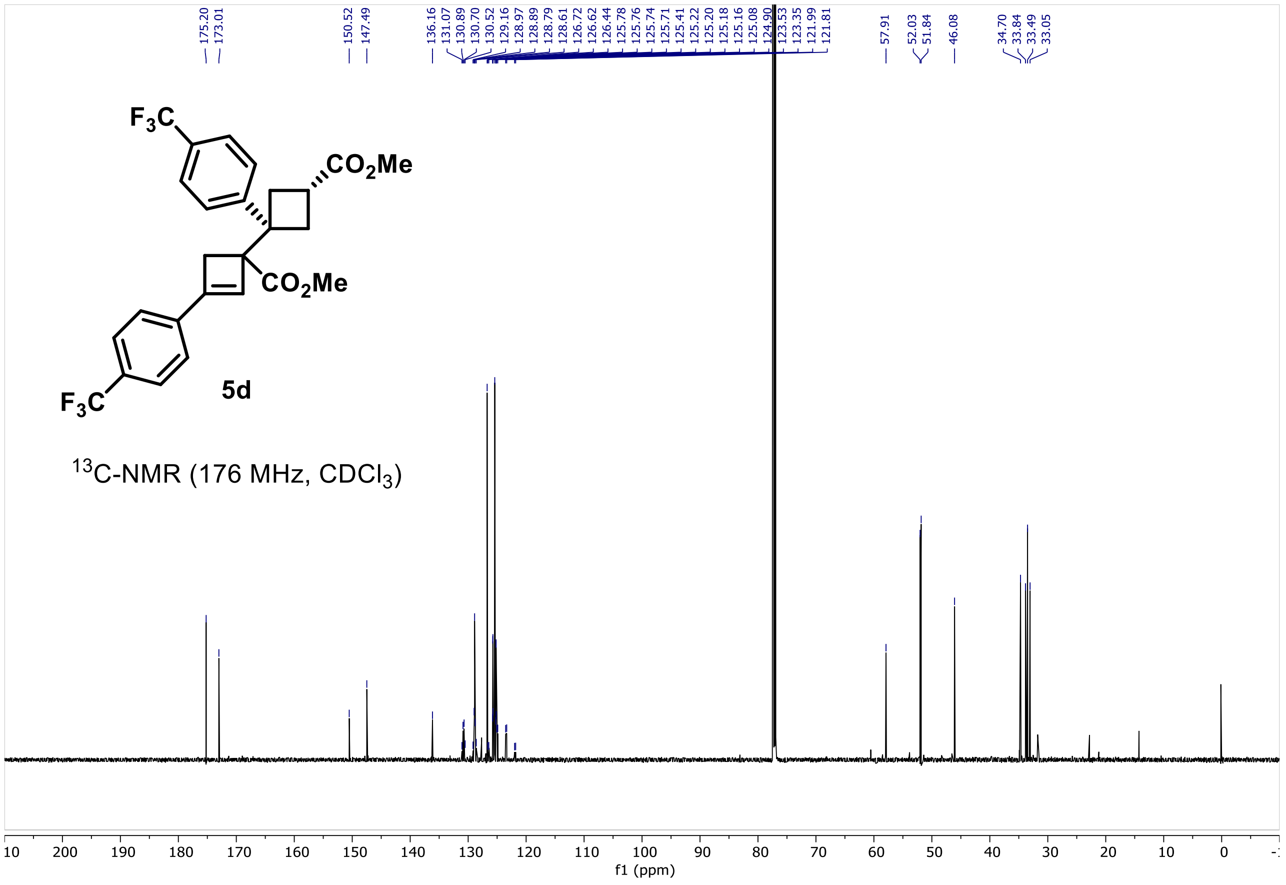

^{13}C-NMR (176 MHz, $CDCl_3$)

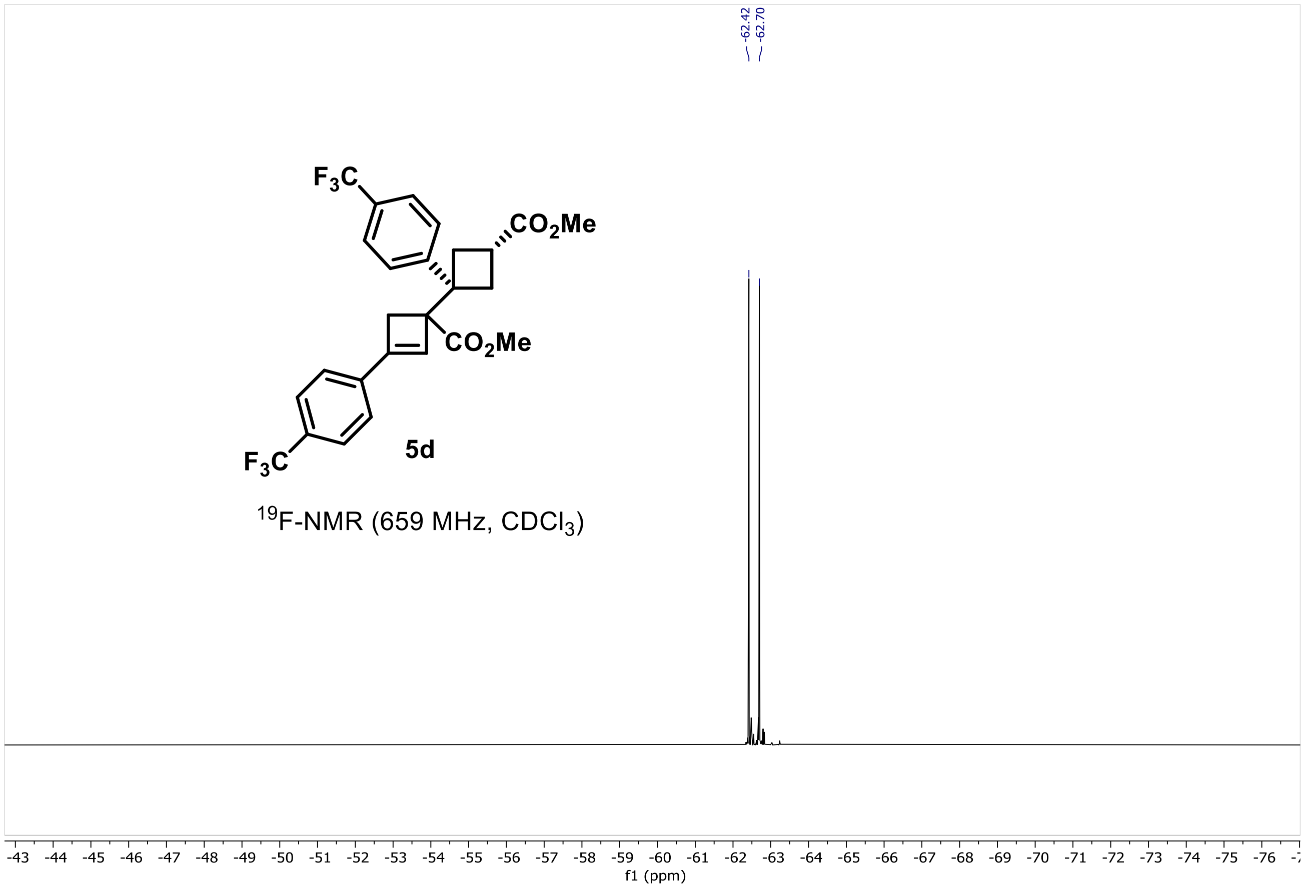
-62.42
-62.70
F3C
CO2Me
CO2Me
F3C
5d
19F-NMR (659 MHz, CDCl3)
f1 (ppm)

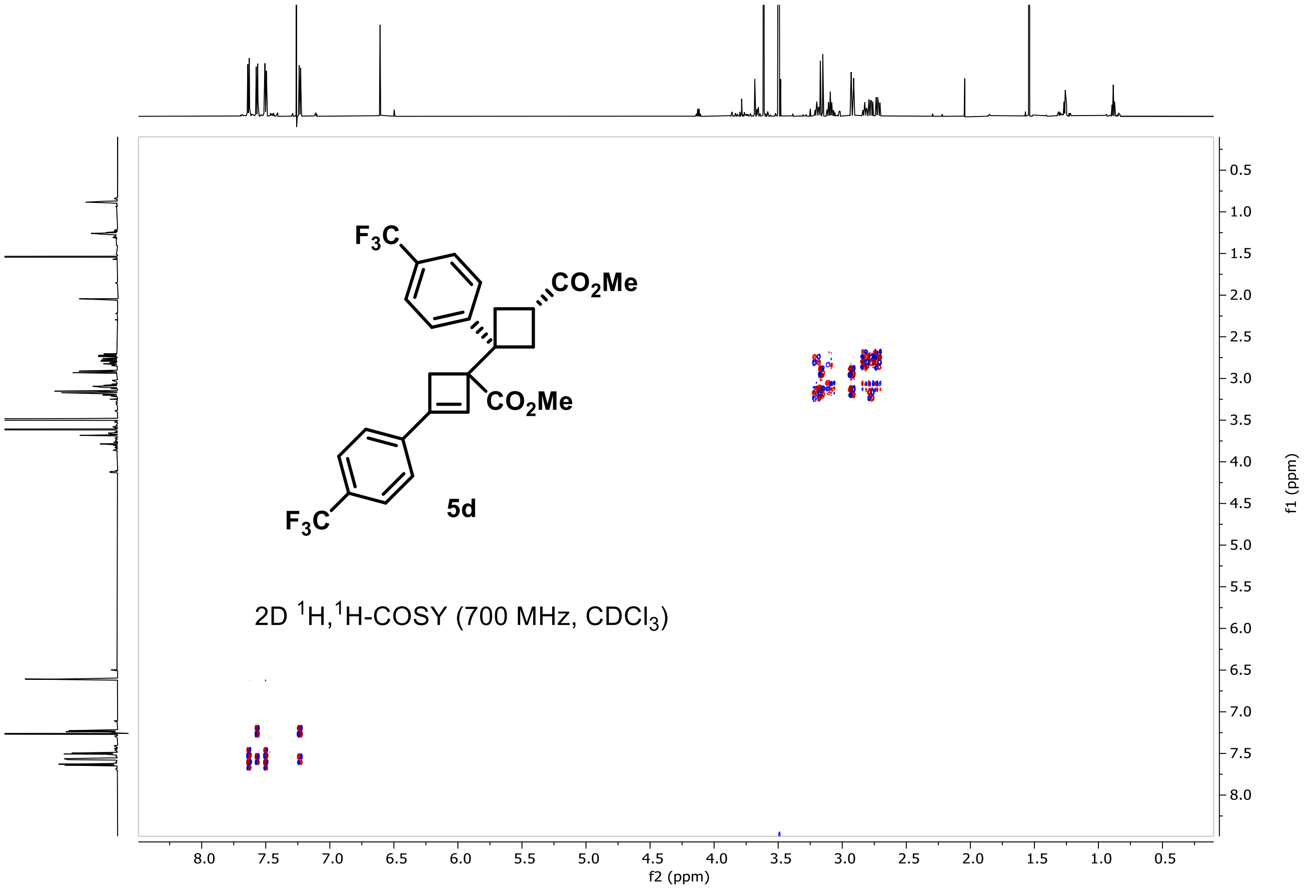
F3C
CO2Me
CO2Me
F3C
5d
2D 1H,1H-COSY (700 MHz, CDCl3)
f2 (ppm)
f1 (ppm)

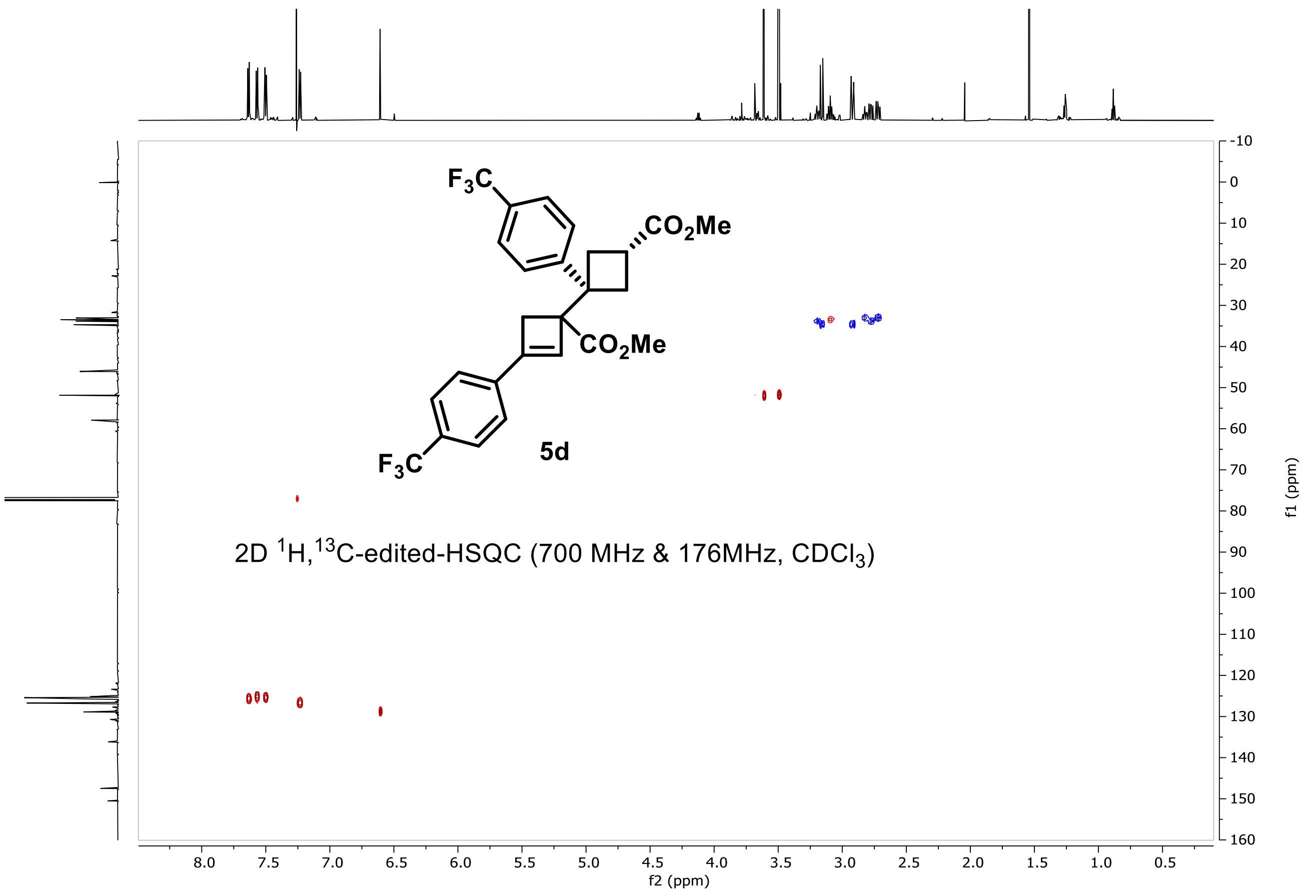
5d
2D ^{1}H,^{13}C-edited-HSQC (700 MHz & 176MHz, CDCl$_3$)
f2 (ppm)
f1 (ppm)

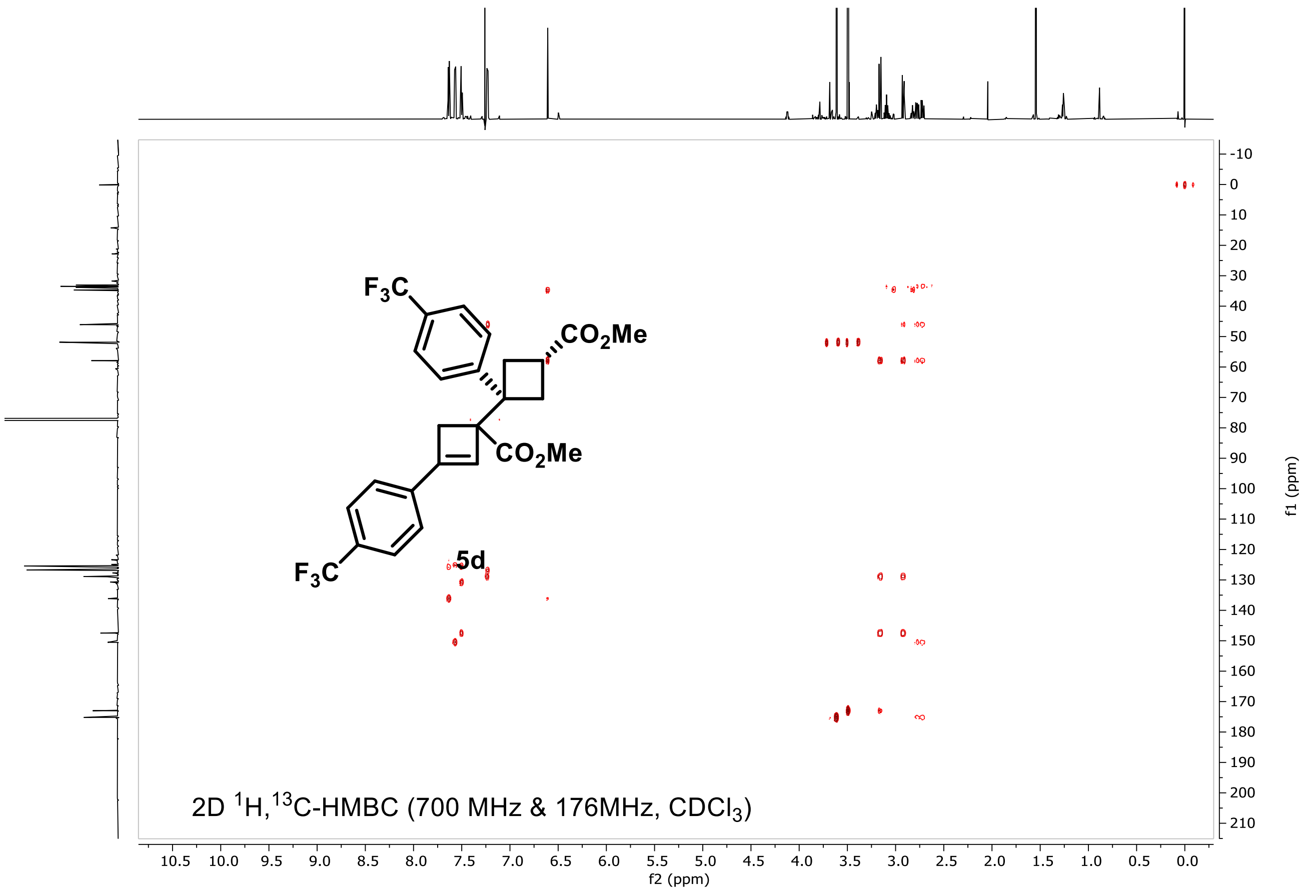
5d
2D ^{1}H,^{13}C-HMBC (700 MHz & 176MHz, CDCl$_3$)
f2 (ppm)
f1 (ppm)

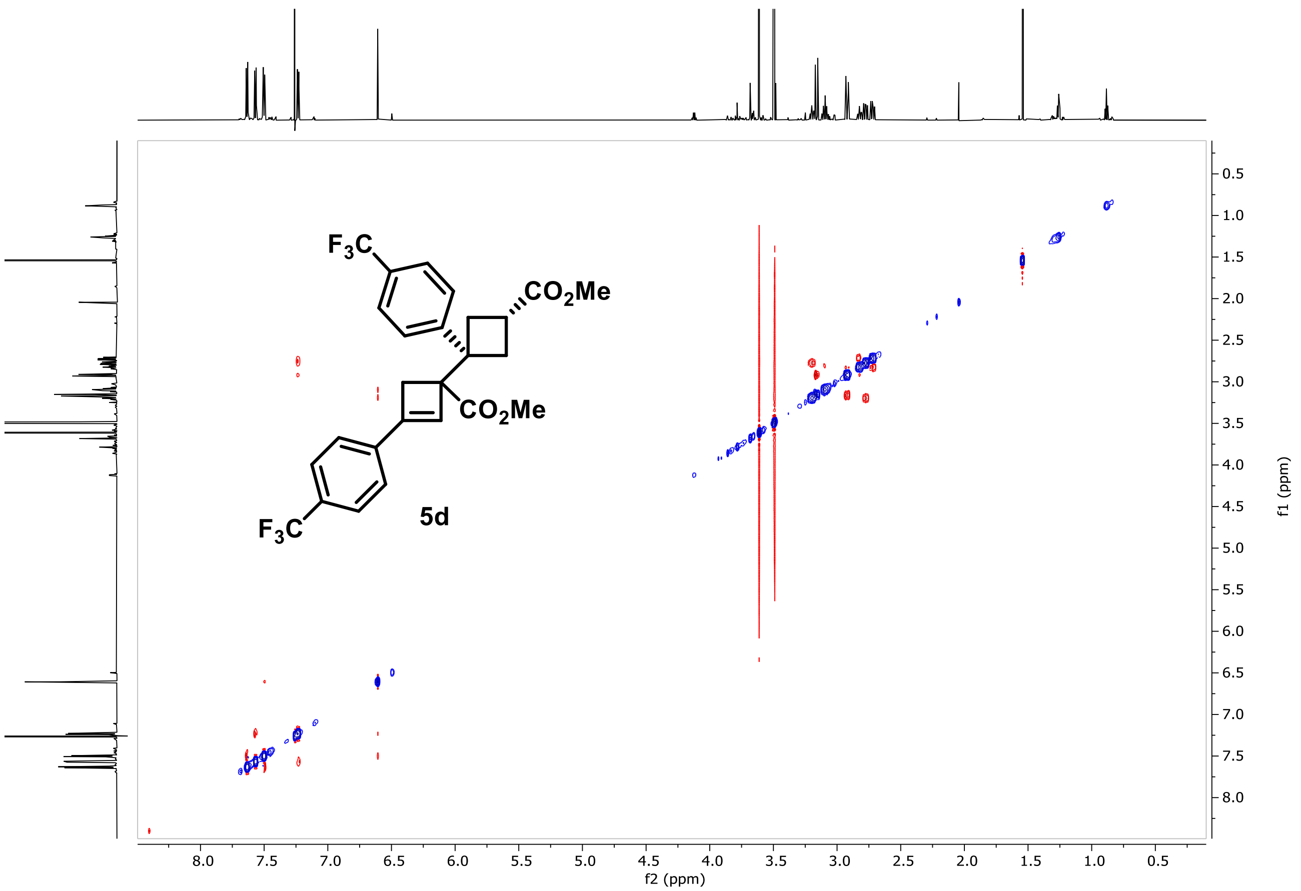

F3C
CO2Me
CO2Me
F3C
5d
f2 (ppm)
f1 (ppm)

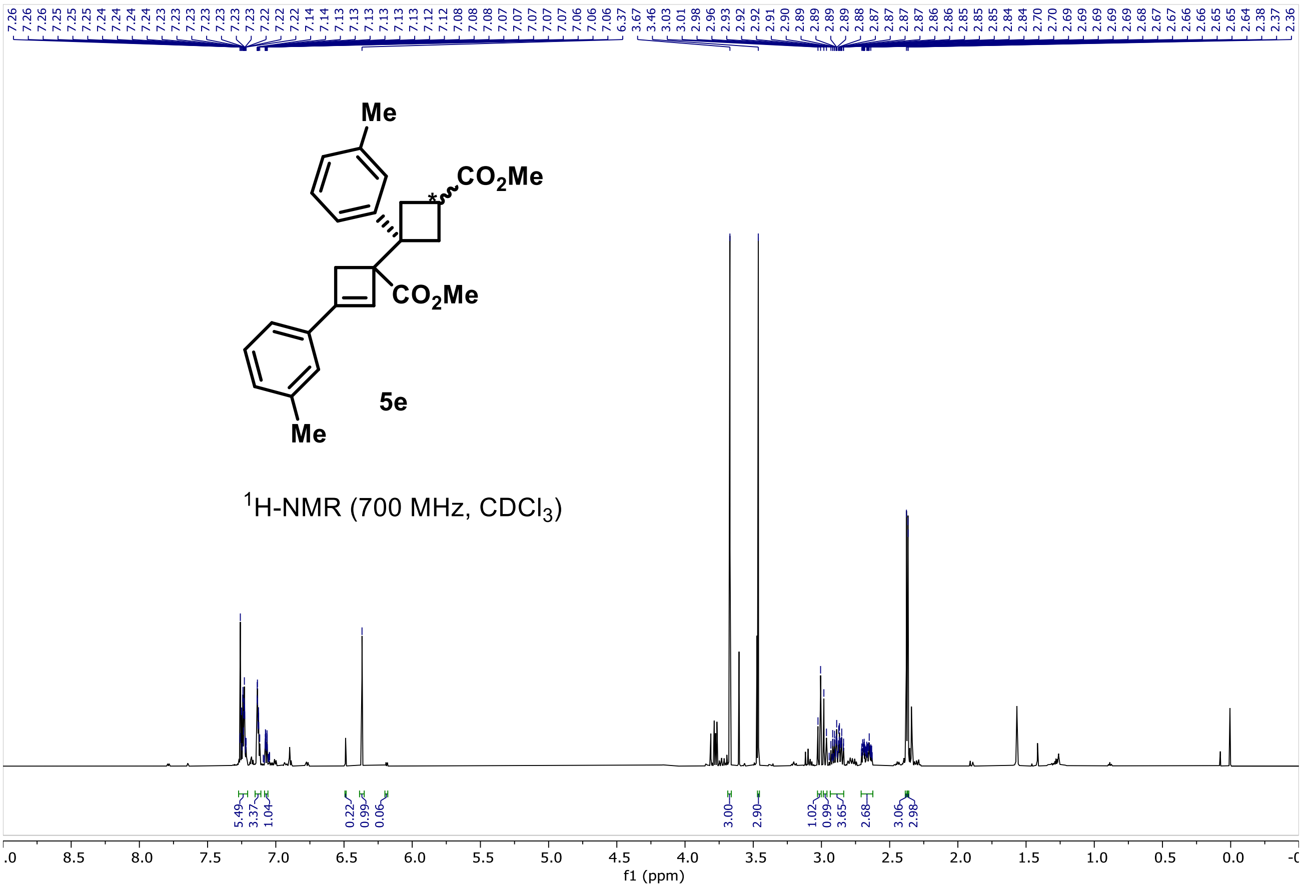
7.26 7.26 7.25 7.25 7.25 7.24 7.24 7.24 7.23 7.23 7.23 7.23 7.23 7.23 7.22 7.22 7.14 7.14 7.13 7.13 7.13 7.13 7.13 7.12 7.12 7.08 7.08 7.08 7.07 7.07 7.07 7.07 7.07 7.06 7.06 7.06 6.37 3.67 3.46 3.03 3.01 2.98 2.96 2.93 2.92 2.92 2.91 2.90 2.89 2.89 2.89 2.89 2.88 2.87 2.87 2.87 2.86 2.86 2.85 2.85 2.84 2.84 2.70 2.70 2.69 2.69 2.69 2.69 2.69 2.68 2.67 2.67 2.66 2.66 2.65 2.65 2.64 2.38 2.37 2.36
Me
CO2Me
CO2Me
Me
5e
1H-NMR (700 MHz, CDCl3)
5.49 3.37 1.04 0.22 0.99 0.06 3.00 2.90 1.02 0.99 3.65 2.68 3.06 2.98
f1 (ppm)

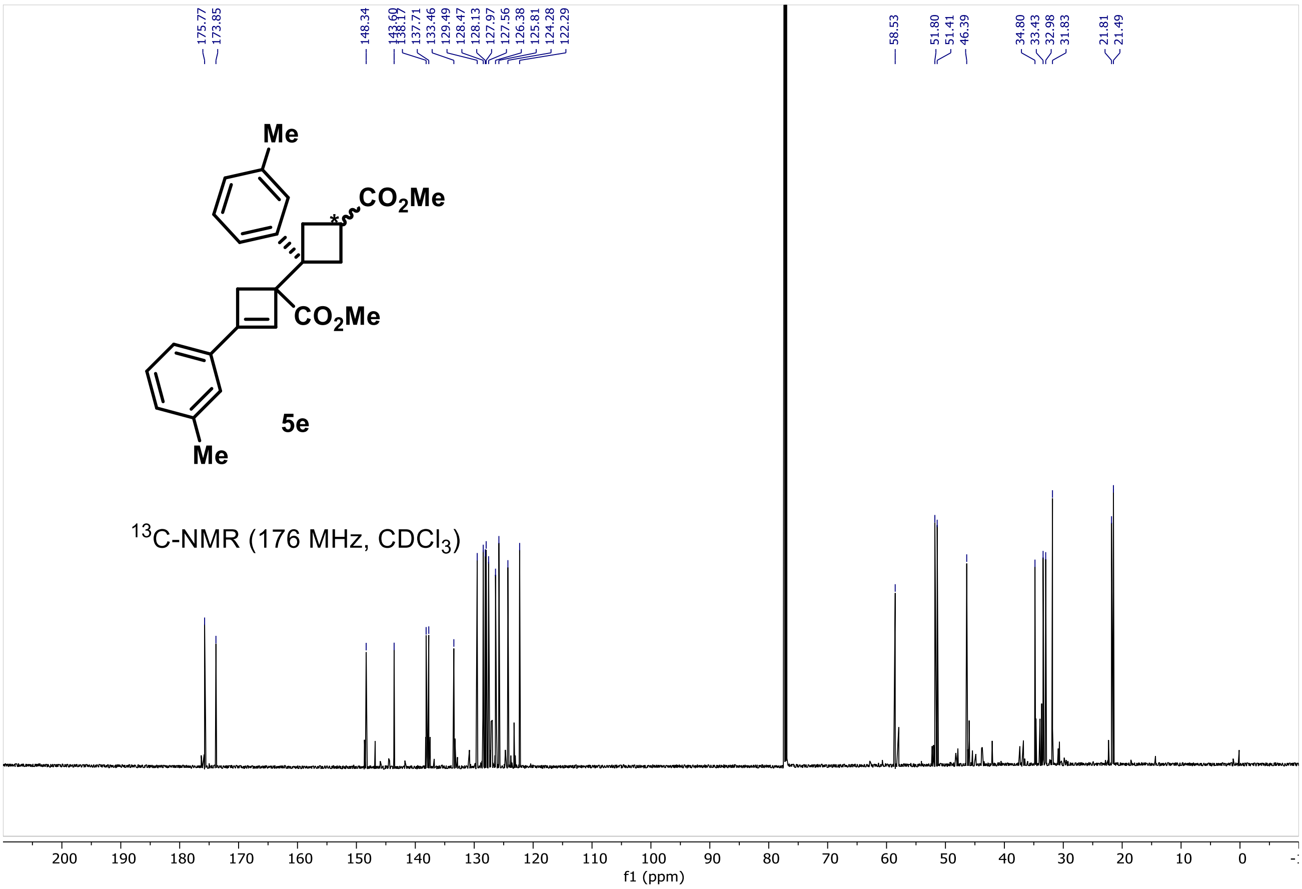
175.77 173.85 148.34 143.60 138.17 137.71 133.46 129.49 128.47 128.13 127.97 127.56 126.38 125.81 124.28 122.29 58.53 51.80 51.41 46.39 34.80 33.43 32.98 31.83 21.81 21.49
Me
CO2Me
CO2Me
Me
5e
13C-NMR (176 MHz, CDCl3)
f1 (ppm)

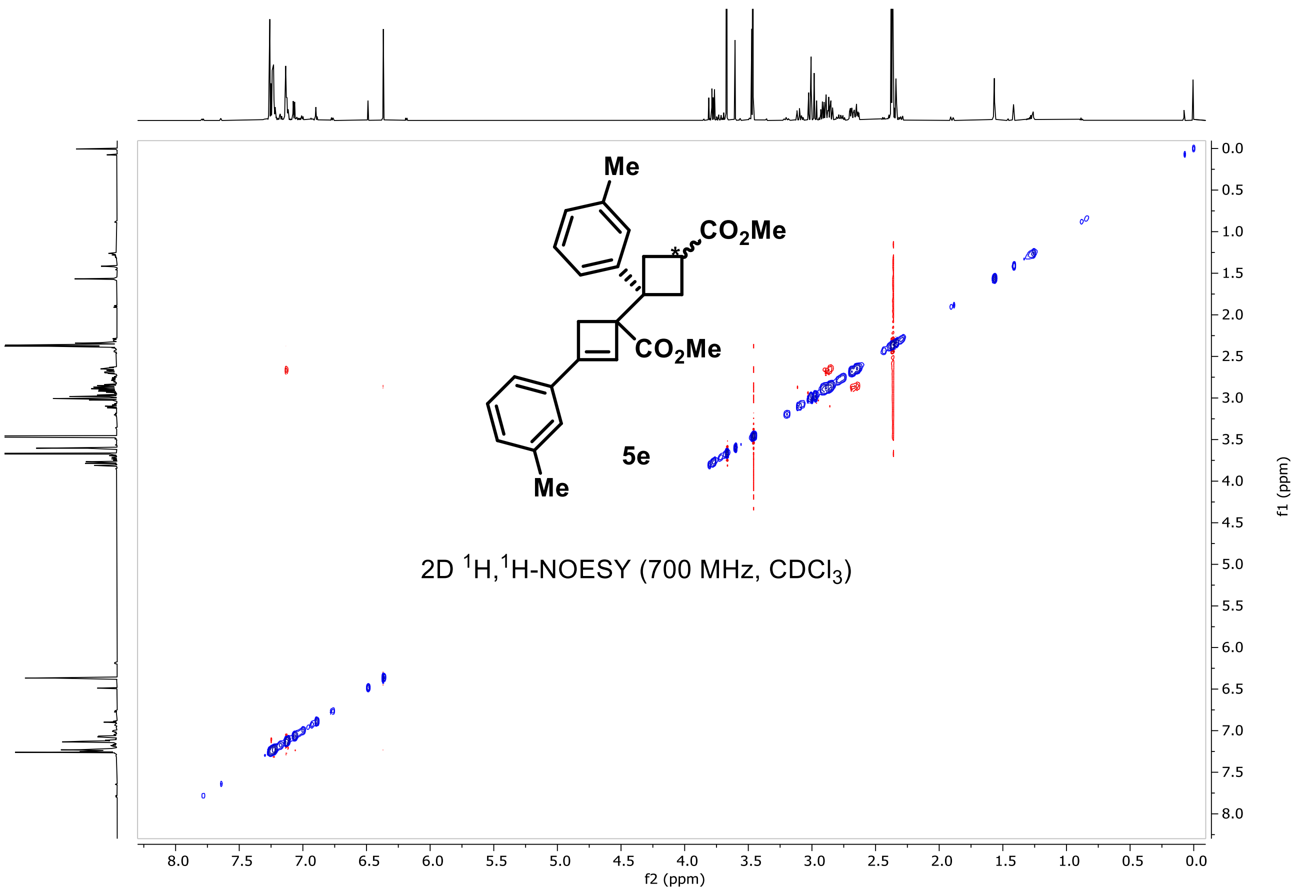

Me
CO2Me
*
CO2Me
Me
5e
2D 1H,1H-NOESY (700 MHz, CDCl3)
f1 (ppm)
f2 (ppm)

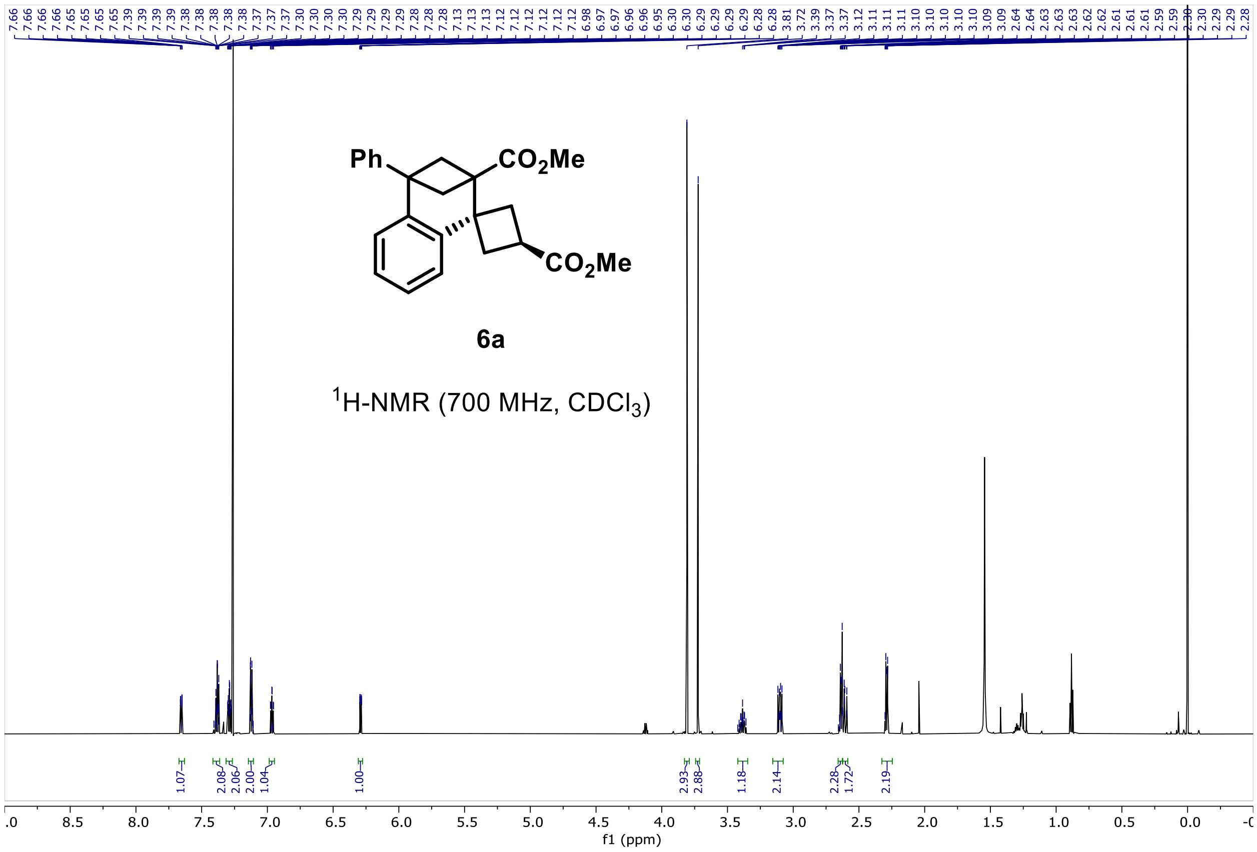

6a

^{1}H-NMR (700 MHz, $CDCl_3$)

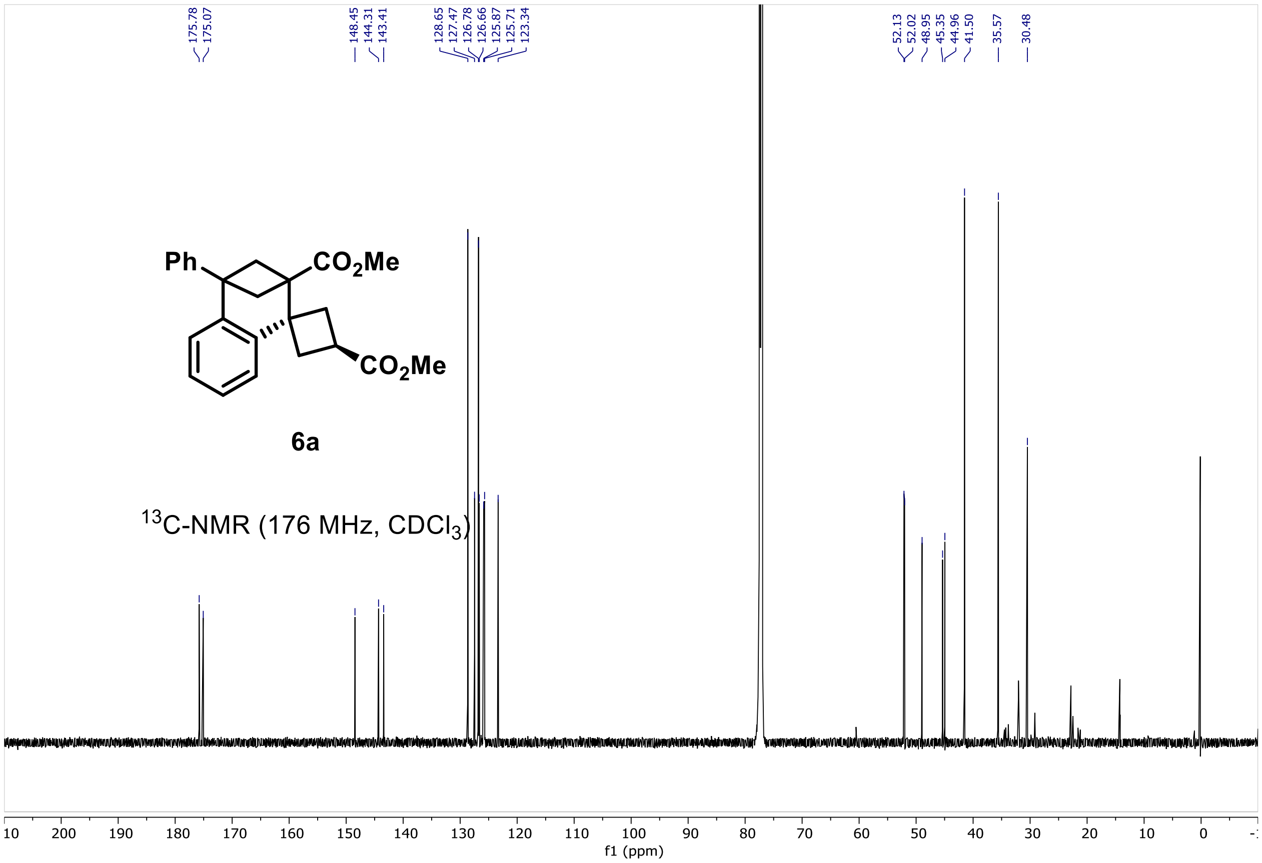

6a

^{13}C-NMR (176 MHz, $CDCl_3$)

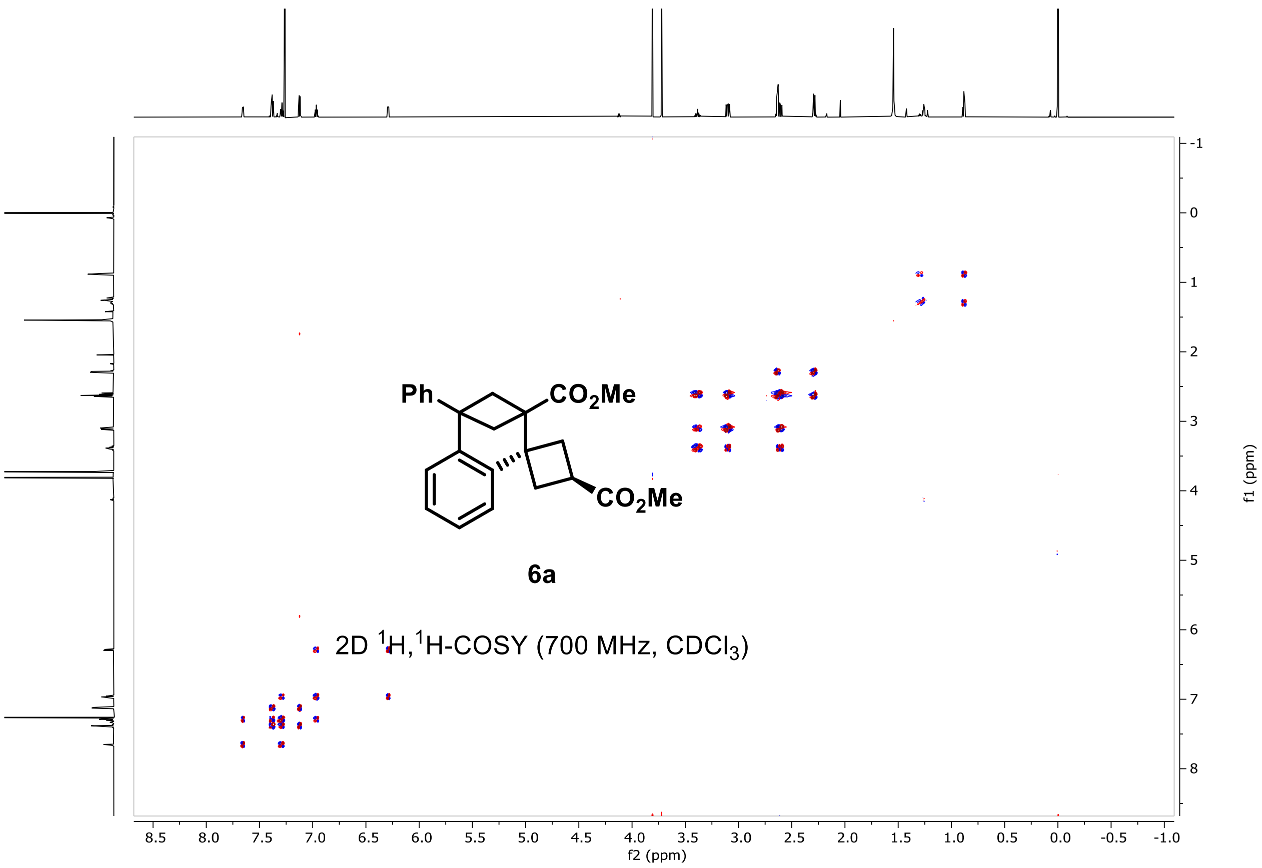
Ph
CO2Me
CO2Me
6a
2D 1H,1H-COSY (700 MHz, CDCl3)
f2 (ppm)
f1 (ppm)

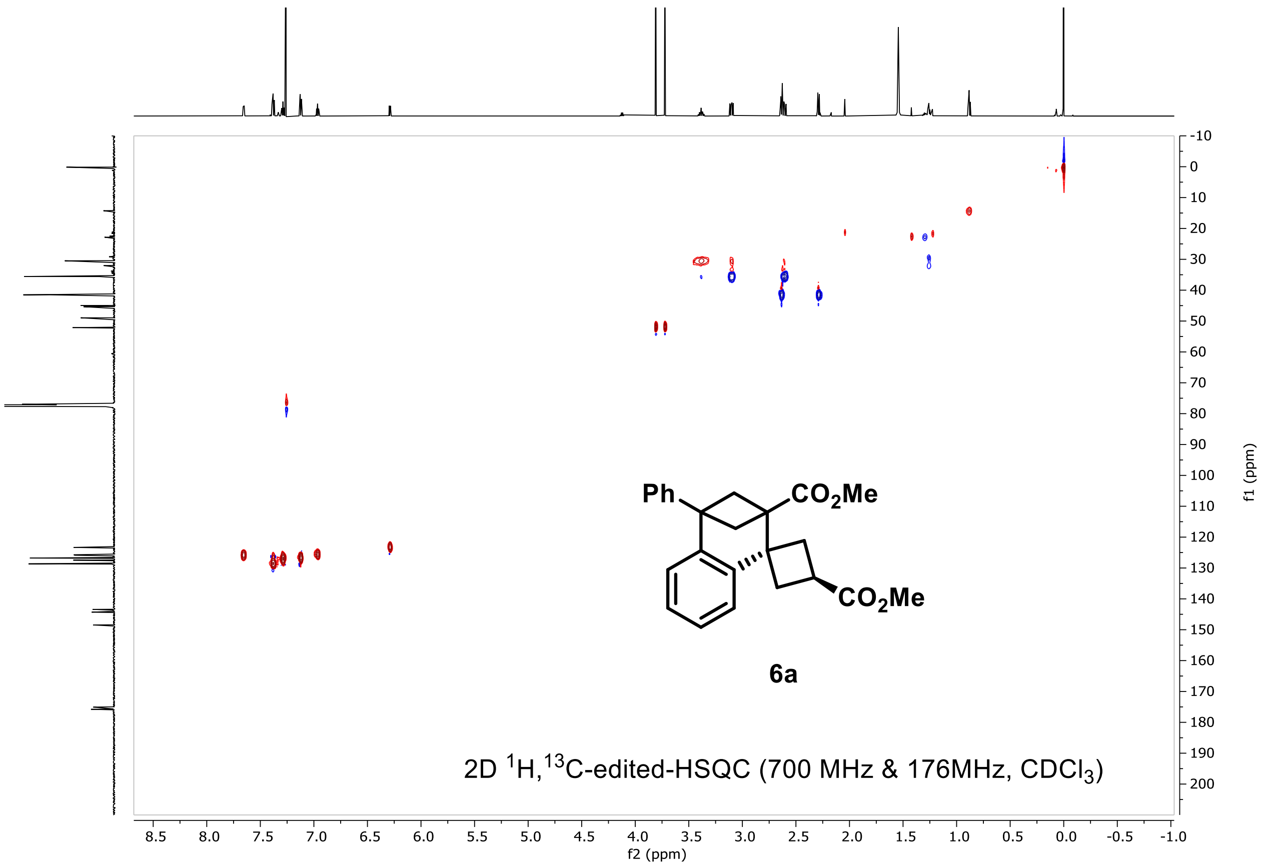
Ph
CO2Me
CO2Me
6a
2D 1H,13C-edited-HSQC (700 MHz & 176MHz, CDCl3)
f2 (ppm)
f1 (ppm)

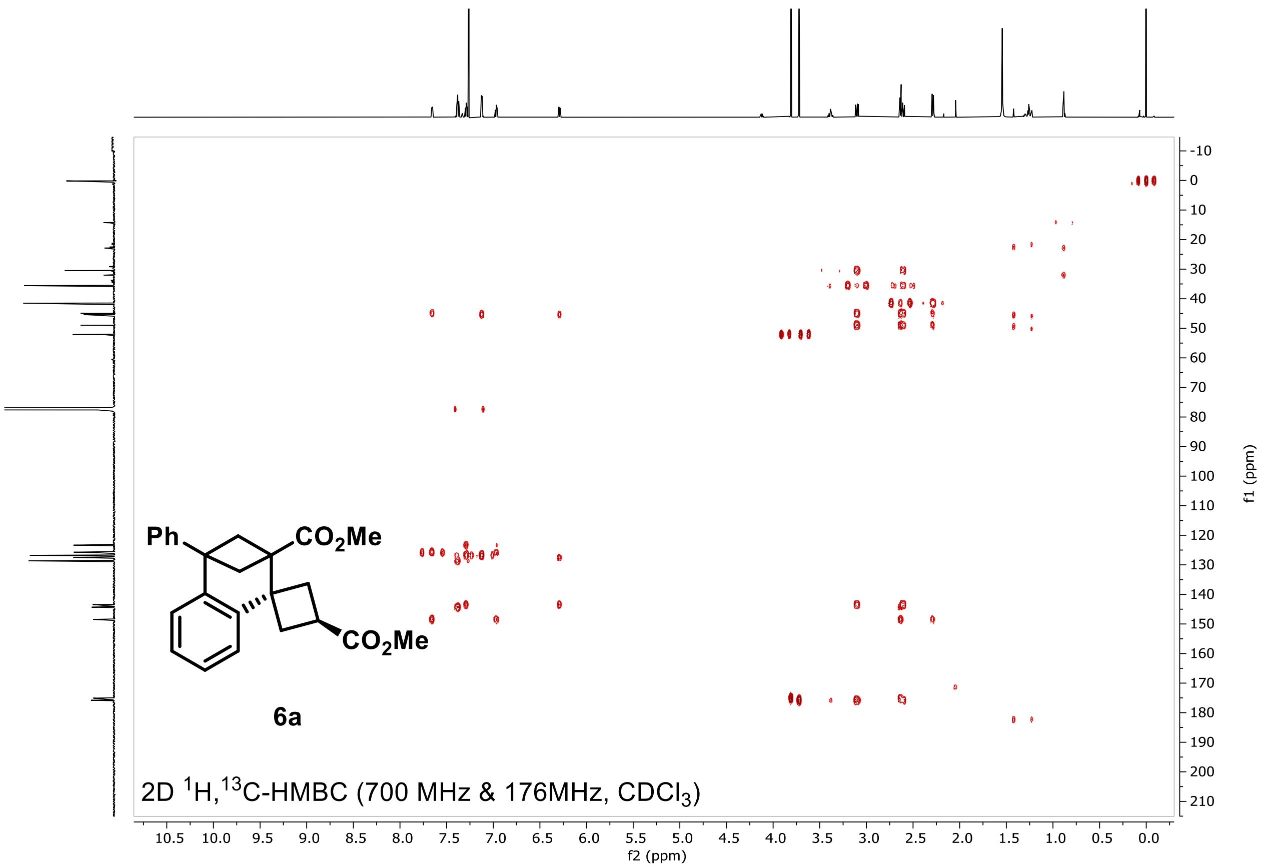
Ph
CO2Me
CO2Me
6a
2D 1H,13C-HMBC (700 MHz & 176MHz, CDCl3)
f2 (ppm)
f1 (ppm)

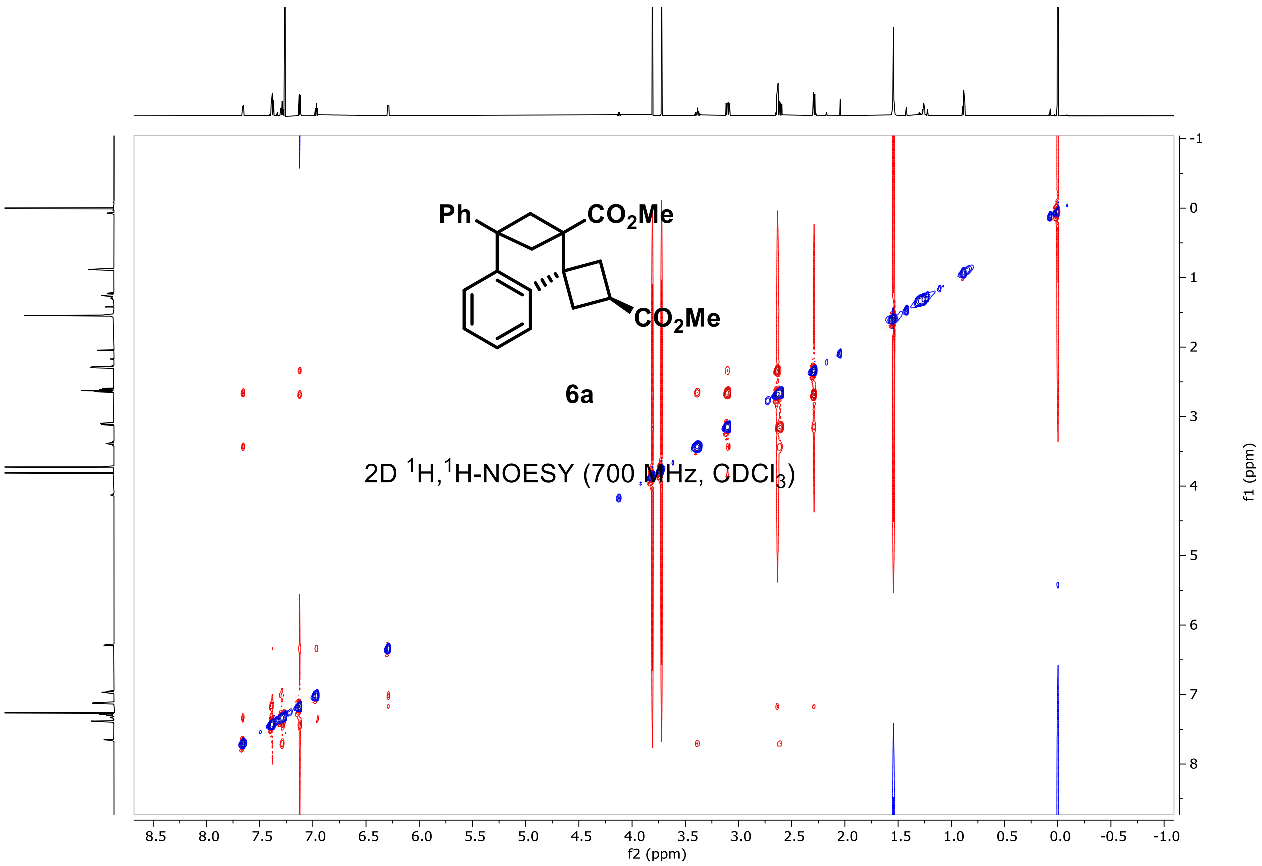
Ph
CO2Me
CO2Me
6a
2D 1H,1H-NOESY (700 MHz, CDCl3)
f2 (ppm)
f1 (ppm)

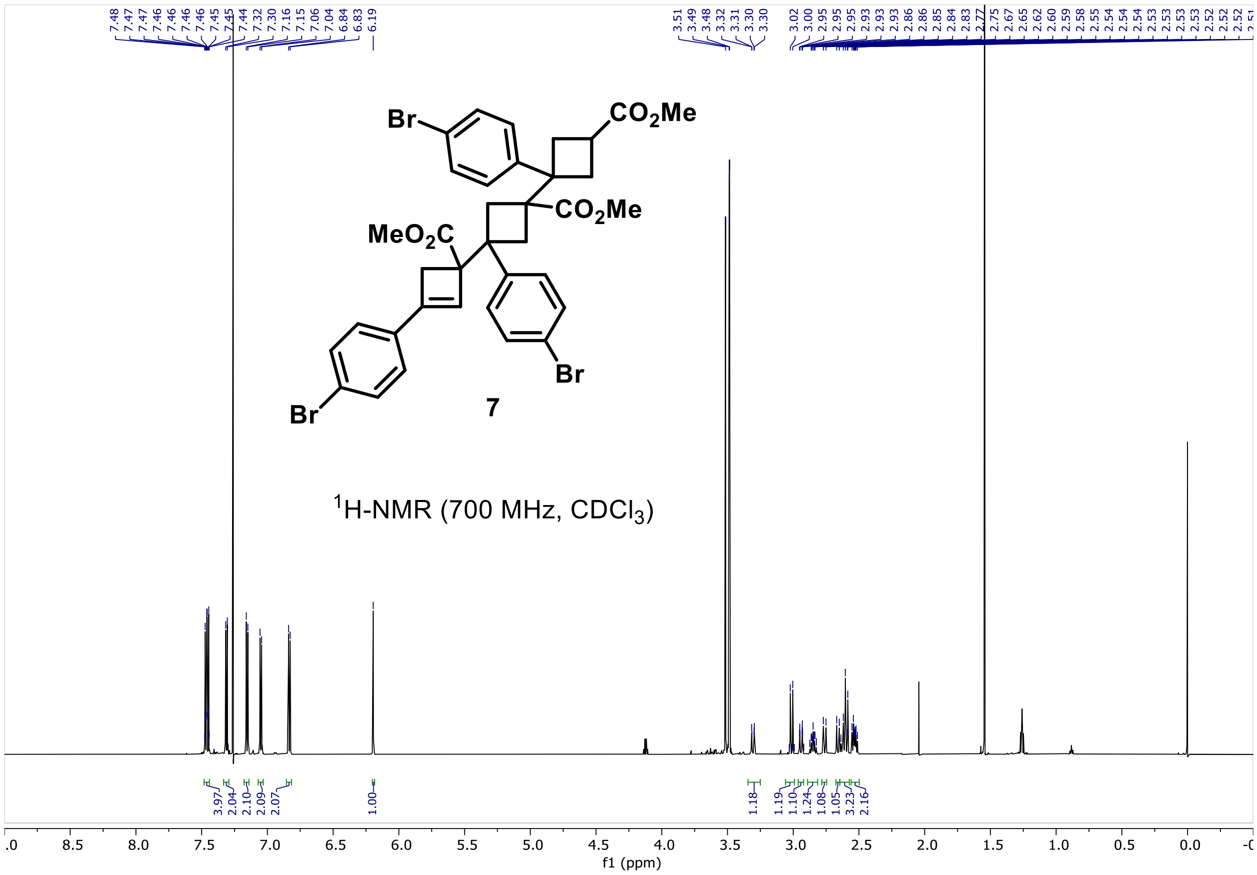
7.48 7.47 7.47 7.46 7.46 7.46 7.45 7.45 7.44 7.32 7.30 7.16 7.15 7.06 7.04 6.84 6.83 6.19
3.51 3.49 3.48 3.32 3.31 3.30 3.30
3.02 3.00 2.95 2.95 2.95 2.93 2.93 2.93 2.86 2.86 2.85 2.84 2.83 2.77 2.75 2.67 2.65 2.62 2.60 2.59 2.58 2.55 2.54 2.54 2.54 2.53 2.53 2.53 2.53 2.52 2.52
Br
CO_2Me
CO_2Me
MeO_2C
Br
Br
7
^{1}H-NMR (700 MHz, $CDCl_3$)
3.97 2.04 2.10 2.09 2.07 1.00 1.18 1.19 1.10 1.24 1.08 1.05 3.23 2.16
8.5 8.0 7.5 7.0 6.5 6.0 5.5 5.0 4.5 4.0 3.5 3.0 2.5 2.0 1.5 1.0 0.5 0.0
f1 (ppm)

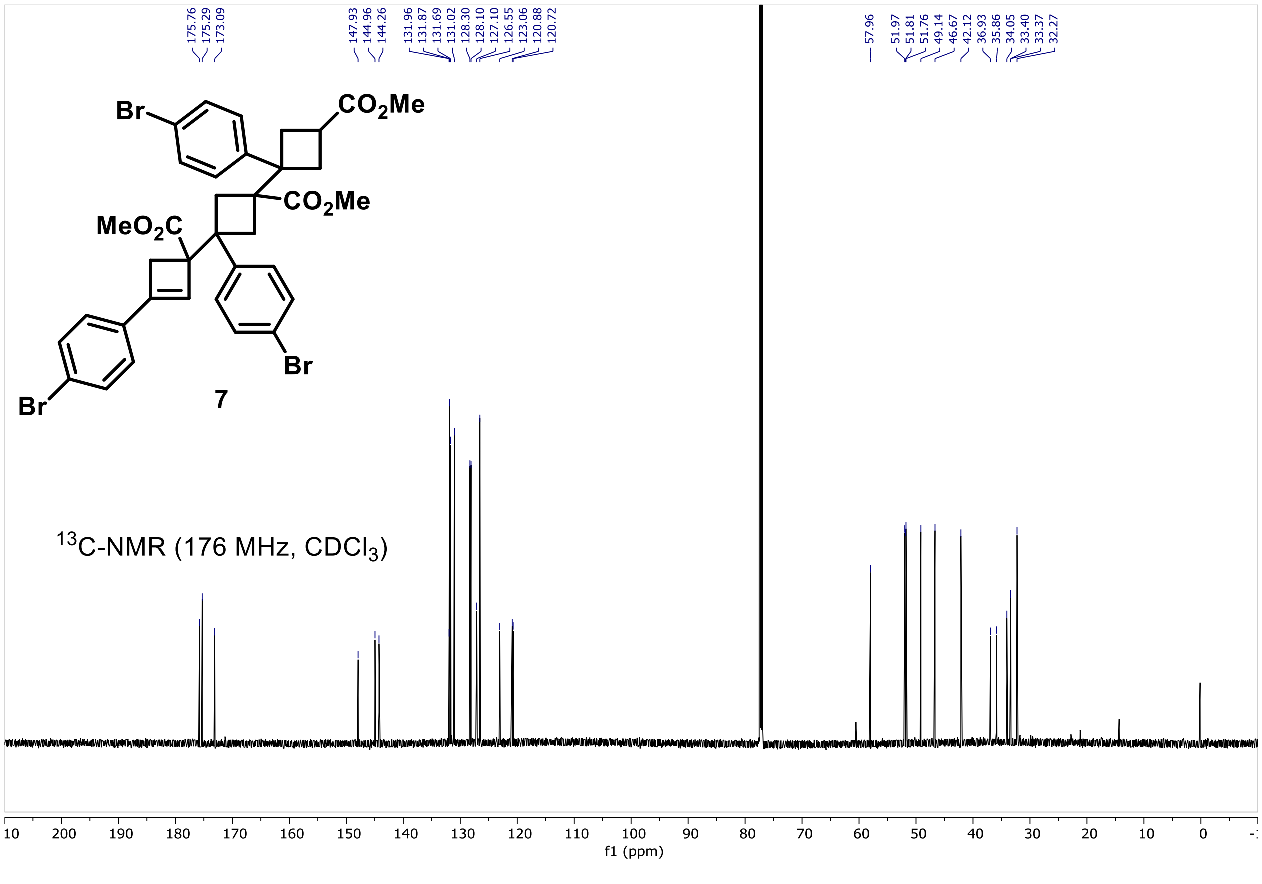
175.76 175.29 173.09
147.93 144.96 144.26
131.96 131.87 131.69 131.02 128.30 128.10 127.10 126.55 123.06 120.88 120.72
57.96
51.97 51.81 51.76 49.14 46.67 42.12 36.93 35.86 34.05 33.40 33.37 32.27
Br
CO_2Me
CO_2Me
MeO_2C
Br
Br
7
^{13}C-NMR (176 MHz, $CDCl_3$)
200 190 180 170 160 150 140 130 120 110 100 90 80 70 60 50 40 30 20 10 0
f1 (ppm)

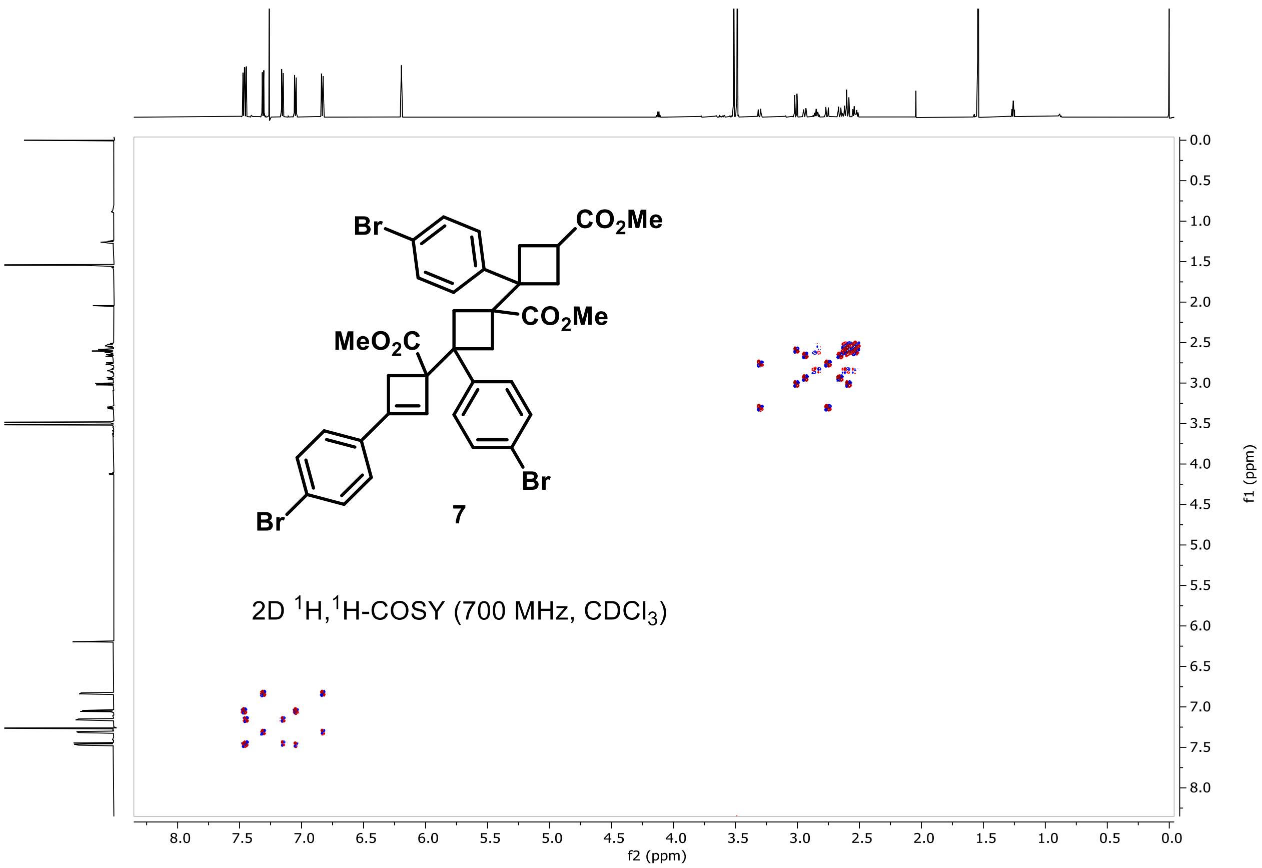
Br
CO2Me
CO2Me
MeO2C
Br
Br
7
2D 1H,1H-COSY (700 MHz, CDCl3)
f2 (ppm)
f1 (ppm)

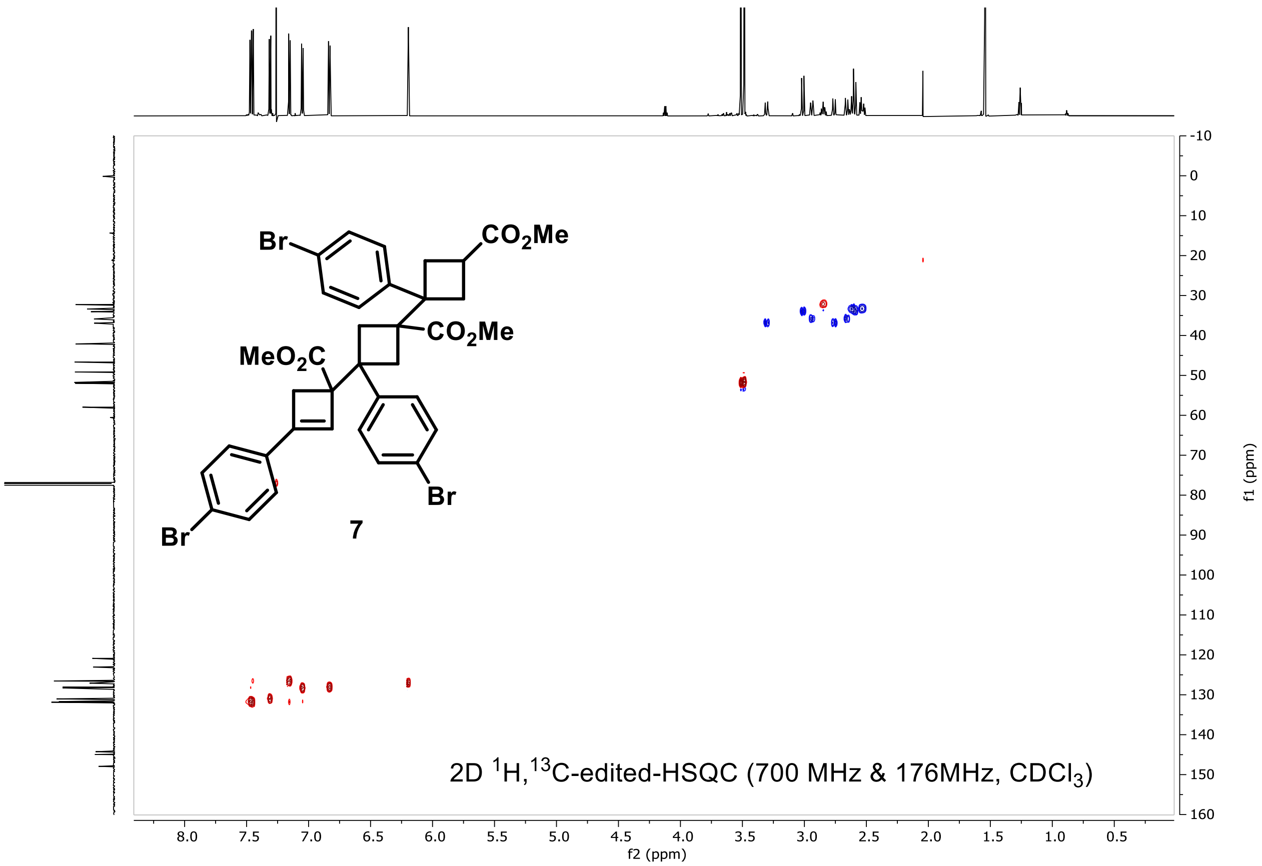
Br
CO2Me
CO2Me
MeO2C
Br
Br
7
2D 1H,13C-edited-HSQC (700 MHz & 176MHz, CDCl3)
f2 (ppm)
f1 (ppm)

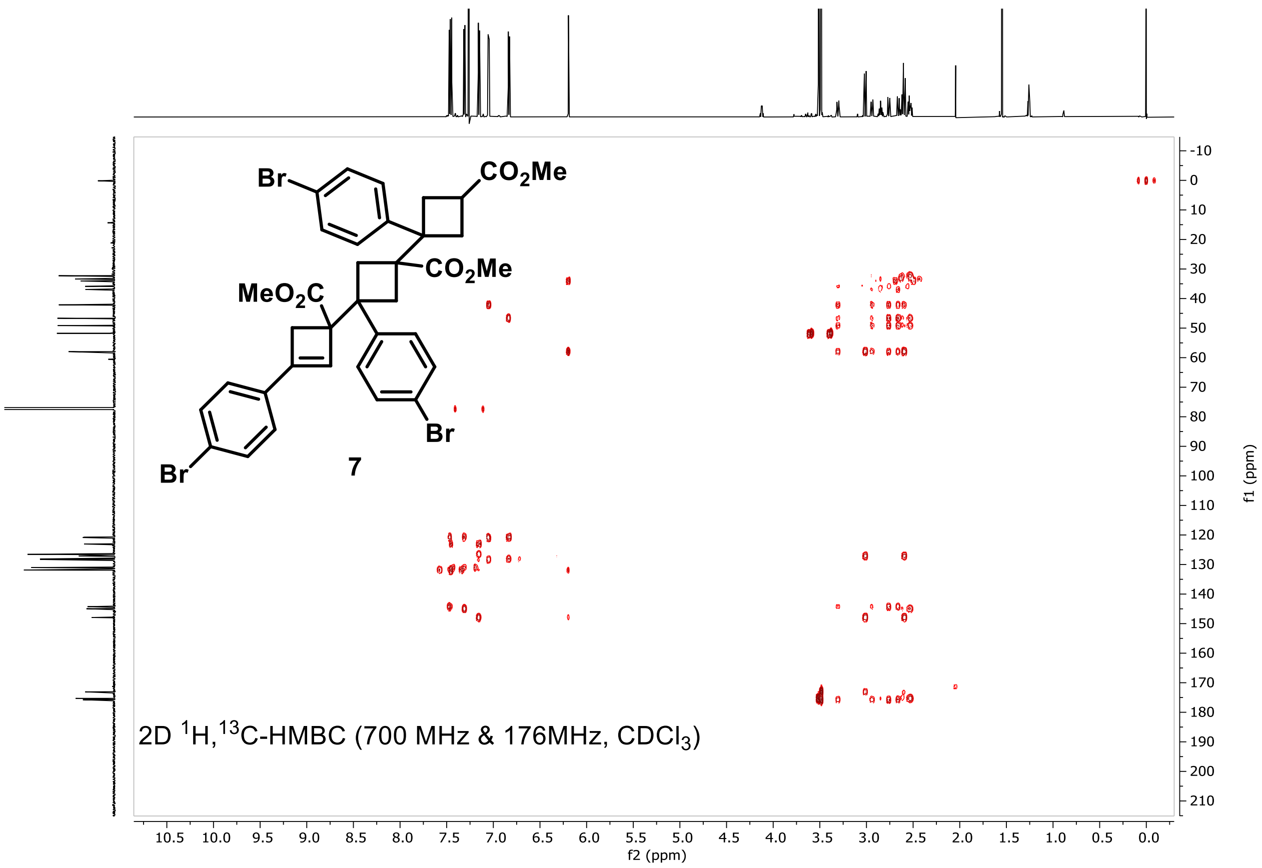

CO2Me
Br
CO2Me
MeO2C
Br
Br
7
2D 1H,13C-HMBC (700 MHz & 176MHz, CDCl3)
f1 (ppm)
f2 (ppm)

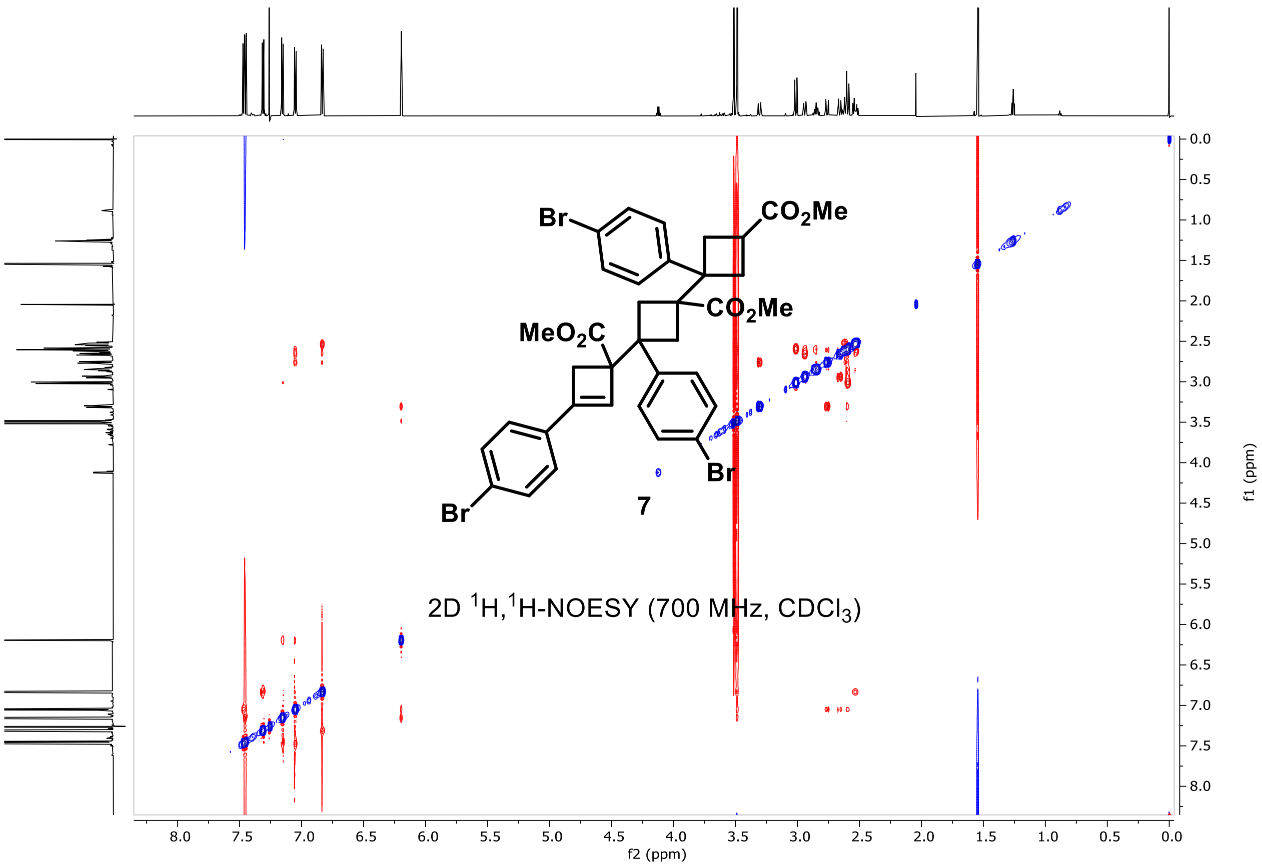

Br
CO2Me
CO2Me
MeO2C
Br
Br
7
2D 1H,1H-NOESY (700 MHz, CDCl3)
f1 (ppm)
f2 (ppm)

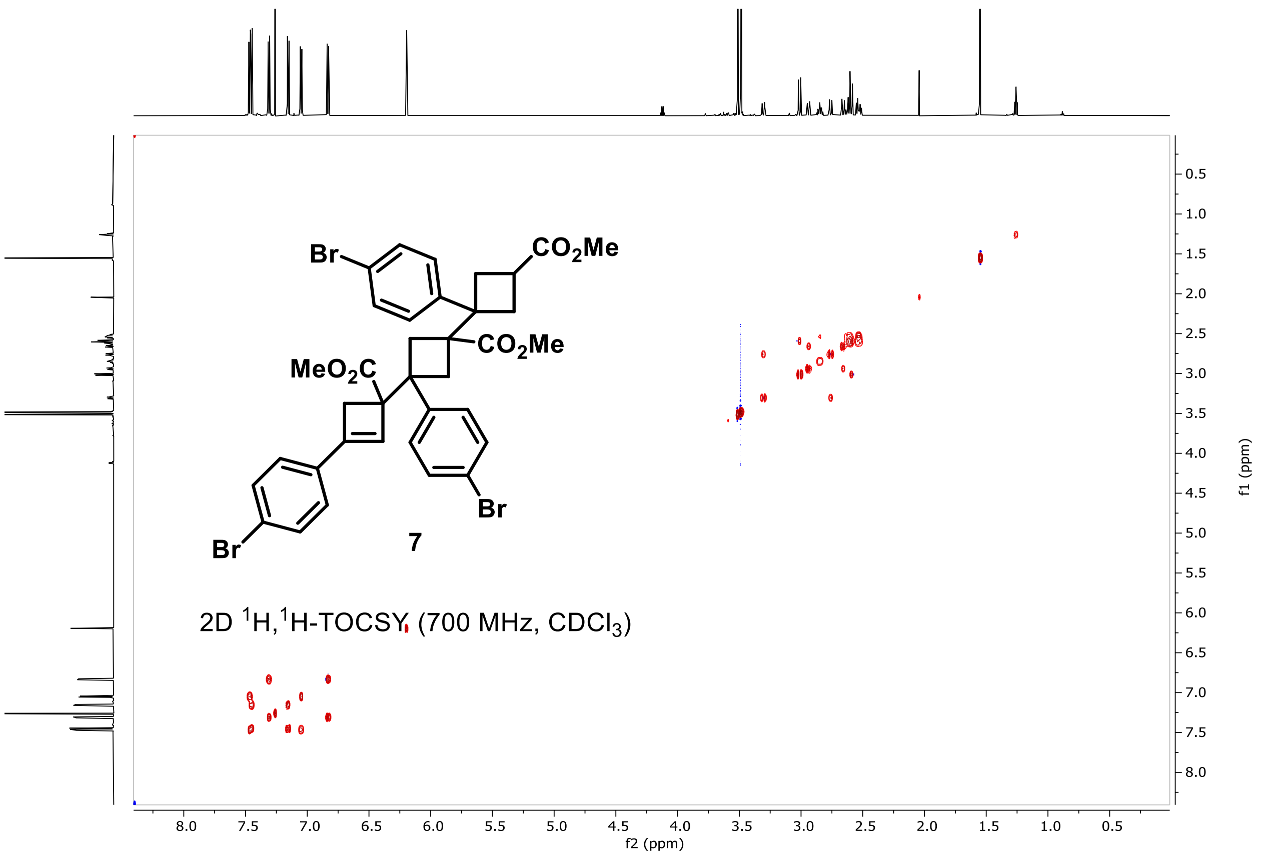
Br
CO2Me
CO2Me
MeO2C
Br
Br
7
2D 1H,1H-TOCSY (700 MHz, CDCl3)
f1 (ppm)
f2 (ppm)

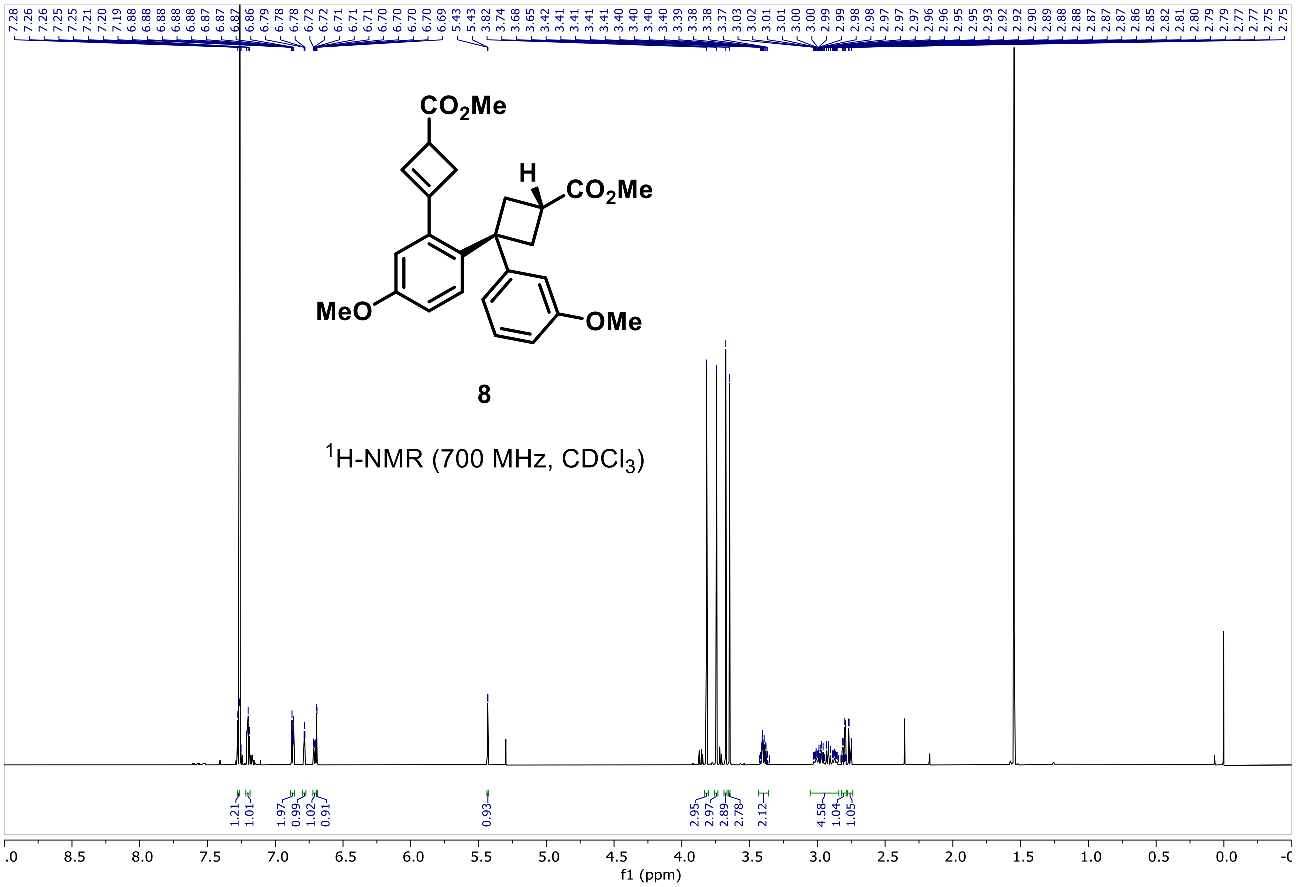
7.28 7.26 7.26 7.25 7.25 7.21 7.20 7.19 6.88 6.88 6.88 6.88 6.88 6.87 6.87 6.87 6.86 6.79 6.78 6.78 6.72 6.72 6.71 6.71 6.71 6.70 6.70 6.70 6.69 5.43 5.43 3.82 3.74 3.68 3.65 3.42 3.41 3.41 3.41 3.40 3.40 3.40 3.39 3.38 3.38 3.37 3.03 3.02 3.01 3.01 3.00 2.99 2.99 2.98 2.98 2.97 2.97 2.97 2.96 2.96 2.95 2.95 2.93 2.92 2.92 2.90 2.89 2.88 2.88 2.87 2.87 2.87 2.86 2.85 2.82 2.81 2.80 2.79 2.79 2.77 2.75 2.75
CO2Me
H
CO2Me
MeO
OMe
8
1H-NMR (700 MHz, CDCl3)
1.21 1.01 1.97 0.99 1.02 0.91 0.93 2.95 2.97 2.89 2.78 2.12 4.58 1.04 1.05
f1 (ppm)

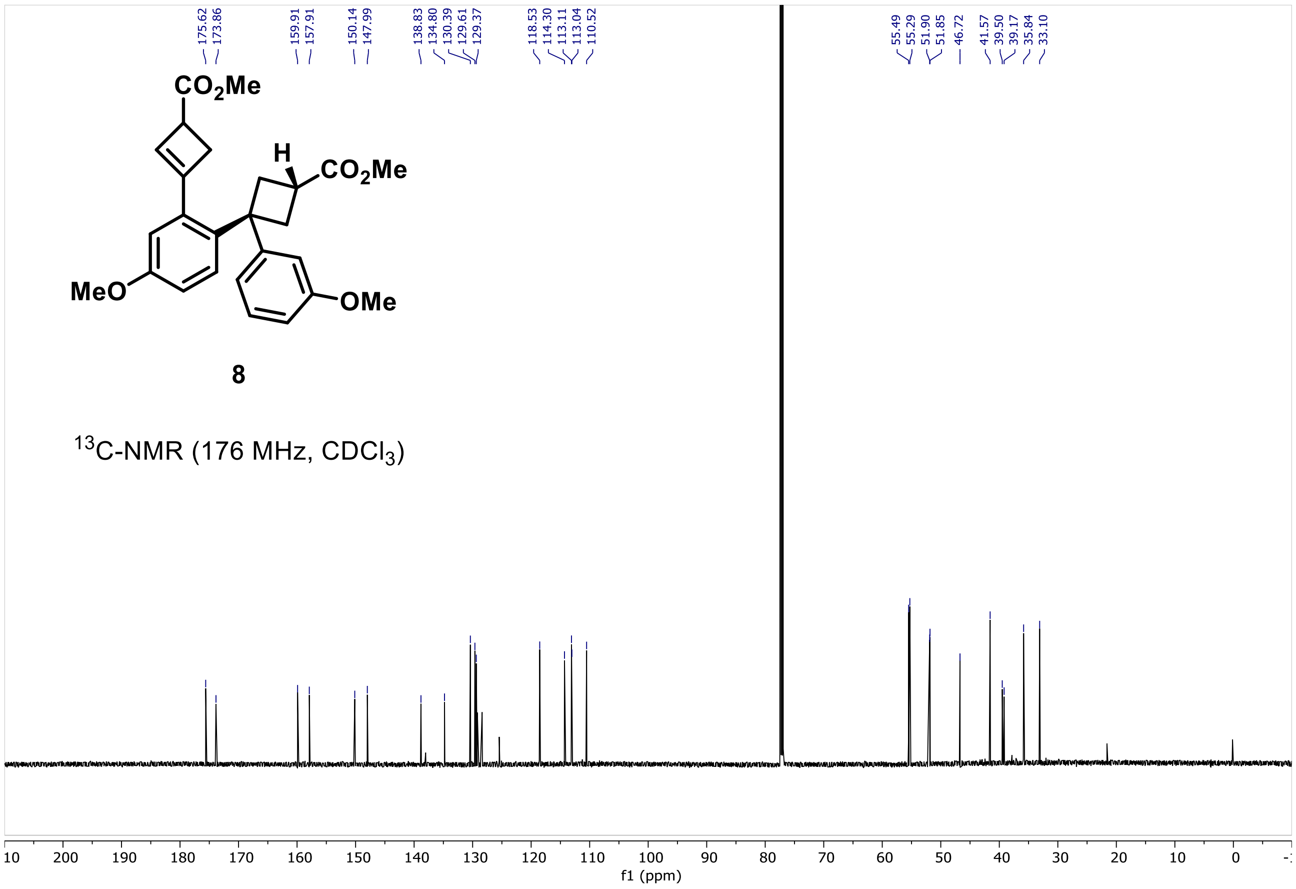
175.62 173.86 159.91 157.91 150.14 147.99 138.83 134.80 130.39 129.61 129.37 118.53 114.30 113.11 113.04 110.52 55.49 55.29 51.90 51.85 46.72 41.57 39.50 39.17 35.84 33.10
CO2Me
H
CO2Me
MeO
OMe
8
13C-NMR (176 MHz, CDCl3)
f1 (ppm)

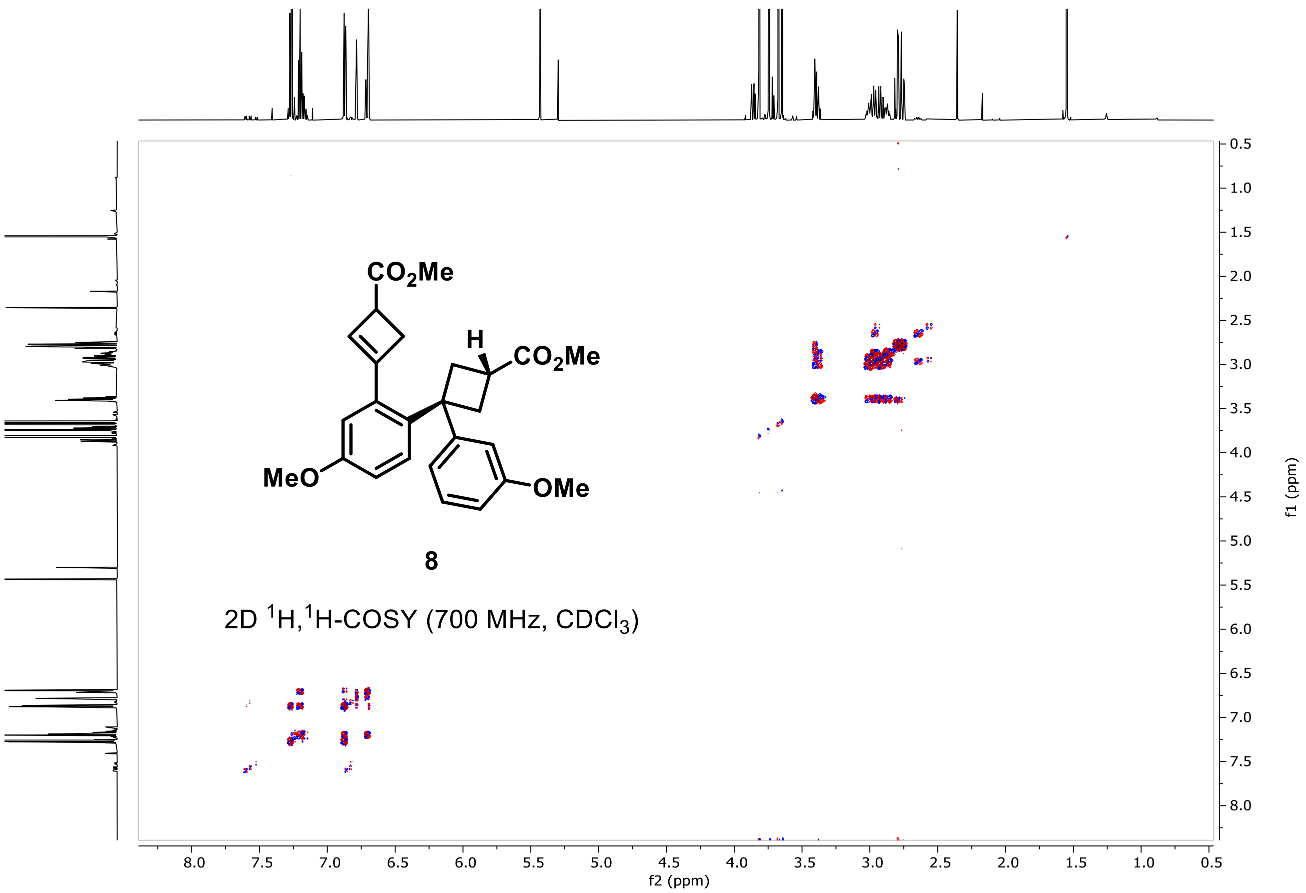
CO2Me
H
CO2Me
MeO
OMe
8
2D 1H,1H-COSY (700 MHz, CDCl3)
f2 (ppm)
f1 (ppm)

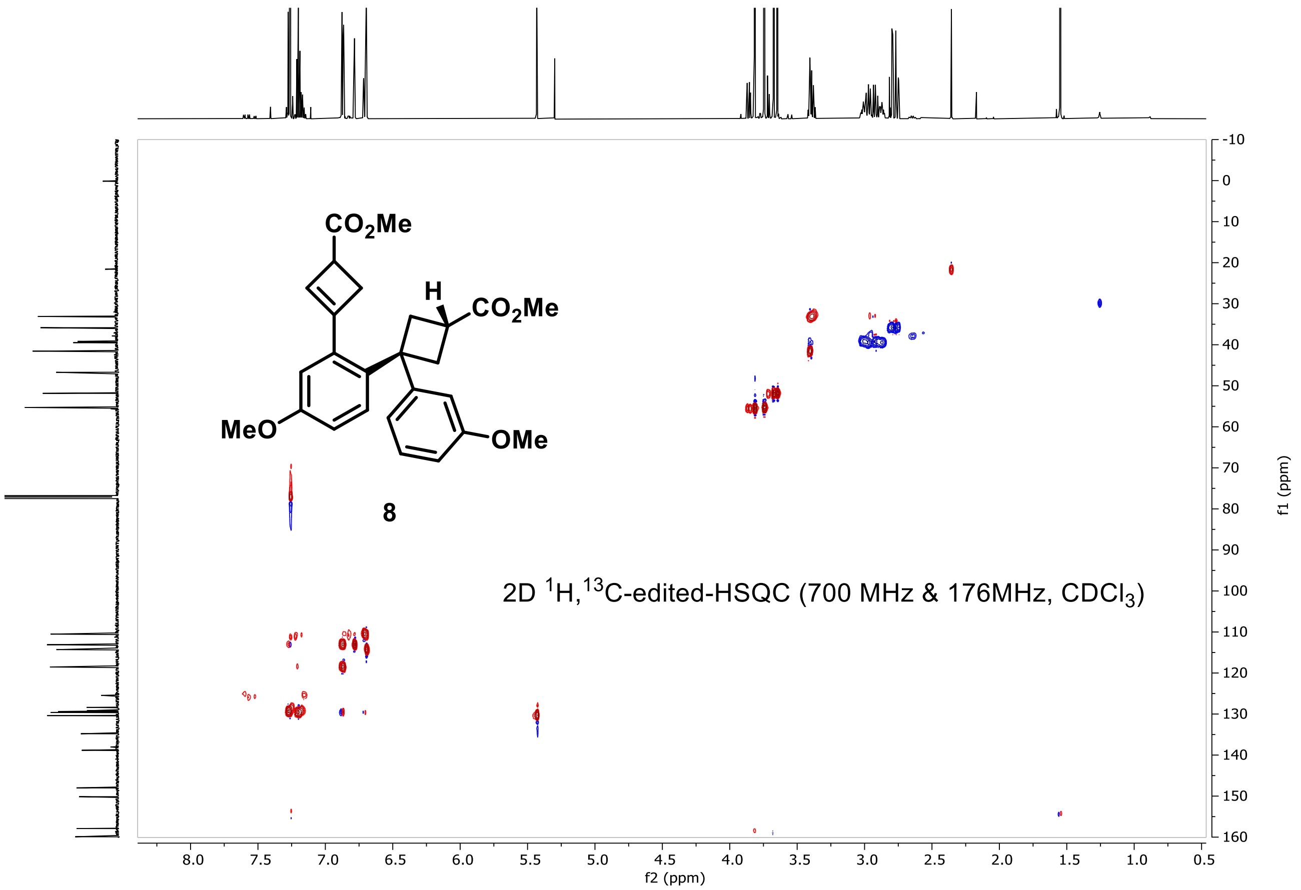
CO2Me
H
CO2Me
MeO
OMe
8
2D 1H,13C-edited-HSQC (700 MHz & 176MHz, CDCl3)
f2 (ppm)
f1 (ppm)

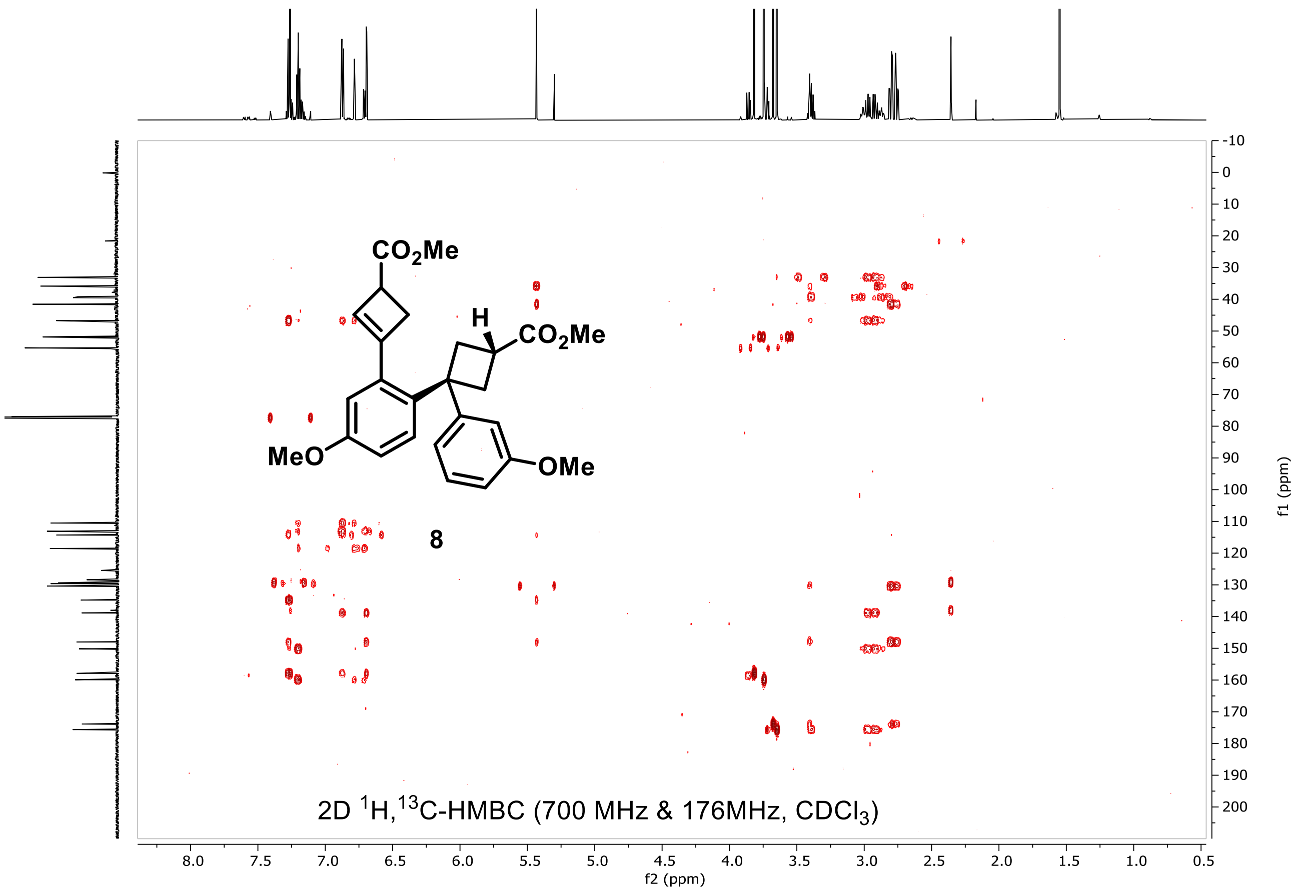

2D $^{1}H,^{13}C$-HMBC (700 MHz & 176MHz, $CDCl_3$)

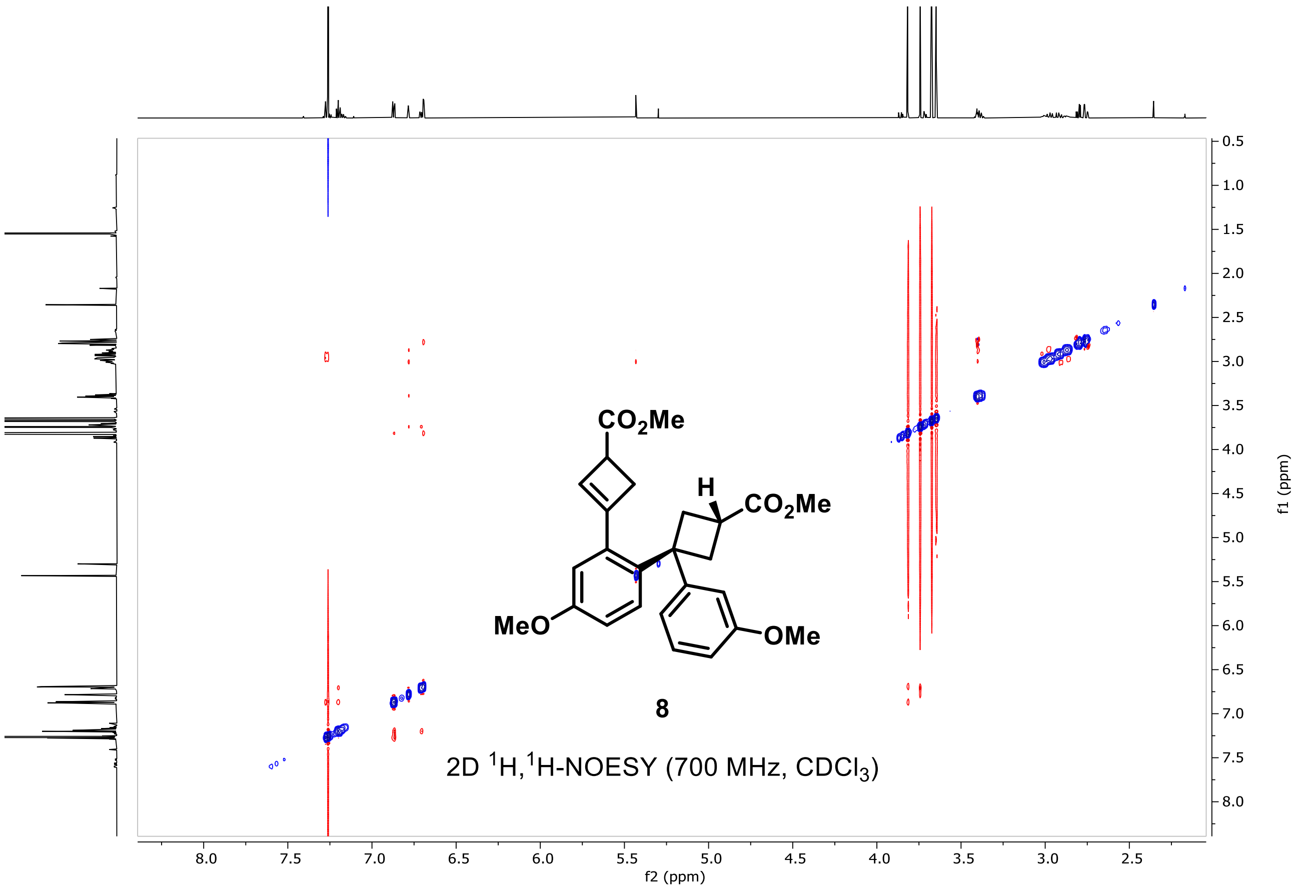

2D $^{1}H,^{1}H$-NOESY (700 MHz, $CDCl_3$)

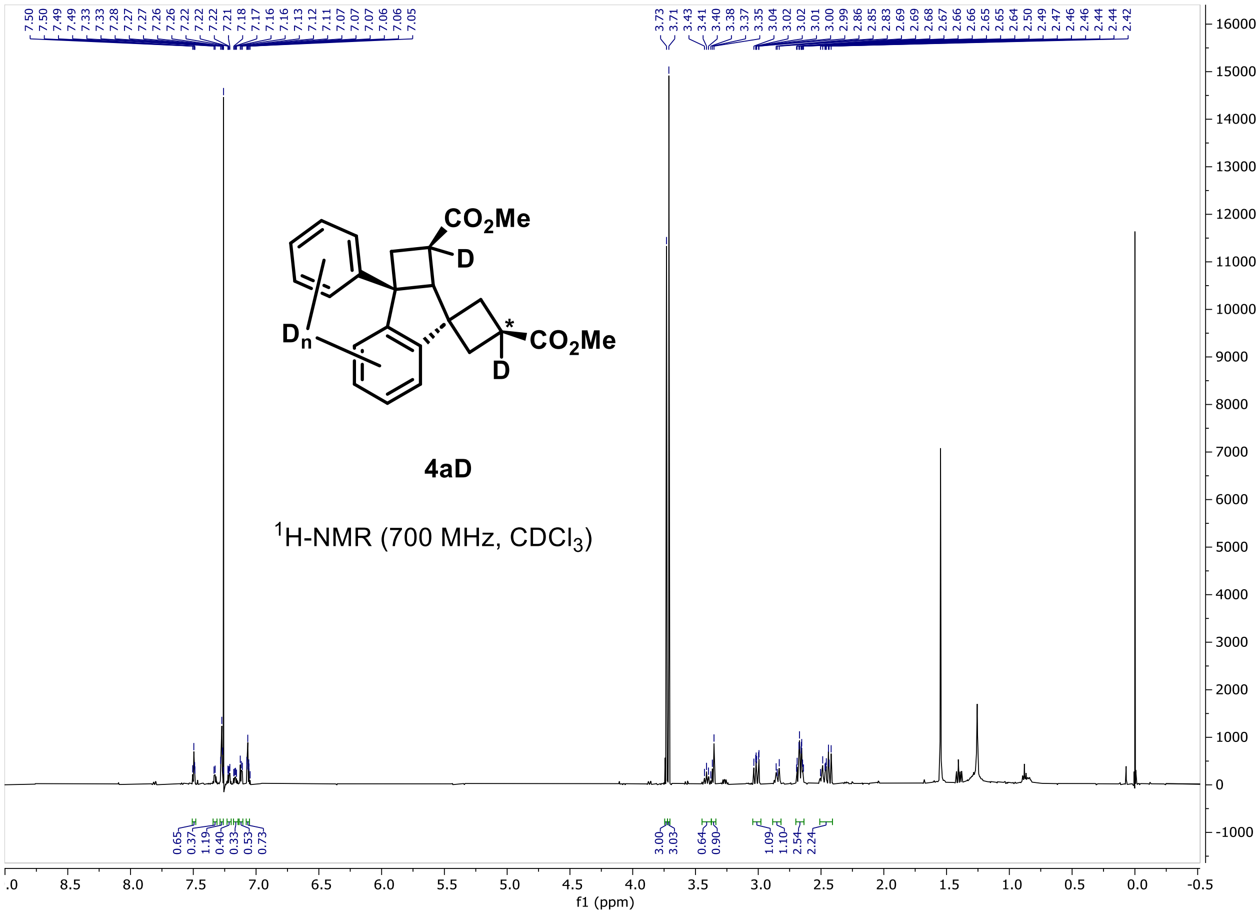

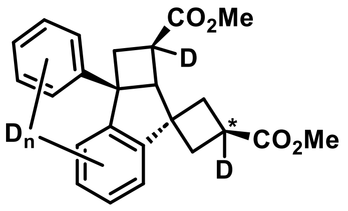

4aD

^{2}H-NMR (107 MHz, $CDCl_3$)

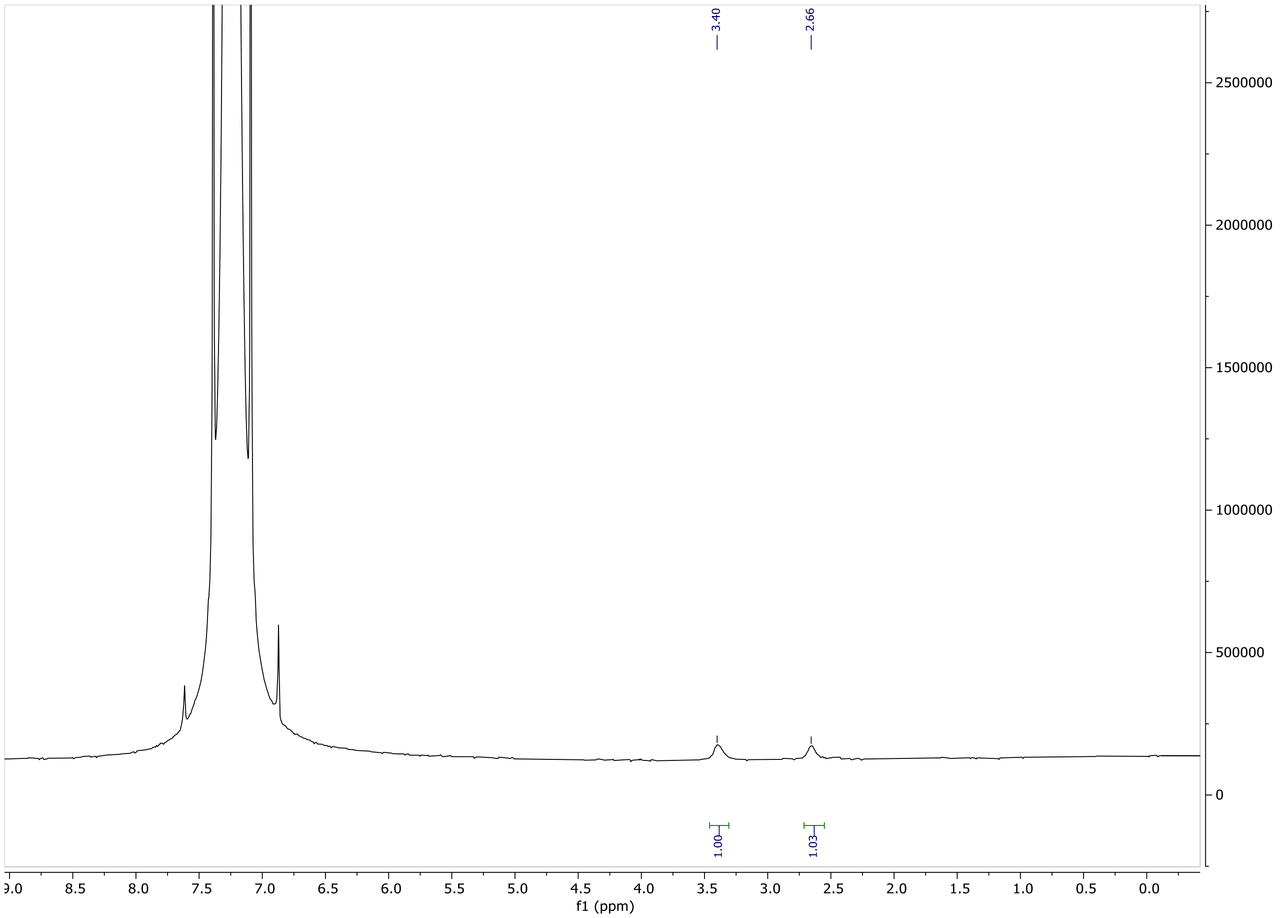
3.40
2.66
1.00
1.03
f1 (ppm)

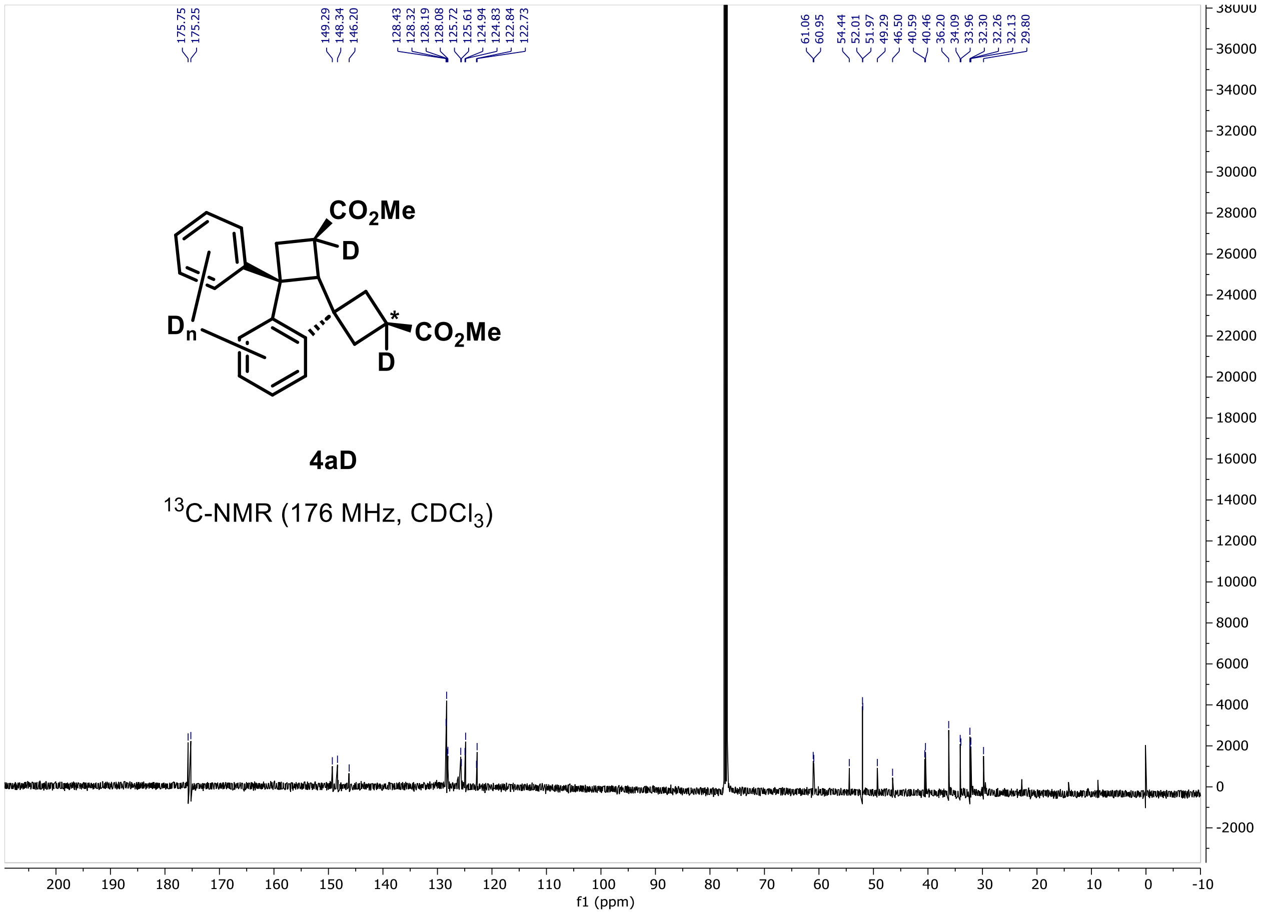

4aD

^{13}C-NMR (176 MHz, $CDCl_3$)

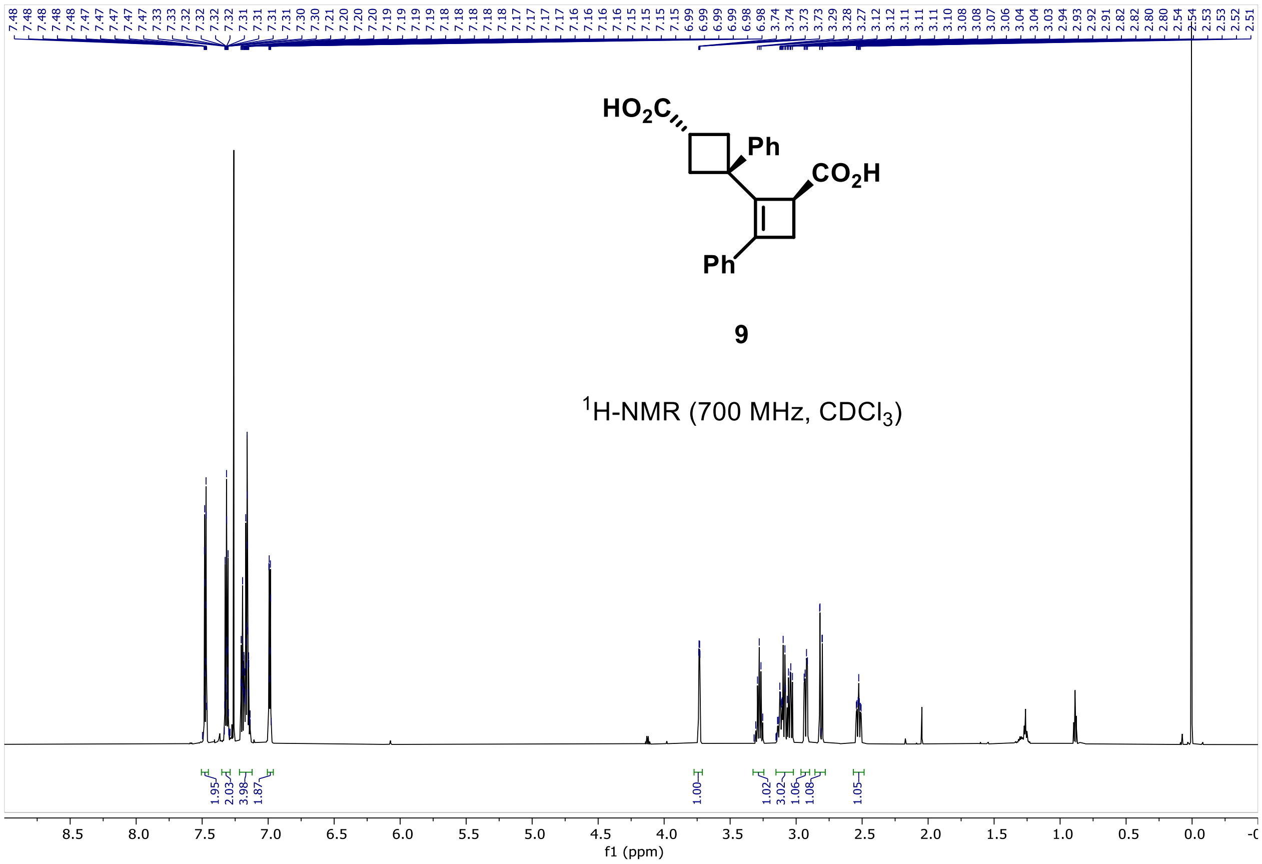
7.48 7.48 7.48 7.48 7.48 7.47 7.47 7.47 7.47 7.33 7.33 7.32 7.32 7.32 7.32 7.31 7.31 7.31 7.30 7.21 7.20 7.20 7.19 7.19 7.19 7.18 7.18 7.18 7.18 7.17 7.17 7.17 7.16 7.16 7.15 7.15 7.15 7.15 6.99 6.99 6.99 6.98 6.98 3.74 3.74 3.73 3.73 3.29 3.28 3.27 3.12 3.12 3.11 3.11 3.11 3.10 3.08 3.08 3.07 3.06 3.04 3.04 3.03 2.94 2.93 2.92 2.91 2.82 2.82 2.80 2.80 2.54 2.54 2.53 2.53 2.52 2.51
HO2C
Ph
CO2H
Ph
9
1H-NMR (700 MHz, CDCl3)
1.95 2.03 3.98 1.87
1.00
1.02 3.02 1.06 1.08
1.05
8.5 8.0 7.5 7.0 6.5 6.0 5.5 5.0 4.5 4.0 3.5 3.0 2.5 2.0 1.5 1.0 0.5 0.0
f1 (ppm)

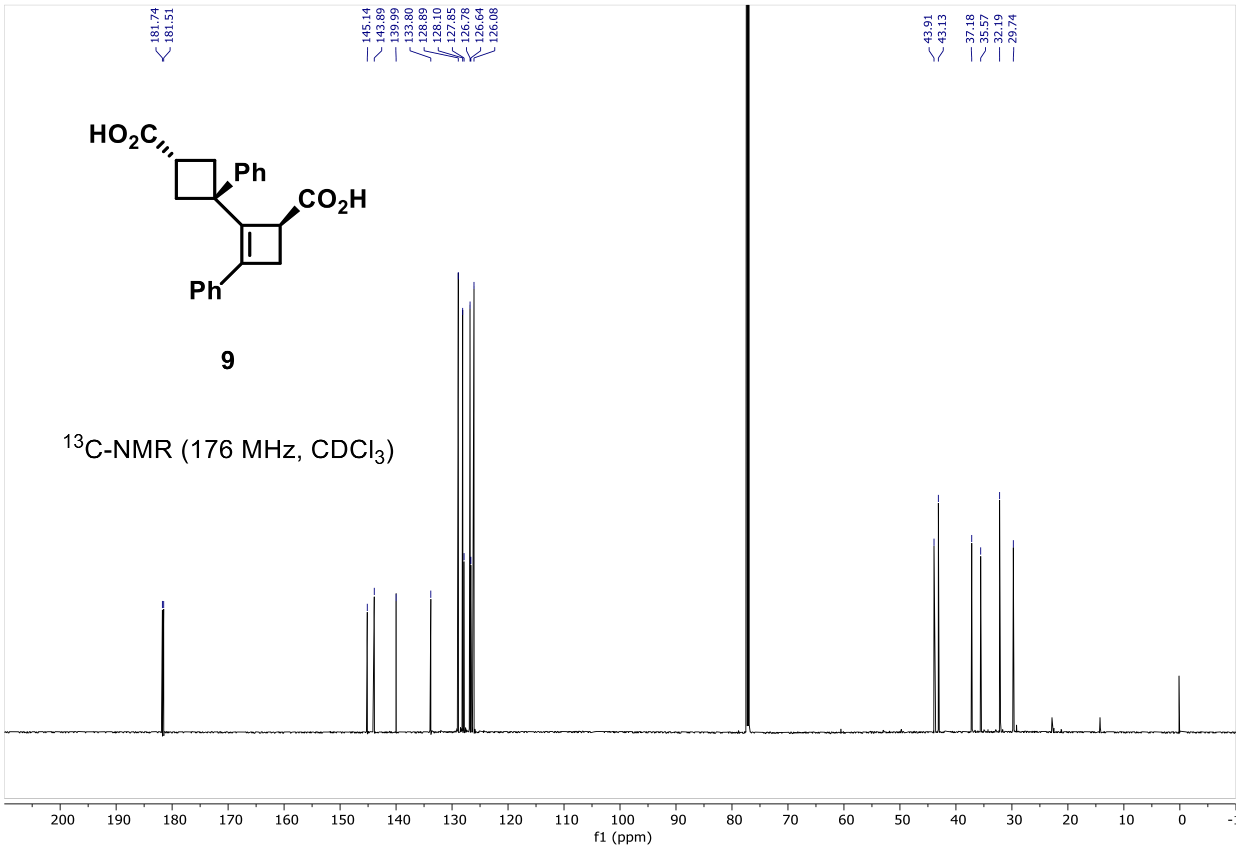
181.74 181.51
145.14 143.89 139.99 133.80 128.89 128.10 127.85 126.78 126.64 126.08
43.91 43.13 37.18 35.57 32.19 29.74
HO2C
Ph
CO2H
Ph
9
13C-NMR (176 MHz, CDCl3)
200 190 180 170 160 150 140 130 120 110 100 90 80 70 60 50 40 30 20 10 0
f1 (ppm)

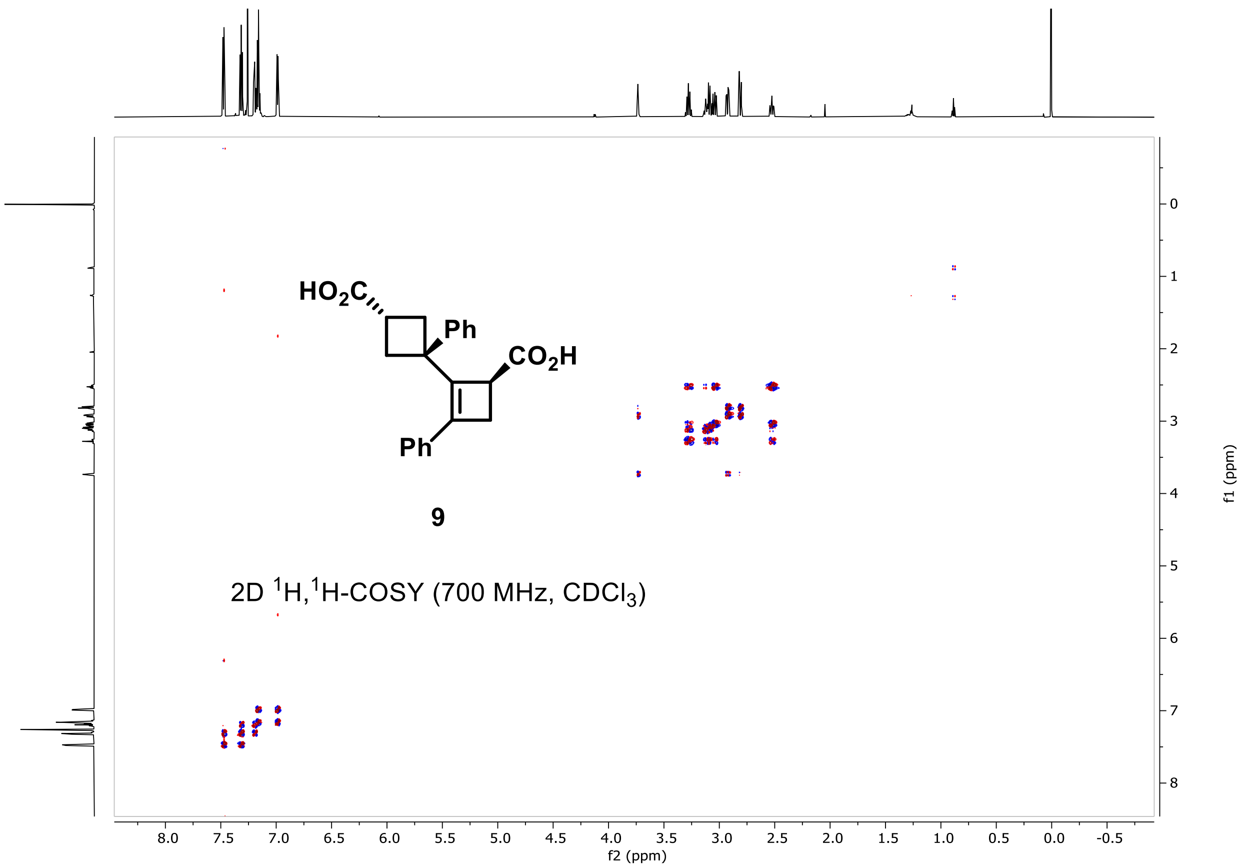

HO2C
Ph
CO2H
Ph
9
2D 1H,1H-COSY (700 MHz, CDCl3)
f2 (ppm)
f1 (ppm)

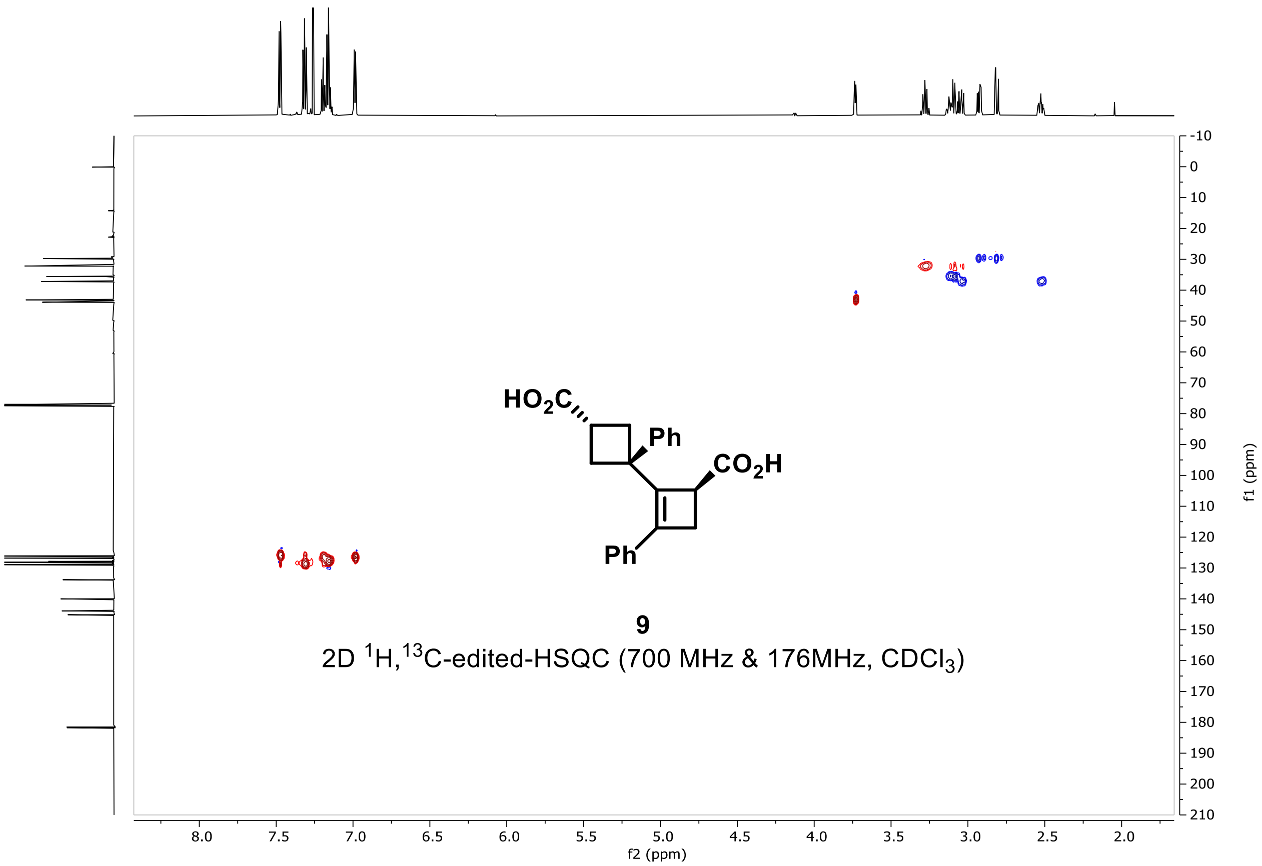

HO2C
Ph
CO2H
Ph
9
2D 1H,13C-edited-HSQC (700 MHz & 176MHz, CDCl3)
f2 (ppm)
f1 (ppm)

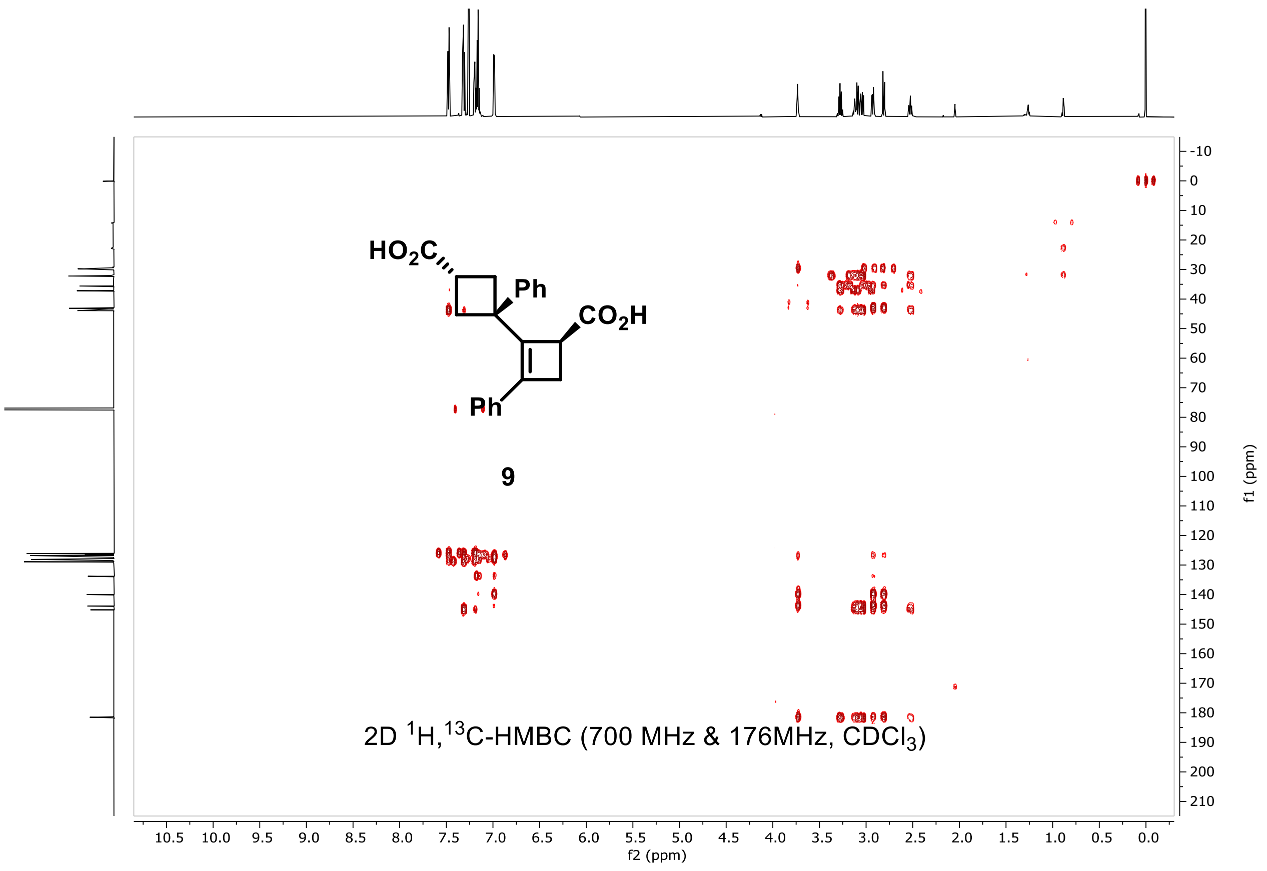
HO2C
Ph
CO2H
Ph
9
2D 1H,13C-HMBC (700 MHz & 176MHz, CDCl3)
f2 (ppm)
f1 (ppm)

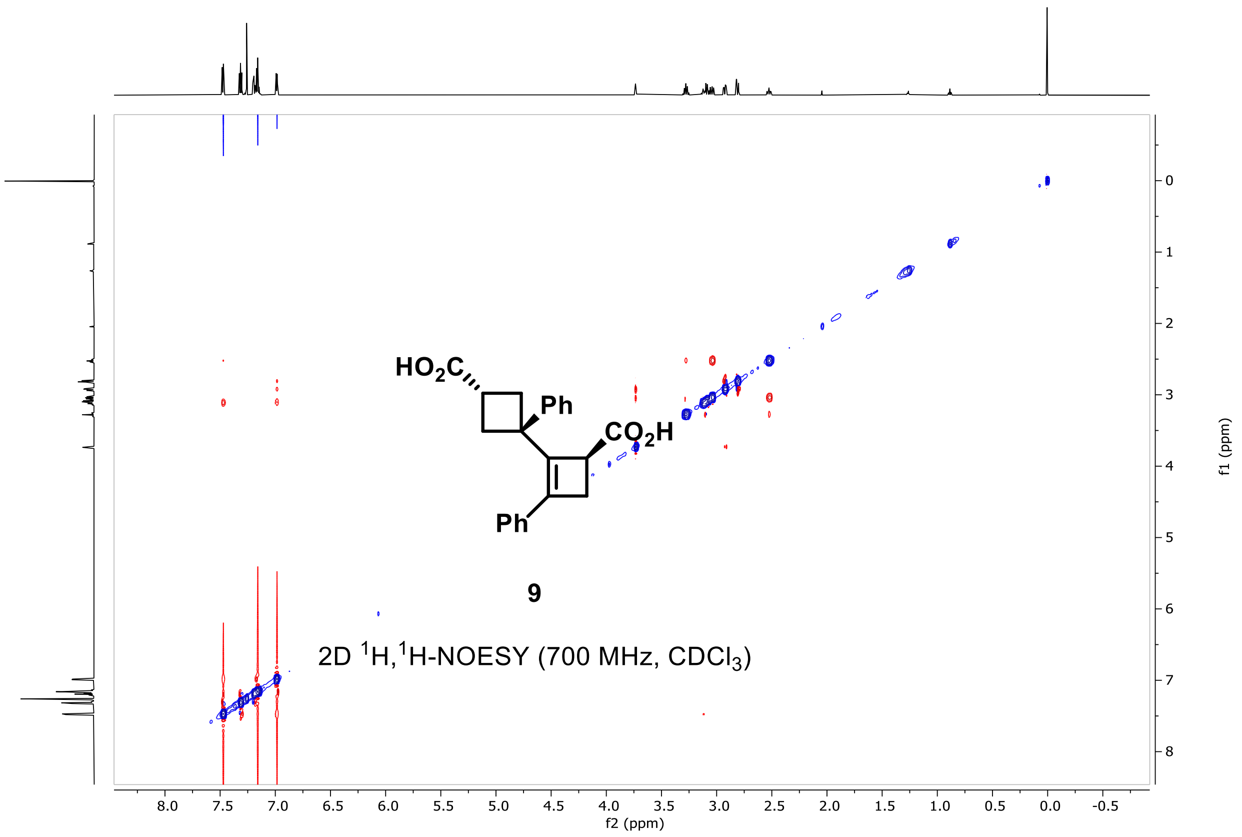
HO2C
Ph
CO2H
Ph
9
2D 1H,1H-NOESY (700 MHz, CDCl3)
f2 (ppm)
f1 (ppm)

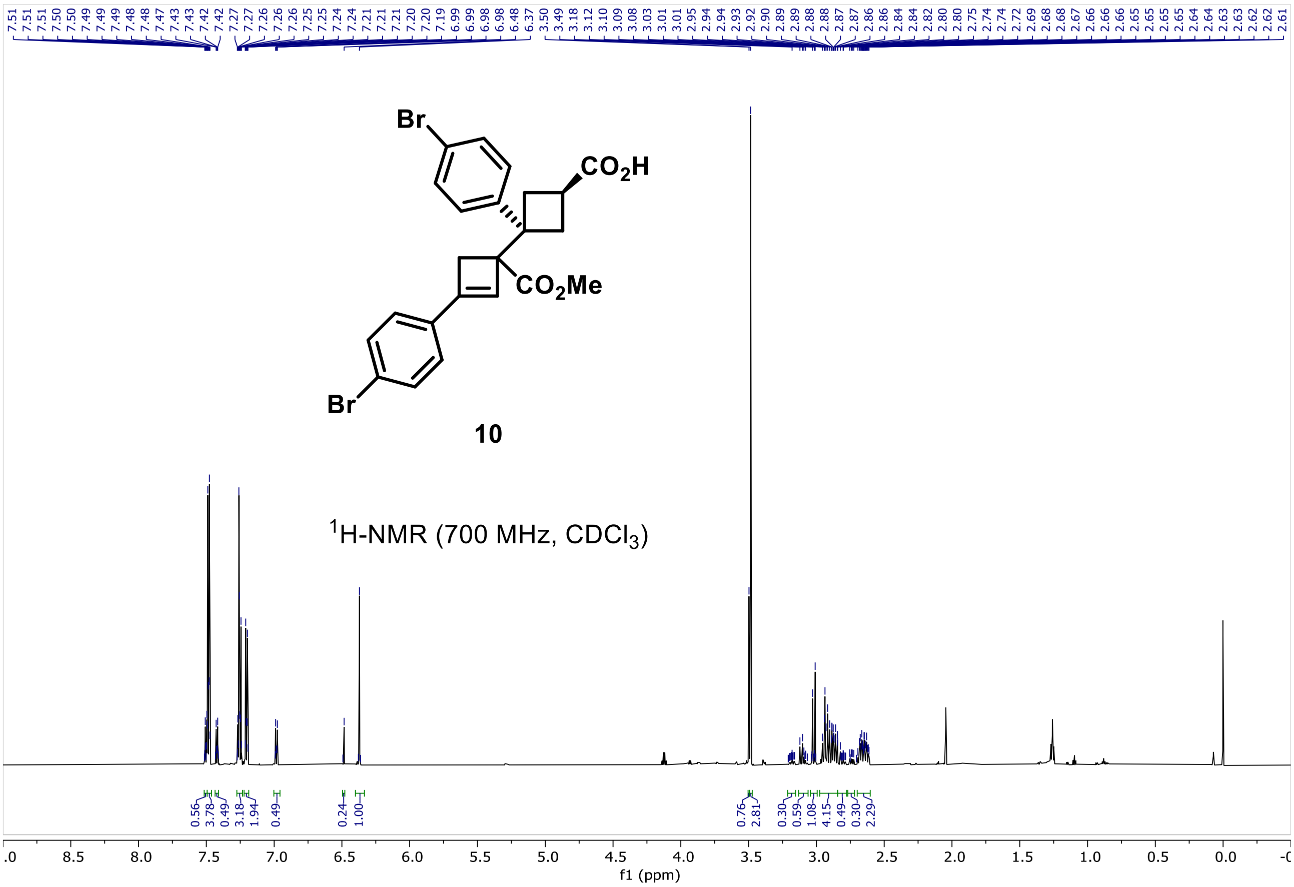
Br
CO2H
CO2Me
Br
10
1H-NMR (700 MHz, CDCl3)
f1 (ppm)

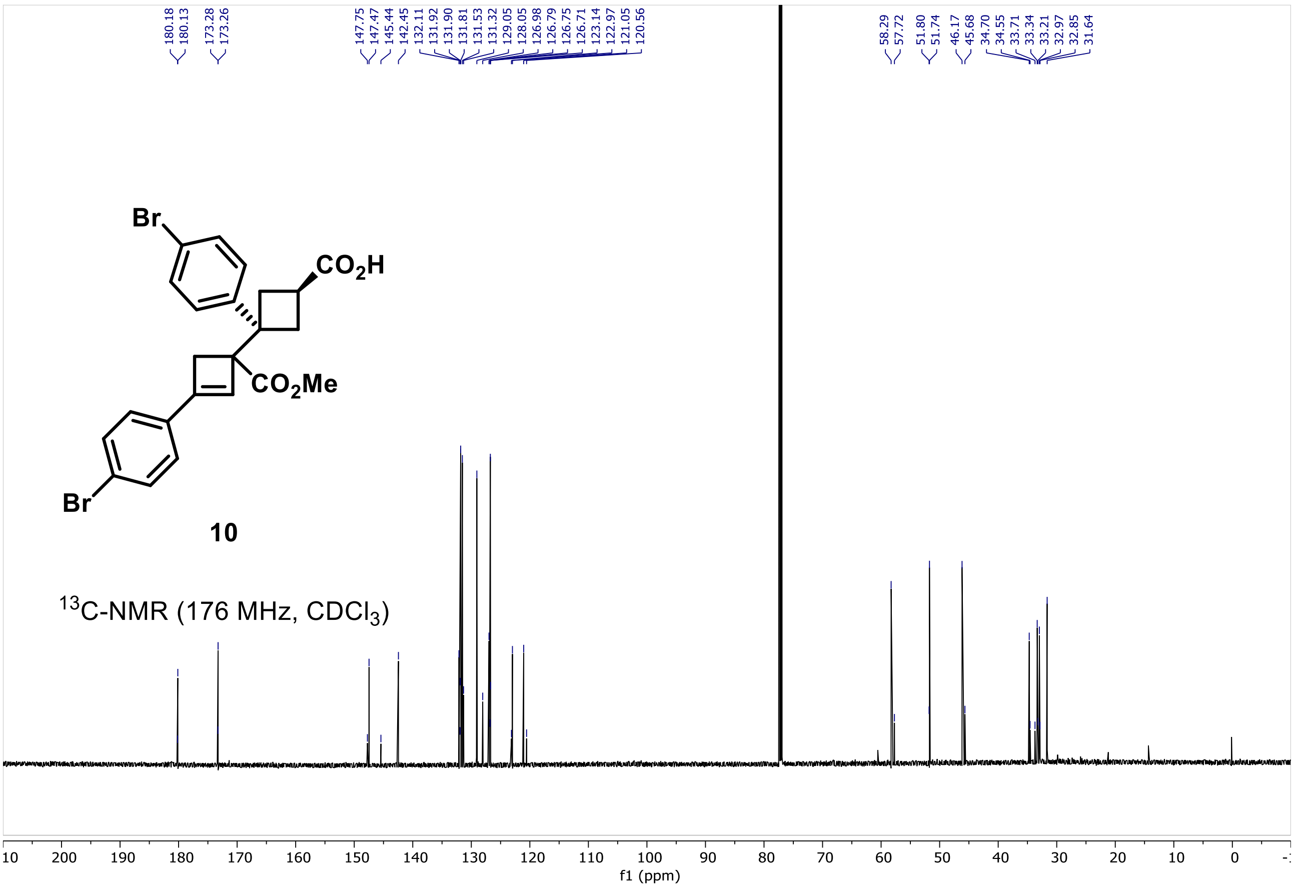
180.18
180.13
173.28
173.26
147.75
147.47
145.44
142.45
132.11
131.92
131.90
131.81
131.53
131.32
129.05
128.05
126.98
126.79
126.75
126.71
123.14
122.97
121.05
120.56
58.29
57.72
51.80
51.74
46.17
45.68
34.70
34.55
33.71
33.34
33.21
32.97
32.85
31.64
Br
CO2H
CO2Me
Br
10
13C-NMR (176 MHz, CDCl3)
f1 (ppm)

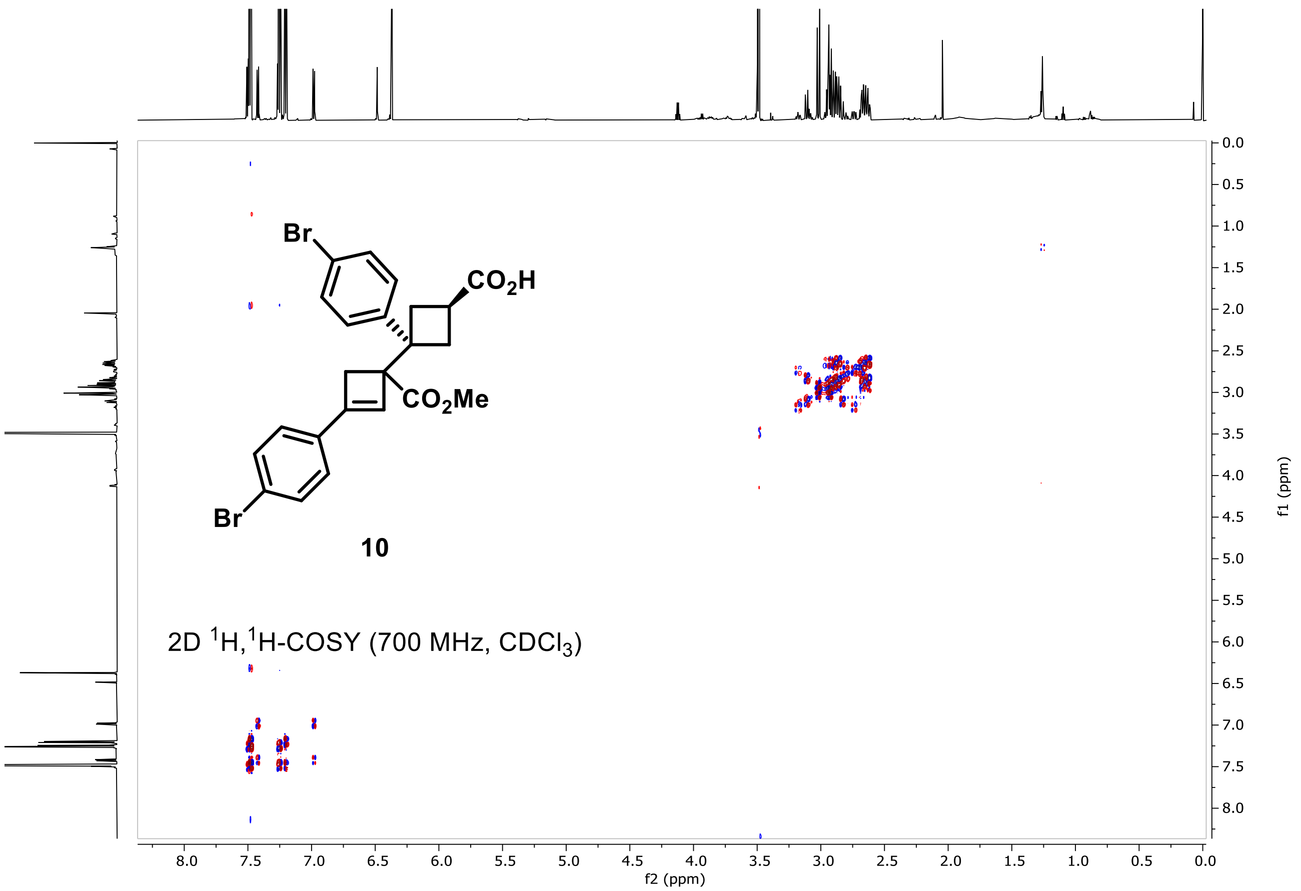
Br
CO2H
CO2Me
Br
10
2D 1H,1H-COSY (700 MHz, CDCl3)
f2 (ppm)
f1 (ppm)

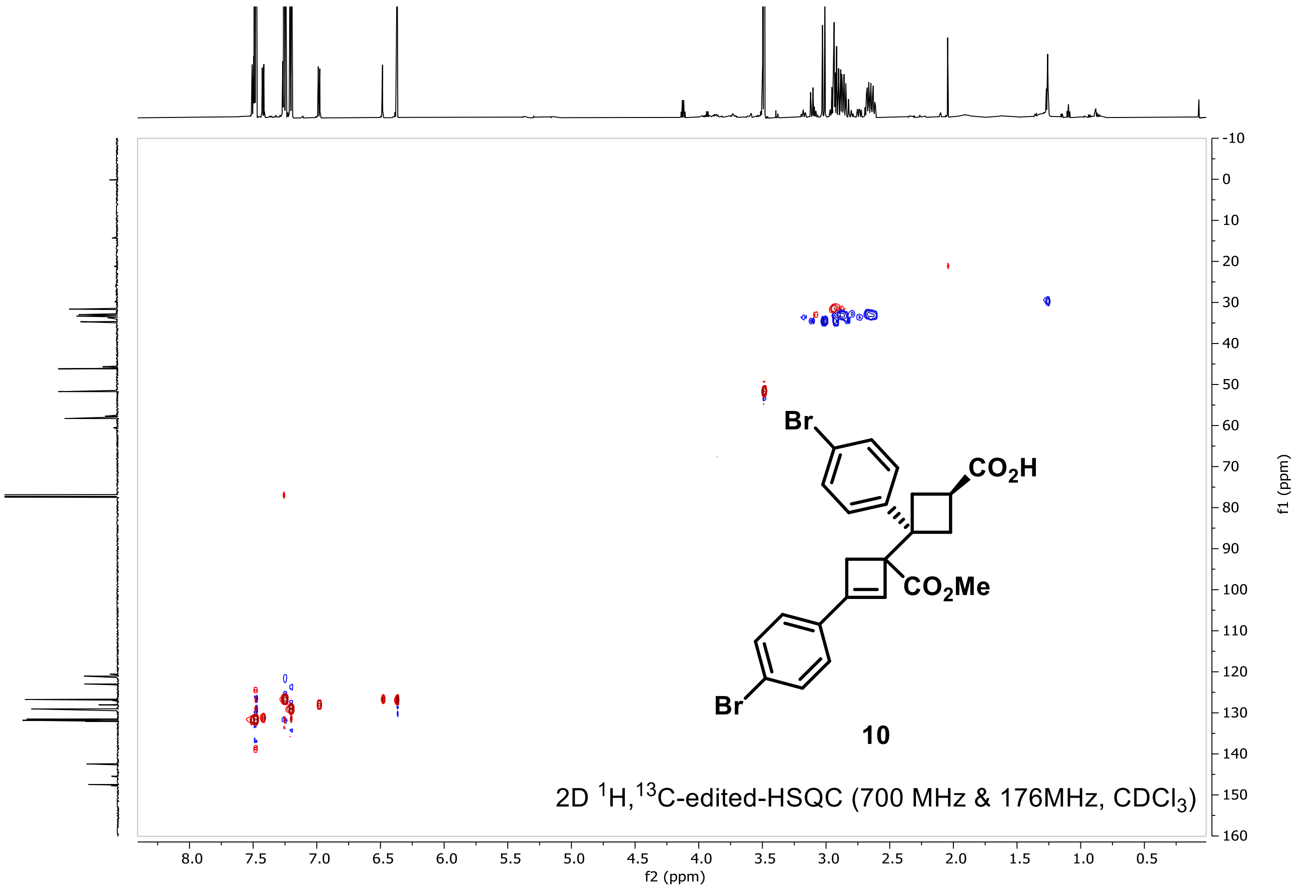
Br
CO2H
CO2Me
Br
10
2D 1H,13C-edited-HSQC (700 MHz & 176MHz, CDCl3)
f2 (ppm)
f1 (ppm)

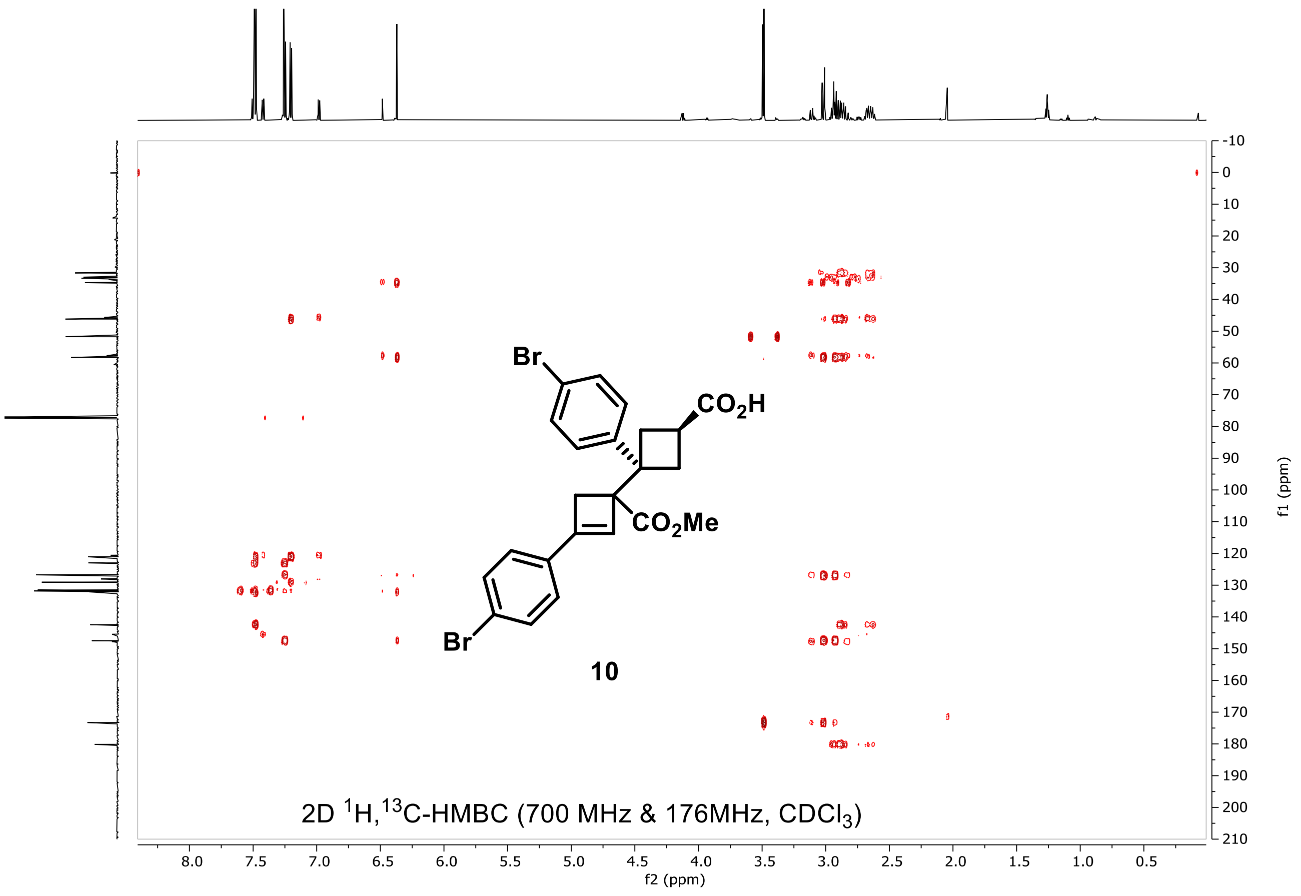
Br
CO2H
CO2Me
Br
10
2D 1H,13C-HMBC (700 MHz & 176MHz, CDCl3)
f2 (ppm)
f1 (ppm)

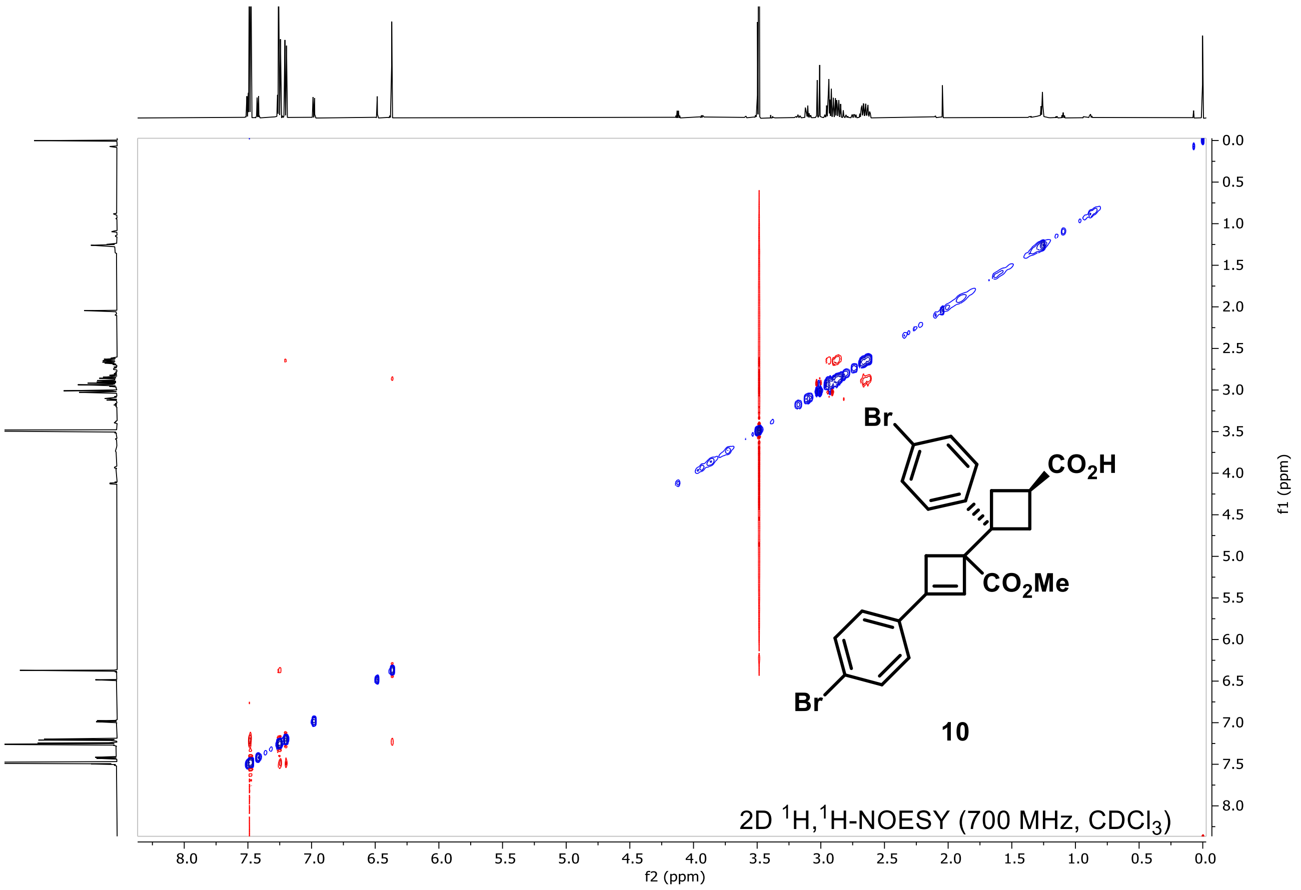
Br
CO2H
CO2Me
Br
10
2D 1H,1H-NOESY (700 MHz, CDCl3)
f2 (ppm)
f1 (ppm)

10. Crystal structure determinations

9.1 (2a'-phenyl-1',2',2a',7a'-tetrahydrospiro[cyclobutane-1,7'-cyclobuta[*a*]indene]-1',3-diyl)bis((3,5-dimethyl-1H-pyrazol-1-yl)methanone) (4l).

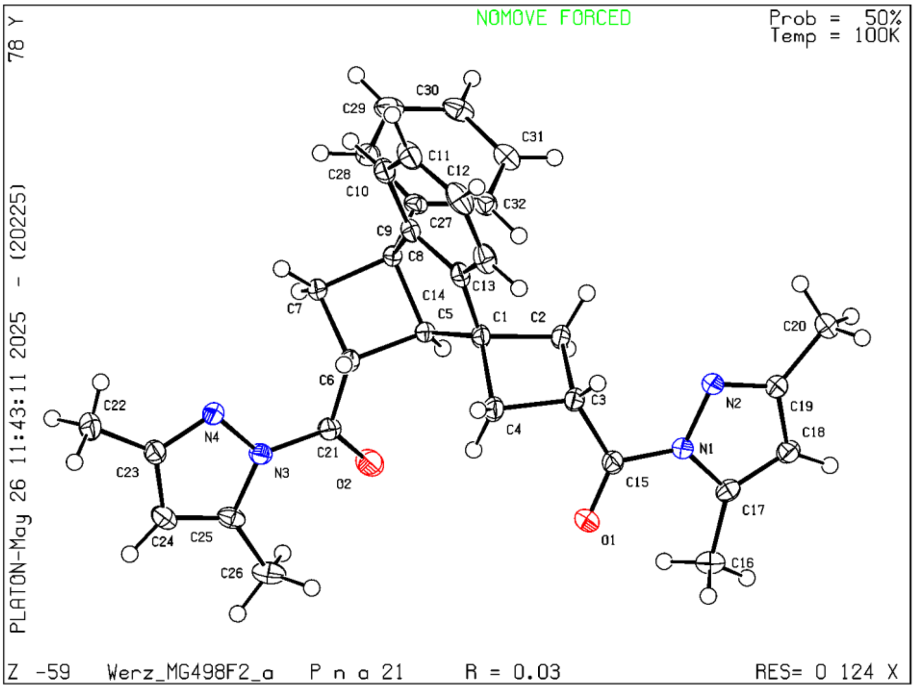

Crystals were obtained at room temperature by gas-phase diffusion of heptane into a solution of the compound dissolved in chloroform. A colourless, block-shaped crystal was mounted on a MiTeGen micromount with perfluoroether oil. Data for Werz_MG498F2_a were collected from a shock-cooled single crystal at 100(2) K on a Bruker D8 VENTURE dual wavelength Mo/Cu three-circle diffractometer with a microfocus sealed X-ray tube using a mirror optics as monochromator and a Bruker PHOTON III detector. The diffractometer was equipped with an Oxford Cryostream 800 low temperature device and used CuK_α radiation (λ = 1.54178 Å). All data were integrated with SAINT V8.41 and a multi-scan absorption correction using SADABS 2016/2 was applied.[17;18] The structure was solved by direct methods with SHELXT and refined by full-matrix least-squares methods against F^2 using SHELXL-2019/2.[19;20] All non-hydrogen atoms were refined with anisotropic displacement parameters. All hydrogen atoms were refined isotropic on calculated positions using a riding model with their U_{iso} values constrained to 1.5 times the U_{eq} of their pivot atoms for terminal sp^3 carbon atoms and 1.2 times for all other carbon atoms. Crystallographic data for the structures reported in this paper have been deposited with the

Cambridge Crystallographic Data Centre.[21] CCDC 2454075 contain the supplementary crystallographic data for this paper. These data can be obtained free of charge from The Cambridge Crystallographic Data Centre via www.ccdc.cam.ac.uk/structures. This report and the CIF file were generated using FinalCif.[22]

Table 1. Crystal data and structure refinement for Werz_MG498F2_a

**Compound**	**4l**
CCDC number	2454075
Empirical formula	$C_{32}H_{32}N_4O_2$
Formula weight	504.61
Temperature [K]	100(2)
Crystal system	orthorhombic
Space group (number)	$Pna2_1$ (33)
a [Å]	8.7218(6)
b [Å]	16.3416(11)
c [Å]	18.7045(14)
α [°]	90
β [°]	90
γ [°]	90
Volume [$Å^3$]	2665.9(3)
Z	4
ρ_{calc} [gcm^{-3}]	1.257
μ [mm^{-1}]	0.630
F(000)	1072
Crystal size [mm^3]	0.163×0.244×0.347
Crystal colour	colourless
Crystal shape	block
Radiation	CuK_α (λ=1.54178 Å)
2θ range [°]	7.18 to 149.25 (0.80 Å)
Index ranges	−10 ≤ h ≤ 10<br>−20 ≤ k ≤ 20<br>−23 ≤ l ≤ 23
Reflections collected	121929
Independent reflections	5441<br>R_{int} = 0.0419<br>R_{sigma} = 0.0156
Completeness to θ = 67.679°	100.0 %
Data / Restraints / Parameters	5441 / 1 / 347
Absorption correction T_{min}/T_{max} (method)	0.6179 / 0.7538 (multi-scan)
Goodness-of-fit on F^2	1.030
Final *R* indexes [$I \geq 2\sigma(I)$]	R_1 = 0.0252<br>wR_2 = 0.0661
Final *R* indexes [all data]	R_1 = 0.0253<br>wR_2 = 0.0662
Largest peak/hole [$eÅ^{-3}$]	0.17/−0.20
Flack X parameter	0.03(3)

Table 2. Atomic coordinates and U_{eq} [Å^2] for Werz_MG498F2_a

Atom	*x*	*y*	*z*	U_{eq}
O1	0.47237(16)	0.60296(7)	0.30002(7)	0.0292(3)
O2	0.22928(18)	0.42572(8)	0.45778(8)	0.0374(3)
N1	0.49273(16)	0.73854(8)	0.32614(7)	0.0197(3)
N2	0.56119(16)	0.79638(8)	0.36947(7)	0.0212(3)
N3	0.30980(15)	0.30681(8)	0.50656(7)	0.0183(3)
N4	0.39624(15)	0.26854(8)	0.55846(7)	0.0194(3)
C1	0.54378(18)	0.57633(9)	0.51088(8)	0.0188(3)
C2	0.5128(2)	0.66377(9)	0.47922(8)	0.0215(3)
H2A	0.402799	0.675889	0.471624	0.026
H2B	0.563534	0.708430	0.506046	0.026
C3	0.59898(19)	0.63839(9)	0.41005(8)	0.0208(3)
H3	0.705858	0.660526	0.409444	0.025
C4	0.5960(2)	0.54799(10)	0.43506(9)	0.0237(3)
H4A	0.697881	0.521315	0.434545	0.028
H4B	0.519041	0.514057	0.409846	0.028
C5	0.40874(17)	0.53255(9)	0.54575(8)	0.0168(3)
H5	0.306755	0.553135	0.529224	0.020
C6	0.41779(17)	0.43761(9)	0.55010(8)	0.0175(3)
H6	0.525665	0.418862	0.542363	0.021
C7	0.38210(17)	0.44261(9)	0.63160(8)	0.0177(3)
H7A	0.272984	0.433184	0.643634	0.021
H7B	0.450323	0.408557	0.661674	0.021
C8	0.42823(17)	0.53457(9)	0.62883(8)	0.0163(3)
C9	0.59949(17)	0.54979(8)	0.63500(9)	0.0180(3)
C10	0.68995(19)	0.54355(10)	0.69606(9)	0.0221(3)
H10	0.646652	0.526554	0.740230	0.027
C11	0.8451(2)	0.56270(10)	0.69114(10)	0.0268(4)
H11	0.908379	0.558602	0.732310	0.032
C12	0.90823(18)	0.58781(10)	0.62640(11)	0.0285(4)

H12	1.014025	0.601346	0.623928	0.034
C13	0.81813(19)	0.59325(10)	0.56547(10)	0.0254(3)
H13	0.861867	0.609953	0.521300	0.030
C14	0.66286(18)	0.57397(9)	0.56975(8)	0.0195(3)
C15	0.51746(19)	0.65534(10)	0.34074(8)	0.0209(3)
C16	0.3131(2)	0.72793(11)	0.21900(9)	0.0258(3)
H16A	0.244729	0.765620	0.193620	0.039
H16B	0.252079	0.685611	0.242837	0.039
H16C	0.383255	0.702201	0.184795	0.039
C17	0.40335(18)	0.77415(10)	0.27343(8)	0.0199(3)
C18	0.41637(19)	0.85619(10)	0.28376(9)	0.0216(3)
H18	0.368280	0.898078	0.256606	0.026
C19	0.51603(19)	0.86707(10)	0.34317(9)	0.0218(3)
C20	0.5718(2)	0.94526(10)	0.37558(10)	0.0293(4)
H20A	0.652152	0.968794	0.345260	0.044
H20B	0.613536	0.934274	0.423276	0.044
H20C	0.486354	0.983930	0.379444	0.044
C21	0.31065(18)	0.39226(9)	0.50117(9)	0.0208(3)
C22	0.4448(2)	0.12685(10)	0.59486(10)	0.0299(4)
H22A	0.499591	0.087140	0.565047	0.045
H22B	0.365639	0.098584	0.622657	0.045
H22C	0.517285	0.153441	0.627472	0.045
C23	0.37165(19)	0.18981(9)	0.54833(9)	0.0215(3)
C24	0.27112(19)	0.17631(10)	0.48964(9)	0.0229(3)
H24	0.237725	0.124831	0.471916	0.027
C25	0.23206(18)	0.25102(10)	0.46384(9)	0.0210(3)
C26	0.13035(19)	0.27330(11)	0.40292(9)	0.0277(4)
H26A	0.188576	0.305290	0.367889	0.042
H26B	0.043970	0.305909	0.420551	0.042
H26C	0.091589	0.223366	0.380221	0.042
C27	0.33595(17)	0.59426(9)	0.67279(8)	0.0176(3)
C28	0.28523(18)	0.57336(10)	0.74139(9)	0.0225(3)
H28	0.311107	0.521584	0.761073	0.027

C29	0.1973(2)	0.62791(11)	0.78079(9)	0.0273(4)
H29	0.163018	0.613065	0.827218	0.033
C30	0.15904(19)	0.70393(11)	0.75300(10)	0.0272(3)
H30	0.096750	0.740430	0.779814	0.033
C31	0.21217(19)	0.72645(10)	0.68582(9)	0.0243(3)
H31	0.188350	0.778874	0.666884	0.029
C32	0.30059(18)	0.67166(9)	0.64639(8)	0.0207(3)
H32	0.337471	0.687404	0.600617	0.025

U_{eq} is defined as 1/3 of the trace of the orthogonalised U_{ij} tensor.

Table 3. Anisotropic displacement parameters [Å^2] for Werz_MG498F2_a. The anisotropic displacement factor exponent takes the form: $-2\pi^2[\, h^2(a^*)^2U_{11} + k^2(b^*)^2U_{22} + \ldots + 2hka^*b^*U_{12} \,]$

Atom	U_{11}	U_{22}	U_{33}	U_{23}	U_{13}	U_{12}
O1	0.0465(7)	0.0187(5)	0.0224(6)	−0.0040(5)	−0.0003(5)	−0.0040(5)
O2	0.0536(8)	0.0226(6)	0.0360(7)	−0.0003(5)	−0.0247(6)	0.0038(6)
N1	0.0258(6)	0.0172(6)	0.0162(6)	−0.0001(5)	−0.0010(5)	−0.0028(5)
N2	0.0285(7)	0.0180(6)	0.0171(6)	−0.0002(5)	−0.0017(5)	−0.0048(5)
N3	0.0191(6)	0.0180(6)	0.0177(6)	−0.0016(5)	−0.0014(5)	−0.0003(5)
N4	0.0235(6)	0.0176(6)	0.0172(6)	0.0010(5)	0.0001(5)	0.0009(5)
C1	0.0255(8)	0.0127(6)	0.0181(7)	0.0016(5)	0.0038(6)	0.0018(6)
C2	0.0335(8)	0.0145(7)	0.0165(7)	0.0028(6)	0.0045(6)	0.0014(6)
C3	0.0288(8)	0.0161(7)	0.0175(7)	0.0018(6)	0.0042(6)	0.0005(6)
C4	0.0358(9)	0.0160(7)	0.0193(7)	0.0015(6)	0.0063(7)	0.0031(6)
C5	0.0209(7)	0.0144(6)	0.0151(7)	0.0001(5)	−0.0012(5)	0.0010(5)
C6	0.0192(7)	0.0149(7)	0.0183(7)	0.0017(5)	−0.0015(6)	0.0003(5)
C7	0.0214(7)	0.0147(6)	0.0170(7)	0.0015(5)	−0.0015(6)	−0.0030(5)
C8	0.0191(7)	0.0140(6)	0.0158(7)	0.0016(5)	−0.0020(6)	−0.0011(5)
C9	0.0201(7)	0.0125(6)	0.0214(7)	0.0006(5)	−0.0003(6)	0.0003(5)
C10	0.0251(8)	0.0155(7)	0.0257(8)	0.0013(6)	−0.0045(6)	−0.0010(6)
C11	0.0251(8)	0.0186(7)	0.0366(9)	0.0001(6)	−0.0104(7)	−0.0001(7)
C12	0.0176(7)	0.0207(7)	0.0472(10)	−0.0043(7)	0.0009(7)	−0.0021(6)
C13	0.0232(8)	0.0187(7)	0.0343(9)	0.0007(6)	0.0069(7)	−0.0017(6)
C14	0.0225(7)	0.0122(6)	0.0237(8)	0.0009(5)	0.0028(6)	0.0001(6)

C15	0.0278(8)	0.0174(7)	0.0176(7)	0.0004(6)	0.0062(6)	−0.0022(6)
C16	0.0257(8)	0.0301(9)	0.0214(8)	−0.0047(7)	−0.0023(6)	−0.0003(7)
C17	0.0199(7)	0.0241(7)	0.0157(7)	0.0008(6)	0.0026(6)	0.0002(6)
C18	0.0256(8)	0.0212(7)	0.0179(7)	0.0009(6)	−0.0007(6)	0.0030(6)
C19	0.0280(8)	0.0194(8)	0.0178(7)	0.0006(6)	0.0015(6)	−0.0019(6)
C20	0.0431(10)	0.0184(7)	0.0264(8)	−0.0007(7)	−0.0055(7)	−0.0042(7)
C21	0.0247(7)	0.0185(7)	0.0193(7)	−0.0006(6)	−0.0023(6)	0.0019(6)
C22	0.0452(10)	0.0172(7)	0.0272(8)	0.0022(7)	0.0027(8)	0.0023(7)
C23	0.0268(8)	0.0174(7)	0.0203(7)	−0.0006(6)	0.0069(6)	−0.0014(6)
C24	0.0240(8)	0.0201(7)	0.0246(8)	−0.0068(6)	0.0072(6)	−0.0059(6)
C25	0.0172(7)	0.0249(7)	0.0207(7)	−0.0077(6)	0.0034(6)	−0.0023(6)
C26	0.0223(8)	0.0350(9)	0.0258(9)	−0.0111(7)	−0.0045(7)	0.0013(7)
C27	0.0162(7)	0.0188(7)	0.0179(7)	−0.0031(6)	−0.0014(5)	−0.0045(6)
C28	0.0250(7)	0.0233(7)	0.0194(7)	−0.0011(6)	−0.0009(6)	−0.0056(6)
C29	0.0298(8)	0.0321(9)	0.0200(8)	−0.0067(7)	0.0047(7)	−0.0088(7)
C30	0.0249(8)	0.0284(8)	0.0282(8)	−0.0122(7)	0.0046(7)	−0.0027(7)
C31	0.0227(7)	0.0195(8)	0.0307(9)	−0.0050(6)	−0.0003(7)	−0.0009(6)
C32	0.0211(7)	0.0196(7)	0.0215(7)	−0.0013(6)	0.0022(6)	−0.0027(6)

Table 4. Bond lengths and angles for Werz_MG498F2_a

Atom–Atom	Length [Å]
O1–C15	1.211(2)
O2–C21	1.209(2)
N1–N2	1.3809(19)
N1–C17	1.385(2)
N1–C15	1.403(2)
N2–C19	1.316(2)
N3–N4	1.3791(18)
N3–C25	1.389(2)
N3–C21	1.4001(19)
N4–C23	1.318(2)
C1–C14	1.514(2)
C1–C5	1.525(2)
C1–C4	1.560(2)
C1–C2	1.5701(19)
C2–C3	1.553(2)
C2–H2A	0.9900
C2–H2B	0.9900
C3–C15	1.504(2)
C3–C4	1.550(2)
C3–H3	1.0000
C4–H4A	0.9900
C4–H4B	0.9900
C5–C6	1.5556(19)
C5–C8	1.563(2)
C5–H5	1.0000

C6–C21	1.503(2)
C6–C7	1.558(2)
C6–H6	1.0000
C7–C8	1.5565(19)
C7–H7A	0.9900
C7–H7B	0.9900
C8–C27	1.508(2)
C8–C9	1.519(2)
C9–C10	1.392(2)
C9–C14	1.397(2)
C10–C11	1.392(2)
C10–H10	0.9500
C11–C12	1.392(3)
C11–H11	0.9500
C12–C13	1.387(3)
C12–H12	0.9500
C13–C14	1.393(2)
C13–H13	0.9500
C16–C17	1.492(2)
C16–H16A	0.9800
C16–H16B	0.9800
C16–H16C	0.9800
C17–C18	1.359(2)
C18–C19	1.422(2)
C18–H18	0.9500
C19–C20	1.496(2)
C20–H20A	0.9800
C20–H20B	0.9800
C20–H20C	0.9800
C22–C23	1.491(2)
C22–H22A	0.9800
C22–H22B	0.9800
C22–H22C	0.9800
C23–C24	1.422(2)
C24–C25	1.356(2)
C24–H24	0.9500
C25–C26	1.489(2)
C26–H26A	0.9800
C26–H26B	0.9800
C26–H26C	0.9800
C27–C32	1.393(2)
C27–C28	1.399(2)
C28–C29	1.388(2)
C28–H28	0.9500
C29–C30	1.387(3)
C29–H29	0.9500
C30–C31	1.389(3)
C30–H30	0.9500
C31–C32	1.393(2)
C31–H31	0.9500
C32–H32	0.9500

**Atom–Atom–Atom**	**Angle [°]**
N2–N1–C17	111.93(12)
N2–N1–C15	118.84(13)
C17–N1–C15	129.21(13)
C19–N2–N1	104.60(13)
N4–N3–C25	111.97(13)
N4–N3–C21	120.01(12)
C25–N3–C21	128.01(14)
C23–N4–N3	104.62(12)
C14–C1–C5	101.93(12)
C14–C1–C4	116.96(13)
C5–C1–C4	118.37(13)
C14–C1–C2	114.55(13)

C5–C1–C2	117.10(13)	C8–C7–H7A	114.0
C4–C1–C2	88.71(11)	C6–C7–H7A	114.0
C3–C2–C1	89.30(11)	C8–C7–H7B	114.0
C3–C2–H2A	113.8	C6–C7–H7B	114.0
C1–C2–H2A	113.8	H7A–C7–H7B	111.2
C3–C2–H2B	113.8	C27–C8–C9	112.17(12)
C1–C2–H2B	113.8	C27–C8–C7	117.92(12)
H2A–C2–H2B	111.0	C9–C8–C7	114.19(12)
C15–C3–C4	115.32(13)	C27–C8–C5	119.83(12)
C15–C3–C2	116.12(13)	C9–C8–C5	100.72(12)
C4–C3–C2	89.71(11)	C7–C8–C5	89.12(11)
C15–C3–H3	111.4	C10–C9–C14	120.89(14)
C4–C3–H3	111.4	C10–C9–C8	127.44(14)
C2–C3–H3	111.4	C14–C9–C8	111.66(13)
C3–C4–C1	89.79(11)	C11–C10–C9	118.71(16)
C3–C4–H4A	113.7	C11–C10–H10	120.6
C1–C4–H4A	113.7	C9–C10–H10	120.6
C3–C4–H4B	113.7	C10–C11–C12	120.56(16)
C1–C4–H4B	113.7	C10–C11–H11	119.7
H4A–C4–H4B	110.9	C12–C11–H11	119.7
C1–C5–C6	116.82(12)	C13–C12–C11	120.62(15)
C1–C5–C8	109.35(12)	C13–C12–H12	119.7
C6–C5–C8	87.91(11)	C11–C12–H12	119.7
C1–C5–H5	113.4	C12–C13–C14	119.28(16)
C6–C5–H5	113.4	C12–C13–H13	120.4
C8–C5–H5	113.4	C14–C13–H13	120.4
C21–C6–C5	115.35(12)	C13–C14–C9	119.93(15)
C21–C6–C7	119.83(13)	C13–C14–C1	128.27(15)
C5–C6–C7	89.35(11)	C9–C14–C1	111.79(13)
C21–C6–H6	110.2	O1–C15–N1	120.82(15)
C5–C6–H6	110.2	O1–C15–C3	124.41(15)
C7–C6–H6	110.2	N1–C15–C3	114.76(13)
C8–C7–C6	88.07(11)	C17–C16–H16A	109.5

C17–C16–H16B	109.5
H16A–C16–H16B	109.5
C17–C16–H16C	109.5
H16A–C16–H16C	109.5
H16B–C16–H16C	109.5
C18–C17–N1	105.44(13)
C18–C17–C16	129.82(15)
N1–C17–C16	124.74(14)
C17–C18–C19	106.59(14)
C17–C18–H18	126.7
C19–C18–H18	126.7
N2–C19–C18	111.43(14)
N2–C19–C20	120.07(15)
C18–C19–C20	128.49(15)
C19–C20–H20A	109.5
C19–C20–H20B	109.5
H20A–C20–H20B	109.5
C19–C20–H20C	109.5
H20A–C20–H20C	109.5
H20B–C20–H20C	109.5
O2–C21–N3	119.76(14)
O2–C21–C6	123.42(14)
N3–C21–C6	116.81(13)
C23–C22–H22A	109.5
C23–C22–H22B	109.5
H22A–C22–H22B	109.5
C23–C22–H22C	109.5
H22A–C22–H22C	109.5
H22B–C22–H22C	109.5
N4–C23–C24	111.27(14)
N4–C23–C22	121.33(15)
C24–C23–C22	127.40(15)
C25–C24–C23	106.85(14)
C25–C24–H24	126.6
C23–C24–H24	126.6
C24–C25–N3	105.27(14)
C24–C25–C26	129.94(15)
N3–C25–C26	124.78(15)
C25–C26–H26A	109.5
C25–C26–H26B	109.5
H26A–C26–H26B	109.5
C25–C26–H26C	109.5
H26A–C26–H26C	109.5
H26B–C26–H26C	109.5
C32–C27–C28	118.48(14)
C32–C27–C8	120.81(13)
C28–C27–C8	120.71(14)
C29–C28–C27	120.32(16)
C29–C28–H28	119.8
C27–C28–H28	119.8
C30–C29–C28	120.61(15)
C30–C29–H29	119.7
C28–C29–H29	119.7
C29–C30–C31	119.73(15)

C29–C30–H30	120.1	C32–C31–H31	120.2
C31–C30–H30	120.1	C27–C32–C31	121.24(15)
C30–C31–C32	119.57(16)	C27–C32–H32	119.4
C30–C31–H31	120.2	C31–C32–H32	119.4

Table 5. Torsion angles for Werz_MG498F2_a

Atom–Atom–Atom–Atom	Torsion Angle [°]
C17–N1–N2–C19	−0.78(17)
C15–N1–N2–C19	−179.41(14)
C25–N3–N4–C23	−0.36(17)
C21–N3–N4–C23	−179.19(14)
C14–C1–C2–C3	107.41(14)
C5–C1–C2–C3	−133.31(14)
C4–C1–C2–C3	−11.82(12)
C1–C2–C3–C15	130.18(14)
C1–C2–C3–C4	11.89(13)
C15–C3–C4–C1	−130.96(14)
C2–C3–C4–C1	−11.97(13)
C14–C1–C4–C3	−105.21(14)
C5–C1–C4–C3	132.22(14)
C2–C1–C4–C3	11.84(12)
C14–C1–C5–C6	−77.33(15)
C4–C1–C5–C6	52.52(19)
C2–C1–C5–C6	156.86(13)
C14–C1–C5–C8	20.39(15)
C4–C1–C5–C8	150.24(13)
C2–C1–C5–C8	−105.42(15)
C1–C5–C6–C21	−108.64(16)
C8–C5–C6–C21	140.68(13)
C1–C5–C6–C7	128.33(13)
C8–C5–C6–C7	17.65(11)
C21–C6–C7–C8	−136.88(13)
C5–C6–C7–C8	−17.73(11)
C6–C7–C8–C27	141.35(13)
C6–C7–C8–C9	−83.72(14)
C6–C7–C8–C5	17.64(11)
C1–C5–C8–C27	102.50(14)
C6–C5–C8–C27	−139.75(13)
C1–C5–C8–C9	−20.96(15)
C6–C5–C8–C9	96.80(11)
C1–C5–C8–C7	−135.43(12)
C6–C5–C8–C7	−17.67(11)
C27–C8–C9–C10	63.6(2)
C7–C8–C9–C10	−73.9(2)
C5–C8–C9–C10	−167.81(14)
C27–C8–C9–C14	−115.11(14)
C7–C8–C9–C14	107.38(14)
C5–C8–C9–C14	13.49(15)
C14–C9–C10–C11	0.6(2)
C8–C9–C10–C11	−177.96(14)
C9–C10–C11–C12	0.2(2)
C10–C11–C12–C13	−0.8(3)
C11–C12–C13–C14	0.6(2)

C12–C13–C14–C9	0.3(2)	C25–N3–C21–O2	4.8(3)
C12–C13–C14–C1	179.36(15)	N4–N3–C21–C6	4.6(2)
C10–C9–C14–C13	−0.9(2)	C25–N3–C21–C6	−174.05(14)
C8–C9–C14–C13	177.94(13)	C5–C6–C21–O2	4.6(2)
C10–C9–C14–C1	179.90(13)	C7–C6–C21–O2	109.52(19)
C8–C9–C14–C1	−1.30(17)	C5–C6–C21–N3	−176.58(13)
C5–C1–C14–C13	168.94(15)	C7–C6–C21–N3	−71.67(18)
C4–C1–C14–C13	38.2(2)	N3–N4–C23–C24	0.67(17)
C2–C1–C14–C13	−63.6(2)	N3–N4–C23–C22	−179.46(14)
C5–C1–C14–C9	−11.90(15)	N4–C23–C24–C25	−0.75(18)
C4–C1–C14–C9	−142.61(13)	C22–C23–C24–C25	179.39(16)
C2–C1–C14–C9	115.58(14)	C23–C24–C25–N3	0.48(17)
N2–N1–C15–O1	−172.57(14)	C23–C24–C25–C26	179.88(16)
C17–N1–C15–O1	9.1(3)	N4–N3–C25–C24	−0.09(17)
N2–N1–C15–C3	8.8(2)	C21–N3–C25–C24	178.62(15)
C17–N1–C15–C3	−169.60(15)	N4–N3–C25–C26	−179.54(14)
C4–C3–C15–O1	−11.4(2)	C21–N3–C25–C26	−0.8(2)
C2–C3–C15–O1	−114.46(18)	C9–C8–C27–C32	82.98(17)
C4–C3–C15–N1	167.21(13)	C7–C8–C27–C32	−141.24(15)
C2–C3–C15–N1	64.16(18)	C5–C8–C27–C32	−34.7(2)
N2–N1–C17–C18	0.19(17)	C9–C8–C27–C28	−96.38(16)
C15–N1–C17–C18	178.64(15)	C7–C8–C27–C28	39.4(2)
N2–N1–C17–C16	−179.12(14)	C5–C8–C27–C28	145.90(14)
C15–N1–C17–C16	−0.7(3)	C32–C27–C28–C29	2.2(2)
N1–C17–C18–C19	0.44(17)	C8–C27–C28–C29	−178.46(14)
C16–C17–C18–C19	179.70(16)	C27–C28–C29–C30	−0.3(2)
N1–N2–C19–C18	1.05(18)	C28–C29–C30–C31	−1.5(3)
N1–N2–C19–C20	−178.22(15)	C29–C30–C31–C32	1.4(2)
C17–C18–C19–N2	−0.98(19)	C28–C27–C32–C31	−2.3(2)
C17–C18–C19–C20	178.22(17)	C8–C27–C32–C31	178.35(14)
N4–N3–C21–O2	−176.58(16)	C30–C31–C32–C27	0.5(2)

9.2 2a'-phenyl-1',2',2a',7a'-tetrahydrospiro[cyclobutane-1,7'-cyclobuta[*a*]indene]-1',3-diyl)bis(naphthalen-2-ylmethanone) (4k).

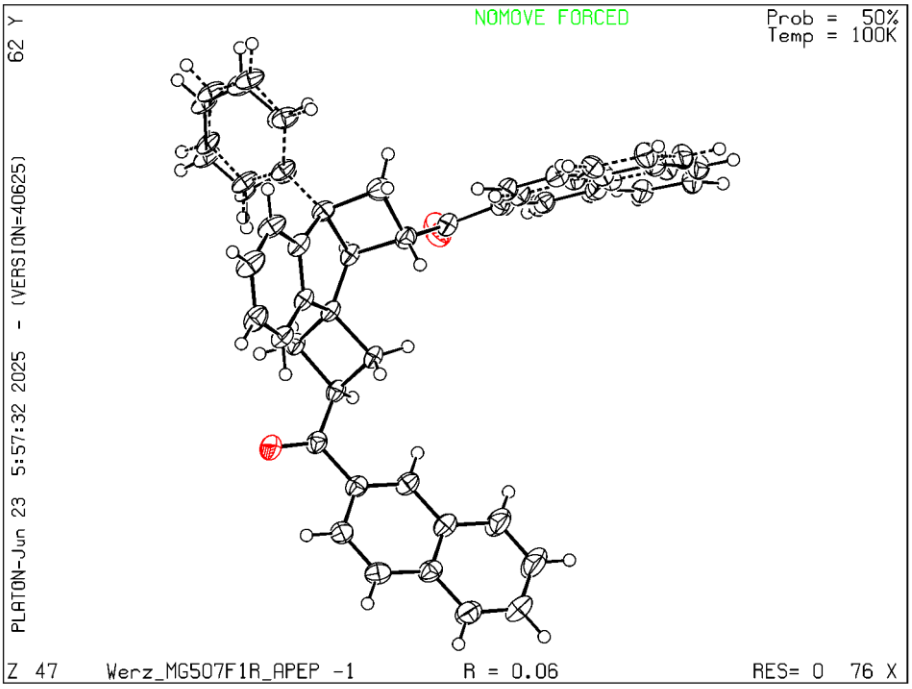

Crystals were obtained at room temperature by slow diffusion of heptane into a solution of the compound dissolved in chloroform by the aid of layering. A colourless, block-shaped crystal was mounted on a MiTeGen micromount with perfluoroether oil. Data for Werz_MG507F1R_APEX_a were collected from a shock-cooled single crystal at 100(2) K on a Bruker APEX2 QUAZAR three-circle diffractometer with a microfocus sealed X-ray tube using a mirror optics as monochromator and a Bruker APEXII detector. The diffractometer was equipped with an Oxford Cryostream 800 low temperature device and used MoK_α radiation (λ = 0.71073 Å). All data were integrated with SAINT V8.41 and a multi-scan absorption correction using SADABS 2016/2 was applied.[17;18]The structure was solved by direct methods with SHELXT and refined by full-matrix least-squares methods against F^2 using SHELXL-2019/2.[19;20] All non-hydrogen atoms were refined with anisotropic displacement parameters. All hydrogen atoms were refined isotropic on calculated positions using a riding model with their U_{iso} values constrained to 1.5 times the U_{eq} of their pivot atoms for terminal sp^3 carbon atoms and 1.2 times for all other carbon atoms. Disordered moieties were refined using bond lengths restraints and displacement parameter restraints. Some parts of the disorder model were introduced by the program DSR.[23]Crystallographic

data for the structures reported in this paper have been deposited with the Cambridge Crystallographic Data Centre.[21] CCDC 246573 contain the supplementary crystallographic data for this paper. These data can be obtained free of charge from The Cambridge Crystallographic Data Centre via www.ccdc.cam.ac.uk/structures. This report and the CIF file were generated using FinalCif.[22]

Table 6. Crystal data and structure refinement for Werz_MG507F1R_APEX_a

**Compound**	**4k**
CCDC number	246573
Empirical formula	$C_{42}H_{32}O_2$
Formula weight	568.67
Temperature [K]	100(2)
Crystal system	triclinic
Space group (number)	$P\bar{1}$ (2)
a [Å]	10.793(10)
b [Å]	11.439(10)
c [Å]	14.225(13)
α [°]	67.520(19)
β [°]	69.114(15)
γ [°]	69.485(12)
Volume [Å^3]	1469(2)
Z	2
ρ_{calc} [gcm^{-3}]	1.286
μ [mm^{-1}]	0.077
F(000)	600
Crystal size [mm^3]	0.168×0.174×0.445
Crystal colour	colourless
Crystal shape	block
Radiation	MoK_α (λ=0.71073 Å)
2θ range [°]	3.21 to 53.19 (0.79 Å)
Index ranges	−13 ≤ h ≤ 13<br>−14 ≤ k ≤ 14<br>−17 ≤ l ≤ 17

Reflections collected	28054
Independent reflections	6085<br>R_{int} = 0.0708<br>R_{sigma} = 0.0616
Completeness to θ = 25.242°	99.9 %
Data / Restraints / Parameters	6085 / 2582 / 543
Absorption correction T_{min}/T_{max} (method)	0.5417 / 0.7454 (multi-scan)
Goodness-of-fit on F^2	1.032
Final *R* indexes [$I \geq 2\sigma(I)$]	R_1 = 0.0584<br>wR_2 = 0.1367
Final *R* indexes [all data]	R_1 = 0.0970<br>wR_2 = 0.1625
Largest peak/hole [eÅ^{-3}]	0.29/−0.32

Table 7. Atomic coordinates and U_{eq} [Å^2] for Werz_MG507F1R_APEX_a

Atom	*x*	*y*	*z*	U_{eq}
O1	−0.0216(2)	0.6023(2)	0.67393(17)	0.0591(6)
O2	0.4387(2)	0.63455(17)	0.16038(15)	0.0462(5)
C1	0.2875(2)	0.49334(19)	0.44466(18)	0.0254(5)
C4	0.6347(3)	0.2496(2)	0.3810(2)	0.0377(6)
H4	0.720814	0.243083	0.330410	0.045
C5	0.6082(3)	0.1410(2)	0.4649(2)	0.0417(7)
H5	0.676205	0.060652	0.471178	0.050
C6	0.4833(2)	0.1484(2)	0.5399(2)	0.0373(6)
H6	0.464832	0.073632	0.596838	0.045
C7	0.3850(2)	0.2679(2)	0.5300(2)	0.0292(5)
C8	0.2421(2)	0.2957(2)	0.60120(19)	0.0274(5)
C9	0.2377(3)	0.3207(2)	0.7021(2)	0.0370(6)
H9A	0.326363	0.284918	0.720984	0.044
H9B	0.162732	0.292995	0.763347	0.044
C10	0.2064(3)	0.4704(2)	0.64613(19)	0.0321(5)
H10	0.289204	0.504162	0.624814	0.039
C11	0.1883(2)	0.44698(19)	0.55219(18)	0.0257(5)
H11	0.090760	0.473325	0.549237	0.031
C12	0.2339(2)	0.5460(2)	0.34357(18)	0.0273(5)
H12A	0.287303	0.496578	0.291880	0.033
H12B	0.134755	0.553941	0.359029	0.033

C13	0.2687(2)	0.6775(2)	0.31359(18)	0.0271(5)
H13	0.185154	0.751965	0.313338	0.032
C14	0.3142(2)	0.6300(2)	0.41780(19)	0.0280(5)
H14A	0.253797	0.678795	0.468823	0.034
H14B	0.411362	0.626590	0.406087	0.034
C15	0.0832(3)	0.5530(2)	0.7037(2)	0.0420(7)
C16	0.3829(2)	0.7071(2)	0.21584(19)	0.0298(5)
C2	0.4109(2)	0.3769(2)	0.44562(19)	0.0264(5)
C3	0.5362(2)	0.3682(2)	0.3704(2)	0.0311(5)
H3	0.554298	0.442230	0.312408	0.037
C1_1	0.169(2)	0.1952(19)	0.6181(18)	0.029(2)
C2_1	0.102(2)	0.2062(15)	0.5472(15)	0.031(2)
H2_1	0.101430	0.278719	0.485464	0.038
C3_1	0.0340(16)	0.1120(12)	0.5651(11)	0.029(2)
H3_1	−0.011679	0.120098	0.516083	0.035
C4_1	0.0349(15)	0.0071(12)	0.6555(10)	0.033(2)
H4_1	−0.010191	−0.057582	0.668609	0.040
C5_1	0.1012(16)	−0.0042(14)	0.7271(12)	0.036(2)
H5_1	0.099729	−0.075725	0.789600	0.043
C6_1	0.169(2)	0.0879(17)	0.7083(16)	0.034(2)
H6_1	0.216648	0.078175	0.756883	0.040
C1_2	0.158(2)	0.2069(17)	0.6143(17)	0.030(2)
C2_2	0.101(2)	0.2269(14)	0.5333(13)	0.028(2)
H2_2	0.108769	0.301114	0.473127	0.034
C3_2	0.0342(17)	0.1391(12)	0.5403(11)	0.034(2)
H3_2	−0.003705	0.154197	0.484618	0.041
C4_2	0.0214(13)	0.0296(11)	0.6272(10)	0.032(2)
H4_2	−0.025469	−0.029370	0.631225	0.039
C5_2	0.0777(15)	0.0077(14)	0.7074(12)	0.039(2)

H5_2	0.071657	−0.067935	0.766419	0.047
C6_2	0.1436(18)	0.0963(17)	0.7019(16)	0.036(2)
H6_2	0.179579	0.081577	0.758593	0.043
C1_3	0.0621(6)	0.5936(6)	0.7957(5)	0.0270(13)
C2_3	−0.0529(5)	0.6898(4)	0.8285(4)	0.0293(11)
H2_3	−0.125254	0.726215	0.794633	0.035
C3_3	−0.0594(5)	0.7303(4)	0.9095(4)	0.0301(11)
H3_3	−0.136920	0.794813	0.931886	0.036
C4_3	0.0481(7)	0.6771(7)	0.9604(6)	0.0291(13)
C5_3	0.0456(5)	0.7207(5)	1.0424(4)	0.0340(11)
H5_3	−0.028451	0.788877	1.062844	0.041
C6_3	0.1491(6)	0.6653(7)	1.0918(5)	0.0373(13)
H6_3	0.145859	0.695573	1.146435	0.045
C7_3	0.2604(6)	0.5640(5)	1.0635(4)	0.0371(12)
H7_3	0.330943	0.525880	1.099244	0.045
C8_3	0.2664(5)	0.5208(5)	0.9839(4)	0.0317(11)
H8_3	0.341538	0.452654	0.964656	0.038
C9_3	0.1614(5)	0.5768(5)	0.9300(4)	0.0273(11)
C10_3	0.1666(6)	0.5384(5)	0.8444(4)	0.0274(11)
H10_3	0.243056	0.473835	0.820940	0.033
C1_4	0.1156(6)	0.5595(5)	0.8010(5)	0.0266(13)
C2_4	0.2280(5)	0.4822(5)	0.8459(4)	0.0282(11)
H2_4	0.291620	0.413570	0.818661	0.034
C3_4	0.2463(5)	0.5046(4)	0.9276(4)	0.0317(10)
H3_4	0.322295	0.451939	0.956006	0.038
C4_4	0.1523(5)	0.6060(5)	0.9698(4)	0.0311(11)
C5_4	0.1739(6)	0.6348(6)	1.0506(5)	0.0373(13)
H5_4	0.249283	0.583058	1.079990	0.045
C6_4	0.0846(6)	0.7382(5)	1.0860(4)	0.0380(12)
H6_4	0.099629	0.758205	1.139356	0.046
C7_4	−0.0296(5)	0.8156(5)	1.0440(4)	0.0380(12)
H7_4	−0.089750	0.887307	1.068753	0.046
C8_4	−0.0530(5)	0.7866(4)	0.9678(4)	0.0345(11)

H8_4	−0.131100	0.836915	0.941477	0.041
C9_4	0.0377(7)	0.6827(7)	0.9278(5)	0.0307(13)
C10_4	0.0205(5)	0.6554(4)	0.8450(4)	0.0272(10)
H10_4	−0.058098	0.703845	0.818867	0.033
C1_6	0.4300(2)	0.8259(2)	0.19134(18)	0.0285(5)
C2_6	0.5486(2)	0.8477(2)	0.10774(19)	0.0321(5)
H2_6	0.595453	0.787557	0.067775	0.039
C3_6	0.5956(3)	0.9548(2)	0.0846(2)	0.0358(6)
H3_6	0.675823	0.967071	0.029409	0.043
C4_6	0.5272(3)	1.0475(2)	0.1412(2)	0.0337(6)
C5_6	0.5728(3)	1.1602(2)	0.1181(2)	0.0442(7)
H5_6	0.651059	1.176074	0.061734	0.053
C6_6	0.5056(4)	1.2455(3)	0.1761(3)	0.0570(8)
H6_6	0.537287	1.320275	0.160156	0.068
C7_6	0.3899(4)	1.2234(3)	0.2591(3)	0.0650(10)
H7_6	0.344632	1.282741	0.299831	0.078
C8_6	0.3410(3)	1.1175(3)	0.2824(3)	0.0524(8)
H8_6	0.261384	1.104755	0.338167	0.063
C9_6	0.4083(3)	1.0270(2)	0.2238(2)	0.0353(6)
C10_6	0.3628(2)	0.9147(2)	0.2472(2)	0.0326(5)
H10_6	0.283603	0.900453	0.303017	0.039

U_{eq} is defined as 1/3 of the trace of the orthogonalised U_{ij} tensor.

Table 8. Anisotropic displacement parameters [Å^2] for Werz_MG507F1R_APEX_a. The anisotropic displacement factor exponent takes the form: $-2\pi^2[\, h^2(a^*)^2U_{11} + k^2(b^*)^2U_{22} + \ldots + 2hka^*b^*U_{12}\,]$

Atom	U_{11}	U_{22}	U_{33}	U_{23}	U_{13}	U_{12}
O1	0.0581(14)	0.0515(12)	0.0572(14)	−0.0299(11)	−0.0117(11)	0.0109(11)
O2	0.0601(13)	0.0334(9)	0.0442(11)	−0.0201(9)	0.0008(9)	−0.0153(9)
C1	0.0265(12)	0.0167(10)	0.0342(13)	−0.0077(9)	−0.0106(10)	−0.0036(8)

C4	0.0275(13)	0.0290(12)	0.0565(18)	−0.0181(12)	−0.0079(12)	−0.0037(10)
C5	0.0269(13)	0.0221(11)	0.069(2)	−0.0120(12)	−0.0133(13)	0.0016(10)
C6	0.0297(13)	0.0212(11)	0.0560(17)	−0.0042(11)	−0.0156(12)	−0.0038(10)
C7	0.0259(12)	0.0227(10)	0.0414(14)	−0.0090(10)	−0.0130(10)	−0.0048(9)
C8	0.0270(12)	0.0192(10)	0.0341(13)	−0.0033(9)	−0.0132(10)	−0.0030(9)
C9	0.0388(14)	0.0343(13)	0.0367(15)	−0.0021(11)	−0.0166(12)	−0.0098(11)
C10	0.0368(14)	0.0306(12)	0.0343(14)	−0.0083(10)	−0.0117(11)	−0.0134(10)
C11	0.0260(12)	0.0189(10)	0.0356(13)	−0.0083(9)	−0.0127(10)	−0.0042(9)
C12	0.0326(12)	0.0200(10)	0.0322(13)	−0.0082(9)	−0.0124(10)	−0.0048(9)
C13	0.0291(12)	0.0179(10)	0.0351(13)	−0.0094(9)	−0.0120(10)	−0.0013(9)
C14	0.0318(12)	0.0198(10)	0.0363(14)	−0.0105(9)	−0.0115(10)	−0.0055(9)
C15	0.0601(19)	0.0321(13)	0.0375(16)	−0.0144(12)	−0.0031(14)	−0.0207(13)
C16	0.0339(13)	0.0210(10)	0.0339(14)	−0.0078(10)	−0.0126(11)	−0.0025(9)
C2	0.0266(12)	0.0191(10)	0.0361(14)	−0.0087(9)	−0.0120(10)	−0.0039(9)
C3	0.0295(12)	0.0229(11)	0.0410(15)	−0.0097(10)	−0.0083(11)	−0.0070(9)
C1_1	0.020(5)	0.017(4)	0.045(4)	−0.007(3)	−0.008(3)	−0.003(4)
C2_1	0.029(4)	0.010(4)	0.050(5)	−0.002(3)	−0.015(3)	−0.001(3)
C3_1	0.026(3)	0.011(5)	0.047(6)	−0.007(4)	−0.012(4)	0.001(3)
C4_1	0.030(4)	0.010(3)	0.050(6)	0.000(4)	−0.012(4)	−0.001(3)
C5_1	0.030(5)	0.016(3)	0.048(5)	0.002(3)	−0.012(3)	−0.002(3)
C6_1	0.027(6)	0.022(3)	0.043(4)	−0.004(3)	−0.008(4)	−0.003(4)
C1_2	0.025(5)	0.014(3)	0.041(4)	−0.007(3)	−0.008(3)	0.006(3)
C2_2	0.027(3)	0.009(4)	0.044(4)	−0.008(3)	−0.008(3)	0.000(3)
C3_2	0.032(3)	0.012(4)	0.050(5)	−0.004(3)	−0.009(4)	−0.002(3)

C4_2	0.024(3)	0.013(4)	0.051(6)	−0.005(3)	−0.007(3)	−0.002(3)
C5_2	0.032(5)	0.021(3)	0.055(5)	−0.001(3)	−0.016(3)	−0.003(3)
C6_2	0.024(5)	0.026(3)	0.048(4)	−0.002(3)	−0.013(3)	−0.002(3)
C1_3	0.022(3)	0.024(3)	0.033(3)	−0.008(2)	−0.004(2)	−0.008(2)
C2_3	0.026(3)	0.027(2)	0.035(3)	−0.0093(19)	−0.012(2)	−0.0030(19)
C3_3	0.030(2)	0.025(2)	0.033(2)	−0.0085(18)	−0.0067(19)	−0.0057(19)
C4_3	0.033(3)	0.028(2)	0.026(3)	−0.006(2)	−0.006(2)	−0.011(2)
C5_3	0.037(3)	0.036(3)	0.030(3)	−0.012(2)	−0.0042(19)	−0.012(2)
C6_3	0.042(3)	0.042(3)	0.033(3)	−0.015(2)	−0.009(2)	−0.013(2)
C7_3	0.039(3)	0.042(3)	0.034(3)	−0.011(2)	−0.016(2)	−0.008(2)
C8_3	0.030(2)	0.036(2)	0.028(2)	−0.0083(19)	−0.0087(19)	−0.0067(19)
C9_3	0.027(2)	0.026(2)	0.027(3)	−0.0048(19)	−0.0048(19)	−0.0088(19)
C10_3	0.024(3)	0.023(3)	0.035(3)	−0.010(2)	−0.007(2)	−0.004(2)
C1_4	0.023(3)	0.025(3)	0.033(3)	−0.013(2)	−0.005(2)	−0.005(2)
C2_4	0.025(2)	0.025(2)	0.034(2)	−0.011(2)	−0.008(2)	−0.0031(19)
C3_4	0.029(2)	0.032(2)	0.037(3)	−0.0078(19)	−0.013(2)	−0.0077(19)
C4_4	0.033(2)	0.031(2)	0.031(3)	−0.0091(19)	−0.006(2)	−0.0115(18)
C5_4	0.039(3)	0.042(3)	0.037(3)	−0.015(2)	−0.009(2)	−0.014(2)
C6_4	0.043(3)	0.043(3)	0.036(3)	−0.016(2)	−0.008(2)	−0.017(2)
C7_4	0.041(3)	0.036(3)	0.038(3)	−0.018(2)	−0.005(2)	−0.010(2)
C8_4	0.036(2)	0.031(2)	0.035(2)	−0.0113(19)	−0.0079(19)	−0.0077(18)
C9_4	0.032(3)	0.027(2)	0.034(3)	−0.009(2)	−0.007(2)	−0.0083(19)

C10_4	0.026(2)	0.022(2)	0.032(2)	−0.0083(18)	−0.0081(19)	−0.0026(18)
C1_6	0.0305(12)	0.0200(10)	0.0328(13)	−0.0040(9)	−0.0139(10)	−0.0020(9)
C2_6	0.0358(13)	0.0256(11)	0.0318(13)	−0.0060(10)	−0.0117(11)	−0.0035(10)
C3_6	0.0349(14)	0.0318(12)	0.0365(15)	−0.0033(11)	−0.0116(11)	−0.0084(10)
C4_6	0.0384(14)	0.0258(11)	0.0371(14)	−0.0027(10)	−0.0172(11)	−0.0080(10)
C5_6	0.0535(17)	0.0382(14)	0.0456(17)	−0.0047(12)	−0.0169(14)	−0.0211(13)
C6_6	0.079(2)	0.0366(14)	0.063(2)	−0.0116(14)	−0.0144(17)	−0.0313(15)
C7_6	0.080(2)	0.0379(15)	0.079(2)	−0.0296(16)	−0.0017(19)	−0.0226(16)
C8_6	0.0563(19)	0.0316(13)	0.067(2)	−0.0230(14)	−0.0024(15)	−0.0123(13)
C9_6	0.0383(14)	0.0238(11)	0.0436(15)	−0.0088(10)	−0.0130(11)	−0.0063(10)
C10_6	0.0315(13)	0.0224(11)	0.0415(15)	−0.0084(10)	−0.0108(11)	−0.0037(9)

Table 9. Bond lengths and angles for Werz_MG507F1R_APEX_a

Atom–Atom	Length [Å]
O1–C15	1.220(4)
O2–C16	1.219(3)
C1–C2	1.518(3)
C1–C11	1.542(3)
C1–C12	1.566(3)
C1–C14	1.567(3)
C4–C5	1.386(4)
C4–C3	1.393(3)
C4–H4	0.9500
C5–C6	1.388(4)
C5–H5	0.9500
C6–C7	1.399(3)
C6–H6	0.9500
C7–C2	1.391(3)
C7–C8	1.514(3)
C8–C1_2	1.508(10)
C8–C1_1	1.514(9)
C8–C9	1.549(4)
C8–C11	1.575(3)

C9–C10	1.557(3)	C2_2–C3_2	1.387(9)
C9–H9A	0.9900	C2_2–H2_2	0.9500
C9–H9B	0.9900	C3_2–C4_2	1.390(7)
C10–C15	1.505(4)	C3_2–H3_2	0.9500
C10–C11	1.546(3)	C4_2–C5_2	1.380(8)
C10–H10	1.0000	C4_2–H4_2	0.9500
C11–H11	1.0000	C5_2–C6_2	1.395(9)
C12–C13	1.542(3)	C5_2–H5_2	0.9500
C12–H12A	0.9900	C6_2–H6_2	0.9500
C12–H12B	0.9900	C1_3–C10_3	1.369(7)
C13–C16	1.510(3)	C1_3–C2_3	1.411(7)
C13–C14	1.566(3)	C2_3–C3_3	1.369(6)
C13–H13	1.0000	C2_3–H2_3	0.9500
C14–H14A	0.9900	C3_3–C4_3	1.417(7)
C14–H14B	0.9900	C3_3–H3_3	0.9500
C15–C1_3	1.470(7)	C4_3–C9_3	1.420(7)
C15–C1_4	1.578(7)	C4_3–C5_3	1.422(7)
C16–C1_6	1.491(3)	C5_3–C6_3	1.367(7)
C2–C3	1.393(3)	C5_3–H5_3	0.9500
C3–H3	0.9500	C6_3–C7_3	1.408(8)
C1_1–C2_1	1.386(10)	C6_3–H6_3	0.9500
C1_1–C6_1	1.396(10)	C7_3–C8_3	1.373(7)
C2_1–C3_1	1.403(9)	C7_3–H7_3	0.9500
C2_1–H2_1	0.9500	C8_3–C9_3	1.420(7)
C3_1–C4_1	1.383(8)	C8_3–H8_3	0.9500
C3_1–H3_1	0.9500	C9_3–C10_3	1.422(7)
C4_1–C5_1	1.385(9)	C10_3–H10_3	0.9500
C4_1–H4_1	0.9500	C1_4–C10_4	1.388(6)
C5_1–C6_1	1.382(9)	C1_4–C2_4	1.424(7)
C5_1–H5_1	0.9500	C2_4–C3_4	1.372(6)
C6_1–H6_1	0.9500	C2_4–H2_4	0.9500
C1_2–C2_2	1.399(10)	C3_4–C4_4	1.417(6)
C1_2–C6_2	1.403(9)	C3_4–H3_4	0.9500

C4_4–C5_4	1.421(7)
C4_4–C9_4	1.422(7)
C5_4–C6_4	1.375(7)
C5_4–H5_4	0.9500
C6_4–C7_4	1.420(7)
C6_4–H6_4	0.9500
C7_4–C8_4	1.371(6)
C7_4–H7_4	0.9500
C8_4–C9_4	1.414(7)
C8_4–H8_4	0.9500
C9_4–C10_4	1.413(7)
C10_4–H10_4	0.9500
C1_6–C10_6	1.370(3)
C1_6–C2_6	1.424(3)
C2_6–C3_6	1.369(3)
C2_6–H2_6	0.9500
C3_6–C4_6	1.413(3)
C3_6–H3_6	0.9500
C4_6–C9_6	1.416(4)
C4_6–C5_6	1.421(3)
C5_6–C6_6	1.360(4)
C5_6–H5_6	0.9500
C6_6–C7_6	1.400(4)
C6_6–H6_6	0.9500
C7_6–C8_6	1.368(4)
C7_6–H7_6	0.9500
C8_6–C9_6	1.412(4)
C8_6–H8_6	0.9500
C9_6–C10_6	1.415(3)
C10_6–H10_6	0.9500

**Atom–Atom–Atom**	**Angle [°]**
C2–C1–C11	102.52(18)
C2–C1–C12	114.67(19)
C11–C1–C12	118.2(2)
C2–C1–C14	117.13(19)
C11–C1–C14	116.23(18)
C12–C1–C14	88.80(16)
C5–C4–C3	120.5(2)
C5–C4–H4	119.7
C3–C4–H4	119.7
C4–C5–C6	120.7(2)
C4–C5–H5	119.7
C6–C5–H5	119.7
C5–C6–C7	118.9(2)
C5–C6–H6	120.6
C7–C6–H6	120.6
C2–C7–C6	120.6(2)
C2–C7–C8	112.18(19)
C6–C7–C8	127.1(2)
C1_2–C8–C7	113.6(10)
C1_1–C8–C7	110.3(11)
C1_2–C8–C9	116.9(9)
C1_1–C8–C9	115.8(9)
C7–C8–C9	113.5(2)
C1_2–C8–C11	118.2(8)
C1_1–C8–C11	123.9(9)
C7–C8–C11	102.09(18)
C9–C8–C11	89.46(17)
C8–C9–C10	89.85(18)
C8–C9–H9A	113.7
C10–C9–H9A	113.7
C8–C9–H9B	113.7
C10–C9–H9B	113.7
H9A–C9–H9B	110.9

C15–C10–C11	115.9(2)	O1–C15–C10	121.1(2)
C15–C10–C9	116.2(2)	C1_3–C15–C10	131.0(3)
C11–C10–C9	90.22(18)	O1–C15–C1_4	129.5(3)
C15–C10–H10	111.0	C10–C15–C1_4	109.4(3)
C11–C10–H10	111.0	O2–C16–C1_6	120.6(2)
C9–C10–H10	111.0	O2–C16–C13	121.6(2)
C1–C11–C10	115.82(19)	C1_6–C16–C13	117.7(2)
C1–C11–C8	109.25(18)	C7–C2–C3	120.0(2)
C10–C11–C8	89.28(17)	C7–C2–C1	112.4(2)
C1–C11–H11	113.4	C3–C2–C1	127.4(2)
C10–C11–H11	113.4	C2–C3–C4	119.3(2)
C8–C11–H11	113.4	C2–C3–H3	120.4
C13–C12–C1	91.11(17)	C4–C3–H3	120.4
C13–C12–H12A	113.4	C2_1–C1_1–C6_1	119.0(9)
C1–C12–H12A	113.4		
C13–C12–H12B	113.4	C2_1–C1_1–C8	122.3(13)
C1–C12–H12B	113.4	C6_1–C1_1–C8	118.7(13)
H12A–C12–H12B	110.7	C1_1–C2_1–C3_1	121.1(9)
C16–C13–C12	116.32(19)	C1_1–C2_1–H2_1	119.5
C16–C13–C14	113.1(2)		
C12–C13–C14	89.73(16)	C3_1–C2_1–H2_1	119.5
C16–C13–H13	112.0		
C12–C13–H13	112.0	C4_1–C3_1–C2_1	118.8(9)
C14–C13–H13	112.0		
C13–C14–C1	90.17(16)	C4_1–C3_1–H3_1	120.6
C13–C14–H14A	113.6		
C1–C14–H14A	113.6	C2_1–C3_1–H3_1	120.6
C13–C14–H14B	113.6		
C1–C14–H14B	113.6	C3_1–C4_1–C5_1	120.5(8)
H14A–C14–H14B	110.9	C3_1–C4_1–H4_1	119.8
O1–C15–C1_3	107.8(3)		

C5_1–C4_1–H4_1	119.8	C5_2–C4_2–H4_2	120.5
C6_1–C5_1–C4_1	120.5(8)	C3_2–C4_2–H4_2	120.5
C6_1–C5_1–H5_1	119.8	C4_2–C5_2–C6_2	120.1(8)
C4_1–C5_1–H5_1	119.8	C4_2–C5_2–H5_2	120.0
C5_1–C6_1–C1_1	120.2(9)	C6_2–C5_2–H5_2	120.0
C5_1–C6_1–H6_1	119.9	C5_2–C6_2–C1_2	121.3(9)
C1_1–C6_1–H6_1	119.9	C5_2–C6_2–H6_2	119.3
C2_2–C1_2–C6_2	117.8(9)	C1_2–C6_2–H6_2	119.3
C2_2–C1_2–C8	119.8(12)	C10_3–C1_3–C2_3	121.2(5)
C6_2–C1_2–C8	122.2(12)		
C3_2–C2_2–C1_2	120.4(9)	C10_3–C1_3–C15	115.6(5)
		C2_3–C1_3–C15	123.1(5)
C3_2–C2_2–H2_2	119.8	C3_3–C2_3–C1_3	119.5(5)
C1_2–C2_2–H2_2	119.8	C3_3–C2_3–H2_3	120.2
C2_2–C3_2–C4_2	121.3(9)	C1_3–C2_3–H2_3	120.2
C2_2–C3_2–H3_2	119.3	C2_3–C3_3–C4_3	120.8(5)
C4_2–C3_2–H3_2	119.3	C2_3–C3_3–H3_3	119.6
C5_2–C4_2–C3_2	119.1(7)	C4_3–C3_3–H3_3	119.6

C3_3–C4_3–C9_3	119.7(5)	C8_3–C9_3–C10_3	122.4(5)
C3_3–C4_3–C5_3	121.7(5)	C4_3–C9_3–C10_3	118.2(5)
C9_3–C4_3–C5_3	118.6(5)	C1_3–C10_3–C9_3	120.5(5)
C6_3–C5_3–C4_3	120.4(5)	C1_3–C10_3–H10_3	119.8
C6_3–C5_3–H5_3	119.8	C9_3–C10_3–H10_3	119.8
C4_3–C5_3–H5_3	119.8	C10_4–C1_4–C2_4	118.4(5)
C5_3–C6_3–C7_3	121.3(6)	C10_4–C1_4–C15	113.3(5)
C5_3–C6_3–H6_3	119.4	C2_4–C1_4–C15	128.3(4)
C7_3–C6_3–H6_3	119.4	C3_4–C2_4–C1_4	121.4(5)
C8_3–C7_3–C6_3	119.6(5)	C3_4–C2_4–H2_4	119.3
C8_3–C7_3–H7_3	120.2	C1_4–C2_4–H2_4	119.3
C6_3–C7_3–H7_3	120.2	C2_4–C3_4–C4_4	120.2(5)
C7_3–C8_3–C9_3	120.7(5)	C2_4–C3_4–H3_4	119.9
C7_3–C8_3–H8_3	119.6	C4_4–C3_4–H3_4	119.9
C9_3–C8_3–H8_3	119.6	C3_4–C4_4–C5_4	120.8(5)
C8_3–C9_3–C4_3	119.4(5)	C3_4–C4_4–C9_4	119.3(5)
		C5_4–C4_4–C9_4	119.8(5)

C6_4–C5_4–C4_4	119.4(5)	C1_4–C10_4–H10_4	119.2
C6_4–C5_4–H5_4	120.3	C9_4–C10_4–H10_4	119.2
C4_4–C5_4–H5_4	120.3	C10_6–C1_6–C2_6	118.9(2)
C5_4–C6_4–C7_4	121.2(5)	C10_6–C1_6–C16	122.0(2)
C5_4–C6_4–H6_4	119.4	C2_6–C1_6–C16	119.1(2)
C7_4–C6_4–H6_4	119.4	C3_6–C2_6–C1_6	120.4(2)
C8_4–C7_4–C6_4	119.8(4)	C3_6–C2_6–H2_6	119.8
C8_4–C7_4–H7_4	120.1	C1_6–C2_6–H2_6	119.8
C6_4–C7_4–H7_4	120.1	C2_6–C3_6–C4_6	121.4(2)
C7_4–C8_4–C9_4	120.8(5)	C2_6–C3_6–H3_6	119.3
C7_4–C8_4–H8_4	119.6	C4_6–C3_6–H3_6	119.3
C9_4–C8_4–H8_4	119.6	C3_6–C4_6–C9_6	118.5(2)
C10_4–C9_4–C8_4	122.0(5)	C3_6–C4_6–C5_6	122.5(2)
C10_4–C9_4–C4_4	119.0(5)	C9_6–C4_6–C5_6	118.9(2)
C8_4–C9_4–C4_4	119.0(5)	C6_6–C5_6–C4_6	120.6(3)
C1_4–C10_4–C9_4	121.5(5)	C6_6–C5_6–H5_6	119.7
		C4_6–C5_6–H5_6	119.7

C5_6–C6_6–C7_6	120.2(3)	C9_6–C8_6–H8_6	119.9
C5_6–C6_6–H6_6	119.9	C8_6–C9_6–C10_6	121.9(2)
C7_6–C6_6–H6_6	119.9	C8_6–C9_6–C4_6	119.0(2)
C8_6–C7_6–C6_6	120.9(3)	C10_6–C9_6–C4_6	119.1(2)
C8_6–C7_6–H7_6	119.5	C1_6–C10_6–C9_6	121.7(2)
C6_6–C7_6–H7_6	119.5	C1_6–C10_6–H10_6	119.2
C7_6–C8_6–C9_6	120.3(3)	C9_6–C10_6–H10_6	119.2
C7_6–C8_6–H8_6	119.9		

Table 10. Torsion angles for Werz_MG507F1R_APEX_a

Atom–Atom–Atom–Atom	Torsion Angle [°]	Atom–Atom–Atom–Atom	Torsion Angle [°]
C3–C4–C5–C6	−0.1(4)	C1_2–C8–C9–C10	−129.8(10)
C4–C5–C6–C7	−0.8(4)	C1_1–C8–C9–C10	−136.1(11)
C5–C6–C7–C2	1.1(4)	C7–C8–C9–C10	94.8(2)
C5–C6–C7–C8	178.3(2)	C11–C8–C9–C10	−8.16(18)
C2–C7–C8–C1_2	122.7(8)	C8–C9–C10–C15	127.6(2)
C6–C7–C8–C1_2	−54.7(9)	C8–C9–C10–C11	8.32(18)
C2–C7–C8–C1_1	127.8(8)	C2–C1–C11–C10	86.3(2)
C6–C7–C8–C1_1	−49.6(9)	C12–C1–C11–C10	−146.56(19)
C2–C7–C8–C9	−100.4(2)	C14–C1–C11–C10	−42.8(3)
C6–C7–C8–C9	82.2(3)	C2–C1–C11–C8	−12.6(2)
C2–C7–C8–C11	−5.7(2)	C12–C1–C11–C8	114.6(2)
C6–C7–C8–C11	176.9(2)	C14–C1–C11–C8	−141.67(19)
		C15–C10–C11–C1	121.2(2)
		C9–C10–C11–C1	−119.3(2)

C15–C10–C11–C8	−127.7(2)
C9–C10–C11–C8	−8.18(18)
C1_2–C8–C11–C1	−114.0(11)
C1_1–C8–C11–C1	−113.4(12)
C7–C8–C11–C1	11.4(2)
C9–C8–C11–C1	125.4(2)
C1_2–C8–C11–C10	128.8(11)
C1_1–C8–C11–C10	129.4(12)
C7–C8–C11–C10	−105.8(2)
C9–C8–C11–C10	8.22(18)
C2–C1–C12–C13	−116.2(2)
C11–C1–C12–C13	122.68(19)
C14–C1–C12–C13	3.31(17)
C1–C12–C13–C16	112.5(2)
C1–C12–C13–C14	−3.32(17)
C16–C13–C14–C1	−115.37(19)
C12–C13–C14–C1	3.31(17)
C2–C1–C14–C13	114.0(2)
C11–C1–C14–C13	−124.4(2)
C12–C1–C14–C13	−3.26(17)
C11–C10–C15–O1	−3.1(3)
C9–C10–C15–O1	−107.2(3)
C11–C10–C15–C1_3	179.1(4)
C9–C10–C15–C1_3	75.0(4)
C11–C10–C15–C1_4	176.3(3)
C9–C10–C15–C1_4	72.2(3)
C12–C13–C16–O2	4.3(3)
C14–C13–C16–O2	106.1(3)
C12–C13–C16–C1_6	−173.18(19)
C14–C13–C16–C1_6	−71.3(2)
C6–C7–C2–C3	−0.6(4)
C8–C7–C2–C3	−178.2(2)
C6–C7–C2–C1	175.3(2)
C8–C7–C2–C1	−2.3(3)
C11–C1–C2–C7	9.3(2)
C12–C1–C2–C7	−120.1(2)
C14–C1–C2–C7	137.8(2)
C11–C1–C2–C3	−175.1(2)
C12–C1–C2–C3	55.5(3)
C14–C1–C2–C3	−46.6(3)
C7–C2–C3–C4	−0.3(4)
C1–C2–C3–C4	−175.5(2)
C5–C4–C3–C2	0.7(4)
C7–C8–C1_1–C2_1	−83.8(15)
C9–C8–C1_1–C2_1	145.6(12)
C11–C8–C1_1–C2_1	37(2)
C7–C8–C1_1–C6_1	96.3(17)
C9–C8–C1_1–C6_1	−34(2)
C11–C8–C1_1–C6_1	−142.5(14)
C6_1–C1_1–C2_1–C3_1	0.3(15)
C8–C1_1–C2_1–C3_1	−180(2)
C1_1–C2_1–C3_1–C4_1	0.2(12)
C2_1–C3_1–C4_1–C5_1	0.2(17)
C3_1–C4_1–C5_1–C6_1	−1.2(19)

C4_1–C5_1–C6_1–C1_1	2(2)
C2_1–C1_1–C6_1–C5_1	−1(2)
C8–C1_1–C6_1–C5_1	178.7(18)
C7–C8–C1_2–C2_2	−76.1(14)
C9–C8–C1_2–C2_2	148.6(10)
C11–C8–C1_2–C2_2	43.5(17)
C7–C8–C1_2–C6_2	98.1(18)
C9–C8–C1_2–C6_2	−37(2)
C11–C8–C1_2–C6_2	−142.4(15)
C6_2–C1_2–C2_2–C3_2	−0.3(14)
C8–C1_2–C2_2–C3_2	174.1(19)
C1_2–C2_2–C3_2–C4_2	0.0(12)
C2_2–C3_2–C4_2–C5_2	−0.6(16)
C3_2–C4_2–C5_2–C6_2	1.5(18)
C4_2–C5_2–C6_2–C1_2	−2(2)
C2_2–C1_2–C6_2–C5_2	1(2)
C8–C1_2–C6_2–C5_2	−173.0(18)
O1–C15–C1_3–C10_3	175.0(4)
C10–C15–C1_3–C10_3	−6.9(7)
O1–C15–C1_3–C2_3	−9.2(6)
C10–C15–C1_3–C2_3	168.8(3)
C10_3–C1_3–C2_3–C3_3	1.1(6)
C15–C1_3–C2_3–C3_3	−174.4(5)
C1_3–C2_3–C3_3–C4_3	0.2(6)
C2_3–C3_3–C4_3–C9_3	−2.6(10)
C2_3–C3_3–C4_3–C5_3	177.9(6)
C3_3–C4_3–C5_3–C6_3	178.3(6)
C9_3–C4_3–C5_3–C6_3	−1.2(10)
C4_3–C5_3–C6_3–C7_3	0.0(9)
C5_3–C6_3–C7_3–C8_3	0.7(9)
C6_3–C7_3–C8_3–C9_3	−0.1(8)
C7_3–C8_3–C9_3–C4_3	−1.1(8)
C7_3–C8_3–C9_3–C10_3	177.5(5)
C3_3–C4_3–C9_3–C8_3	−177.8(6)

C5_3–C4_3–C9_3–C8_3	1.8(10)
C3_3–C4_3–C9_3–C10_3	3.6(10)
C5_3–C4_3–C9_3–C10_3	−176.9(6)
C2_3–C1_3–C10_3–C9_3	0.1(8)
C15–C1_3–C10_3–C9_3	175.9(4)
C8_3–C9_3–C10_3–C1_3	179.1(5)
C4_3–C9_3–C10_3–C1_3	−2.4(9)
O1–C15–C1_4–C10_4	−13.9(6)
C10–C15–C1_4–C10_4	166.7(4)
O1–C15–C1_4–C2_4	166.9(4)
C10–C15–C1_4–C2_4	−12.4(6)
C10_4–C1_4–C2_4–C3_4	−2.8(6)
C15–C1_4–C2_4–C3_4	176.3(4)
C1_4–C2_4–C3_4–C4_4	0.3(5)
C2_4–C3_4–C4_4–C5_4	−176.7(4)
C2_4–C3_4–C4_4–C9_4	1.1(8)

C3_4–C4_4–C5_4–C6_4	176.6(5)
C9_4–C4_4–C5_4–C6_4	−1.1(9)
C4_4–C5_4–C6_4–C7_4	0.8(8)
C5_4–C6_4–C7_4–C8_4	0.7(8)
C6_4–C7_4–C8_4–C9_4	−1.9(8)
C7_4–C8_4–C9_4–C10_4	−176.3(6)
C7_4–C8_4–C9_4–C4_4	1.6(10)
C3_4–C4_4–C9_4–C10_4	0.1(10)
C5_4–C4_4–C9_4–C10_4	177.9(6)
C3_4–C4_4–C9_4–C8_4	−177.8(6)
C5_4–C4_4–C9_4–C8_4	0.0(10)
C2_4–C1_4–C10_4–C9_4	4.1(8)
C15–C1_4–C10_4–C9_4	−175.2(5)
C8_4–C9_4–C10_4–C1_4	175.1(6)
C4_4–C9_4–C10_4–C1_4	−2.8(10)
O2–C16–C1_6–C10_6	174.8(2)

C13–C16–C1_6–C10_6	−7.7(3)	C6_6–C7_6–C8_6–C9_6	1.1(6)
O2–C16–C1_6–C2_6	−5.5(3)	C7_6–C8_6–C9_6–C10_6	178.4(3)
C13–C16–C1_6–C2_6	172.02(19)	C7_6–C8_6–C9_6–C4_6	0.3(5)
C10_6–C1_6–C2_6–C3_6	0.8(3)	C3_6–C4_6–C9_6–C8_6	178.6(3)
C16–C1_6–C2_6–C3_6	−178.9(2)	C5_6–C4_6–C9_6–C8_6	−1.6(4)
C1_6–C2_6–C3_6–C4_6	−1.1(3)	C3_6–C4_6–C9_6–C10_6	0.4(4)
C2_6–C3_6–C4_6–C9_6	0.5(4)	C5_6–C4_6–C9_6–C10_6	−179.8(2)
C2_6–C3_6–C4_6–C5_6	−179.3(2)	C2_6–C1_6–C10_6–C9_6	0.0(4)
C3_6–C4_6–C5_6–C6_6	−178.6(3)	C16–C1_6–C10_6–C9_6	179.7(2)
C9_6–C4_6–C5_6–C6_6	1.6(4)	C8_6–C9_6–C10_6–C1_6	−178.8(3)
C4_6–C5_6–C6_6–C7_6	−0.3(5)	C4_6–C9_6–C10_6–C1_6	−0.6(4)
C5_6–C6_6–C7_6–C8_6	−1.1(6)		

9.3 Dimethyl 1'-phenyl-1',2'-dihydro-3'H-spiro[cyclobutane-1,4'-[1,3]methanonaphthalene]-3,3'-dicarboxylate (6a).

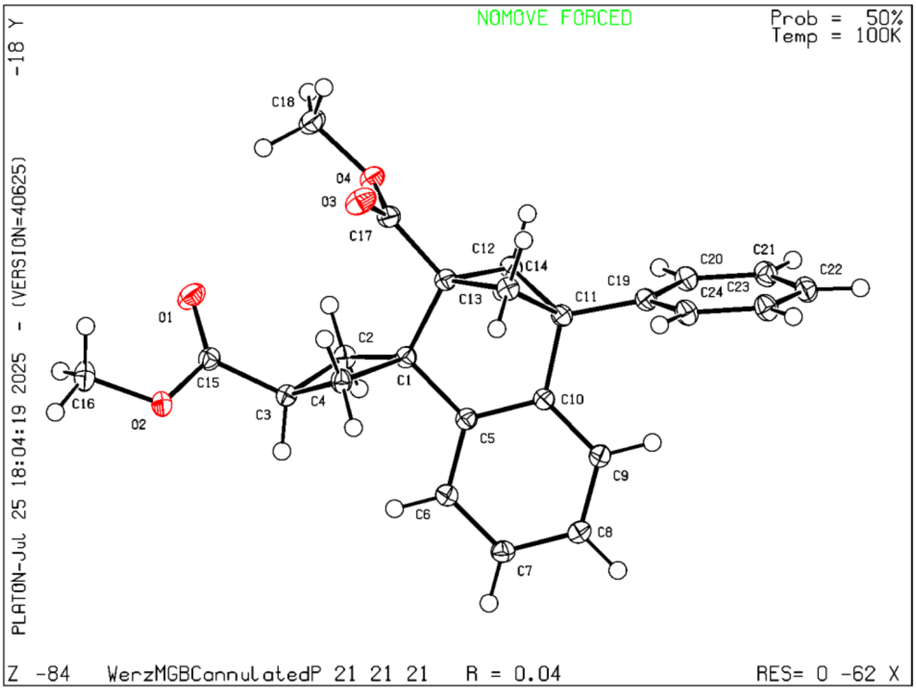

Crystals were obtained at room temperature by gas-phase diffusion of *n*-heptane into a solution of the compound dissolved in chloroform. A colourless, block-shaped crystal was mounted on a MiTeGen micromount with perfluoroether oil. Data for WerzMGBCannulated_a were collected from a shock-cooled single crystal at 100(2) K on a Bruker D8 VENTURE dual wavelength Mo/Cu three-circle diffractometer with a microfocus sealed X-ray tube using a mirror optics as monochromator and a Bruker PHOTON III detector. The diffractometer was equipped with an Oxford Cryostream 800 low temperature device and used MoK_α radiation (λ = 0.71073 Å). All data were integrated with SAINT V8.41 and a multi-scan absorption correction using SADABS 2016/2 was applied.[17;18]The structure was solved by direct methods with SHELXT and refined by full-matrix least-squares methods against F^2 using SHELXL-2019/2.[19;20] All non-hydrogen atoms were refined with anisotropic displacement parameters. All hydrogen atoms were refined isotropic on calculated positions using a riding model with their U_{iso} values constrained to 1.5 times the U_{eq} of their pivot atoms for terminal sp^3 carbon atoms and 1.2 times for all other carbon atoms. Crystallographic data for the structures reported in this paper have been deposited

with the Cambridge Crystallographic Data Centre.[21] CCDC 2476077 contain the supplementary crystallographic data for this paper. These data can be obtained free of charge from The Cambridge Crystallographic Data Centre via www.ccdc.cam.ac.uk/ structures. This report and the CIF file were generated using FinalCif.[22]

Table 11. Crystal data and structure refinement for WerzMGBCannulated_a

**Compound**	**6a**
CCDC number	2476077
Empirical formula	$C_{24}H_{24}O_4$
Formula weight	376.43
Temperature [K]	100(2)
Crystal system	orthorhombic
Space group (number)	$P2_12_12_1$ (19)
a [Å]	8.3477(18)
b [Å]	14.523(3)
c [Å]	15.319(3)
α [°]	90
β [°]	90
γ [°]	90
Volume [Å^3]	1857.2(7)
Z	4
ρ_{calc} [gcm^{-3}]	1.346
μ [mm^{-1}]	0.091
F(000)	800
Crystal size [mm^3]	0.116×0.237×0.295
Crystal colour	colourless
Crystal shape	block
Radiation	MoK_α (λ=0.71073 Å)
2θ range [°]	3.86 to 80.69 (0.55 Å)
Index ranges	−15≤ h ≤ 14<br>−26 ≤ k ≤ 25<br>−27 ≤ l ≤ 27
Reflections collected	193462

Independent reflections	11588 R_{int} = 0.0787 R_{sigma} = 0.0358
Completeness to θ = 25.242°	99.9 %
Data / Restraints / Parameters	11588 / 0 / 255
Absorption correction T_{min}/T_{max} (method)	0.6659 / 0.7479 (multi-scan)
Goodness-of-fit on F^2	1.056
Final R indexes [$I \geq 2\sigma(I)$]	R_1 = 0.0398 wR_2 = 0.0957
Final R indexes [all data]	R_1 = 0.0496 wR_2 = 0.1012
Largest peak/hole [eÅ^{-3}]	0.34/−0.23
Flack X parameter	-0.1(2)

Table 12. Atomic coordinates and U_{eq} [Å^2] for WerzMGBCannulated_a

Atom	*x*	*y*	*z*	U_{eq}
O1	0.29313(10)	0.85192(6)	−0.01286(6)	0.02431(15)
O2	0.03243(10)	0.89019(5)	−0.01753(5)	0.01850(13)
O3	0.43829(11)	0.91818(5)	0.22653(6)	0.02362(16)
O4	0.56895(9)	0.79379(5)	0.17518(5)	0.01795(13)
C1	0.19744(11)	0.74218(6)	0.21586(6)	0.01384(13)
C2	0.22830(11)	0.70884(6)	0.12002(6)	0.01578(14)
H2A	0.338881	0.720371	0.099381	0.019
H2B	0.196981	0.643998	0.109691	0.019
C3	0.10528(11)	0.77921(6)	0.08573(6)	0.01488(14)
H3	0.001013	0.748801	0.071513	0.018
C4	0.10021(12)	0.82494(6)	0.17784(6)	0.01542(14)
H4A	−0.009162	0.830568	0.202244	0.019
H4B	0.157753	0.884459	0.180926	0.019
C5	0.09279(11)	0.67732(6)	0.26895(6)	0.01445(14)
C6	−0.05376(12)	0.64532(7)	0.23720(6)	0.01652(14)
H6	−0.087319	0.661900	0.180066	0.020
C7	−0.15142(12)	0.58925(7)	0.28854(7)	0.01753(15)
H7	−0.250945	0.567819	0.266379	0.021
C8	−0.10292(12)	0.56483(7)	0.37209(7)	0.01776(15)
H8	−0.170289	0.527815	0.407685	0.021
C9	0.04472(12)	0.59470(6)	0.40363(6)	0.01690(15)
H9	0.078780	0.576647	0.460268	0.020
C10	0.14292(11)	0.65083(6)	0.35282(6)	0.01419(13)
C11	0.30348(11)	0.68792(6)	0.38378(6)	0.01436(13)
C12	0.42445(11)	0.67773(6)	0.30670(6)	0.01528(14)
H12A	0.538256	0.684077	0.323849	0.018
H12B	0.407202	0.622430	0.270100	0.018
C13	0.34847(11)	0.76717(6)	0.26952(6)	0.01444(14)
C14	0.30247(12)	0.79364(6)	0.36454(6)	0.01559(14)
H14A	0.195974	0.823028	0.370254	0.019

H14B	0.385873	0.828860	0.396094	0.019
C15	0.15726(12)	0.84239(6)	0.01359(6)	0.01571(14)
C16	0.06612(16)	0.95130(8)	−0.08956(7)	0.02366(19)
H16A	0.103897	0.915341	−0.139624	0.035
H16B	0.148993	0.995475	−0.072258	0.035
H16C	−0.031645	0.984557	−0.105677	0.035
C17	0.45400(11)	0.83523(6)	0.22252(6)	0.01626(14)
C18	0.66341(13)	0.85516(8)	0.12139(8)	0.02195(18)
H18A	0.752179	0.820849	0.095041	0.033
H18B	0.706227	0.905135	0.157503	0.033
H18C	0.595789	0.881058	0.075221	0.033
C19	0.34898(11)	0.65349(6)	0.47256(6)	0.01507(14)
C20	0.44159(12)	0.57376(6)	0.48130(6)	0.01702(15)
H20	0.488053	0.546281	0.431046	0.020
C21	0.46628(13)	0.53427(7)	0.56315(7)	0.01893(16)
H21	0.528640	0.479824	0.568327	0.023
C22	0.39982(13)	0.57441(7)	0.63733(7)	0.01942(16)
H22	0.413852	0.546439	0.692865	0.023
C23	0.31262(13)	0.65582(7)	0.62966(6)	0.01964(16)
H23	0.270168	0.684577	0.680326	0.024
C24	0.28748(13)	0.69523(7)	0.54763(6)	0.01788(15)
H24	0.228144	0.750819	0.542823	0.021

U_{eq} is defined as 1/3 of the trace of the orthogonalised U_{ij} tensor.

Table 13. Anisotropic displacement parameters [Å^2] for WerzMGBCannulated_a. The anisotropic displacement factor exponent takes the form: $-2\pi^2[h^2(a^*)^2U_{11} + k^2(b^*)^2U_{22} + \ldots + 2hka^*b^*U_{12}]$

Atom	U_{11}	U_{22}	U_{33}	U_{23}	U_{13}	U_{12}
O1	0.0194(3)	0.0252(3)	0.0283(4)	0.0068(3)	0.0074(3)	0.0008(3)
O2	0.0191(3)	0.0209(3)	0.0155(3)	0.0042(2)	−0.0012(2)	0.0007(2)
O3	0.0234(4)	0.0141(3)	0.0333(4)	0.0008(3)	0.0064(3)	−0.0001(3)
O4	0.0163(3)	0.0172(3)	0.0204(3)	0.0018(2)	0.0032(2)	−0.0002(2)
C1	0.0140(3)	0.0132(3)	0.0144(3)	0.0005(2)	0.0006(3)	0.0008(2)

C2	0.0170(3)	0.0153(3)	0.0150(3)	−0.0008(3)	0.0005(3)	0.0015(3)
C3	0.0149(3)	0.0161(3)	0.0136(3)	0.0011(3)	0.0007(3)	−0.0004(3)
C4	0.0170(4)	0.0150(3)	0.0143(3)	0.0007(3)	0.0005(3)	0.0025(3)
C5	0.0146(3)	0.0138(3)	0.0150(3)	0.0002(3)	0.0004(3)	0.0002(3)
C6	0.0158(3)	0.0174(3)	0.0163(3)	0.0008(3)	−0.0016(3)	−0.0013(3)
C7	0.0162(4)	0.0172(3)	0.0192(4)	0.0004(3)	−0.0011(3)	−0.0021(3)
C8	0.0175(4)	0.0170(3)	0.0187(4)	0.0018(3)	0.0005(3)	−0.0030(3)
C9	0.0178(4)	0.0172(3)	0.0157(3)	0.0019(3)	−0.0006(3)	−0.0021(3)
C10	0.0144(3)	0.0138(3)	0.0144(3)	−0.0001(3)	−0.0005(3)	0.0001(3)
C11	0.0143(3)	0.0142(3)	0.0145(3)	−0.0002(3)	−0.0007(3)	0.0010(3)
C12	0.0149(3)	0.0145(3)	0.0165(3)	0.0002(3)	0.0005(3)	0.0019(3)
C13	0.0143(3)	0.0134(3)	0.0156(3)	0.0003(3)	0.0006(3)	0.0003(3)
C14	0.0170(3)	0.0138(3)	0.0160(3)	−0.0010(3)	−0.0004(3)	0.0005(3)
C15	0.0167(3)	0.0161(3)	0.0143(3)	−0.0001(3)	0.0009(3)	−0.0004(3)
C16	0.0307(5)	0.0232(4)	0.0171(4)	0.0056(3)	−0.0009(4)	0.0002(4)
C17	0.0148(3)	0.0153(3)	0.0187(4)	0.0010(3)	0.0000(3)	−0.0003(3)
C18	0.0186(4)	0.0227(4)	0.0246(4)	0.0042(4)	0.0046(3)	−0.0011(3)
C19	0.0155(3)	0.0149(3)	0.0148(3)	−0.0008(3)	−0.0012(3)	0.0004(3)
C20	0.0178(4)	0.0161(3)	0.0172(3)	−0.0005(3)	−0.0006(3)	0.0016(3)
C21	0.0202(4)	0.0166(3)	0.0200(4)	0.0014(3)	−0.0029(3)	0.0013(3)
C22	0.0218(4)	0.0197(4)	0.0167(4)	0.0010(3)	−0.0039(3)	−0.0008(3)
C23	0.0218(4)	0.0218(4)	0.0154(4)	−0.0018(3)	−0.0015(3)	0.0019(3)
C24	0.0195(4)	0.0185(4)	0.0157(3)	−0.0018(3)	−0.0013(3)	0.0027(3)

Table 14. Bond lengths and angles for WerzMGBCannulated_a

Atom–Atom	Length [Å]
O1–C15	1.2123(13)
O2–C15	1.3399(12)
O2–C16	1.4436(13)
O3–C17	1.2133(12)
O4–C17	1.3449(12)
O4–C18	1.4475(13)
C1–C5	1.5205(13)
C1–C13	1.5482(13)
C1–C4	1.5629(13)
C1–C2	1.5673(13)
C2–C3	1.5412(13)
C2–H2A	0.9900
C2–H2B	0.9900
C3–C15	1.5004(13)
C3–C4	1.5601(13)

C3–H3	1.0000
C4–H4A	0.9900
C4–H4B	0.9900
C5–C6	1.3961(13)
C5–C10	1.4050(13)
C6–C7	1.3951(14)
C6–H6	0.9500
C7–C8	1.3885(14)
C7–H7	0.9500
C8–C9	1.3930(14)
C8–H8	0.9500
C9–C10	1.3936(13)
C9–H9	0.9500
C10–C11	1.5203(13)
C11–C19	1.4980(13)
C11–C12	1.5608(13)
C11–C14	1.5634(13)
C12–C13	1.5536(13)
C12–H12A	0.9900
C12–H12B	0.9900
C13–C17	1.5071(13)
C13–C14	1.5537(13)
C14–H14A	0.9900
C14–H14B	0.9900
C16–H16A	0.9800
C16–H16B	0.9800
C16–H16C	0.9800
C18–H18A	0.9800
C18–H18B	0.9800
C18–H18C	0.9800
C19–C24	1.3978(13)
C19–C20	1.3986(13)
C20–C21	1.3940(14)
C20–H20	0.9500
C21–C22	1.3925(15)
C21–H21	0.9500
C22–C23	1.3934(15)
C22–H22	0.9500
C23–C24	1.3967(14)
C23–H23	0.9500
C24–H24	0.9500

**Atom–Atom–Atom**	**Angle [°]**
C15–O2–C16	116.04(9)
C17–O4–C18	114.84(8)
C5–C1–C13	109.22(7)
C5–C1–C4	112.17(8)
C13–C1–C4	116.14(7)
C5–C1–C2	113.83(7)
C13–C1–C2	115.84(8)
C4–C1–C2	88.50(7)
C3–C2–C1	90.28(7)
C3–C2–H2A	113.6
C1–C2–H2A	113.6
C3–C2–H2B	113.6
C1–C2–H2B	113.6
H2A–C2–H2B	110.9
C15–C3–C2	117.64(8)
C15–C3–C4	114.44(8)
C2–C3–C4	89.54(7)
C15–C3–H3	111.2
C2–C3–H3	111.2
C4–C3–H3	111.2
C3–C4–C1	89.75(7)
C3–C4–H4A	113.7

C1–C4–H4A	113.7
C3–C4–H4B	113.7
C1–C4–H4B	113.7
H4A–C4–H4B	110.9
C6–C5–C10	119.24(8)
C6–C5–C1	121.56(8)
C10–C5–C1	119.18(8)
C7–C6–C5	120.65(9)
C7–C6–H6	119.7
C5–C6–H6	119.7
C8–C7–C6	119.90(9)
C8–C7–H7	120.1
C6–C7–H7	120.1
C7–C8–C9	119.87(9)
C7–C8–H8	120.1
C9–C8–H8	120.1
C8–C9–C10	120.59(9)
C8–C9–H9	119.7
C10–C9–H9	119.7
C9–C10–C5	119.72(9)
C9–C10–C11	123.47(8)
C5–C10–C11	116.80(8)
C19–C11–C10	112.86(8)
C19–C11–C12	119.42(8)
C10–C11–C12	107.51(8)
C19–C11–C14	120.03(8)
C10–C11–C14	106.52(7)
C12–C11–C14	87.36(7)
C13–C12–C11	86.22(7)
C13–C12–H12A	114.3
C11–C12–H12A	114.3
C13–C12–H12B	114.3
C11–C12–H12B	114.3
H12A–C12–H12B	111.4
C17–C13–C1	112.09(8)
C17–C13–C12	119.00(8)
C1–C13–C12	109.34(7)
C17–C13–C14	115.45(8)
C1–C13–C14	110.74(8)
C12–C13–C14	87.95(7)
C13–C14–C11	86.12(7)
C13–C14–H14A	114.3
C11–C14–H14A	114.3
C13–C14–H14B	114.3
C11–C14–H14B	114.3
H14A–C14–H14B	111.4
O1–C15–O2	123.33(9)
O1–C15–C3	125.91(9)
O2–C15–C3	110.73(8)
O2–C16–H16A	109.5
O2–C16–H16B	109.5
H16A–C16–H16B	109.5
O2–C16–H16C	109.5
H16A–C16–H16C	109.5
H16B–C16–H16C	109.5
O3–C17–O4	123.27(9)
O3–C17–C13	124.32(9)
O4–C17–C13	112.41(8)
O4–C18–H18A	109.5
O4–C18–H18B	109.5

H18A–C18–H18B	109.5	C22–C21–H21	119.9
		C20–C21–H21	119.9
O4–C18–H18C	109.5	C21–C22–C23	119.63(9)
H18A–C18–H18C	109.5	C21–C22–H22	120.2
		C23–C22–H22	120.2
H18B–C18–H18C	109.5	C22–C23–C24	120.14(9)
		C22–C23–H23	119.9
C24–C19–C20	118.90(9)	C24–C23–H23	119.9
C24–C19–C11	120.60(8)	C23–C24–C19	120.49(9)
C20–C19–C11	120.23(8)	C23–C24–H24	119.8
C21–C20–C19	120.56(9)	C19–C24–H24	119.8
C21–C20–H20	119.7		
C19–C20–H20	119.7		
C22–C21–C20	120.19(9)		

Table 15. Torsion angles for WerzMGBCannulated_a

Atom–Atom–Atom–Atom	Torsion Angle [°]	Atom–Atom–Atom–Atom	Torsion Angle [°]
C5–C1–C2–C3	103.16(8)	C4–C1–C5–C10	−130.21(9)
C13–C1–C2–C3	−128.97(8)	C2–C1–C5–C10	131.21(9)
C4–C1–C2–C3	−10.49(7)	C10–C5–C6–C7	1.34(14)
C1–C2–C3–C15	128.07(8)	C1–C5–C6–C7	−177.03(9)
C1–C2–C3–C4	10.50(7)	C5–C6–C7–C8	0.00(15)
C15–C3–C4–C1	−130.92(8)	C6–C7–C8–C9	−1.44(15)
C2–C3–C4–C1	−10.53(7)	C7–C8–C9–C10	1.54(15)
C5–C1–C4–C3	−104.84(8)	C8–C9–C10–C5	−0.19(14)
C13–C1–C4–C3	128.58(8)	C8–C9–C10–C11	178.45(9)
C2–C1–C4–C3	10.36(7)	C6–C5–C10–C9	−1.23(13)
C13–C1–C5–C6	178.38(8)	C1–C5–C10–C9	177.17(8)
C4–C1–C5–C6	48.15(11)	C6–C5–C10–C11	−179.96(8)
C2–C1–C5–C6	−50.43(12)	C1–C5–C10–C11	−1.56(12)
C13–C1–C5–C10	0.01(11)	C9–C10–C11–C19	3.21(12)
		C5–C10–C11–C19	−178.11(8)
		C9–C10–C11–C12	136.99(9)

C5–C10–C11–C12	−44.33(10)
C9–C10–C11–C14	−130.54(9)
C5–C10–C11–C14	48.14(10)
C19–C11–C12–C13	−149.36(8)
C10–C11–C12–C13	80.43(8)
C14–C11–C12–C13	−26.06(7)
C5–C1–C13–C17	−177.15(7)
C4–C1–C13–C17	−49.11(10)
C2–C1–C13–C17	52.74(10)
C5–C1–C13–C12	48.67(9)
C4–C1–C13–C12	176.71(7)
C2–C1–C13–C12	−81.45(9)
C5–C1–C13–C14	−46.64(9)
C4–C1–C13–C14	81.41(9)
C2–C1–C13–C14	−176.75(7)
C11–C12–C13–C17	144.39(8)
C11–C12–C13–C1	−85.06(8)
C11–C12–C13–C14	26.23(7)
C17–C13–C14–C11	−147.54(8)
C1–C13–C14–C11	83.74(8)
C12–C13–C14–C11	−26.18(7)
C19–C11–C14–C13	148.83(9)
C10–C11–C14–C13	−81.42(8)
C12–C11–C14–C13	26.06(7)
C16–O2–C15–O1	3.67(14)
C16–O2–C15–C3	−177.91(8)
C2–C3–C15–O1	−10.35(15)
C4–C3–C15–O1	92.83(12)
C2–C3–C15–O2	171.28(8)
C4–C3–C15–O2	−85.54(10)
C18–O4–C17–O3	−6.27(15)
C18–O4–C17–C13	173.82(8)
C1–C13–C17–O3	86.98(12)
C12–C13–C17–O3	−143.71(10)
C14–C13–C17–O3	−41.08(14)
C1–C13–C17–O4	−93.11(9)
C12–C13–C17–O4	36.20(12)
C14–C13–C17–O4	138.83(8)
C10–C11–C19–C24	−81.93(11)
C12–C11–C19–C24	150.29(9)
C14–C11–C19–C24	44.94(13)
C10–C11–C19–C20	92.04(11)
C12–C11–C19–C20	−35.74(13)
C14–C11–C19–C20	−141.09(9)
C24–C19–C20–C21	2.80(15)
C11–C19–C20–C21	−171.27(9)
C19–C20–C21–C22	−0.53(15)
C20–C21–C22–C23	−1.97(16)
C21–C22–C23–C24	2.15(16)
C22–C23–C24–C19	0.15(16)
C20–C19–C24–C23	−2.61(15)
C11–C19–C24–C23	171.44(10)

9.4 1',4-Diphenyl-[1,1'-bi(cyclobutan)]-4-ene-2,3'-dicarboxylic acid (9).

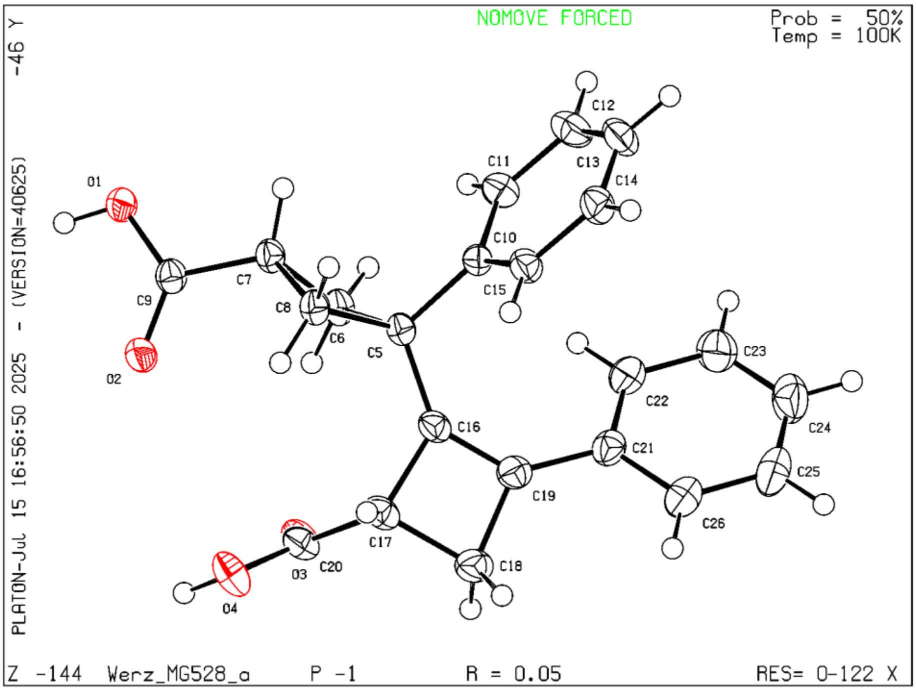

Crystals were obtained at room temperature by gas-phase diffusion of heptane into a solution of the compound dissolved in dichloromethane. A colourless, block-shaped crystal was mounted on a MiTeGen micromount with perfluoroether oil. Data for Werz_MG528_a were collected from a shock-cooled single crystal at 100(2) K on a Bruker D8 VENTURE dual wavelength Mo/Cu three-circle diffractometer with a microfocus sealed X-ray tube using a mirror optics as monochromator and a Bruker PHOTON III detector. The diffractometer was equipped with an Oxford Cryostream 800 low temperature device and used MoK_α radiation (λ = 0.71073 Å). All data were integrated with SAINT V8.41 and a multi-scan absorption correction using TWINABS Bruker was applied.[17;18] The structure was solved by direct methods with SHELXT and refined by full-matrix least-squares methods against F^2 using SHELXL-2019/2.[19;20] All non-hydrogen atoms were refined with anisotropic displacement parameters. All C-bound hydrogen atoms were refined isotropic on calculated positions using a riding model with their U_{iso} values constrained to 1.5 times the U_{eq} of their pivot atoms for terminal sp^3 carbon atoms and 1.2 times for all other carbon atoms. Crystallographic data for the structures reported in this paper have been deposited with the Cambridge Crystallographic Data Centre.[21] CCDC 2473002 contain the supplementary

crystallographic data for this paper. These data can be obtained free of charge from The Cambridge Crystallographic Data Centre via www.ccdc.cam.ac.uk/structures. This report and the CIF file were generated using FinalCif.[22]

Table 16. Crystal data and structure refinement for Werz_MG528_a

**Compound**	**9**
CCDC number	2473002
Empirical formula	$C_{22}H_{20}O_4$
Formula weight	348.38
Temperature [K]	100(2)
Crystal system	triclinic
Space group (number)	$P\bar{1}$ (2)
a [Å]	6.5073(13)
b [Å]	7.6713(14)
c [Å]	18.211(4)
α [°]	99.787(7)
β [°]	92.633(7)
γ [°]	99.396(9)
Volume [Å^3]	881.3(3)
Z	2
ρ_{calc} [gcm^{-3}]	1.313
μ [mm^{-1}]	0.090
F(000)	368
Crystal size [mm^3]	0.047×0.112×0.255
Crystal colour	colourless
Crystal shape	block
Radiation	MoK_α (λ=0.71073 Å)
2θ range [°]	4.55 to 56.81 (0.75 Å)
Index ranges	$-8 \le h \le 8$<br>$-10 \le k \le 10$<br>$0 \le l \le 24$
Reflections collected	4419
Independent reflections	4419<br>R_{int} = 0.0704<br>R_{sigma} = 0.0220
Completeness to θ = 25.242°	99.9 %
Data / Restraints / Parameters	4419 / 0 / 239
Absorption correction T_{min}/T_{max} (method)	0.6613 / 0.7457 (multi-scan)
Goodness-of-fit on F^2	1.081
Final *R* indexes [$I \ge 2\sigma(I)$]	R_1 = 0.0481<br>wR_2 = 0.1215
Final *R* indexes [all data]	R_1 = 0.0580<br>wR_2 = 0.1277
Largest peak/hole [eÅ^{-3}]	0.28/−0.25

Table 17. Atomic coordinates and U_{eq} [Å^2] for Werz_MG528_a

Atom	*x*	*y*	*z*	U_{eq}
O1	1.03338(15)	0.66875(14)	0.58476(6)	0.0257(2)
H1	1.0947(19)	0.6305(19)	0.5438(11)	0.039
O2	0.77696(16)	0.44326(14)	0.53325(6)	0.0279(2)
O3	0.45525(18)	0.05096(15)	0.59008(6)	0.0328(3)
O4	0.29941(18)	0.13243(16)	0.49250(6)	0.0337(3)
H4	0.389(3)	0.064(3)	0.4661(7)	0.051
C5	0.4518(2)	0.50650(18)	0.70283(7)	0.0209(3)
C6	0.6770(2)	0.46254(19)	0.69509(8)	0.0241(3)
H6A	0.761105	0.481735	0.743370	0.029
H6B	0.680014	0.342093	0.665649	0.029
C7	0.7296(2)	0.61743(19)	0.65044(8)	0.0228(3)
H7	0.798485	0.732077	0.683271	0.027
C8	0.4918(2)	0.60999(19)	0.63594(8)	0.0231(3)
H8A	0.433039	0.538697	0.586799	0.028
H8B	0.449244	0.729529	0.644034	0.028
C9	0.8477(2)	0.57041(19)	0.58343(8)	0.0232(3)
C10	0.4253(2)	0.63574(18)	0.77342(7)	0.0217(3)
C11	0.5768(2)	0.6900(2)	0.83286(8)	0.0285(3)
H11	0.705200	0.646246	0.830218	0.034
C12	0.5421(3)	0.8079(2)	0.89614(9)	0.0352(4)
H12	0.647063	0.844195	0.936320	0.042
C13	0.3563(3)	0.8727(2)	0.90111(9)	0.0351(4)
H13	0.332584	0.951205	0.944954	0.042
C14	0.2044(3)	0.8226(2)	0.84188(9)	0.0310(3)
H14	0.077183	0.868156	0.844581	0.037
C15	0.2400(2)	0.70524(19)	0.77846(8)	0.0259(3)
H15	0.136225	0.671763	0.737820	0.031
C16	0.2803(2)	0.34588(18)	0.68718(8)	0.0234(3)
C17	0.1890(2)	0.23386(19)	0.61146(8)	0.0268(3)
H17	0.109282	0.302842	0.581894	0.032

C18	0.0428(2)	0.1188(2)	0.65841(9)	0.0307(3)
H18A	−0.107748	0.121604	0.649462	0.037
H18B	0.068716	−0.005618	0.655784	0.037
C19	0.1493(2)	0.24954(19)	0.72669(8)	0.0258(3)
C20	0.3280(2)	0.13096(19)	0.56418(8)	0.0267(3)
C21	0.1077(2)	0.25905(19)	0.80573(9)	0.0276(3)
C22	0.2670(3)	0.3185(2)	0.86267(9)	0.0324(3)
H22	0.406972	0.349025	0.850296	0.039
C23	0.2239(3)	0.3335(2)	0.93693(10)	0.0418(4)
H23	0.333919	0.375888	0.975089	0.050
C24	0.0209(3)	0.2870(3)	0.95591(11)	0.0444(4)
H24	−0.008547	0.298606	1.006952	0.053
C25	−0.1381(3)	0.2239(2)	0.90036(11)	0.0412(4)
H25	−0.277188	0.191263	0.913284	0.049
C26	−0.0961(2)	0.2078(2)	0.82536(10)	0.0335(3)
H26	−0.206056	0.161862	0.787437	0.040

U_{eq} is defined as 1/3 of the trace of the orthogonalised U_{ij} tensor.

Table 18. Anisotropic displacement parameters [Å^2] for Werz_MG528_a. The anisotropic displacement factor exponent takes the form: $-2\pi^2[\,h^2(a^*)^2U_{11} + k^2(b^*)^2U_{22} + \ldots + 2hka^*b^*U_{12}\,]$

Atom	U_{11}	U_{22}	U_{33}	U_{23}	U_{13}	U_{12}
O1	0.0206(5)	0.0297(5)	0.0255(5)	0.0008(4)	0.0056(4)	0.0037(4)
O2	0.0241(5)	0.0320(5)	0.0246(5)	−0.0019(4)	0.0053(4)	0.0026(4)
O3	0.0383(6)	0.0358(6)	0.0266(5)	0.0011(4)	0.0016(4)	0.0179(5)
O4	0.0400(6)	0.0410(6)	0.0226(5)	−0.0006(4)	0.0013(4)	0.0209(5)
C5	0.0208(6)	0.0234(6)	0.0191(6)	0.0030(5)	0.0030(5)	0.0059(5)
C6	0.0223(6)	0.0288(7)	0.0230(7)	0.0056(5)	0.0030(5)	0.0086(5)
C7	0.0206(6)	0.0272(7)	0.0207(6)	0.0030(5)	0.0040(5)	0.0057(5)
C8	0.0212(6)	0.0287(7)	0.0206(6)	0.0050(5)	0.0032(5)	0.0070(5)
C9	0.0213(6)	0.0270(7)	0.0224(6)	0.0047(5)	0.0028(5)	0.0066(5)
C10	0.0243(6)	0.0204(6)	0.0204(6)	0.0037(5)	0.0044(5)	0.0032(5)
C11	0.0295(7)	0.0288(7)	0.0258(7)	0.0019(6)	−0.0012(6)	0.0051(6)

C12	0.0448(9)	0.0330(8)	0.0240(7)	−0.0019(6)	−0.0042(6)	0.0049(7)
C13	0.0496(9)	0.0295(7)	0.0239(7)	−0.0031(6)	0.0073(7)	0.0070(7)
C14	0.0342(8)	0.0276(7)	0.0321(8)	0.0017(6)	0.0103(6)	0.0092(6)
C15	0.0267(7)	0.0266(7)	0.0241(7)	0.0013(5)	0.0037(5)	0.0062(5)
C16	0.0234(6)	0.0230(6)	0.0230(7)	−0.0010(5)	0.0022(5)	0.0066(5)
C17	0.0270(7)	0.0263(7)	0.0257(7)	−0.0026(5)	0.0012(5)	0.0086(5)
C18	0.0253(7)	0.0270(7)	0.0366(8)	−0.0027(6)	0.0042(6)	0.0037(5)
C19	0.0243(6)	0.0223(6)	0.0306(7)	0.0020(5)	0.0039(5)	0.0058(5)
C20	0.0281(7)	0.0254(7)	0.0247(7)	−0.0024(5)	−0.0002(5)	0.0068(5)
C21	0.0297(7)	0.0224(6)	0.0323(8)	0.0073(5)	0.0085(6)	0.0056(5)
C22	0.0357(8)	0.0299(7)	0.0323(8)	0.0102(6)	0.0055(6)	0.0017(6)
C23	0.0547(11)	0.0398(9)	0.0302(8)	0.0105(7)	0.0029(7)	0.0014(8)
C24	0.0599(12)	0.0434(10)	0.0350(9)	0.0152(8)	0.0171(8)	0.0114(8)
C25	0.0407(9)	0.0411(9)	0.0491(11)	0.0192(8)	0.0221(8)	0.0116(7)
C26	0.0298(8)	0.0312(8)	0.0423(9)	0.0122(7)	0.0092(6)	0.0060(6)

Table 19. Bond lengths and angles for Werz_MG528_a

Atom–Atom	Length [Å]
O1–C9	1.3129(17)
O1–H1	0.89(2)
O2–C9	1.2288(17)
O3–C20	1.2302(18)
O4–C20	1.3122(18)
O4–H4	0.94(2)
C5–C16	1.4992(19)
C5–C10	1.5194(18)
C5–C6	1.5632(18)
C5–C8	1.5724(19)
C6–C7	1.5528(19)
C6–H6A	0.9900
C6–H6B	0.9900
C7–C9	1.4957(18)
C7–C8	1.5475(19)
C7–H7	1.0000
C8–H8A	0.9900
C8–H8B	0.9900
C10–C11	1.390(2)
C10–C15	1.3962(19)
C11–C12	1.390(2)
C11–H11	0.9500
C12–C13	1.381(2)
C12–H12	0.9500
C13–C14	1.388(2)
C13–H13	0.9500
C14–C15	1.392(2)
C14–H14	0.9500
C15–H15	0.9500
C16–C19	1.350(2)
C16–C17	1.5323(19)

C17–C20	1.5021(19)
C17–C18	1.572(2)
C17–H17	1.0000
C18–C19	1.517(2)
C18–H18A	0.9900
C18–H18B	0.9900
C19–C21	1.469(2)
C21–C22	1.394(2)
C21–C26	1.401(2)
C22–C23	1.383(2)
C22–H22	0.9500
C23–C24	1.386(3)
C23–H23	0.9500
C24–C25	1.379(3)
C24–H24	0.9500
C25–C26	1.394(2)
C25–H25	0.9500
C26–H26	0.9500

**Atom–Atom–Atom**	**Angle [°]**
C9–O1–H1	109.5
C20–O4–H4	109.5
C16–C5–C10	112.40(11)
C16–C5–C6	114.72(11)
C10–C5–C6	115.05(11)
C16–C5–C8	115.10(11)
C10–C5–C8	110.50(11)
C6–C5–C8	86.68(9)
C7–C6–C5	89.73(10)
C7–C6–H6A	113.7
C5–C6–H6A	113.7
C7–C6–H6B	113.7
C5–C6–H6B	113.7
H6A–C6–H6B	110.9
C9–C7–C8	116.58(12)
C9–C7–C6	113.94(11)
C8–C7–C6	87.92(10)
C9–C7–H7	112.1
C8–C7–H7	112.1
C6–C7–H7	112.1
C7–C8–C5	89.59(10)
C7–C8–H8A	113.7
C5–C8–H8A	113.7
C7–C8–H8B	113.7
C5–C8–H8B	113.7
H8A–C8–H8B	111.0
O2–C9–O1	123.58(12)
O2–C9–C7	121.37(12)
O1–C9–C7	114.96(12)
C11–C10–C15	118.31(13)
C11–C10–C5	123.40(12)
C15–C10–C5	118.28(12)
C10–C11–C12	120.52(14)
C10–C11–H11	119.7
C12–C11–H11	119.7
C13–C12–C11	120.61(15)
C13–C12–H12	119.7
C11–C12–H12	119.7
C12–C13–C14	119.79(14)
C12–C13–H13	120.1
C14–C13–H13	120.1
C13–C14–C15	119.49(14)
C13–C14–H14	120.3
C15–C14–H14	120.3
C14–C15–C10	121.27(14)

C14–C15–H15	119.4	O3–C20–C17	123.18(14)
C10–C15–H15	119.4	O4–C20–C17	113.78(13)
C19–C16–C5	137.48(13)	C22–C21–C26	118.45(15)
C19–C16–C17	94.00(12)	C22–C21–C19	121.61(13)
C5–C16–C17	128.50(12)	C26–C21–C19	119.94(14)
C20–C17–C16	118.60(12)	C23–C22–C21	120.87(16)
C20–C17–C18	114.52(12)	C23–C22–H22	119.6
C16–C17–C18	85.40(11)	C21–C22–H22	119.6
C20–C17–H17	111.9	C22–C23–C24	120.28(18)
C16–C17–H17	111.9	C22–C23–H23	119.9
C18–C17–H17	111.9	C24–C23–H23	119.9
C19–C18–C17	86.22(11)	C25–C24–C23	119.71(16)
C19–C18–H18A	114.3	C25–C24–H24	120.1
C17–C18–H18A	114.3	C23–C24–H24	120.1
C19–C18–H18B	114.3	C24–C25–C26	120.41(16)
C17–C18–H18B	114.3	C24–C25–H25	119.8
H18A–C18–H18B	111.4	C26–C25–H25	119.8
		C25–C26–C21	120.22(16)
C16–C19–C21	136.04(14)	C25–C26–H26	119.9
C16–C19–C18	94.32(12)	C21–C26–H26	119.9
C21–C19–C18	129.61(13)		
O3–C20–O4	123.04(13)		

Table 20. Torsion angles for Werz_MG528_a

Atom–Atom–Atom–Atom	Torsion Angle [°]
C16–C5–C6–C7	134.55(12)
C10–C5–C6–C7	−92.74(12)
C8–C5–C6–C7	18.37(10)
C5–C6–C7–C9	−136.93(12)
C5–C6–C7–C8	−18.66(10)
C9–C7–C8–C5	134.38(12)
C6–C7–C8–C5	18.54(10)
C16–C5–C8–C7	−134.25(11)
C10–C5–C8–C7	97.11(12)
C6–C5–C8–C7	−18.44(10)
C8–C7–C9–O2	−42.07(19)
C6–C7–C9–O2	58.16(18)
C8–C7–C9–O1	141.16(13)
C6–C7–C9–O1	−118.61(13)

C16–C5–C10–C11	123.84(14)
C6–C5–C10–C11	−9.95(19)
C8–C5–C10–C11	−106.07(15)
C16–C5–C10–C15	−56.96(16)
C6–C5–C10–C15	169.25(12)
C8–C5–C10–C15	73.14(15)
C15–C10–C11–C12	1.2(2)
C5–C10–C11–C12	−179.58(14)
C10–C11–C12–C13	0.2(2)
C11–C12–C13–C14	−1.3(3)
C12–C13–C14–C15	1.0(2)
C13–C14–C15–C10	0.4(2)
C11–C10–C15–C14	−1.5(2)
C5–C10–C15–C14	179.25(13)
C10–C5–C16–C19	−28.2(2)
C6–C5–C16–C19	105.74(19)
C8–C5–C16–C19	−155.91(16)
C10–C5–C16–C17	149.62(13)
C6–C5–C16–C17	−76.43(17)
C8–C5–C16–C17	21.93(19)
C19–C16–C17–C20	−117.35(14)
C5–C16–C17–C20	64.1(2)
C19–C16–C17–C18	−1.85(11)
C5–C16–C17–C18	179.61(14)
C20–C17–C18–C19	121.06(13)
C16–C17–C18–C19	1.65(10)
C5–C16–C19–C21	1.9(3)
C17–C16–C19–C21	−176.38(17)
C5–C16–C19–C18	−179.78(16)
C17–C16–C19–C18	1.92(12)
C17–C18–C19–C16	−1.87(11)
C17–C18–C19–C21	176.60(15)
C16–C17–C20–O3	40.0(2)
C18–C17–C20–O3	−58.6(2)
C16–C17–C20–O4	−140.81(14)
C18–C17–C20–O4	120.66(15)
C16–C19–C21–C22	−34.6(3)
C18–C19–C21–C22	147.56(16)
C16–C19–C21–C26	145.31(17)
C18–C19–C21–C26	−32.5(2)
C26–C21–C22–C23	−2.6(2)
C19–C21–C22–C23	177.36(15)
C21–C22–C23–C24	0.9(3)
C22–C23–C24–C25	0.6(3)
C23–C24–C25–C26	−0.4(3)
C24–C25–C26–C21	−1.3(3)
C22–C21–C26–C25	2.8(2)
C19–C21–C26–C25	−177.18(14)

9.5 1,3'-Bis(4-bromophenyl)-1'-(methoxycarbonyl)-[1,1'-bi(cyclobutan)]-2'-ene-3-carboxylic acid (10).

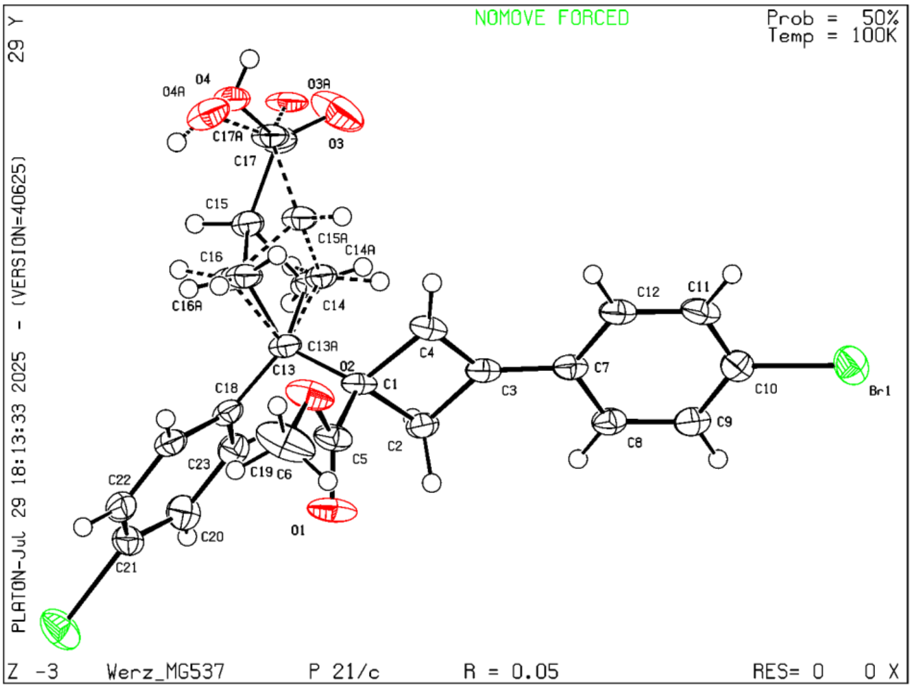

Crystals were obtained at room temperature by gas-phase diffusion of *n*-hexane into a solution of the compound dissolved in dichloromethane. A colourless, block-shaped crystal was mounted on a MiTeGen micromount with perfluoroether oil. Data for Werz_MG537 were collected from a shock-cooled single crystal at 100(2) K on a Bruker D8 VENTURE dual wavelength Mo/Cu three-circle diffractometer with a microfocus sealed X-ray tube using a mirror optics as monochromator and a Bruker PHOTON III detector. The diffractometer was equipped with an Oxford Cryostream 800 low temperature device and used MoK_{α} radiation (λ = 0.71073 Å). All data were integrated with SAINT V8.41 and a multi-scan absorption correction using SADABS 2016/2 was applied.[17;18]The structure was solved by direct methods with SHELXD and refined by full-matrix least-squares methods against F^2 using SHELXL-2019/2.[19;20] All non-hydrogen atoms were refined with anisotropic displacement parameters. All hydrogen atoms were refined isotropic on calculated positions using a riding model with their U_{iso} values constrained to 1.5 times the U_{eq} of their pivot atoms for terminal sp^3 carbon atoms and 1.2 times for all other carbon atoms. Disordered moieties were refined using bond lengths restraints and displacement parameter restraints. Crystallographic data for the structures reported in this paper have been deposited with the

Cambridge Crystallographic Data Centre.[21] CCDC 2476959 contain the supplementary crystallographic data for this paper. These data can be obtained free of charge from The Cambridge Crystallographic Data Centre via www.ccdc.cam.ac.uk/structures. This report and the CIF file were generated using FinalCif.[22]

Table 21. Crystal data and structure refinement for Werz_MG537

**Compound**	**10**
CCDC number	2476959
Empirical formula	$C_{23}H_{20}Br_2O_4$
Formula weight	520.21
Temperature [K]	100(2)
Crystal system	monoclinic
Space group (number)	$P2_1/c$ (14)
a [Å]	20.433(5)
b [Å]	9.030(2)
c [Å]	11.857(3)
α [°]	90
β [°]	106.600(14)
γ [°]	90
Volume [Å^3]	2096.5(9)
Z	4
ρ_{calc} [gcm^{-3}]	1.648
μ [mm^{-1}]	3.894
F(000)	1040
Crystal size [mm^3]	0.156×0.192×0.364
Crystal colour	colourless
Crystal shape	block
Radiation	MoK_α (λ=0.71073 Å)
2θ range [°]	3.58 to 56.56 (0.75 Å)

Index ranges	$-27 \leq h \leq 27$<br>$-12 \leq k \leq 12$<br>$-15 \leq l \leq 15$
Reflections collected	258244
Independent reflections	5185<br>R_{int} = 0.0657<br>R_{sigma} = 0.0181
Completeness to θ = 25.242°	99.6 %
Data / Restraints / Parameters	5185 / 315 / 323
Absorption correction T_{min}/T_{max} (method)	0.5770 / 0.7461 (multi-scan)
Goodness-of-fit on F^2	1.080
Final *R* indexes [$I \geq 2\sigma(I)$]	R_1 = 0.0469<br>wR_2 = 0.1030
Final *R* indexes [all data]	R_1 = 0.0475<br>wR_2 = 0.1033
Largest peak/hole [eÅ^{-3}]	1.02/−1.39

Refinement details for Werz_MG537

Refined as a 2-component twin.

Table 22. Atomic coordinates and U_{eq} [Å^2] for Werz_MG537

Atom	*x*	*y*	*z*	U_{eq}
Br1	1.02155(3)	0.05401(6)	0.22138(5)	0.03933(13)
Br2	0.49808(3)	−0.36411(6)	0.69184(5)	0.04490(14)
O1	0.78624(19)	−0.1704(4)	0.7536(3)	0.0415(9)
O2	0.8165(2)	0.0420(4)	0.8499(3)	0.0406(9)
C1	0.7652(2)	0.0604(4)	0.6458(4)	0.0256(8)
C2	0.7677(2)	−0.0208(5)	0.5314(4)	0.0271(9)
H2A	0.779197	−0.127320	0.542545	0.033
H2B	0.726662	−0.005049	0.463866	0.033
C3	0.8261(2)	0.0748(4)	0.5300(4)	0.0259(8)
C4	0.8238(2)	0.1566(5)	0.6284(4)	0.0290(9)
H4	0.848546	0.239164	0.668829	0.035
C5	0.7899(2)	−0.0374(5)	0.7532(4)	0.0296(9)
C6	0.8431(4)	−0.0428(7)	0.9569(5)	0.0585(19)
H6A	0.805275	−0.090484	0.978331	0.088
H6B	0.867490	0.023425	1.020577	0.088
H6C	0.874586	−0.118643	0.944232	0.088
C7	0.8720(2)	0.0735(4)	0.4561(4)	0.0258(8)
C8	0.8619(2)	−0.0287(5)	0.3641(4)	0.0278(8)
H8	0.824237	−0.094730	0.349429	0.033
C9	0.9057(2)	−0.0357(5)	0.2938(4)	0.0303(9)
H9	0.898510	−0.106278	0.231969	0.036
C10	0.9603(2)	0.0622(5)	0.3153(4)	0.0285(9)
C11	0.9720(2)	0.1648(5)	0.4065(4)	0.0330(10)
H11	1.009863	0.230279	0.420897	0.040
C12	0.9282(2)	0.1707(5)	0.4759(4)	0.0303(9)
H12	0.935962	0.241232	0.537776	0.036
C13	0.6967(2)	0.1366(4)	0.6435(4)	0.0241(7)

C14	0.6697(3)	0.2485(5)	0.5406(4)	0.0244(11)
H14A	0.704150	0.276563	0.500411	0.029
H14B	0.626499	0.217233	0.483035	0.029
C15	0.6599(3)	0.3648(5)	0.6285(4)	0.0258(9)
H15	0.611212	0.369794	0.629733	0.031
C16	0.7040(4)	0.2676(5)	0.7325(5)	0.0306(15)
H16A	0.751450	0.303136	0.765454	0.037
H16B	0.682051	0.249037	0.795612	0.037
C17	0.6886(4)	0.5170(6)	0.6217(5)	0.0278(10)
O3	0.7065(4)	0.5644(6)	0.5412(4)	0.0657(18)
O4	0.6934(3)	0.5951(5)	0.7185(4)	0.0311(10)
H4A	0.710(4)	0.667(8)	0.715(3)	0.047
C13A	0.6967(2)	0.1366(4)	0.6435(4)	0.0241(7)
C14A	0.688(3)	0.262(3)	0.549(2)	0.024(4)
H14C	0.716475	0.248304	0.494839	0.029
H14D	0.640271	0.284674	0.506125	0.029
C15A	0.7196(18)	0.370(2)	0.652(3)	0.027(3)
H15A	0.770654	0.369295	0.675565	0.033
C16A	0.689(4)	0.264(3)	0.730(4)	0.026(4)
H16C	0.718350	0.251559	0.811450	0.032
H16D	0.641246	0.286067	0.727321	0.032
C17A	0.691(4)	0.524(3)	0.642(3)	0.031(5)
O3A	0.665(2)	0.586(3)	0.552(3)	0.035(6)
O4A	0.683(3)	0.569(5)	0.744(3)	0.041(8)
H4AA	0.66(3)	0.52(5)	0.76(3)	0.061
C18	0.6451(2)	0.0190(5)	0.6532(4)	0.0242(8)
C19	0.6014(2)	−0.0471(5)	0.5540(4)	0.0267(8)
H19	0.601900	−0.014888	0.478008	0.032
C20	0.5568(2)	−0.1596(5)	0.5642(5)	0.0320(9)
H20	0.526712	−0.203098	0.495973	0.038
C21	0.5571(2)	−0.2070(5)	0.6751(4)	0.0293(9)
C22	0.6000(2)	−0.1454(5)	0.7755(4)	0.0289(9)
H22	0.599818	−0.179796	0.851146	0.035

C23	0.6434(2)	−0.0323(4)	0.7637(4)	0.0271(8)
H23	0.672702	0.011581	0.832528	0.033

U_{eq} is defined as 1/3 of the trace of the orthogonalised U_{ij} tensor.

Table 23. Anisotropic displacement parameters [Å^2] for Werz_MG537. The anisotropic displacement factor exponent takes the form: $-2\pi^2[\,h^2(a^*)^2U_{11} + k^2(b^*)^2U_{22} + \ldots + 2hka^*b^*U_{12}\,]$

Atom	U_{11}	U_{22}	U_{33}	U_{23}	U_{13}	U_{12}
Br1	0.0285(2)	0.0437(3)	0.0460(3)	0.0135(2)	0.0111(2)	0.0051(2)
Br2	0.0277(2)	0.0493(3)	0.0551(3)	0.0110(2)	0.0076(2)	−0.0106(2)
O1	0.0416(19)	0.0169(15)	0.054(2)	0.0093(14)	−0.0048(17)	−0.0013(14)
O2	0.054(2)	0.0278(17)	0.0299(17)	0.0064(14)	−0.0036(16)	−0.0105(15)
C1	0.0274(19)	0.0130(16)	0.033(2)	0.0011(15)	0.0031(16)	−0.0013(15)
C2	0.025(2)	0.0178(18)	0.038(2)	−0.0023(17)	0.0083(18)	0.0007(15)
C3	0.0214(18)	0.0161(17)	0.036(2)	0.0008(16)	0.0009(16)	−0.0006(15)
C4	0.029(2)	0.0177(18)	0.035(2)	0.0043(17)	0.0024(18)	−0.0037(15)
C5	0.0231(19)	0.0213(19)	0.038(2)	0.0042(18)	−0.0012(18)	−0.0030(16)
C6	0.068(4)	0.050(3)	0.039(3)	0.018(3)	−0.015(3)	−0.021(3)
C7	0.0223(19)	0.0178(18)	0.033(2)	0.0009(16)	0.0009(16)	0.0004(15)
C8	0.0252(19)	0.0206(19)	0.034(2)	−0.0031(17)	0.0034(17)	−0.0018(15)
C9	0.027(2)	0.023(2)	0.038(2)	−0.0014(18)	0.0036(18)	0.0005(17)
C10	0.0190(18)	0.028(2)	0.035(2)	0.0094(18)	0.0027(16)	0.0063(16)
C11	0.027(2)	0.0201(19)	0.047(3)	0.0040(18)	0.003(2)	−0.0048(16)

C12	0.028(2)	0.0179(18)	0.041(2)	0.0001(17)	0.0034(18)	−0.0021(16)
C13	0.034(2)	0.0149(15)	0.0235(17)	−0.0002(13)	0.0076(16)	0.0020(14)
C14	0.033(3)	0.0185(18)	0.0231(18)	0.0033(15)	0.0092(19)	0.0042(18)
C15	0.033(2)	0.0185(17)	0.026(2)	−0.0001(15)	0.0085(18)	0.0014(15)
C16	0.052(4)	0.0158(19)	0.025(2)	0.0012(15)	0.013(2)	0.001(2)
C17	0.034(2)	0.0210(19)	0.024(2)	0.0038(16)	0.001(2)	0.0023(18)
O3	0.115(5)	0.050(3)	0.037(2)	−0.0049(19)	0.029(3)	−0.041(3)
O4	0.039(2)	0.0162(17)	0.038(2)	−0.0014(16)	0.0109(18)	−0.0014(16)
C13A	0.034(2)	0.0149(15)	0.0235(17)	−0.0002(13)	0.0076(16)	0.0020(14)
C14A	0.032(9)	0.016(5)	0.026(5)	0.001(4)	0.009(6)	0.002(6)
C15A	0.039(7)	0.016(4)	0.026(6)	0.001(5)	0.008(6)	0.000(5)
C16A	0.039(9)	0.015(5)	0.025(5)	−0.001(4)	0.009(6)	0.001(7)
C17A	0.045(9)	0.015(5)	0.032(8)	0.002(5)	0.011(7)	0.001(7)
O3A	0.057(14)	0.011(10)	0.035(8)	0.004(7)	0.011(9)	0.000(9)
O4A	0.058(19)	0.028(14)	0.033(8)	0.000(9)	0.010(10)	0.014(14)
C18	0.028(2)	0.0188(18)	0.0262(19)	0.0003(15)	0.0078(16)	0.0068(15)
C19	0.0240(18)	0.028(2)	0.027(2)	0.0022(17)	0.0050(15)	0.0049(16)
C20	0.0192(18)	0.035(2)	0.039(2)	0.002(2)	0.0037(18)	0.0007(17)
C21	0.0197(18)	0.025(2)	0.044(2)	0.0028(18)	0.0093(17)	−0.0002(16)
C22	0.031(2)	0.026(2)	0.033(2)	0.0049(17)	0.0130(17)	0.0070(17)
C23	0.032(2)	0.0202(18)	0.029(2)	−0.0005(17)	0.0091(18)	0.0051(16)

Table 24. Bond lengths and angles for Werz_MG537

Atom–Atom	Length [Å]
Br1–C10	1.900(5)
Br2–C21	1.909(4)
O1–C5	1.203(5)

O2–C5	1.330(6)	C15–C16	1.571(8)
O2–C6	1.449(6)	C15–H15	1.0000
C1–C5	1.513(6)	C16–H16A	0.9900
C1–C4	1.539(6)	C16–H16B	0.9900
C1–C13A	1.554(6)	C17–O3	1.195(8)
C1–C13	1.554(6)	C17–O4	1.326(6)
C1–C2	1.555(6)	O4–H4A	0.74(7)
C2–C3	1.478(6)	C13A–C18	1.523(6)
C2–H2A	0.9900	C13A–C14A	1.567(19)
C2–H2B	0.9900	C13A–C16A	1.573(19)
C3–C4	1.393(7)	C14A–C15A	1.54(2)
C3–C7	1.455(6)	C14A–H14C	0.9900
C4–H4	0.9500	C14A–H14D	0.9900
C6–H6A	0.9800	C15A–C17A	1.506(19)
C6–H6B	0.9800	C15A–C16A	1.58(2)
C6–H6C	0.9800	C15A–H15A	1.0000
C7–C8	1.398(6)	C16A–H16C	0.9900
C7–C12	1.411(6)	C16A–H16D	0.9900
C8–C9	1.389(7)	C17A–O3A	1.19(2)
C8–H8	0.9500	C17A–O4A	1.327(19)
C9–C10	1.388(6)	O4A–H4AA	0.74(7)
C9–H9	0.9500	C18–C19	1.393(6)
C10–C11	1.392(7)	C18–C23	1.401(6)
C11–C12	1.378(7)	C19–C20	1.392(6)
C11–H11	0.9500	C19–H19	0.9500
C12–H12	0.9500	C20–C21	1.381(7)
C13–C18	1.523(6)	C20–H20	0.9500
C13–C14	1.559(6)	C21–C22	1.378(6)
C13–C16	1.563(6)	C22–C23	1.385(6)
C14–C15	1.532(7)	C22–H22	0.9500
C14–H14A	0.9900	C23–H23	0.9500
C14–H14B	0.9900		
C15–C17	1.505(8)		

Atom–Atom–Atom	Angle [°]
C5–O2–C6	115.4(4)
C5–C1–C4	111.0(3)
C5–C1–C13A	110.8(4)
C4–C1–C13A	118.6(3)
C5–C1–C13	110.8(4)
C4–C1–C13	118.6(3)
C5–C1–C2	111.9(3)
C4–C1–C2	86.0(3)
C13A–C1–C2	116.5(3)
C13–C1–C2	116.5(3)
C3–C2–C1	87.8(3)
C3–C2–H2A	114.0
C1–C2–H2A	114.0
C3–C2–H2B	114.0
C1–C2–H2B	114.0
H2A–C2–H2B	111.2
C4–C3–C7	133.7(4)
C4–C3–C2	94.6(4)
C7–C3–C2	131.6(4)
C3–C4–C1	91.5(3)
C3–C4–H4	134.2
C1–C4–H4	134.2
O1–C5–O2	123.1(4)
O1–C5–C1	125.4(4)
O2–C5–C1	111.5(4)
O2–C6–H6A	109.5
O2–C6–H6B	109.5
H6A–C6–H6B	109.5
O2–C6–H6C	109.5
H6A–C6–H6C	109.5
H6B–C6–H6C	109.5
C8–C7–C12	118.2(4)
C8–C7–C3	119.9(4)
C12–C7–C3	121.9(4)
C9–C8–C7	121.5(4)
C9–C8–H8	119.3
C7–C8–H8	119.3
C10–C9–C8	118.8(4)
C10–C9–H9	120.6
C8–C9–H9	120.6
C9–C10–C11	121.2(4)
C9–C10–Br1	119.7(4)
C11–C10–Br1	119.1(3)
C12–C11–C10	119.6(4)
C12–C11–H11	120.2
C10–C11–H11	120.2
C11–C12–C7	120.9(4)
C11–C12–H12	119.6
C7–C12–H12	119.6
C18–C13–C1	109.2(3)
C18–C13–C14	114.4(4)
C1–C13–C14	114.6(4)
C18–C13–C16	114.3(4)
C1–C13–C16	114.3(4)
C14–C13–C16	89.0(3)
C15–C14–C13	89.9(3)
C15–C14–H14A	113.7
C13–C14–H14A	113.7
C15–C14–H14B	113.7
C13–C14–H14B	113.7
H14A–C14–H14B	110.9
C17–C15–C14	117.4(4)
C17–C15–C16	114.1(4)

C14–C15–C16	89.6(3)
C17–C15–H15	111.3
C14–C15–H15	111.3
C16–C15–H15	111.3
C13–C16–C15	88.3(4)
C13–C16–H16A	113.9
C15–C16–H16A	113.9
C13–C16–H16B	113.9
C15–C16–H16B	113.9
H16A–C16–H16B	111.1
O3–C17–O4	122.5(6)
O3–C17–C15	125.6(5)
O4–C17–C15	111.9(5)
C17–O4–H4A	109.5
C18–C13A–C1	109.2(3)
C18–C13A–C14A	128.5(19)
C1–C13A–C14A	104(2)
C18–C13A–C16A	105.1(19)
C1–C13A–C16A	125(3)
C14A–C13A–C16A	85.5(15)
C15A–C14A–C13A	87.8(14)
C15A–C14A–H14C	114.0
C13A–C14A–H14C	114.0
C15A–C14A–H14D	114.0
C13A–C14A–H14D	114.0
H14C–C14A–H14D	111.2
C17A–C15A–C14A	117(3)
C17A–C15A–C16A	113(3)
C14A–C15A–C16A	86.1(17)
C17A–C15A–H15A	112.6
C14A–C15A–H15A	112.6
C16A–C15A–H15A	112.6
C13A–C16A–C15A	86.2(14)
C13A–C16A–H16C	114.3
C15A–C16A–H16C	114.3
C13A–C16A–H16D	114.3
C15A–C16A–H16D	114.3
H16C–C16A–H16D	111.4
O3A–C17A–O4A	123(3)
O3A–C17A–C15A	124(2)
O4A–C17A–C15A	111(2)

C17A–O4A–H4AA	109.5	C22–C21–C20	121.7(4)
C19–C18–C23	117.8(4)	C22–C21–Br2	118.3(3)
C19–C18–C13A	121.8(4)	C20–C21–Br2	119.9(3)
C23–C18–C13A	120.3(4)	C21–C22–C23	118.5(4)
C19–C18–C13	121.8(4)	C21–C22–H22	120.7
C23–C18–C13	120.3(4)	C23–C22–H22	120.7
C20–C19–C18	121.2(4)	C22–C23–C18	121.8(4)
C20–C19–H19	119.4	C22–C23–H23	119.1
C18–C19–H19	119.4	C18–C23–H23	119.1
C21–C20–C19	118.9(4)		
C21–C20–H20	120.5		
C19–C20–H20	120.5		

Table 25. Torsion angles for Werz_MG537

Atom–Atom–Atom–Atom	Torsion Angle [°]		
C5–C1–C2–C3	−108.2(4)	C13A–C1–C5–O1	103.7(6)
C4–C1–C2–C3	2.8(3)	C13–C1–C5–O1	103.7(6)
C13A–C1–C2–C3	122.9(4)	C2–C1–C5–O1	−28.2(7)
C13–C1–C2–C3	122.9(4)	C4–C1–C5–O2	57.8(5)
C1–C2–C3–C4	−3.1(3)	C13A–C1–C5–O2	−76.1(5)
C1–C2–C3–C7	172.8(5)	C13–C1–C5–O2	−76.1(5)
C7–C3–C4–C1	−172.7(5)	C2–C1–C5–O2	152.0(4)
C2–C3–C4–C1	3.1(3)	C4–C3–C7–C8	177.4(5)
C5–C1–C4–C3	108.9(4)	C2–C3–C7–C8	2.9(7)
C13A–C1–C4–C3	−121.1(4)	C4–C3–C7–C12	−1.1(7)
C13–C1–C4–C3	−121.1(4)	C2–C3–C7–C12	−175.5(4)
C2–C1–C4–C3	−3.0(3)	C12–C7–C8–C9	0.2(6)
C6–O2–C5–O1	1.7(8)	C3–C7–C8–C9	−178.3(4)
C6–O2–C5–C1	−178.5(5)	C7–C8–C9–C10	−0.6(7)
C4–C1–C5–O1	−122.4(5)	C8–C9–C10–C11	0.8(6)
		C8–C9–C10–Br1	179.5(3)
		C9–C10–C11–C12	−0.8(7)

Br1–C10–C11–C12	−179.4(3)	C4–C1–C13A–C14A	35.5(13)
C10–C11–C12–C7	0.4(7)		
C8–C7–C12–C11	−0.2(6)	C2–C1–C13A–C14A	−65.0(13)
C3–C7–C12–C11	178.3(4)		
C5–C1–C13–C18	−54.2(4)	C5–C1–C13A–C16A	71.0(14)
C4–C1–C13–C18	175.8(4)		
C2–C1–C13–C18	75.2(4)	C4–C1–C13A–C16A	−59.0(14)
C5–C1–C13–C14	175.9(4)		
C4–C1–C13–C14	45.9(5)	C2–C1–C13A–C16A	−159.6(14)
C2–C1–C13–C14	−54.6(5)		
C5–C1–C13–C16	75.2(4)	C18–C13A–C14A–C15A	134.1(18)
C4–C1–C13–C16	−54.8(5)		
C2–C1–C13–C16	−155.3(4)	C1–C13A–C14A–C15A	−96(2)
C18–C13–C14–C15	102.7(4)		
C1–C13–C14–C15	−130.0(4)	C16A–C13A–C14A–C15A	28(3)
C16–C13–C14–C15	−13.6(4)		
C13–C14–C15–C17	130.7(5)	C13A–C14A–C15A–C17A	−142(3)
C13–C14–C15–C16	13.6(4)		
C18–C13–C16–C15	−103.1(4)	C13A–C14A–C15A–C16A	−28(3)
C1–C13–C16–C15	130.0(4)		
C14–C13–C16–C15	13.3(4)	C18–C13A–C16A–C15A	−156(2)
C17–C15–C16–C13	−133.7(5)		
C14–C15–C16–C13	−13.5(4)	C1–C13A–C16A–C15A	77(3)
C14–C15–C17–O3	15.3(11)		
C16–C15–C17–O3	118.2(9)	C14A–C13A–C16A–C15A	−28(3)
C14–C15–C17–O4	−163.4(5)		
C16–C15–C17–O4	−60.4(7)	C17A–C15A–C16A–C13A	146(3)
C5–C1–C13A–C18	−54.2(4)		
C4–C1–C13A–C18	175.8(4)	C14A–C15A–C16A–C13A	28(3)
C2–C1–C13A–C18	75.2(4)		
C5–C1–C13A–C14A	165.5(12)	C14A–C15A–C17A–O3A	−28(8)

C16A–C15A–C17A–O3A	−126(7)
C14A–C15A–C17A–O4A	137(6)
C16A–C15A–C17A–O4A	40(7)
C1–C13A–C18–C19	−90.7(5)
C14A–C13A–C18–C19	37(2)
C16A–C13A–C18–C19	134(3)
C1–C13A–C18–C23	85.4(5)
C14A–C13A–C18–C23	−146.8(19)
C16A–C13A–C18–C23	−50(3)
C1–C13–C18–C19	−90.7(5)
C14–C13–C18–C19	39.3(5)
C16–C13–C18–C19	139.9(5)
C1–C13–C18–C23	85.4(5)
C14–C13–C18–C23	−144.6(4)
C16–C13–C18–C23	−44.0(6)
C23–C18–C19–C20	0.6(6)
C13A–C18–C19–C20	176.8(4)
C13–C18–C19–C20	176.8(4)
C18–C19–C20–C21	−0.9(6)
C19–C20–C21–C22	0.3(7)
C19–C20–C21–Br2	−178.6(3)
C20–C21–C22–C23	0.5(6)
Br2–C21–C22–C23	179.4(3)
C21–C22–C23–C18	−0.8(6)
C19–C18–C23–C22	0.2(6)
C13A–C18–C23–C22	−176.0(4)
C13–C18–C23–C22	−176.0(4)

11. References

1 R. M. Bychek, V. Hutskalova, Y. P. Bas, O. A. Zaporozhets, S. Zozulya, V. V. Levterov and P. K. Mykhailiuk, *J. Org. Chem.*, 2019, **84**, 15106.

2 K. Livingstone, K. Siebold, S. Meyer, V. Martín-Heras, C. G. Daniliuc and R. Gilmour, *ACS Catal.*, 2022, **12**, 14507.

3 T. V. T. Nguyen, A. Bossonnet, M. D. Wodrich and J. Waser, *J. Am. Chem. Soc.*, 2023, **145**, 25411.

4 Q. Fu, S. Cao, J. Wang, X. Lv, H. Wang, X. Zhao and Z. Jiang, *J. Am. Chem. Soc.*, 2024, **146**, 8372.

5 S.-L. Lin, Y.-H. Chen, H.-H. Liu, S.-H. Xiang and B. Tan, *J. Am. Chem. Soc.*, 2023, **145**, 21152.

6 J. Jeong, S. Cao, H.-J. Kang, H. Yoon, J. Lee, S. Shin, D. Kim and S. Hong, *J. Am. Chem. Soc.*, 2024, **146**, 27830.

7 Y. Liang, F. Paulus, C. G. Daniliuc and F. Glorius, *Angew. Chem. Int. Ed.*, 2023, **62**, e202305043.

8 R. Guo, Y.-C. Chang, L. Herter, C. Salome, S. E. Braley, T. C. Fessard and M. K. Brown, *J. Am. Chem. Soc.*, 2022, **144**, 7988.

9 D. A. Knyazev, M. George and D. B. Werz, *Chem. Sci.*, 2025, **16**, 8588.

10 M. Golfmann, M. Reinhold, J. D. Steen, M. S. Deike, B. Rodemann, C. Golz, S. Crespi and J. C. L. Walker, *ACS Catal.*, 2024, **14**, 13987.

11 a) F. Neese, *WIREs Comput. Mol. Sci.*, 2025, **15**; b) F. Neese, *WIREs Comput. Mol. Sci.*, 2012, **2**, 73; c) F. Neese, *WIREs Comput. Mol. Sci.*, 2025, **15**.

12 S. Grimme, J. Antony, S. Ehrlich and H. Krieg, *J. Chem. Phys.*, 2010, **132**, 154104.

13 M. Garcia-Ratés and F. Neese, *J. Comput. Chem.*, 2020, **41**, 922.

14 V. Barone and M. Cossi, *J. Phys. Chem. A*, 1998, **102**, 1995.

15 a) Y. Zhao and D. G. Truhlar, *Theor. Chem. Acc.*, 2008, **120**, 215; b) F. Weigend and R. Ahlrichs, *Phys. Chem. Chem. Phys.*, 2005, **7**, 3297; c) S. Grimme, A. Hansen, S. Ehlert and J.-M. Mewes, *J. Chem. Phys.*, 2021, **154**, 64103.

16 S. Grimme, *Chem. Eur. J.*, 2012, **18**, 9955.

17 Bruker, *SAINT, V8.40B, Bruker AXS Inc., Madison, Wisconsin, USA.*

18 L. Krause, R. Herbst-Irmer, G. M. Sheldrick and D. Stalke, *J. Appl. Crystallogr.*, 2015, **48**, 3.

19 G. M. Sheldrick, *Acta crystallographica. Section A, Foundations and advances*, 2015, **71**, 3.

20 G. M. Sheldrick, *Acta crystallographica. Section C, Structural chemistry*, 2015, **71**, 3.

21 C. R. Groom, I. J. Bruno, M. P. Lightfoot and S. C. Ward, *Acta crystallographica Section B, Structural science, crystal engineering and materials*, 2016, **72**, 171.

22 D. Kratzert, *FinalCif, V139, https://dkratzert.de/finalcif.html.*

23 D. Kratzert and I. Krossing, *J. Appl. Crystallogr.*, 2018, **51**, 928.
